# Automatic Detection and Counting of Wheat Spikelet Using Semi-Automatic Labeling and Deep Learning

**DOI:** 10.3389/fpls.2022.872555

**Published:** 2022-05-30

**Authors:** Ruicheng Qiu, Yong He, Man Zhang

**Affiliations:** ^1^College of Biosystem Engineering and Food Science, Zhejiang University, Hangzhou, China; ^2^Key Laboratory of Spectroscopy Sensing, Ministry of Agriculture and Rural Affairs, Zhejiang University, Hangzhou, China; ^3^Key Laboratory of Modern Precision Agriculture System Integration Research, Ministry of Education, China Agricultural University, Beijing, China

**Keywords:** wheat spikelet, spike, annotation, deep learning, computer vision

## Abstract

The number of wheat spikelets is an important phenotypic trait and can be used to assess the grain yield of the wheat crop. However, manual counting of spikelets is time-consuming and labor-intensive. To develop a cost-effective and highly efficient phenotyping system for counting the number of spikelets under laboratory conditions, methods based on imaging processing techniques and deep learning were proposed to accurately detect and count spikelets from color images of wheat spikes captured at the grain filling stage. An unsupervised learning-based method was first developed to automatically detect and label spikelets from spike color images and build the datasets for the model training. Based on the constructed datasets, a deep convolutional neural network model was retrained using transfer learning to detect the spikelets. Testing results showed that the root mean squared errors, relative root mean squared errors, and the coefficients of determination between the automatic and manual counted spikelets for four wheat lines were 0.62, 0.58, 0.54, and 0.77; 3.96, 3.73, 3.34, and 4.94%; and 0.73, 0.78, 0.84, and 0.67, respectively. We demonstrated that the proposed methods can effectively estimate the number of wheat spikelets, which improves the counting efficiency of wheat spikelets and contributes to the analysis of the developmental characteristics of wheat spikes.

## Introduction

Breeding of high-yield wheat (*Triticum aestivum* L.) cultivars is crucial for ensuring food safety, as wheat is a staple food in the world. Researchers have reported that wheat yield is highly associated with several phenotypic traits, such as spike number per unit area (SNA), grain number per spike (GNS), and thousand-grain weights. It is broadly agreed that improving the SNA and GNS of wheat is important to increase the wheat yield (Vahamidis et al., 2019). A wheat spike consists of many spikelets and a rachis, and each spikelet contains two or more florets. In general, only 1–3 florets can become fertile florets and develop into grains. Improvements in spikelet and floret (floret primordia and fertile floret) numbers contribute significantly to an increment in GNS (García et al., 2014). In addition, the number of spikelets, fertile florets, and grains would enable the calculation of the spikelet fertility, fertile floret proportion, and grain/fertile floret ratio to further assess the spike characteristics (Guo et al., 2018). Therefore, counting the number of spikes, spikelets, and florets during the breeding process is of great importance for screening high-yield wheat cultivars. However, conventional methods for the manual phenotyping of wheat spike traits are time-consuming and labor-intensive, which in turn delays progress in breeding programs. Consequently, it is urgent to develop an efficient method to accurately and quickly acquire phenotypic traits of wheat spikes. Recent advances in computer vision technology provide innovative ways to assess phenotypic traits of wheat spikes, and techniques like color, X-ray, and computed tomography (CT) imaging have been investigated.

Color imaging has been widely applied to measure the phenotypic traits of crops (Qiu et al., 2018). With respect to wheat spikes, many researchers focused on the automatic detection and counting of spikes. The color features (e.g., red, green, and blue), texture features (e.g., gray level co-occurrence matrix), and image features (e.g., contour and edge) were selected or combined to train a model (e.g., support vector machine and neural network models) using supervised learning, to facilitate the detection of wheat spikes (Li et al., 2017; Zhou et al., 2018; Xu et al., 2020). In addition, Genaev et al. (2019) analyzed wheat spike images in the laboratory and estimated the morphometric traits of spikes, such as spike length, width, and circularity. Liu et al. (2017); Kaya and Saritas (2019) developed a real-time sorting system and an application program, respectively, to identify each grain and count the number of wheat grains. However, color imaging has not been adequately exploited for the detection and counting of wheat spikelets. Researchers have also explored the usefulness of deep learning techniques for measuring spike phenotypic traits. Concerning target detection, deep convolutional neural network (DCNN) models have been widely implemented. The Faster Region-based Convolutional Network (RCNN) model (Madec et al., 2019) and Mask RCNN model (Qiu et al., 2019) were retrained to detect wheat spikes from color images captured in field conditions. Pound et al. (2017) developed a DCNN model and presented the Annotated Crop Image Dataset (ACID) to count wheat spikes and spikelets. Besides, Khoroshevsky et al. (2021) developed a deep neural network to detect and count the number of spikelets per spike in a field. Chen et al. (2021) proposed a method to train deep networks on data with reduced numbers of annotations to count wheat spikelets. TasselNetv2 (Xiong et al., 2019b), SpikeletFCN (Alkhudaydi et al., 2019), SpikeSegNet (Misra et al., 2020), and DeepCount (Sadeghi-Tehran et al., 2019) were also developed to detect and count wheat spikes or spikelets in the field and laboratory. These studies revealed that deep learning techniques are promising for detecting and counting wheat spikes and spikelets. However, one of the current challenges is to obtain a large number of labeled datasets to train the deep learning models. Manual labeling is a heavy burden. Furthermore, some researchers applied adversarial learning to leaf and spikelet countings with unsupervised training (Giuffrida et al., 2019; Hu et al., 2019; Ayalew et al., 2020), but the models are difficult to train.

Furthermore, X-ray and CT imaging have been explored to non-destructively measure phenotypic traits of wheat spikes. Both X-ray and CT imaging can measure the inner structures of spikes and acquire information on grains (Duan et al., 2011; Xiong et al., 2019a; Yu et al., 2021), which can be further used to distinguish and count the filled spikelets (Zhou et al., 2021). As for CT imaging, it can reconstruct spikes in three dimensions as well. Thus, wheat spike and grain traits, including spike height, grain number, grain width, height, and depth, can be extracted and measured (Hughes et al., 2017; Xiong et al., 2019a). Although X-ray and CT imaging can provide considerable inner and spatial information about wheat spikes, they are expensive and the imaging systems are complicated, which limits their application.

Currently, color images are easily acquired at a low cost, and most studies focus on the automatic measurement and counting of wheat spikes using color imaging. But studies regarding the automatic detection of wheat spikelets have not been well reported, and the usefulness of color imaging for counting spikelets needs to be further investigated. Deep learning has been proved very helpful for the phenotyping of wheat spikes. However, manual annotation is a laborious and tedious process. Therefore, this study focuses on detecting and counting spikelets using color imaging and deep learning techniques. Four common wheat lines were selected as the objects of this study, and the color images of their spikes were collected in the laboratory. The objectives of this study were to (1) develop an unsupervised learning-based method to automatically detect and label spikelets from spike color images and build the datasets for DCNN model training and (2) train a DCNN model that can detect and count spikelets. The counting results of spikelets will be compared with manual countings to evaluate the performance of the proposed methods.

## Materials and Methods

The counting system mainly included the following steps: image collection, image annotation, spikelet detection and counting, and performance evaluation. Several algorithms were used and developed to detect spikelets and build datasets in the section of image annotation, and a DCNN model was trained using a deep learning method to detect spikelets in the section of spikelet detection and counting. Each step is described in detail in the following sections. A desktop computer with AMD R5-2600 CPU, NVIDIA GeForce GTX 1070, 8G RAM, and Windows 10 64-bit system was utilized to process the images of wheat spikes and test the proposed methods. Image annotation and spikelet detection and counting were conducted using Matlab R2021b software and Tensorflow, respectively.

### Image Collection

**Figure 1:**
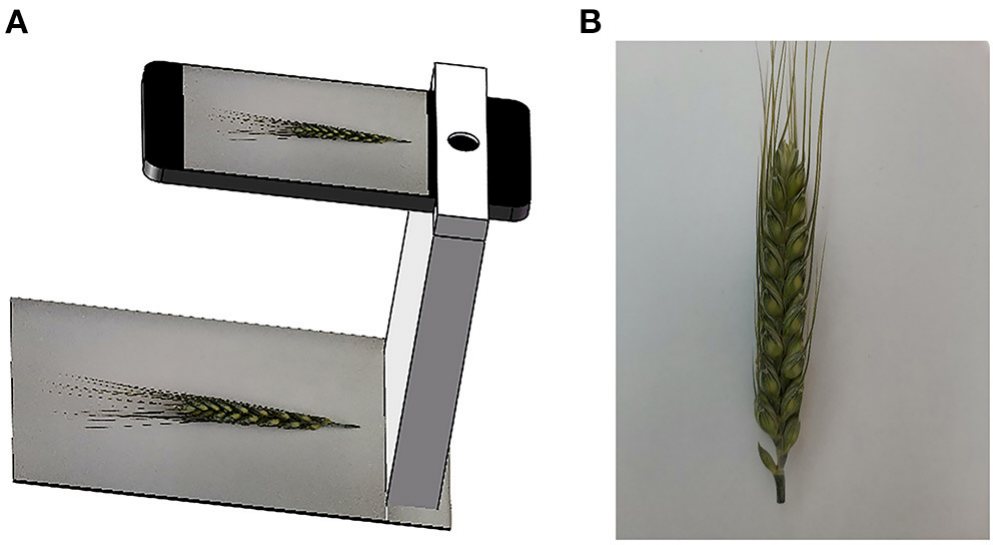
Schematic diagram of wheat spike image capturing system and captured images. **(A)** Image capturing system and **(B)** a spike image. A spike example of Shiyou 20 was used in Figures 1–4 to explain our work.

The experiment was conducted in a field station in the city of Hengshui (38°21′ N, 115°65′ E), Hebei Province, China. The cultivated wheat lines were Shiyou 20, Shannong 25, Liangxing 99, and Shenmai 818, which were largely cultivated in the North China Plain. Their wheat spikes with awns have different shapes and colors. Wheat spike samples for each wheat line were randomly selected and collected on May 18, 2021, and most of the wheat spikes were at the grain filling stage of development. At this stage, the wheat spikelets have basically been formed, wheat awns and glumes have not begun to senesce, and the color contrast of spikelet and awn is great (Qiu et al., 2019). The collected wheat spikes were transferred to the laboratory on the same day. Wheat spikes were placed on a flat board, and a HUAWEI Honor 9X PRO smartphone was used to capture spike images, as illustrated in Figure 1A. A spike was captured twice by rolling 180° to acquire its images on two sides. The captured spike images (Figure 1B) have a 4,000 × 3,000 pixel resolution in a JPEG (Joint Photographic Experts Group) compressed format. In the image, the apical spikelet is located on the top of the spike, other spikelets are located on both sides of the spike rachis, and the glumes of spikelets are clear. Spikelets can be counted by detecting the glumes in the spike image. Finally, more than 300 spike images for each wheat line were collected.

### Image Annotation

In this study, an unsupervised learning method based on the watershed algorithm was developed to annotate the spike images. In addition, a DCNN model was trained to optimize the annotation. The method contains several steps, which are described in detail in the following sections. The proposed labeling method was implemented to process the collected spike images of four wheat lines.

#### Image Preprocessing

**Figure 2:**
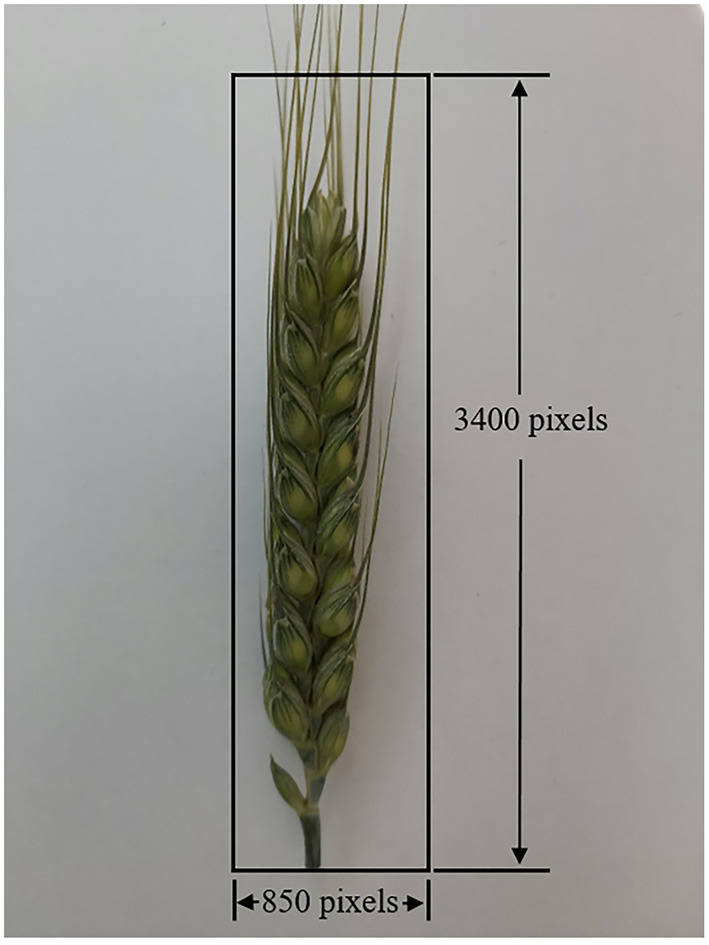
Schematic diagram of region of interest.

The color images of raw spike have high resolution, which is not conducive to the subsequent image processing. Therefore, a region of interest (ROI) was set to the color images of the wheat spike, to reduce the computation. The ROI is shown in Figure 2, and the whole spike was extracted following the steps depicted in the figure. Moreover, the extracted images were downsampled using the bicubic method to reduce image resolutions. The scale factor was set to 0.3, and the spike images were reshaped to 256 × 1,021 pixel resolution.

#### Spike Extraction

**Figure 3:**
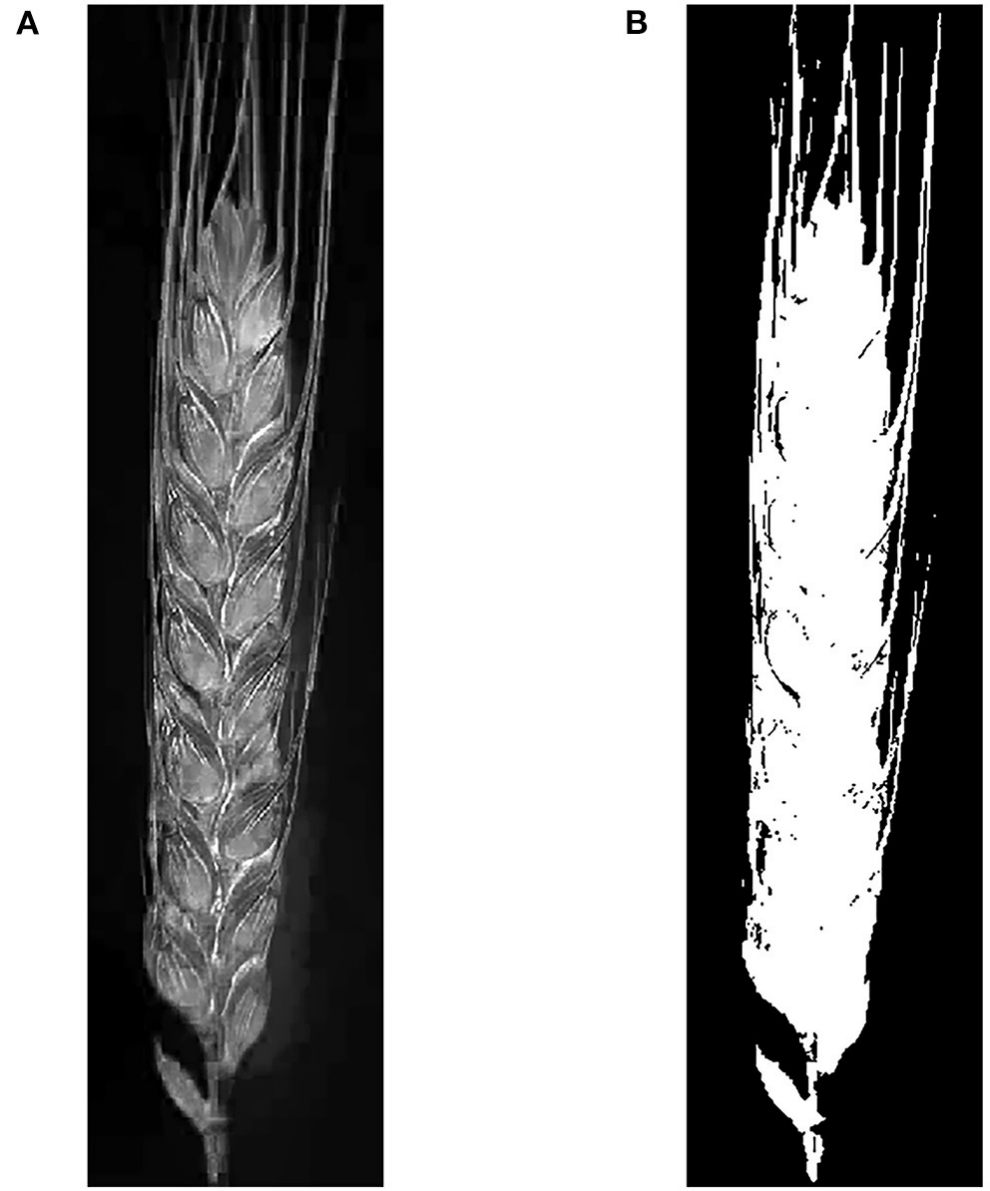
Spike segmentation. **(A)** Gray (S component) image of wheat spike and **(B)** segmentation results of wheat spike.

It is of significance to segment spikes from the spike images in advance, to minimize detection areas and improve the detection efficiencies and precision of spikelets. Although the color characteristics of the spike and background in the spike images are different, the color characteristics of spikes for different wheat lines are not uniform in RGB (Red, Green, and Blue) color space, which makes it difficult to apply RGB color features to segment and extract spikes. After tests, HSV (Hue, Saturation, and Value) color space was applied to process the reshaped images after image preprocessing. S component of the spike in the color image was calculated using the function “rgb2hsv” provided by Matlab to generate the gray image to represent the color characteristics of the spike, as shown in Figure 3A. The contrast between spike and background is stark so that the spike can be detected and extracted accurately. Otsu's algorithm was implemented to process the gray image of the spike and generate its binary image (Figure 3B). In the binary image, the pixel values of spike and background are 1 and 0, respectively. As shown in Figure 3B, the spike was successfully extracted.

#### Spikelet Segmentation and Annotation

The color characteristics of spikelets are different from the other parts of spikes, which contributes to the detection and segmentation of spikelets. Specifically, some parts of glumes are prominent in the color images.

**Figure 4:**
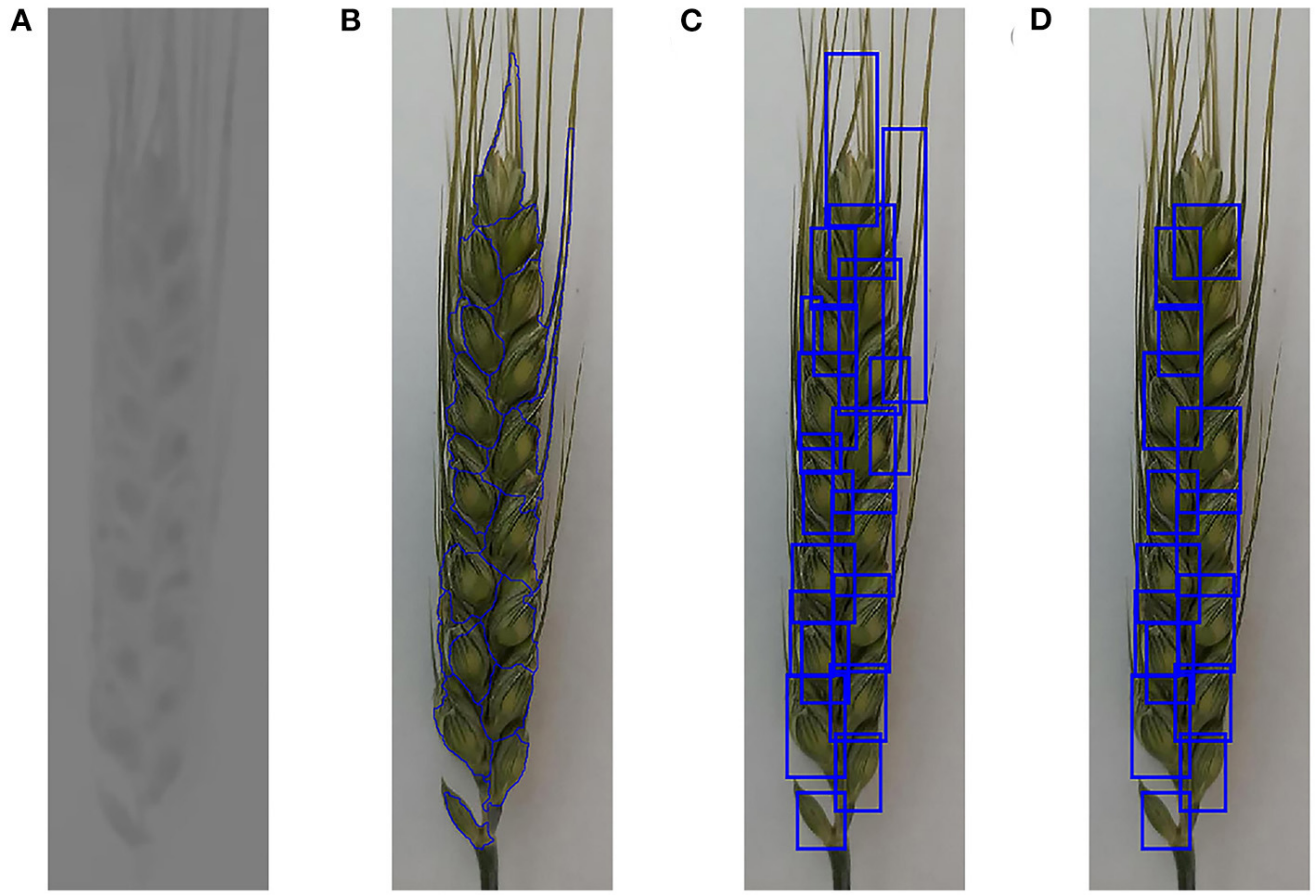
Spikelet annotation. **(A)** Gray (Cb component) image of wheat spike, **(B)** segmentation results of watershed algorithm, **(C)** MER for all candidate spikelet regions, and **(D)** optimized MER for candidate spikelet regions.

The color images of the spike were transformed into several color spaces, such as RGB, HSV, and YCbCr, to find suitable features for spikelet segmentation. Testing results showed that the color features of spikelets and their boundaries were significant in YCbCr color space. Cb component of the spike in the color image was calculated using the function “rgb2ycbcr” provided by Matlab to generate the gray image to represent the color characteristics of spikelets. Then, a bilateral filter was used to enhance the contrast between the spikelets and their boundaries in the gray image, as shown in Figure 4A. By performing a per-pixel dot product between Figures 4A, 3B, the spike gray image was extracted, and the gray values of ground pixels were set to 0.

In the spike gray image, the values of glumes are lower than that of their surrounding pixels. Based on this characteristic, the watershed algorithm can be used to segment the spikelets from spikes. However, the watershed algorithm usually produces over-segmentation. To improve the segmentation accuracy, a watershed algorithm, marked by a local minimum threshold, was applied to process the extracted gray image of the spike. Tests showed that when the local minimum threshold was set to 3, the obtained results can remove some local minimums and avoid over-segmentation. The segmented boundaries generated by the watershed algorithm can divide the spike into many regions (Figure 4B), which contained many candidate spikelets. After the initial segmentation of the spikelet, the areas and minimum enclosed rectangles (MER) for all the candidate spikelet regions were calculated (Figure 4C). According to the sizes and shapes of spikelets, the regions whose areas were between 1,000 and 5,000 and ratios of length to width for the corresponding MER were in the range of 1–2.5 were reserved, as displayed in Figure 4D. After that, the upper-left coordinates, widths, and lengths of MER were saved to obtain the bounding boxes for the initial labeling of spikelets.

Three hundred spike images of each wheat line were randomly selected from their captured images and initially labeled using the described method. As shown in Figure 4D, there were some mislabeled or unlabeled spikelets. To acquire better labels, the labeling results were manually checked, and 100 spike images of each wheat line annotated with high accuracy were selected to generate the dataset. XML files for all labeled spike images were generated based on the coordinates of MER, which were used for the subsequent dataset construction and DCNN model training.

#### DCNN Model Training

In recent years, some DCNN models were developed and widely implemented for object detection. In the present study, Faster RCNN, proposed by Ren et al. (2017), was selected to detect the spikelets given its high detection accuracy, which can classify objects and realize semantic segmentation of spikelets. A Faster RCNN has two main parts, a regional proposal network (RPN) and a Fast RCNN. The RPN is a type of fully convolutional network and generates many anchor regions as candidate bounding boxes. Each anchor is assessed and scored based on its intersection over union (IOU) ratio with the ground truth. The anchors are classified as positive and negative using a Softmax function, and the bounding box regressions of positive anchors are conducted to obtain corrected region proposals, which are used by Fast RCNN for object detection training. In addition, some proposals may overlap with each other, and non-maximum suppression (NMS) is adopted to reduce the number of proposals. The loss function (*L*) was defined as a function (1).


(1) $$\begin{array}{l} L=L_{rpn\text{\_}cls}+L_{rpn\text{\_}reg}+L_{rcnn\text{\_}cls}+L_{rcnn\text{\_}reg} \end{array}$$

where *L*_*rpn*_*cls*_ and *L*_*rpn*_*reg*_, and *L*_*rcnn*_*cls*_ and *L*_*rcnn*_*reg*_ represent the classification and bounding box regression losses for RPN and Fast RCNN, respectively.

In this study, Faster RCNN was implemented using Tensorflow object detection API (Huang et al., 2017). The model was pre-trained using the COCO dataset. The Inception-V2 model was used to extract features because of its high speed. The scales (i.e., 0.25, 0.5, 1.0, and 2.0) and ratios (i.e., 0.5, 1.0, and 2.0) were set for the anchors. If the values of the IOU ratio with the labeled bounding box were higher than 0.6, the anchors were considered to contain a wheat spikelet. The batch size was set to 1 because it saves memory and computation time. The IOU threshold for NMS was set to 0.6. The momentum was fixed to 1. The initial learning rate (LR) was 0.0005. After 4,000 and 8,000 iterations, the LR dropped to 0.0003 and 0.0001, respectively. The training results were recorded once every 60 s.

Before training, 80 spike images were randomly selected from the annotated images (100 images) for each wheat line to generate the training dataset, and the remaining 20 spike images were used as the validation dataset. One hundred seventy spike images for each wheat line that were not annotated were used to build the testing dataset.

The model was retrained and fine-tuned using transfer learning on the desktop computer with Tensorflow 1.10.0, Anaconda 3.5.2, CUDA 9.0, and Python 3.6.7.

If the IOU between a predicted bounding box and a labeled bounding box is higher than a set threshold, the predicted bounding box is considered as a true positive (*TP*). Otherwise, it is considered as a false positive (*FP*). If a labeled spikelet cannot be detected, it is considered as a false negative (*FN*). Then, the recall and precision can be calculated by functions (2) and (3).


(2) $$\begin{array}{l} recall=\frac{TP}{TP\;+\;FN} \end{array}$$

(3) $$\begin{array}{l} precision=\frac{TP}{TP\;+\;FP} \end{array}$$

The average precision (AP), which is the area under the precision-recall curve, was applied as an indicator to quantify the performance of the trained Faster RCNN model. In this study, the standard IOU threshold value of 0.5 was used, and the AP@0.5IOU was calculated (Madec et al., 2019).

#### Dataset Optimization

After training, the spike images in the training and validation datasets were processed again using the trained DCNN model to detect and label the spikelets. The coordinates and confidence scores of detected bounding boxes were saved. The high confidence scores (up to 1) indicated that the detected boxes most probably contain spikelets. The bounding boxes of detected spikelets whose confidence scores were higher than 0.75 were reserved, which were used to update the image annotation and the training and validation datasets.

As the performance of the watershed algorithm for spikelet segmentation and labeling in Section Spikelet Segmentation and Annotation was not perfect, the training and validation datasets used for DCNN model training contain many mislabeled or unlabeled spikelets, and the bounding boxes of spikelet samples generated by the trained DCNN model were incomplete. Therefore, manual corrections were conducted by removing mislabeled regions and adding new spikelet labels to the updated training and validation datasets.

### Spikelet Detection and Counting

The ultimate goal of this study was to detect and count the wheat spikelets in spike images. The Faster RCNN model was retrained again using the optimized training and validation datasets in Section Dataset Optimization. The batch size was set to 2, and the momentum was adjusted to 0.8, to prevent overfitting. The initial LR was set to 0.0005. After 6,000 and 10,000 iterations, the LR dropped to 0.0001 and 0.00001, respectively. The training results were recorded once every 15 s.

The retrained model was implemented to detect the spikelets in the spike images of the testing dataset. The confidence score threshold of the detected bounding box was set to 0.75. The final results were counted to obtain the spikelet numbers for all wheat lines.

### Performance Evaluation

One hundred seventy spike images of each wheat line in the testing dataset were used to assess the performance of the proposed detection and counting methods for wheat spikelets, which were evaluated using several statistical parameters, including the root mean squared error (*RMSE*), relative *RMSE* (*rRMSE*), and the coefficient of determination (R^2^), as described in the following equations.


(4) $$\begin{array}{l} RMSE=\sqrt{\frac{1}{n}\overset{n}{\underset{i=1}{\sum }}{\left(t_{i}-d_{i}\right)}^{2}} \end{array}$$

(5) $$\begin{array}{l} rRMSE=\sqrt{\frac{1}{n}\overset{n}{\underset{i=1}{\sum }}{\left(\frac{t_{i}-d_{i}}{t_{i}}\right)}^{2}} \end{array}$$

(6) $$\begin{array}{l} R^{2}=1-\frac{\sum_{i=1}^{n}{\left(t_{i}-d_{i}\right)}^{2}}{\sum_{i=1}^{n}{\left(t_{i}-{\bar{t}}_{i}\right)}^{2}} \end{array}$$

where *n* indicates the number of testing images, *t*_*i*_ is the manually counted number of spikelets, *d*_*i*_ is the automatically counted number of spikelets, and ${\bar{t}}_{i}$ is the mean value of *t*_*i*_.

## Results

### Image Annotation Results

#### Spikelet Segmentation and Annotation Results

Although the bounding boxes generated by the water algorithm contain some non-spikelet areas and their boundaries show errors, they incorporate the main parts of spikelets. Some unsatisfactory MER were removed in Section Spikelet Segmentation and Annotation, so that many spikelets were inevitably skipped and not annotated. A manual check was performed only to estimate the labeling results, and the boundaries of bounding boxes were not adjusted during the manual check process.

After manual check and selection, the labeled spikelet numbers of 100 spike images (80 images for the training dataset and 20 images for the validation dataset) for each wheat line are summarized in Table 1. The results indicated that the proposed method can realize the segmentation and detection of spikelets in spike images, even though the color characteristics of wheat lines are different. Also, it is effective for the initial spikelet labeling of different wheat lines. Finally, a total of 5,772 spikelets were roughly labeled. All the labeled spike images are provided in the Supplementary Material.

**Table 1:** The number of spikelets labeled by the watershed algorithm for training and validation datasets.

**Wheat line**	**Training**	**Validation**	**SUM**
Shiyou 20	1,097	267	1,364
Shannong 25	1,142	265	1,407
Liangxing 99	1,095	269	1,364
Shenmai 818	1,304	333	1,637
SUM	4,638	1,134	5,772

#### Model Training and Dataset Optimization Results

**Figure 5:**
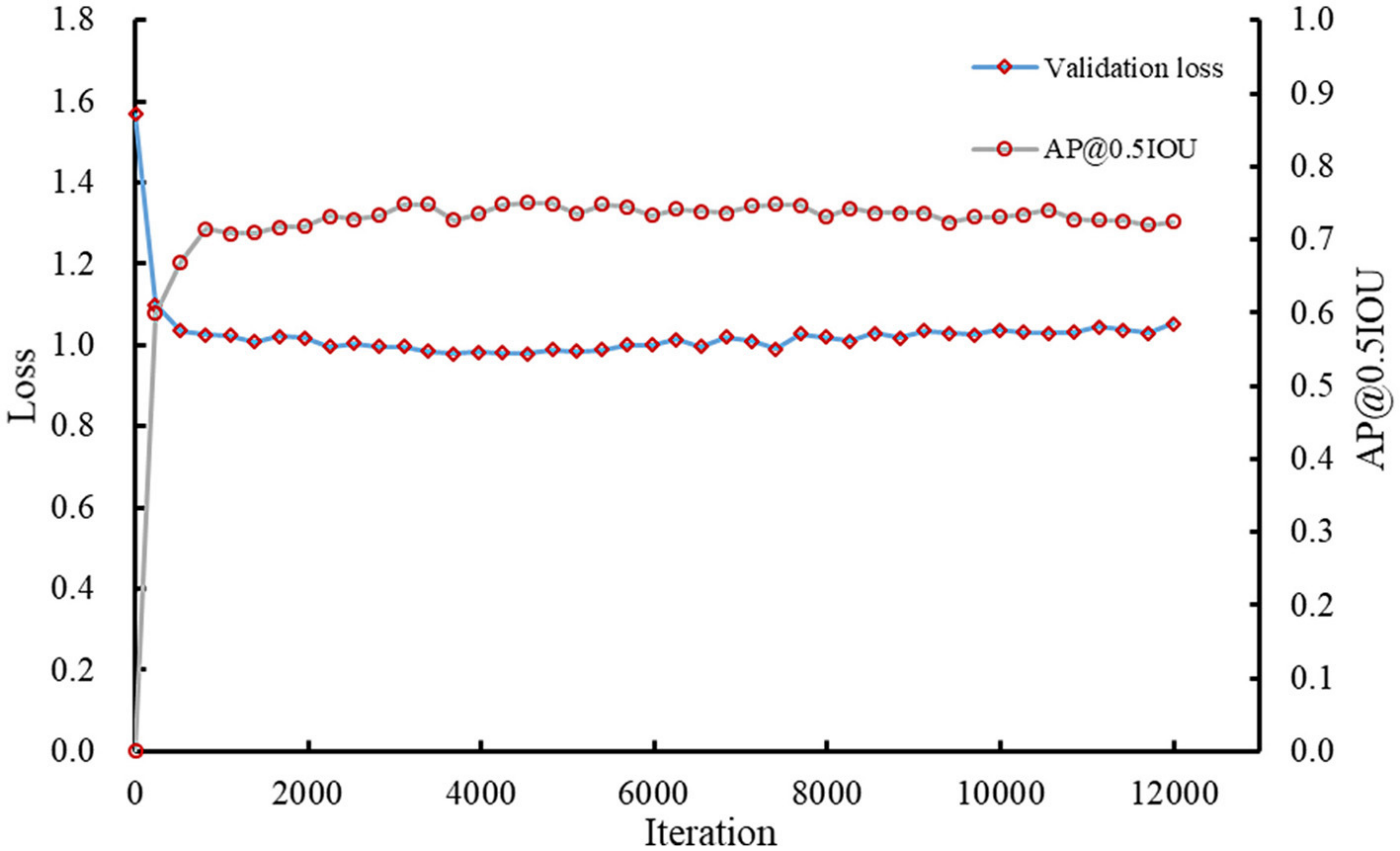
Loss and AP@0.5IOU of the Faster RCNN model for validation.

The Faster RCNN model was trained using the initial labeled spikelet samples. As presented in Table 1, the training and validation datasets contain 320 (80 images × 4 lines) and 80 (20 images × 4 lines) spike images, and 4,638 and 1,134 labeled spikelets, respectively. The loss for the validation dataset was calculated, and the precision (AP@0.5IOU) was selected after each iteration to monitor the training process of the Faster RCNN model. The validation loss and AP@0.5IOU are summarized in Figure 5.

The AP@0.5IOU of the obtained model was not high due to the inaccuracy and imperfection of the initial labeling datasets; therefore, the training was stopped early when the AP@0.5IOU was essentially unchanged, which was about 0.7479 at iteration 5,393. The final loss of the model for the validation dataset was ~0.9886.

The training and validation datasets were processed using the obtained model to detect their spikelets. The labeled spikelet numbers for the wheat lines using the model are also summarized in Table 2. After the DCNN model labeling, the number of labeled spikelets for the training and validation datasets were 3,973 and 955, respectively. Although the number of labeled spikelets declined, the labeling qualities of spikelets were improved. The boundaries of spikelets were flagged more accurately. In the Supplementary Material, the labeled spikelets for each wheat line are detailed.

**Table 2:** The number of labeled spikelets for training and validation datasets.

**Wheat line**	**Labeled by the DCNN model**			**After manual correction**		
	**Training**	**Validation**	**SUM**	**Training**	**Validation**	**SUM**
Shiyou 20	982	224	1,206	1,272	297	1,569
Shannong 25	986	232	1,218	1,238	293	1,531
Liangxing 99	954	227	1,181	1,263	312	1,575
Shenmai 818	1,051	272	1,323	1,251	302	1,553
SUM	3,973	955	4,928	5,024	1,204	6,228

According to the detection results, we found that the robustness of the obtained model for the spikelets at the bottoms of wheat spikes (as the lowest spikelet in Figure 4D) is poor. In practice, these spikelets are sterile and not taken into account. A manual correction was conducted to remove the mislabeled samples. Besides, the model cannot detect all spikelets because some spikelets in the initial datasets were not labeled. Bounding boxes were manually added to handle this situation. The final labeled spikelet samples after manual correction are summarized in Table 2, and the training and validation datasets contain 5,024 and 1,204 labeled spikelets, respectively.

### Spikelet Detection and Counting Results

#### Model Training for Spikelet Detection

**Figure 6:**
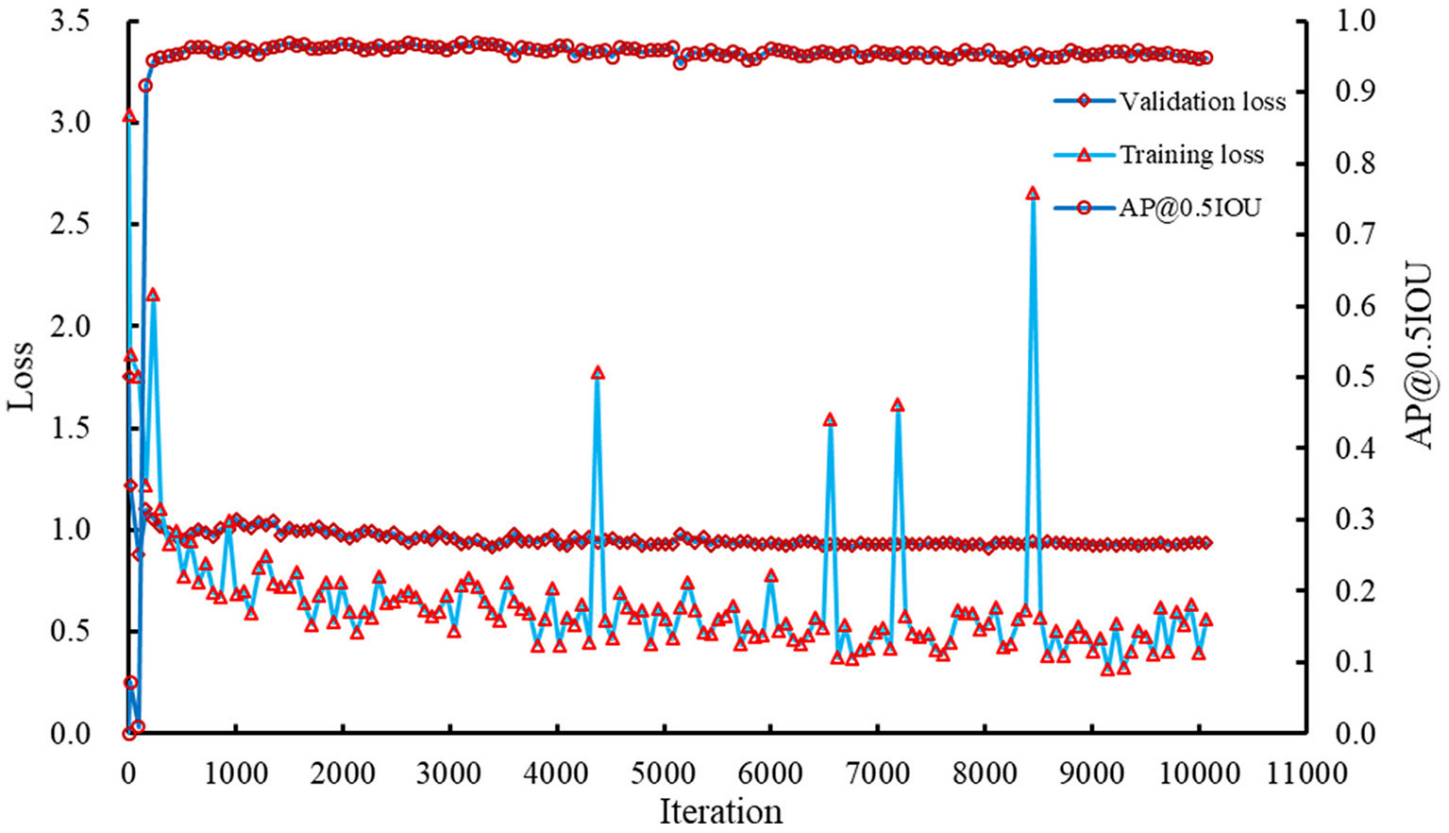
Losses and AP@0.5IOU of the Faster RCNN model during the training process.

The Faster RCNN model was retrained using the corrected training and validation datasets. The recorded losses and AP@0.5IOU are summarized in Figure 6. Results showed that the training and validation losses of the model decreased slowly with increasing iterations. AP@0.5IOU reached a high value after a few hundred iterations and basically kept stable. Combing AP@0.5IOU with the training and validation losses as the indicators, the model saved at iteration 8,026 was selected as the reference model, which was used to detect and count the spikelets in spike images. At this iteration, the training loss, validation loss, and AP@0.5IOU were about 0.5389, 0.9108, and 0.9582, respectively.

#### Spikelet Detection and Counting Results

**Figure 7:**
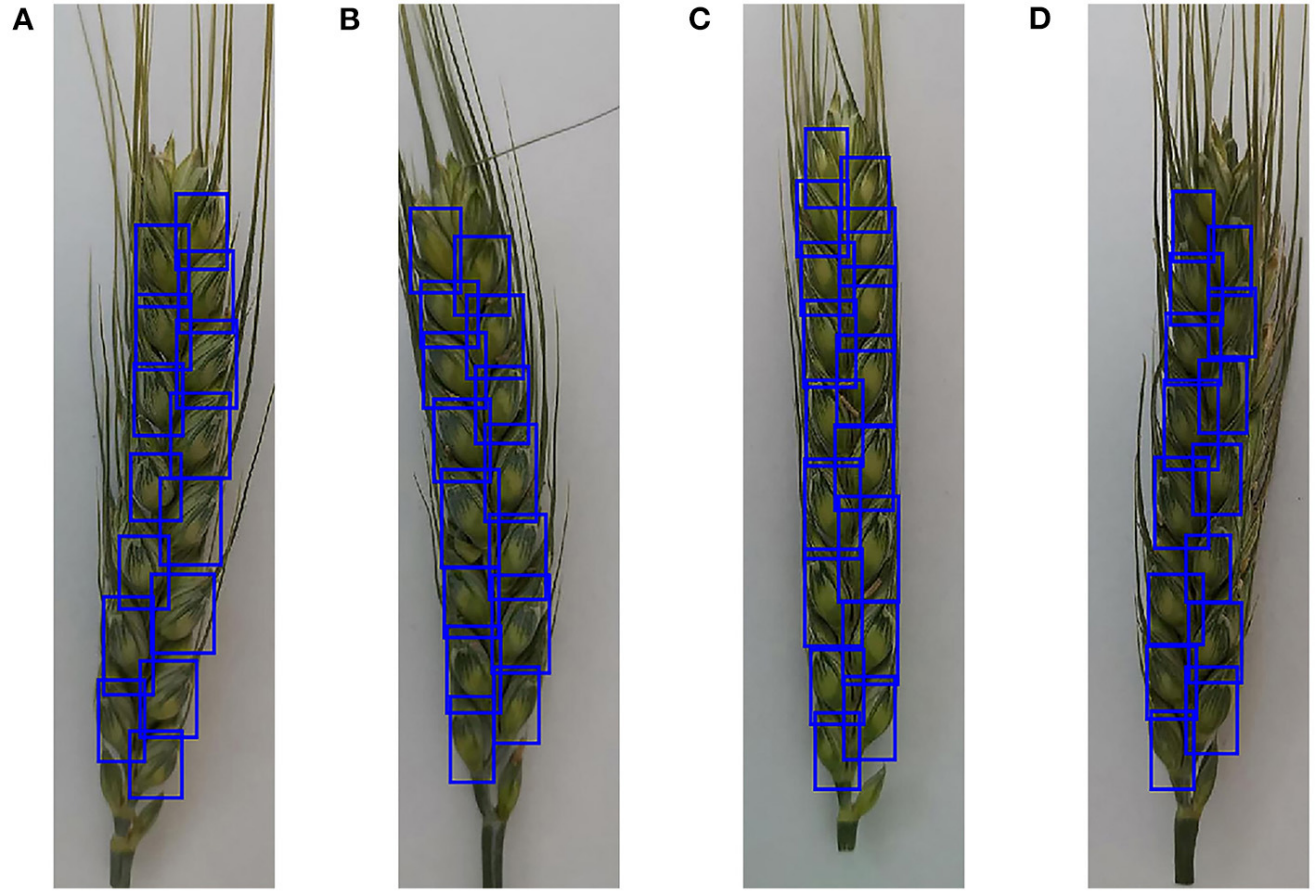
Examples to show the detection results of spikelets for different wheat lines. **(A)** Detection results of Shiyou 20, **(B)** detection results of Shannong 25, **(C)** detection results of Liangxing 99, and **(D)** detection results of Shenmai 818.

**Figure 8:**
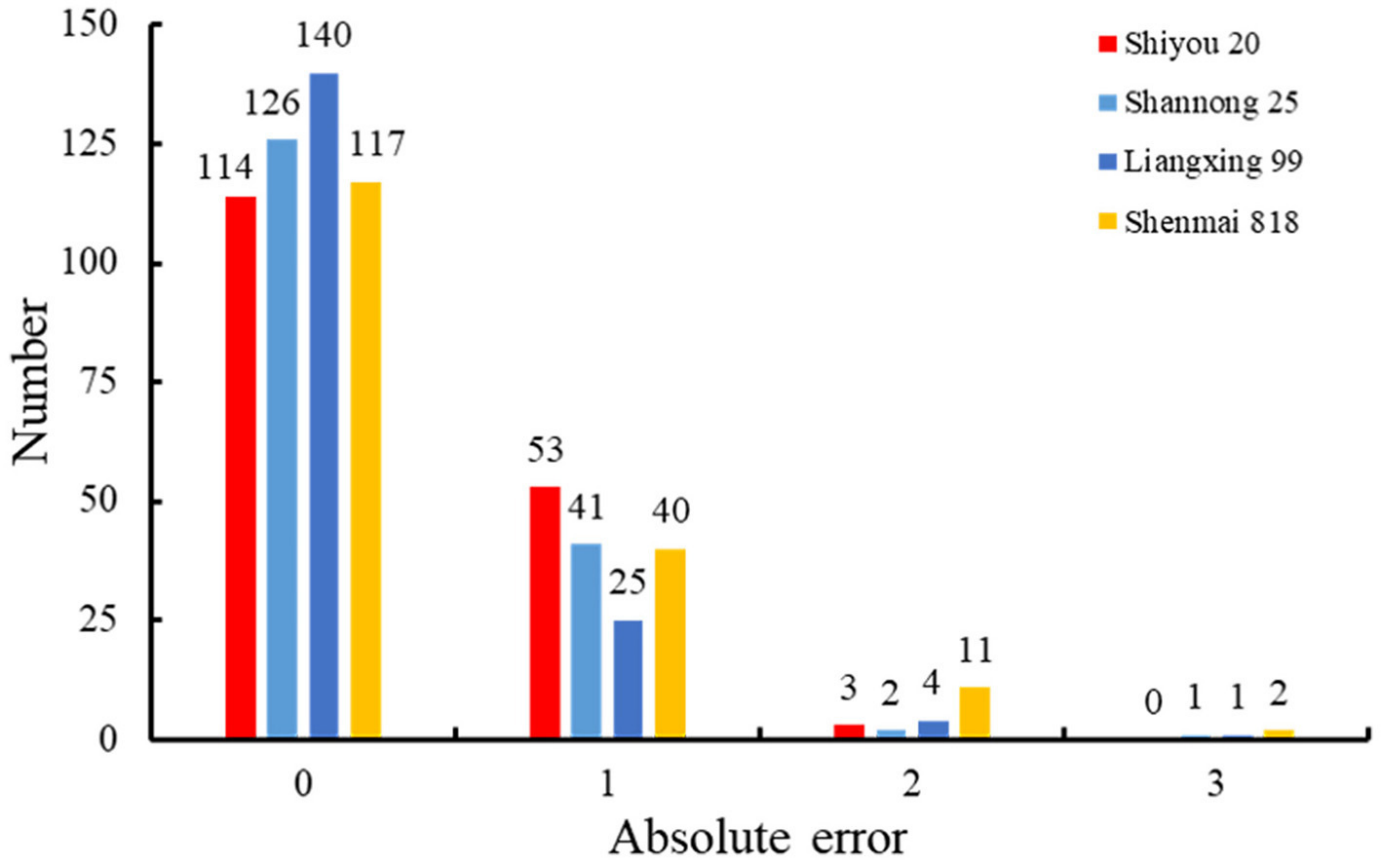
Distribution of the spikelet counting errors for all wheat lines.

**Figure 9:**
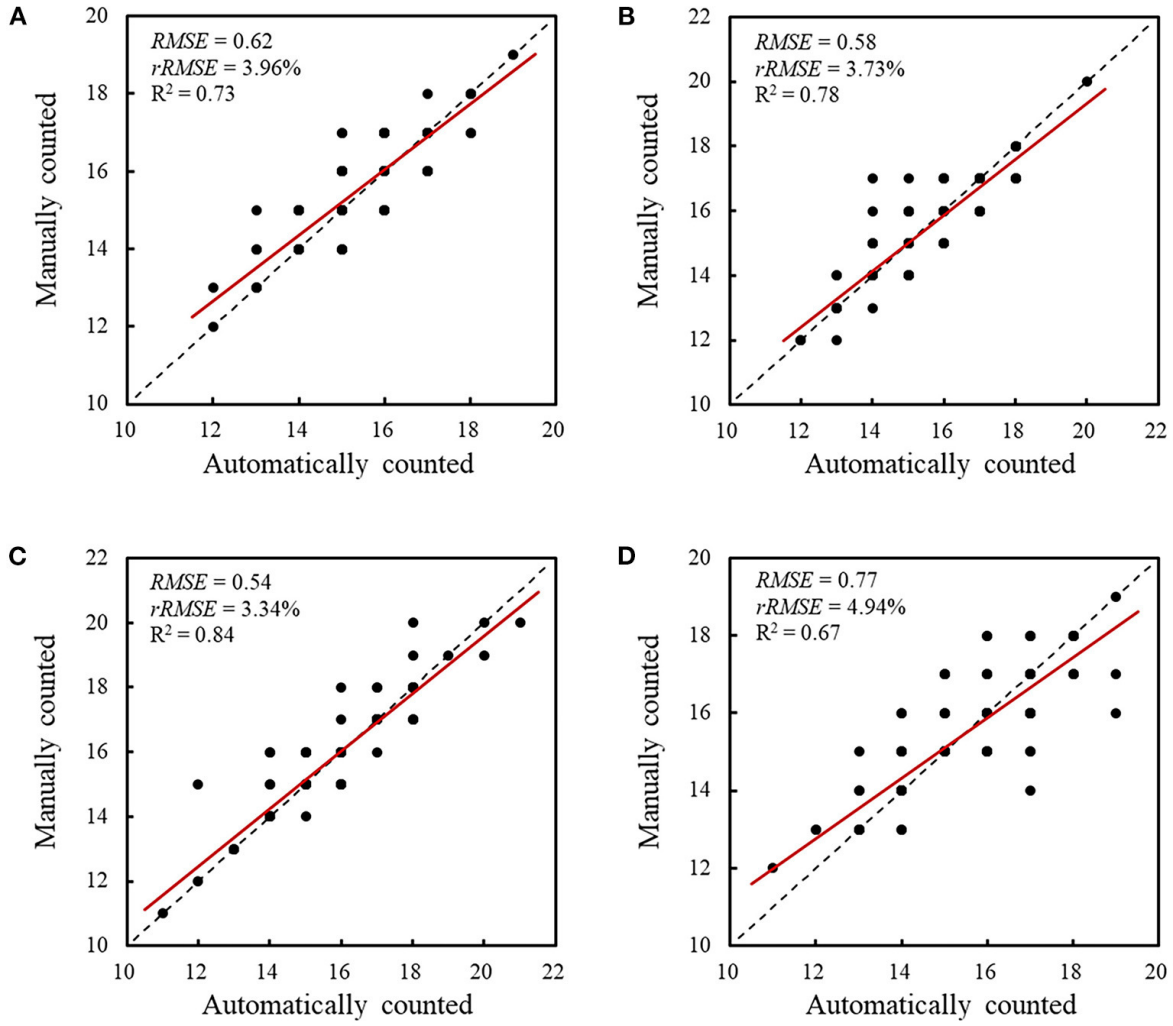
Comparison between the automatically and manually counted spikelet results. **(A)** Comparison and correlation for Shiyou 20, **(B)** comparison and correlation for Shannong 25, **(C)** comparison and correlation for Liangxing 99, and **(D)** comparison and correlation for Shenmai 818. The red lines indicate the least squares linear regression lines. The dashed lines are 1:1 lines.

To evaluate the performance of the retrained Faster RCNN model, the testing dataset consisting of 170 spike images for each wheat line that were not selected to generate the training and validation datasets was used to assess the detection and counting methods. The spikelets for each wheat line were detected (Figure 7) and counted using the retrained model. The results were summarized and compared with manual counting, as shown in Figures 8, 9. The average processing time of spike images required for spikelet counting was ~0.4 s. Most absolute errors for all wheat lines were not more than 1. The *RMSE, rRMSE*, and R^2^ between the automatically and manually counted results for Shiyou 20, Shannong 25, Liangxing 99, and Shenmai 818 varieties were 0.62, 0.58, 0.54, and 0.77; 3.96, 3.73, 3.34, and 4.94%; and 0.73, 0.78, 0.84, and 0.67, respectively. The detection results for all wheat lines are provided in the Supplementary Material.

## Discussion

### Image Annotation

A method based on a watershed algorithm was proposed to assist in the labeling of spikelet samples. Cb component of the spike in the color image was applied to obtain the gray image of the spike. The color component makes full use of the color characteristics of wheat glumes. On this basis, the watershed algorithm can roughly segment the spike into many regions that contain spikelets. To a certain extent, the application of MER reduces the segmentation errors produced by the watershed algorithm. In addition, the effect of wheat awns on spikelet labeling is small. The areas and ratio of length to width were set for MER, which can eliminate the regions that contain wheat awns. Although the proposed method successfully labeled many spikelets, it can achieve better performance on the wheat line whose color distribution is relatively uniform, such as Liangxing 99.

Manual corrections were conducted twice. For the first time, labeled spike images with higher annotation accuracy were manually selected. For the second time, more work was done to supplement unlabeled spikelets, modify some bounding boxes that were with small errors, and remove the labels for the sterile spikelets at the bottom of wheat spikes. Manual corrections assist us to improve the accuracy of annotation.

In the ACID dataset, Pound et al. (2017) annotated each spikelet by placing a dot in its center. In the present study, the spikelets were labeled using bounding boxes. The bounding boxes cannot only help in the detection and counting of the spikelets, but also facilitate the calculation of the length, width, and area of the spikelet in the present study.

Labeling the datasets by integrating machine learning algorithms and deep learning techniques can effectively reduce the labor cost and obtain high-quality datasets. The annotated datasets can be used for DCNN model training.

### Spikelet Detection and Counting

In this study, a Faster RCNN model was applied to detect and count the spikelets of the wheat spike. In the image detection field, image resolution has an impact on the detection performance of the model. During the training process, high image resolution requires expensive processes and higher hardware specifications. To detect the spikes in color images with high resolution, Madec et al. (2019) split the spike images into multiple sub-images and kept a 50% overlap between the sub-images to develop datasets for model training. The sub-images were processed, and spikes were detected to generate bounding boxes for spikes. The overlapped ratios of bounding boxes for neighbored sub-images were judged to remove some repeated detections. An object can be easily detected more than once using this strategy, and the detection results are highly dependent on the sub-images and overlapped ratio setting. Therefore, we prefer to reduce the image resolution to get satisfactory images. Although the ratio of length to width for the image is not 1:1 and is nearly 4:1, which is limited by the spike shape, the performance of our model for spikelet detection and counting shows that this strategy is appropriate for processing the color image that contains a single spike.

Pound et al. (2017) conducted similar work to develop models for spikelet counting using manually labeled samples. Their spikelet counting errors ranged from 0.06 to 3.81%. In our study, the spikelet counting and absolute errors were found to be 0.23 and 2.00%, respectively. There were no significant differences between our studies. Therefore, the proposed labeling method and developed model are practical for spikelet counting.

The spikelet detection and counting accuracy of our system is negatively affected by some factors. First and foremost, the testing results demonstrate that the sterile spikelets at the bottom of wheat spikes were mistakenly taken into account. Although the spikelet datasets were manually corrected and the sterile spikelet labels were removed, the sterile spikelets are not uniform for different wheat lines. First, some wheat lines hardly produce sterile spikelets. Second, even for the same wheat line, the sterile spikelets are missing in different spike samples. Third, there are great differences in the locations of the sterile spikelets. Many of them lay next to the spikelets that need to be counted, while others are independent. The performance of the final DCNN model for the sterile spikelet discrimination has been significantly improved compared to the model trained using the initial image annotation. Further work can be conducted to improve the sterile spikelet detection by combing its location and shapes (e.g., area, width, and the ratio of length to width). Another potential problem is that many spikelets are too big to be counted twice. Some grains inside the spikelets are plump, which makes the lateral florets prominent, due to which the lateral florets were incorrectly identified as spikelets. Finally, the effects of wheat awns on spikelet detection should be considered. Some spikelets were covered by awns in some spikes, particularly the upper spikelets, which were hard to be detected. In the following work, the covered spikelets may be estimated according to the symmetrical characteristics of spikelets.

## Conclusion

In this study, novel methods using color component selection and image processing techniques, combined with deep learning, were proposed to detect and count wheat spikelets in color images. Cb component and a watershed algorithm were implemented to process the color images of the spike and automatically label the spikelets. A DCNN model that was trained using the initially labeled datasets can further enlarge and optimize the datasets. The proposed labeling method can improve the efficiency and accuracy of dataset annotation. Then, a Faster RCNN model, retrained through the transfer learning technique and the obtained datasets, was capable of detecting and counting the spikelets in a spike image. For four wheat lines, *RMSE, rRMSE*, and R^2^ for the automatic and manual countings of spikelets were 0.62, 0.58, 0.54, and 0.77; 3.96, 3.73, 3.34, and 4.94%; and 0.73, 0.78, 0.84, and 0.67, respectively. These results demonstrated that the proposed methods can effectively detect and count spikelets, which will help breeders collect sufficient data to analyze the developmental characteristics of wheat spikes.

In future work, color images of spikes of other wheat lines will be collected to further test the applicability of the proposed methods. The detected spikelets contained several sterile spikelets at times. Hence, a model that can recognize sterile spikelets needs to be developed. The results of this study can also be combined with other wheat spike properties (e.g., GNS) to evaluate more wheat traits, such as spikelet fertilities. In addition, an app for smartphones can be developed to acquire the number of spikelet samples for field observation.

## Data Availability Statement

The original contributions presented in the study are included in the article/Supplementary Material, further inquiries can be directed to the corresponding author.

## Author Contributions

RQ: methodology, software, and writing of the original draft. YH: supervision and fund acquisition. MZ: writing, reviewing, and editing. All authors contributed to the article and approved the submitted version.

## Funding

This work was supported by the Key Projects of International Scientific and Technological Innovation Cooperation among Governments under National Key R&D Plan (2019YFE0103800).

## Conflict of Interest

The authors declare that the research was conducted in the absence of any commercial or financial relationships that could be construed as a potential conflict of interest.

## Publisher's Note

All claims expressed in this article are solely those of the authors and do not necessarily represent those of their affiliated organizations, or those of the publisher, the editors and the reviewers. Any product that may be evaluated in this article, or claim that may be made by its manufacturer, is not guaranteed or endorsed by the publisher.

## References

[B1] Alkhudaydi T. Zhou J. De La lglesia B. (2019). SpikeletFCN: counting spikelets from infield wheat crop images using fully convolutional networks, in International Conference Artificial Intelligence and Soft Computing (Zakopane), 3–13.

[B2] Ayalew T. W. Ubbens J. R. Stavness I. (2020). Unsupervised domain adaptation for plant organ counting, in Proceedings of European Conference on Computer Vision (ECCV) (Glasgow), 330–346.

[B3] Chen F. Pound M. P. French A. P. (2021). Learning to localise and count with incomplete dot-annotations, in Proceedings of the IEEE International Conference on Computer Vision (Montreal, QC), 1612–1620.

[B4] Duan L. Yang W. Bi K. Chen S. Luo Q. Liu Q. (2011). Fast discrimination and counting of filled/unfilled rice spikelets based on bi-modal imaging. Comput. Electron. Agric. 75, 196–203. 10.1016/j.compag.2010.11.004

[B5] García G. A. Serrago R. A. González F. G. Slafer G. A. Reynolds M. P. Miralles D. J. (2014). Wheat grain number: identification of favourable physiological traits in an elite doubled-haploid population. F. Crop. Res. 168, 126–134. 10.1016/j.fcr.2014.07.018

[B6] Genaev M. A. Komyshev E. G. Smirnov N. V. Kruchinina Y. V. Goncharov N. P. Afonnikov D. A. (2019). Morphometry of the wheat spike by analyzing 2D images. Agronomy 9, 390. 10.3390/agronomy9070390

[B7] Giuffrida M. V. Dobrescu A. Doerner P. Tsaftaris S. A. (2019). Leaf counting without annotations using adversarial unsupervised domain adaptation, in IEEE/CVF Conference on Computer Vision and Pattern Recognition Workshops, 2590–2599.

[B8] Guo Z. Zhao Y. Röder M. S. Reif J. C. Ganal M. W. Chen D.. (2018). Manipulation and prediction of spike morphology traits for the improvement of grain yield in wheat. Sci. Rep. 8, 14435. 10.1038/s41598-018-31977-3 30258057 PMC6158183

[B9] Hu G. Qian L. Liang D. Wan M. (2019). Self-adversarial training and attention for multi-task wheat phenotyping. Appll. Eng. Agric. 35, 1009–1014. 10.13031/aea.13406

[B10] Huang J. Rathod V. Sun C. Zhu M. Korattikara A. Fathi A.. (2017). Speed/accuracy trade-offs for modern convolutional object detectors, in Proceedings 30th IEEE Conference Computer Vision and Pattern Recognition, 3296–3305.

[B11] Hughes N. Askew K. Scotson C. P. Williams K. Sauze C. Corke F.. (2017). Non-destructive, high-content analysis of wheat grain traits using X-ray micro computed tomography. Plant Methods 13, 76. 10.1186/s13007-017-0229-8 29118820 PMC5664813

[B12] Kaya E. Saritas I. (2019). Towards a real-time sorting system: identification of vitreous durum wheat kernels using ANN based on their morphological, colour, wavelet and gaborlet features. Comput. Electron. Agric. 166, 105016. 10.1016/j.compag.2019.105016

[B13] Khoroshevsky F. Khoroshevsky S. Bar-Hillel A. (2021). Parts-per-object count in agricultural images: solving phenotyping problems *via* a single deep neural network. Remote Sens. 13, 2496. 10.3390/rs13132496

[B14] Li Q. Cai J. Berger B. Okamoto M. Miklavcic S. J. (2017). Detecting spikes of wheat plants using neural networks with Laws texture energy. Plant Methods 13, 83. 10.1186/s13007-017-0231-1 29046709 PMC5640952

[B15] Liu T. Chen W. Wang Y. Wu W. Sun C. Ding J.. (2017). Rice and wheat grain counting method and software development based on Android system. Comput. Electron. Agric. 141, 302–309. 10.1016/j.compag.2017.08.011

[B16] Madec S. Jin X. Lu H. De Solan B. Liu S. Duyme F.. (2019). Ear density estimation from high resolution RGB imagery using deep learning technique. Agric. For. Meteorol. 264, 225–234. 10.1016/j.agrformet.2018.10.013

[B17] Misra T. Arora A. Marwaha S. Chinnusamy V. Rao A. R. Jain R.. (2020). SpikeSegNet-a deep learning approach utilizing encoder-decoder network with hourglass for spike segmentation and counting in wheat plant from visual imaging. Plant Methods 16, 40. 10.1186/s13007-020-00582-9 32206080 PMC7079463

[B18] Pound M. P. Atkinson J. A. Wells D. M. Pridmore T. P. French A. P. (2017). Deep learning for multi-task plant phenotyping, in Proceedings of the IEEE International Conference on Computer Vision Workshops, 2055–2063.

[B19] Qiu R. Wei S. Zhang M. Li H. Sun H. Liu G.. (2018). Sensors for measuring plant phenotyping: a review. Int. J. Agric. Biol. Eng. 11, 1–17. 10.25165/j.ijabe.20181102.2696

[B20] Qiu R. Yang C. Moghimi A. Zhang M. Steffenson B. J. Hirsch C. D. (2019). Detection of Fusarium head blight in wheat using a deep neural network and color imaging. Remote Sens. 11, 2658. 10.3390/rs11222658

[B21] Ren S. He K. Girshick R. Sun J. (2017). Faster R-CNN: towards real-time object detection with region proposal networks. IEEE Trans. Pattern Anal. Mach. Intell. 39, 1137–1149. 10.1109/TPAMI.2016.2577031 27295650

[B22] Sadeghi-Tehran P. Virlet N. Ampe E. M. Reyns P. Hawkesford M. J. (2019). DeepCount: in-field automatic quantification of wheat spikes using simple linear iterative clustering and deep convolutional neural networks. Front. Plant Sci. 10, 1176. 10.3389/fpls.2019.01176 31616456 PMC6775245

[B23] Vahamidis P. Karamanos A. J. Economou G. (2019). Grain number determination in durum wheat as affected by drought stress: an analysis at spike and spikelet level. Ann. Appl. Biol. 174, 190–208. 10.1111/aab.12487

[B24] Xiong B. Wang B. Xiong S. Lin C. Yuan X. (2019a). 3D morphological processing for wheat spike phenotypes using computed tomography images. Remote Sens. 11, 1110. 10.3390/rs11091110

[B25] Xiong H. Cao Z. Lu H. Madec S. Liu L. Shen C. (2019b). TasselNetv2: In-field counting of wheat spikes with context-augmented local regression networks. Plant Methods 15, 150. 10.1186/s13007-019-0537-2 31857821 PMC6905110

[B26] Xu X. Li H. Yin F. Xi L. Qiao H. Ma Z.. (2020). Wheat ear counting using K-means clustering segmentation and convolutional neural network. Plant Methods 16, 106. 10.1186/s13007-020-00648-8 32782453 PMC7412807

[B27] Yu L. Shi J. Huang C. Duan L. Wu D. Fu D.. (2021). An integrated rice panicle phenotyping method based on X-ray and RGB scanning and deep learning. Crop J. 9, 42–56. 10.1016/j.cj.2020.06.009

[B28] Zhou C. Liang D. Yang X. Yang H. Yue J. Yang G. (2018). Wheat ears counting in field conditions based on multi-feature optimization and TWSVM. Front. Plant Sci. 9, 1024. 10.3389/fpls.2018.01024 30057587 PMC6053621

[B29] Zhou H. Riche A. B. Hawkesford M. J. Whalley W. R. Atkinson B. S. Sturrock C. J.. (2021). Determination of wheat spike and spikelet architecture and grain traits using X-ray Computed Tomography imaging. Plant Methods 17, 26. 10.1186/s13007-021-00726-5 33750418 PMC7945051

