# Supplementary figures and images for: Automatic Detection and Counting of Wheat Spikelet Using Semi-Automatic Labeling and Deep Learning (part 2 of 8)
Source: Front Plant Sci. 2022 May 30;13:872555. doi: 10.3389/fpls.2022.872555 (PMC9189412; doi:10.3389/fpls.2022.872555)

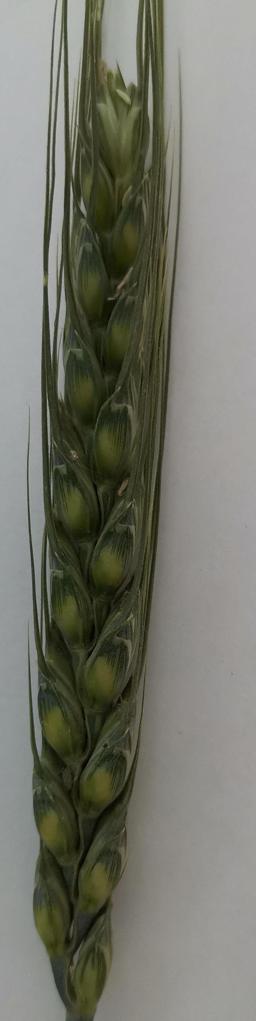

Supplement: Supplementary file 1 [file Data_Sheet_1.ZIP › 2. Datasets/1. training dataset for model training/Shannong 25/2080.jpg]

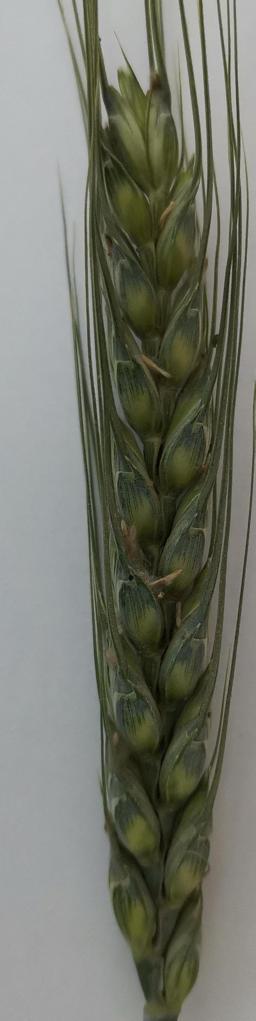

Supplement: Supplementary file 1 [file Data_Sheet_1.ZIP › 2. Datasets/1. training dataset for model training/Shannong 25/2146.jpg]

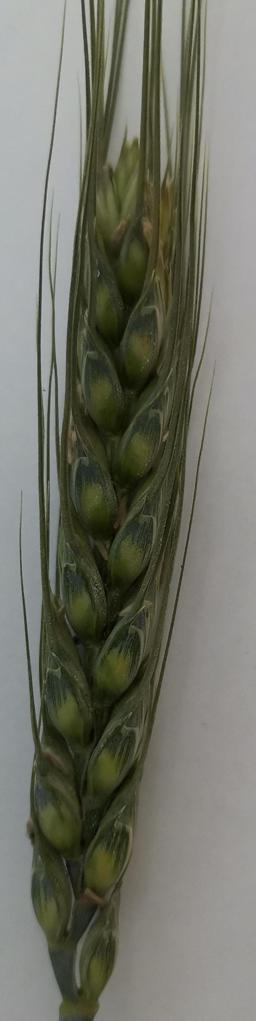

Supplement: Supplementary file 1 [file Data_Sheet_1.ZIP › 2. Datasets/1. training dataset for model training/Shannong 25/2147.jpg]

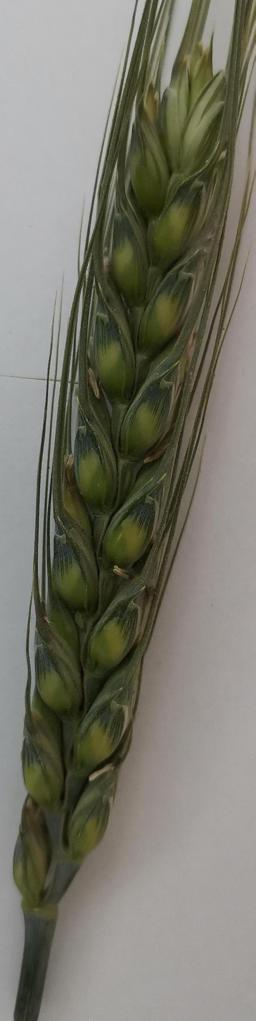

Supplement: Supplementary file 1 [file Data_Sheet_1.ZIP › 2. Datasets/1. training dataset for model training/Shannong 25/2148.jpg]

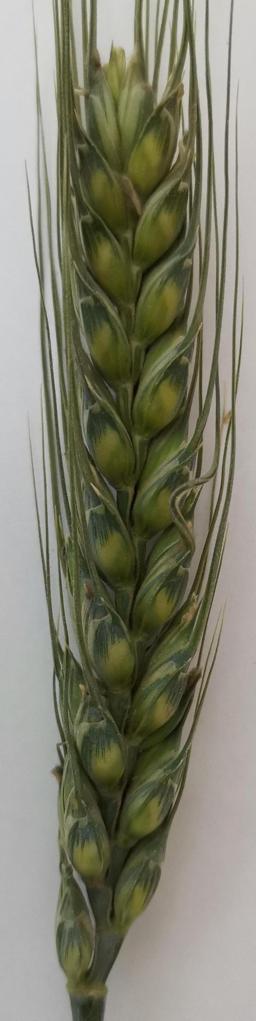

Supplement: Supplementary file 1 [file Data_Sheet_1.ZIP › 2. Datasets/1. training dataset for model training/Shannong 25/2150.jpg]

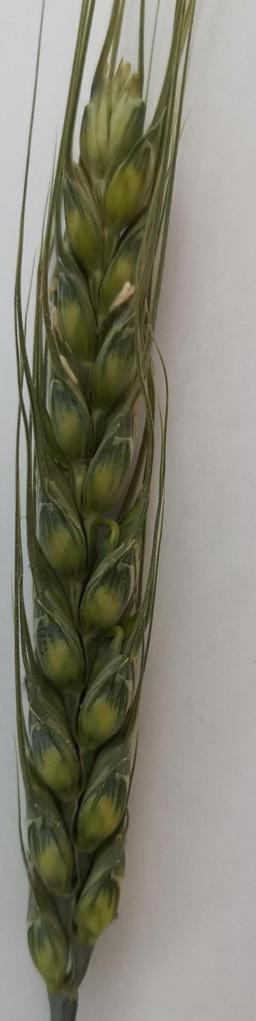

Supplement: Supplementary file 1 [file Data_Sheet_1.ZIP › 2. Datasets/1. training dataset for model training/Shannong 25/2152.jpg]

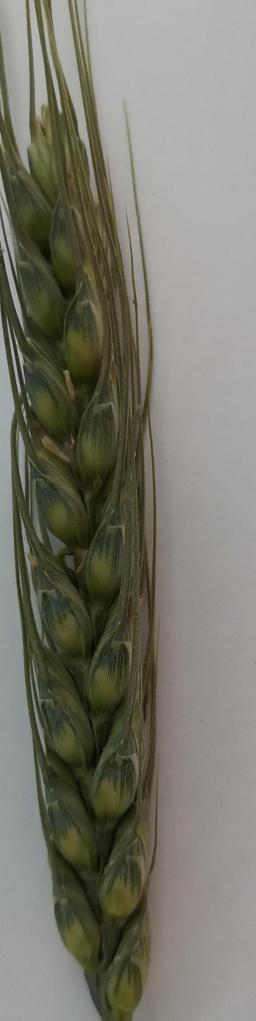

Supplement: Supplementary file 1 [file Data_Sheet_1.ZIP › 2. Datasets/1. training dataset for model training/Shannong 25/2153.jpg]

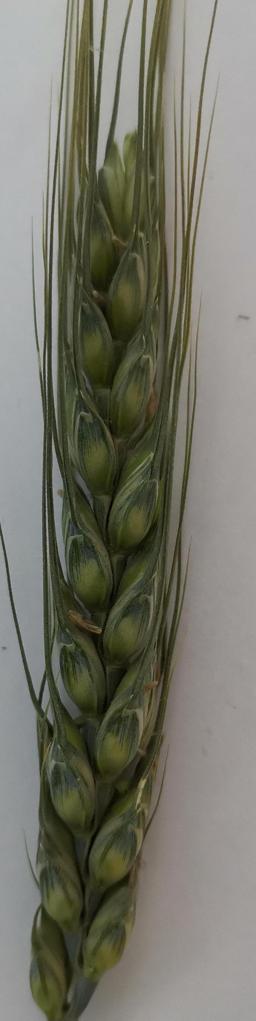

Supplement: Supplementary file 1 [file Data_Sheet_1.ZIP › 2. Datasets/1. training dataset for model training/Shannong 25/2162.jpg]

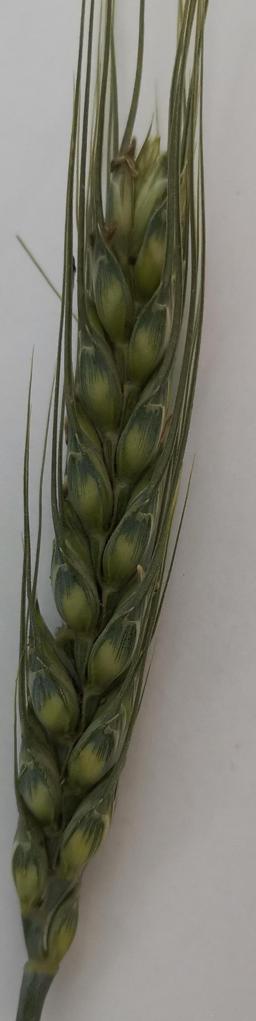

Supplement: Supplementary file 1 [file Data_Sheet_1.ZIP › 2. Datasets/1. training dataset for model training/Shannong 25/2163.jpg]

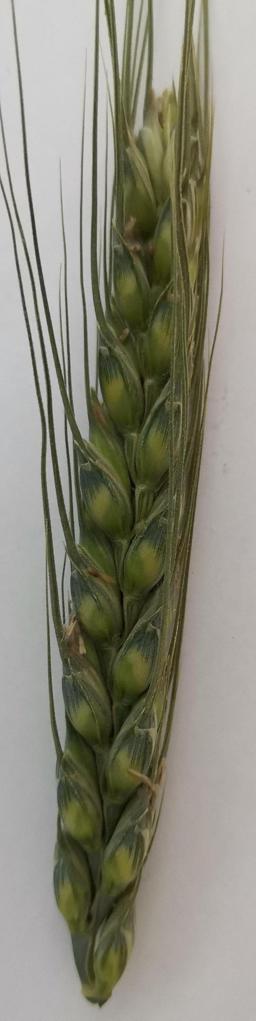

Supplement: Supplementary file 1 [file Data_Sheet_1.ZIP › 2. Datasets/1. training dataset for model training/Shannong 25/2169.jpg]

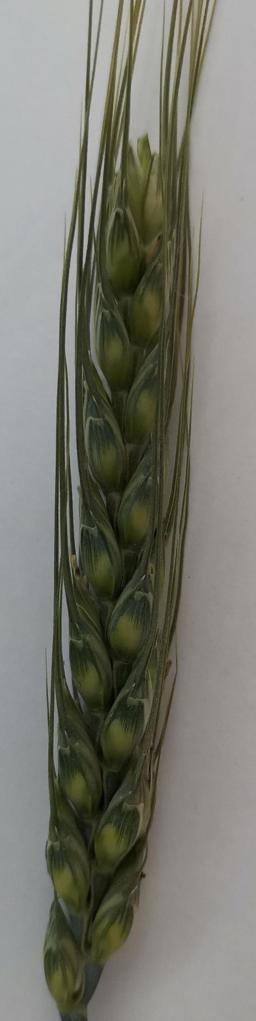

Supplement: Supplementary file 1 [file Data_Sheet_1.ZIP › 2. Datasets/1. training dataset for model training/Shannong 25/2178.jpg]

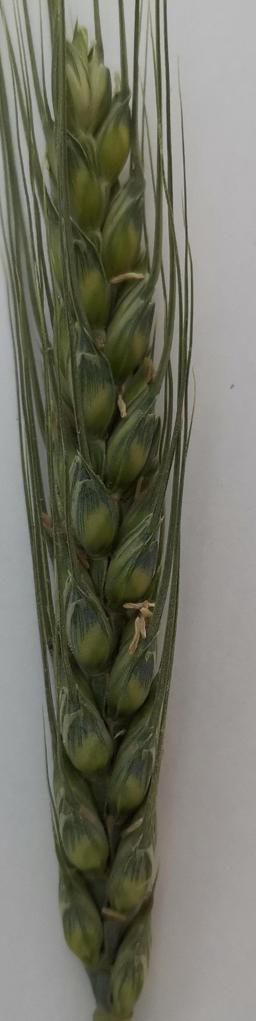

Supplement: Supplementary file 1 [file Data_Sheet_1.ZIP › 2. Datasets/1. training dataset for model training/Shannong 25/2179.jpg]

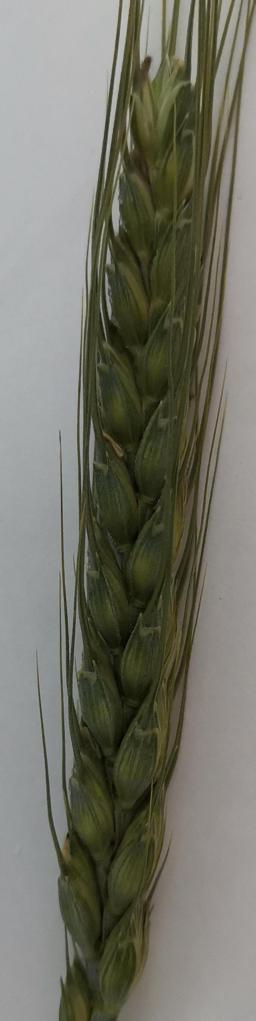

Supplement: Supplementary file 1 [file Data_Sheet_1.ZIP › 2. Datasets/1. training dataset for model training/Shannong 25/2181.jpg]

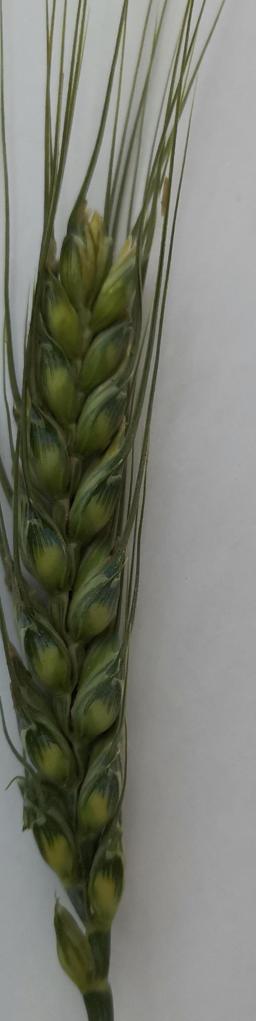

Supplement: Supplementary file 1 [file Data_Sheet_1.ZIP › 2. Datasets/1. training dataset for model training/Shannong 25/2187.jpg]

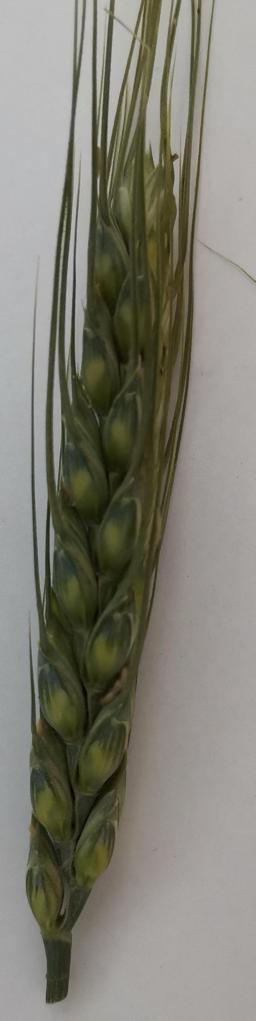

Supplement: Supplementary file 1 [file Data_Sheet_1.ZIP › 2. Datasets/1. training dataset for model training/Shannong 25/2189.jpg]

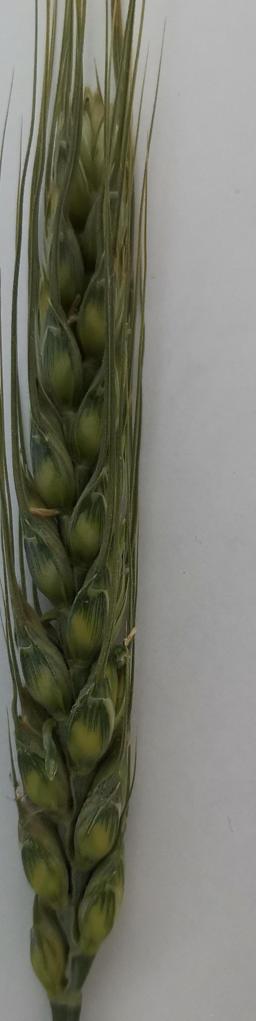

Supplement: Supplementary file 1 [file Data_Sheet_1.ZIP › 2. Datasets/1. training dataset for model training/Shannong 25/2191.jpg]

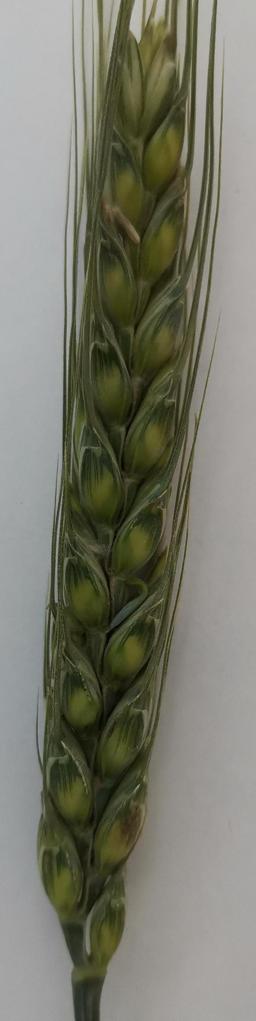

Supplement: Supplementary file 1 [file Data_Sheet_1.ZIP › 2. Datasets/1. training dataset for model training/Shannong 25/2192.jpg]

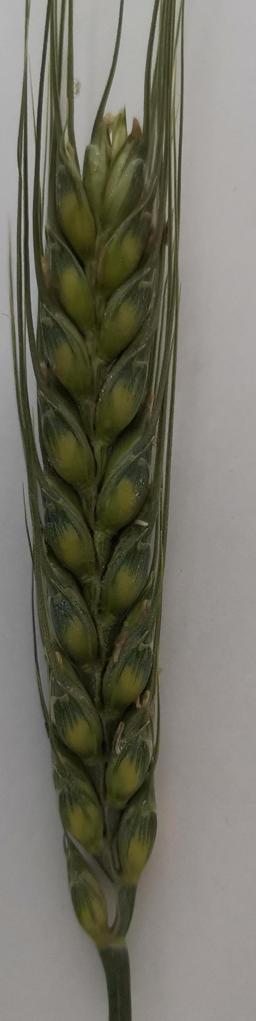

Supplement: Supplementary file 1 [file Data_Sheet_1.ZIP › 2. Datasets/1. training dataset for model training/Shannong 25/2195.jpg]

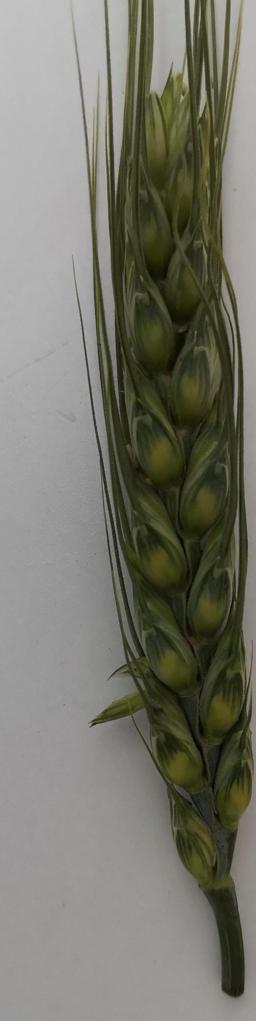

Supplement: Supplementary file 1 [file Data_Sheet_1.ZIP › 2. Datasets/1. training dataset for model training/Shannong 25/2197.jpg]

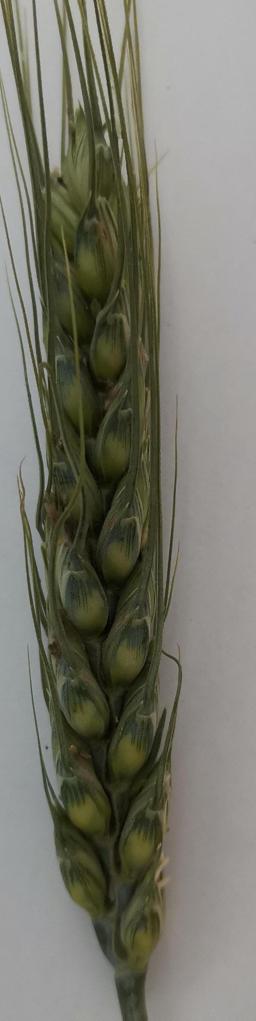

Supplement: Supplementary file 1 [file Data_Sheet_1.ZIP › 2. Datasets/1. training dataset for model training/Shannong 25/2201.jpg]

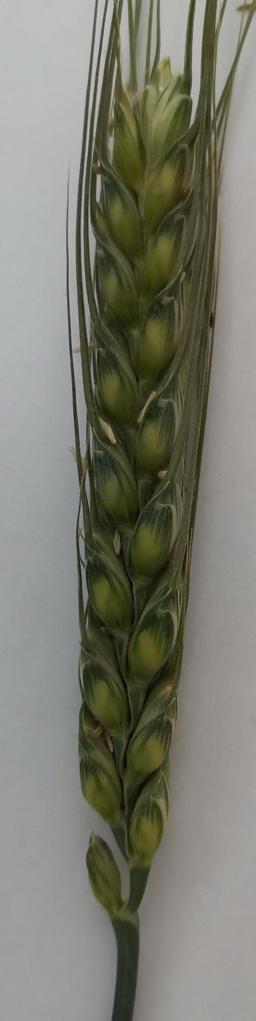

Supplement: Supplementary file 1 [file Data_Sheet_1.ZIP › 2. Datasets/1. training dataset for model training/Shannong 25/2211.jpg]

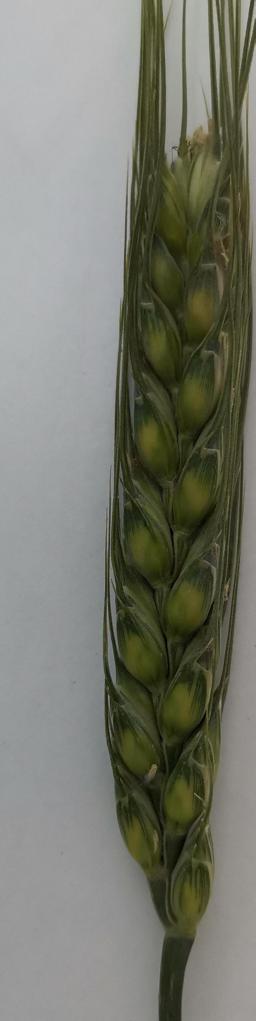

Supplement: Supplementary file 1 [file Data_Sheet_1.ZIP › 2. Datasets/1. training dataset for model training/Shannong 25/2239.jpg]

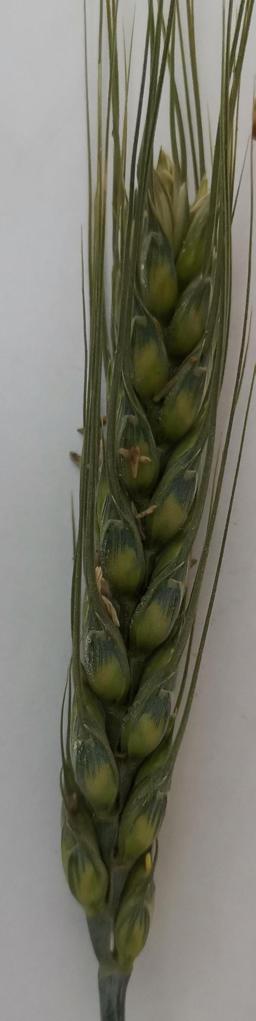

Supplement: Supplementary file 1 [file Data_Sheet_1.ZIP › 2. Datasets/1. training dataset for model training/Shannong 25/2240.jpg]

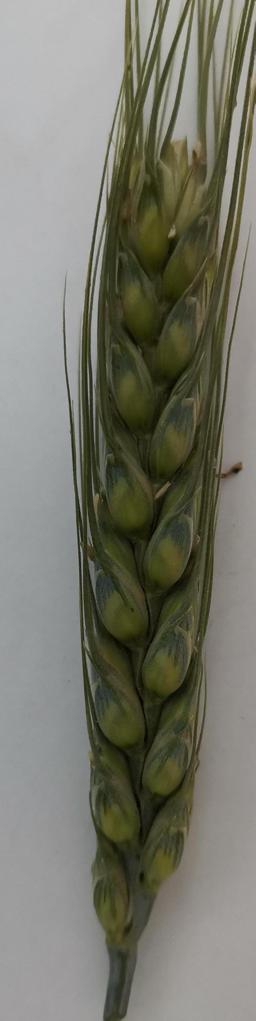

Supplement: Supplementary file 1 [file Data_Sheet_1.ZIP › 2. Datasets/1. training dataset for model training/Shannong 25/2241.jpg]

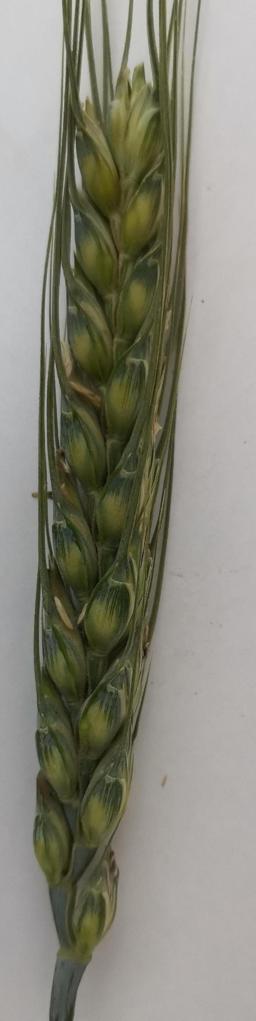

Supplement: Supplementary file 1 [file Data_Sheet_1.ZIP › 2. Datasets/1. training dataset for model training/Shannong 25/2243.jpg]

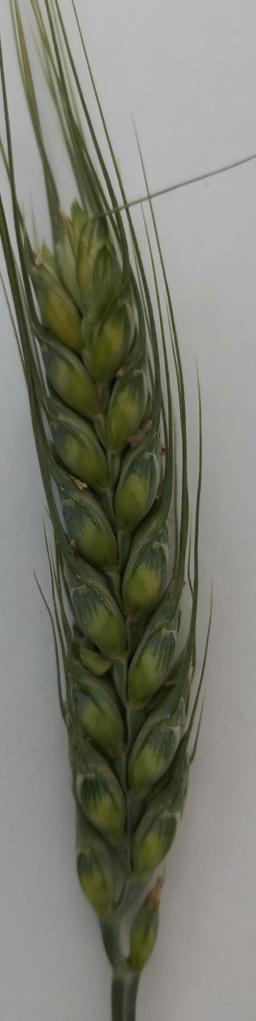

Supplement: Supplementary file 1 [file Data_Sheet_1.ZIP › 2. Datasets/1. training dataset for model training/Shannong 25/2258.jpg]

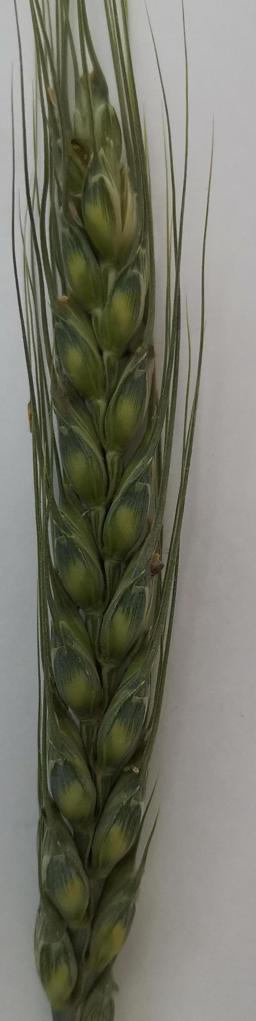

Supplement: Supplementary file 1 [file Data_Sheet_1.ZIP › 2. Datasets/1. training dataset for model training/Shannong 25/2259.jpg]

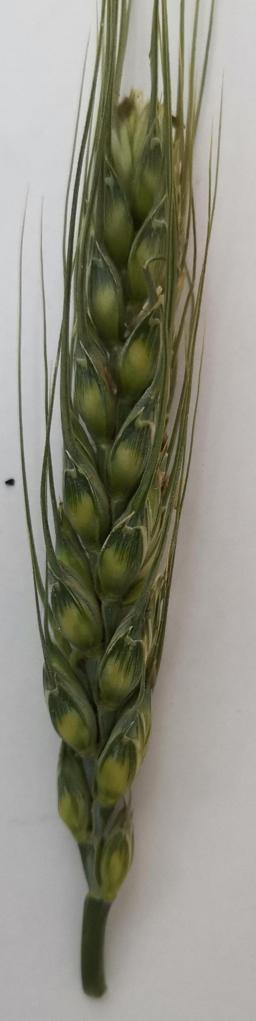

Supplement: Supplementary file 1 [file Data_Sheet_1.ZIP › 2. Datasets/1. training dataset for model training/Shannong 25/2261.jpg]

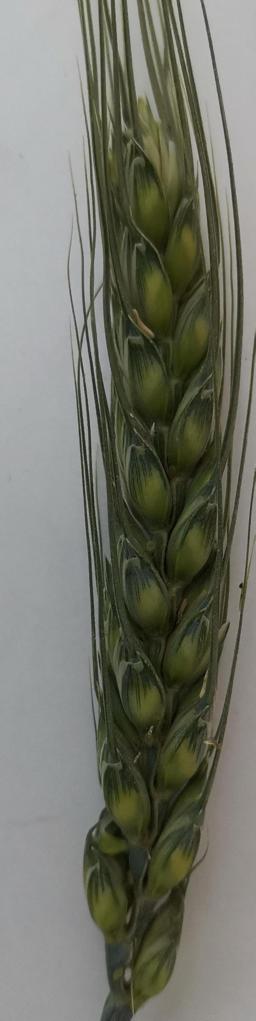

Supplement: Supplementary file 1 [file Data_Sheet_1.ZIP › 2. Datasets/1. training dataset for model training/Shannong 25/2263.jpg]

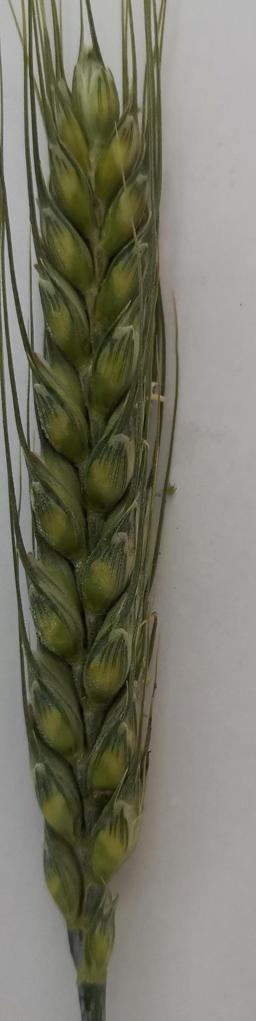

Supplement: Supplementary file 1 [file Data_Sheet_1.ZIP › 2. Datasets/1. training dataset for model training/Shannong 25/2266.jpg]

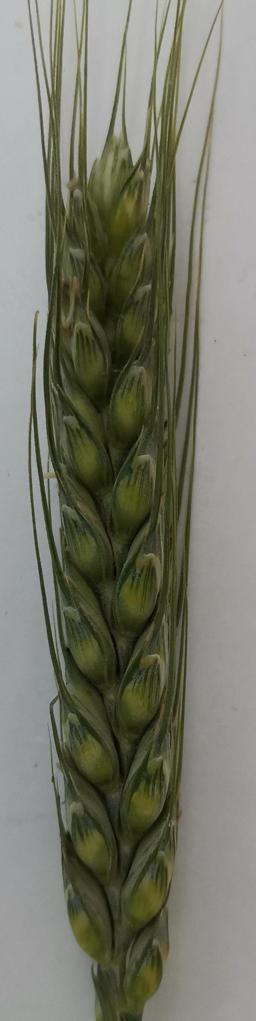

Supplement: Supplementary file 1 [file Data_Sheet_1.ZIP › 2. Datasets/1. training dataset for model training/Shannong 25/2267.jpg]

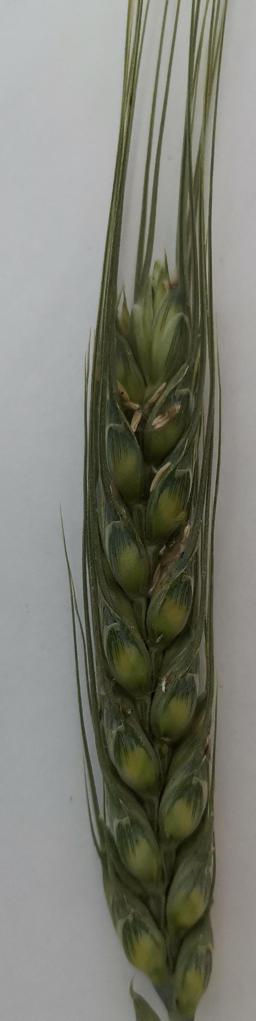

Supplement: Supplementary file 1 [file Data_Sheet_1.ZIP › 2. Datasets/1. training dataset for model training/Shannong 25/2270.jpg]

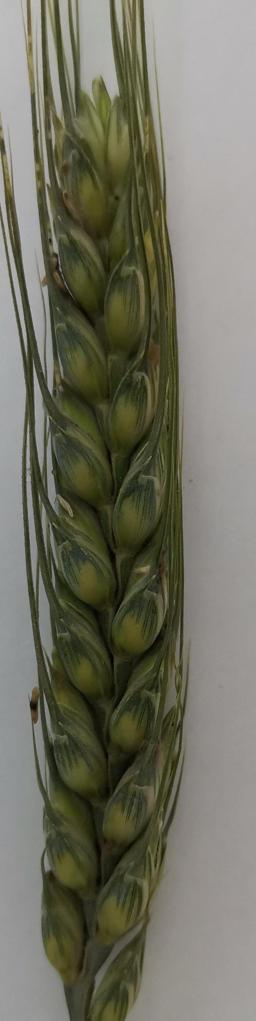

Supplement: Supplementary file 1 [file Data_Sheet_1.ZIP › 2. Datasets/1. training dataset for model training/Shannong 25/2273.jpg]

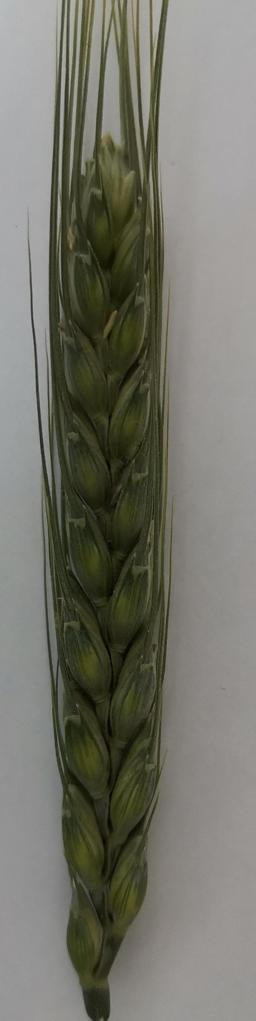

Supplement: Supplementary file 1 [file Data_Sheet_1.ZIP › 2. Datasets/1. training dataset for model training/Shannong 25/2275.jpg]

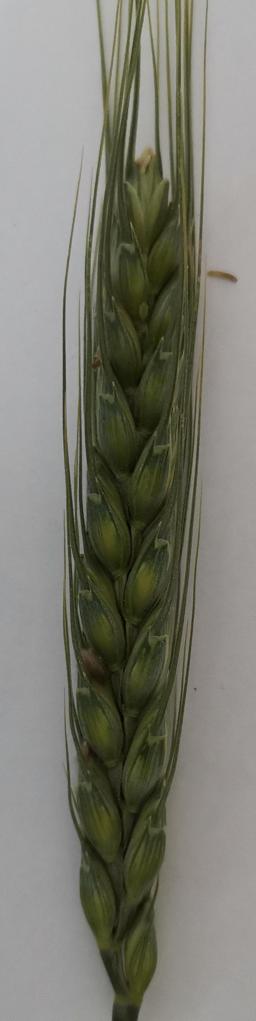

Supplement: Supplementary file 1 [file Data_Sheet_1.ZIP › 2. Datasets/1. training dataset for model training/Shannong 25/2276.jpg]

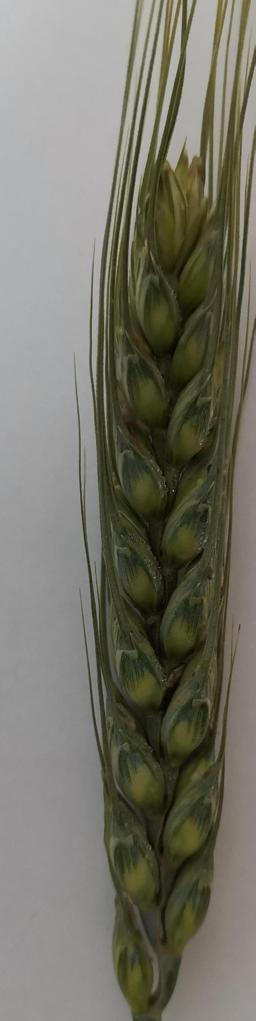

Supplement: Supplementary file 1 [file Data_Sheet_1.ZIP › 2. Datasets/1. training dataset for model training/Shannong 25/2280.jpg]

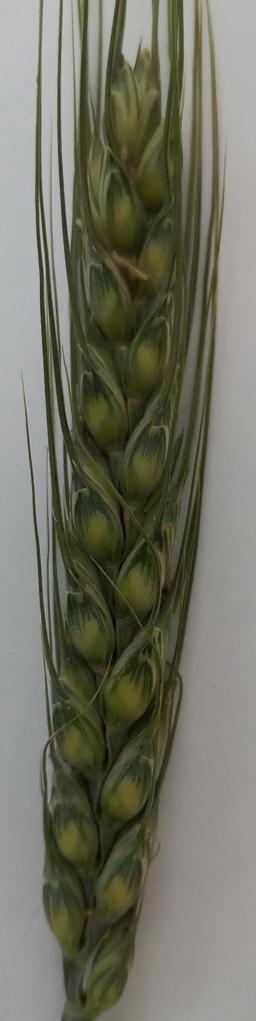

Supplement: Supplementary file 1 [file Data_Sheet_1.ZIP › 2. Datasets/1. training dataset for model training/Shannong 25/2282.jpg]

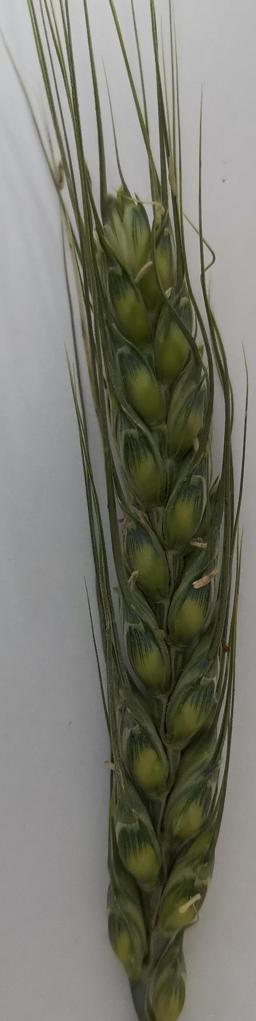

Supplement: Supplementary file 1 [file Data_Sheet_1.ZIP › 2. Datasets/1. training dataset for model training/Shannong 25/2286.jpg]

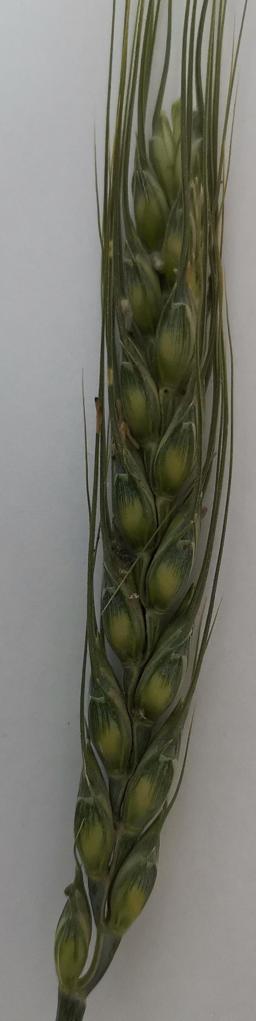

Supplement: Supplementary file 1 [file Data_Sheet_1.ZIP › 2. Datasets/1. training dataset for model training/Shannong 25/2287.jpg]

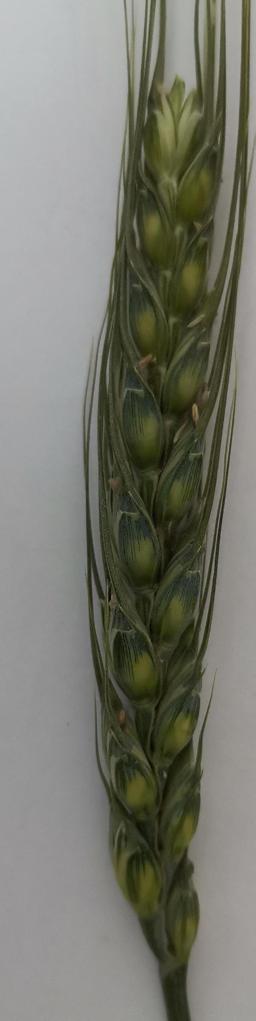

Supplement: Supplementary file 1 [file Data_Sheet_1.ZIP › 2. Datasets/1. training dataset for model training/Shannong 25/2288.jpg]

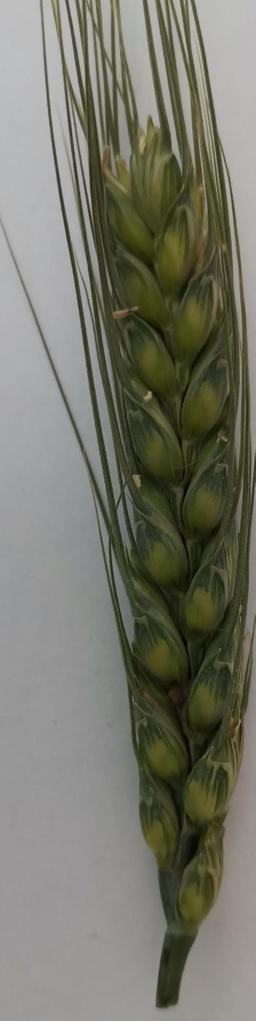

Supplement: Supplementary file 1 [file Data_Sheet_1.ZIP › 2. Datasets/1. training dataset for model training/Shannong 25/2289.jpg]

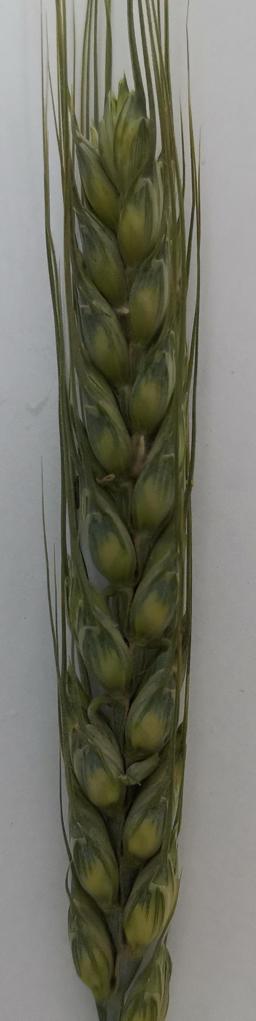

Supplement: Supplementary file 1 [file Data_Sheet_1.ZIP › 2. Datasets/1. training dataset for model training/Shannong 25/2291.jpg]

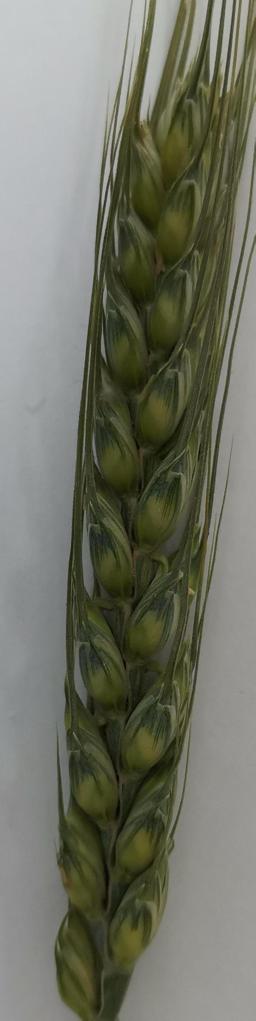

Supplement: Supplementary file 1 [file Data_Sheet_1.ZIP › 2. Datasets/1. training dataset for model training/Shannong 25/2292.jpg]

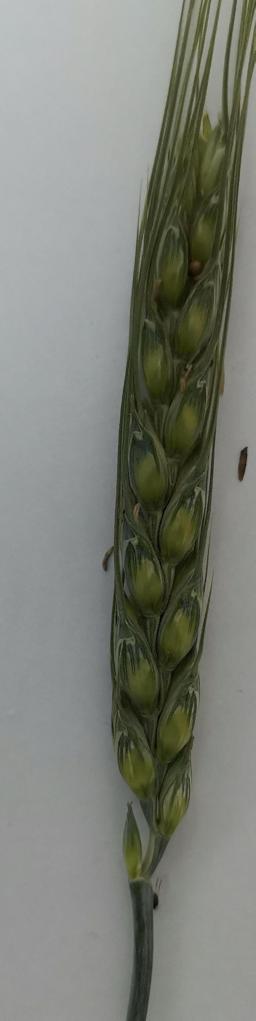

Supplement: Supplementary file 1 [file Data_Sheet_1.ZIP › 2. Datasets/1. training dataset for model training/Shannong 25/2294.jpg]

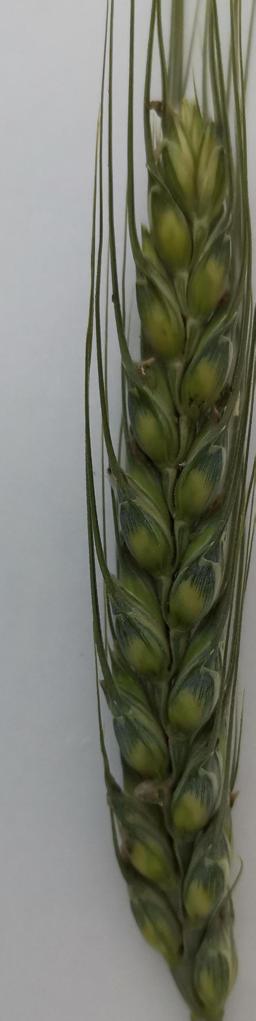

Supplement: Supplementary file 1 [file Data_Sheet_1.ZIP › 2. Datasets/1. training dataset for model training/Shannong 25/2296.jpg]

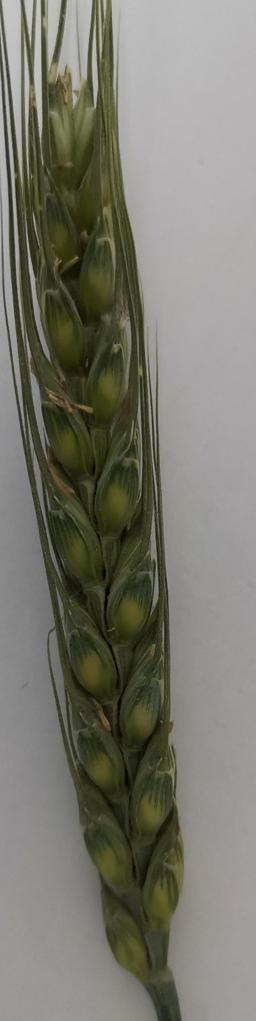

Supplement: Supplementary file 1 [file Data_Sheet_1.ZIP › 2. Datasets/1. training dataset for model training/Shannong 25/2299.jpg]

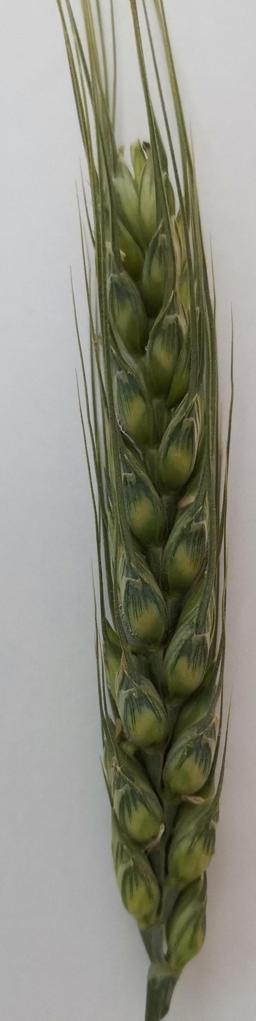

Supplement: Supplementary file 1 [file Data_Sheet_1.ZIP › 2. Datasets/1. training dataset for model training/Shannong 25/2313.jpg]

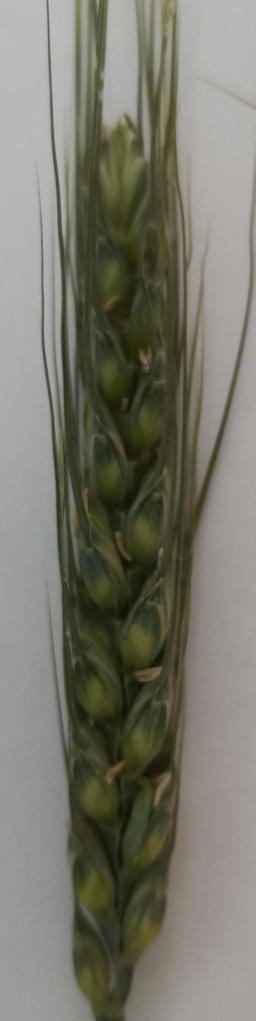

Supplement: Supplementary file 1 [file Data_Sheet_1.ZIP › 2. Datasets/1. training dataset for model training/Shannong 25/2318.jpg]

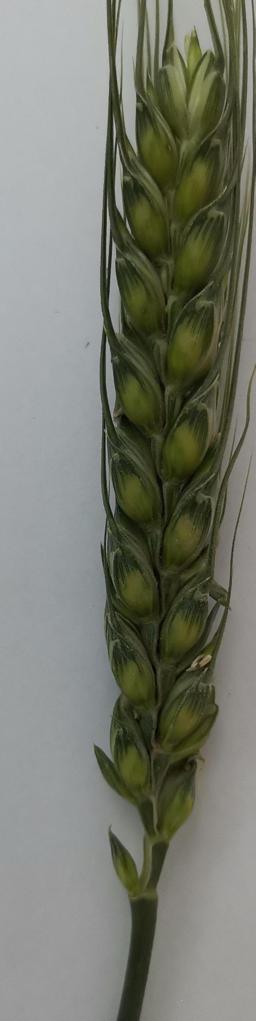

Supplement: Supplementary file 1 [file Data_Sheet_1.ZIP › 2. Datasets/1. training dataset for model training/Shannong 25/2319.jpg]

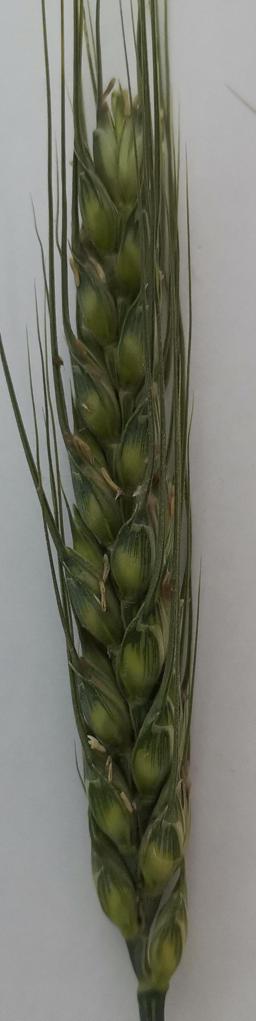

Supplement: Supplementary file 1 [file Data_Sheet_1.ZIP › 2. Datasets/1. training dataset for model training/Shannong 25/2320.jpg]

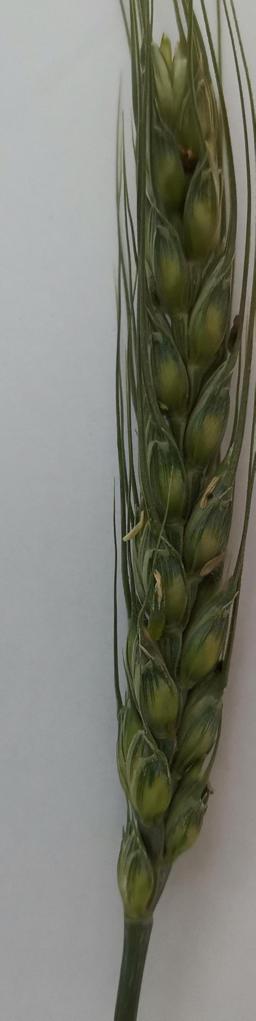

Supplement: Supplementary file 1 [file Data_Sheet_1.ZIP › 2. Datasets/1. training dataset for model training/Shannong 25/2321.jpg]

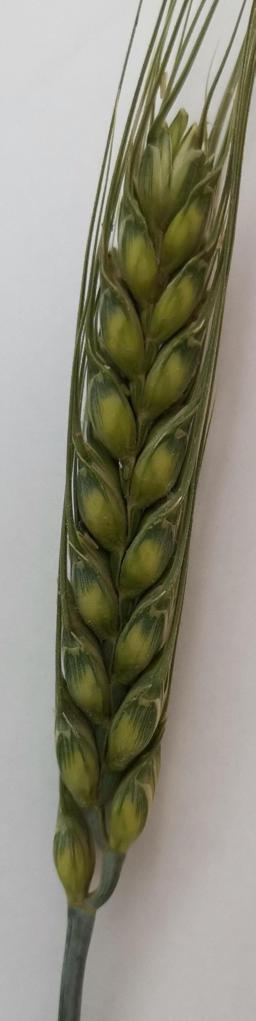

Supplement: Supplementary file 1 [file Data_Sheet_1.ZIP › 2. Datasets/1. training dataset for model training/Shannong 25/2324.jpg]

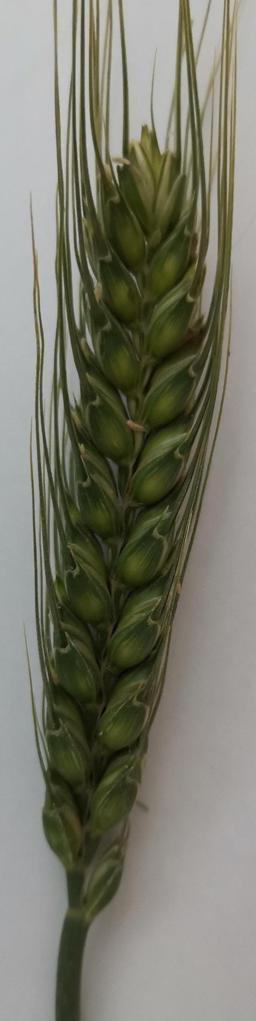

Supplement: Supplementary file 1 [file Data_Sheet_1.ZIP › 2. Datasets/1. training dataset for model training/Shannong 25/2328.jpg]

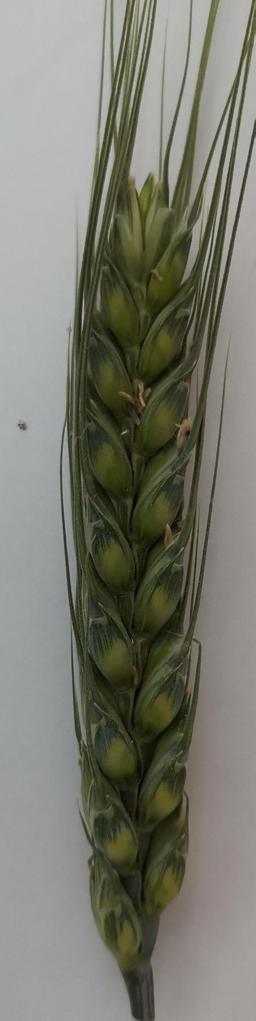

Supplement: Supplementary file 1 [file Data_Sheet_1.ZIP › 2. Datasets/1. training dataset for model training/Shannong 25/2329.jpg]

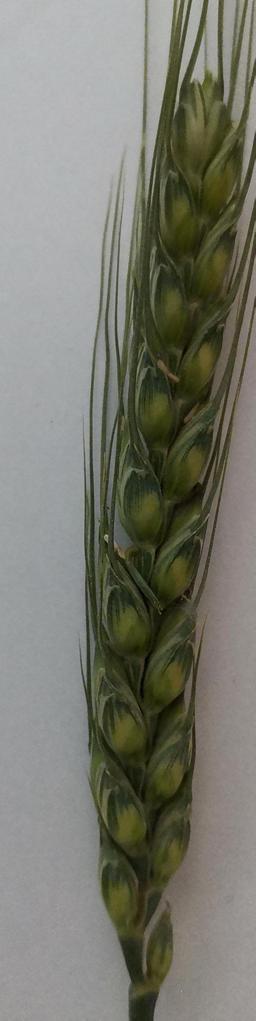

Supplement: Supplementary file 1 [file Data_Sheet_1.ZIP › 2. Datasets/1. training dataset for model training/Shannong 25/2334.jpg]

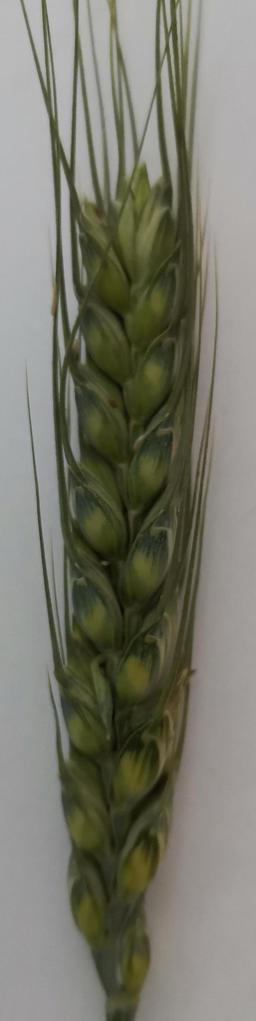

Supplement: Supplementary file 1 [file Data_Sheet_1.ZIP › 2. Datasets/1. training dataset for model training/Shannong 25/2337.jpg]

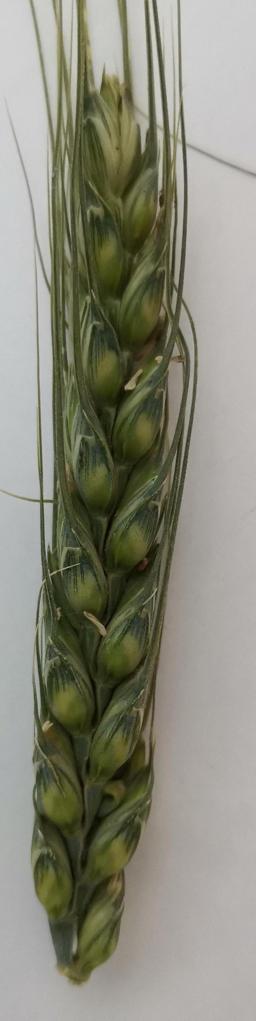

Supplement: Supplementary file 1 [file Data_Sheet_1.ZIP › 2. Datasets/1. training dataset for model training/Shannong 25/2339.jpg]

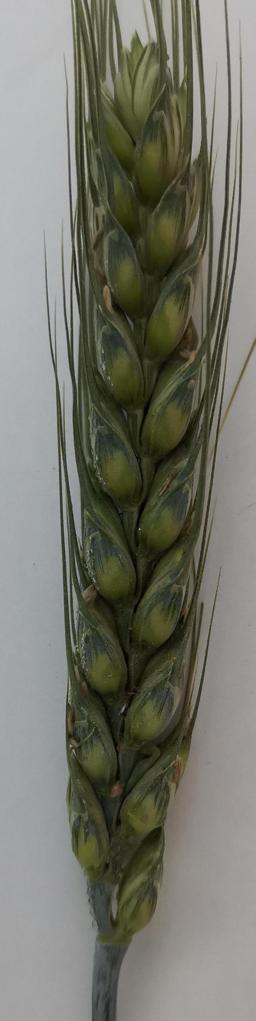

Supplement: Supplementary file 1 [file Data_Sheet_1.ZIP › 2. Datasets/1. training dataset for model training/Shannong 25/2343.jpg]

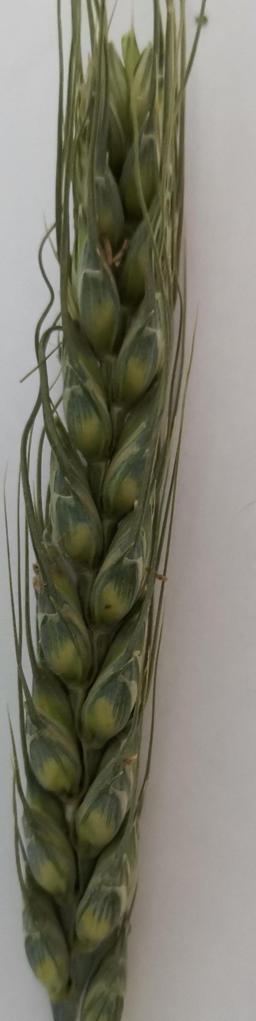

Supplement: Supplementary file 1 [file Data_Sheet_1.ZIP › 2. Datasets/1. training dataset for model training/Shannong 25/2360.jpg]

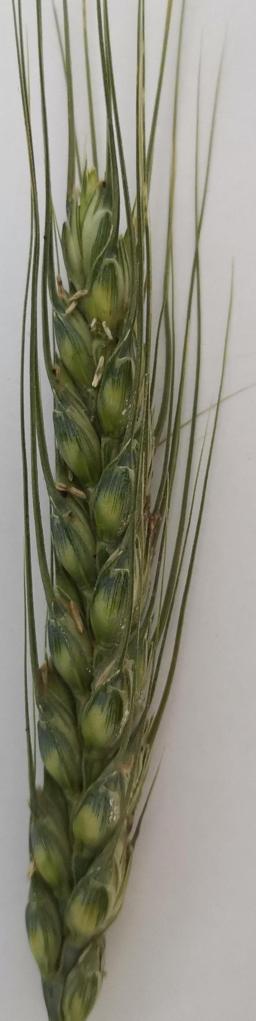

Supplement: Supplementary file 1 [file Data_Sheet_1.ZIP › 2. Datasets/1. training dataset for model training/Shannong 25/2368.jpg]

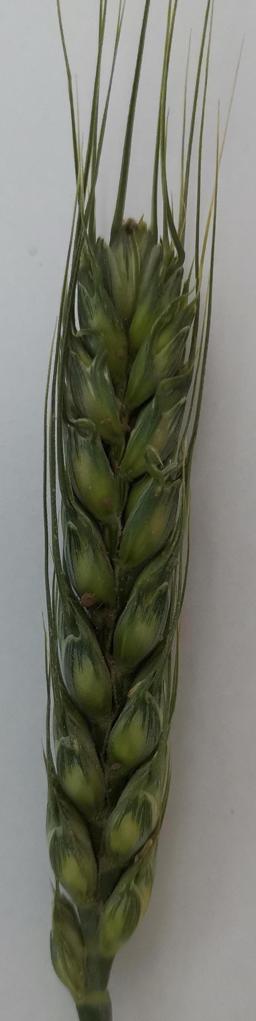

Supplement: Supplementary file 1 [file Data_Sheet_1.ZIP › 2. Datasets/1. training dataset for model training/Shenmai 818/5001.jpg]

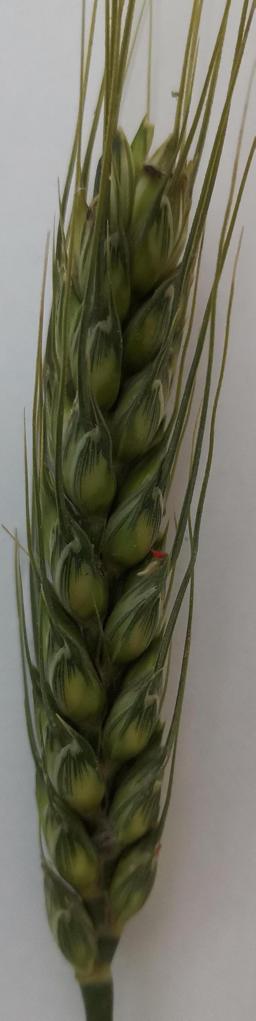

Supplement: Supplementary file 1 [file Data_Sheet_1.ZIP › 2. Datasets/1. training dataset for model training/Shenmai 818/5009.jpg]

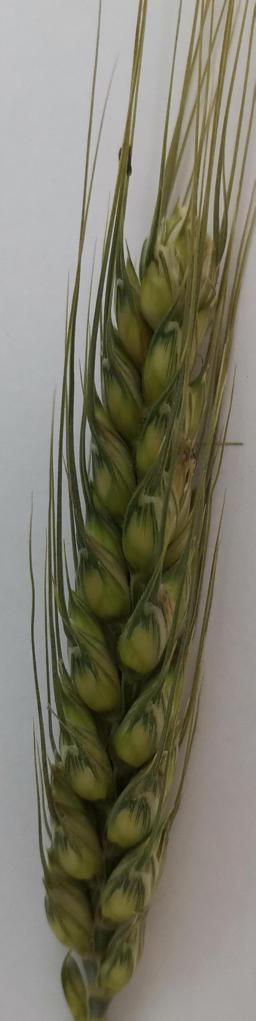

Supplement: Supplementary file 1 [file Data_Sheet_1.ZIP › 2. Datasets/1. training dataset for model training/Shenmai 818/5011.jpg]

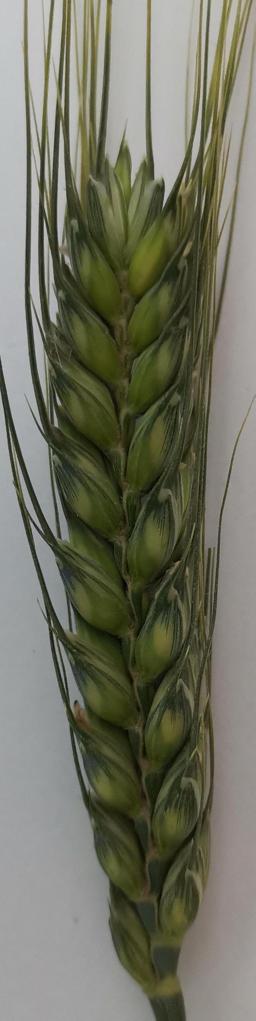

Supplement: Supplementary file 1 [file Data_Sheet_1.ZIP › 2. Datasets/1. training dataset for model training/Shenmai 818/5015.jpg]

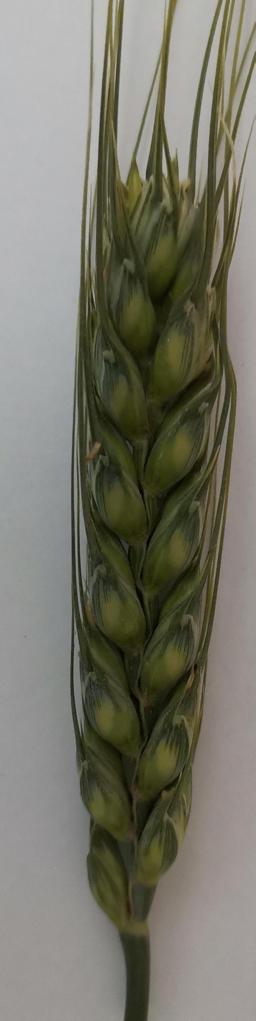

Supplement: Supplementary file 1 [file Data_Sheet_1.ZIP › 2. Datasets/1. training dataset for model training/Shenmai 818/5030.jpg]

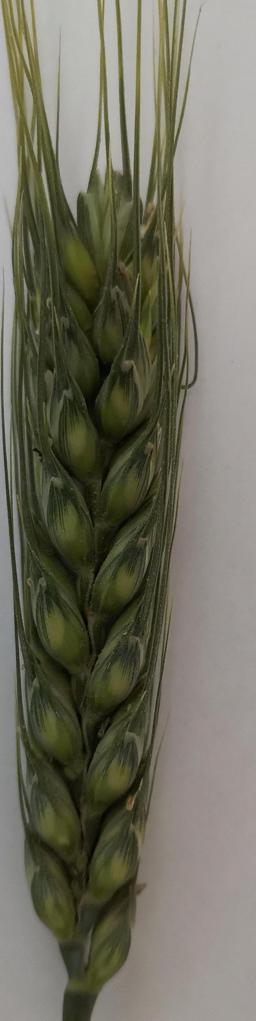

Supplement: Supplementary file 1 [file Data_Sheet_1.ZIP › 2. Datasets/1. training dataset for model training/Shenmai 818/5032.jpg]

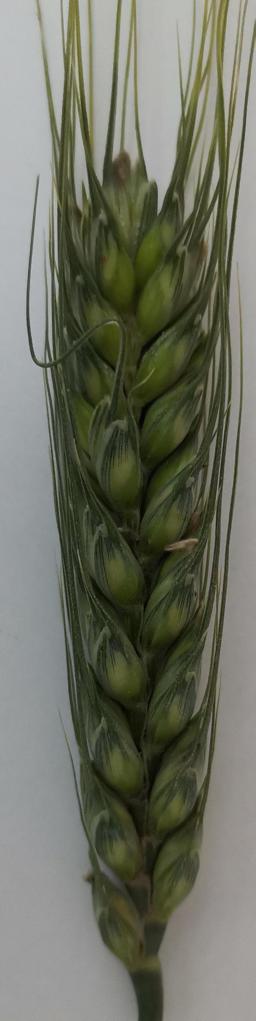

Supplement: Supplementary file 1 [file Data_Sheet_1.ZIP › 2. Datasets/1. training dataset for model training/Shenmai 818/5033.jpg]

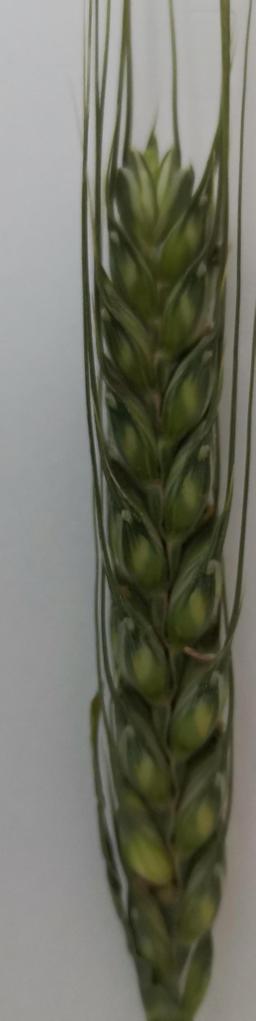

Supplement: Supplementary file 1 [file Data_Sheet_1.ZIP › 2. Datasets/1. training dataset for model training/Shenmai 818/5035.jpg]

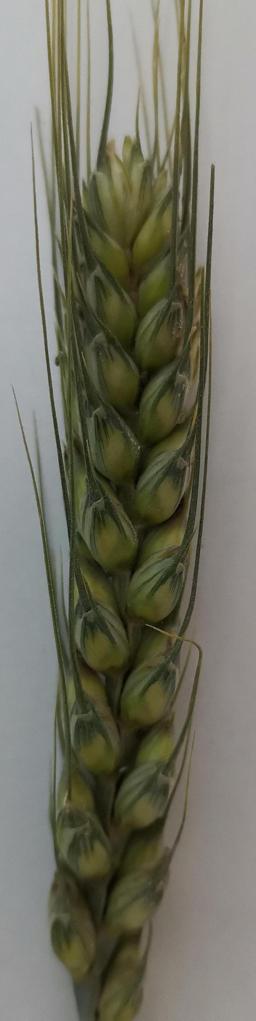

Supplement: Supplementary file 1 [file Data_Sheet_1.ZIP › 2. Datasets/1. training dataset for model training/Shenmai 818/5036.jpg]

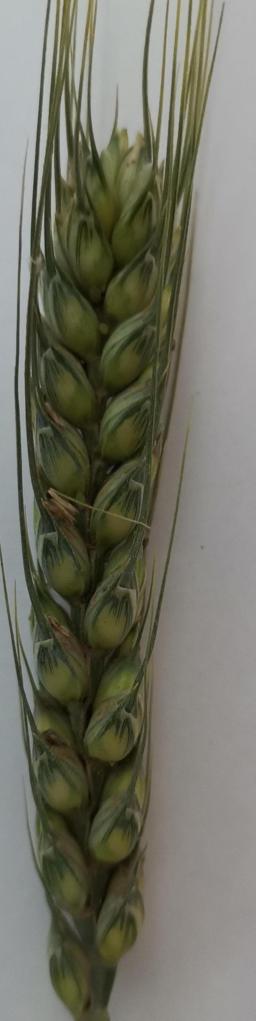

Supplement: Supplementary file 1 [file Data_Sheet_1.ZIP › 2. Datasets/1. training dataset for model training/Shenmai 818/5037.jpg]

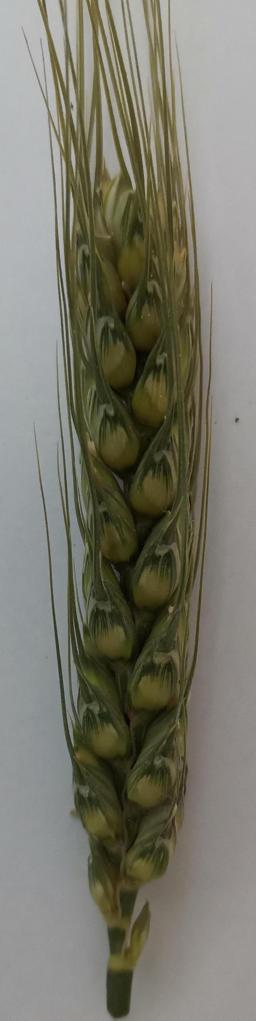

Supplement: Supplementary file 1 [file Data_Sheet_1.ZIP › 2. Datasets/1. training dataset for model training/Shenmai 818/5039.jpg]

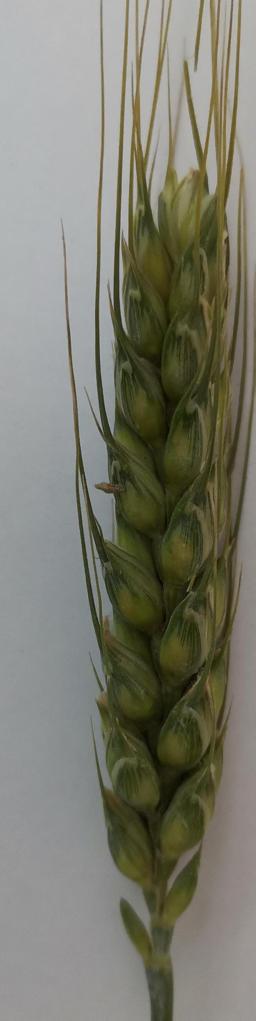

Supplement: Supplementary file 1 [file Data_Sheet_1.ZIP › 2. Datasets/1. training dataset for model training/Shenmai 818/5043.jpg]

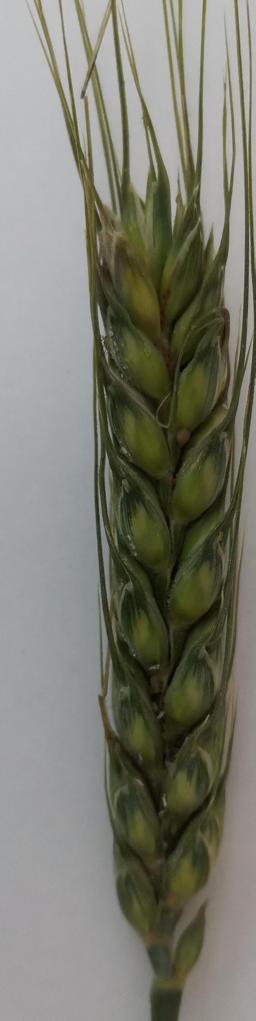

Supplement: Supplementary file 1 [file Data_Sheet_1.ZIP › 2. Datasets/1. training dataset for model training/Shenmai 818/5048.jpg]

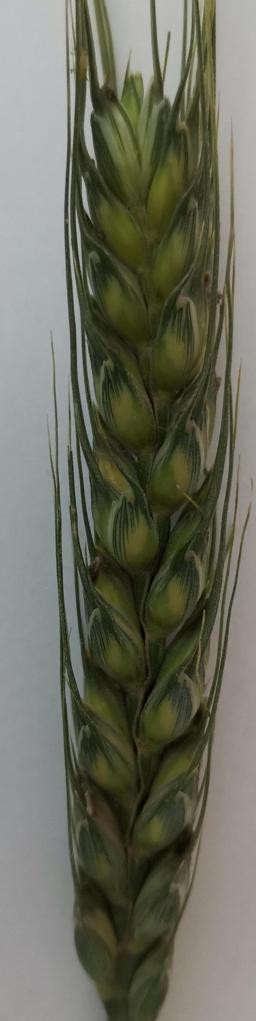

Supplement: Supplementary file 1 [file Data_Sheet_1.ZIP › 2. Datasets/1. training dataset for model training/Shenmai 818/5051.jpg]

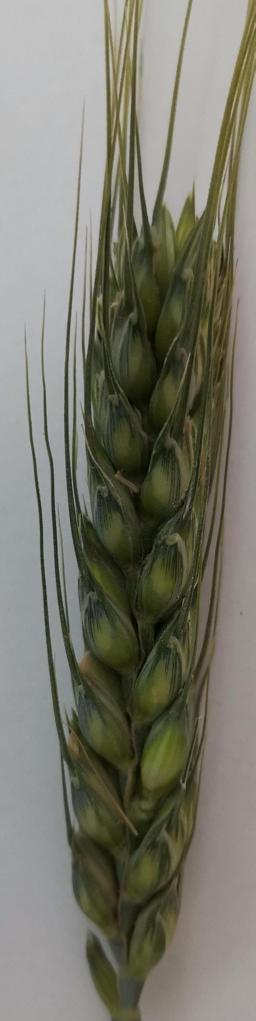

Supplement: Supplementary file 1 [file Data_Sheet_1.ZIP › 2. Datasets/1. training dataset for model training/Shenmai 818/5052.jpg]

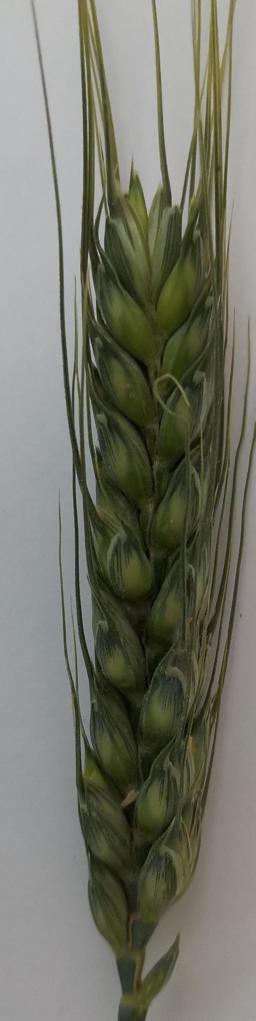

Supplement: Supplementary file 1 [file Data_Sheet_1.ZIP › 2. Datasets/1. training dataset for model training/Shenmai 818/5053.jpg]

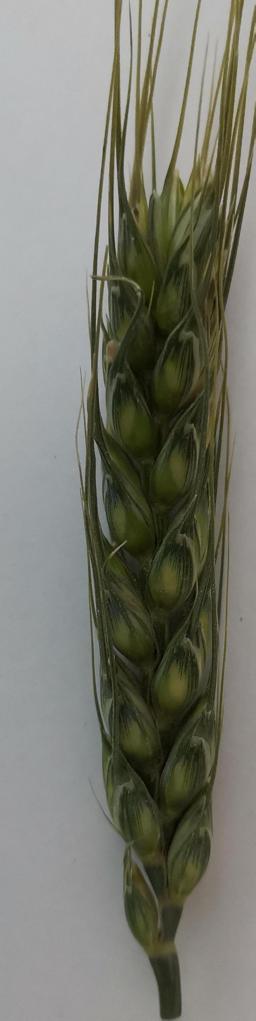

Supplement: Supplementary file 1 [file Data_Sheet_1.ZIP › 2. Datasets/1. training dataset for model training/Shenmai 818/5056.jpg]

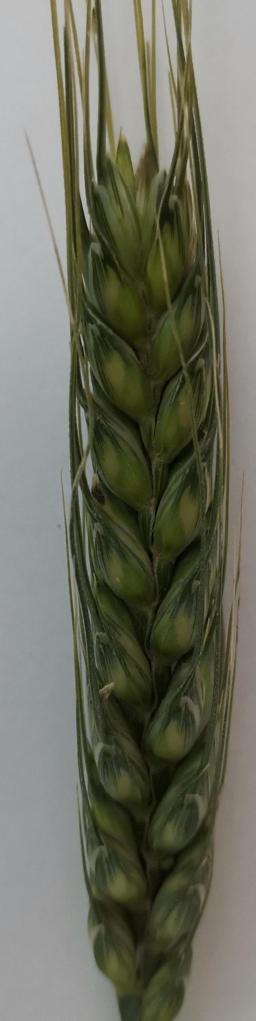

Supplement: Supplementary file 1 [file Data_Sheet_1.ZIP › 2. Datasets/1. training dataset for model training/Shenmai 818/5057.jpg]

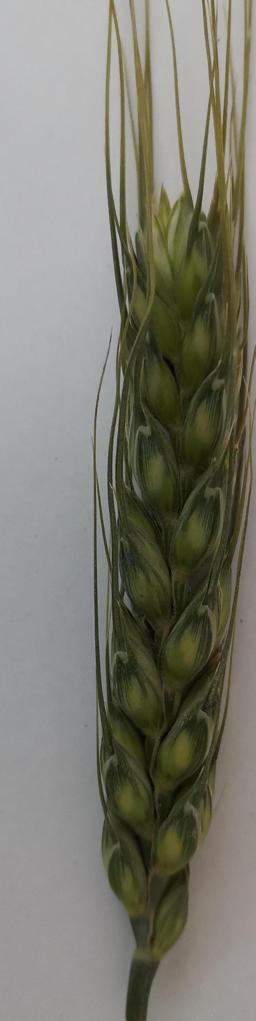

Supplement: Supplementary file 1 [file Data_Sheet_1.ZIP › 2. Datasets/1. training dataset for model training/Shenmai 818/5058.jpg]

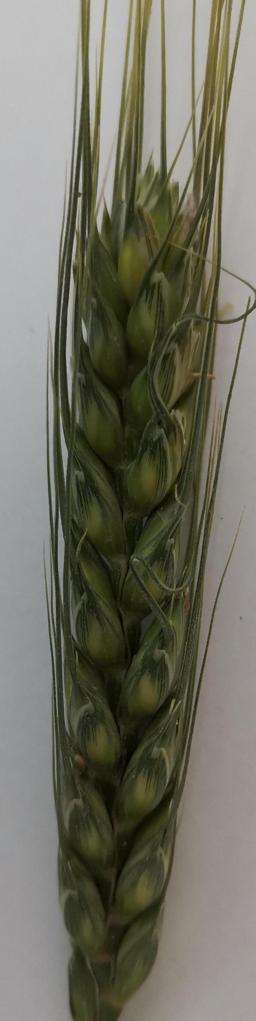

Supplement: Supplementary file 1 [file Data_Sheet_1.ZIP › 2. Datasets/1. training dataset for model training/Shenmai 818/5059.jpg]

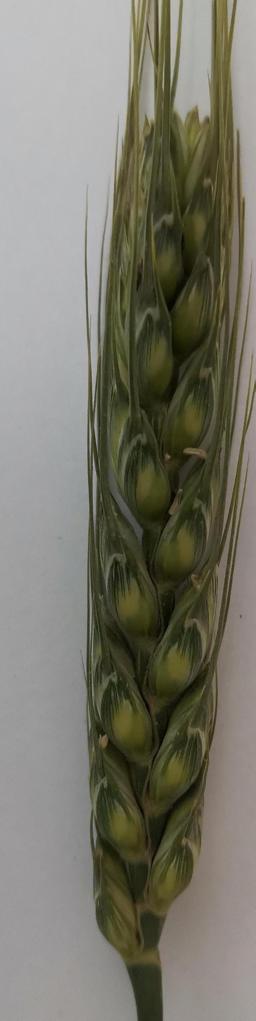

Supplement: Supplementary file 1 [file Data_Sheet_1.ZIP › 2. Datasets/1. training dataset for model training/Shenmai 818/5061.jpg]

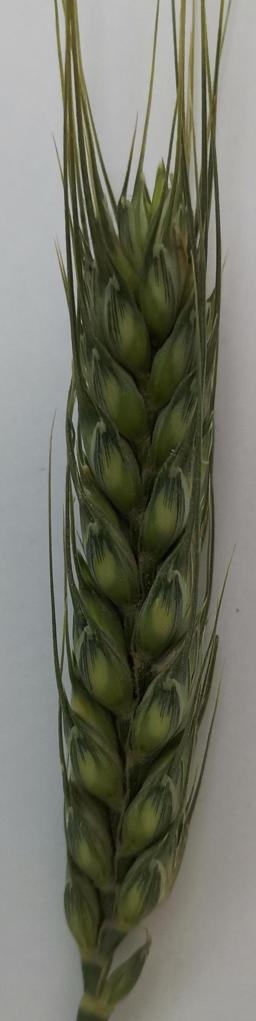

Supplement: Supplementary file 1 [file Data_Sheet_1.ZIP › 2. Datasets/1. training dataset for model training/Shenmai 818/5064.jpg]

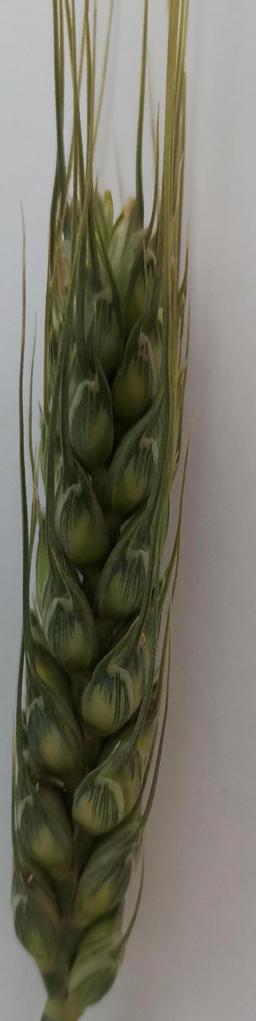

Supplement: Supplementary file 1 [file Data_Sheet_1.ZIP › 2. Datasets/1. training dataset for model training/Shenmai 818/5068.jpg]

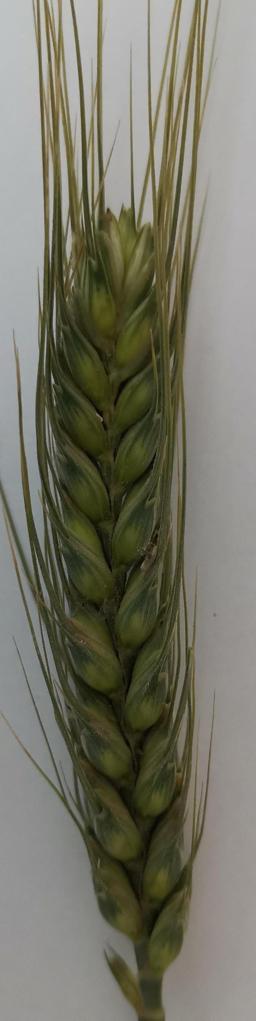

Supplement: Supplementary file 1 [file Data_Sheet_1.ZIP › 2. Datasets/1. training dataset for model training/Shenmai 818/5069.jpg]

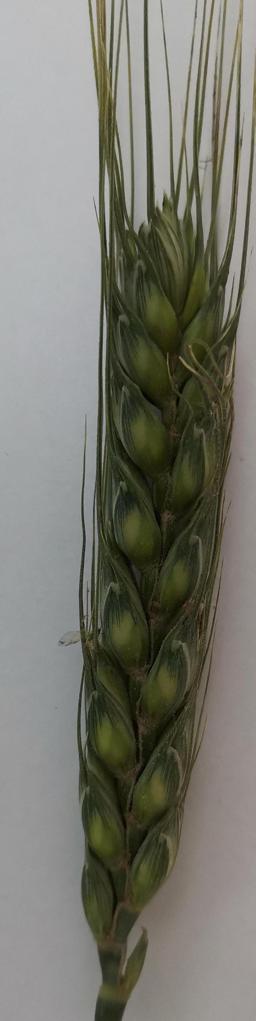

Supplement: Supplementary file 1 [file Data_Sheet_1.ZIP › 2. Datasets/1. training dataset for model training/Shenmai 818/5070.jpg]

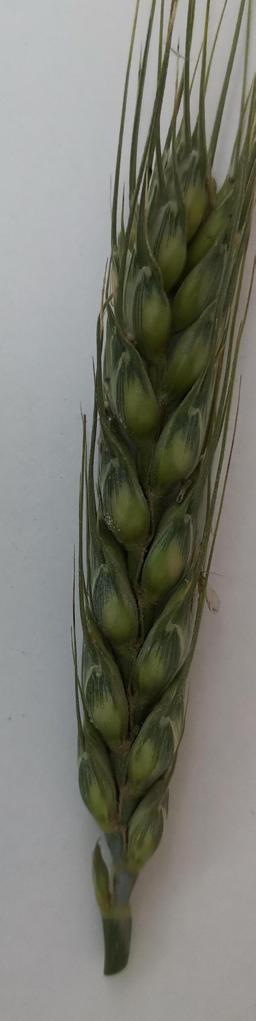

Supplement: Supplementary file 1 [file Data_Sheet_1.ZIP › 2. Datasets/1. training dataset for model training/Shenmai 818/5071.jpg]

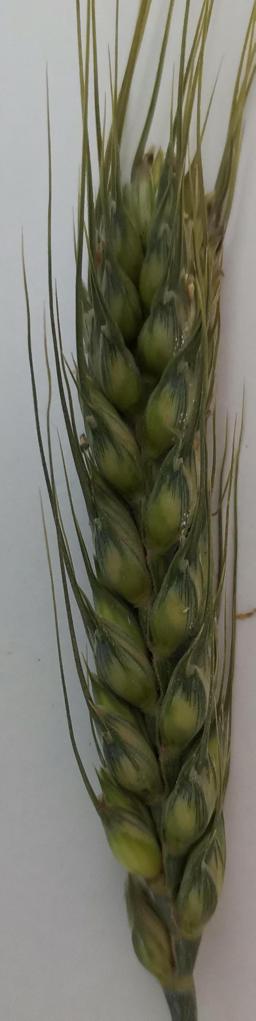

Supplement: Supplementary file 1 [file Data_Sheet_1.ZIP › 2. Datasets/1. training dataset for model training/Shenmai 818/5073.jpg]

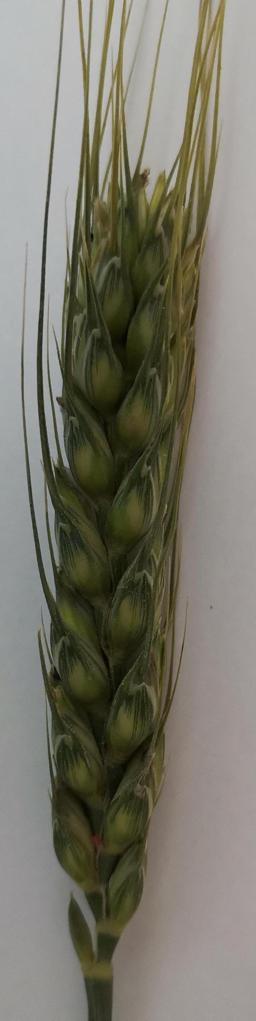

Supplement: Supplementary file 1 [file Data_Sheet_1.ZIP › 2. Datasets/1. training dataset for model training/Shenmai 818/5074.jpg]

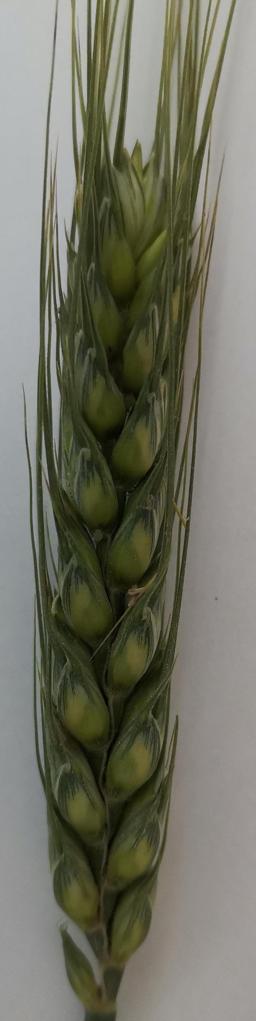

Supplement: Supplementary file 1 [file Data_Sheet_1.ZIP › 2. Datasets/1. training dataset for model training/Shenmai 818/5077.jpg]

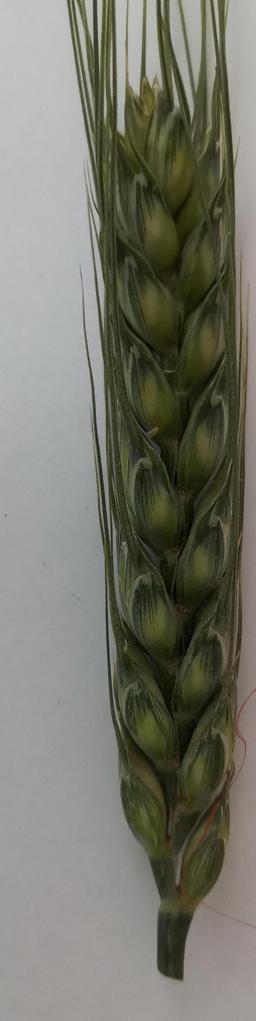

Supplement: Supplementary file 1 [file Data_Sheet_1.ZIP › 2. Datasets/1. training dataset for model training/Shenmai 818/5080.jpg]

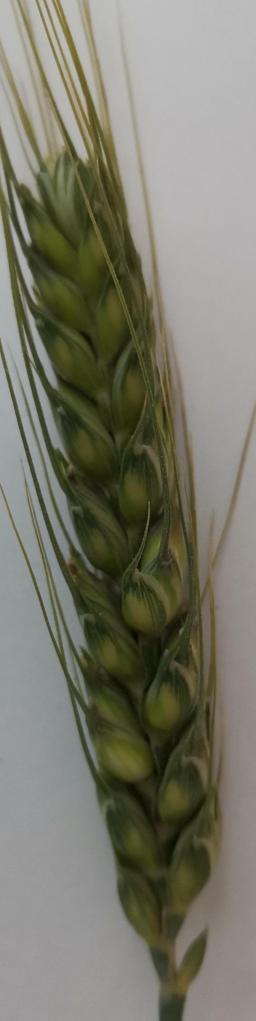

Supplement: Supplementary file 1 [file Data_Sheet_1.ZIP › 2. Datasets/1. training dataset for model training/Shenmai 818/5082.jpg]

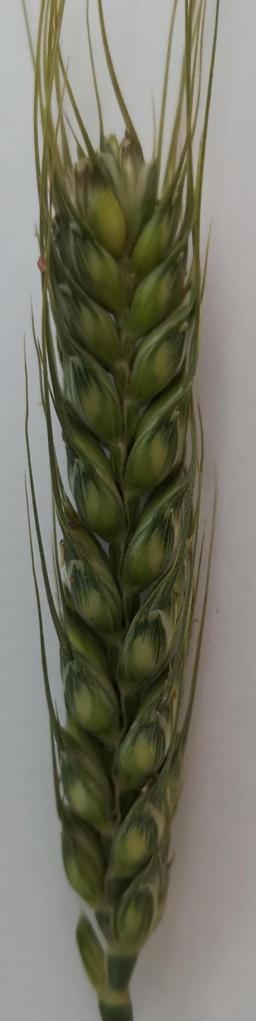

Supplement: Supplementary file 1 [file Data_Sheet_1.ZIP › 2. Datasets/1. training dataset for model training/Shenmai 818/5085.jpg]

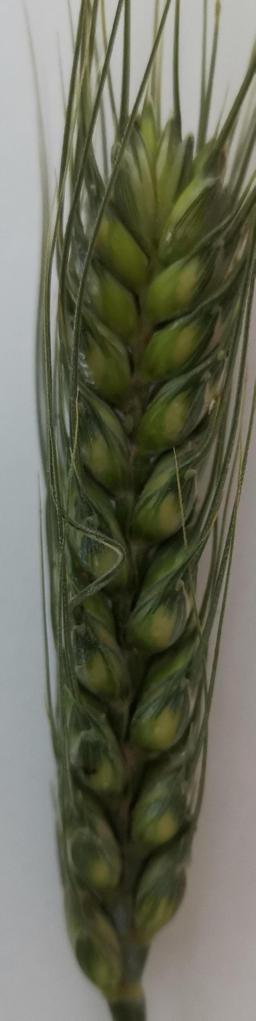

Supplement: Supplementary file 1 [file Data_Sheet_1.ZIP › 2. Datasets/1. training dataset for model training/Shenmai 818/5086.jpg]

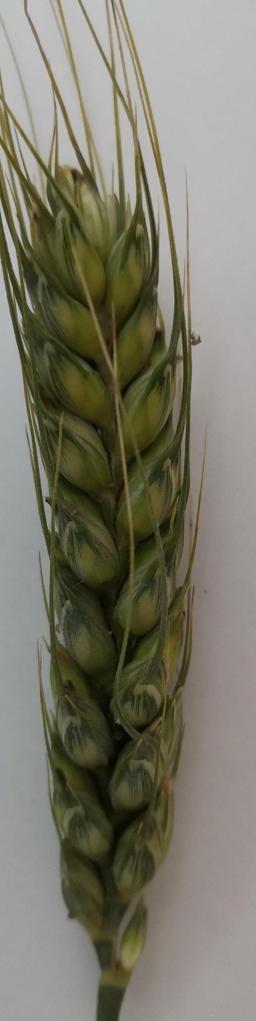

Supplement: Supplementary file 1 [file Data_Sheet_1.ZIP › 2. Datasets/1. training dataset for model training/Shenmai 818/5089.jpg]

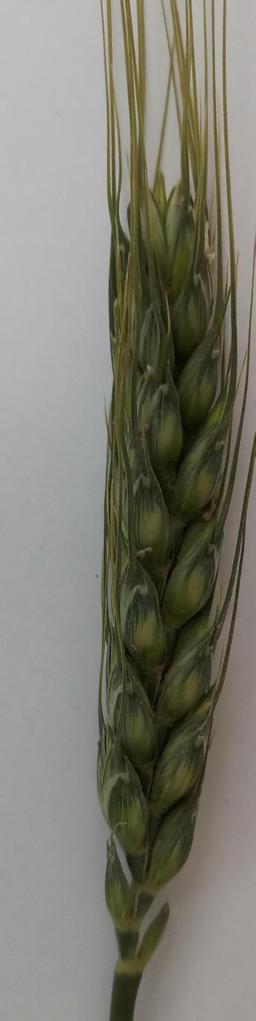

Supplement: Supplementary file 1 [file Data_Sheet_1.ZIP › 2. Datasets/1. training dataset for model training/Shenmai 818/5090.jpg]

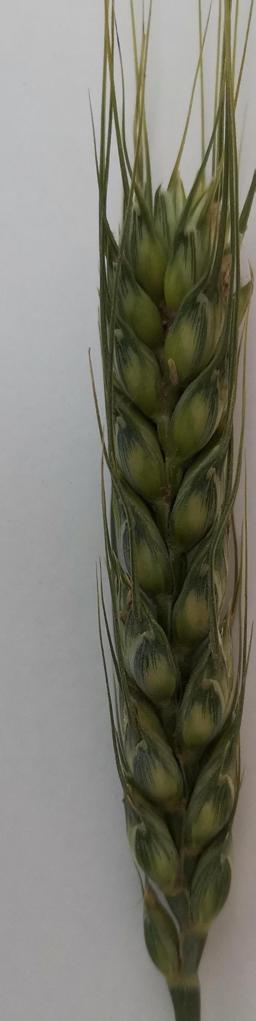

Supplement: Supplementary file 1 [file Data_Sheet_1.ZIP › 2. Datasets/1. training dataset for model training/Shenmai 818/5092.jpg]

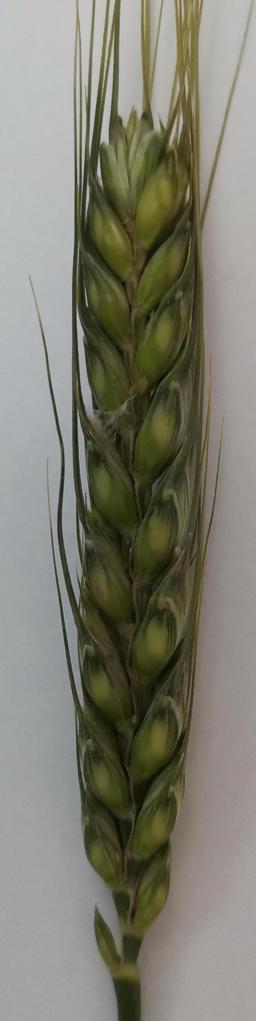

Supplement: Supplementary file 1 [file Data_Sheet_1.ZIP › 2. Datasets/1. training dataset for model training/Shenmai 818/5094.jpg]

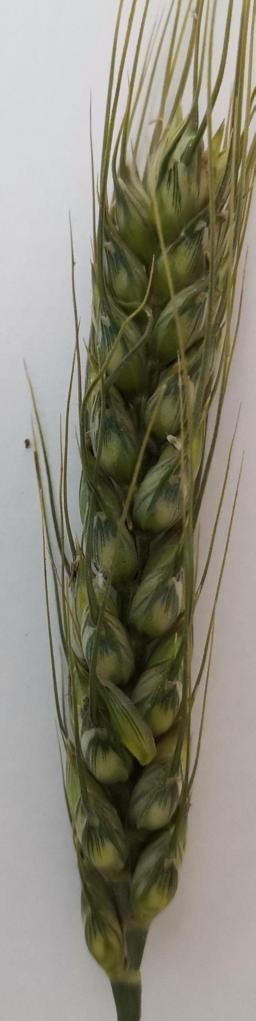

Supplement: Supplementary file 1 [file Data_Sheet_1.ZIP › 2. Datasets/1. training dataset for model training/Shenmai 818/5095.jpg]

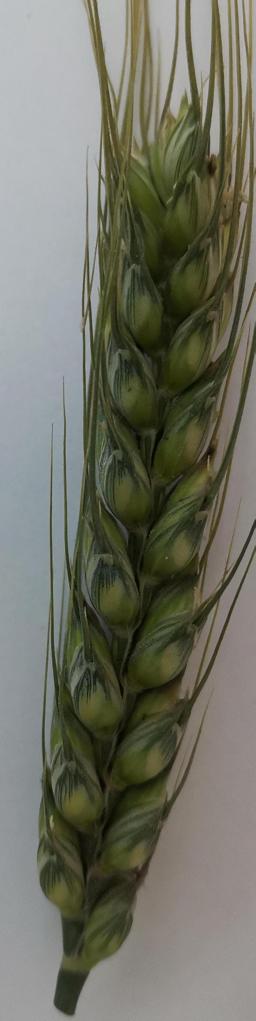

Supplement: Supplementary file 1 [file Data_Sheet_1.ZIP › 2. Datasets/1. training dataset for model training/Shenmai 818/5096.jpg]

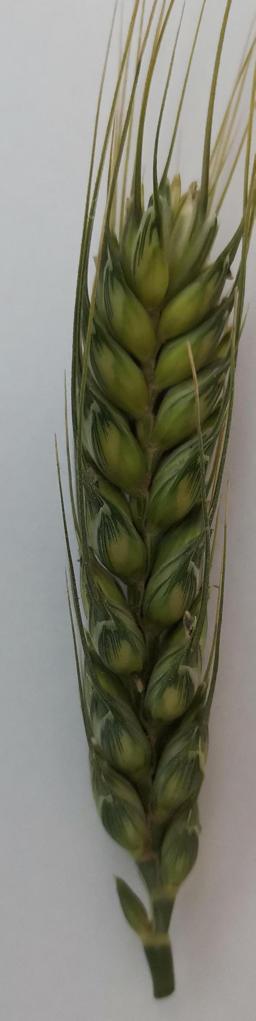

Supplement: Supplementary file 1 [file Data_Sheet_1.ZIP › 2. Datasets/1. training dataset for model training/Shenmai 818/5097.jpg]
