# Supplementary figures and images for: Automatic Detection and Counting of Wheat Spikelet Using Semi-Automatic Labeling and Deep Learning (part 1 of 8)
Source: Front Plant Sci. 2022 May 30;13:872555. doi: 10.3389/fpls.2022.872555 (PMC9189412; doi:10.3389/fpls.2022.872555)

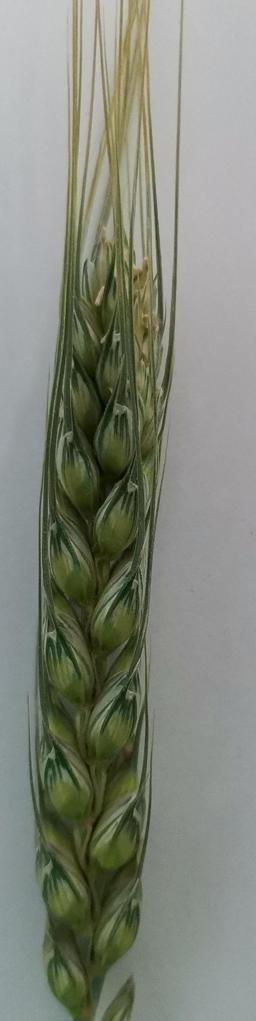

Supplement: Supplementary file 1 [file Data_Sheet_1.ZIP › 2. Datasets/1. training dataset for model training/Liangxing 99/3005.jpg]

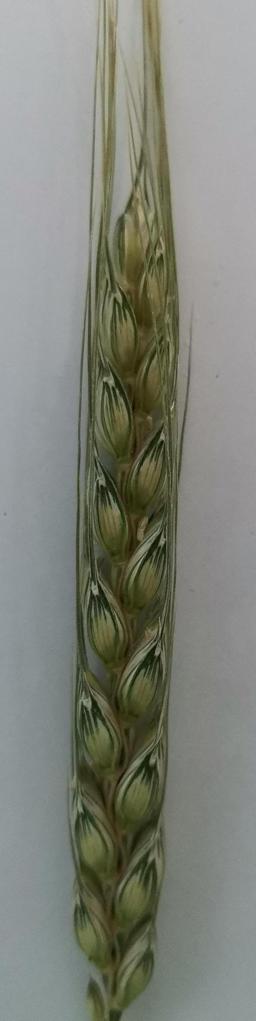

Supplement: Supplementary file 1 [file Data_Sheet_1.ZIP › 2. Datasets/1. training dataset for model training/Liangxing 99/3009.jpg]

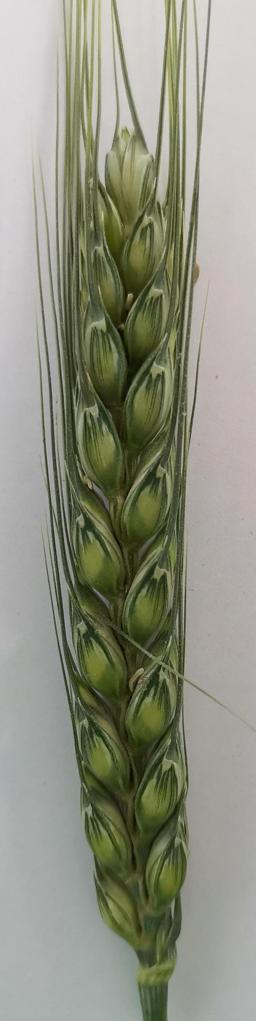

Supplement: Supplementary file 1 [file Data_Sheet_1.ZIP › 2. Datasets/1. training dataset for model training/Liangxing 99/3010.jpg]

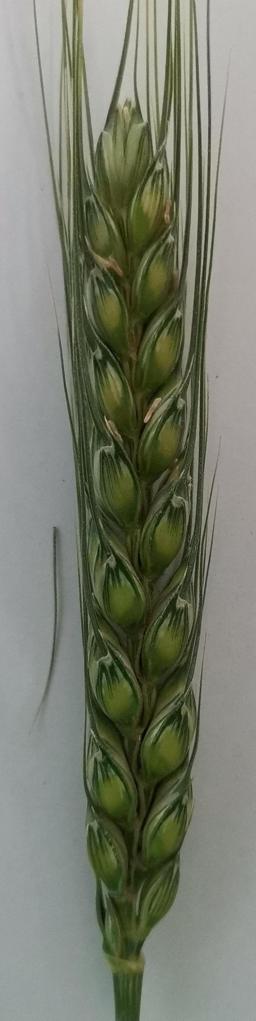

Supplement: Supplementary file 1 [file Data_Sheet_1.ZIP › 2. Datasets/1. training dataset for model training/Liangxing 99/3011.jpg]

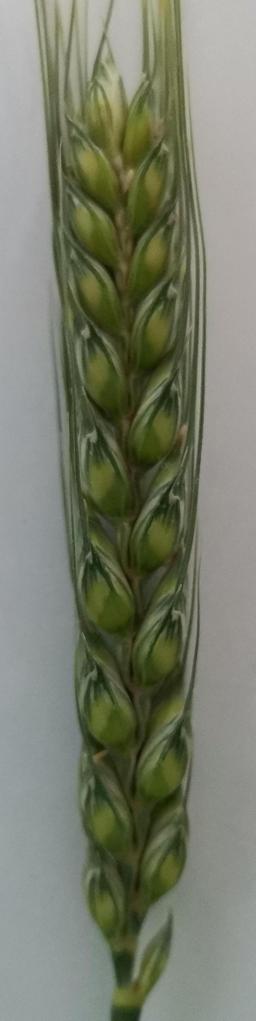

Supplement: Supplementary file 1 [file Data_Sheet_1.ZIP › 2. Datasets/1. training dataset for model training/Liangxing 99/3014.jpg]

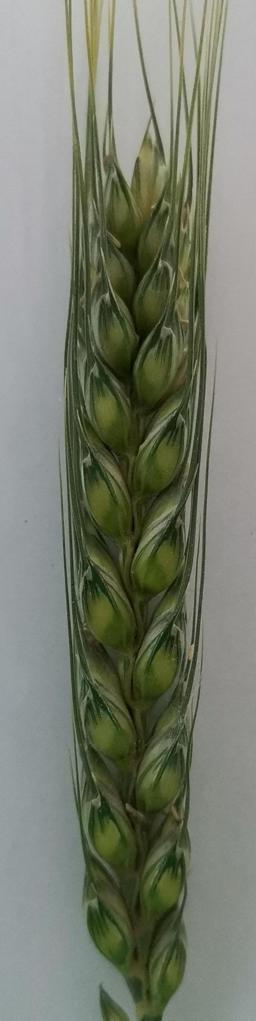

Supplement: Supplementary file 1 [file Data_Sheet_1.ZIP › 2. Datasets/1. training dataset for model training/Liangxing 99/3015.jpg]

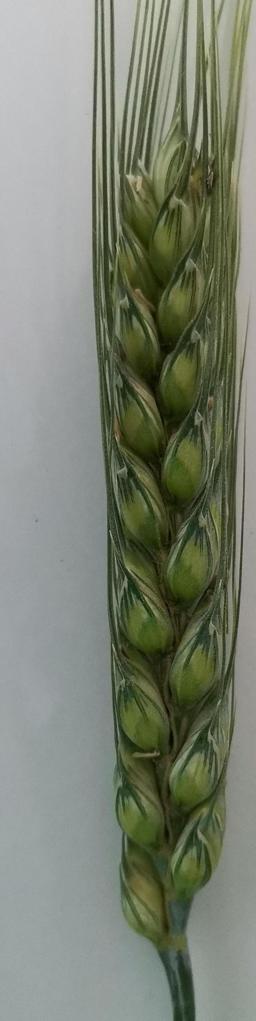

Supplement: Supplementary file 1 [file Data_Sheet_1.ZIP › 2. Datasets/1. training dataset for model training/Liangxing 99/3018.jpg]

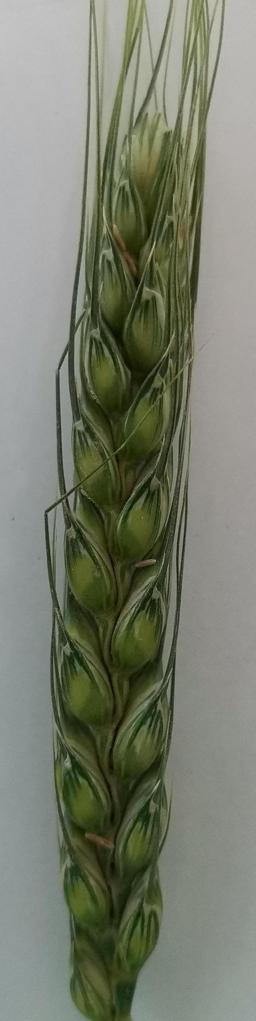

Supplement: Supplementary file 1 [file Data_Sheet_1.ZIP › 2. Datasets/1. training dataset for model training/Liangxing 99/3020.jpg]

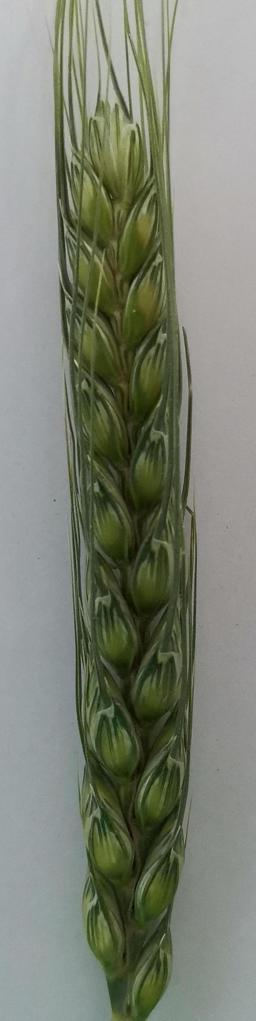

Supplement: Supplementary file 1 [file Data_Sheet_1.ZIP › 2. Datasets/1. training dataset for model training/Liangxing 99/3021.jpg]

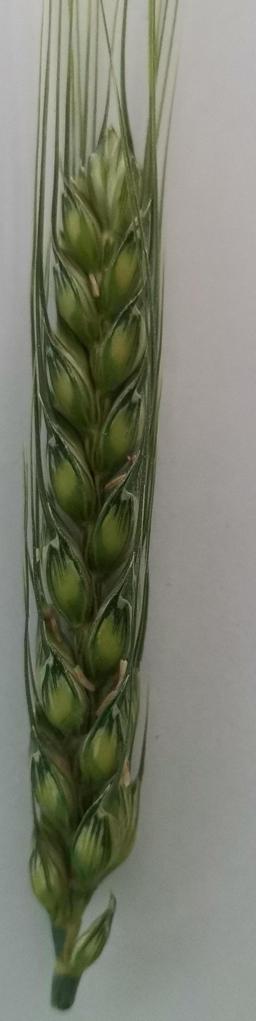

Supplement: Supplementary file 1 [file Data_Sheet_1.ZIP › 2. Datasets/1. training dataset for model training/Liangxing 99/3022.jpg]

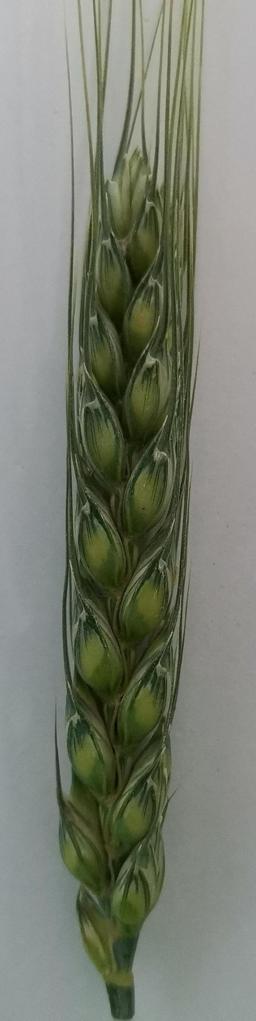

Supplement: Supplementary file 1 [file Data_Sheet_1.ZIP › 2. Datasets/1. training dataset for model training/Liangxing 99/3023.jpg]

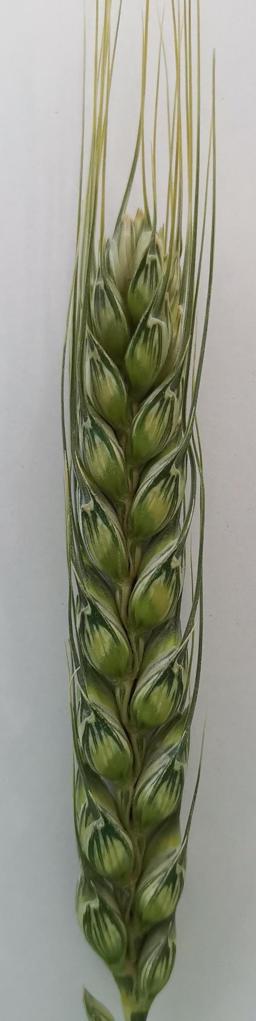

Supplement: Supplementary file 1 [file Data_Sheet_1.ZIP › 2. Datasets/1. training dataset for model training/Liangxing 99/3027.jpg]

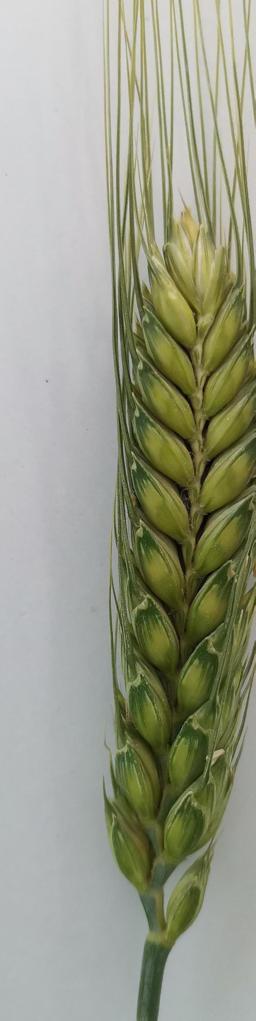

Supplement: Supplementary file 1 [file Data_Sheet_1.ZIP › 2. Datasets/1. training dataset for model training/Liangxing 99/3030.jpg]

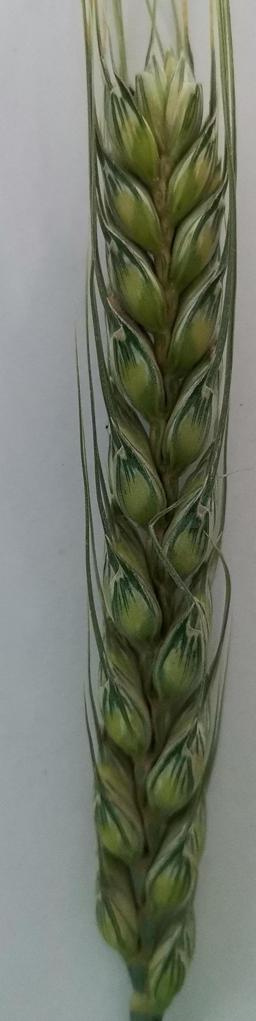

Supplement: Supplementary file 1 [file Data_Sheet_1.ZIP › 2. Datasets/1. training dataset for model training/Liangxing 99/3033.jpg]

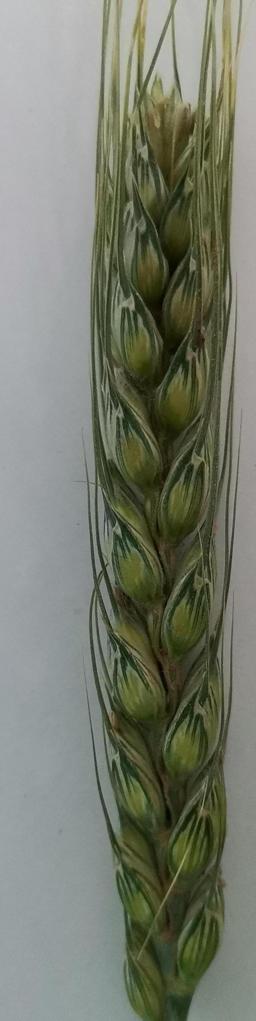

Supplement: Supplementary file 1 [file Data_Sheet_1.ZIP › 2. Datasets/1. training dataset for model training/Liangxing 99/3034.jpg]

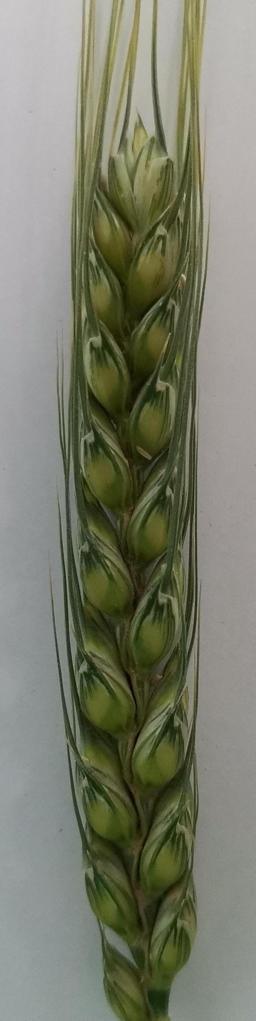

Supplement: Supplementary file 1 [file Data_Sheet_1.ZIP › 2. Datasets/1. training dataset for model training/Liangxing 99/3036.jpg]

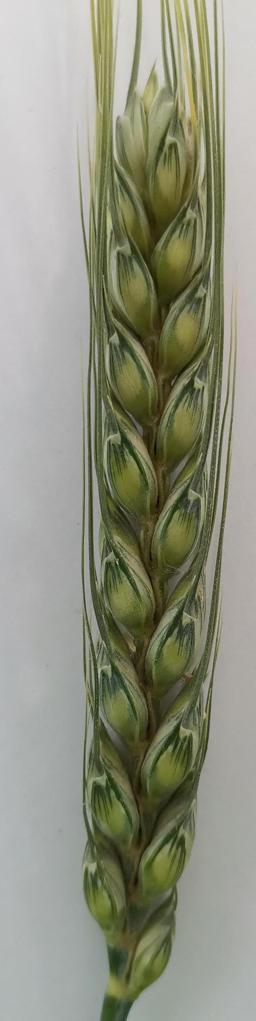

Supplement: Supplementary file 1 [file Data_Sheet_1.ZIP › 2. Datasets/1. training dataset for model training/Liangxing 99/3037.jpg]

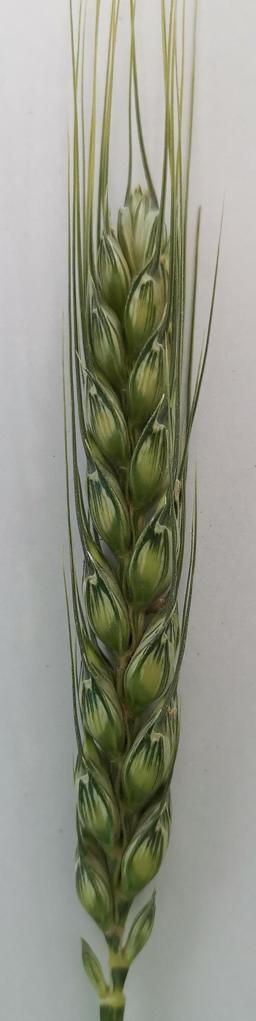

Supplement: Supplementary file 1 [file Data_Sheet_1.ZIP › 2. Datasets/1. training dataset for model training/Liangxing 99/3039.jpg]

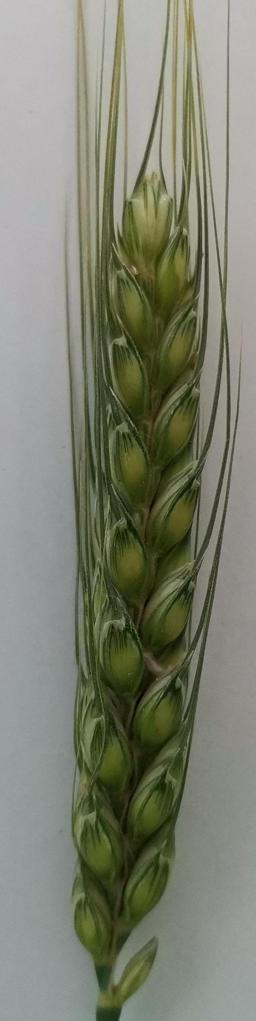

Supplement: Supplementary file 1 [file Data_Sheet_1.ZIP › 2. Datasets/1. training dataset for model training/Liangxing 99/3040.jpg]

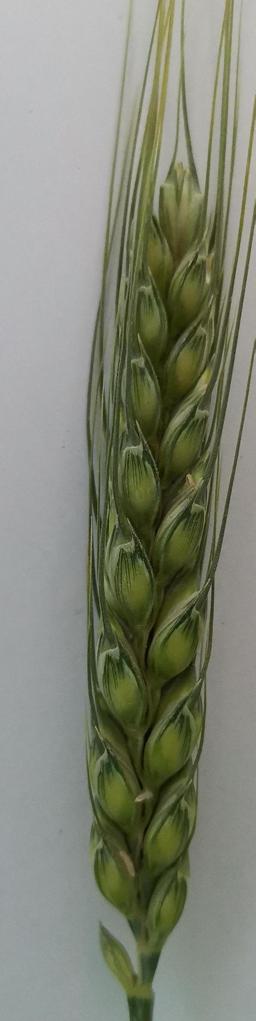

Supplement: Supplementary file 1 [file Data_Sheet_1.ZIP › 2. Datasets/1. training dataset for model training/Liangxing 99/3041.jpg]

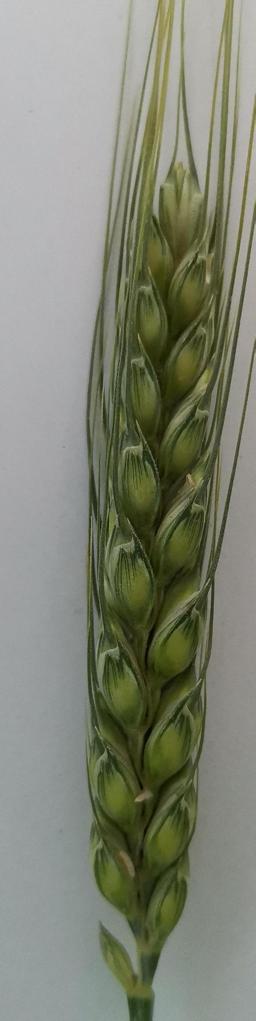

Supplement: Supplementary file 1 [file Data_Sheet_1.ZIP › 2. Datasets/1. training dataset for model training/Liangxing 99/3042.jpg]

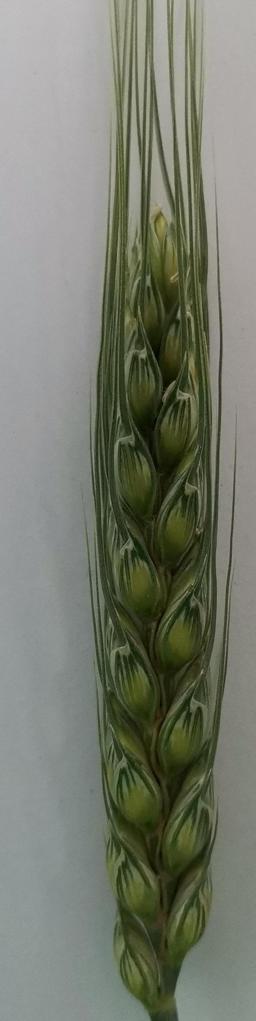

Supplement: Supplementary file 1 [file Data_Sheet_1.ZIP › 2. Datasets/1. training dataset for model training/Liangxing 99/3043.jpg]

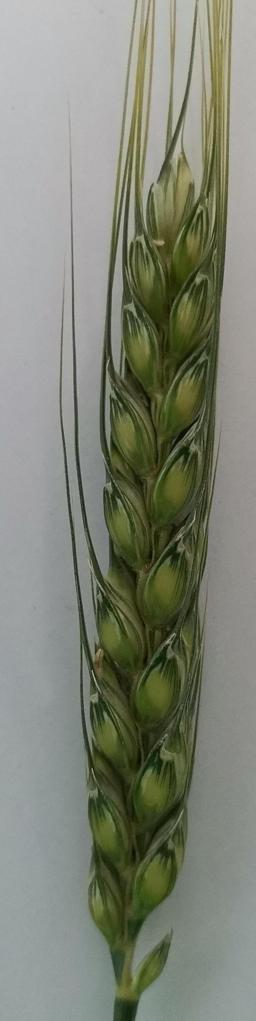

Supplement: Supplementary file 1 [file Data_Sheet_1.ZIP › 2. Datasets/1. training dataset for model training/Liangxing 99/3052.jpg]

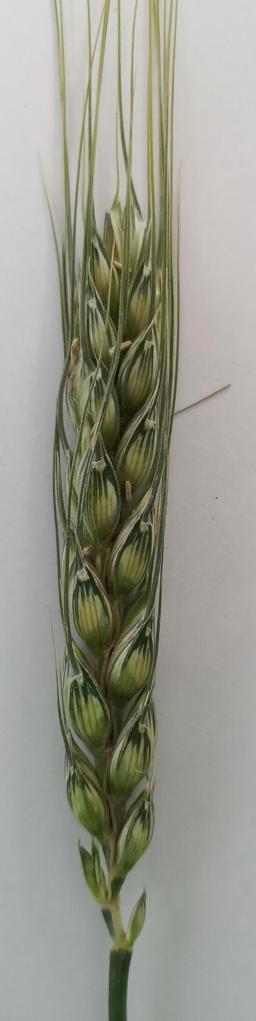

Supplement: Supplementary file 1 [file Data_Sheet_1.ZIP › 2. Datasets/1. training dataset for model training/Liangxing 99/3055.jpg]

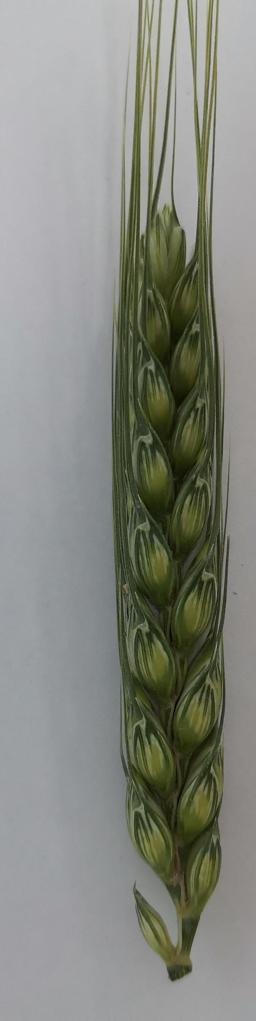

Supplement: Supplementary file 1 [file Data_Sheet_1.ZIP › 2. Datasets/1. training dataset for model training/Liangxing 99/3057.jpg]

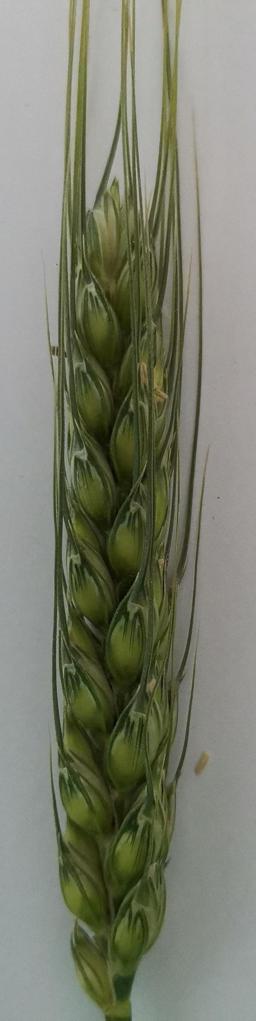

Supplement: Supplementary file 1 [file Data_Sheet_1.ZIP › 2. Datasets/1. training dataset for model training/Liangxing 99/3058.jpg]

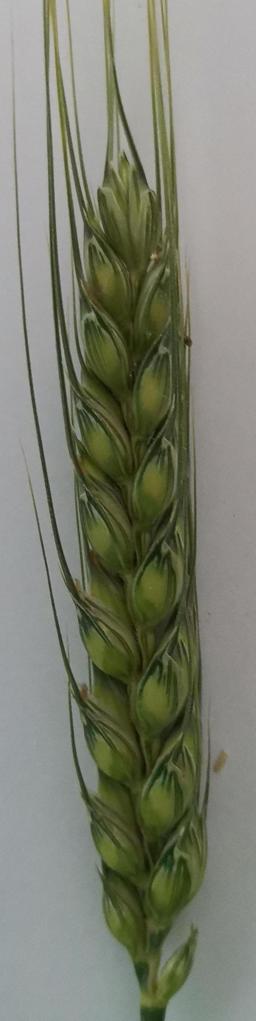

Supplement: Supplementary file 1 [file Data_Sheet_1.ZIP › 2. Datasets/1. training dataset for model training/Liangxing 99/3059.jpg]

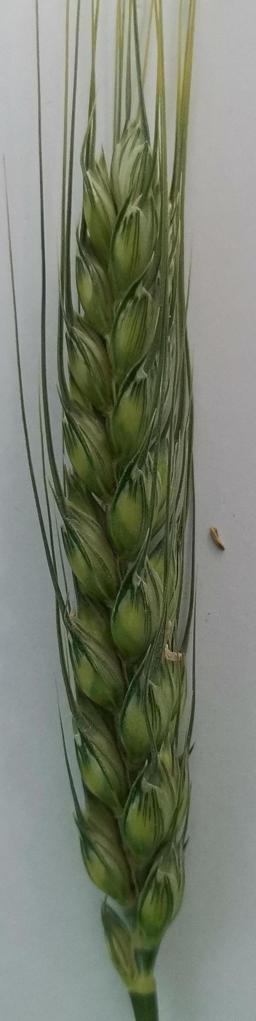

Supplement: Supplementary file 1 [file Data_Sheet_1.ZIP › 2. Datasets/1. training dataset for model training/Liangxing 99/3060.jpg]

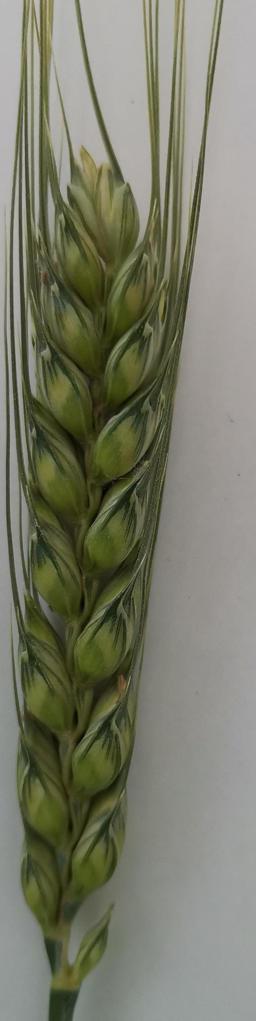

Supplement: Supplementary file 1 [file Data_Sheet_1.ZIP › 2. Datasets/1. training dataset for model training/Liangxing 99/3064.jpg]

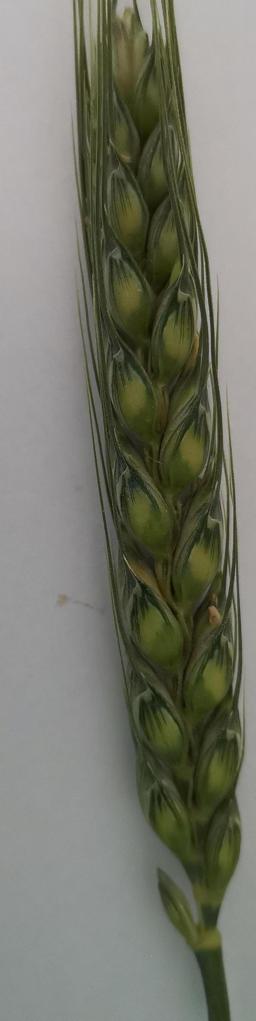

Supplement: Supplementary file 1 [file Data_Sheet_1.ZIP › 2. Datasets/1. training dataset for model training/Liangxing 99/3071.jpg]

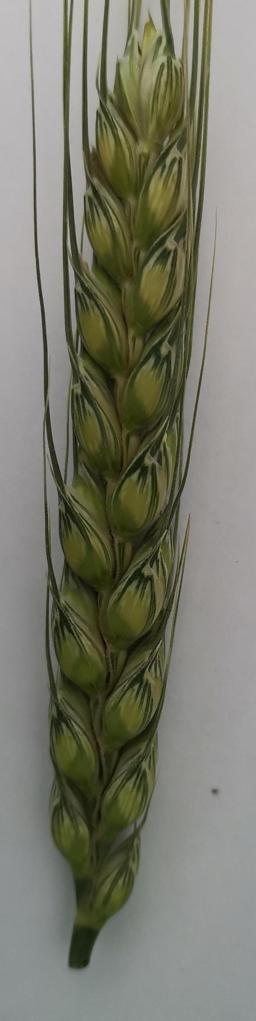

Supplement: Supplementary file 1 [file Data_Sheet_1.ZIP › 2. Datasets/1. training dataset for model training/Liangxing 99/3073.jpg]

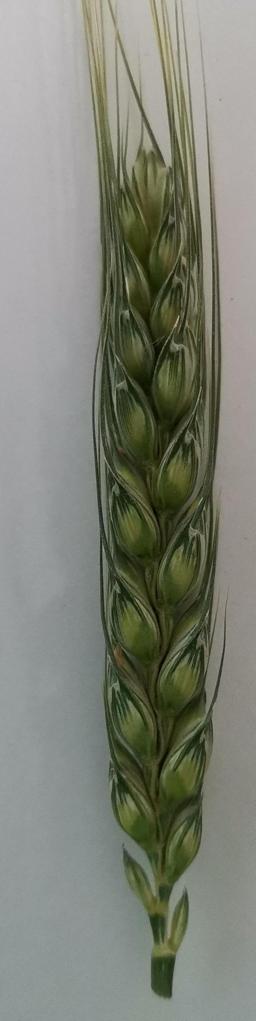

Supplement: Supplementary file 1 [file Data_Sheet_1.ZIP › 2. Datasets/1. training dataset for model training/Liangxing 99/3075.jpg]

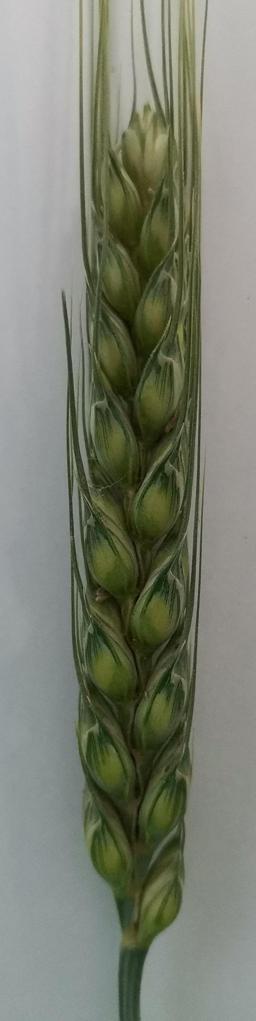

Supplement: Supplementary file 1 [file Data_Sheet_1.ZIP › 2. Datasets/1. training dataset for model training/Liangxing 99/3076.jpg]

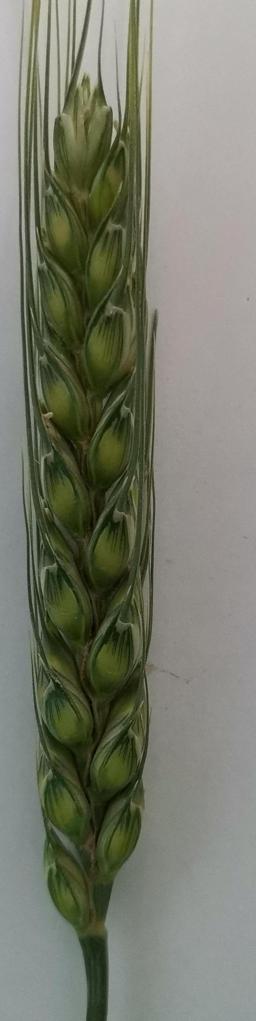

Supplement: Supplementary file 1 [file Data_Sheet_1.ZIP › 2. Datasets/1. training dataset for model training/Liangxing 99/3077.jpg]

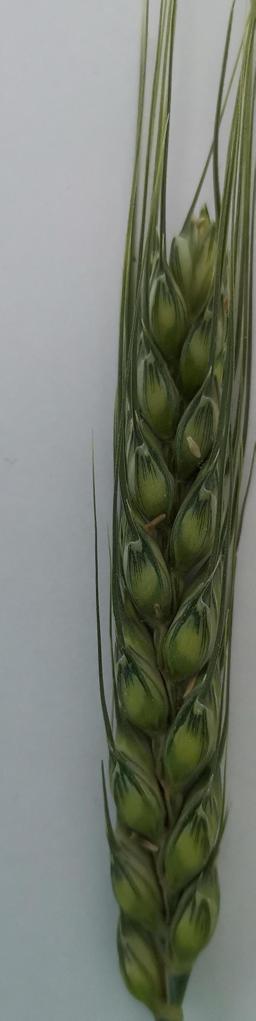

Supplement: Supplementary file 1 [file Data_Sheet_1.ZIP › 2. Datasets/1. training dataset for model training/Liangxing 99/3110.jpg]

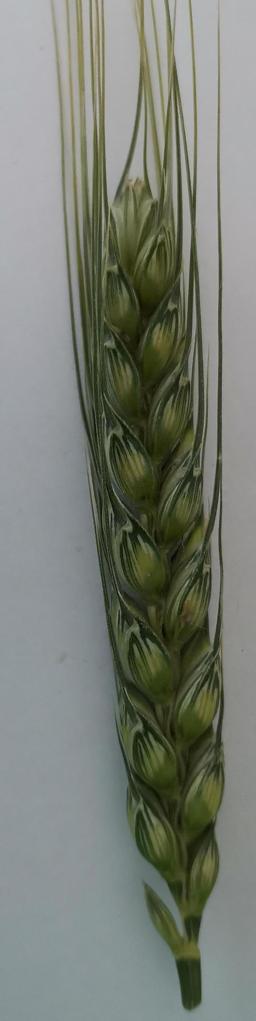

Supplement: Supplementary file 1 [file Data_Sheet_1.ZIP › 2. Datasets/1. training dataset for model training/Liangxing 99/3115.jpg]

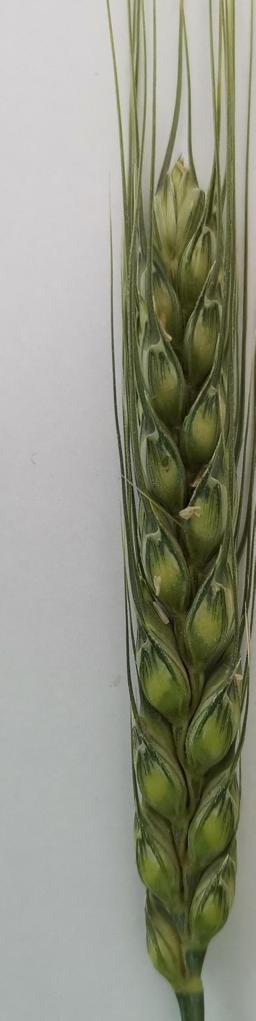

Supplement: Supplementary file 1 [file Data_Sheet_1.ZIP › 2. Datasets/1. training dataset for model training/Liangxing 99/3116.jpg]

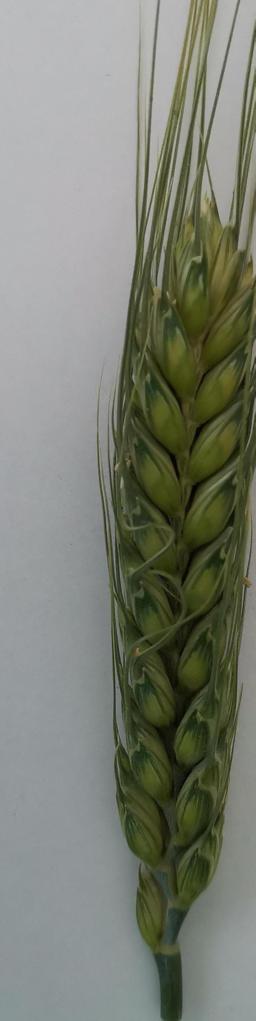

Supplement: Supplementary file 1 [file Data_Sheet_1.ZIP › 2. Datasets/1. training dataset for model training/Liangxing 99/3120.jpg]

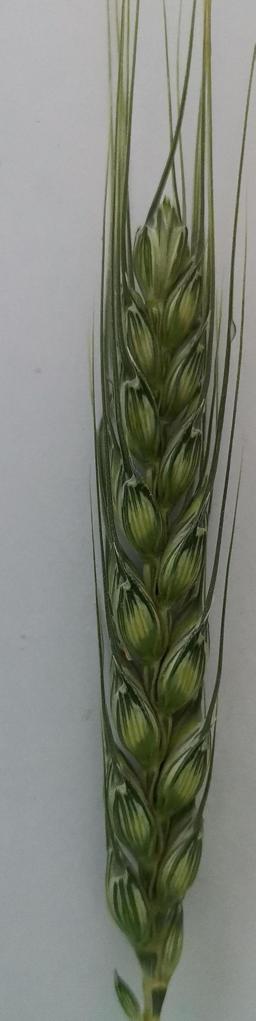

Supplement: Supplementary file 1 [file Data_Sheet_1.ZIP › 2. Datasets/1. training dataset for model training/Liangxing 99/3133.jpg]

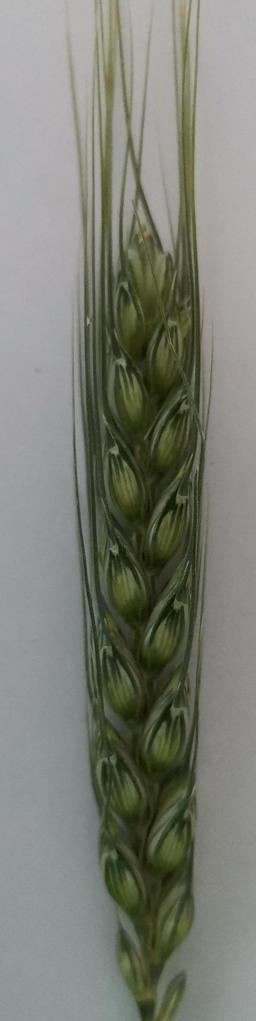

Supplement: Supplementary file 1 [file Data_Sheet_1.ZIP › 2. Datasets/1. training dataset for model training/Liangxing 99/3134.jpg]

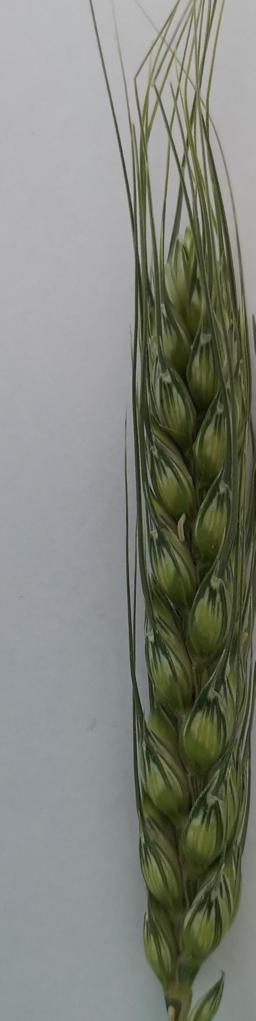

Supplement: Supplementary file 1 [file Data_Sheet_1.ZIP › 2. Datasets/1. training dataset for model training/Liangxing 99/3140.jpg]

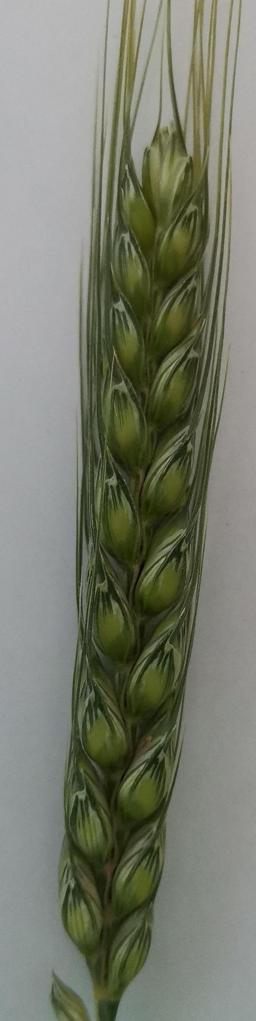

Supplement: Supplementary file 1 [file Data_Sheet_1.ZIP › 2. Datasets/1. training dataset for model training/Liangxing 99/3143.jpg]

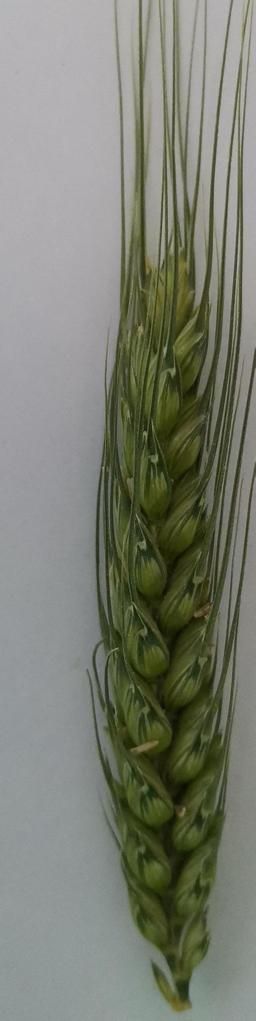

Supplement: Supplementary file 1 [file Data_Sheet_1.ZIP › 2. Datasets/1. training dataset for model training/Liangxing 99/3149.jpg]

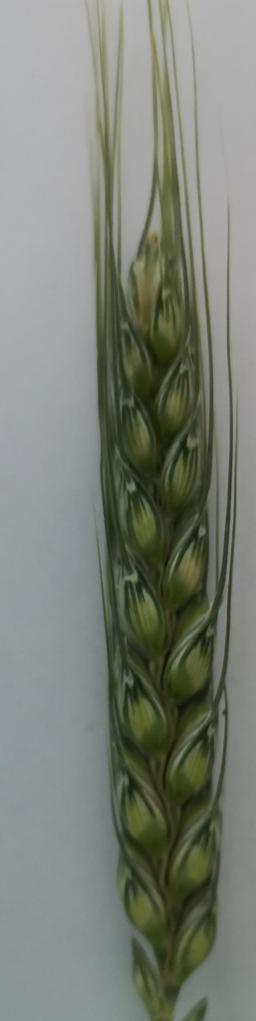

Supplement: Supplementary file 1 [file Data_Sheet_1.ZIP › 2. Datasets/1. training dataset for model training/Liangxing 99/3151.jpg]

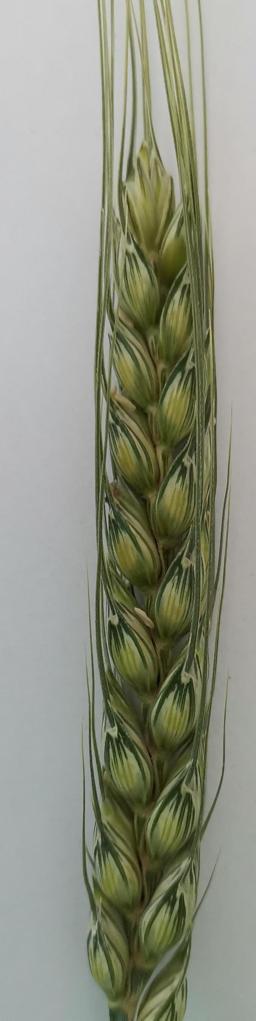

Supplement: Supplementary file 1 [file Data_Sheet_1.ZIP › 2. Datasets/1. training dataset for model training/Liangxing 99/3153.jpg]

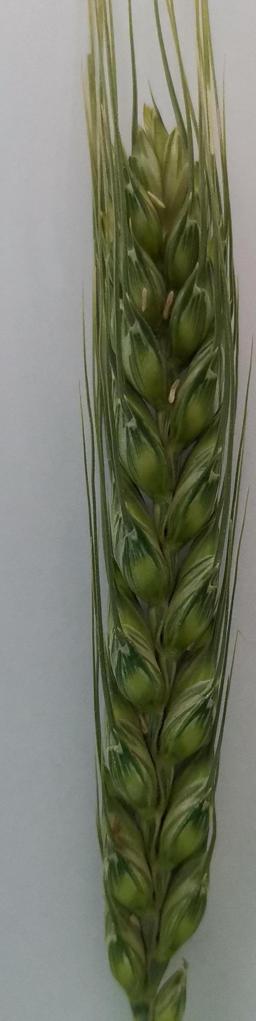

Supplement: Supplementary file 1 [file Data_Sheet_1.ZIP › 2. Datasets/1. training dataset for model training/Liangxing 99/3157.jpg]

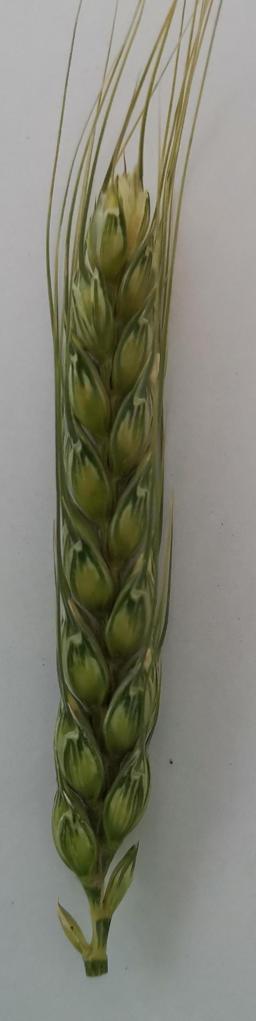

Supplement: Supplementary file 1 [file Data_Sheet_1.ZIP › 2. Datasets/1. training dataset for model training/Liangxing 99/3166.jpg]

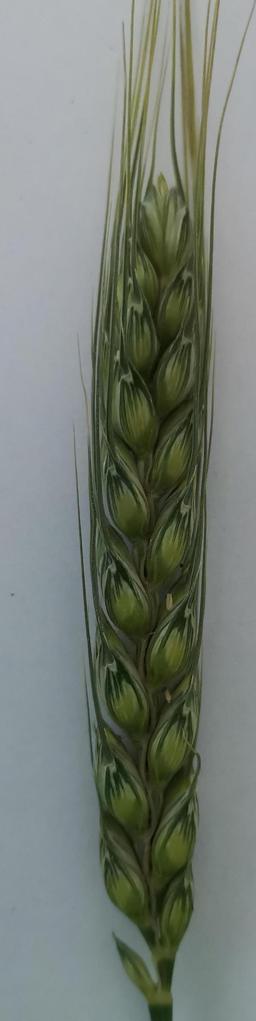

Supplement: Supplementary file 1 [file Data_Sheet_1.ZIP › 2. Datasets/1. training dataset for model training/Liangxing 99/3170.jpg]

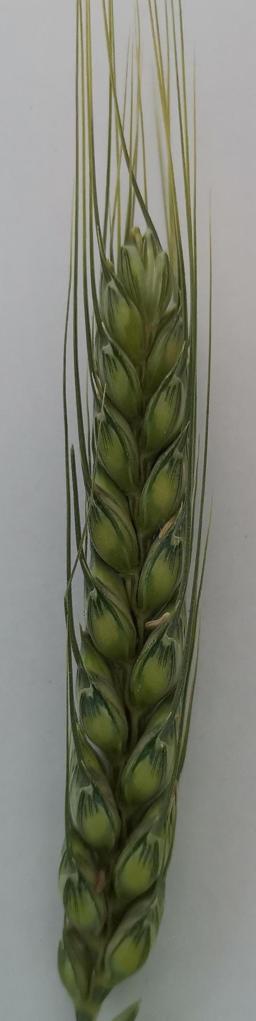

Supplement: Supplementary file 1 [file Data_Sheet_1.ZIP › 2. Datasets/1. training dataset for model training/Liangxing 99/3174.jpg]

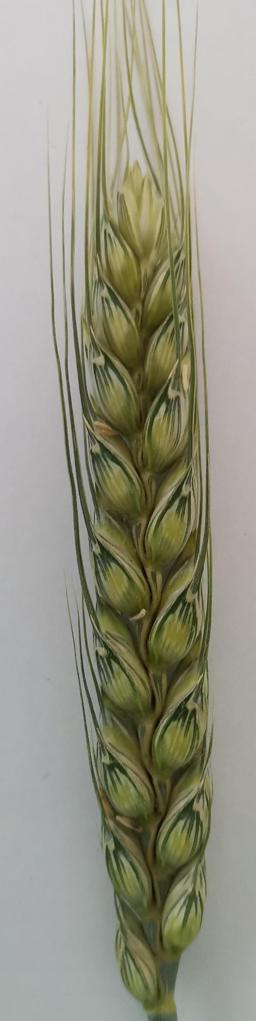

Supplement: Supplementary file 1 [file Data_Sheet_1.ZIP › 2. Datasets/1. training dataset for model training/Liangxing 99/3175.jpg]

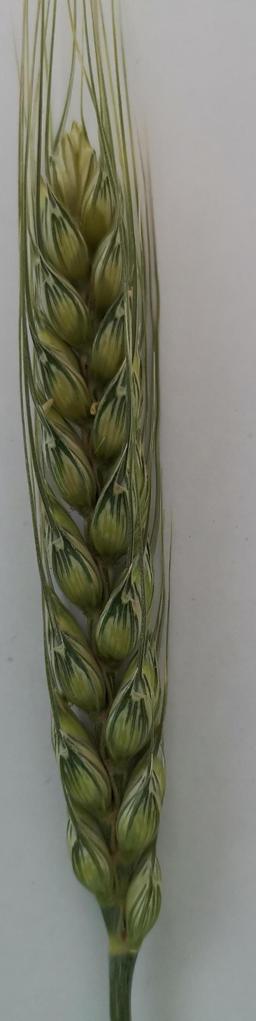

Supplement: Supplementary file 1 [file Data_Sheet_1.ZIP › 2. Datasets/1. training dataset for model training/Liangxing 99/3176.jpg]

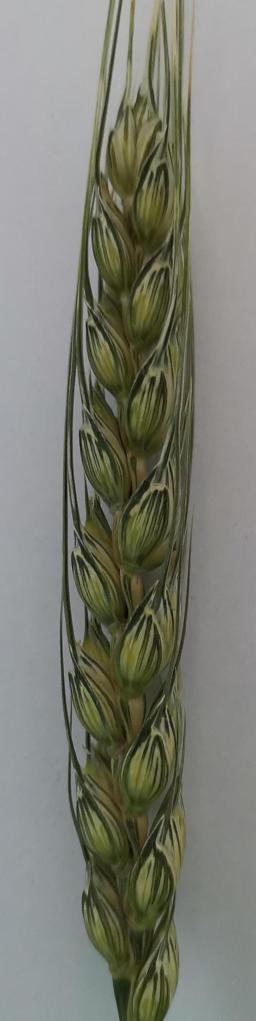

Supplement: Supplementary file 1 [file Data_Sheet_1.ZIP › 2. Datasets/1. training dataset for model training/Liangxing 99/3182.jpg]

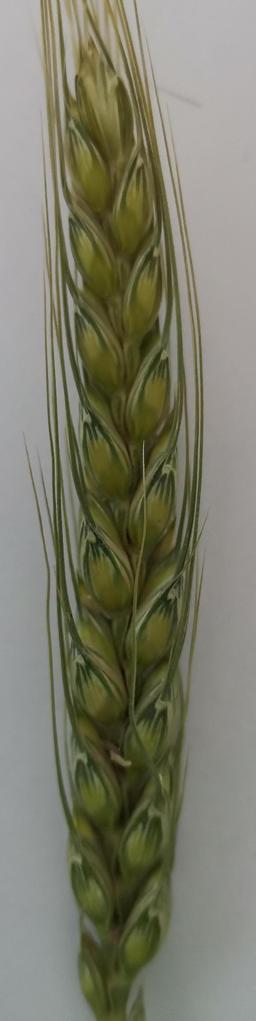

Supplement: Supplementary file 1 [file Data_Sheet_1.ZIP › 2. Datasets/1. training dataset for model training/Liangxing 99/3184.jpg]

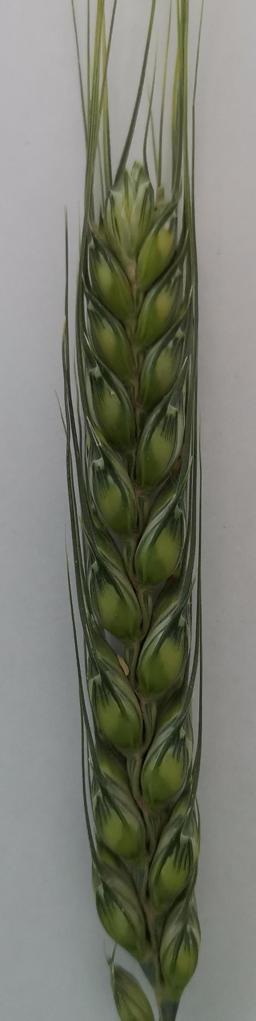

Supplement: Supplementary file 1 [file Data_Sheet_1.ZIP › 2. Datasets/1. training dataset for model training/Liangxing 99/3185.jpg]

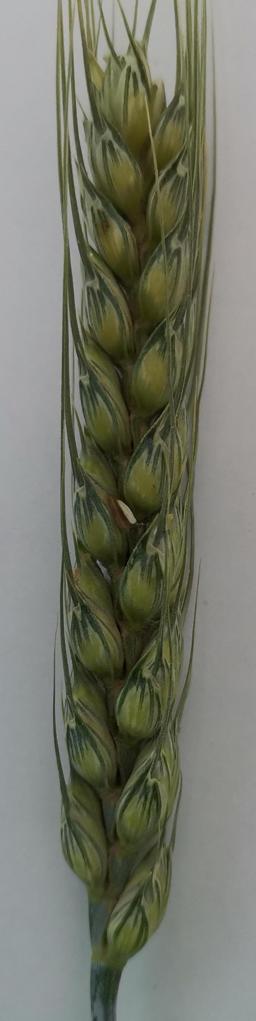

Supplement: Supplementary file 1 [file Data_Sheet_1.ZIP › 2. Datasets/1. training dataset for model training/Liangxing 99/3188.jpg]

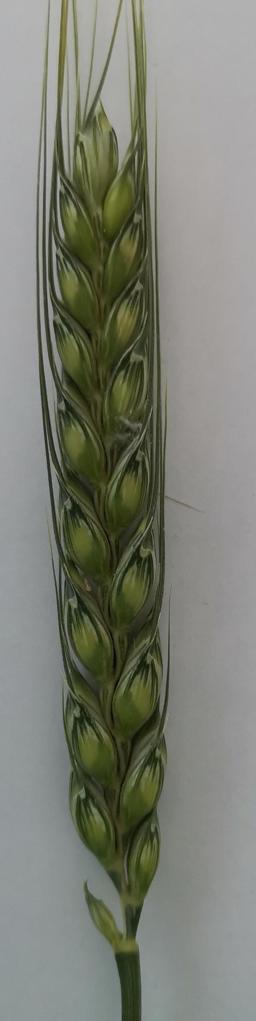

Supplement: Supplementary file 1 [file Data_Sheet_1.ZIP › 2. Datasets/1. training dataset for model training/Liangxing 99/3189.jpg]

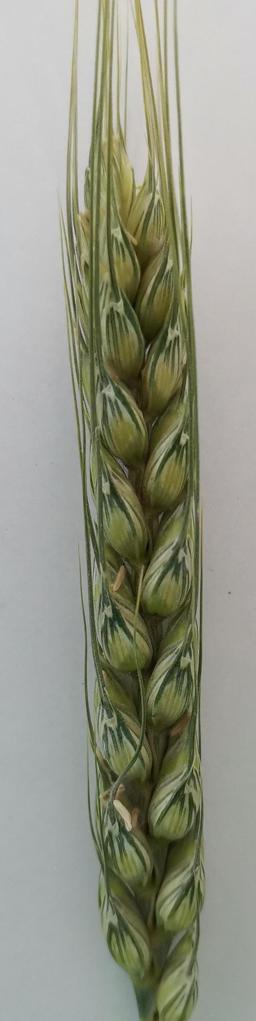

Supplement: Supplementary file 1 [file Data_Sheet_1.ZIP › 2. Datasets/1. training dataset for model training/Liangxing 99/3190.jpg]

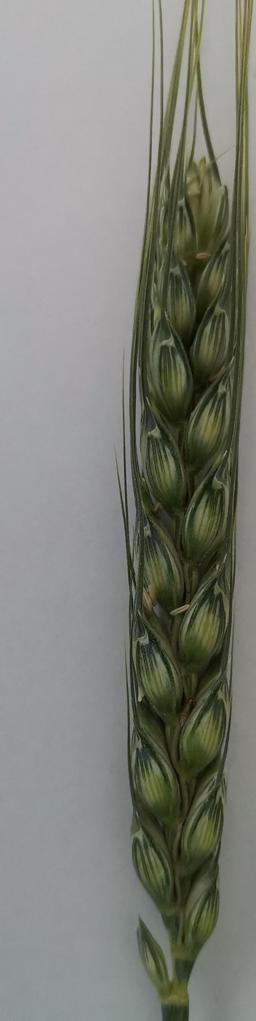

Supplement: Supplementary file 1 [file Data_Sheet_1.ZIP › 2. Datasets/1. training dataset for model training/Liangxing 99/3193.jpg]

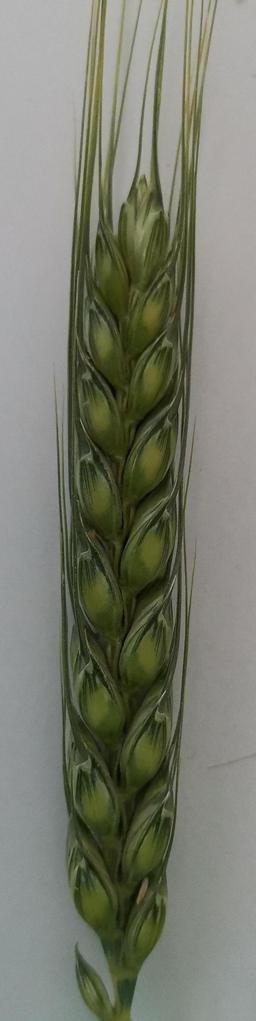

Supplement: Supplementary file 1 [file Data_Sheet_1.ZIP › 2. Datasets/1. training dataset for model training/Liangxing 99/3195.jpg]

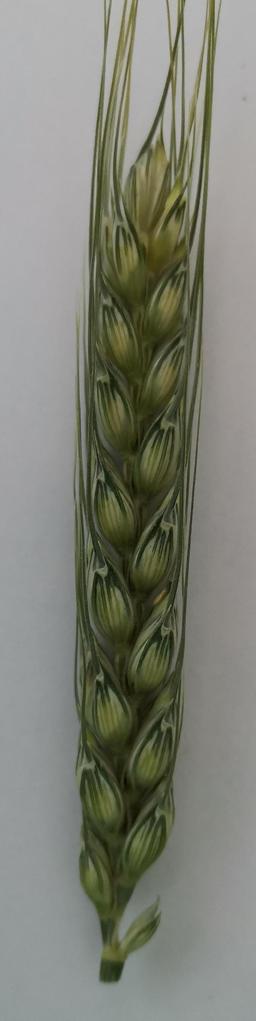

Supplement: Supplementary file 1 [file Data_Sheet_1.ZIP › 2. Datasets/1. training dataset for model training/Liangxing 99/3204.jpg]

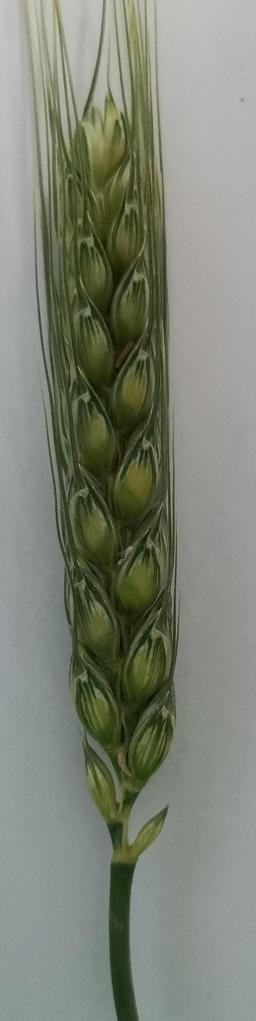

Supplement: Supplementary file 1 [file Data_Sheet_1.ZIP › 2. Datasets/1. training dataset for model training/Liangxing 99/3205.jpg]

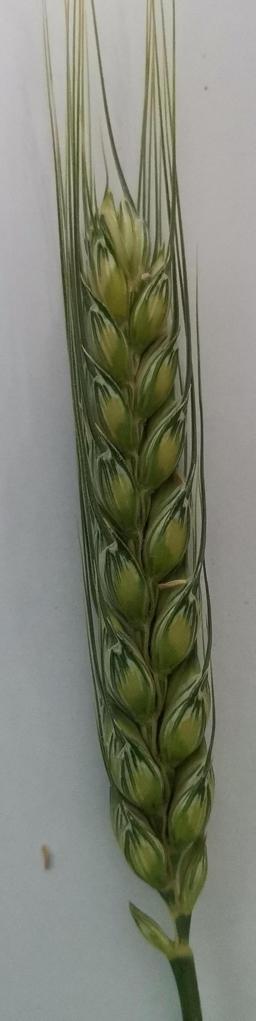

Supplement: Supplementary file 1 [file Data_Sheet_1.ZIP › 2. Datasets/1. training dataset for model training/Liangxing 99/3206.jpg]

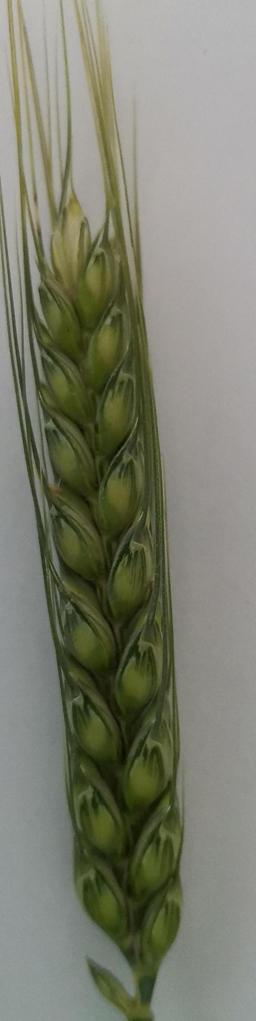

Supplement: Supplementary file 1 [file Data_Sheet_1.ZIP › 2. Datasets/1. training dataset for model training/Liangxing 99/3209.jpg]

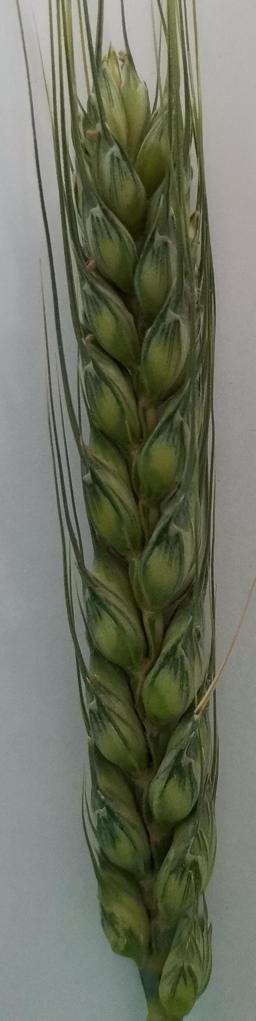

Supplement: Supplementary file 1 [file Data_Sheet_1.ZIP › 2. Datasets/1. training dataset for model training/Liangxing 99/3217.jpg]

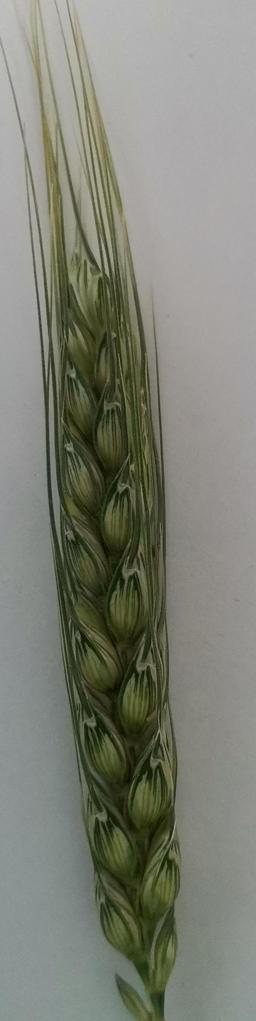

Supplement: Supplementary file 1 [file Data_Sheet_1.ZIP › 2. Datasets/1. training dataset for model training/Liangxing 99/3219.jpg]

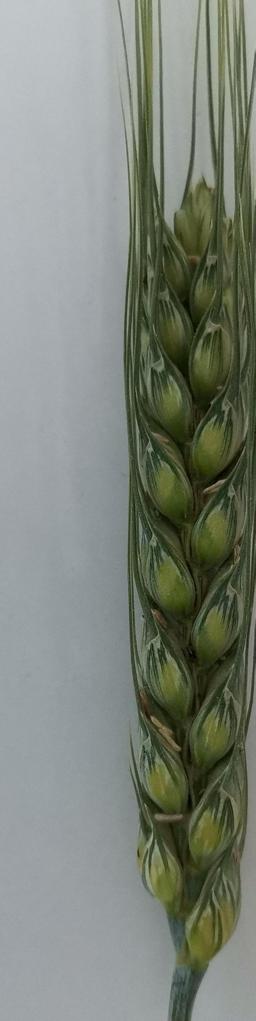

Supplement: Supplementary file 1 [file Data_Sheet_1.ZIP › 2. Datasets/1. training dataset for model training/Liangxing 99/3223.jpg]

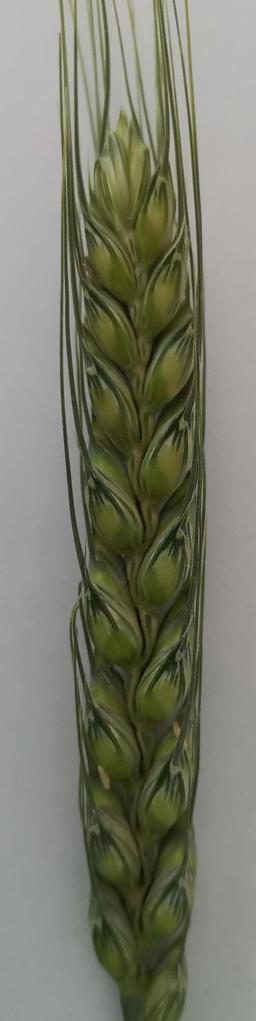

Supplement: Supplementary file 1 [file Data_Sheet_1.ZIP › 2. Datasets/1. training dataset for model training/Liangxing 99/3225.jpg]

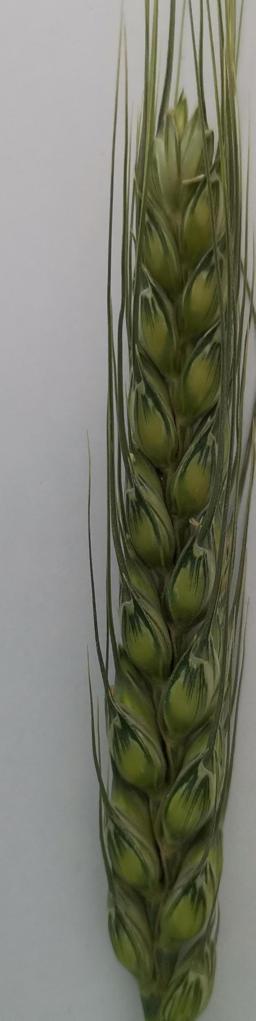

Supplement: Supplementary file 1 [file Data_Sheet_1.ZIP › 2. Datasets/1. training dataset for model training/Liangxing 99/3227.jpg]

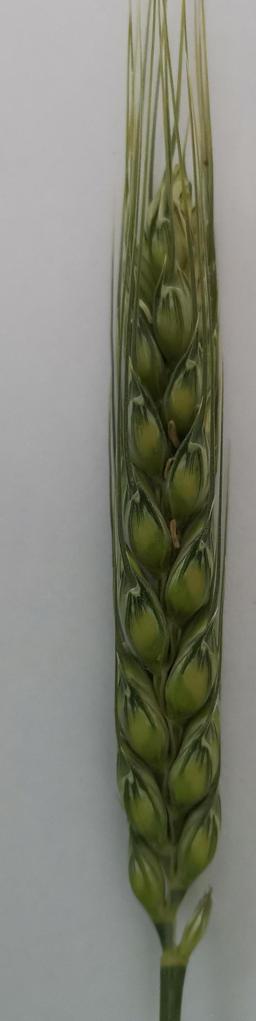

Supplement: Supplementary file 1 [file Data_Sheet_1.ZIP › 2. Datasets/1. training dataset for model training/Liangxing 99/3230.jpg]

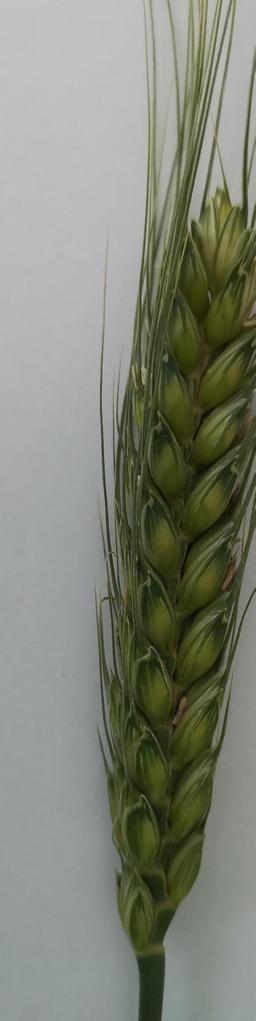

Supplement: Supplementary file 1 [file Data_Sheet_1.ZIP › 2. Datasets/1. training dataset for model training/Liangxing 99/3233.jpg]

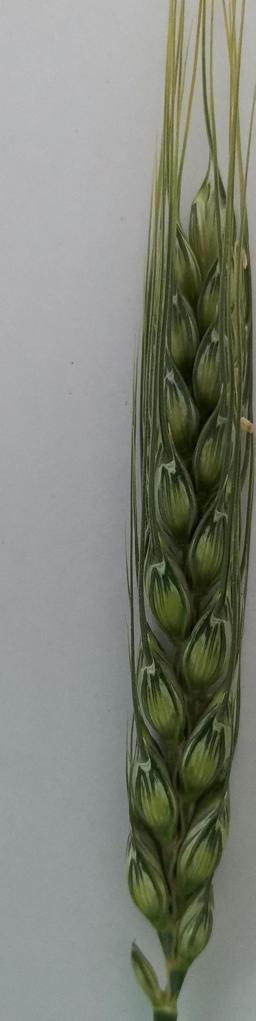

Supplement: Supplementary file 1 [file Data_Sheet_1.ZIP › 2. Datasets/1. training dataset for model training/Liangxing 99/3235.jpg]

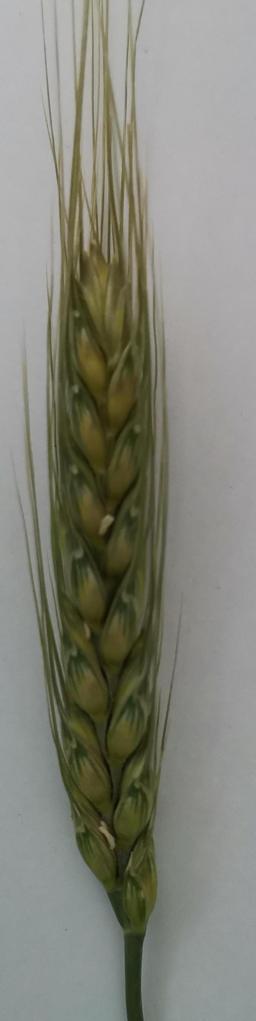

Supplement: Supplementary file 1 [file Data_Sheet_1.ZIP › 2. Datasets/1. training dataset for model training/Liangxing 99/3238.jpg]

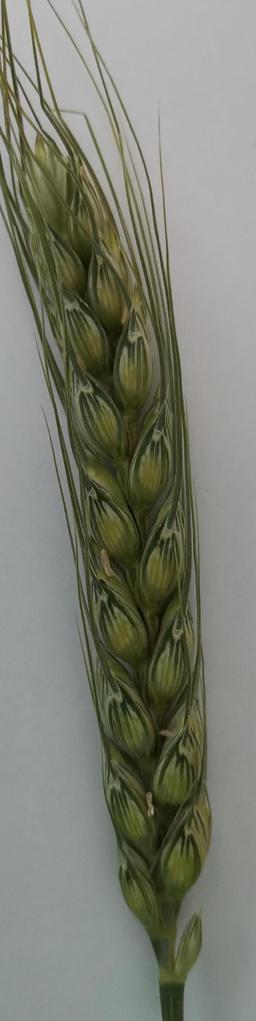

Supplement: Supplementary file 1 [file Data_Sheet_1.ZIP › 2. Datasets/1. training dataset for model training/Liangxing 99/3241.jpg]

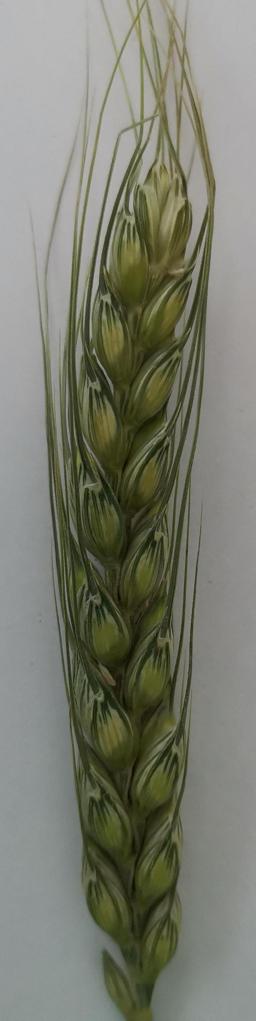

Supplement: Supplementary file 1 [file Data_Sheet_1.ZIP › 2. Datasets/1. training dataset for model training/Liangxing 99/3242.jpg]

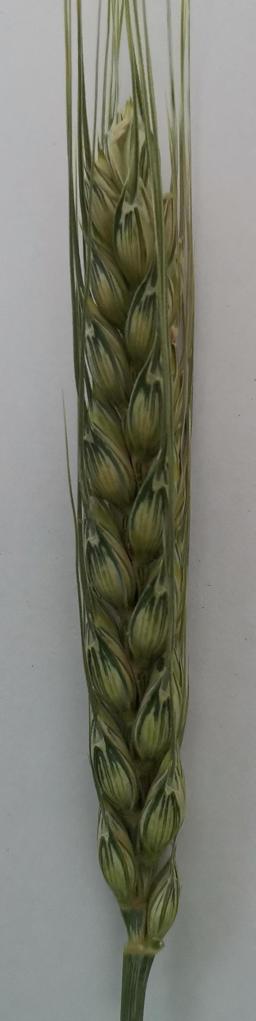

Supplement: Supplementary file 1 [file Data_Sheet_1.ZIP › 2. Datasets/1. training dataset for model training/Liangxing 99/3247.jpg]

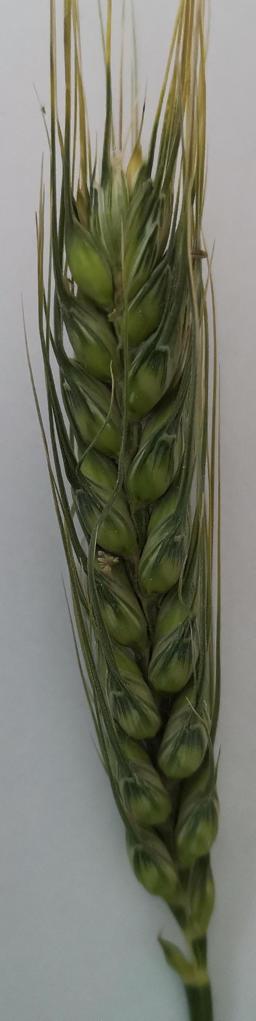

Supplement: Supplementary file 1 [file Data_Sheet_1.ZIP › 2. Datasets/1. training dataset for model training/Liangxing 99/3252.jpg]

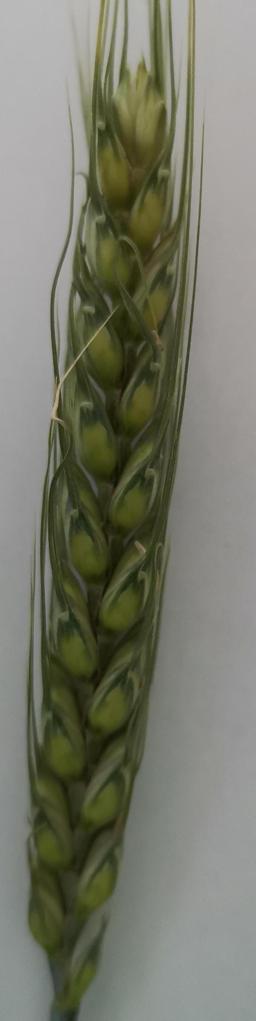

Supplement: Supplementary file 1 [file Data_Sheet_1.ZIP › 2. Datasets/1. training dataset for model training/Liangxing 99/3253.jpg]

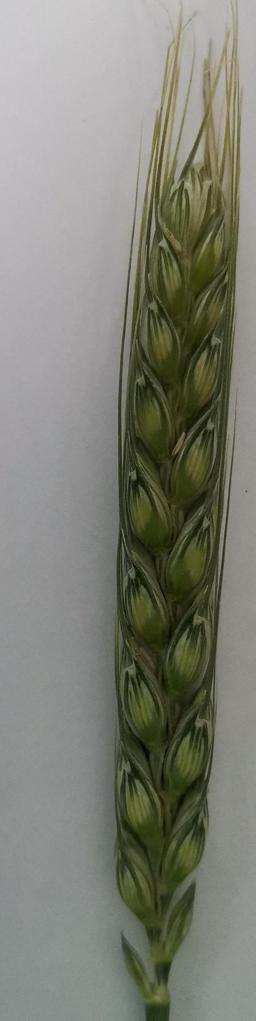

Supplement: Supplementary file 1 [file Data_Sheet_1.ZIP › 2. Datasets/1. training dataset for model training/Liangxing 99/3262.jpg]

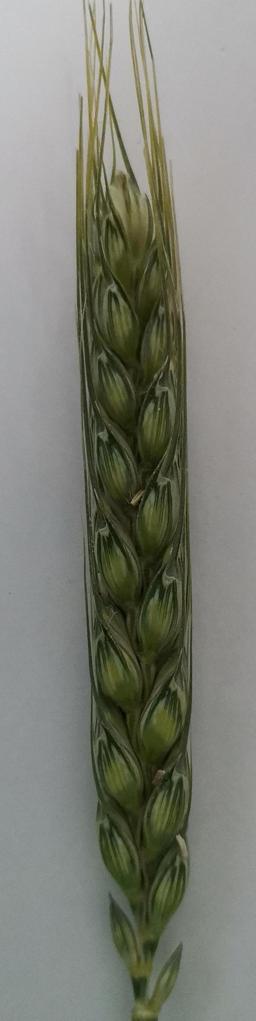

Supplement: Supplementary file 1 [file Data_Sheet_1.ZIP › 2. Datasets/1. training dataset for model training/Liangxing 99/3263.jpg]

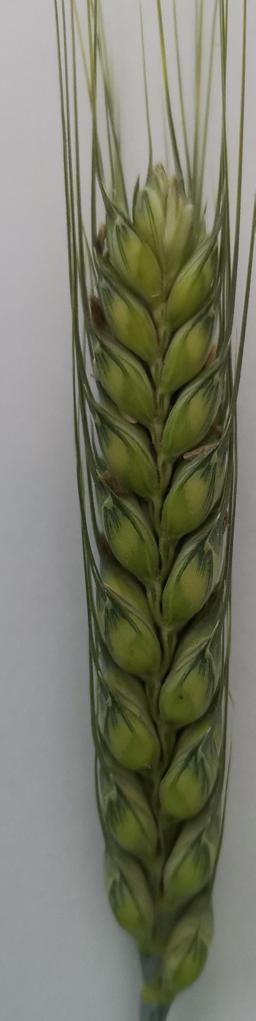

Supplement: Supplementary file 1 [file Data_Sheet_1.ZIP › 2. Datasets/1. training dataset for model training/Liangxing 99/3264.jpg]

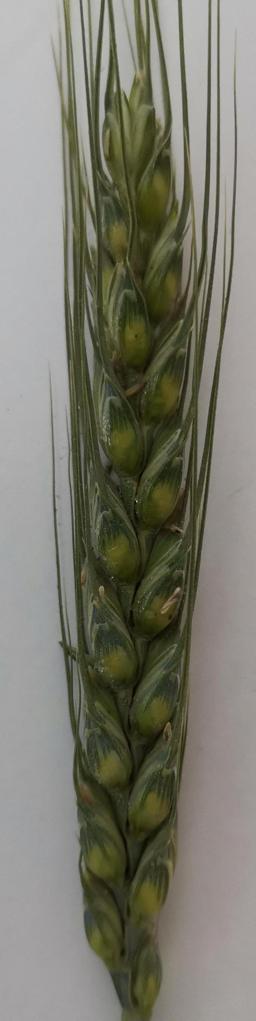

Supplement: Supplementary file 1 [file Data_Sheet_1.ZIP › 2. Datasets/1. training dataset for model training/Shannong 25/2003.jpg]

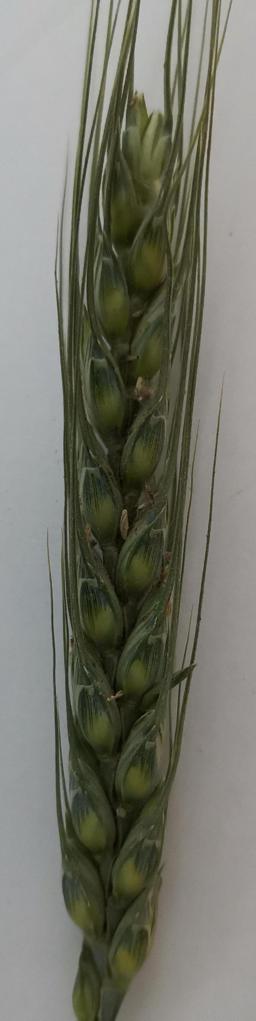

Supplement: Supplementary file 1 [file Data_Sheet_1.ZIP › 2. Datasets/1. training dataset for model training/Shannong 25/2004.jpg]

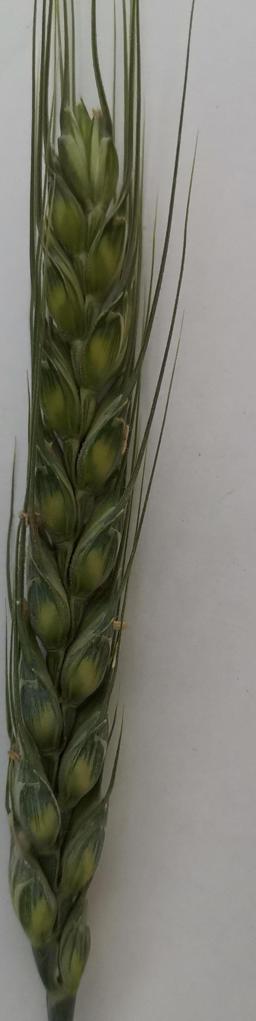

Supplement: Supplementary file 1 [file Data_Sheet_1.ZIP › 2. Datasets/1. training dataset for model training/Shannong 25/2006.jpg]

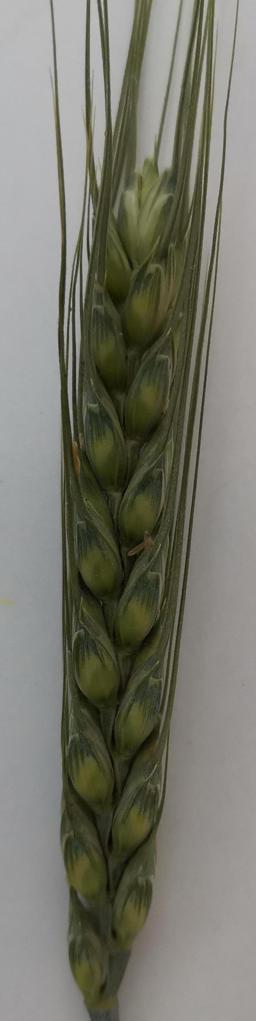

Supplement: Supplementary file 1 [file Data_Sheet_1.ZIP › 2. Datasets/1. training dataset for model training/Shannong 25/2012.jpg]

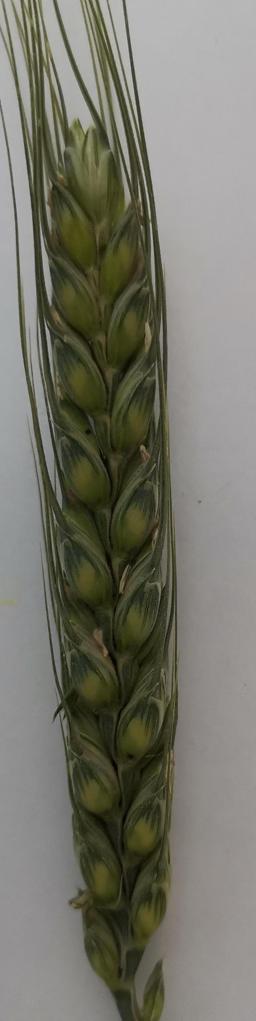

Supplement: Supplementary file 1 [file Data_Sheet_1.ZIP › 2. Datasets/1. training dataset for model training/Shannong 25/2014.jpg]

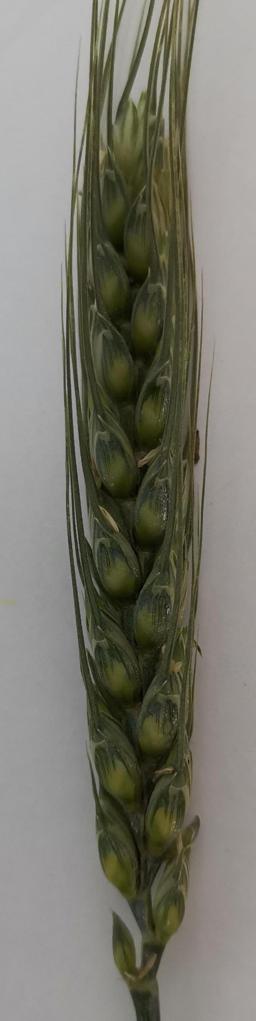

Supplement: Supplementary file 1 [file Data_Sheet_1.ZIP › 2. Datasets/1. training dataset for model training/Shannong 25/2015.jpg]

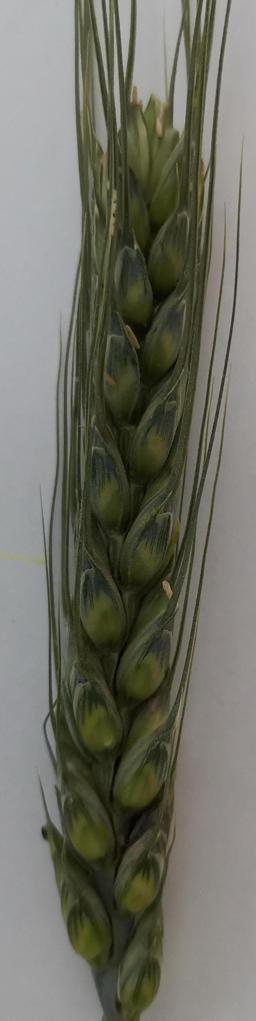

Supplement: Supplementary file 1 [file Data_Sheet_1.ZIP › 2. Datasets/1. training dataset for model training/Shannong 25/2016.jpg]

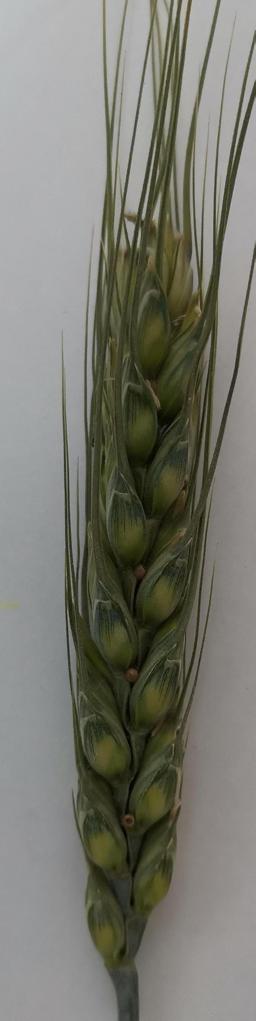

Supplement: Supplementary file 1 [file Data_Sheet_1.ZIP › 2. Datasets/1. training dataset for model training/Shannong 25/2019.jpg]

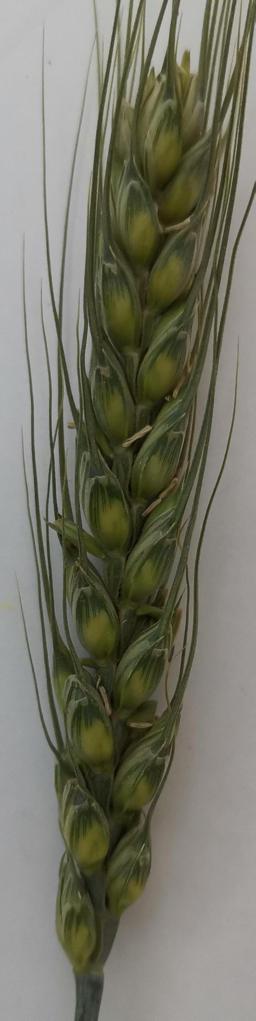

Supplement: Supplementary file 1 [file Data_Sheet_1.ZIP › 2. Datasets/1. training dataset for model training/Shannong 25/2021.jpg]

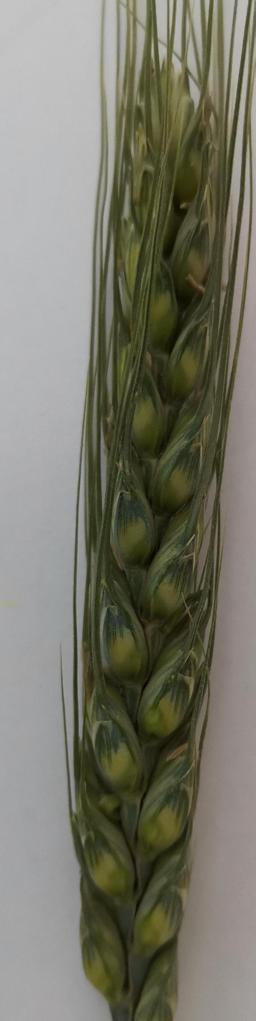

Supplement: Supplementary file 1 [file Data_Sheet_1.ZIP › 2. Datasets/1. training dataset for model training/Shannong 25/2023.jpg]

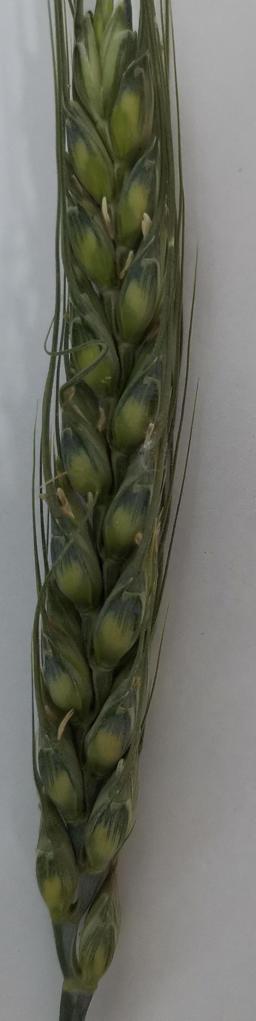

Supplement: Supplementary file 1 [file Data_Sheet_1.ZIP › 2. Datasets/1. training dataset for model training/Shannong 25/2029.jpg]

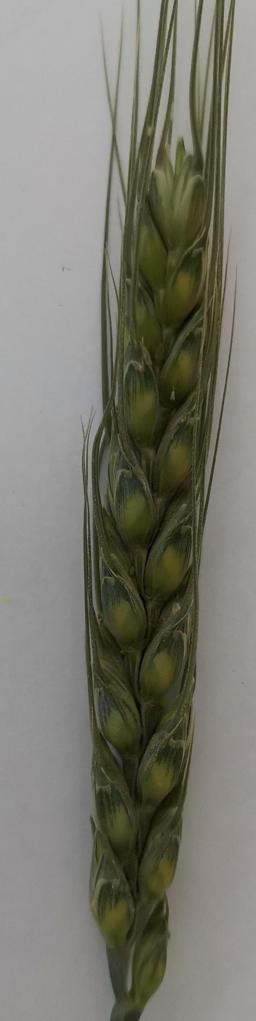

Supplement: Supplementary file 1 [file Data_Sheet_1.ZIP › 2. Datasets/1. training dataset for model training/Shannong 25/2032.jpg]

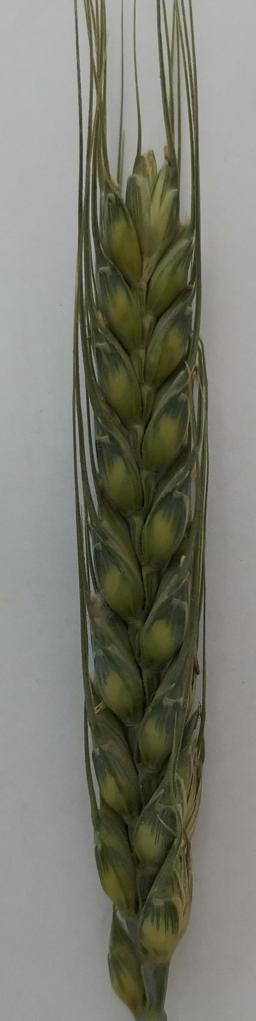

Supplement: Supplementary file 1 [file Data_Sheet_1.ZIP › 2. Datasets/1. training dataset for model training/Shannong 25/2034.jpg]

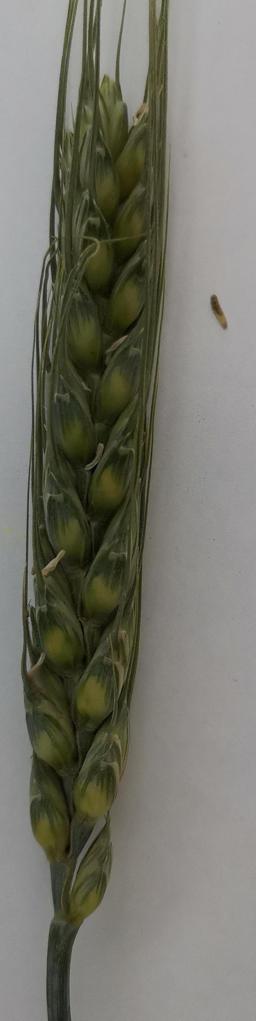

Supplement: Supplementary file 1 [file Data_Sheet_1.ZIP › 2. Datasets/1. training dataset for model training/Shannong 25/2036.jpg]

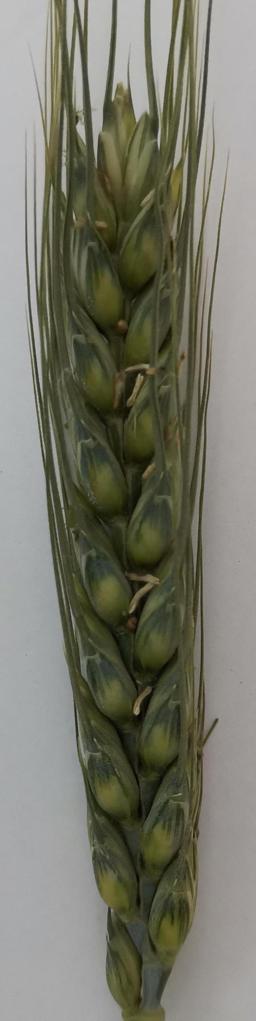

Supplement: Supplementary file 1 [file Data_Sheet_1.ZIP › 2. Datasets/1. training dataset for model training/Shannong 25/2051.jpg]

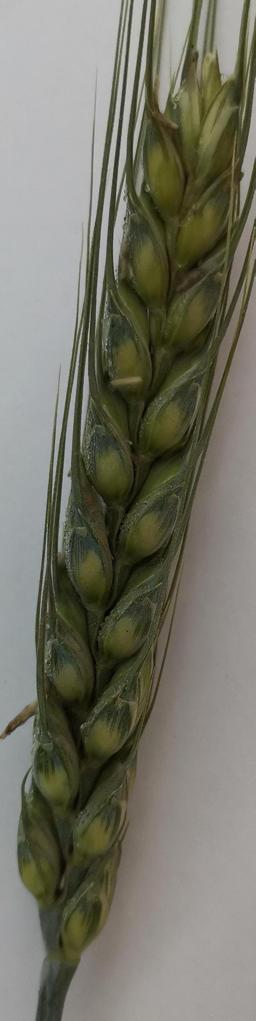

Supplement: Supplementary file 1 [file Data_Sheet_1.ZIP › 2. Datasets/1. training dataset for model training/Shannong 25/2054.jpg]

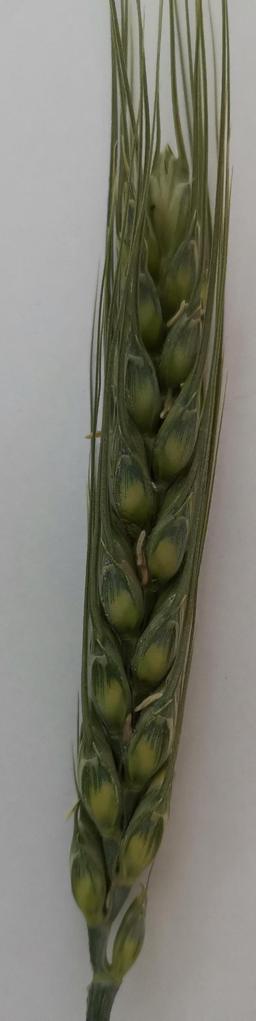

Supplement: Supplementary file 1 [file Data_Sheet_1.ZIP › 2. Datasets/1. training dataset for model training/Shannong 25/2065.jpg]

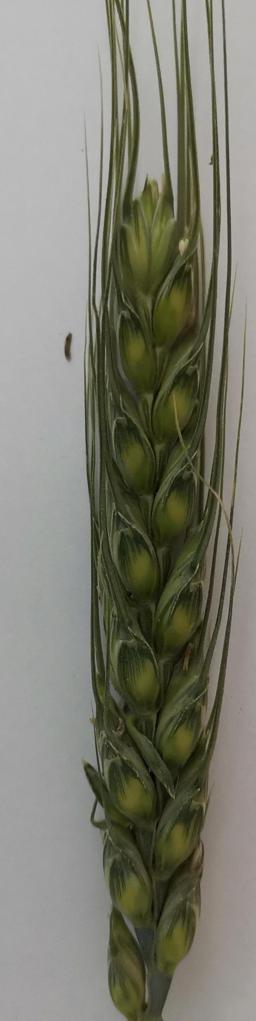

Supplement: Supplementary file 1 [file Data_Sheet_1.ZIP › 2. Datasets/1. training dataset for model training/Shannong 25/2068.jpg]

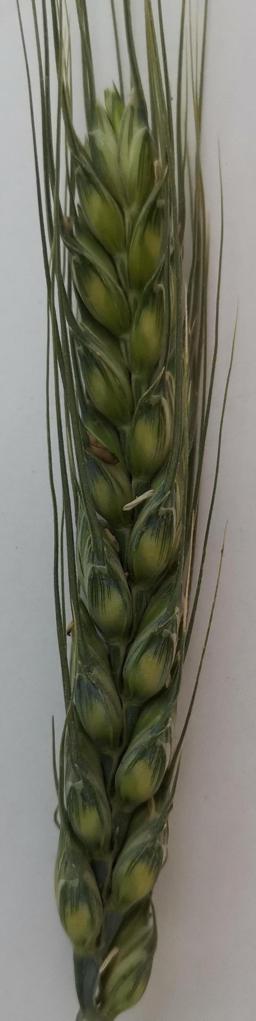

Supplement: Supplementary file 1 [file Data_Sheet_1.ZIP › 2. Datasets/1. training dataset for model training/Shannong 25/2070.jpg]

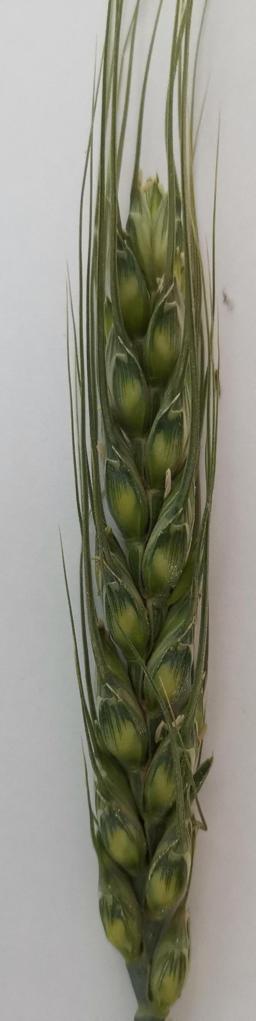

Supplement: Supplementary file 1 [file Data_Sheet_1.ZIP › 2. Datasets/1. training dataset for model training/Shannong 25/2079.jpg]
