# Supplementary figures and images for: Automatic Detection and Counting of Wheat Spikelet Using Semi-Automatic Labeling and Deep Learning (part 4 of 8)
Source: Front Plant Sci. 2022 May 30;13:872555. doi: 10.3389/fpls.2022.872555 (PMC9189412; doi:10.3389/fpls.2022.872555)

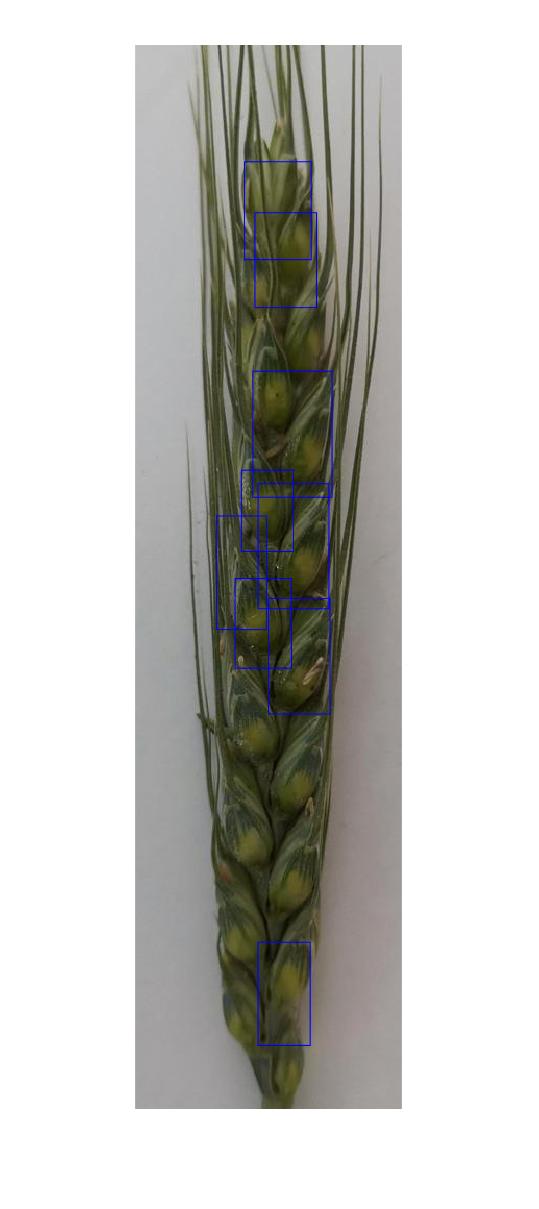

Supplement: Supplementary file 2 [file Data_Sheet_2.zip › 3. Labeling results of watershed algorithm (section Spikelet segmentation and annotation)/Shannong 25/2003b.jpg]

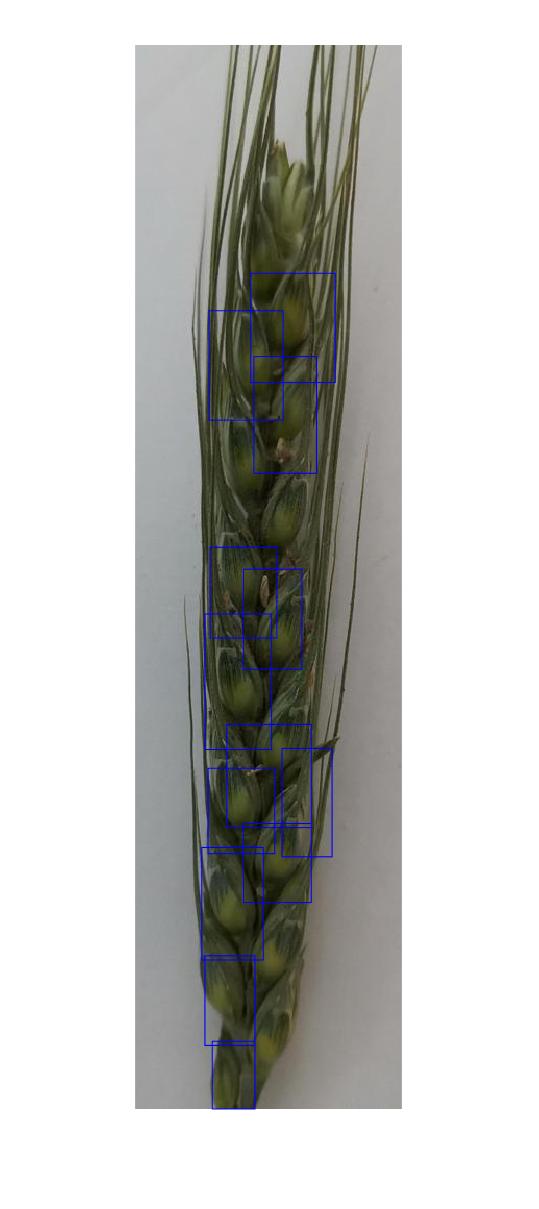

Supplement: Supplementary file 2 [file Data_Sheet_2.zip › 3. Labeling results of watershed algorithm (section Spikelet segmentation and annotation)/Shannong 25/2004b.jpg]

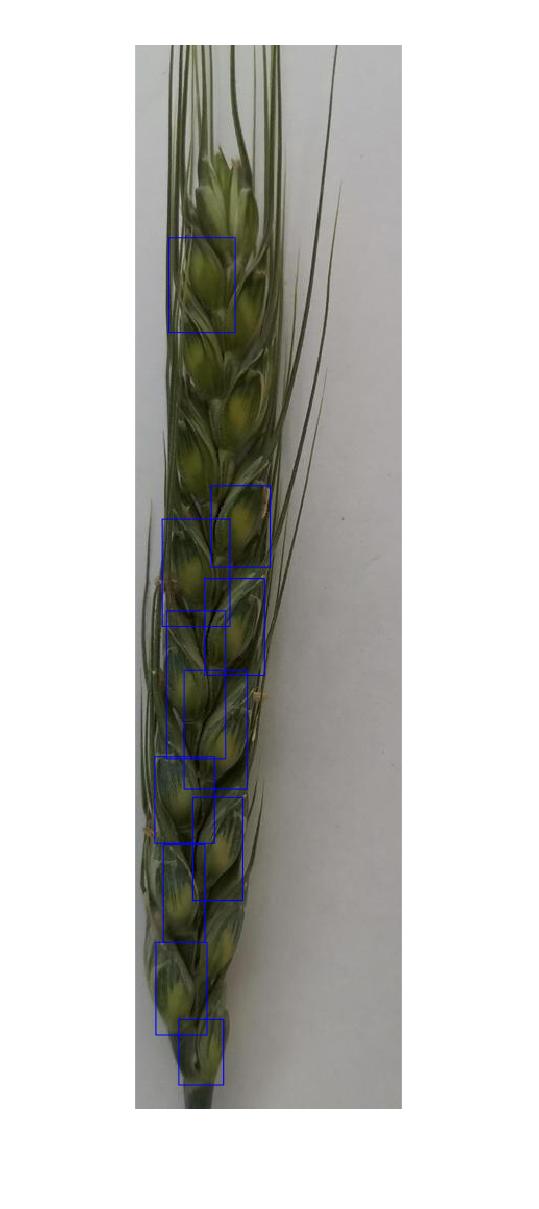

Supplement: Supplementary file 2 [file Data_Sheet_2.zip › 3. Labeling results of watershed algorithm (section Spikelet segmentation and annotation)/Shannong 25/2006b.jpg]

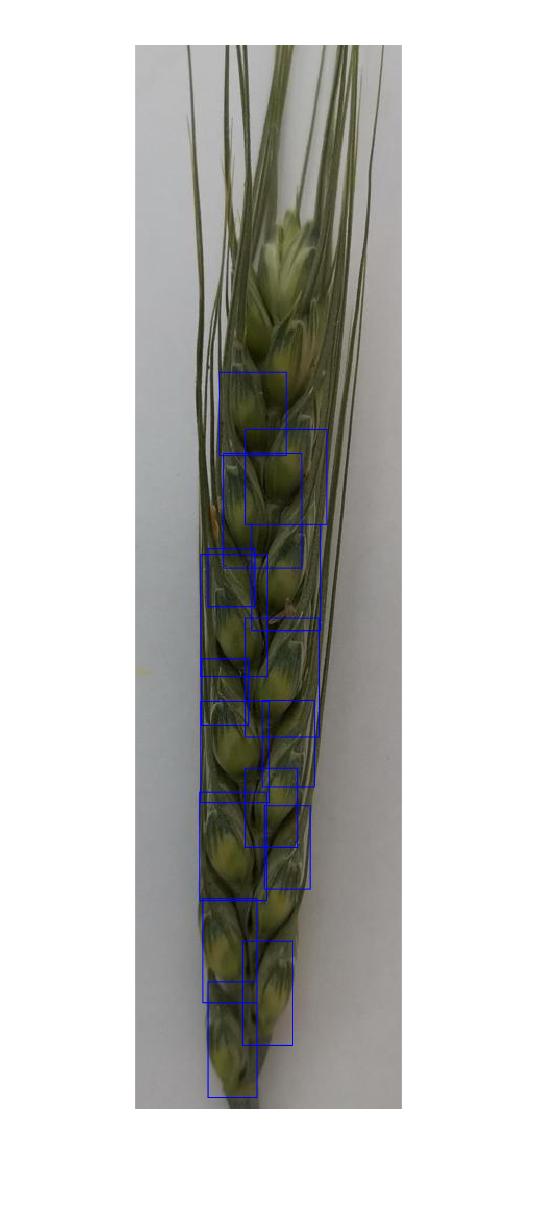

Supplement: Supplementary file 2 [file Data_Sheet_2.zip › 3. Labeling results of watershed algorithm (section Spikelet segmentation and annotation)/Shannong 25/2012b.jpg]

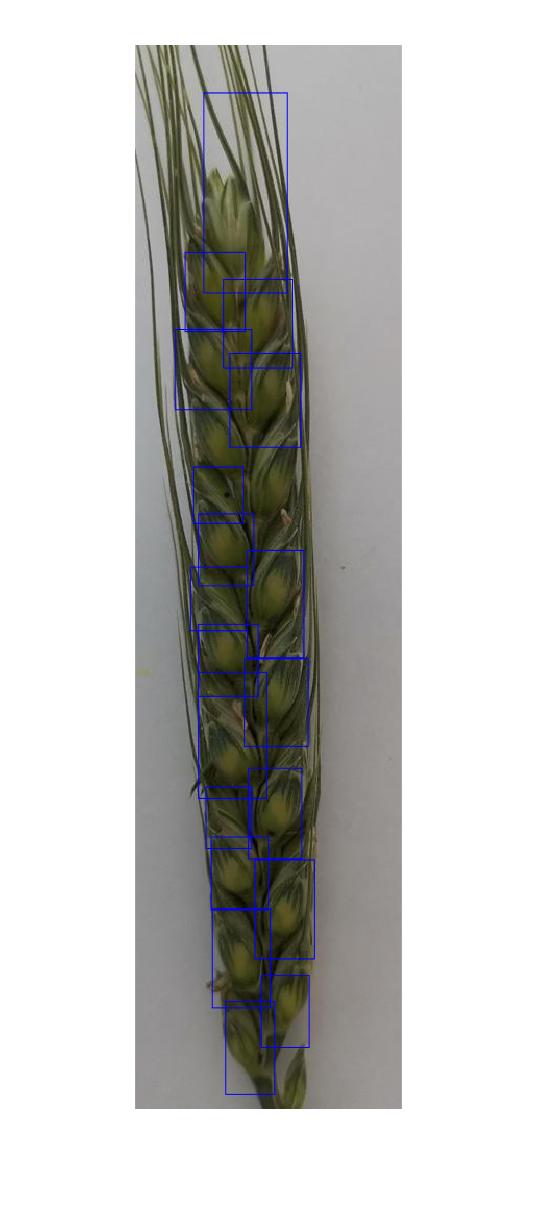

Supplement: Supplementary file 2 [file Data_Sheet_2.zip › 3. Labeling results of watershed algorithm (section Spikelet segmentation and annotation)/Shannong 25/2014b.jpg]

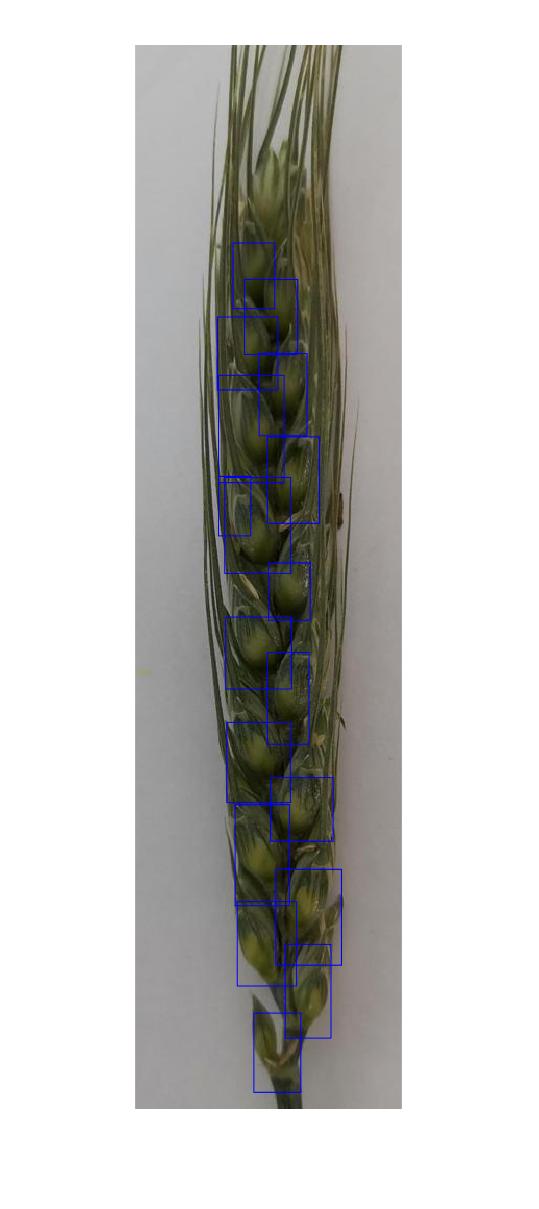

Supplement: Supplementary file 2 [file Data_Sheet_2.zip › 3. Labeling results of watershed algorithm (section Spikelet segmentation and annotation)/Shannong 25/2015b.jpg]

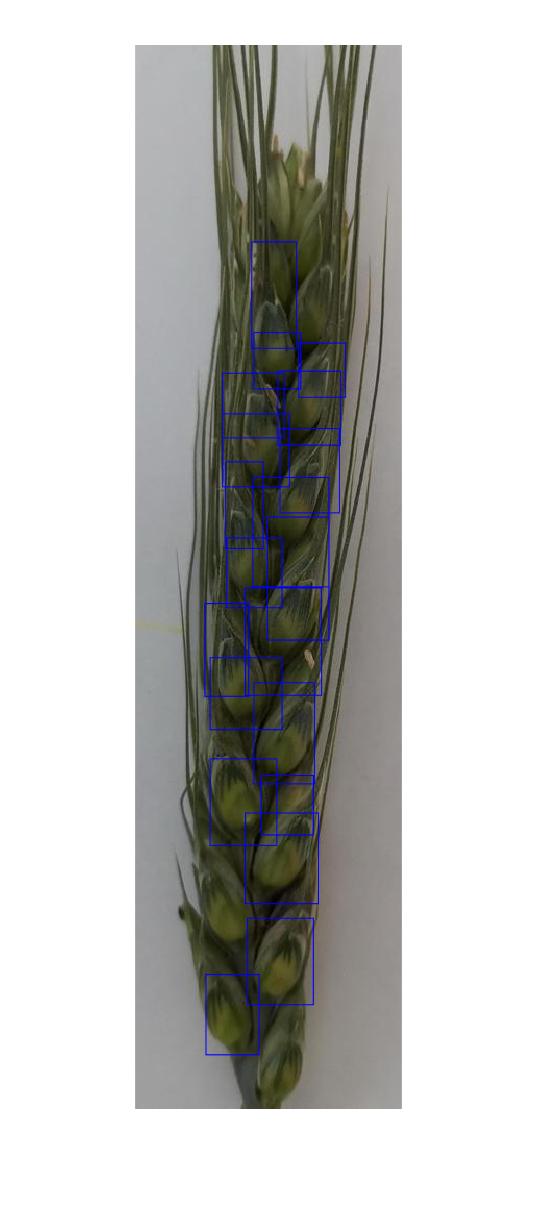

Supplement: Supplementary file 2 [file Data_Sheet_2.zip › 3. Labeling results of watershed algorithm (section Spikelet segmentation and annotation)/Shannong 25/2016b.jpg]

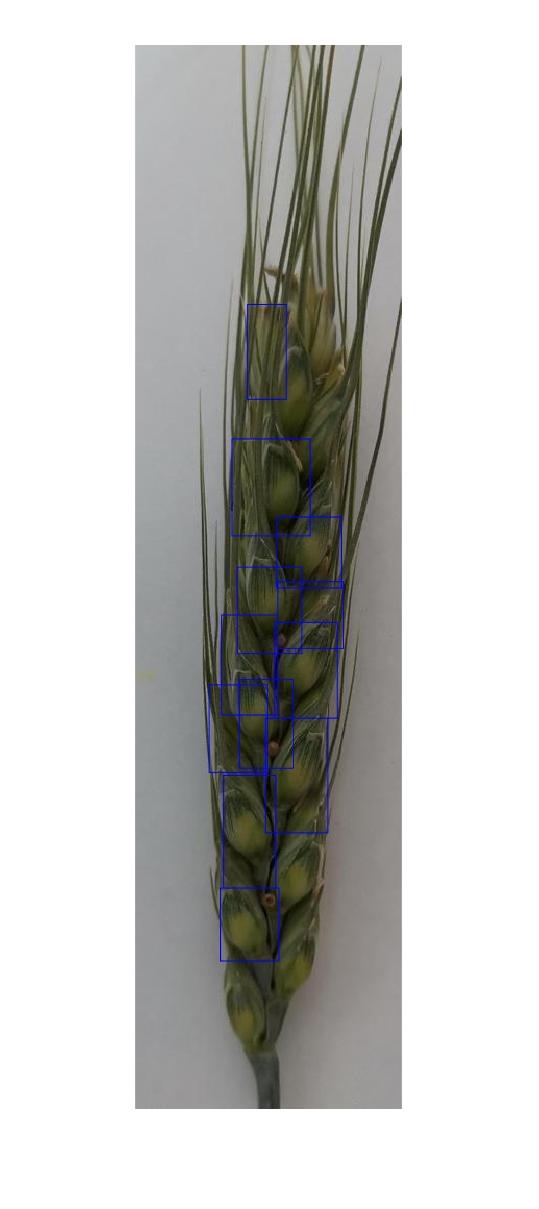

Supplement: Supplementary file 2 [file Data_Sheet_2.zip › 3. Labeling results of watershed algorithm (section Spikelet segmentation and annotation)/Shannong 25/2019b.jpg]

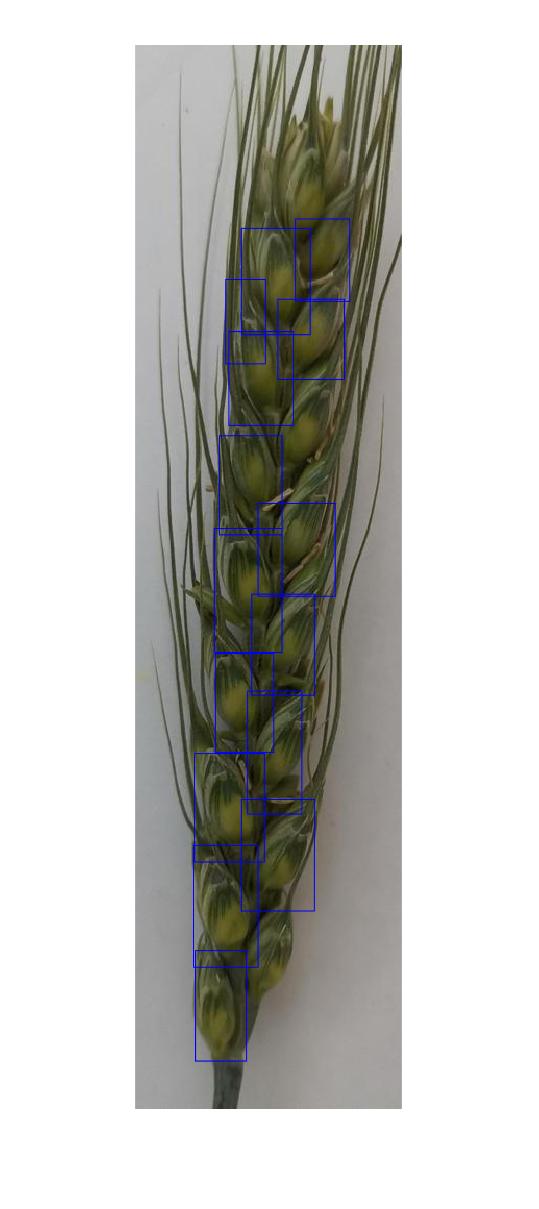

Supplement: Supplementary file 2 [file Data_Sheet_2.zip › 3. Labeling results of watershed algorithm (section Spikelet segmentation and annotation)/Shannong 25/2021b.jpg]

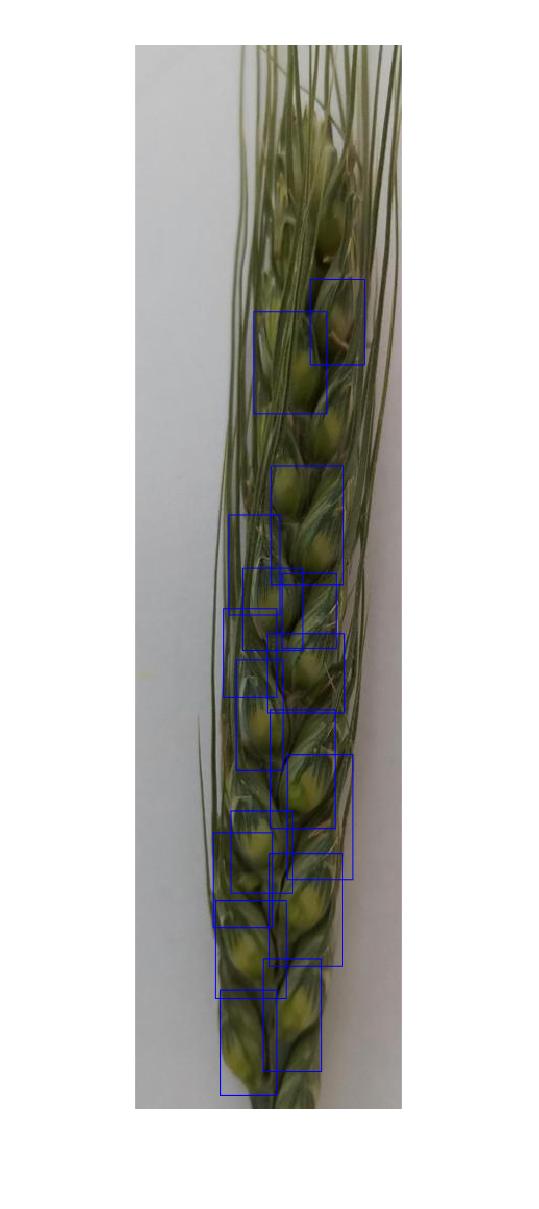

Supplement: Supplementary file 2 [file Data_Sheet_2.zip › 3. Labeling results of watershed algorithm (section Spikelet segmentation and annotation)/Shannong 25/2023b.jpg]

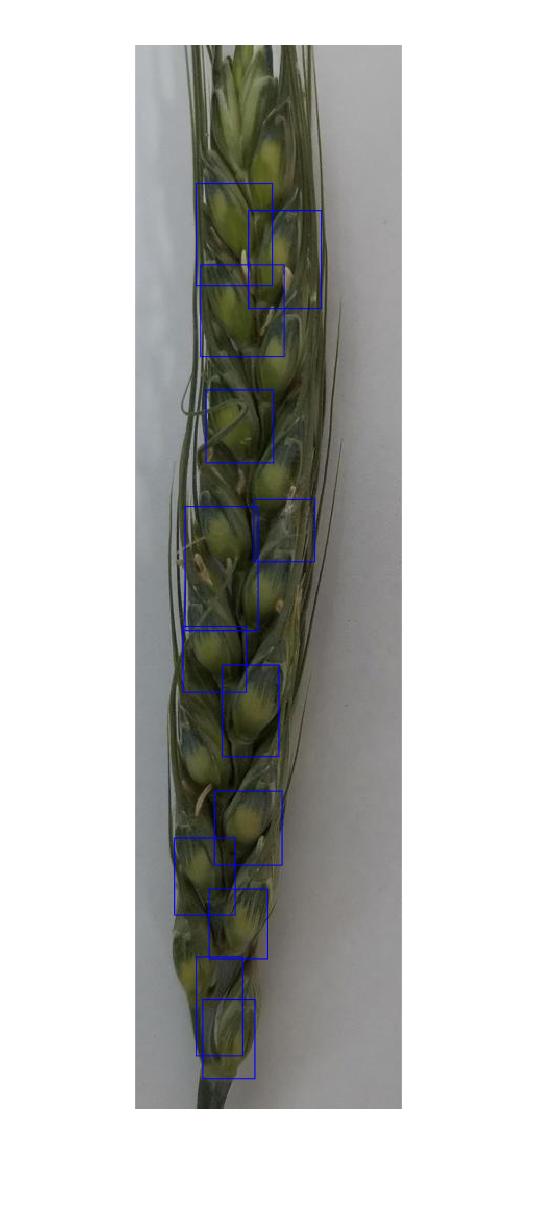

Supplement: Supplementary file 2 [file Data_Sheet_2.zip › 3. Labeling results of watershed algorithm (section Spikelet segmentation and annotation)/Shannong 25/2029b.jpg]

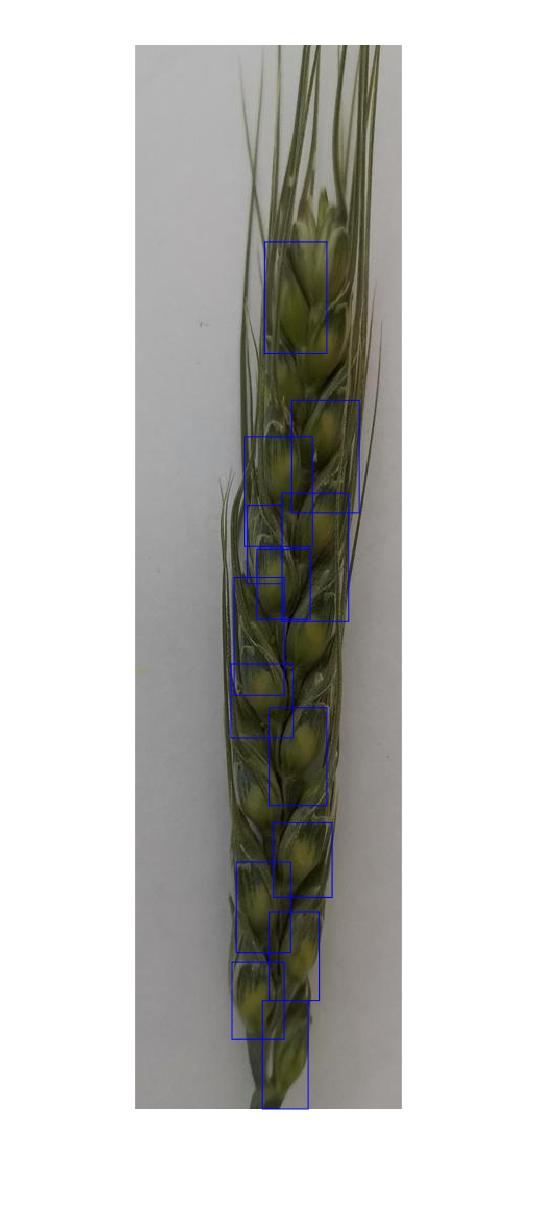

Supplement: Supplementary file 2 [file Data_Sheet_2.zip › 3. Labeling results of watershed algorithm (section Spikelet segmentation and annotation)/Shannong 25/2032b.jpg]

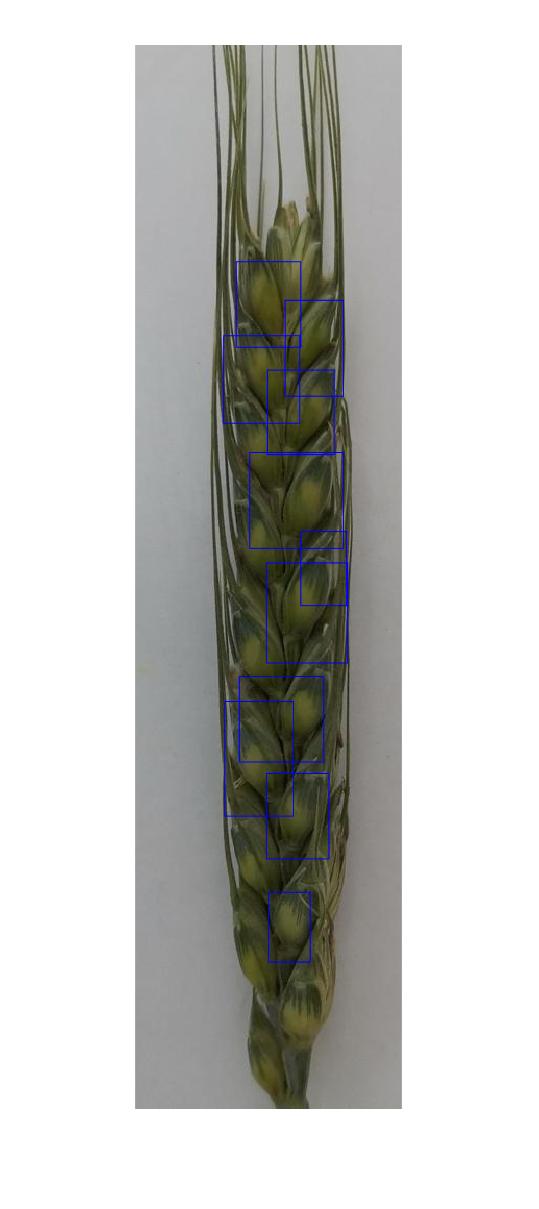

Supplement: Supplementary file 2 [file Data_Sheet_2.zip › 3. Labeling results of watershed algorithm (section Spikelet segmentation and annotation)/Shannong 25/2034b.jpg]

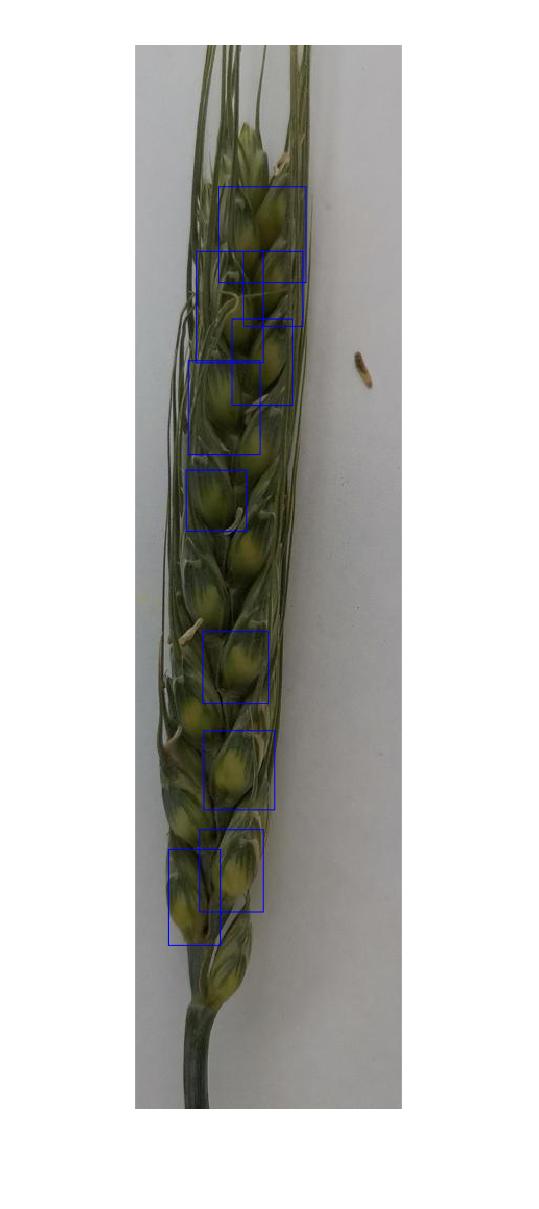

Supplement: Supplementary file 2 [file Data_Sheet_2.zip › 3. Labeling results of watershed algorithm (section Spikelet segmentation and annotation)/Shannong 25/2036b.jpg]

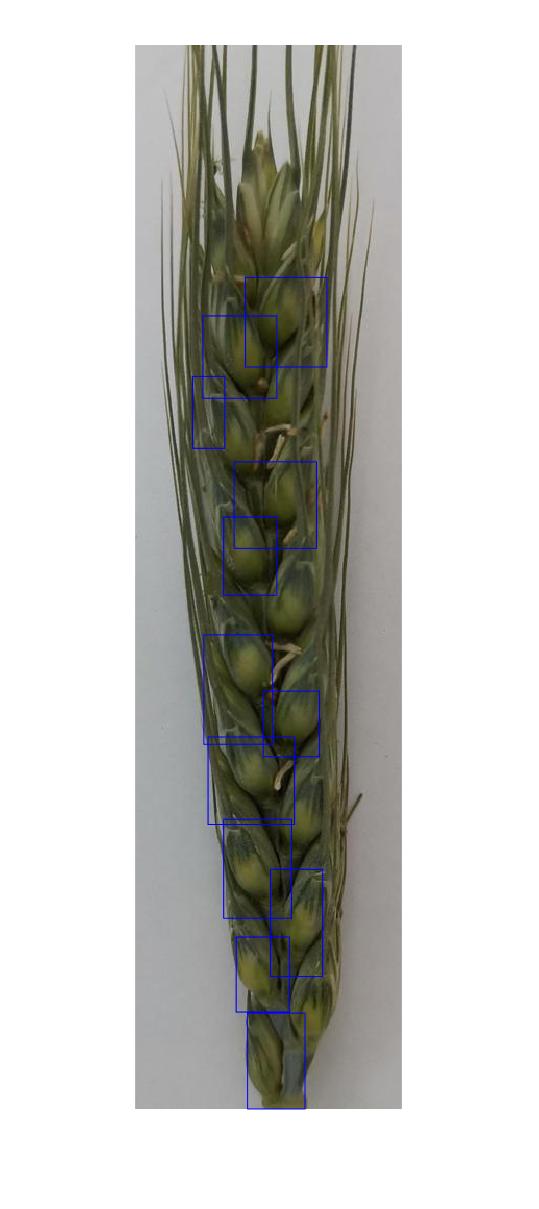

Supplement: Supplementary file 2 [file Data_Sheet_2.zip › 3. Labeling results of watershed algorithm (section Spikelet segmentation and annotation)/Shannong 25/2051b.jpg]

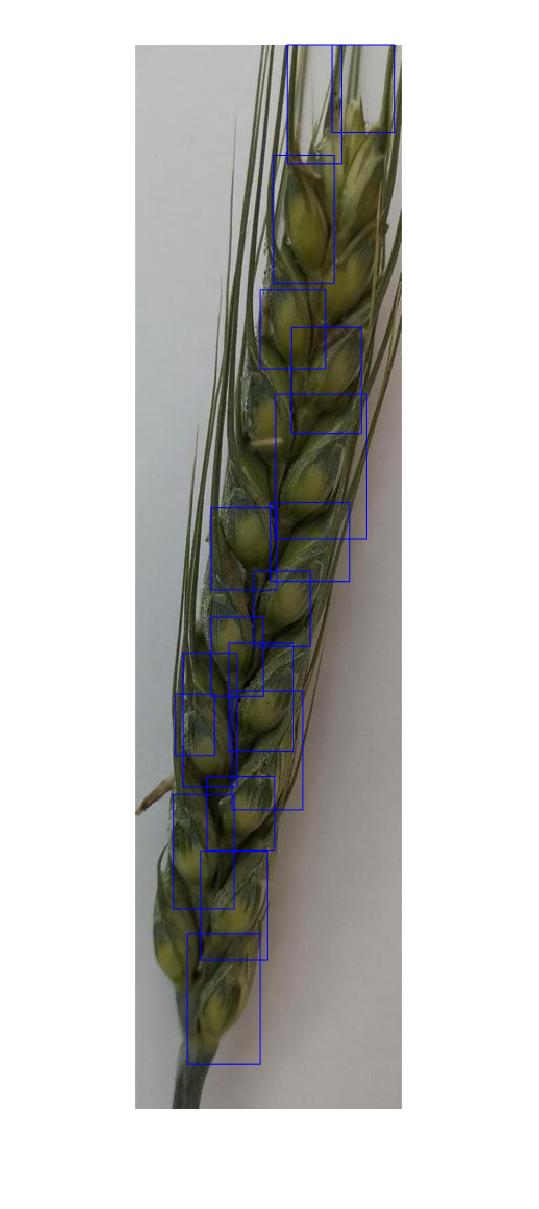

Supplement: Supplementary file 2 [file Data_Sheet_2.zip › 3. Labeling results of watershed algorithm (section Spikelet segmentation and annotation)/Shannong 25/2054b.jpg]

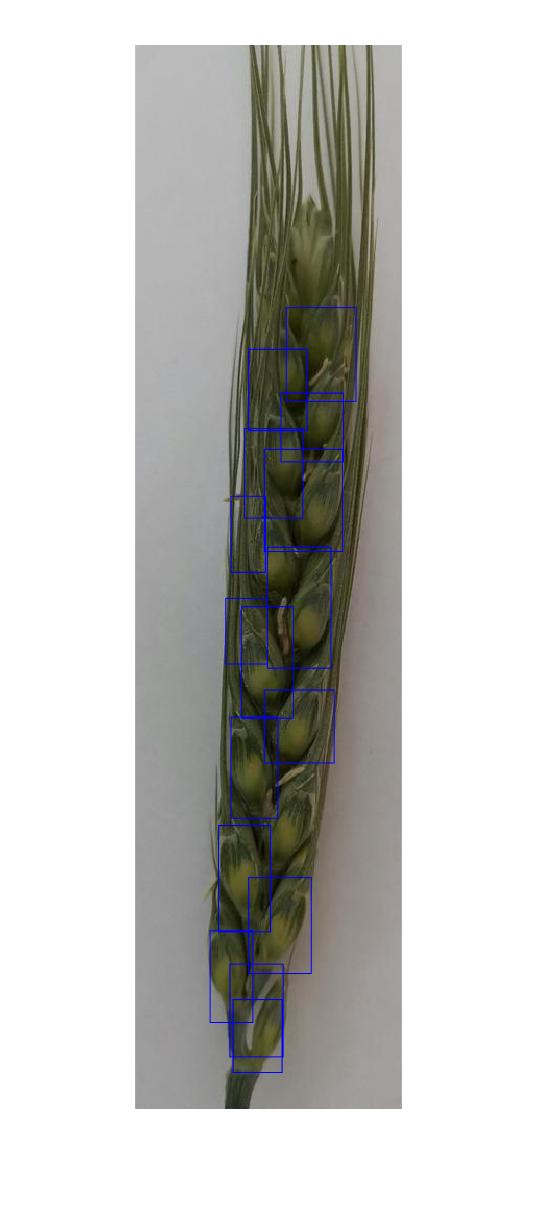

Supplement: Supplementary file 2 [file Data_Sheet_2.zip › 3. Labeling results of watershed algorithm (section Spikelet segmentation and annotation)/Shannong 25/2065b.jpg]

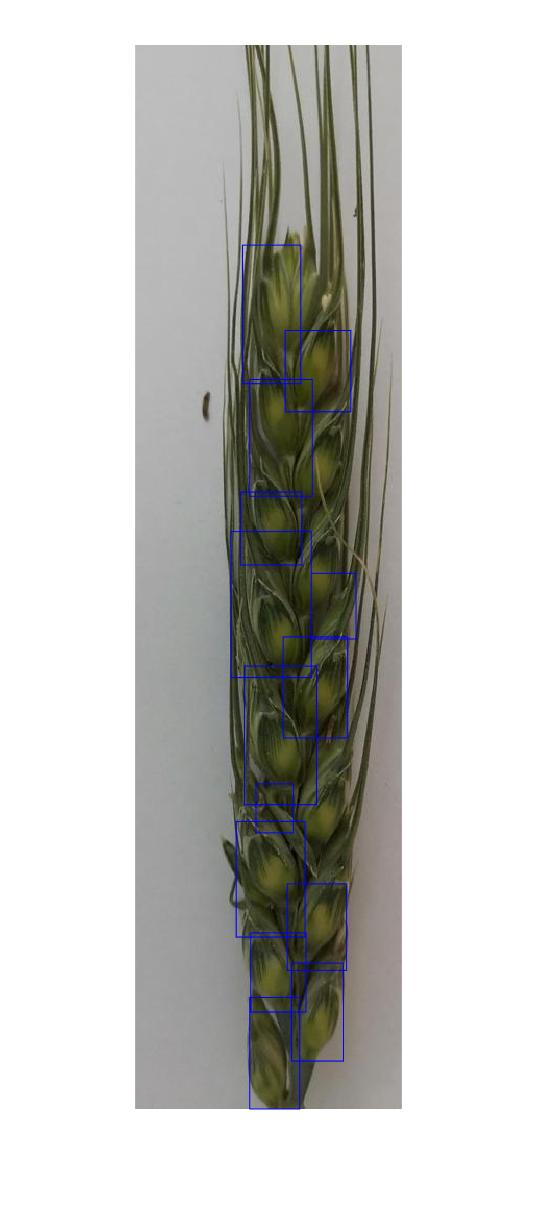

Supplement: Supplementary file 2 [file Data_Sheet_2.zip › 3. Labeling results of watershed algorithm (section Spikelet segmentation and annotation)/Shannong 25/2068b.jpg]

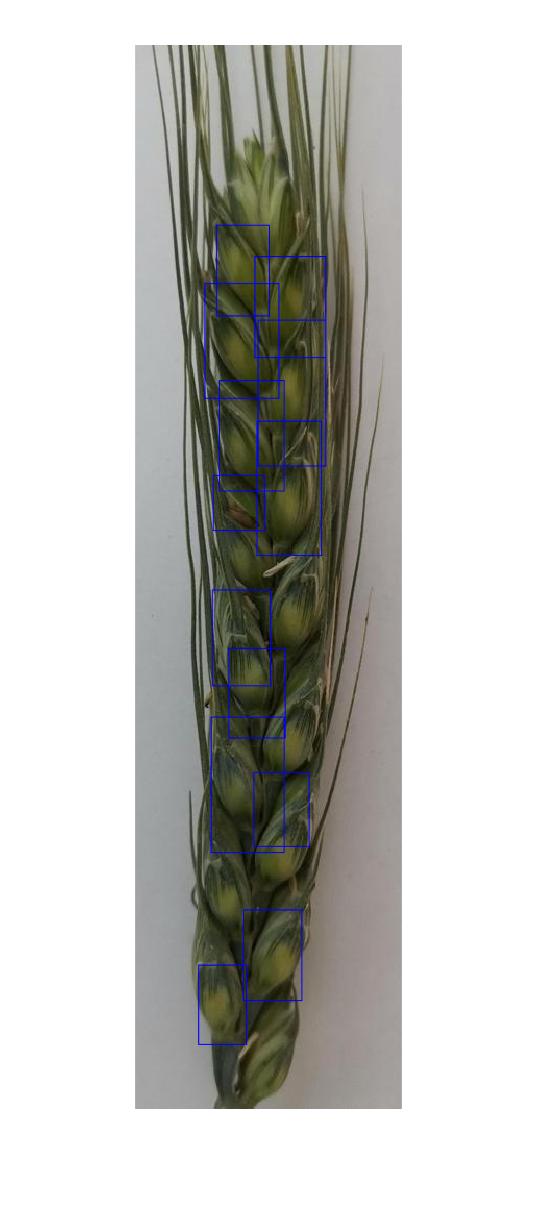

Supplement: Supplementary file 2 [file Data_Sheet_2.zip › 3. Labeling results of watershed algorithm (section Spikelet segmentation and annotation)/Shannong 25/2070b.jpg]

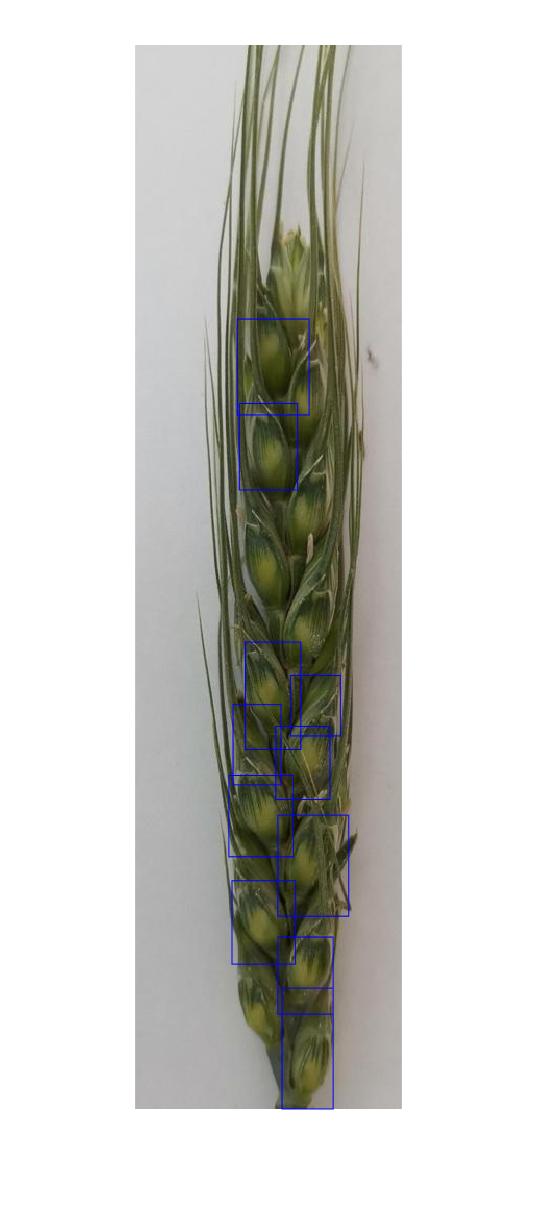

Supplement: Supplementary file 2 [file Data_Sheet_2.zip › 3. Labeling results of watershed algorithm (section Spikelet segmentation and annotation)/Shannong 25/2079b.jpg]

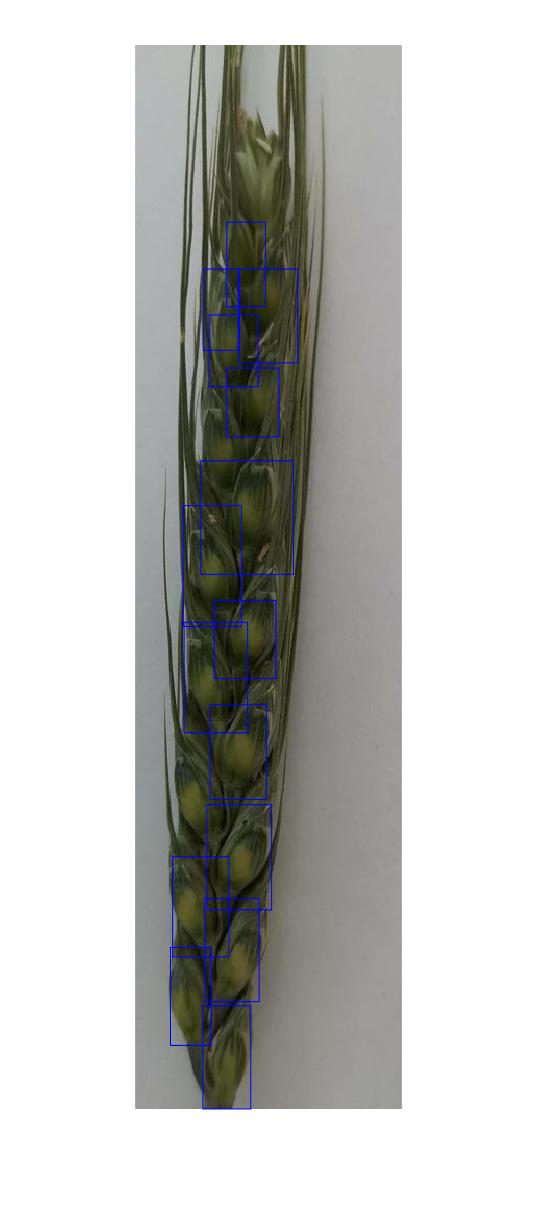

Supplement: Supplementary file 2 [file Data_Sheet_2.zip › 3. Labeling results of watershed algorithm (section Spikelet segmentation and annotation)/Shannong 25/2080b.jpg]

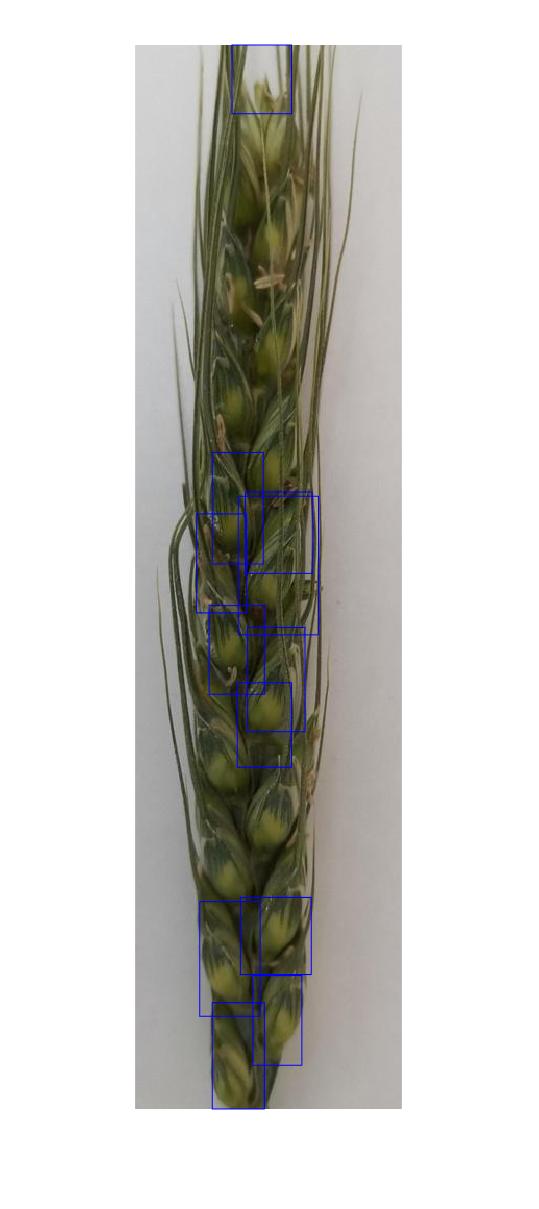

Supplement: Supplementary file 2 [file Data_Sheet_2.zip › 3. Labeling results of watershed algorithm (section Spikelet segmentation and annotation)/Shannong 25/2109b.jpg]

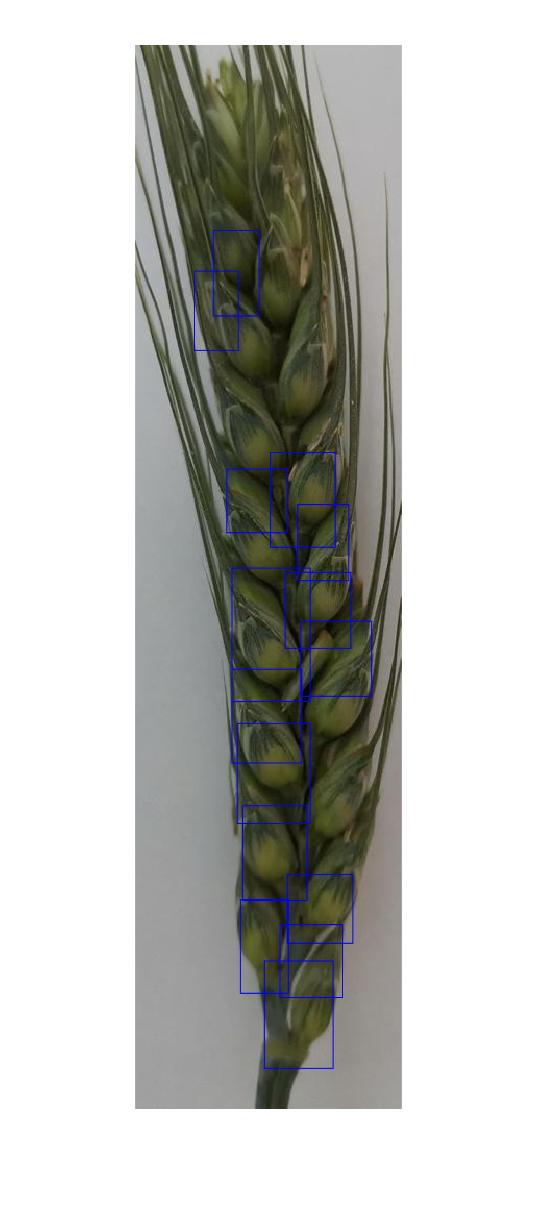

Supplement: Supplementary file 2 [file Data_Sheet_2.zip › 3. Labeling results of watershed algorithm (section Spikelet segmentation and annotation)/Shannong 25/2111b.jpg]

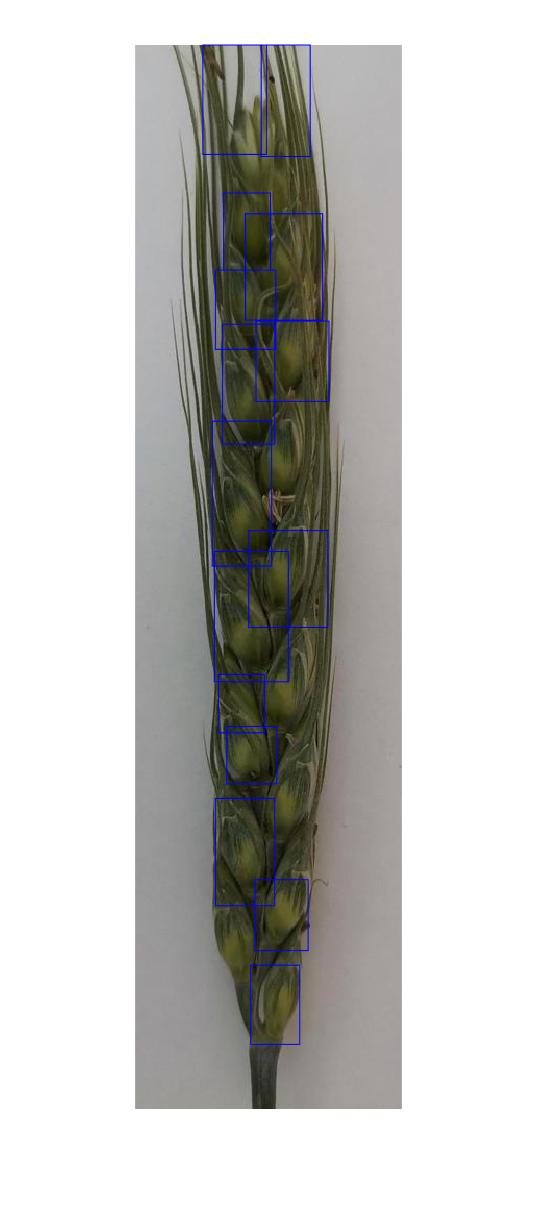

Supplement: Supplementary file 2 [file Data_Sheet_2.zip › 3. Labeling results of watershed algorithm (section Spikelet segmentation and annotation)/Shannong 25/2112b.jpg]

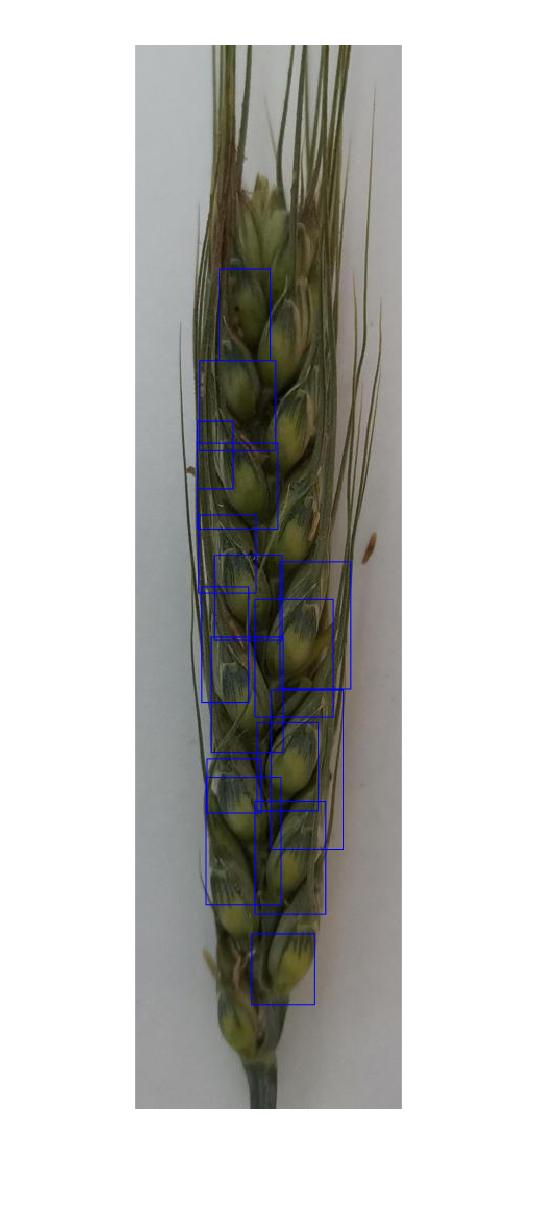

Supplement: Supplementary file 2 [file Data_Sheet_2.zip › 3. Labeling results of watershed algorithm (section Spikelet segmentation and annotation)/Shannong 25/2121b.jpg]

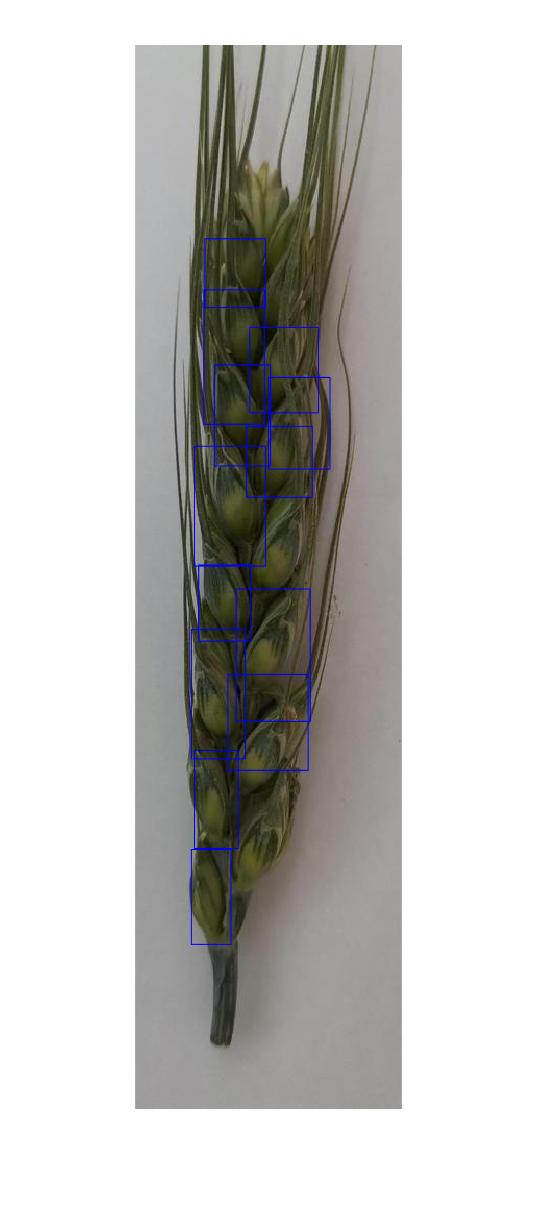

Supplement: Supplementary file 2 [file Data_Sheet_2.zip › 3. Labeling results of watershed algorithm (section Spikelet segmentation and annotation)/Shannong 25/2124b.jpg]

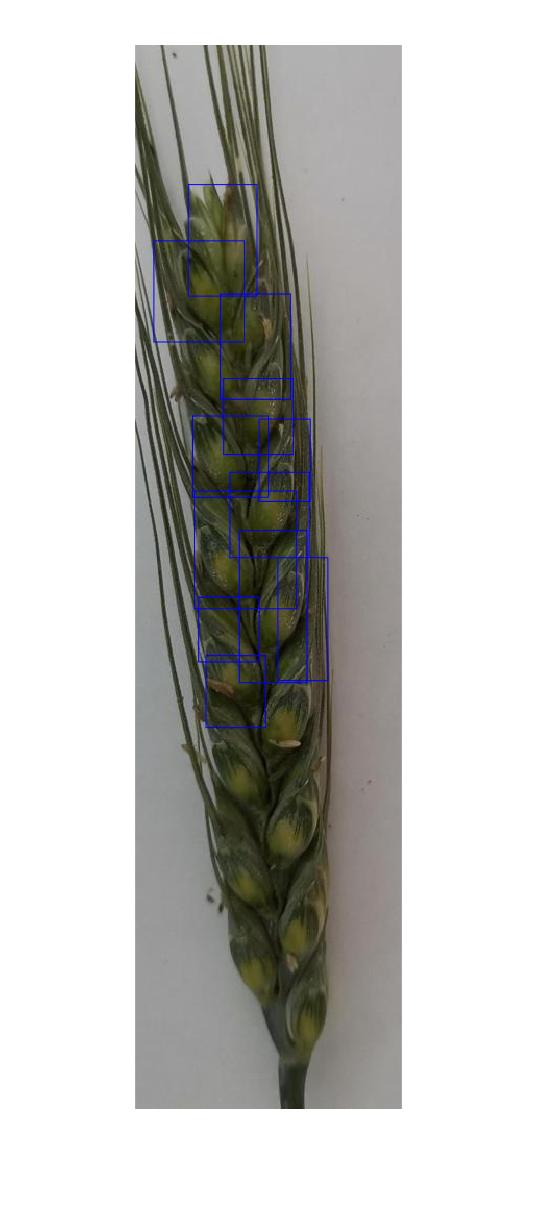

Supplement: Supplementary file 2 [file Data_Sheet_2.zip › 3. Labeling results of watershed algorithm (section Spikelet segmentation and annotation)/Shannong 25/2129b.jpg]

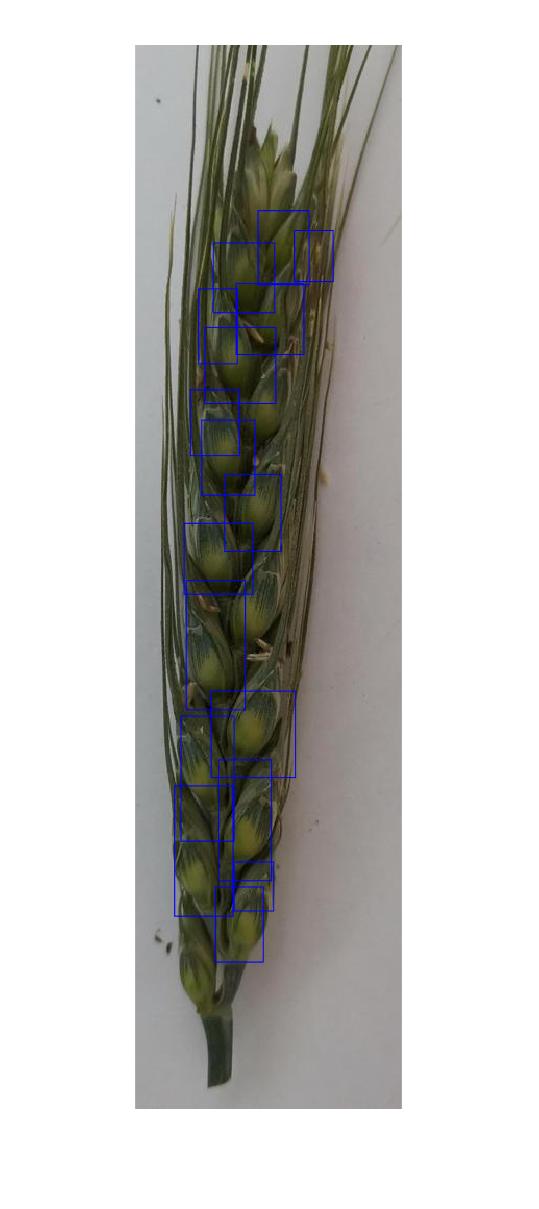

Supplement: Supplementary file 2 [file Data_Sheet_2.zip › 3. Labeling results of watershed algorithm (section Spikelet segmentation and annotation)/Shannong 25/2130b.jpg]

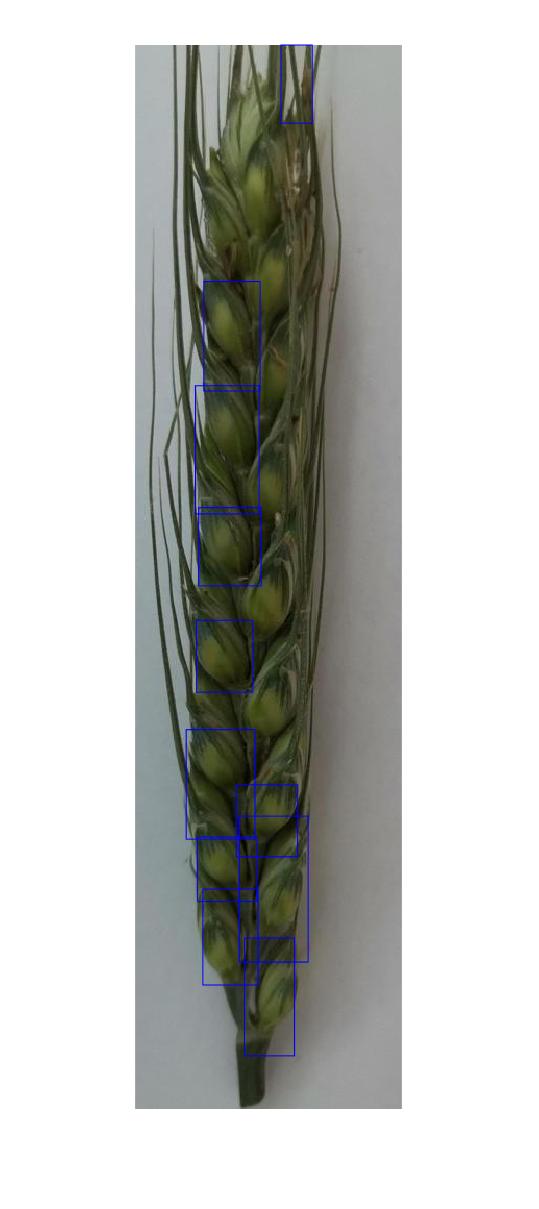

Supplement: Supplementary file 2 [file Data_Sheet_2.zip › 3. Labeling results of watershed algorithm (section Spikelet segmentation and annotation)/Shannong 25/2134b.jpg]

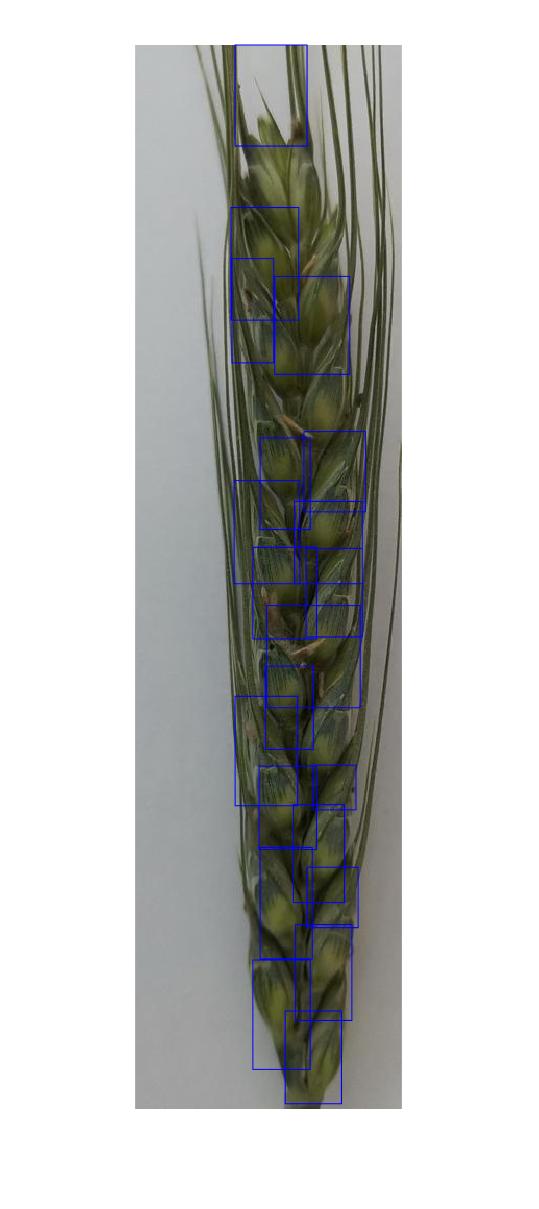

Supplement: Supplementary file 2 [file Data_Sheet_2.zip › 3. Labeling results of watershed algorithm (section Spikelet segmentation and annotation)/Shannong 25/2146b.jpg]

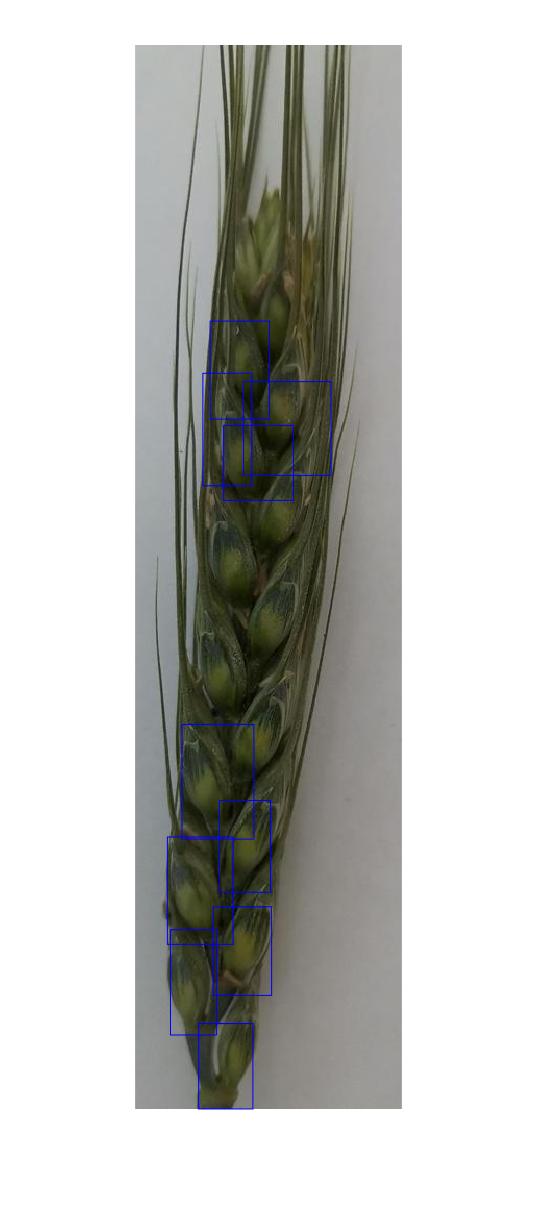

Supplement: Supplementary file 2 [file Data_Sheet_2.zip › 3. Labeling results of watershed algorithm (section Spikelet segmentation and annotation)/Shannong 25/2147b.jpg]

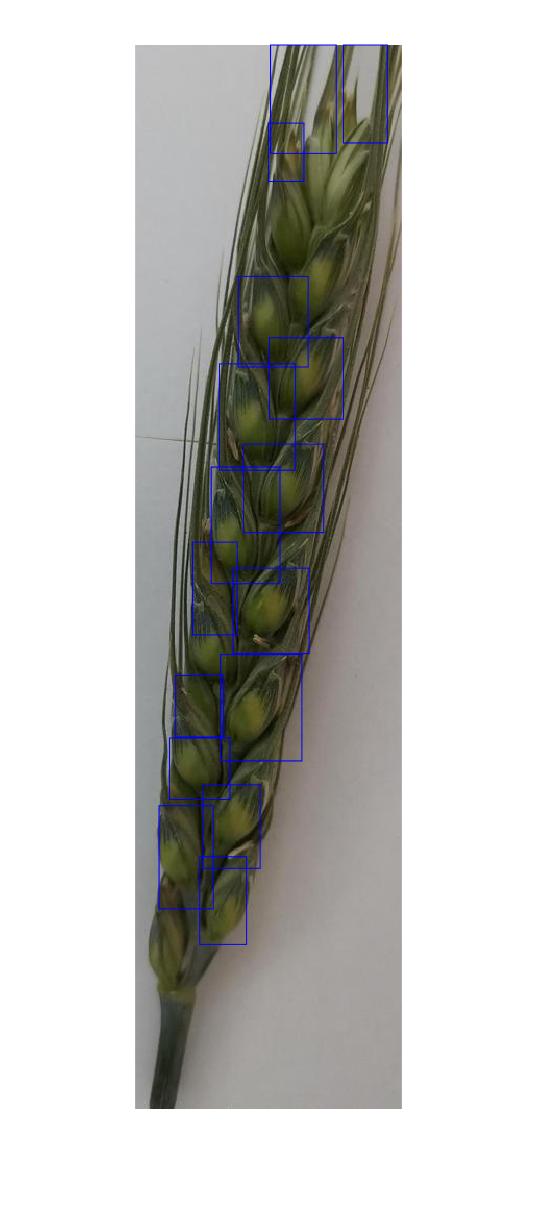

Supplement: Supplementary file 2 [file Data_Sheet_2.zip › 3. Labeling results of watershed algorithm (section Spikelet segmentation and annotation)/Shannong 25/2148b.jpg]

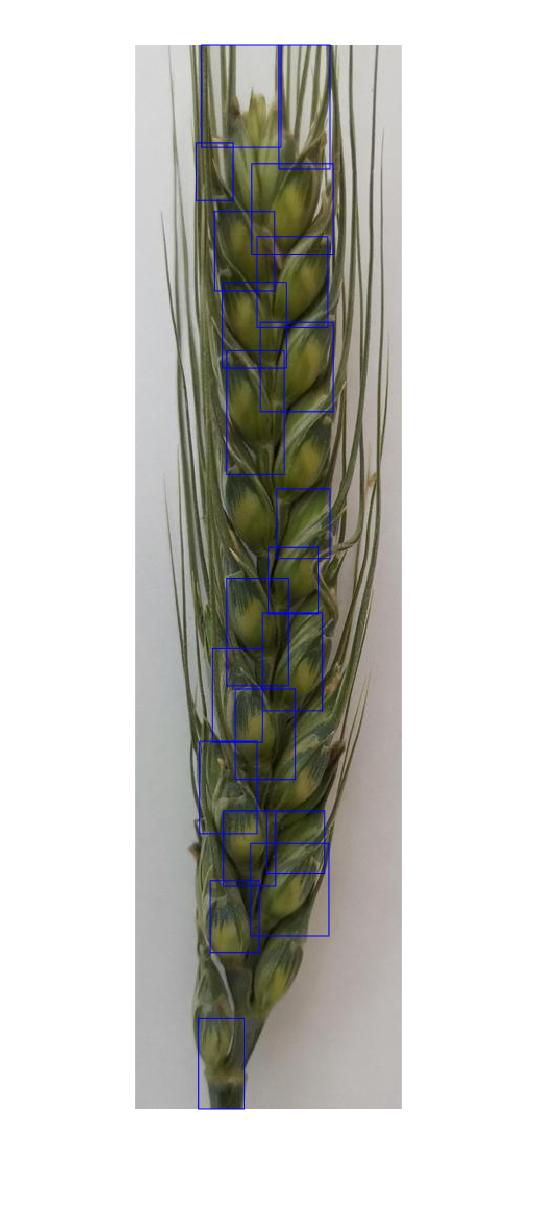

Supplement: Supplementary file 2 [file Data_Sheet_2.zip › 3. Labeling results of watershed algorithm (section Spikelet segmentation and annotation)/Shannong 25/2150b.jpg]

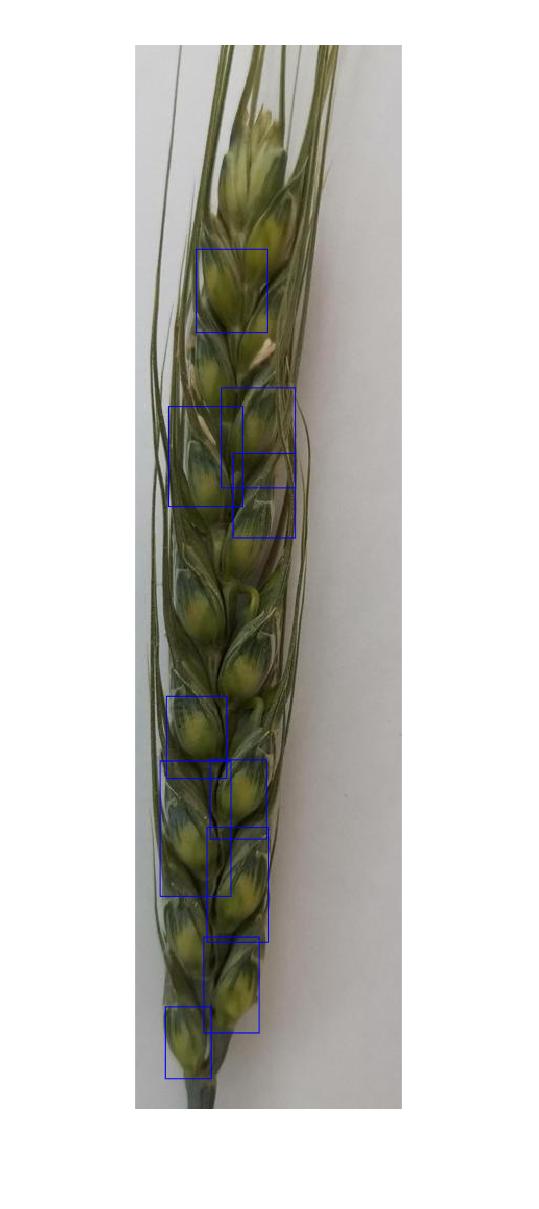

Supplement: Supplementary file 2 [file Data_Sheet_2.zip › 3. Labeling results of watershed algorithm (section Spikelet segmentation and annotation)/Shannong 25/2152b.jpg]

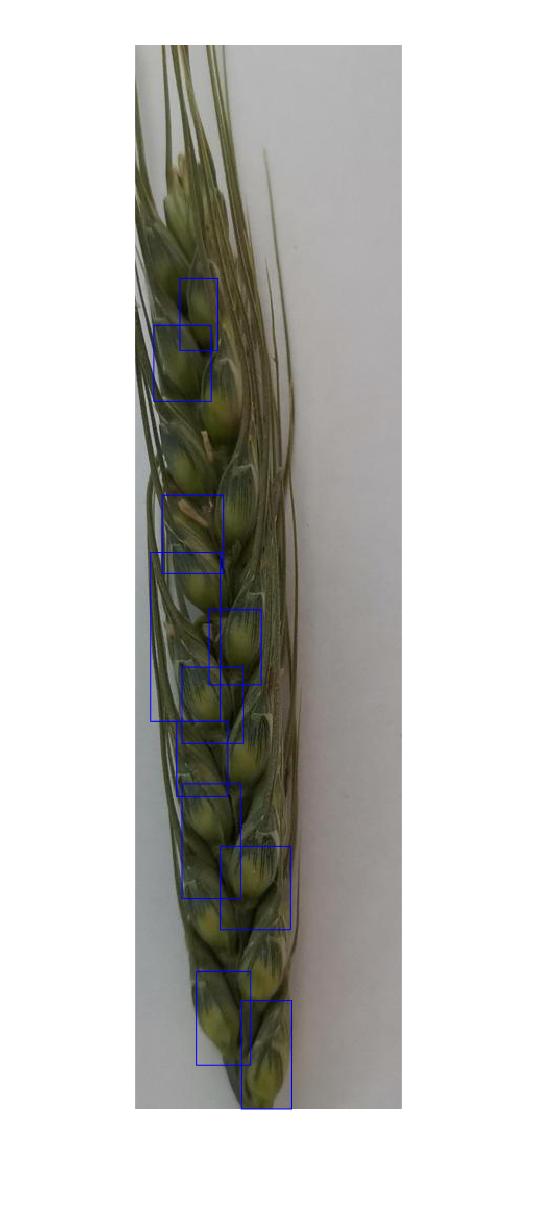

Supplement: Supplementary file 2 [file Data_Sheet_2.zip › 3. Labeling results of watershed algorithm (section Spikelet segmentation and annotation)/Shannong 25/2153b.jpg]

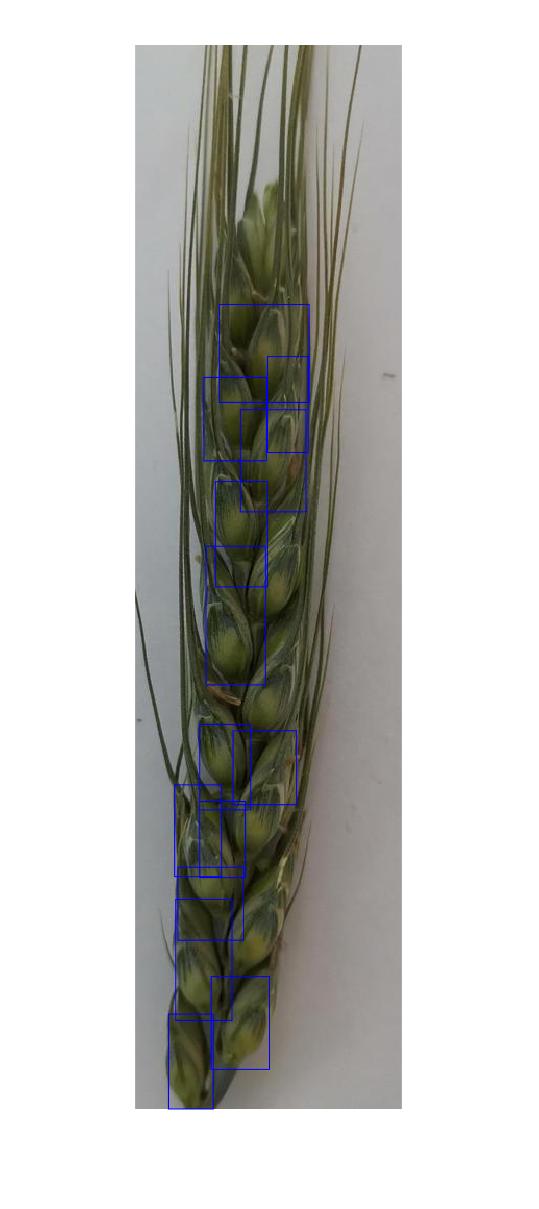

Supplement: Supplementary file 2 [file Data_Sheet_2.zip › 3. Labeling results of watershed algorithm (section Spikelet segmentation and annotation)/Shannong 25/2162b.jpg]

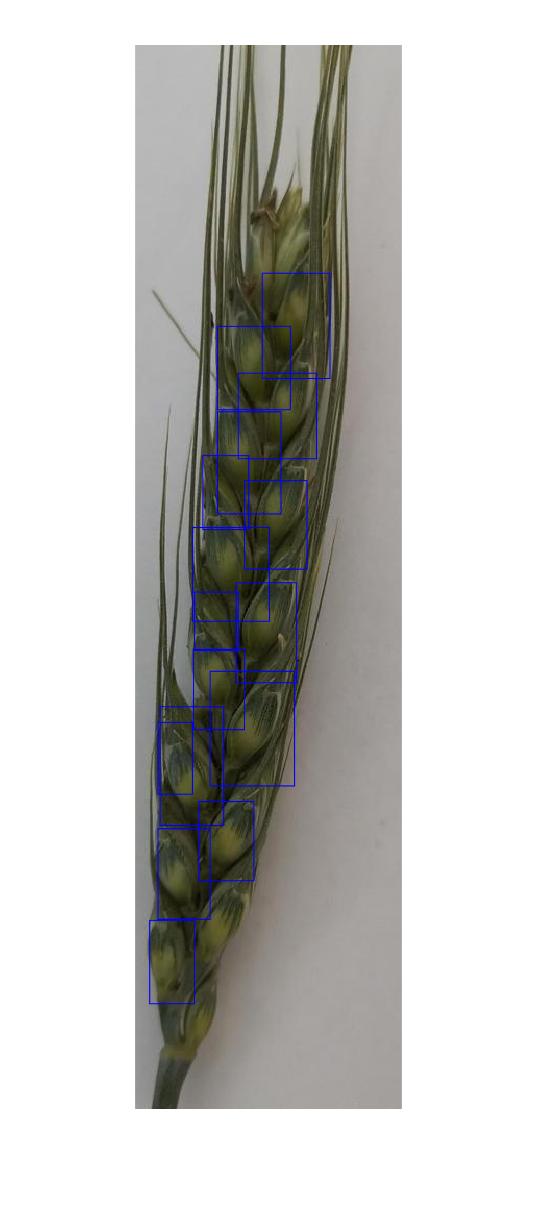

Supplement: Supplementary file 2 [file Data_Sheet_2.zip › 3. Labeling results of watershed algorithm (section Spikelet segmentation and annotation)/Shannong 25/2163b.jpg]

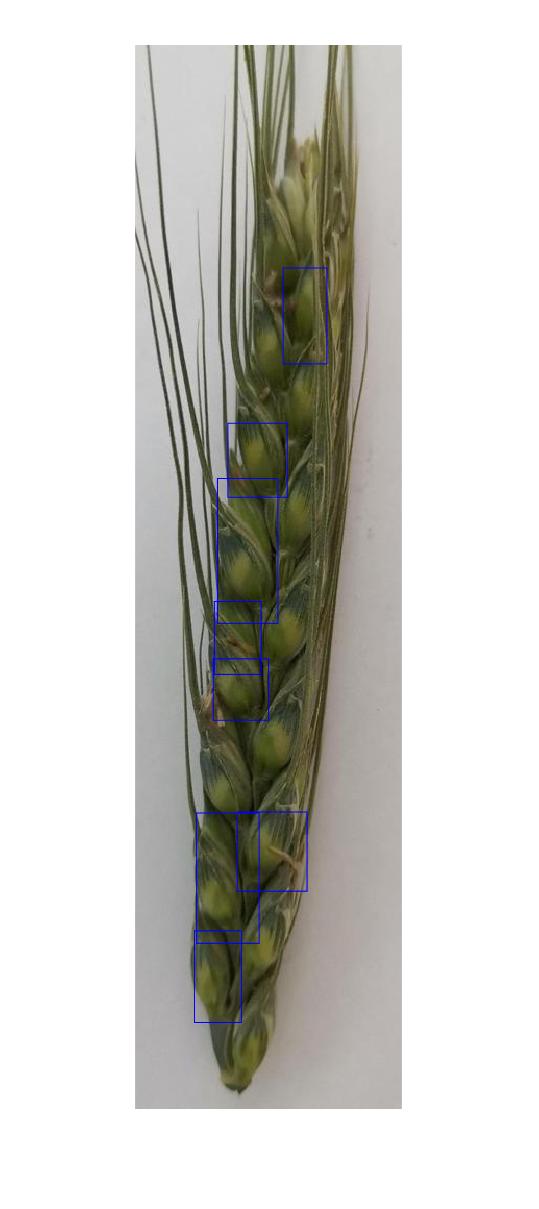

Supplement: Supplementary file 2 [file Data_Sheet_2.zip › 3. Labeling results of watershed algorithm (section Spikelet segmentation and annotation)/Shannong 25/2169b.jpg]

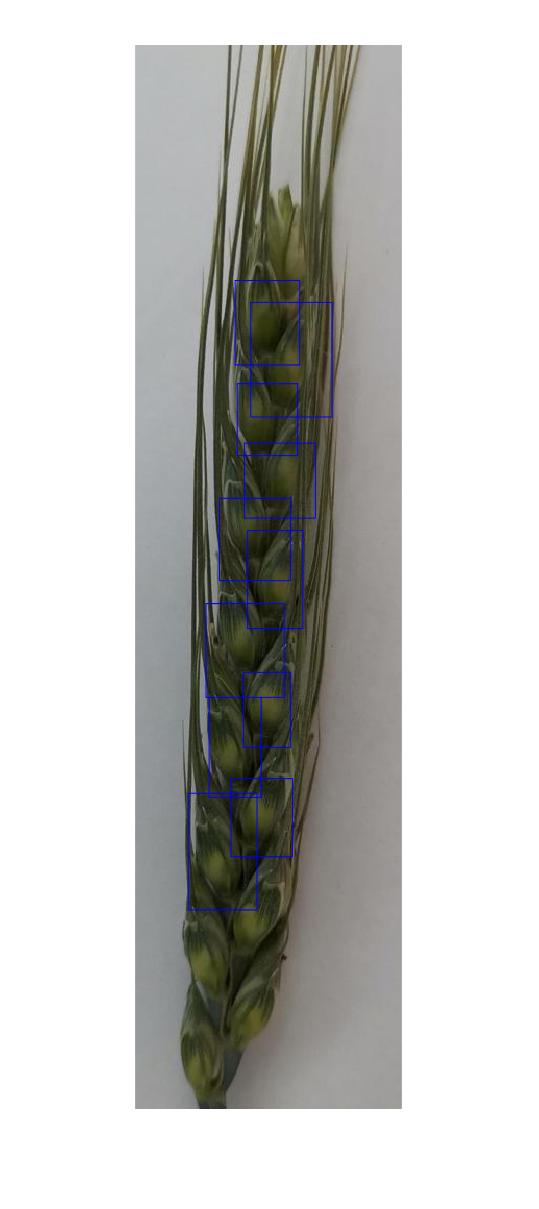

Supplement: Supplementary file 2 [file Data_Sheet_2.zip › 3. Labeling results of watershed algorithm (section Spikelet segmentation and annotation)/Shannong 25/2178b.jpg]

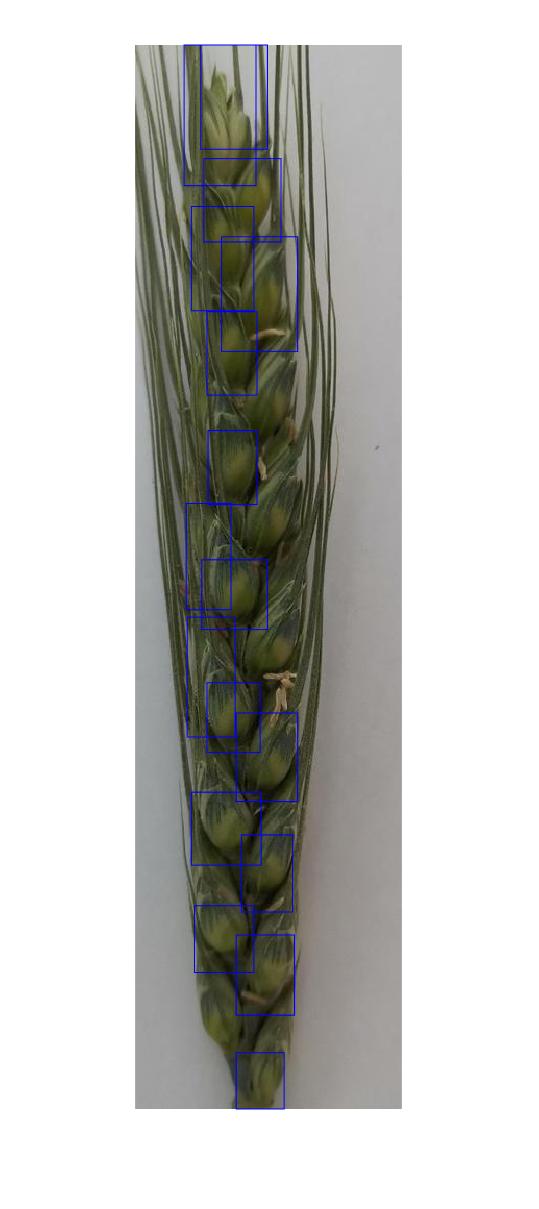

Supplement: Supplementary file 2 [file Data_Sheet_2.zip › 3. Labeling results of watershed algorithm (section Spikelet segmentation and annotation)/Shannong 25/2179b.jpg]

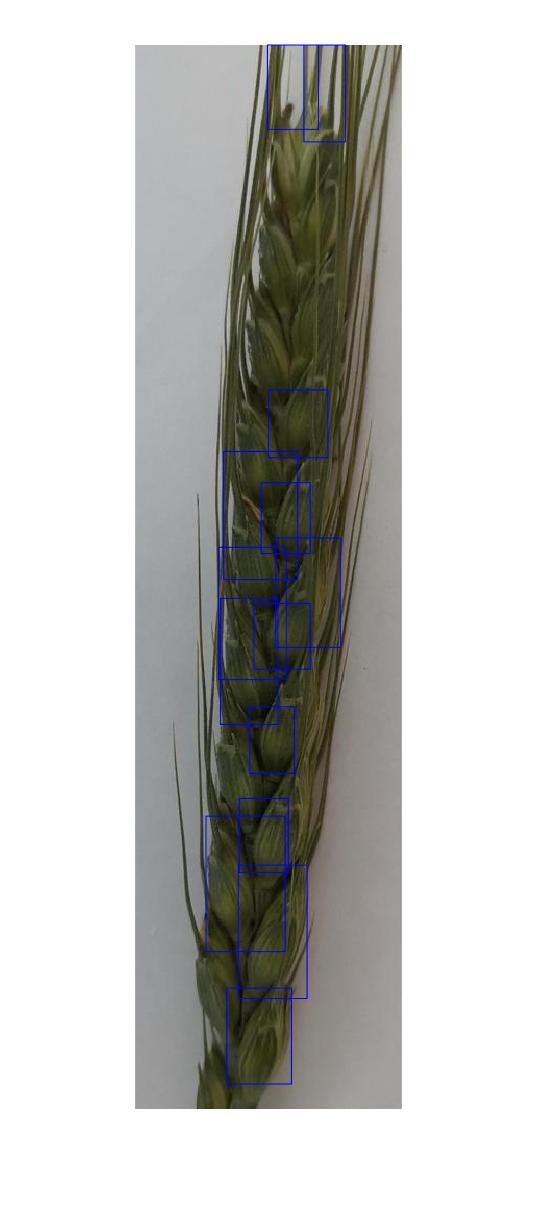

Supplement: Supplementary file 2 [file Data_Sheet_2.zip › 3. Labeling results of watershed algorithm (section Spikelet segmentation and annotation)/Shannong 25/2181b.jpg]

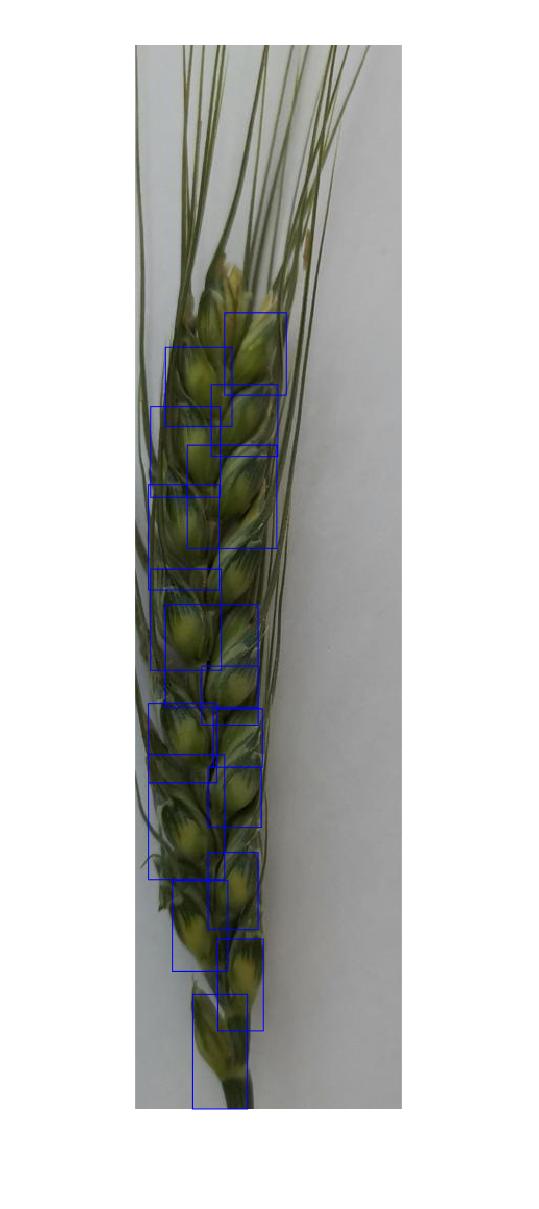

Supplement: Supplementary file 2 [file Data_Sheet_2.zip › 3. Labeling results of watershed algorithm (section Spikelet segmentation and annotation)/Shannong 25/2187b.jpg]

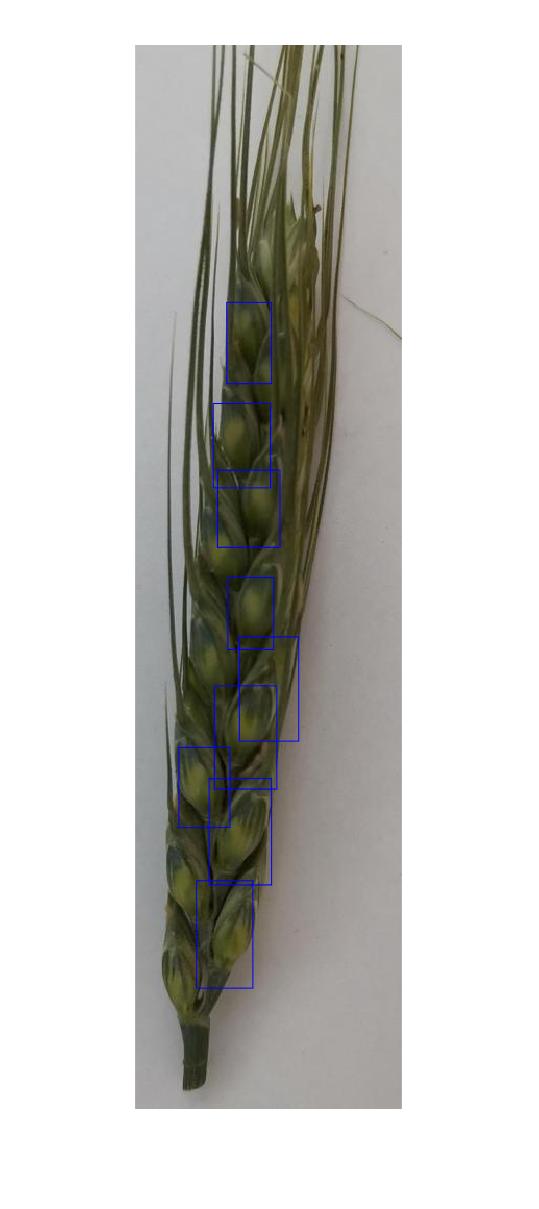

Supplement: Supplementary file 2 [file Data_Sheet_2.zip › 3. Labeling results of watershed algorithm (section Spikelet segmentation and annotation)/Shannong 25/2189b.jpg]

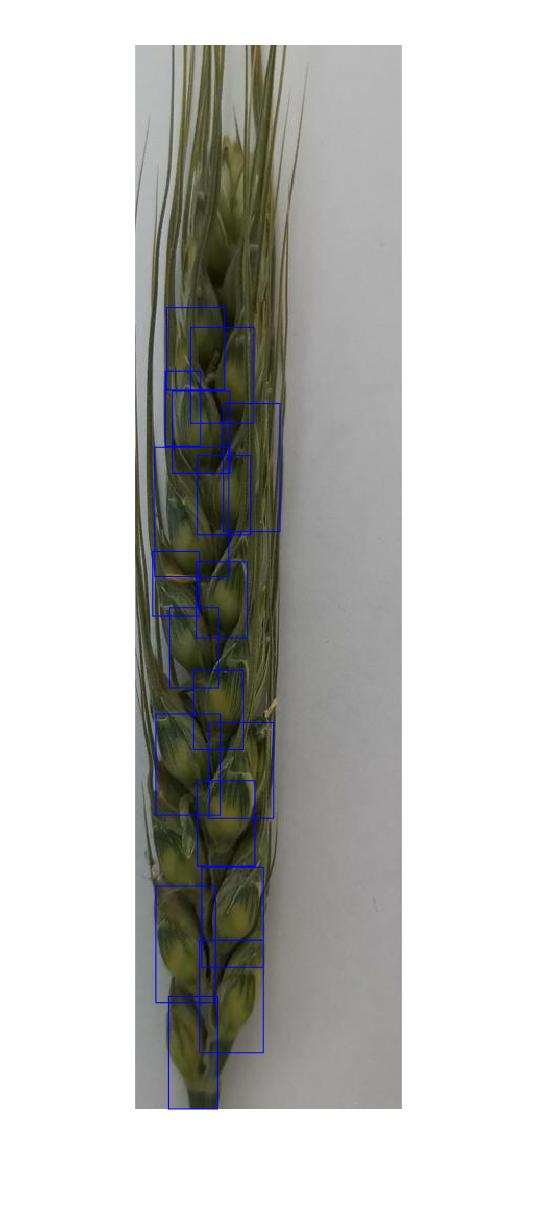

Supplement: Supplementary file 2 [file Data_Sheet_2.zip › 3. Labeling results of watershed algorithm (section Spikelet segmentation and annotation)/Shannong 25/2191b.jpg]

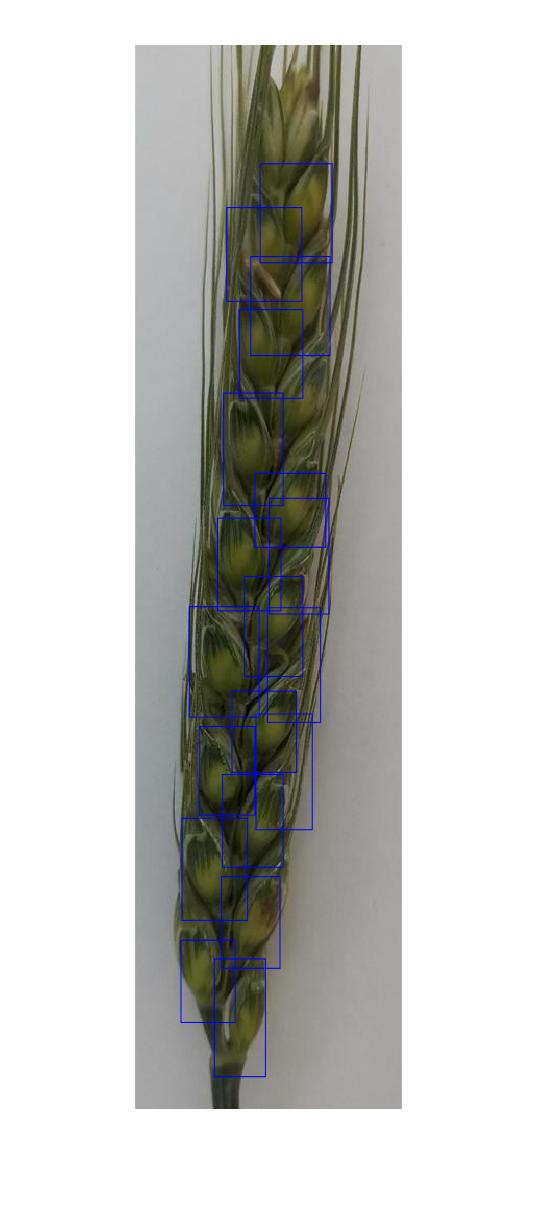

Supplement: Supplementary file 2 [file Data_Sheet_2.zip › 3. Labeling results of watershed algorithm (section Spikelet segmentation and annotation)/Shannong 25/2192b.jpg]

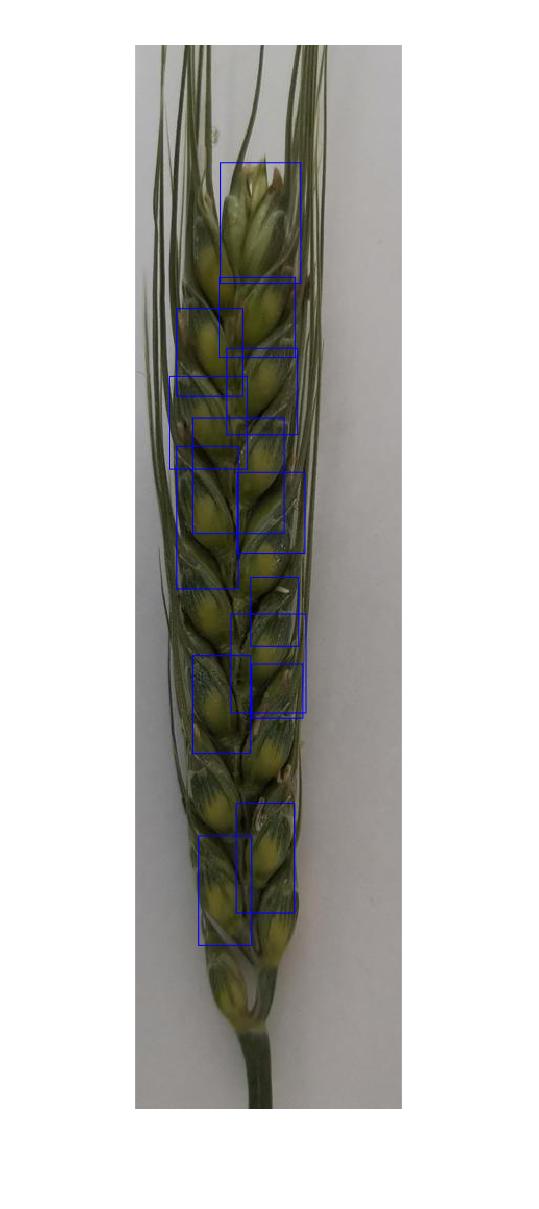

Supplement: Supplementary file 2 [file Data_Sheet_2.zip › 3. Labeling results of watershed algorithm (section Spikelet segmentation and annotation)/Shannong 25/2195b.jpg]

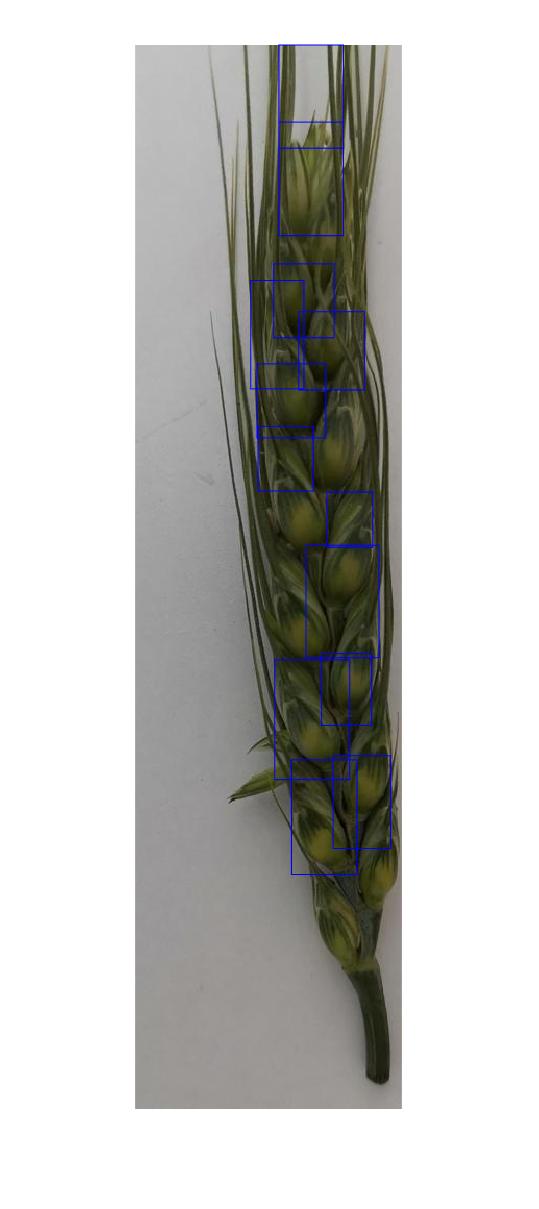

Supplement: Supplementary file 2 [file Data_Sheet_2.zip › 3. Labeling results of watershed algorithm (section Spikelet segmentation and annotation)/Shannong 25/2197b.jpg]

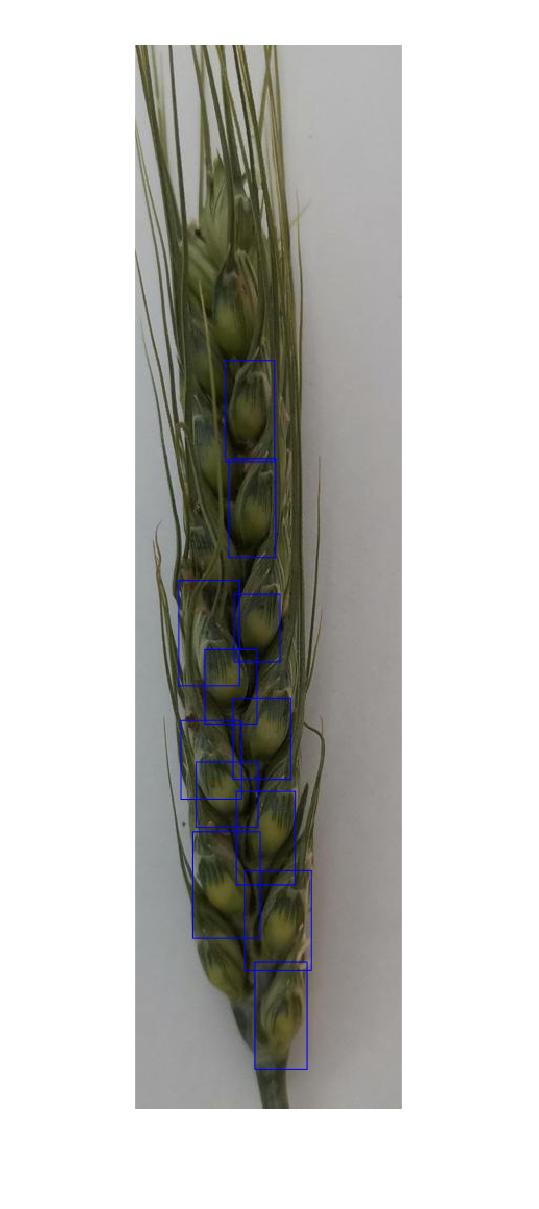

Supplement: Supplementary file 2 [file Data_Sheet_2.zip › 3. Labeling results of watershed algorithm (section Spikelet segmentation and annotation)/Shannong 25/2201b.jpg]

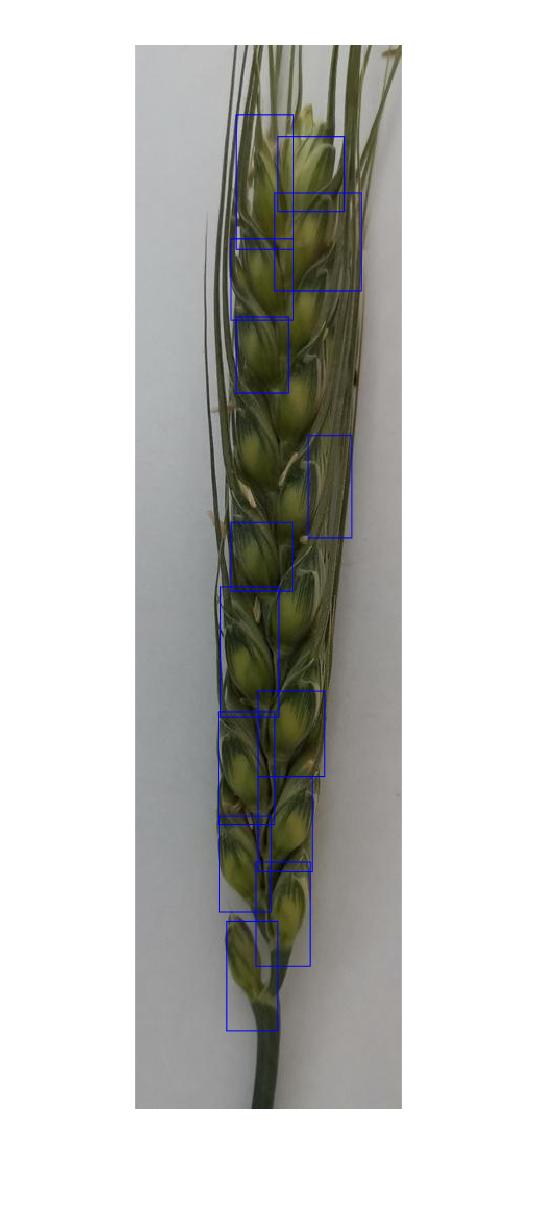

Supplement: Supplementary file 2 [file Data_Sheet_2.zip › 3. Labeling results of watershed algorithm (section Spikelet segmentation and annotation)/Shannong 25/2211b.jpg]

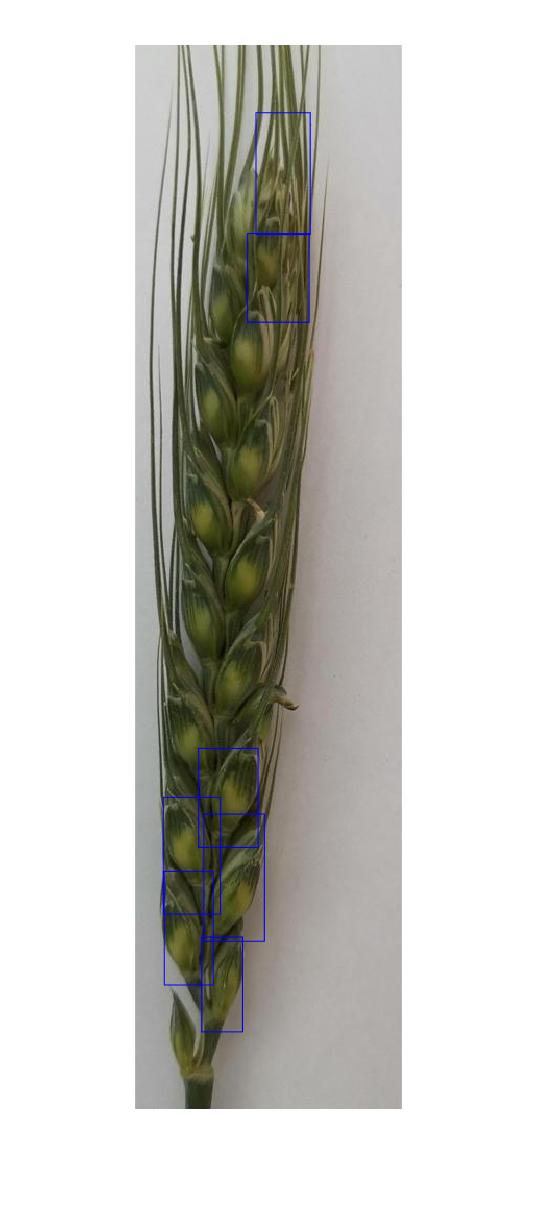

Supplement: Supplementary file 2 [file Data_Sheet_2.zip › 3. Labeling results of watershed algorithm (section Spikelet segmentation and annotation)/Shannong 25/2212b.jpg]

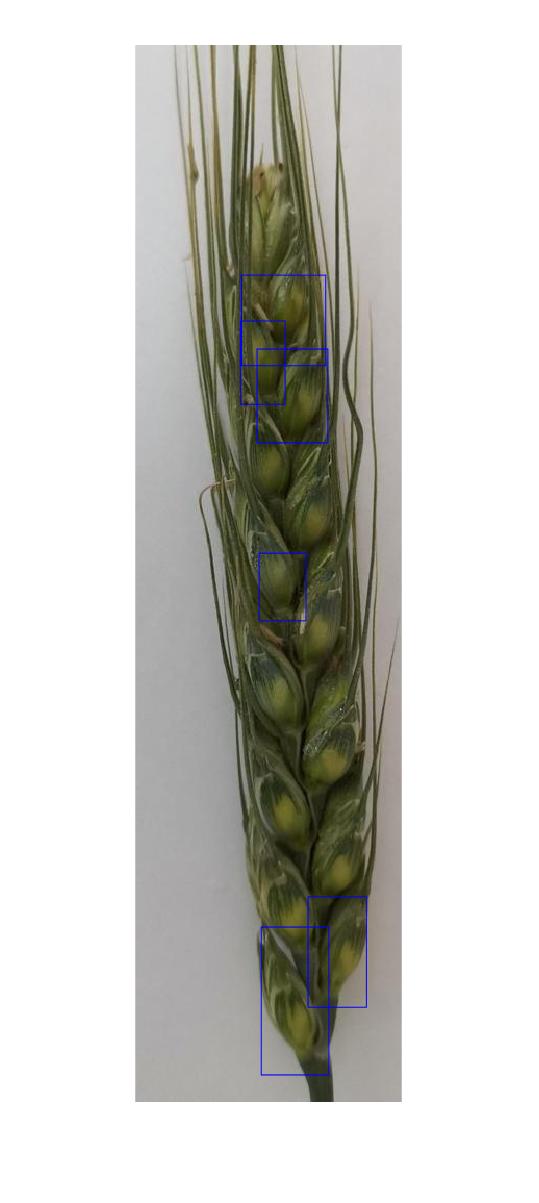

Supplement: Supplementary file 2 [file Data_Sheet_2.zip › 3. Labeling results of watershed algorithm (section Spikelet segmentation and annotation)/Shannong 25/2213b.jpg]

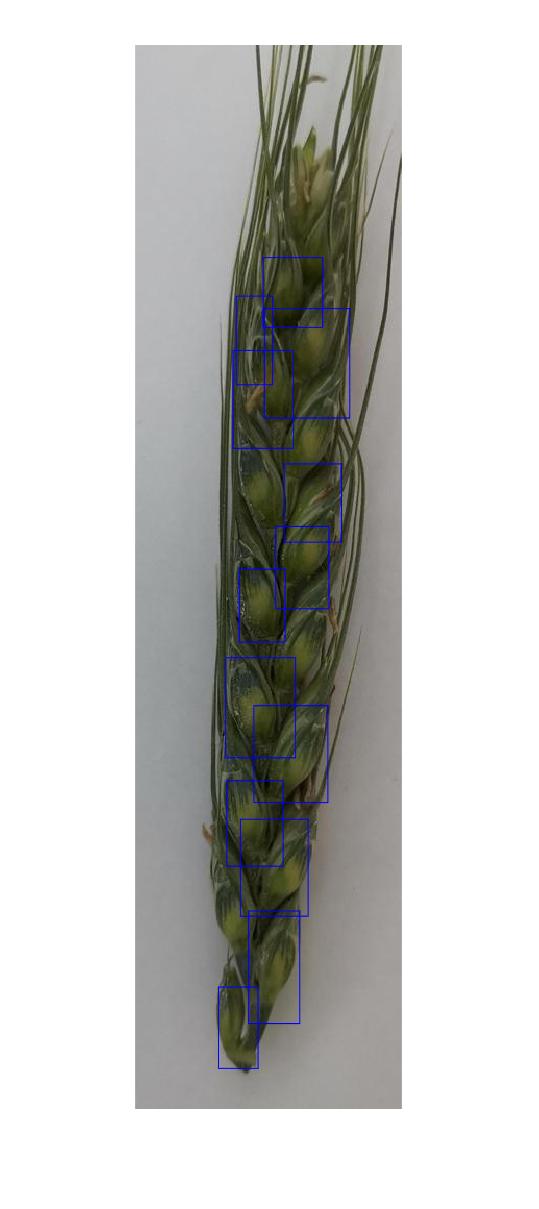

Supplement: Supplementary file 2 [file Data_Sheet_2.zip › 3. Labeling results of watershed algorithm (section Spikelet segmentation and annotation)/Shannong 25/2221b.jpg]

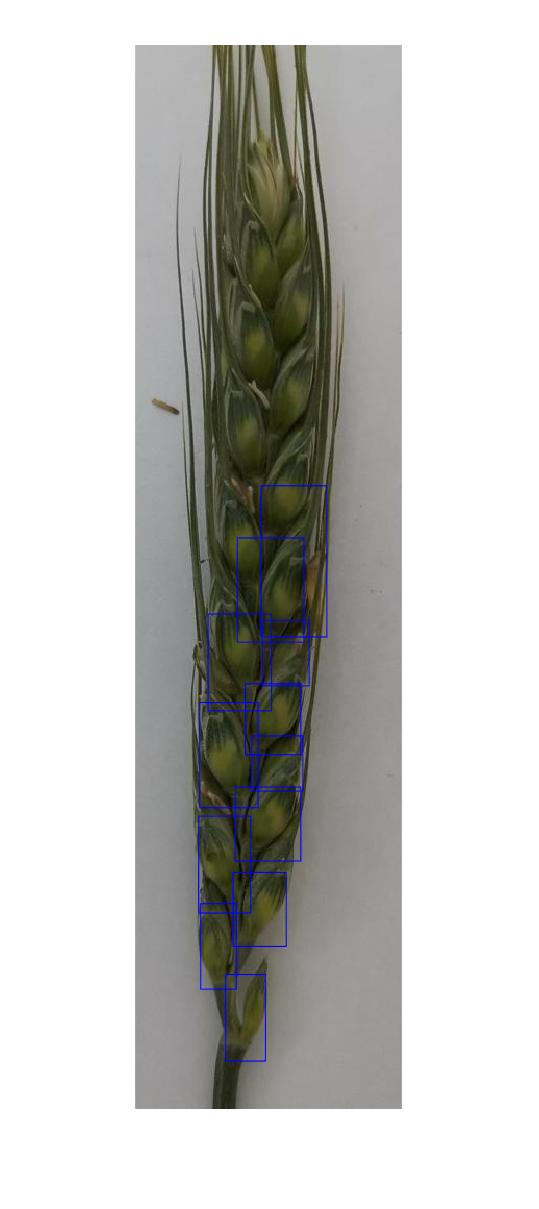

Supplement: Supplementary file 2 [file Data_Sheet_2.zip › 3. Labeling results of watershed algorithm (section Spikelet segmentation and annotation)/Shannong 25/2227b.jpg]

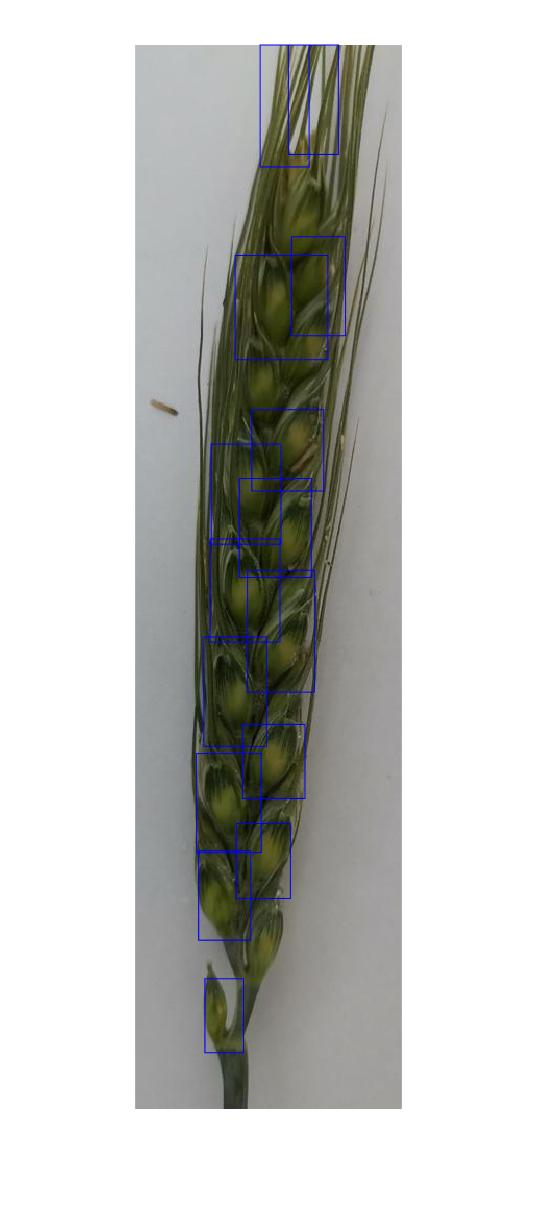

Supplement: Supplementary file 2 [file Data_Sheet_2.zip › 3. Labeling results of watershed algorithm (section Spikelet segmentation and annotation)/Shannong 25/2228b.jpg]

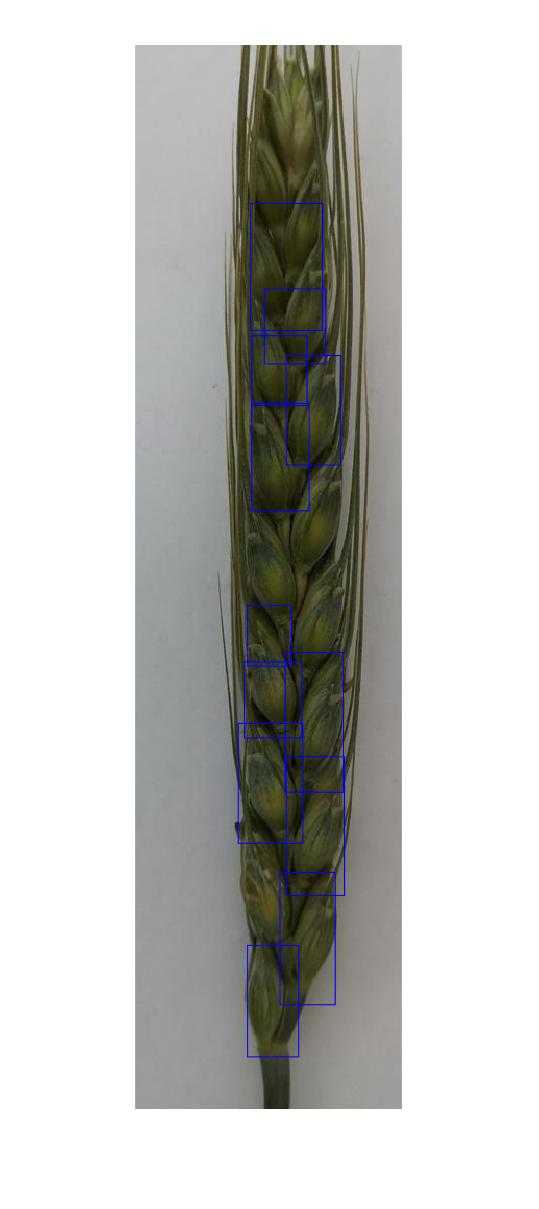

Supplement: Supplementary file 2 [file Data_Sheet_2.zip › 3. Labeling results of watershed algorithm (section Spikelet segmentation and annotation)/Shannong 25/2230b.jpg]

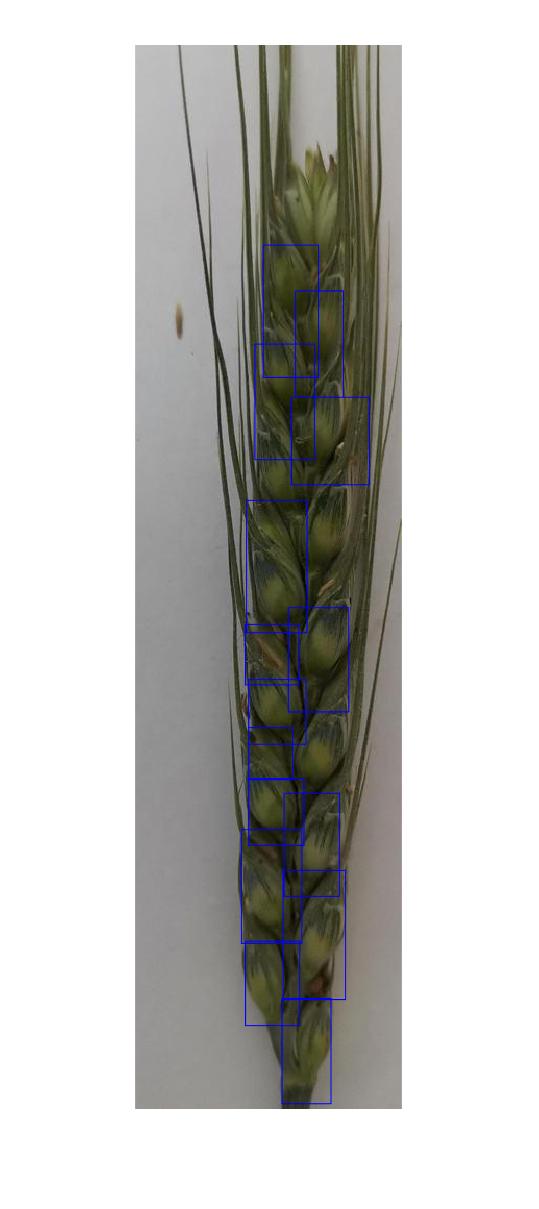

Supplement: Supplementary file 2 [file Data_Sheet_2.zip › 3. Labeling results of watershed algorithm (section Spikelet segmentation and annotation)/Shannong 25/2231b.jpg]

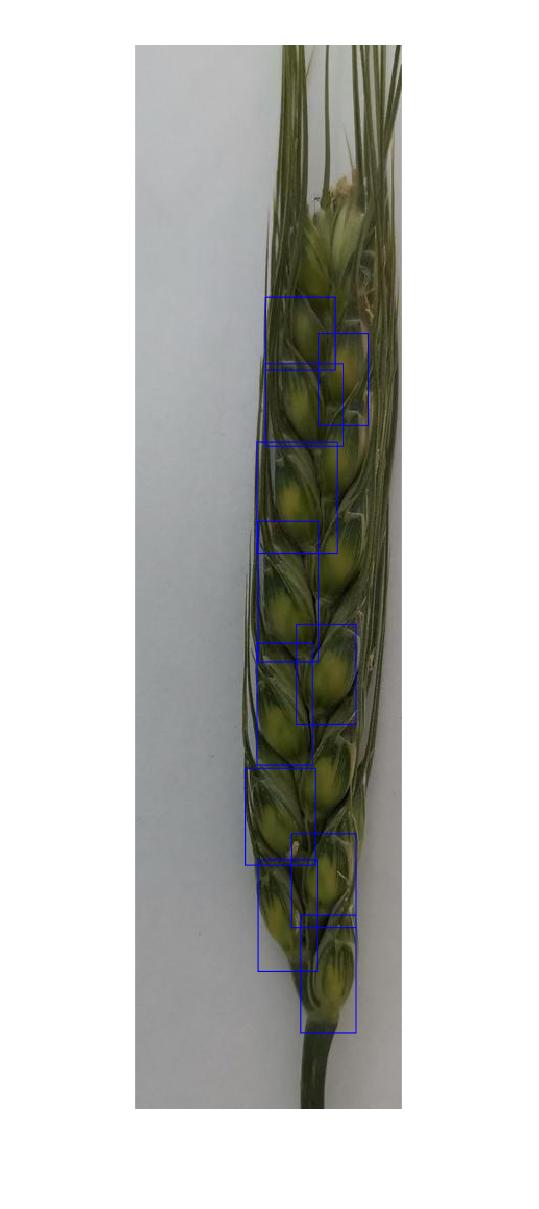

Supplement: Supplementary file 2 [file Data_Sheet_2.zip › 3. Labeling results of watershed algorithm (section Spikelet segmentation and annotation)/Shannong 25/2239b.jpg]

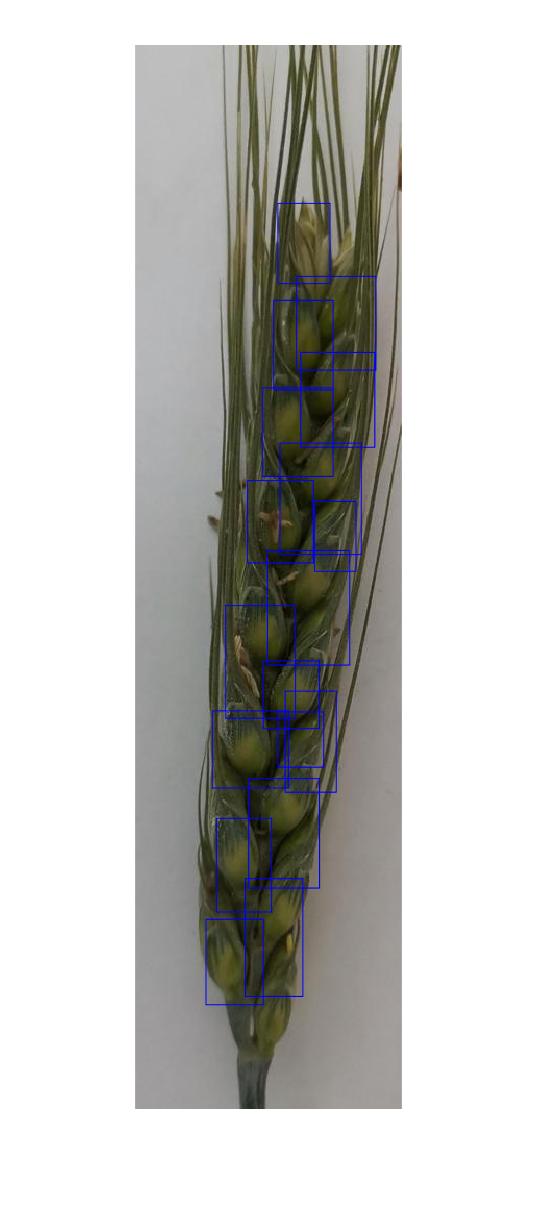

Supplement: Supplementary file 2 [file Data_Sheet_2.zip › 3. Labeling results of watershed algorithm (section Spikelet segmentation and annotation)/Shannong 25/2240b.jpg]

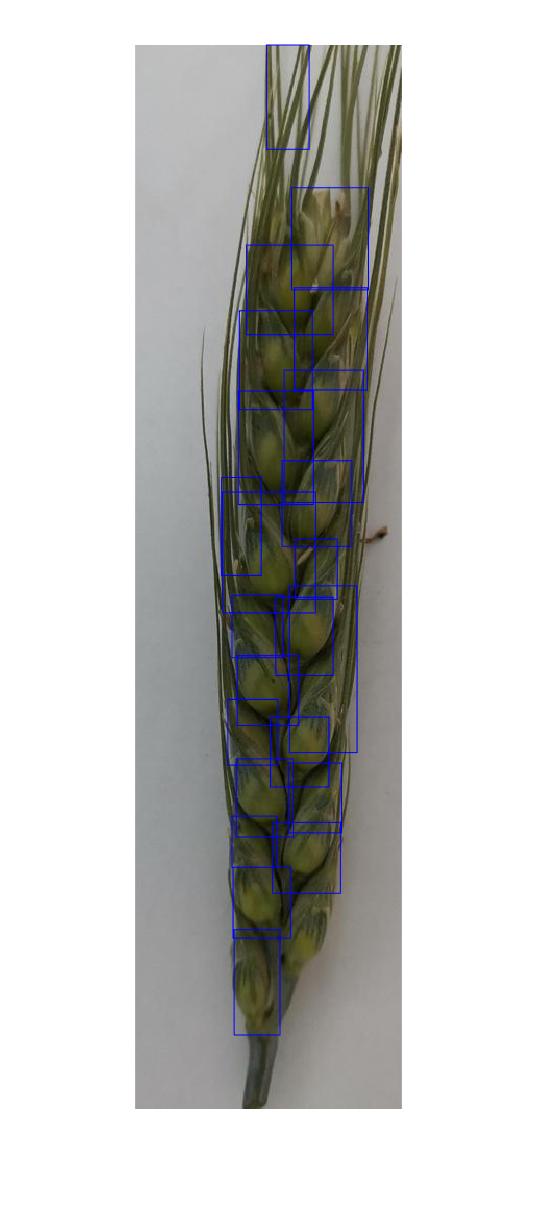

Supplement: Supplementary file 2 [file Data_Sheet_2.zip › 3. Labeling results of watershed algorithm (section Spikelet segmentation and annotation)/Shannong 25/2241b.jpg]

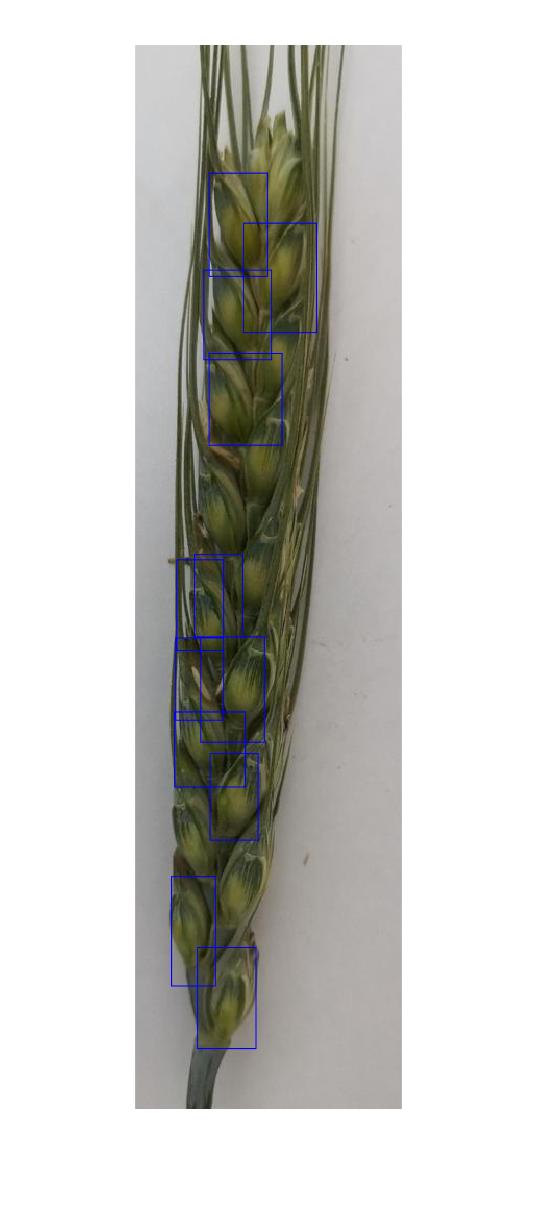

Supplement: Supplementary file 2 [file Data_Sheet_2.zip › 3. Labeling results of watershed algorithm (section Spikelet segmentation and annotation)/Shannong 25/2243b.jpg]

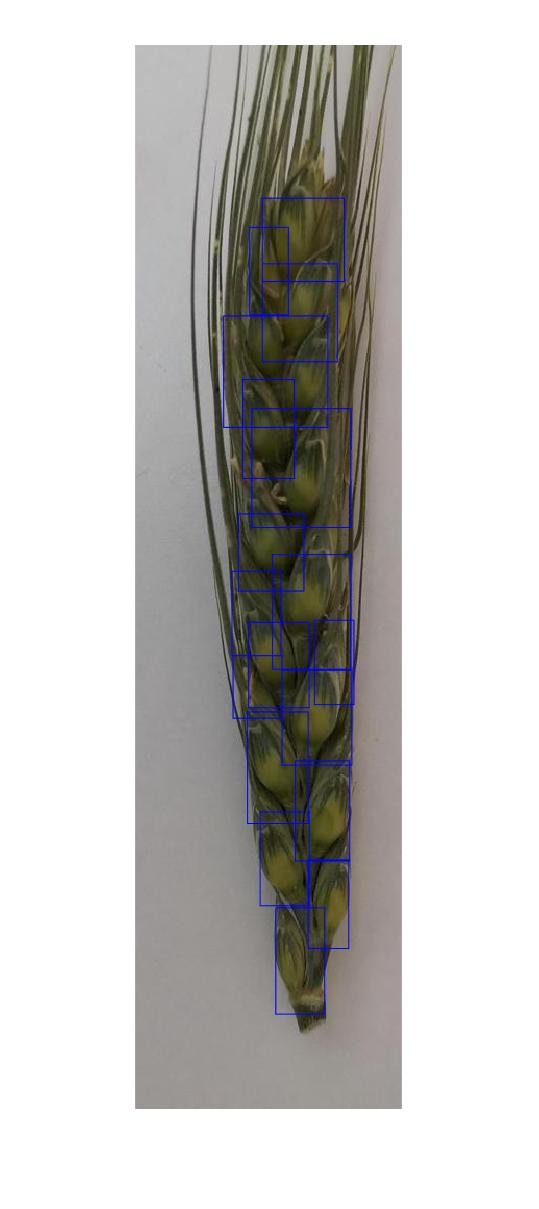

Supplement: Supplementary file 2 [file Data_Sheet_2.zip › 3. Labeling results of watershed algorithm (section Spikelet segmentation and annotation)/Shannong 25/2248b.jpg]

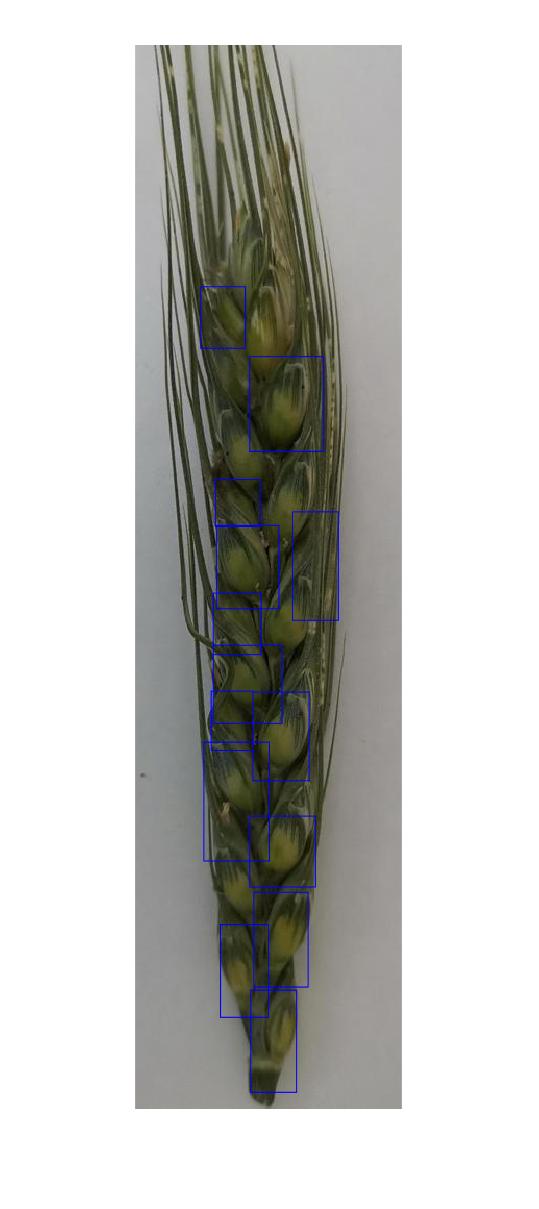

Supplement: Supplementary file 2 [file Data_Sheet_2.zip › 3. Labeling results of watershed algorithm (section Spikelet segmentation and annotation)/Shannong 25/2249b.jpg]

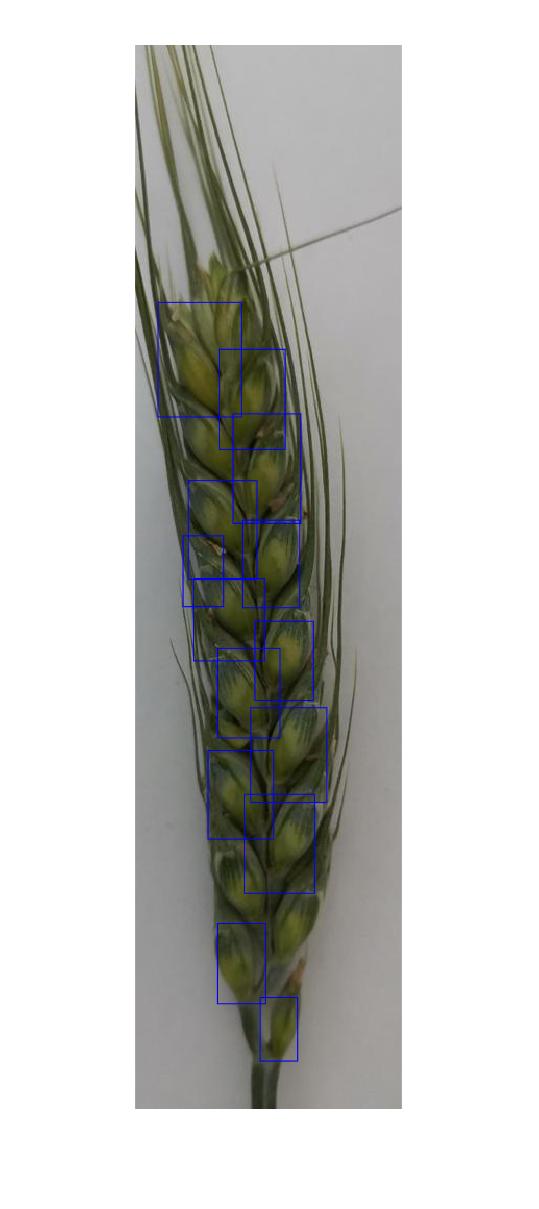

Supplement: Supplementary file 2 [file Data_Sheet_2.zip › 3. Labeling results of watershed algorithm (section Spikelet segmentation and annotation)/Shannong 25/2258b.jpg]

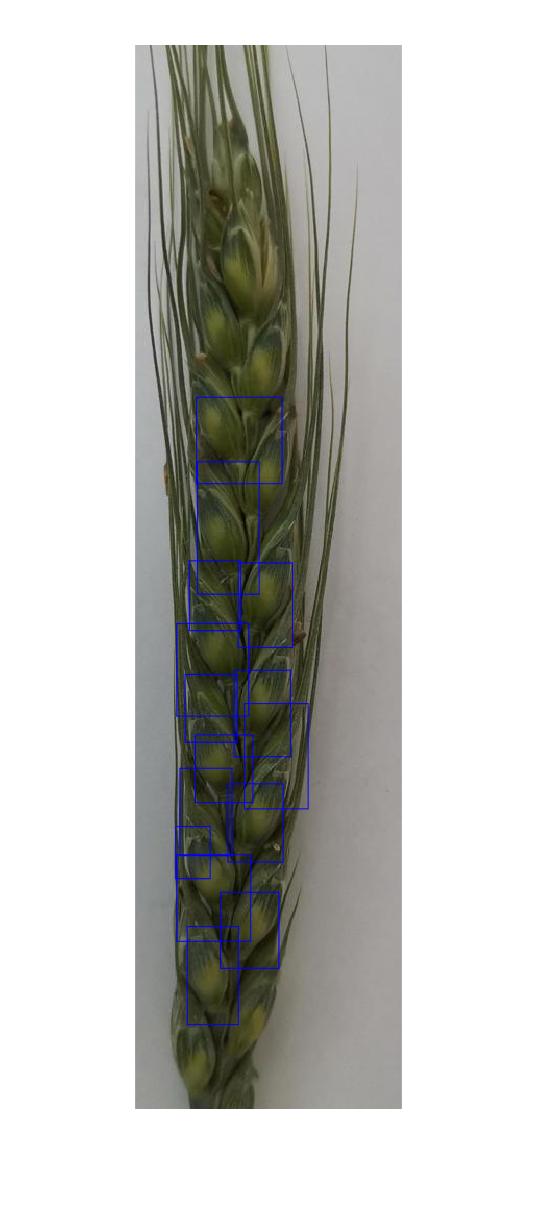

Supplement: Supplementary file 2 [file Data_Sheet_2.zip › 3. Labeling results of watershed algorithm (section Spikelet segmentation and annotation)/Shannong 25/2259b.jpg]

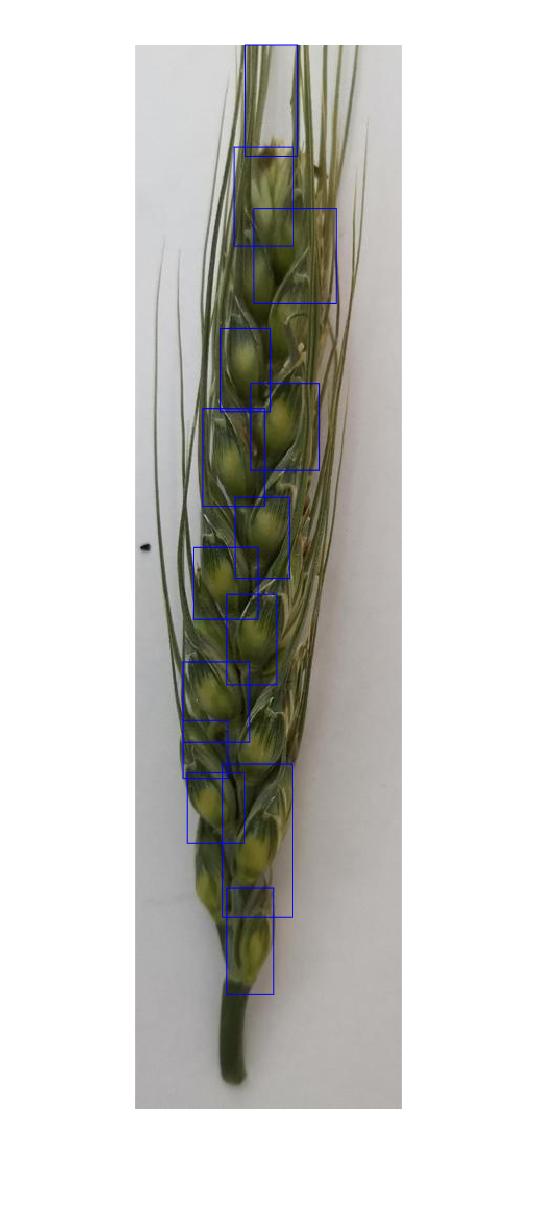

Supplement: Supplementary file 2 [file Data_Sheet_2.zip › 3. Labeling results of watershed algorithm (section Spikelet segmentation and annotation)/Shannong 25/2261b.jpg]

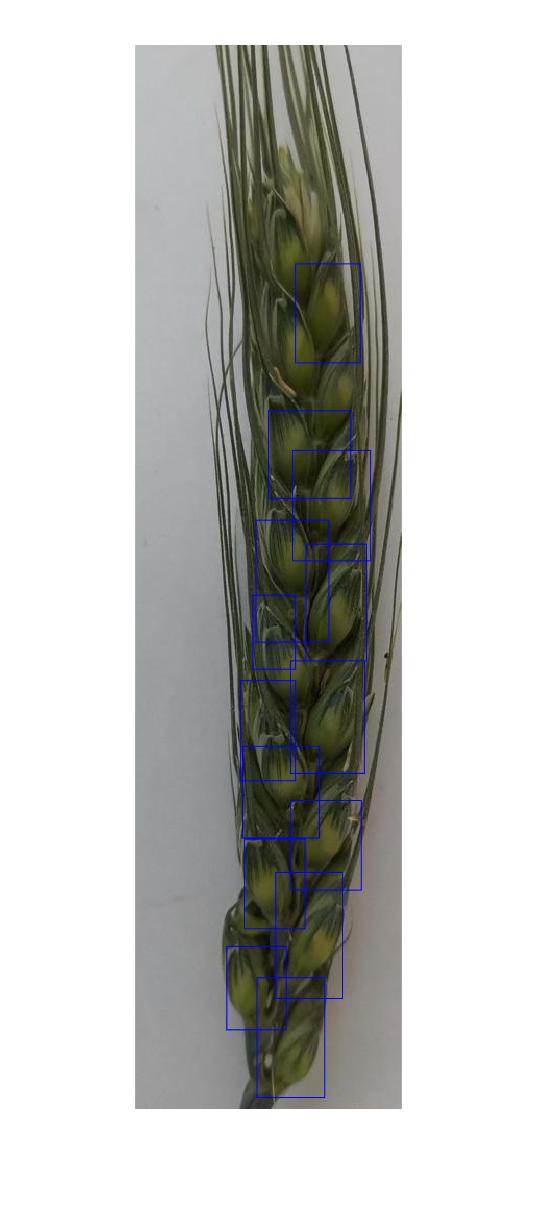

Supplement: Supplementary file 2 [file Data_Sheet_2.zip › 3. Labeling results of watershed algorithm (section Spikelet segmentation and annotation)/Shannong 25/2263b.jpg]

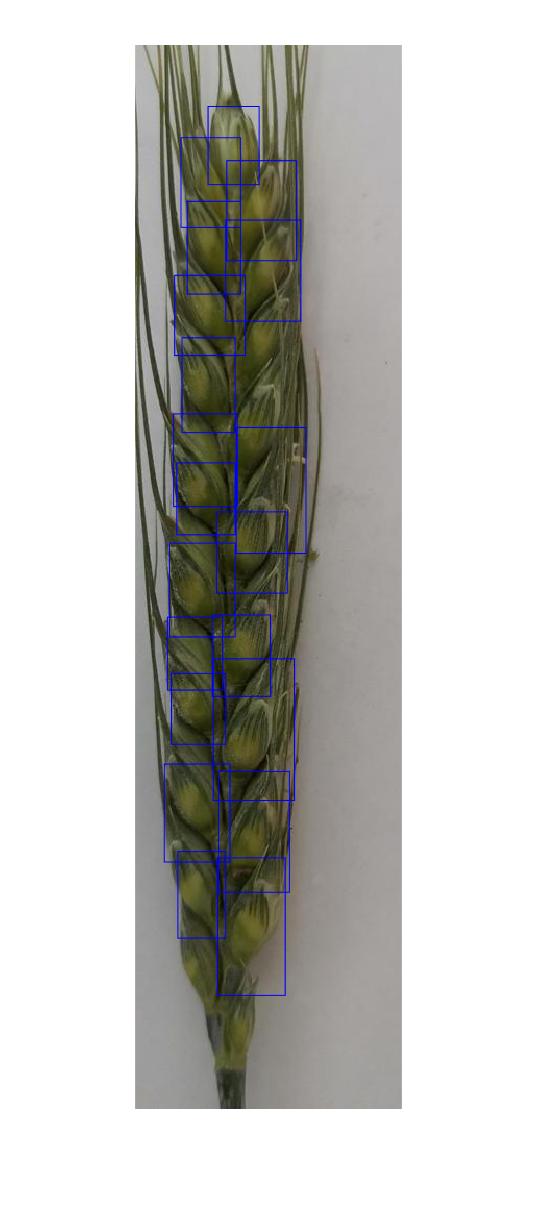

Supplement: Supplementary file 2 [file Data_Sheet_2.zip › 3. Labeling results of watershed algorithm (section Spikelet segmentation and annotation)/Shannong 25/2266b.jpg]

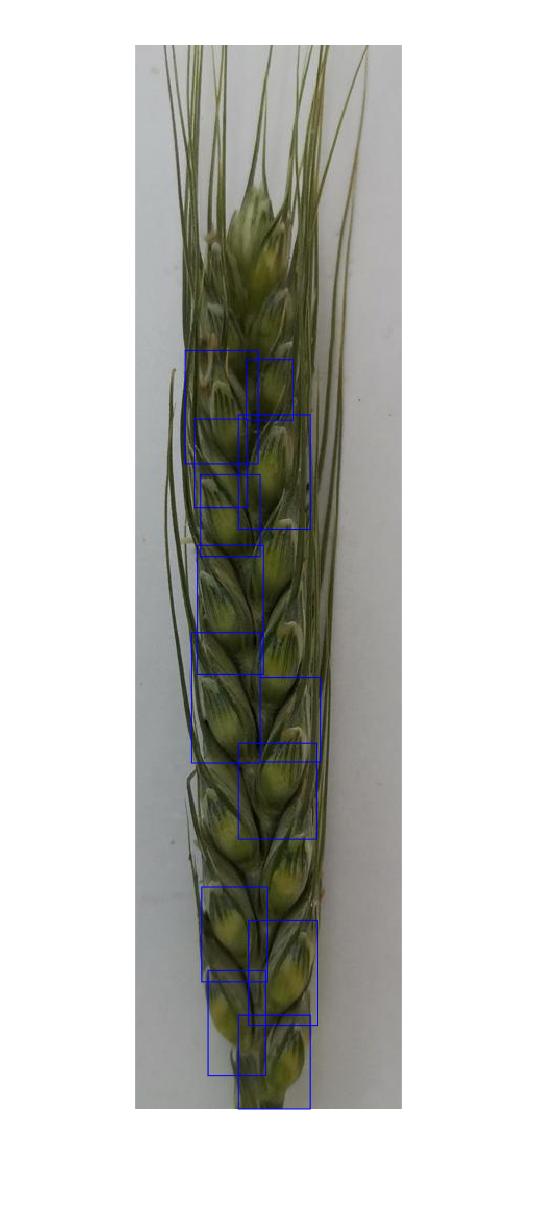

Supplement: Supplementary file 2 [file Data_Sheet_2.zip › 3. Labeling results of watershed algorithm (section Spikelet segmentation and annotation)/Shannong 25/2267b.jpg]

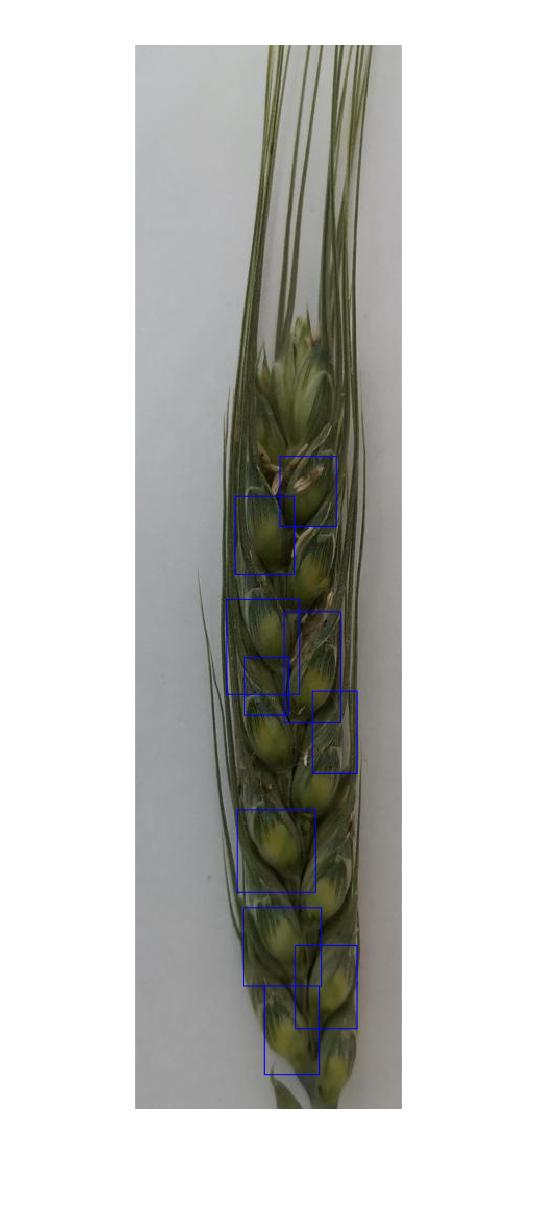

Supplement: Supplementary file 2 [file Data_Sheet_2.zip › 3. Labeling results of watershed algorithm (section Spikelet segmentation and annotation)/Shannong 25/2270b.jpg]

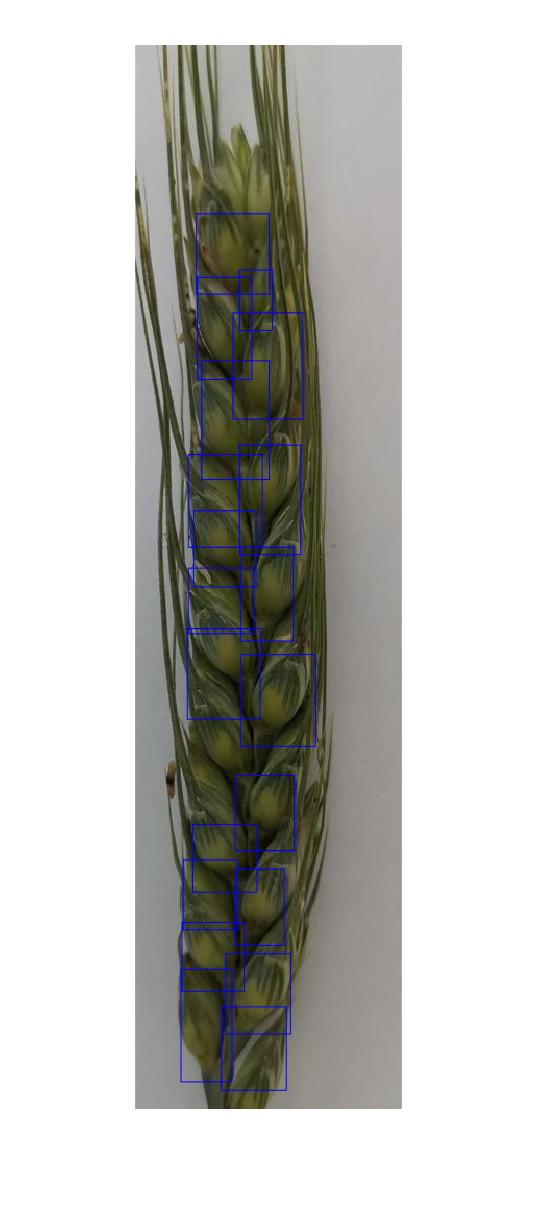

Supplement: Supplementary file 2 [file Data_Sheet_2.zip › 3. Labeling results of watershed algorithm (section Spikelet segmentation and annotation)/Shannong 25/2273b.jpg]

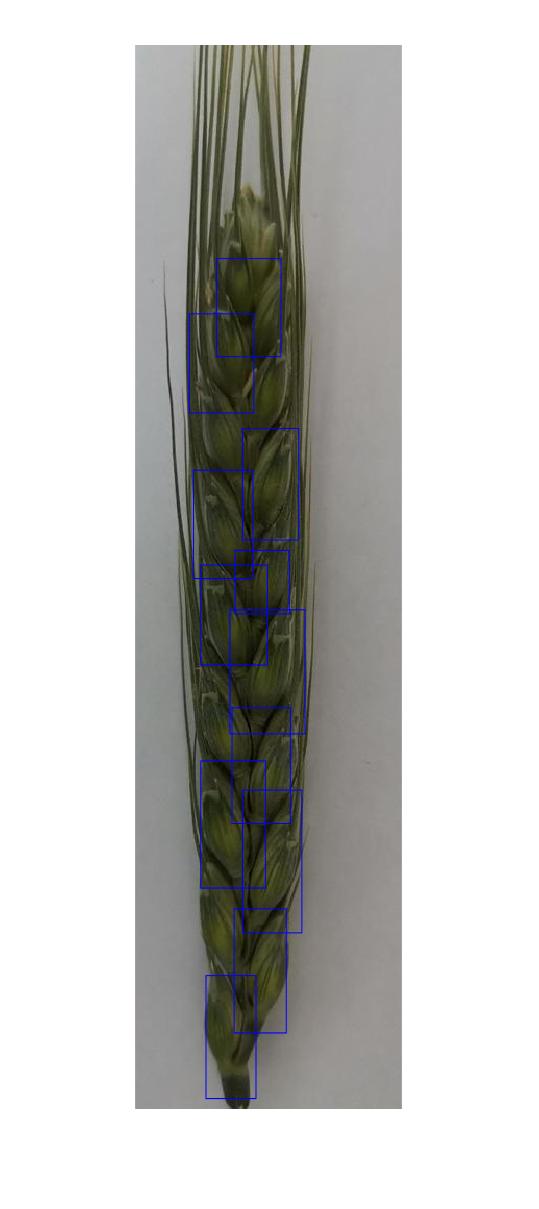

Supplement: Supplementary file 2 [file Data_Sheet_2.zip › 3. Labeling results of watershed algorithm (section Spikelet segmentation and annotation)/Shannong 25/2275b.jpg]

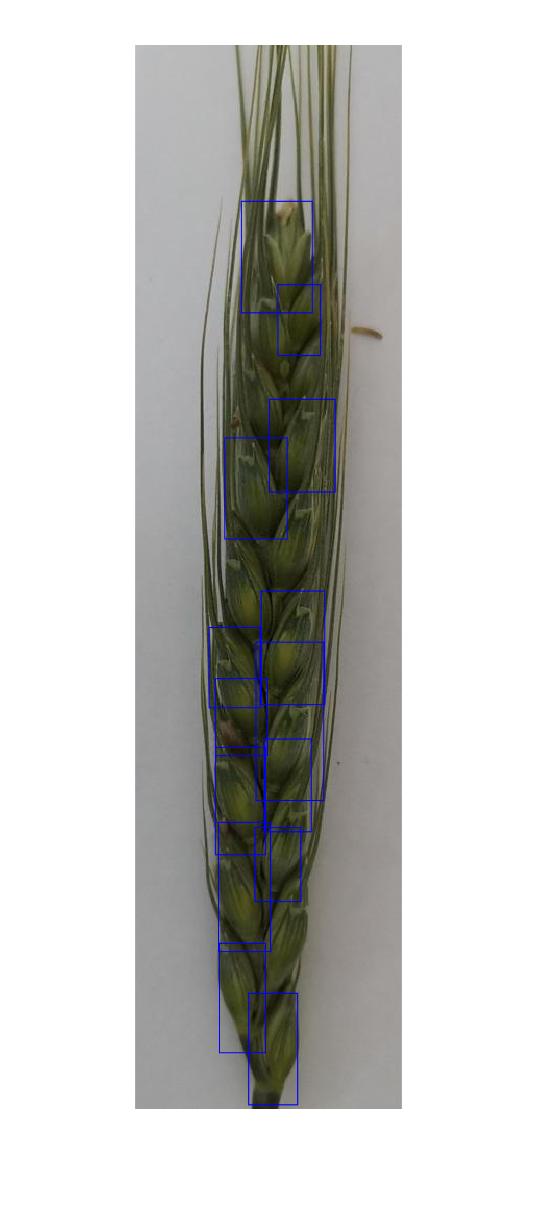

Supplement: Supplementary file 2 [file Data_Sheet_2.zip › 3. Labeling results of watershed algorithm (section Spikelet segmentation and annotation)/Shannong 25/2276b.jpg]

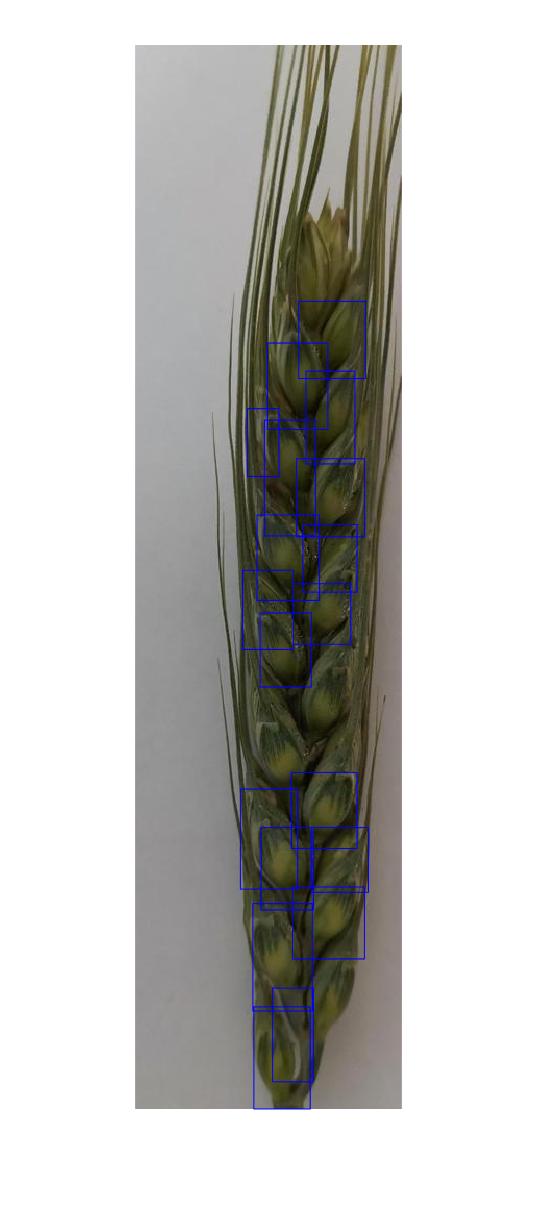

Supplement: Supplementary file 2 [file Data_Sheet_2.zip › 3. Labeling results of watershed algorithm (section Spikelet segmentation and annotation)/Shannong 25/2280b.jpg]

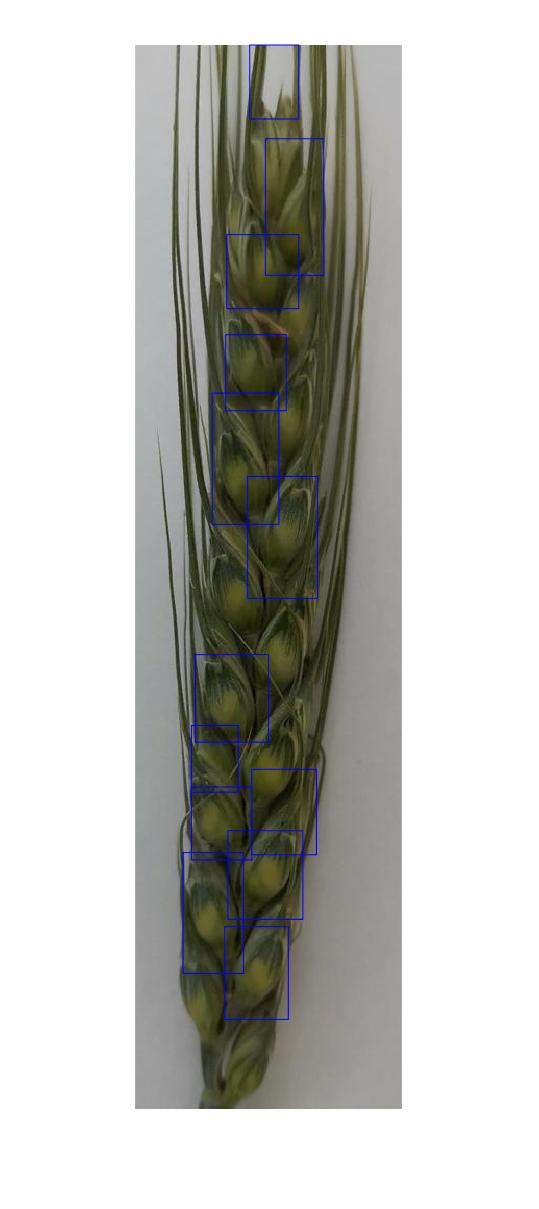

Supplement: Supplementary file 2 [file Data_Sheet_2.zip › 3. Labeling results of watershed algorithm (section Spikelet segmentation and annotation)/Shannong 25/2282b.jpg]

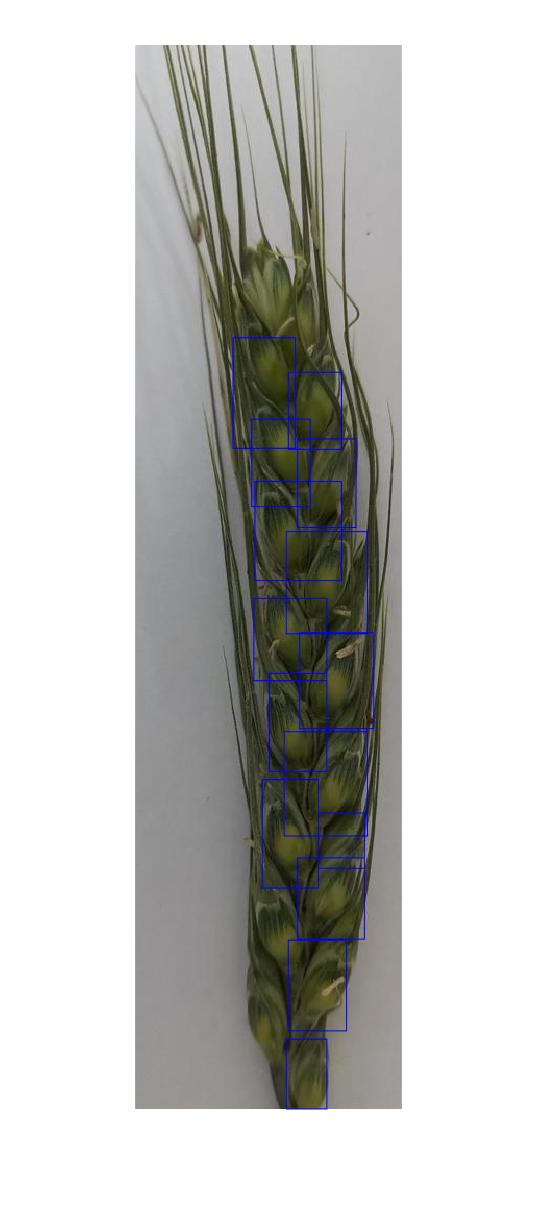

Supplement: Supplementary file 2 [file Data_Sheet_2.zip › 3. Labeling results of watershed algorithm (section Spikelet segmentation and annotation)/Shannong 25/2286b.jpg]

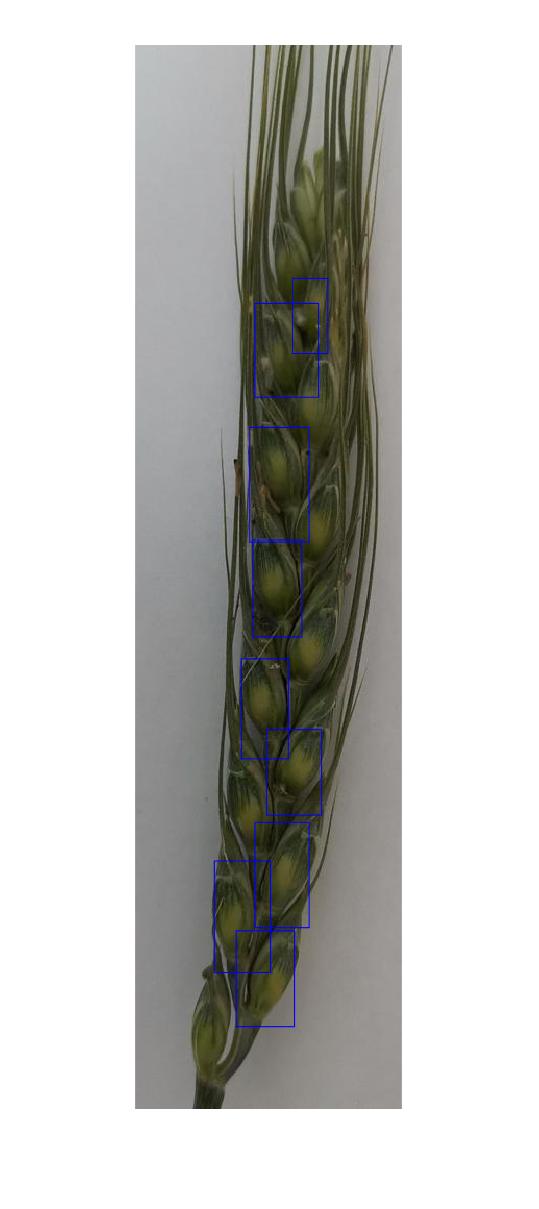

Supplement: Supplementary file 2 [file Data_Sheet_2.zip › 3. Labeling results of watershed algorithm (section Spikelet segmentation and annotation)/Shannong 25/2287b.jpg]

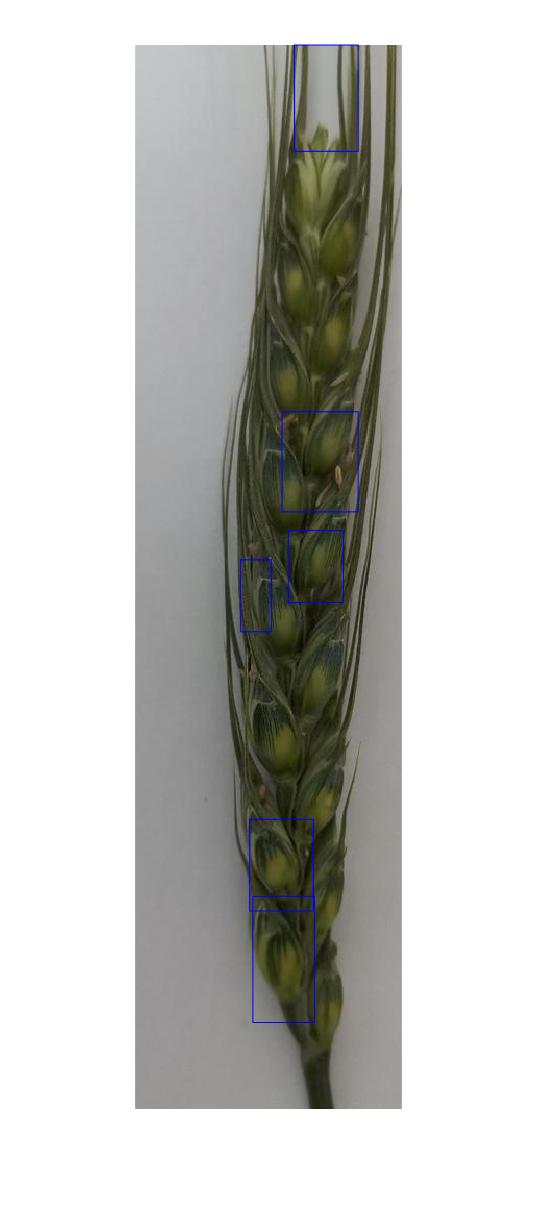

Supplement: Supplementary file 2 [file Data_Sheet_2.zip › 3. Labeling results of watershed algorithm (section Spikelet segmentation and annotation)/Shannong 25/2288b.jpg]

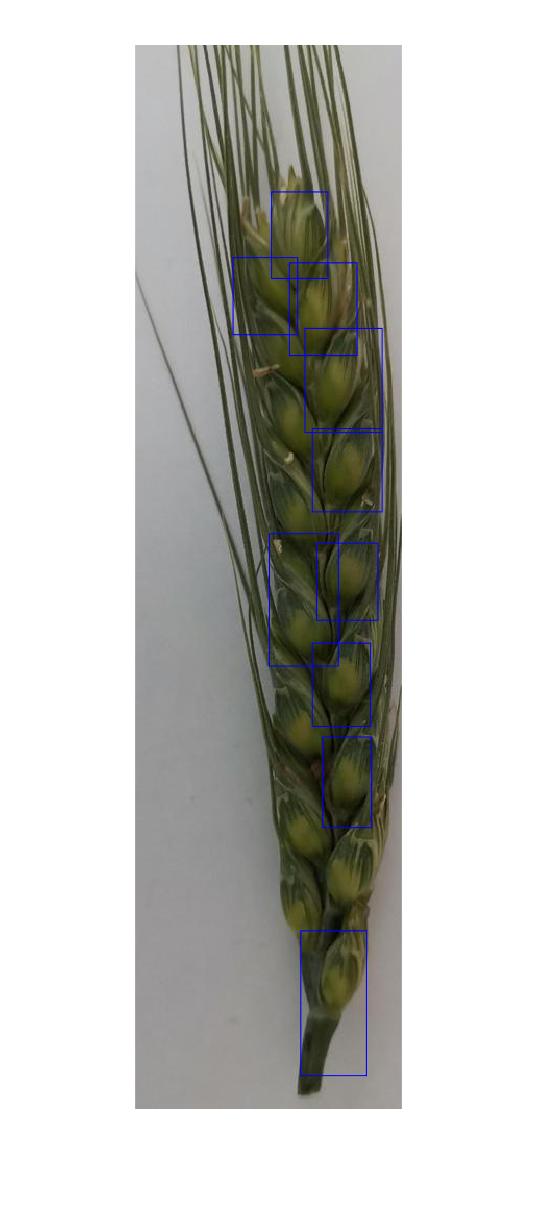

Supplement: Supplementary file 2 [file Data_Sheet_2.zip › 3. Labeling results of watershed algorithm (section Spikelet segmentation and annotation)/Shannong 25/2289b.jpg]

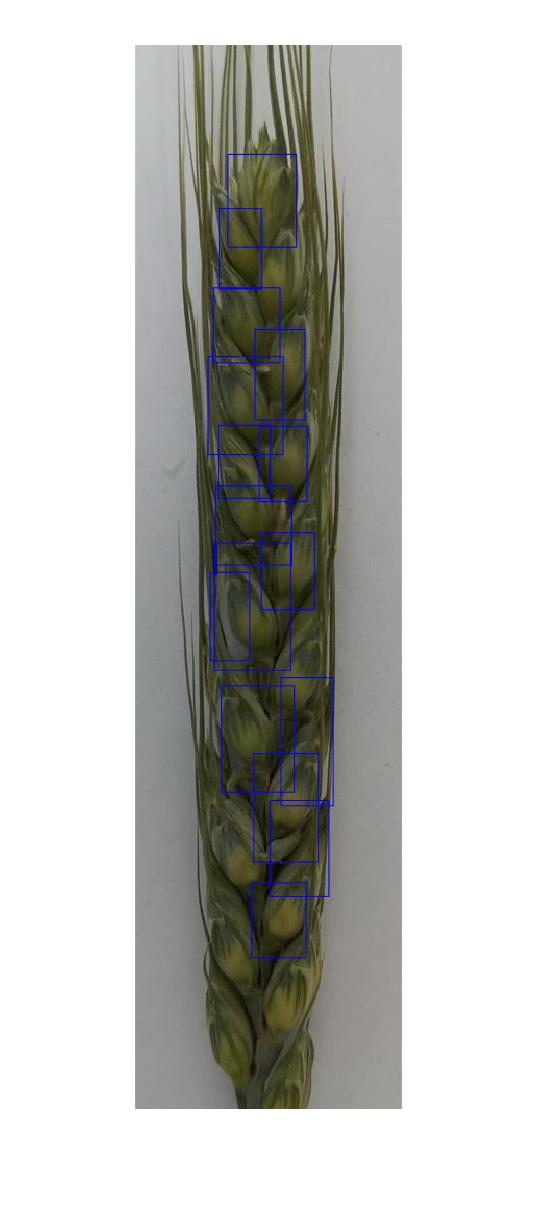

Supplement: Supplementary file 2 [file Data_Sheet_2.zip › 3. Labeling results of watershed algorithm (section Spikelet segmentation and annotation)/Shannong 25/2291b.jpg]

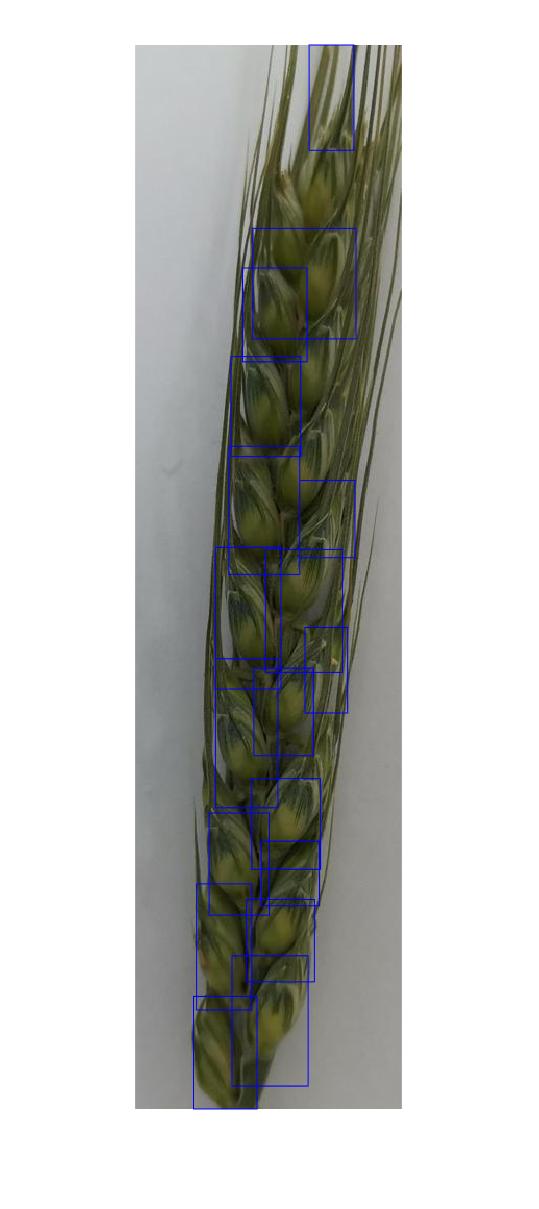

Supplement: Supplementary file 2 [file Data_Sheet_2.zip › 3. Labeling results of watershed algorithm (section Spikelet segmentation and annotation)/Shannong 25/2292b.jpg]

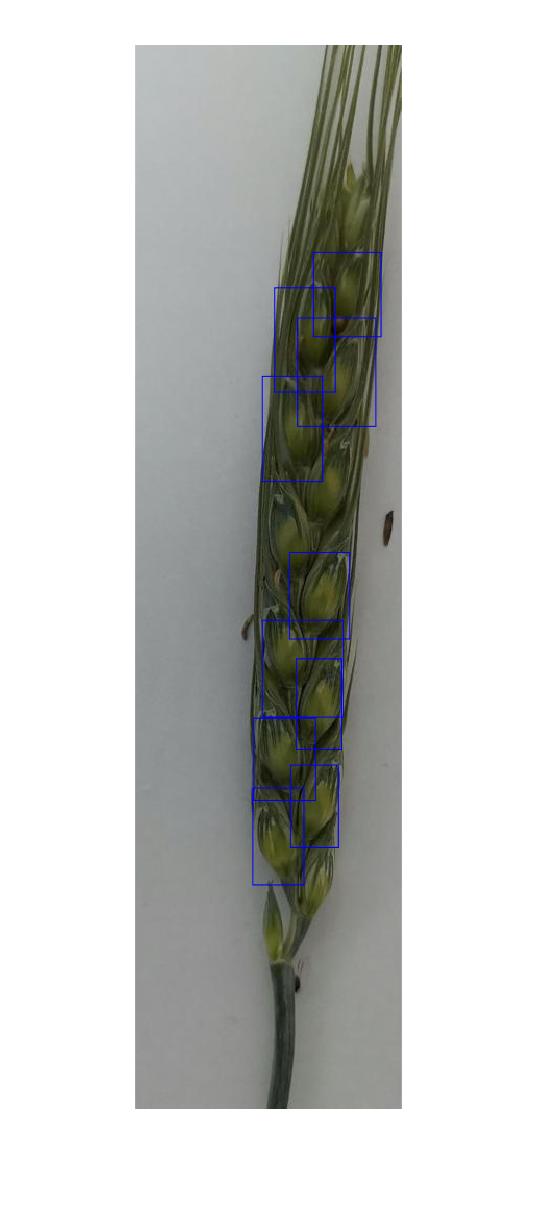

Supplement: Supplementary file 2 [file Data_Sheet_2.zip › 3. Labeling results of watershed algorithm (section Spikelet segmentation and annotation)/Shannong 25/2294b.jpg]

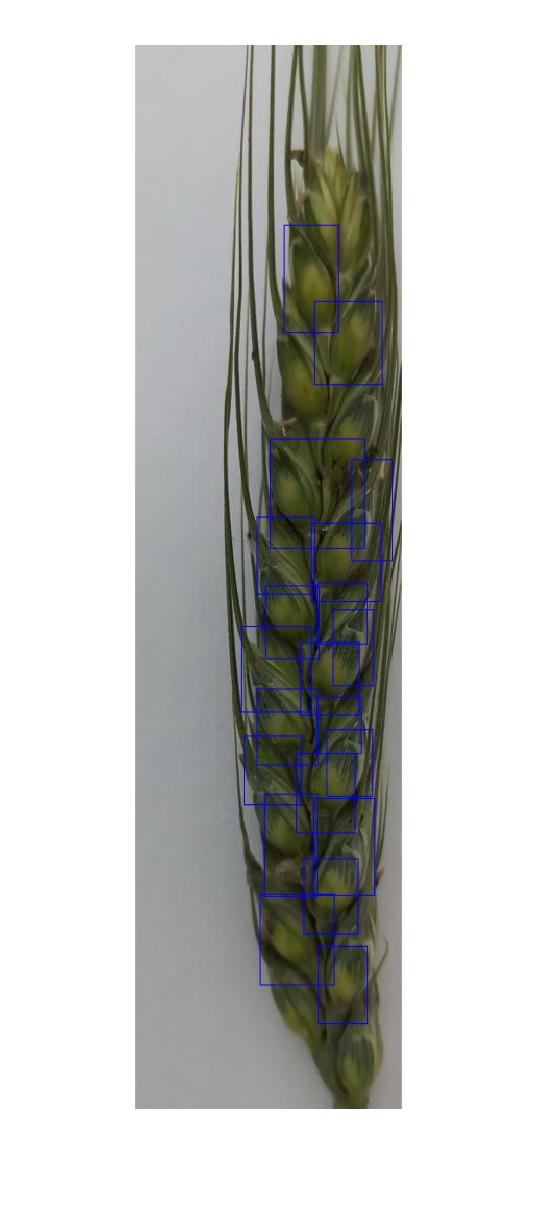

Supplement: Supplementary file 2 [file Data_Sheet_2.zip › 3. Labeling results of watershed algorithm (section Spikelet segmentation and annotation)/Shannong 25/2296b.jpg]

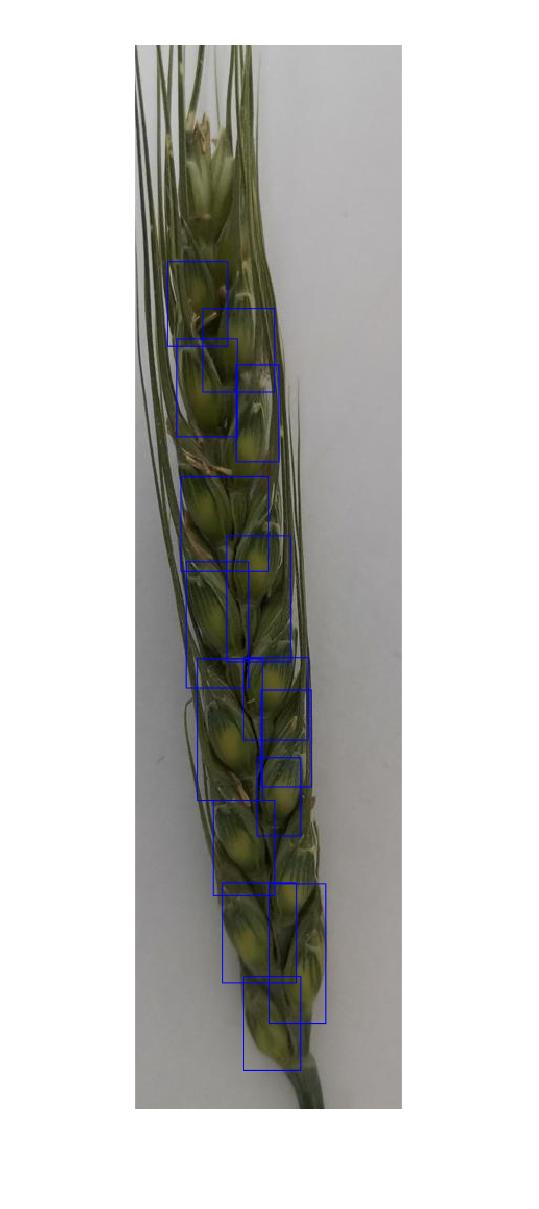

Supplement: Supplementary file 2 [file Data_Sheet_2.zip › 3. Labeling results of watershed algorithm (section Spikelet segmentation and annotation)/Shannong 25/2299b.jpg]

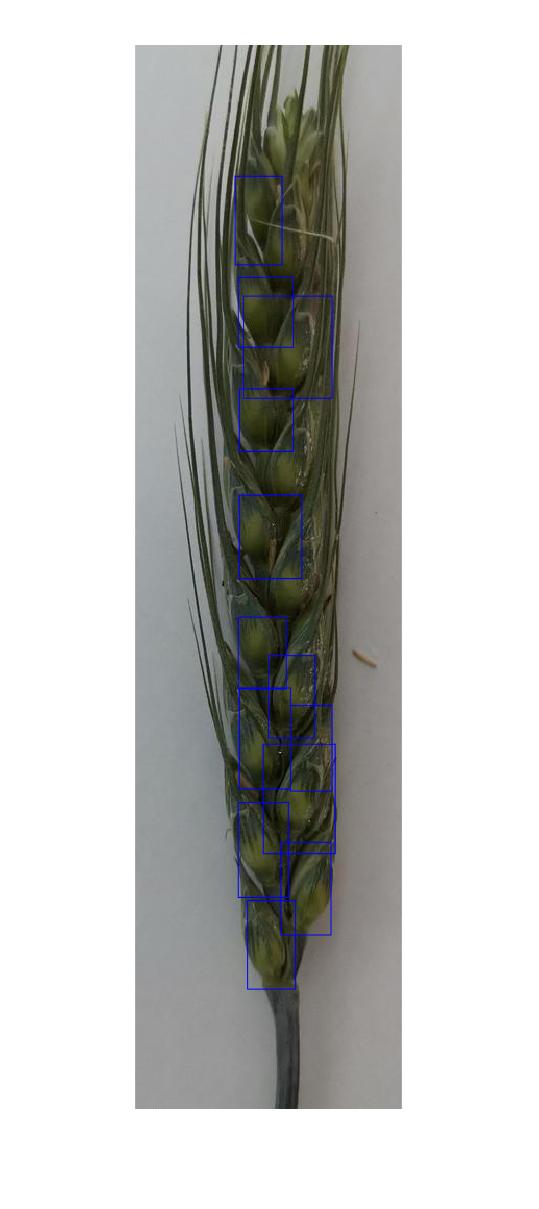

Supplement: Supplementary file 2 [file Data_Sheet_2.zip › 3. Labeling results of watershed algorithm (section Spikelet segmentation and annotation)/Shannong 25/2301b.jpg]

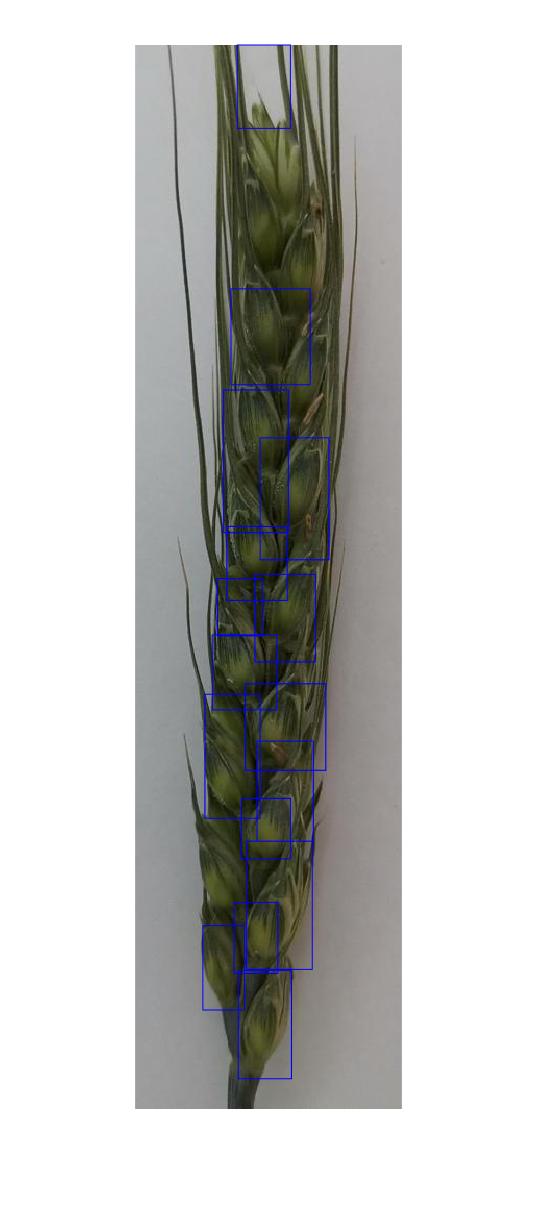

Supplement: Supplementary file 2 [file Data_Sheet_2.zip › 3. Labeling results of watershed algorithm (section Spikelet segmentation and annotation)/Shannong 25/2306b.jpg]

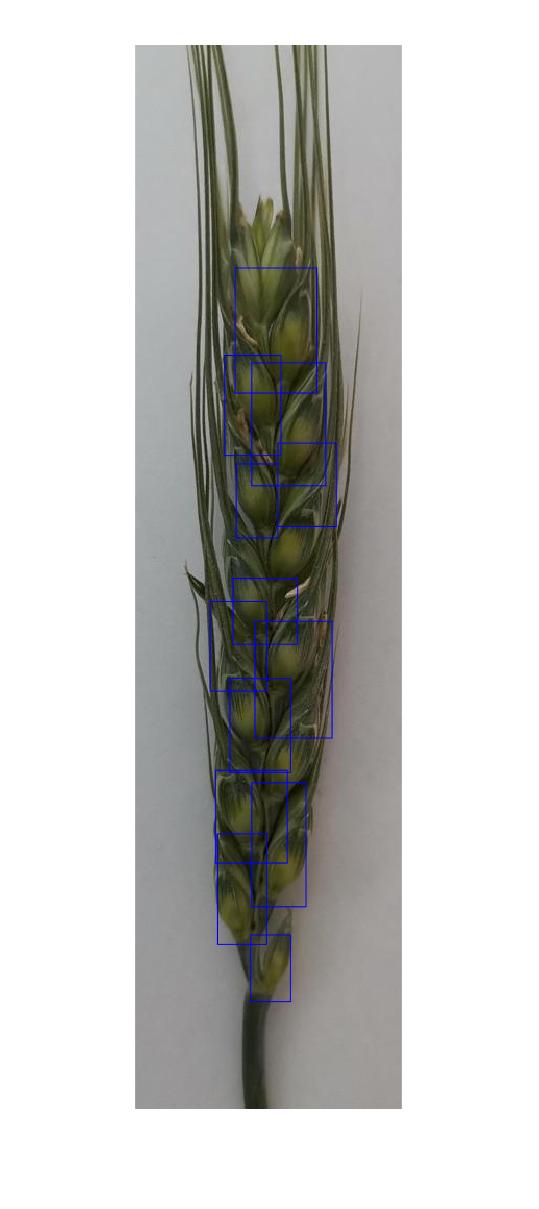

Supplement: Supplementary file 2 [file Data_Sheet_2.zip › 3. Labeling results of watershed algorithm (section Spikelet segmentation and annotation)/Shannong 25/2310b.jpg]

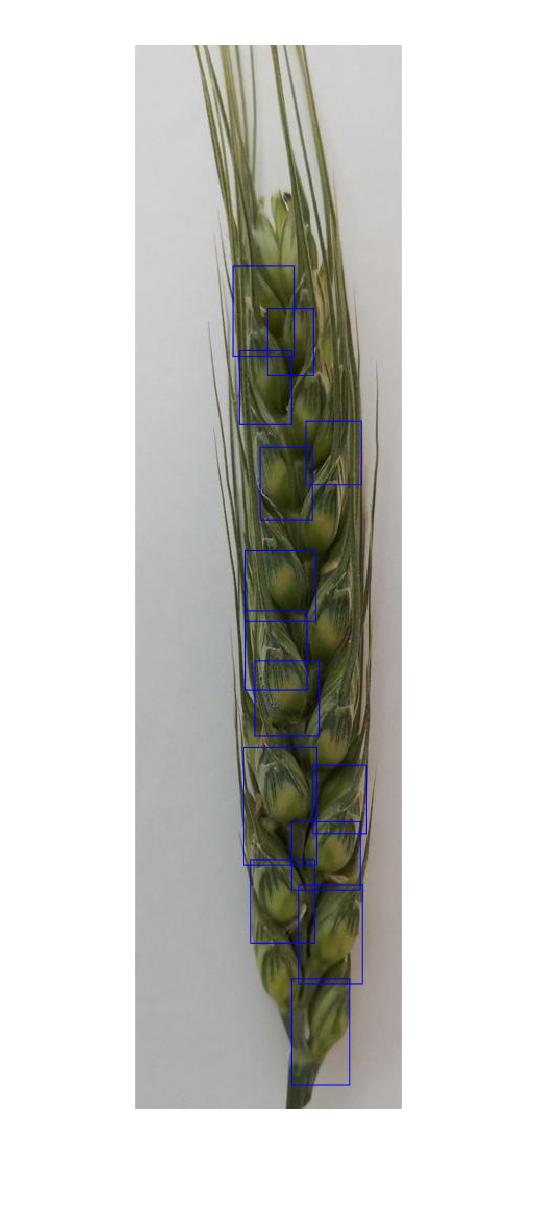

Supplement: Supplementary file 2 [file Data_Sheet_2.zip › 3. Labeling results of watershed algorithm (section Spikelet segmentation and annotation)/Shannong 25/2313b.jpg]

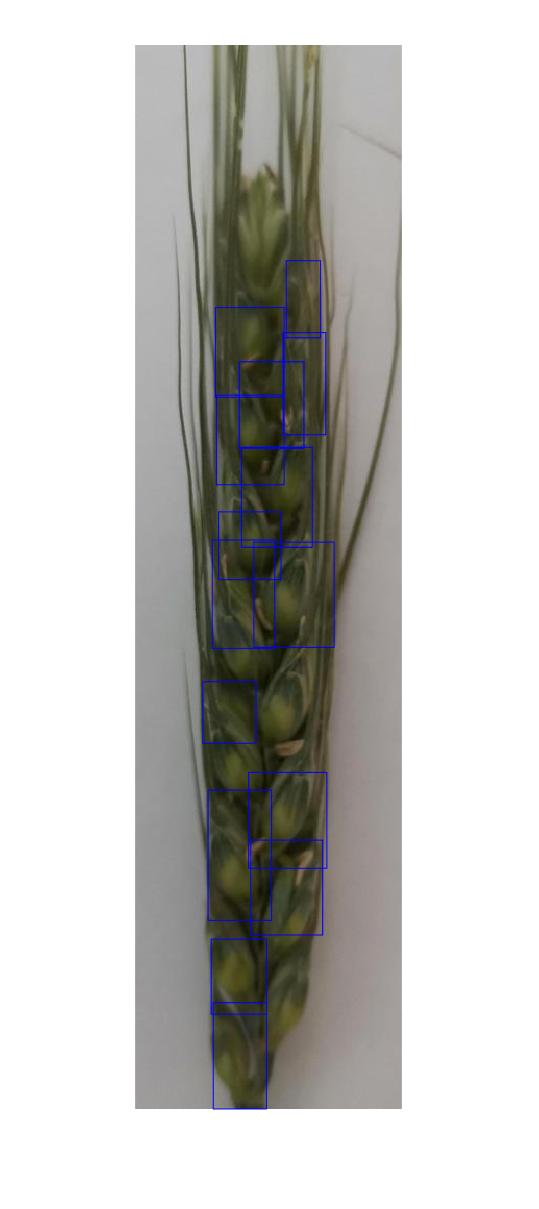

Supplement: Supplementary file 2 [file Data_Sheet_2.zip › 3. Labeling results of watershed algorithm (section Spikelet segmentation and annotation)/Shannong 25/2318b.jpg]

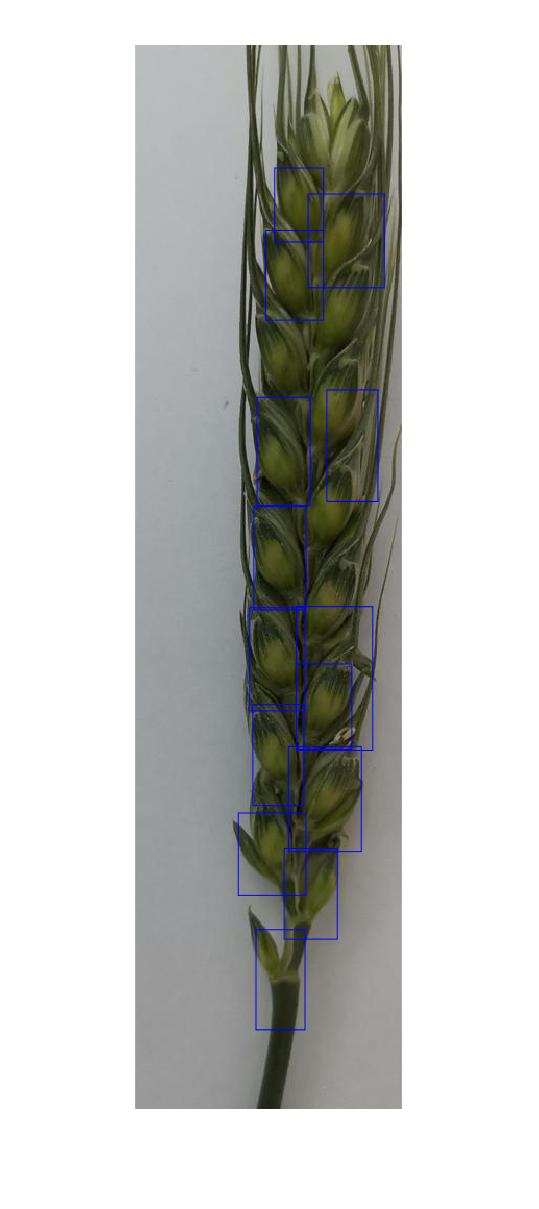

Supplement: Supplementary file 2 [file Data_Sheet_2.zip › 3. Labeling results of watershed algorithm (section Spikelet segmentation and annotation)/Shannong 25/2319b.jpg]

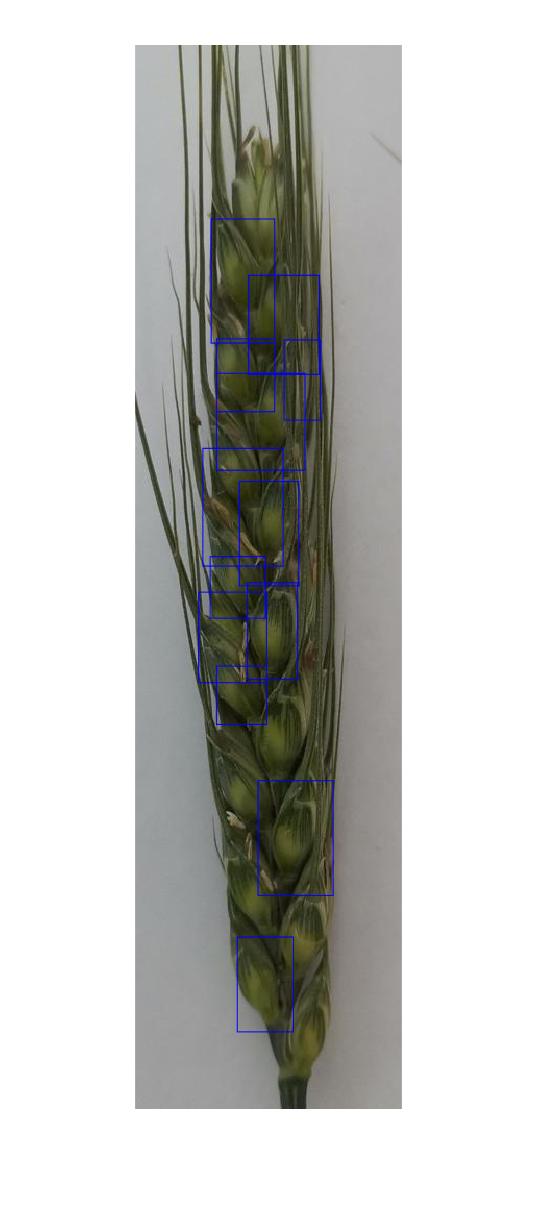

Supplement: Supplementary file 2 [file Data_Sheet_2.zip › 3. Labeling results of watershed algorithm (section Spikelet segmentation and annotation)/Shannong 25/2320b.jpg]

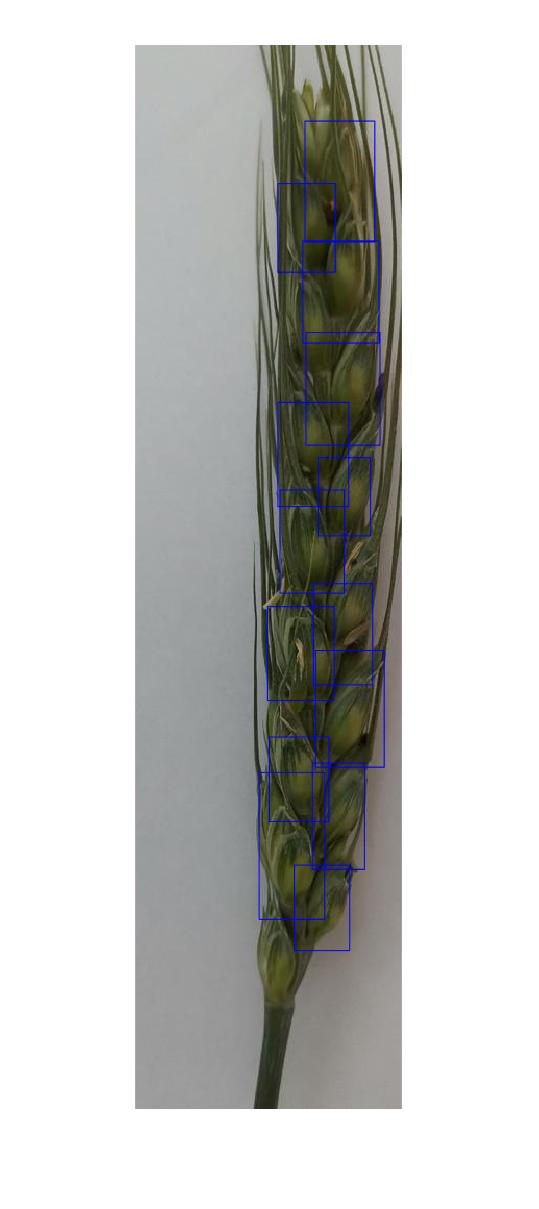

Supplement: Supplementary file 2 [file Data_Sheet_2.zip › 3. Labeling results of watershed algorithm (section Spikelet segmentation and annotation)/Shannong 25/2321b.jpg]

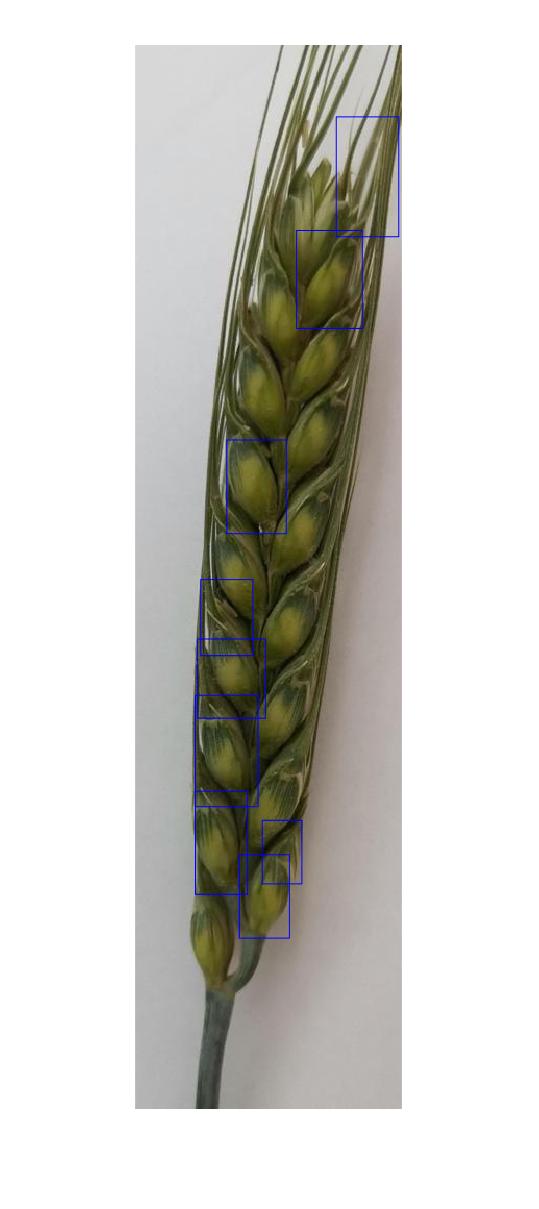

Supplement: Supplementary file 2 [file Data_Sheet_2.zip › 3. Labeling results of watershed algorithm (section Spikelet segmentation and annotation)/Shannong 25/2324b.jpg]

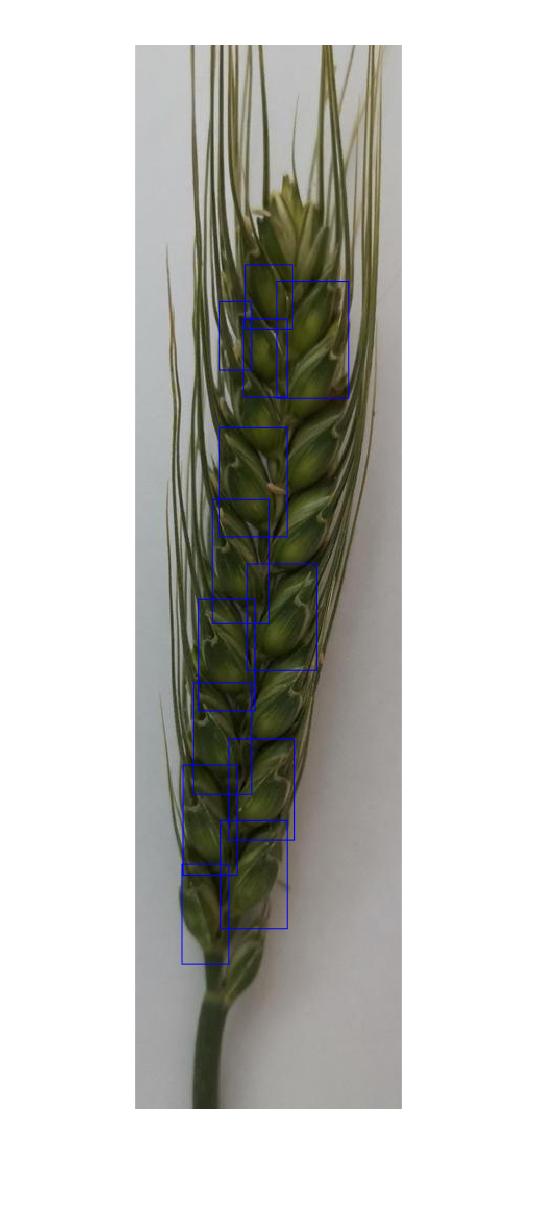

Supplement: Supplementary file 2 [file Data_Sheet_2.zip › 3. Labeling results of watershed algorithm (section Spikelet segmentation and annotation)/Shannong 25/2328b.jpg]

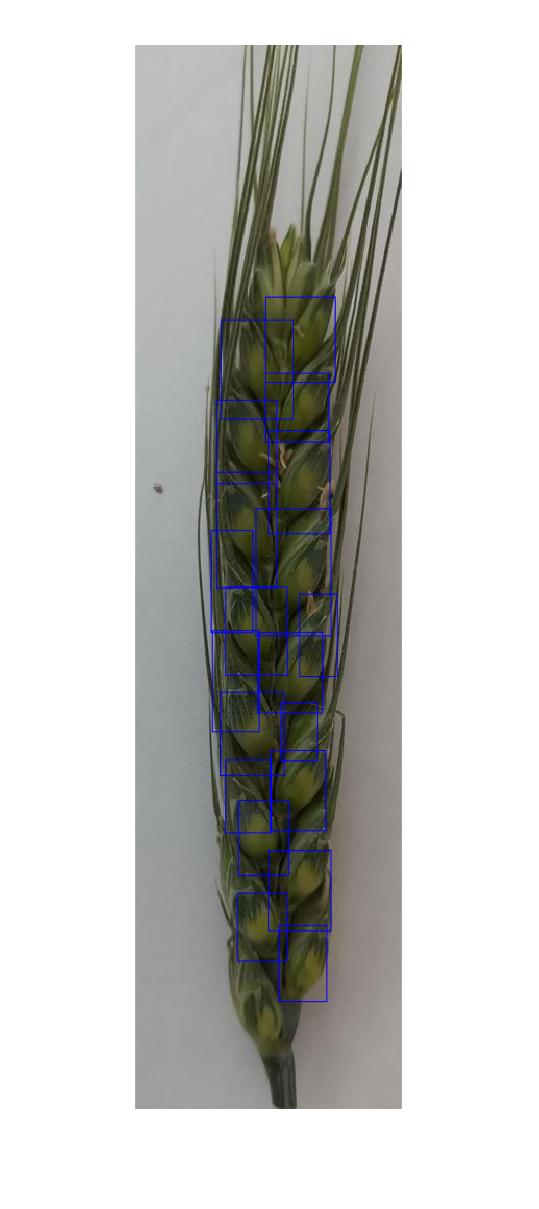

Supplement: Supplementary file 2 [file Data_Sheet_2.zip › 3. Labeling results of watershed algorithm (section Spikelet segmentation and annotation)/Shannong 25/2329b.jpg]

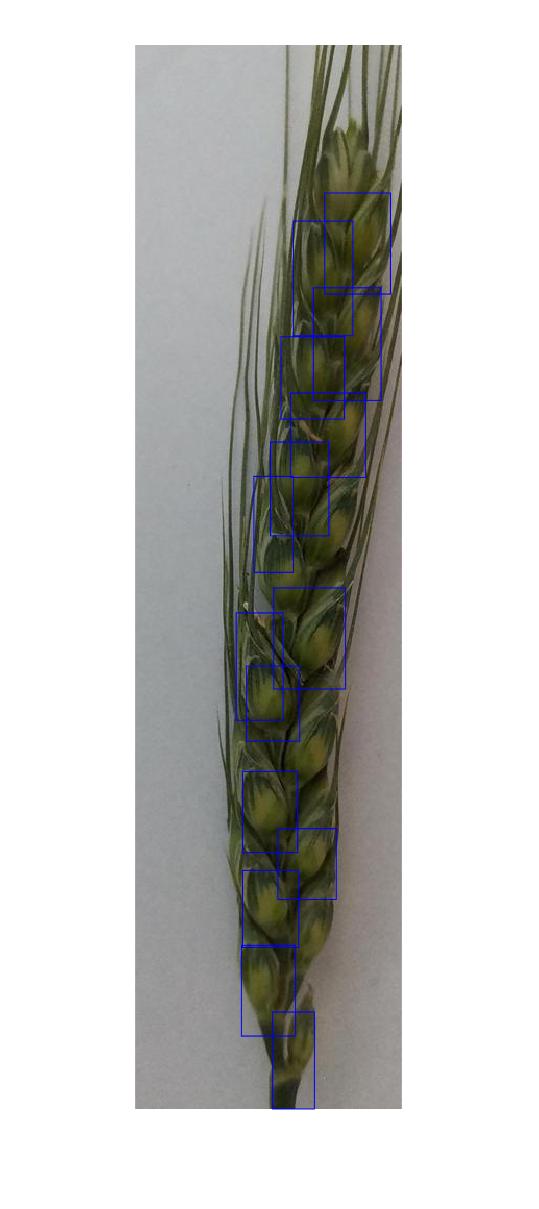

Supplement: Supplementary file 2 [file Data_Sheet_2.zip › 3. Labeling results of watershed algorithm (section Spikelet segmentation and annotation)/Shannong 25/2334b.jpg]

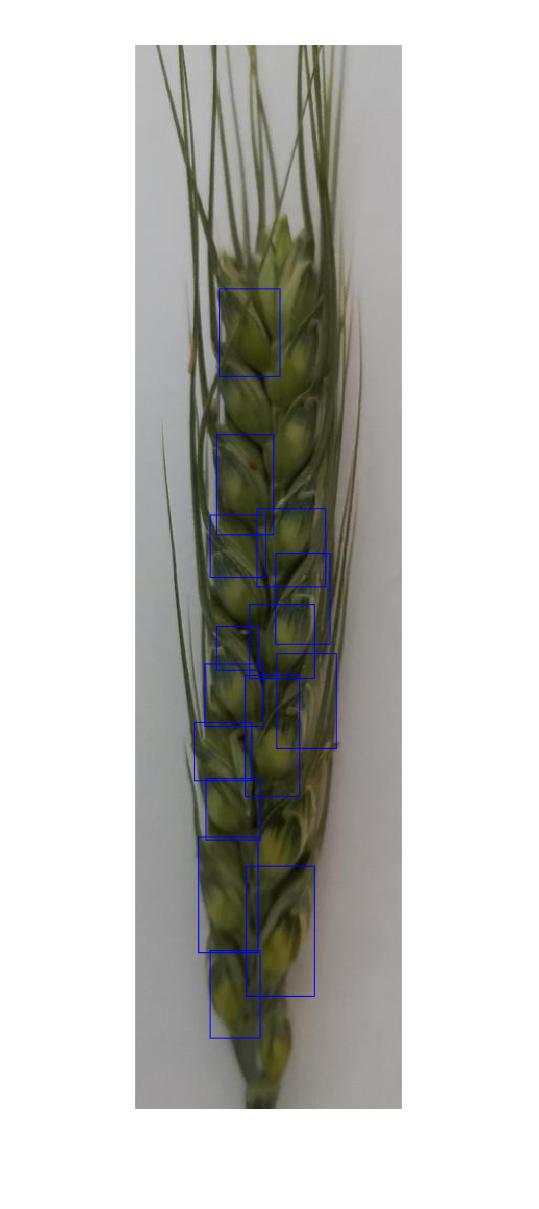

Supplement: Supplementary file 2 [file Data_Sheet_2.zip › 3. Labeling results of watershed algorithm (section Spikelet segmentation and annotation)/Shannong 25/2337b.jpg]

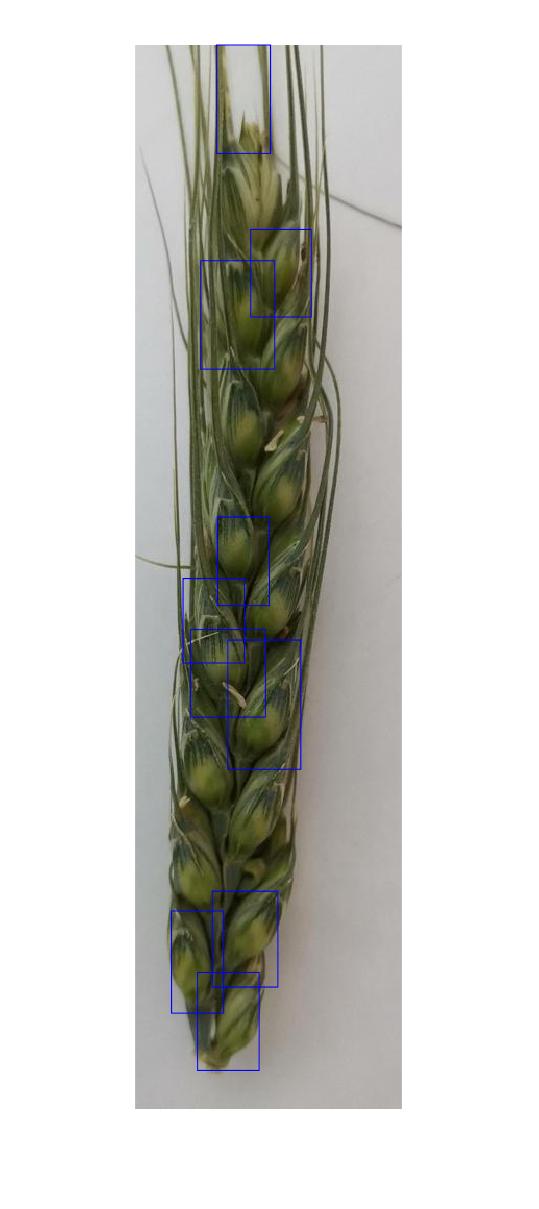

Supplement: Supplementary file 2 [file Data_Sheet_2.zip › 3. Labeling results of watershed algorithm (section Spikelet segmentation and annotation)/Shannong 25/2339b.jpg]

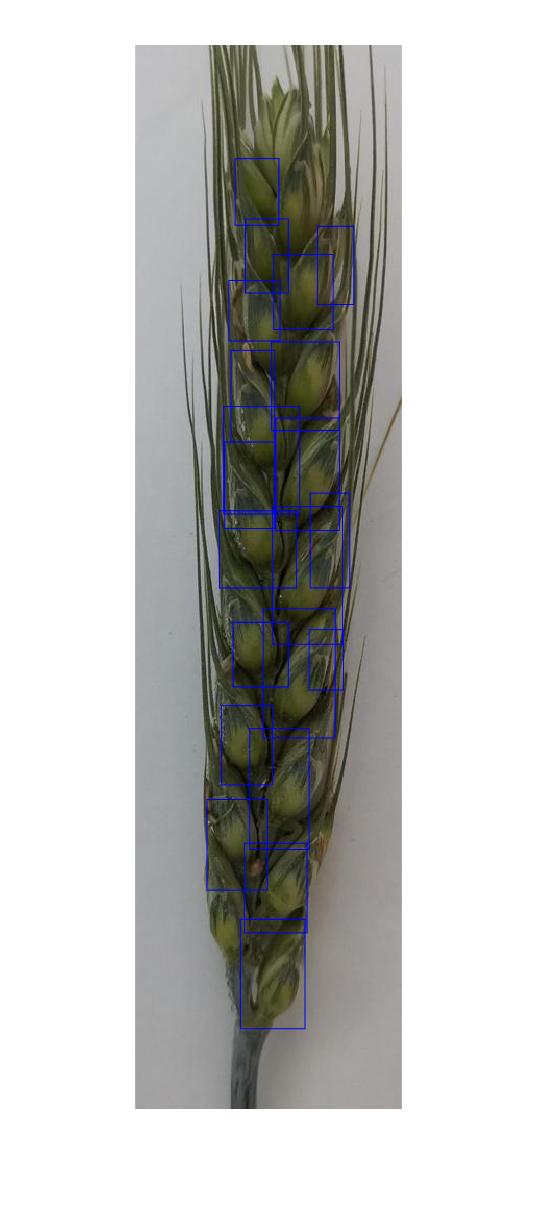

Supplement: Supplementary file 2 [file Data_Sheet_2.zip › 3. Labeling results of watershed algorithm (section Spikelet segmentation and annotation)/Shannong 25/2343b.jpg]

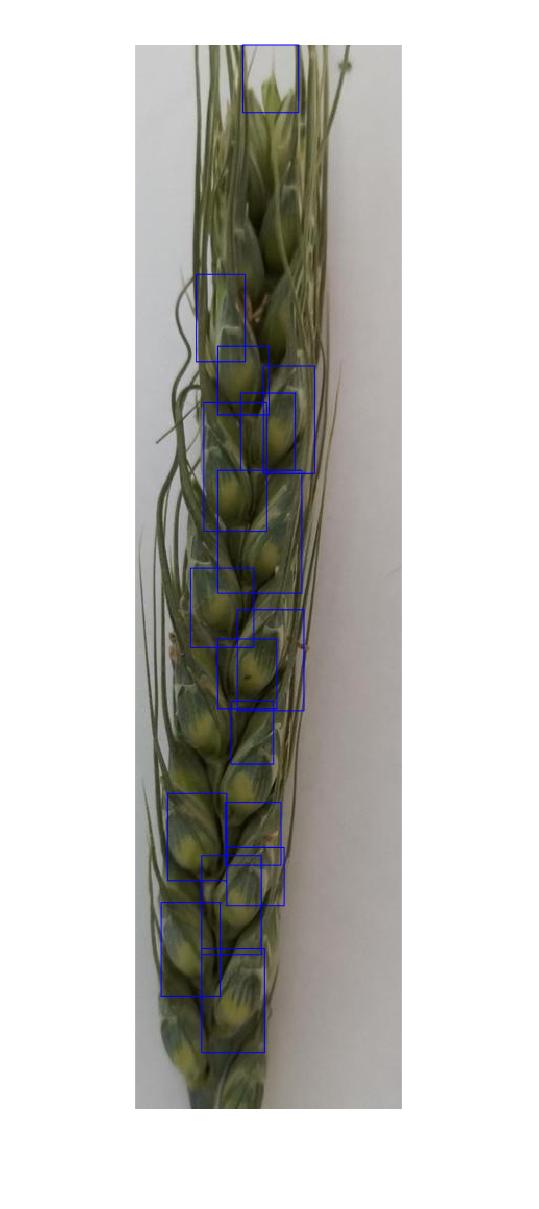

Supplement: Supplementary file 2 [file Data_Sheet_2.zip › 3. Labeling results of watershed algorithm (section Spikelet segmentation and annotation)/Shannong 25/2360b.jpg]

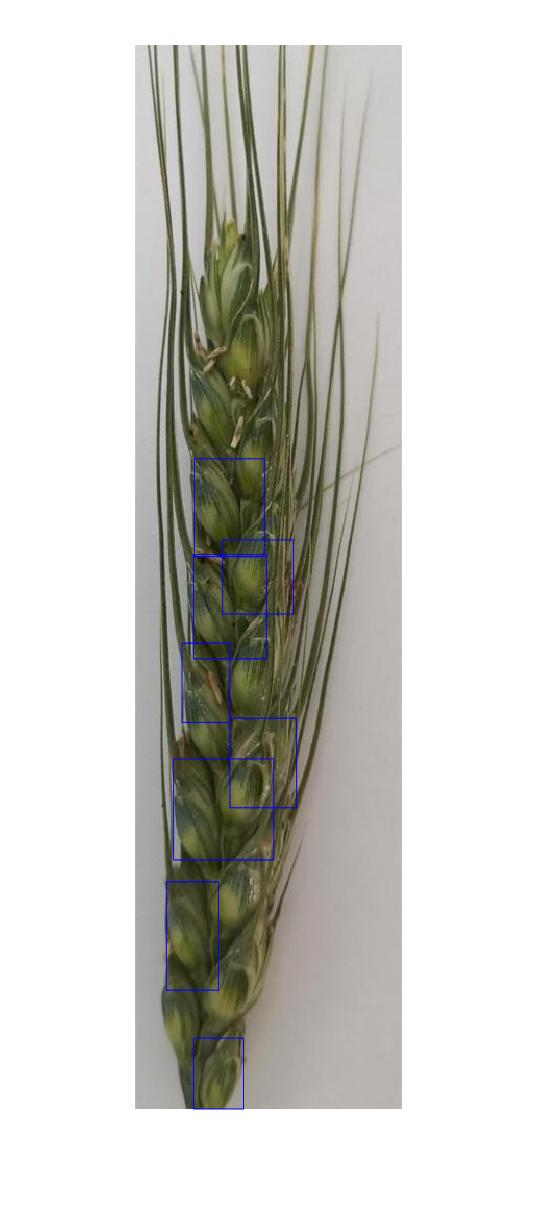

Supplement: Supplementary file 2 [file Data_Sheet_2.zip › 3. Labeling results of watershed algorithm (section Spikelet segmentation and annotation)/Shannong 25/2368b.jpg]
