# Supplementary figures and images for: Automatic Detection and Counting of Wheat Spikelet Using Semi-Automatic Labeling and Deep Learning (part 3 of 8)
Source: Front Plant Sci. 2022 May 30;13:872555. doi: 10.3389/fpls.2022.872555 (PMC9189412; doi:10.3389/fpls.2022.872555)

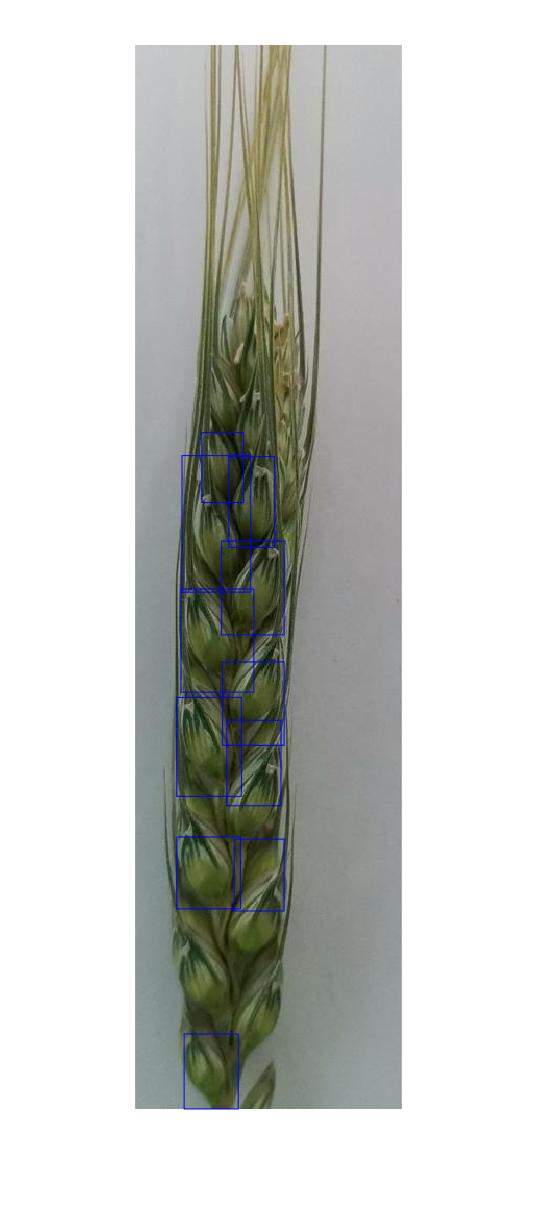

Supplement: Supplementary file 2 [file Data_Sheet_2.zip › 3. Labeling results of watershed algorithm (section Spikelet segmentation and annotation)/Liangxing 99/3005b.jpg]

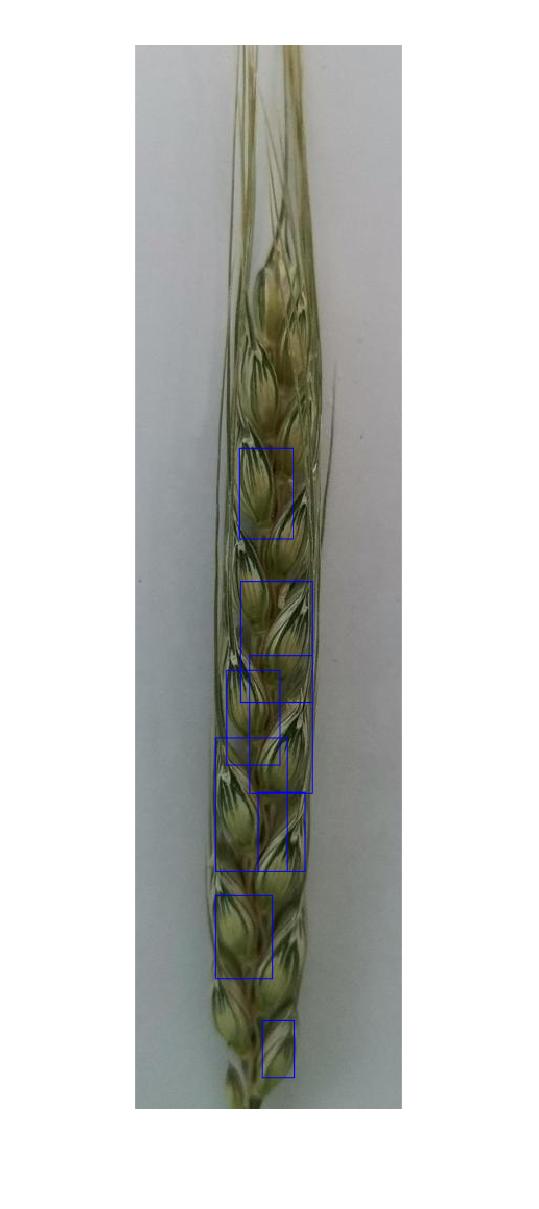

Supplement: Supplementary file 2 [file Data_Sheet_2.zip › 3. Labeling results of watershed algorithm (section Spikelet segmentation and annotation)/Liangxing 99/3009b.jpg]

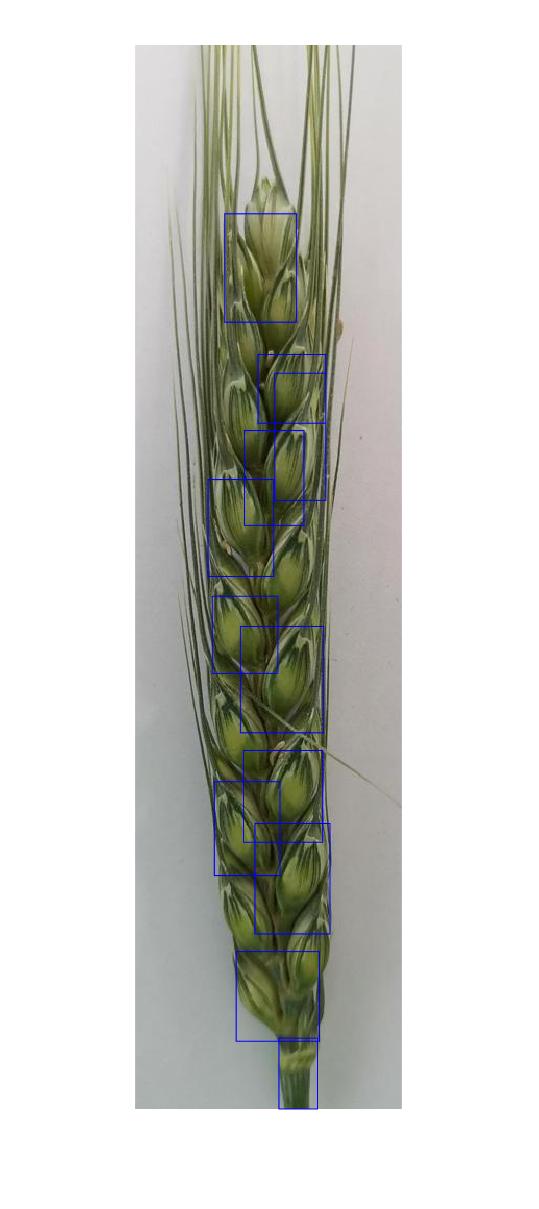

Supplement: Supplementary file 2 [file Data_Sheet_2.zip › 3. Labeling results of watershed algorithm (section Spikelet segmentation and annotation)/Liangxing 99/3010b.jpg]

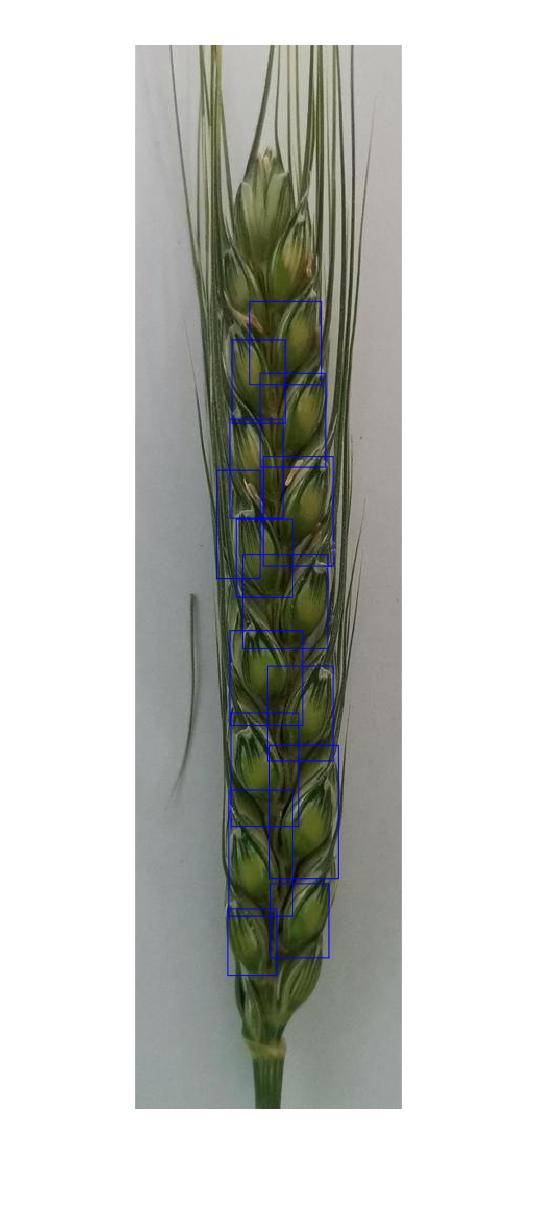

Supplement: Supplementary file 2 [file Data_Sheet_2.zip › 3. Labeling results of watershed algorithm (section Spikelet segmentation and annotation)/Liangxing 99/3011b.jpg]

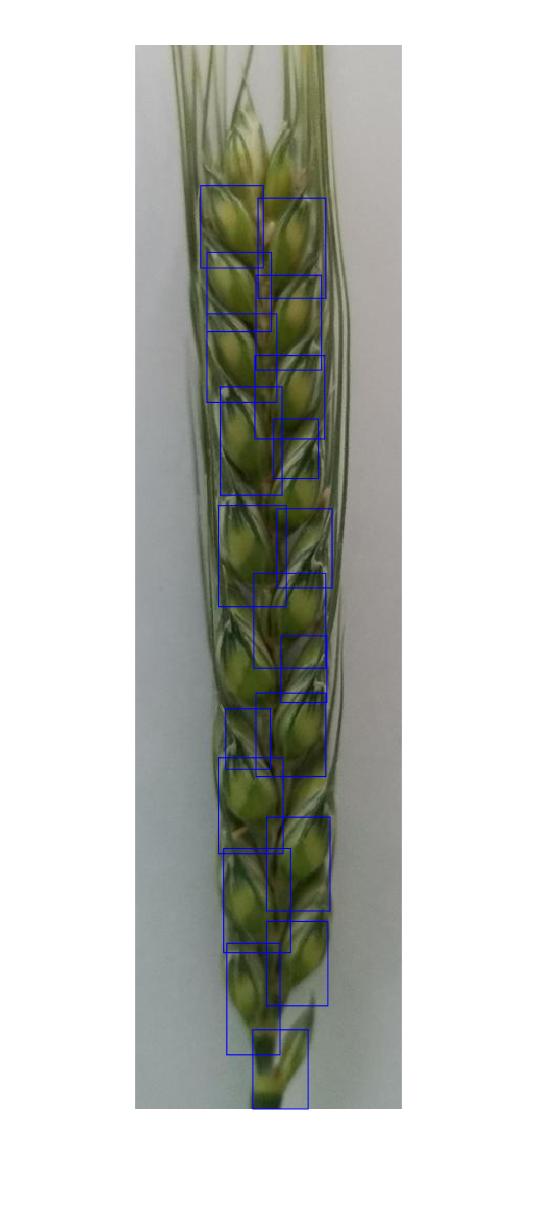

Supplement: Supplementary file 2 [file Data_Sheet_2.zip › 3. Labeling results of watershed algorithm (section Spikelet segmentation and annotation)/Liangxing 99/3014b.jpg]

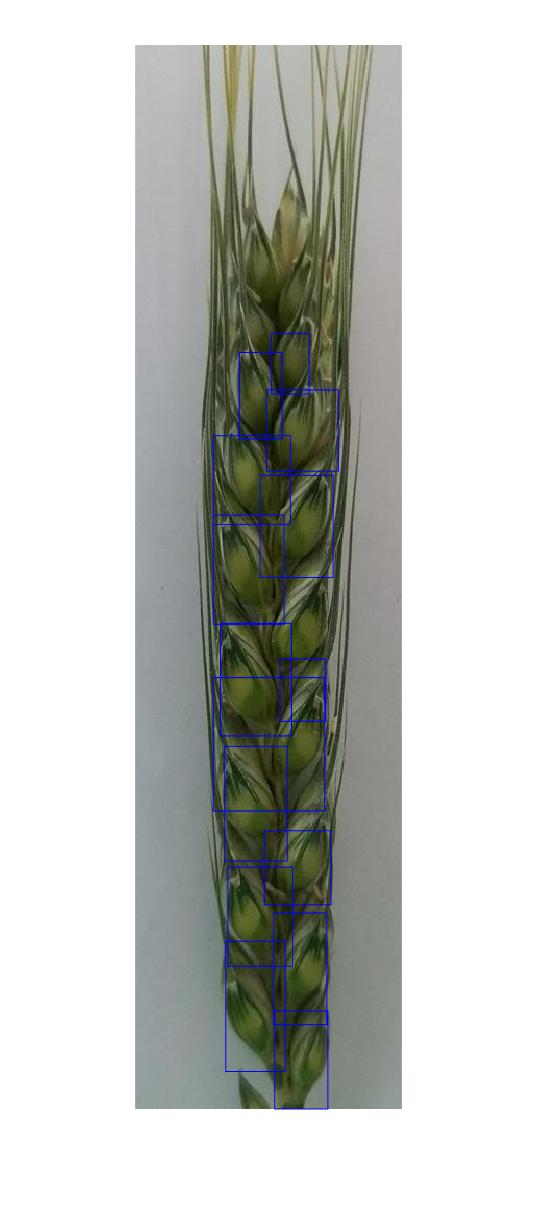

Supplement: Supplementary file 2 [file Data_Sheet_2.zip › 3. Labeling results of watershed algorithm (section Spikelet segmentation and annotation)/Liangxing 99/3015b.jpg]

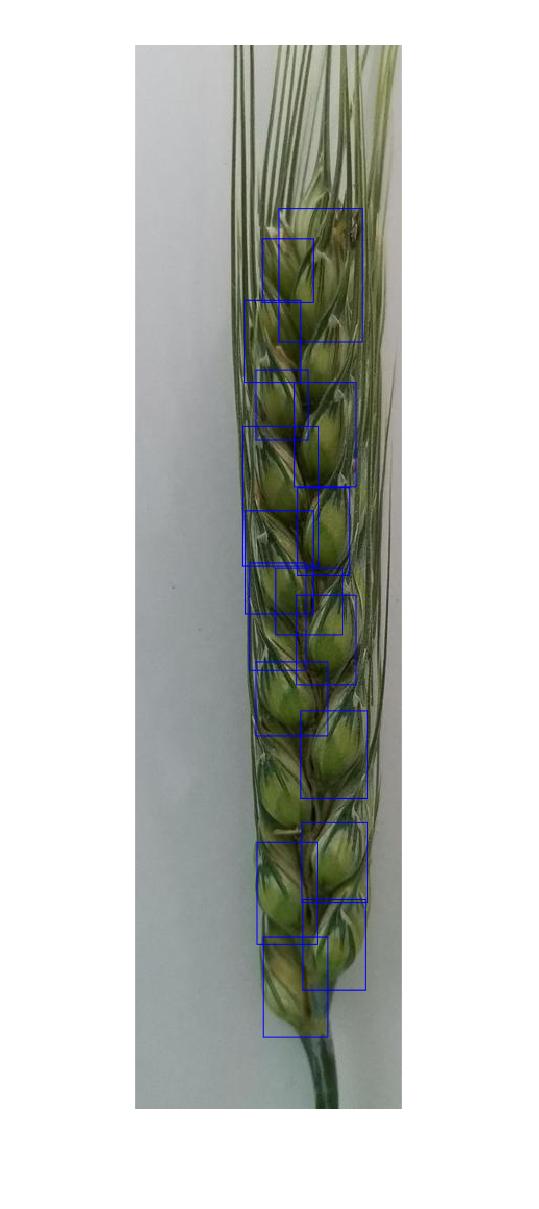

Supplement: Supplementary file 2 [file Data_Sheet_2.zip › 3. Labeling results of watershed algorithm (section Spikelet segmentation and annotation)/Liangxing 99/3018b.jpg]

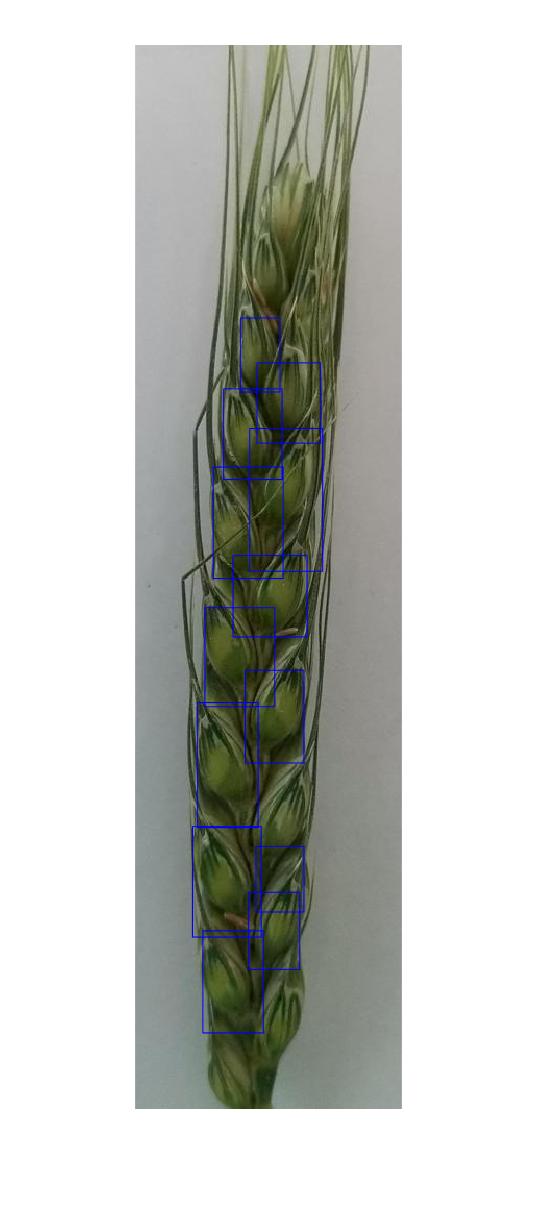

Supplement: Supplementary file 2 [file Data_Sheet_2.zip › 3. Labeling results of watershed algorithm (section Spikelet segmentation and annotation)/Liangxing 99/3020b.jpg]

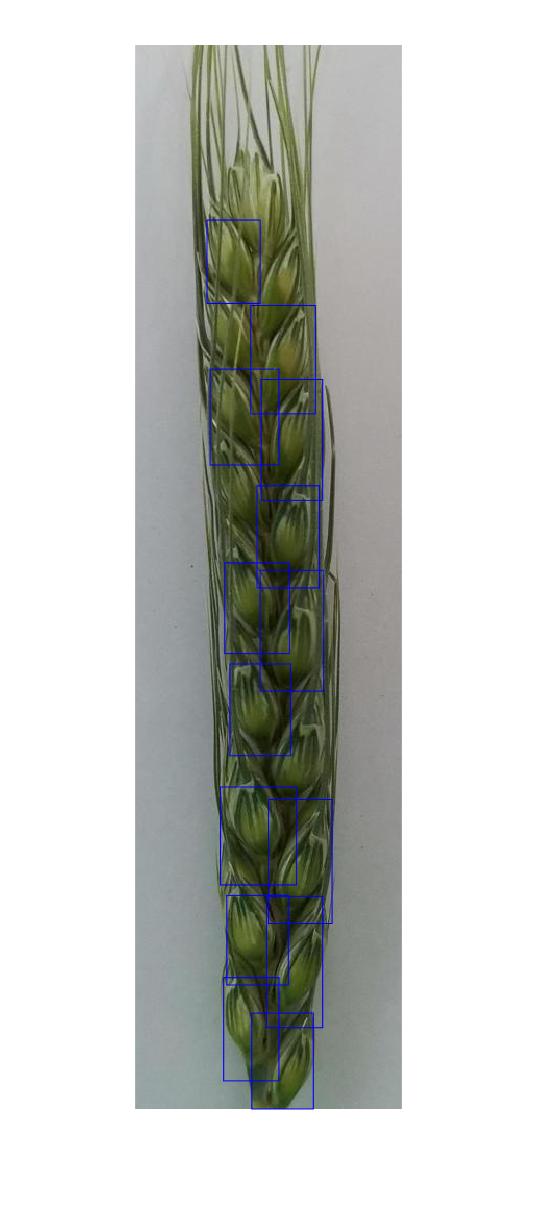

Supplement: Supplementary file 2 [file Data_Sheet_2.zip › 3. Labeling results of watershed algorithm (section Spikelet segmentation and annotation)/Liangxing 99/3021b.jpg]

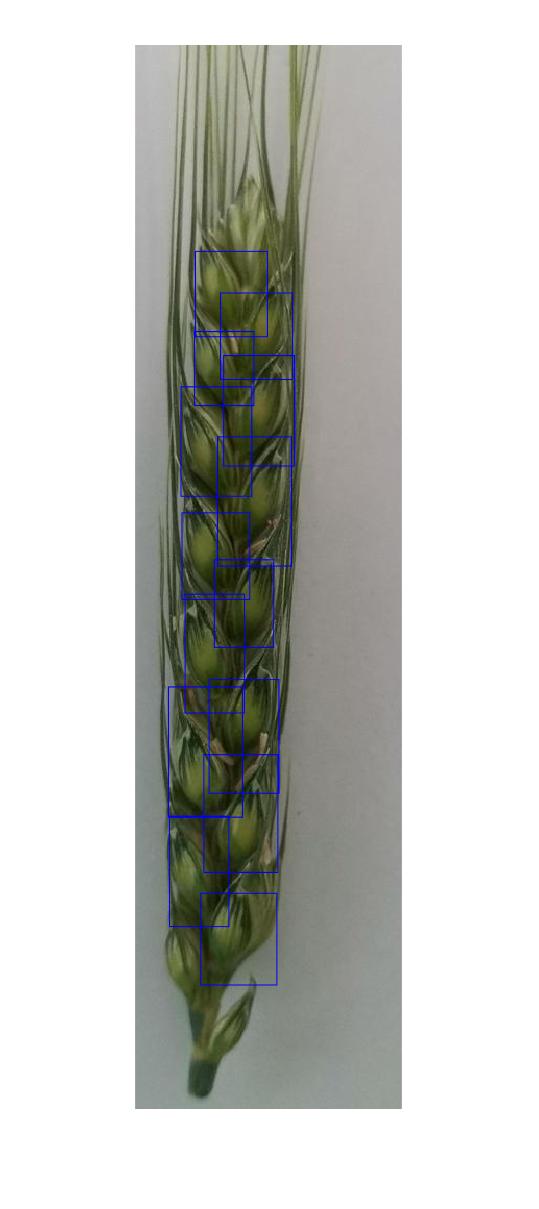

Supplement: Supplementary file 2 [file Data_Sheet_2.zip › 3. Labeling results of watershed algorithm (section Spikelet segmentation and annotation)/Liangxing 99/3022b.jpg]

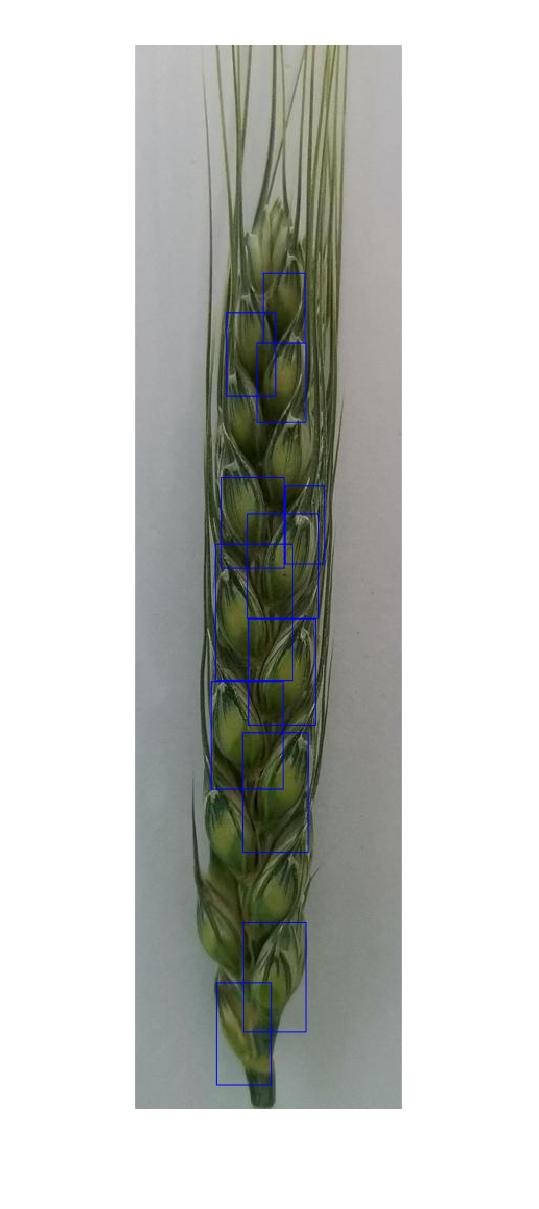

Supplement: Supplementary file 2 [file Data_Sheet_2.zip › 3. Labeling results of watershed algorithm (section Spikelet segmentation and annotation)/Liangxing 99/3023b.jpg]

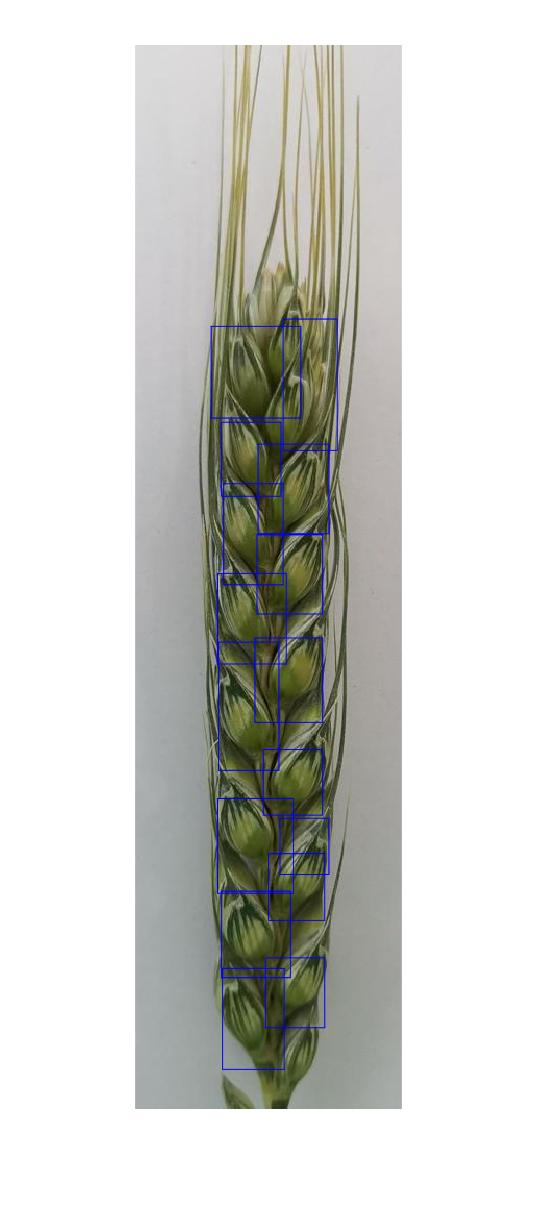

Supplement: Supplementary file 2 [file Data_Sheet_2.zip › 3. Labeling results of watershed algorithm (section Spikelet segmentation and annotation)/Liangxing 99/3027b.jpg]

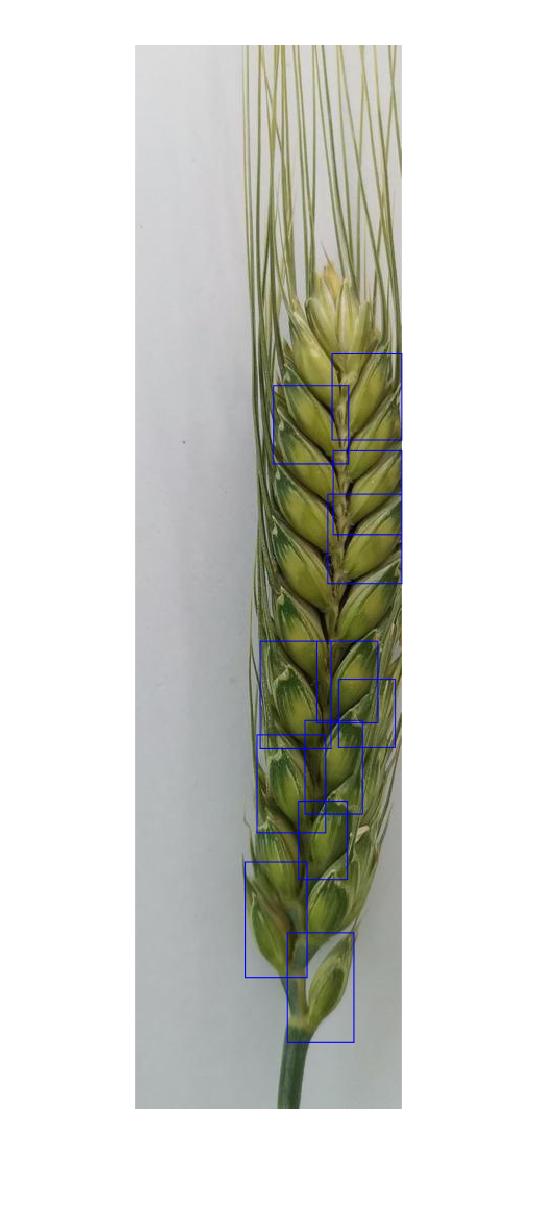

Supplement: Supplementary file 2 [file Data_Sheet_2.zip › 3. Labeling results of watershed algorithm (section Spikelet segmentation and annotation)/Liangxing 99/3030b.jpg]

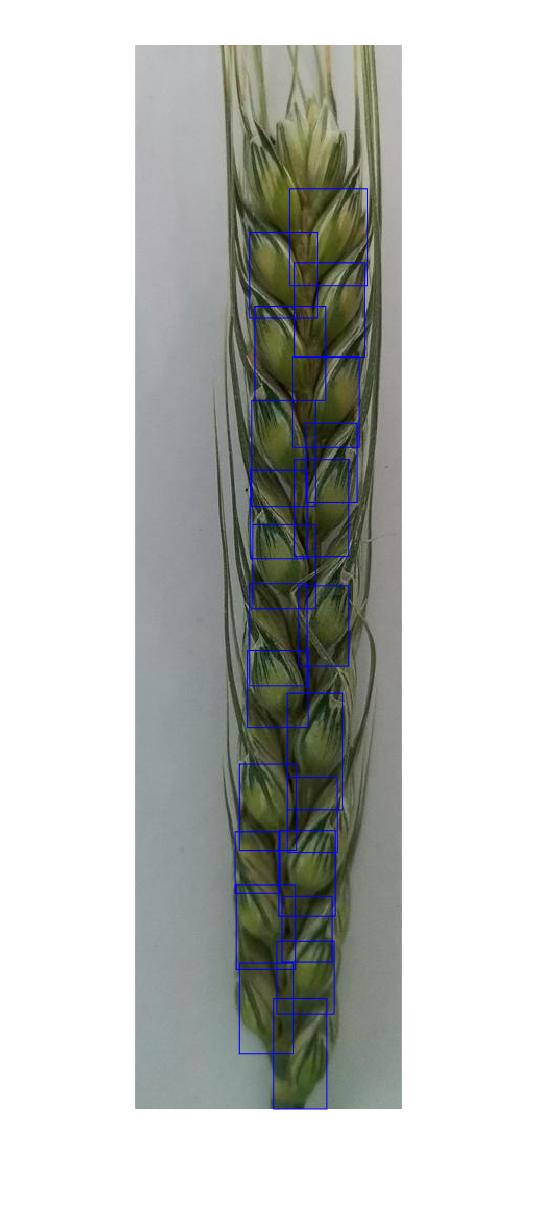

Supplement: Supplementary file 2 [file Data_Sheet_2.zip › 3. Labeling results of watershed algorithm (section Spikelet segmentation and annotation)/Liangxing 99/3033b.jpg]

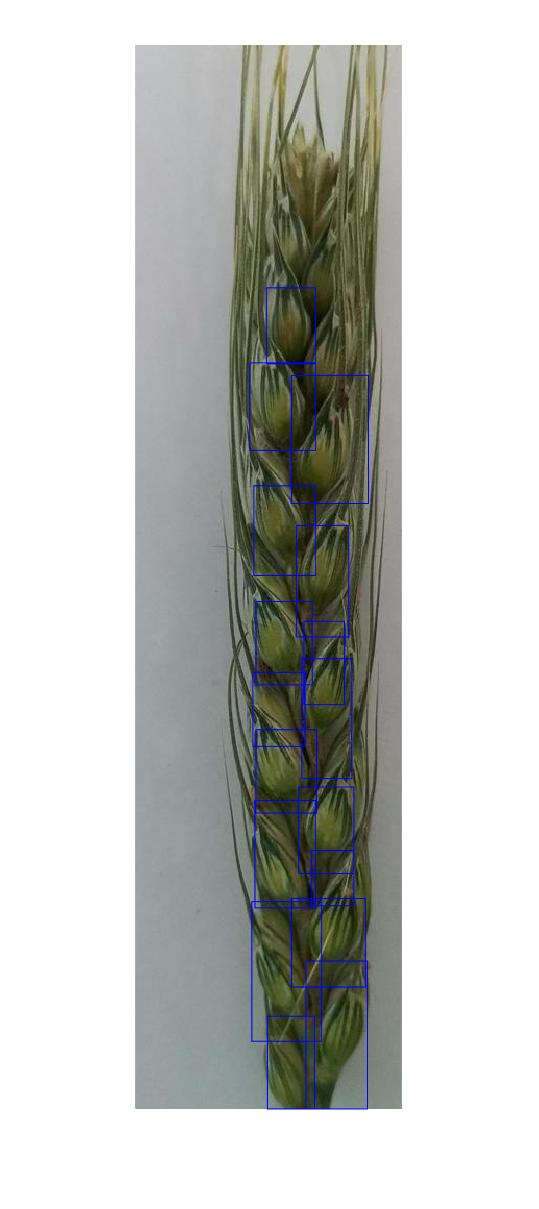

Supplement: Supplementary file 2 [file Data_Sheet_2.zip › 3. Labeling results of watershed algorithm (section Spikelet segmentation and annotation)/Liangxing 99/3034b.jpg]

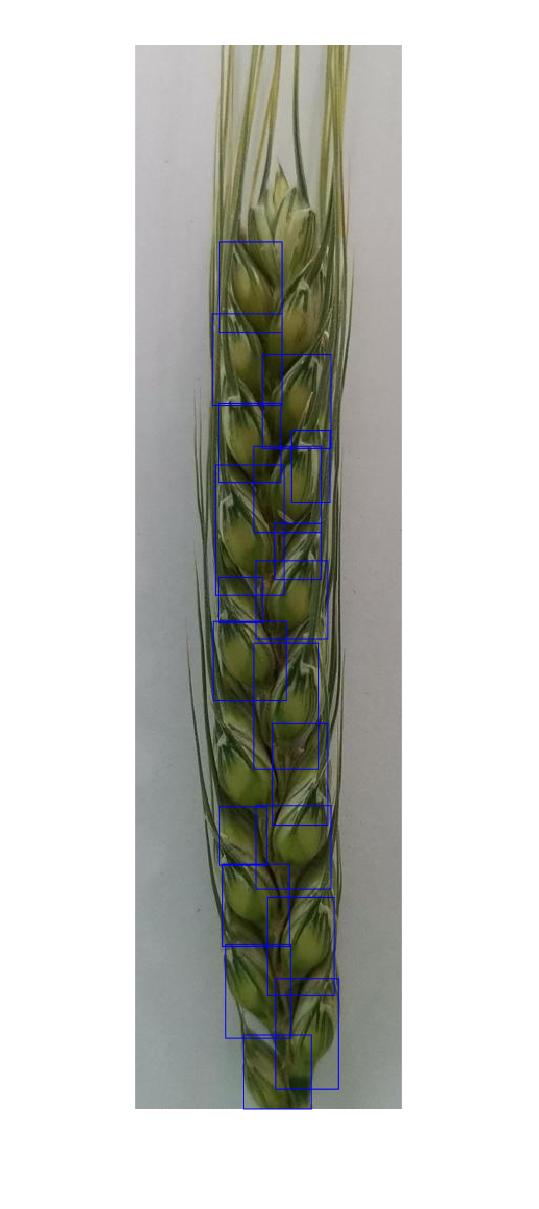

Supplement: Supplementary file 2 [file Data_Sheet_2.zip › 3. Labeling results of watershed algorithm (section Spikelet segmentation and annotation)/Liangxing 99/3036b.jpg]

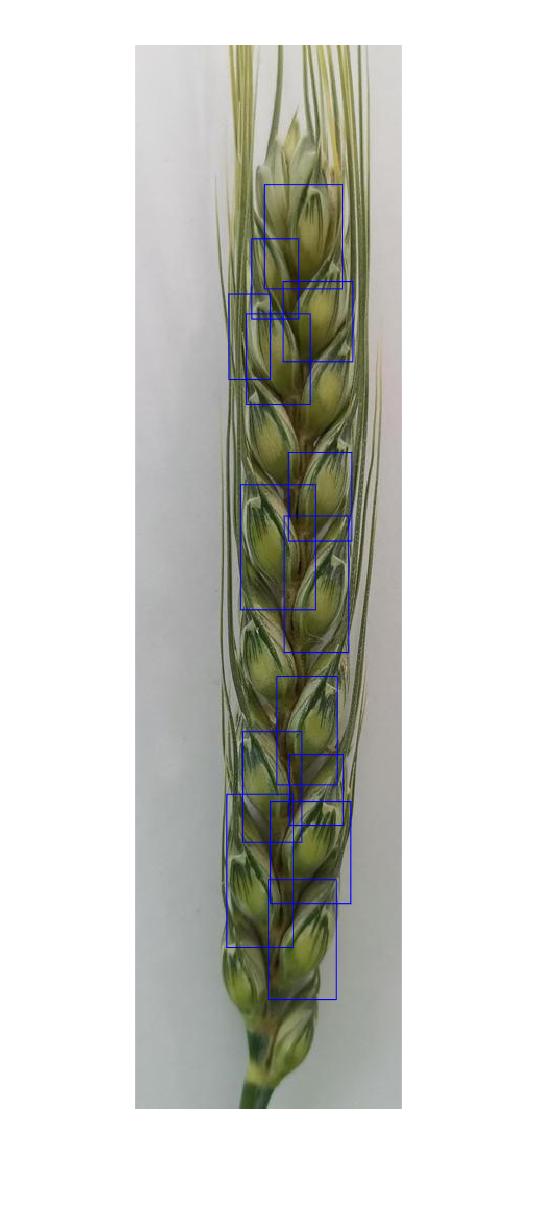

Supplement: Supplementary file 2 [file Data_Sheet_2.zip › 3. Labeling results of watershed algorithm (section Spikelet segmentation and annotation)/Liangxing 99/3037b.jpg]

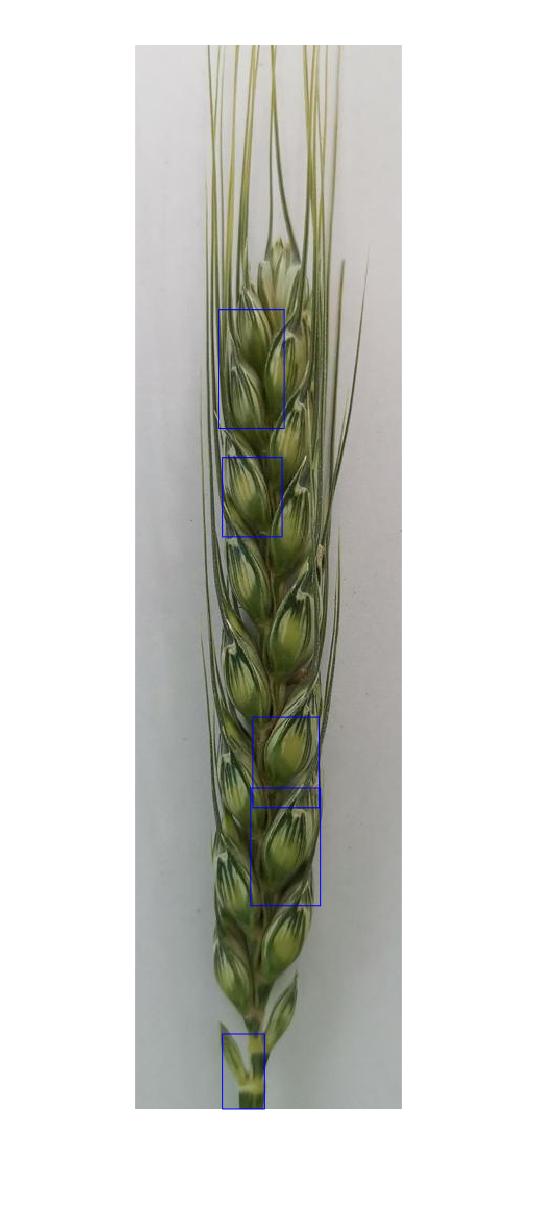

Supplement: Supplementary file 2 [file Data_Sheet_2.zip › 3. Labeling results of watershed algorithm (section Spikelet segmentation and annotation)/Liangxing 99/3039b.jpg]

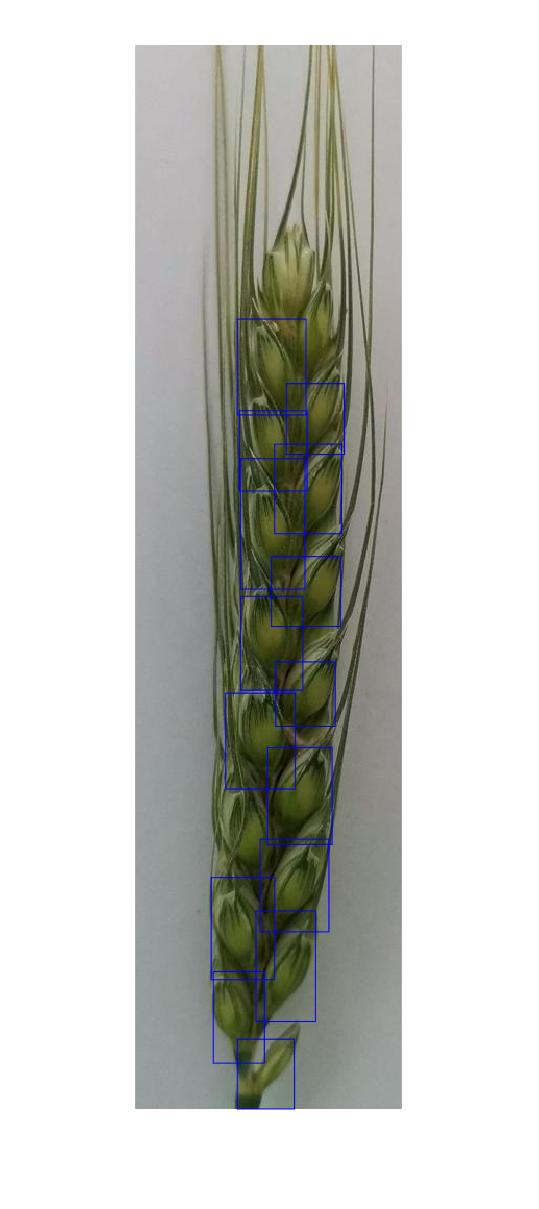

Supplement: Supplementary file 2 [file Data_Sheet_2.zip › 3. Labeling results of watershed algorithm (section Spikelet segmentation and annotation)/Liangxing 99/3040b.jpg]

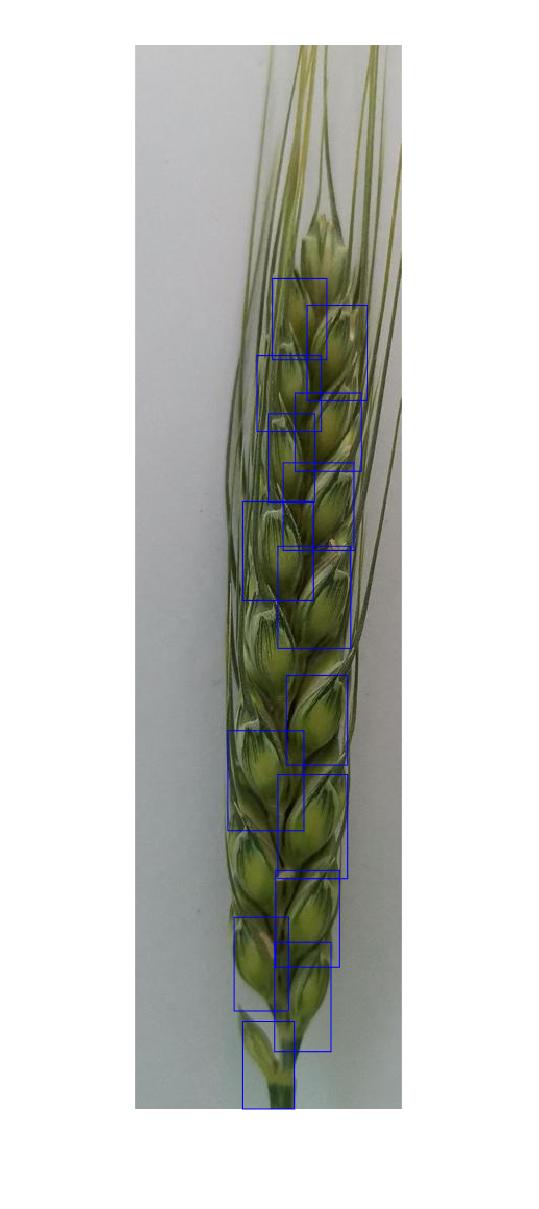

Supplement: Supplementary file 2 [file Data_Sheet_2.zip › 3. Labeling results of watershed algorithm (section Spikelet segmentation and annotation)/Liangxing 99/3041b.jpg]

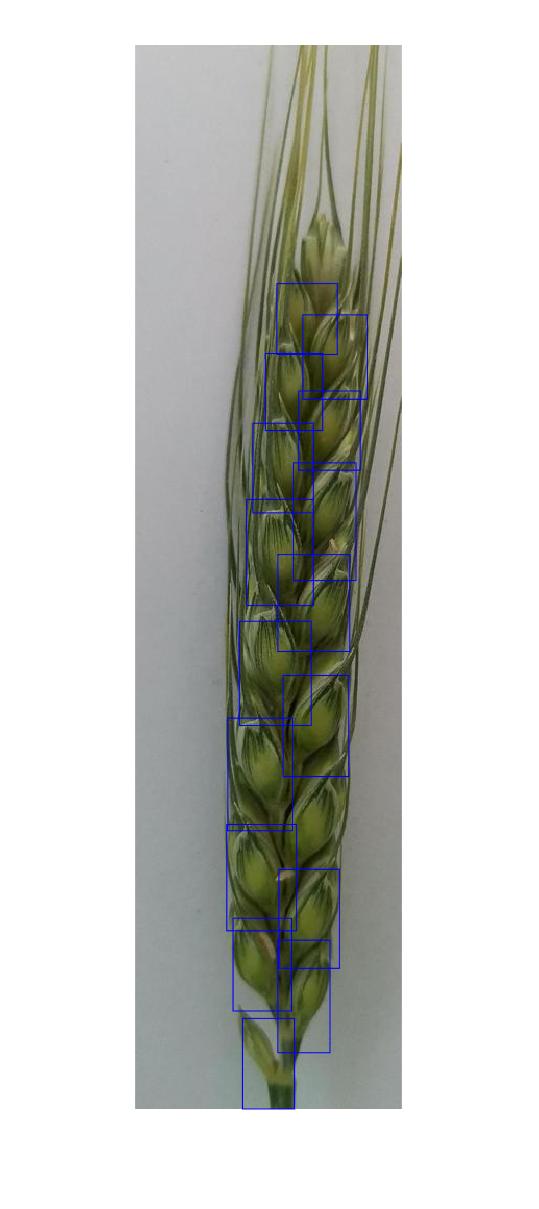

Supplement: Supplementary file 2 [file Data_Sheet_2.zip › 3. Labeling results of watershed algorithm (section Spikelet segmentation and annotation)/Liangxing 99/3042b.jpg]

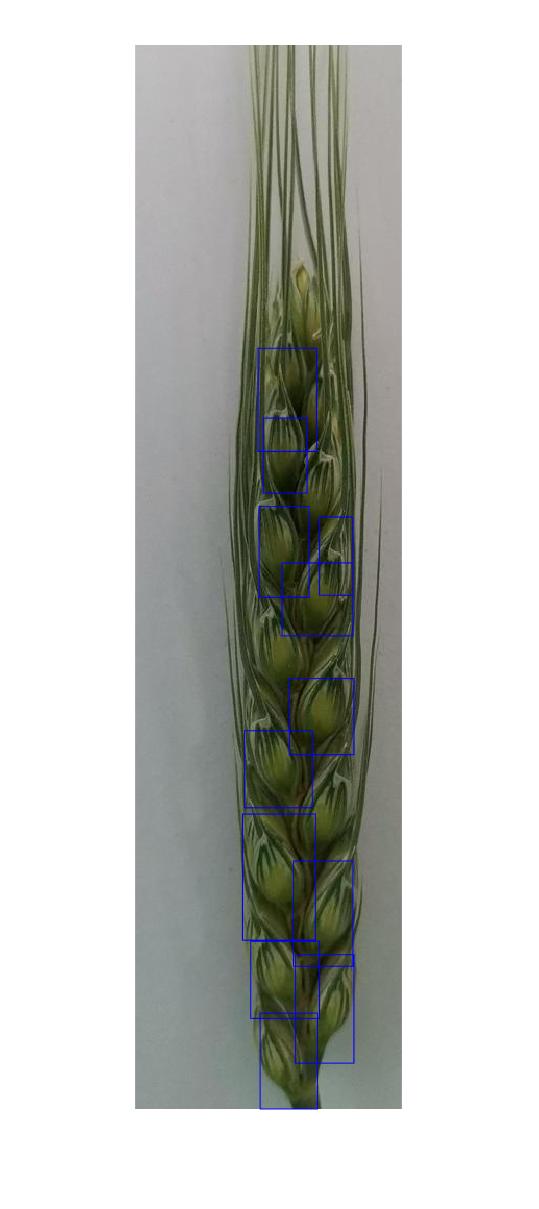

Supplement: Supplementary file 2 [file Data_Sheet_2.zip › 3. Labeling results of watershed algorithm (section Spikelet segmentation and annotation)/Liangxing 99/3043b.jpg]

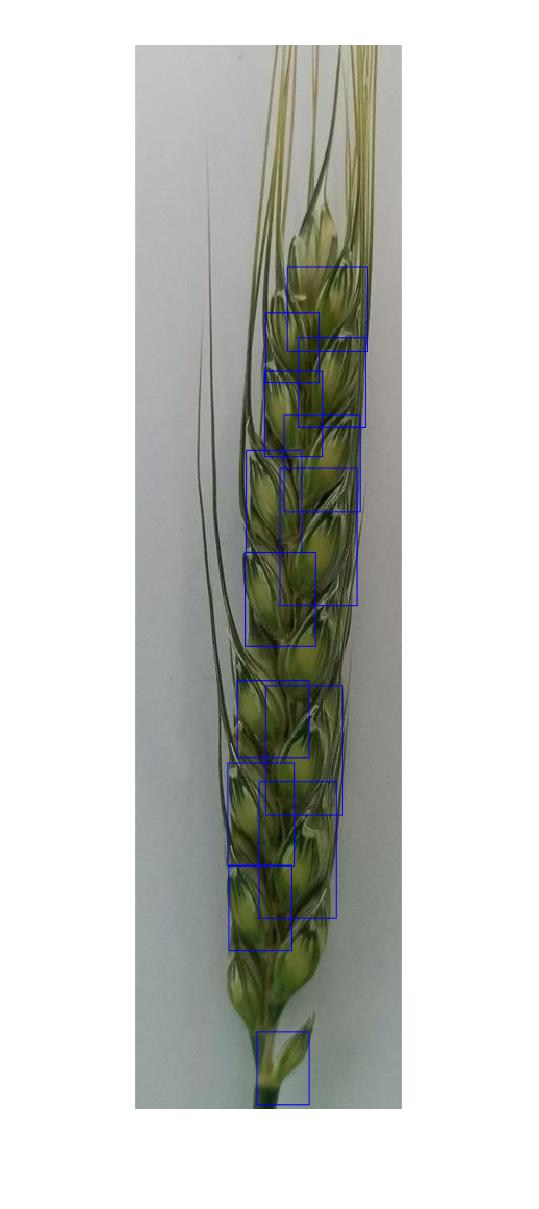

Supplement: Supplementary file 2 [file Data_Sheet_2.zip › 3. Labeling results of watershed algorithm (section Spikelet segmentation and annotation)/Liangxing 99/3052b.jpg]

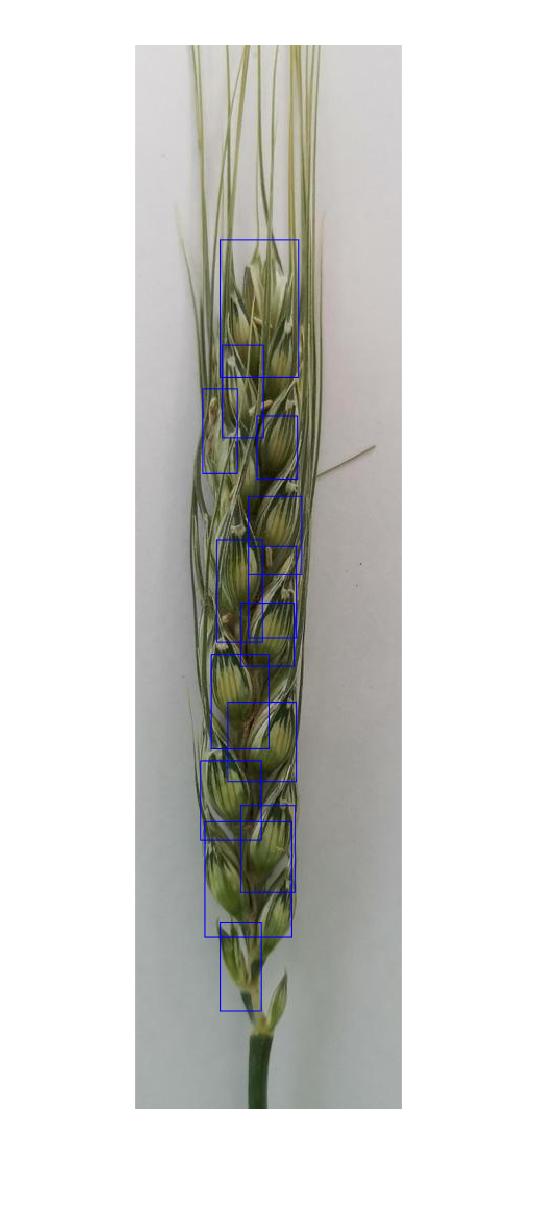

Supplement: Supplementary file 2 [file Data_Sheet_2.zip › 3. Labeling results of watershed algorithm (section Spikelet segmentation and annotation)/Liangxing 99/3055b.jpg]

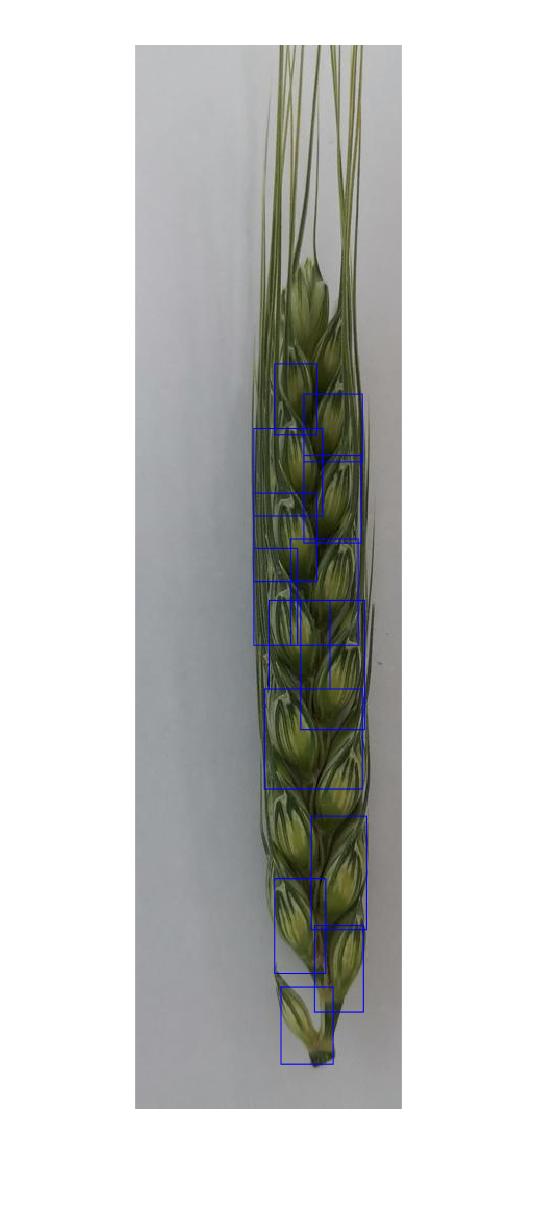

Supplement: Supplementary file 2 [file Data_Sheet_2.zip › 3. Labeling results of watershed algorithm (section Spikelet segmentation and annotation)/Liangxing 99/3057b.jpg]

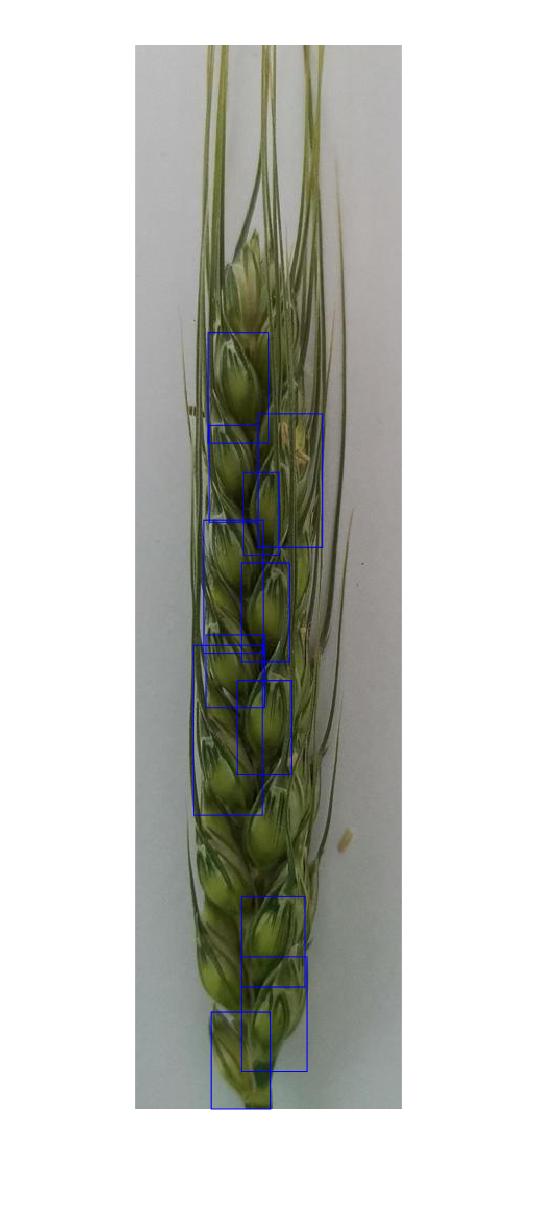

Supplement: Supplementary file 2 [file Data_Sheet_2.zip › 3. Labeling results of watershed algorithm (section Spikelet segmentation and annotation)/Liangxing 99/3058b.jpg]

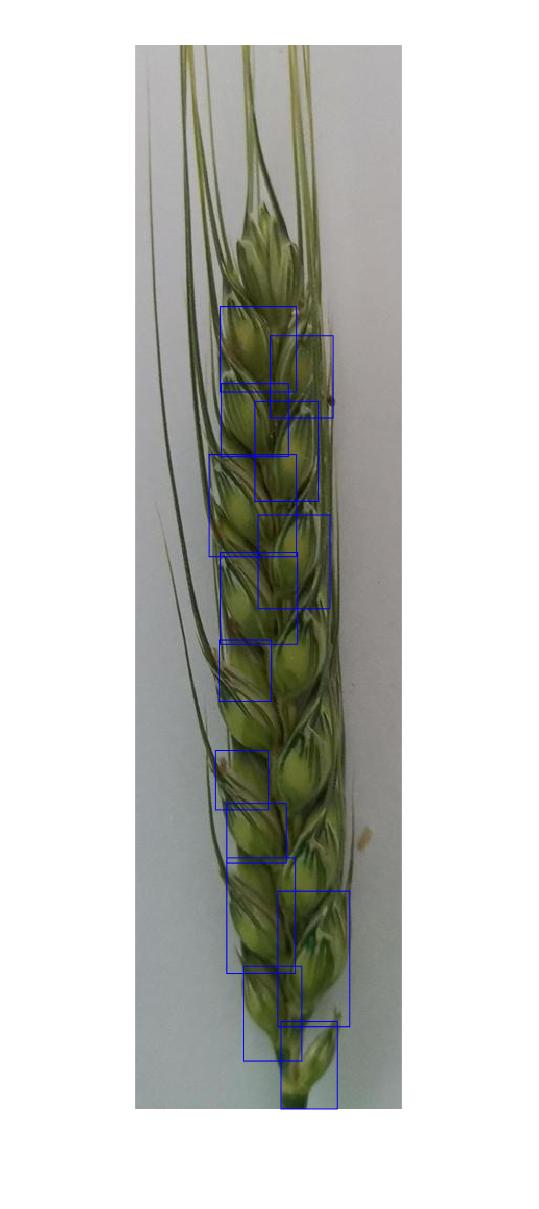

Supplement: Supplementary file 2 [file Data_Sheet_2.zip › 3. Labeling results of watershed algorithm (section Spikelet segmentation and annotation)/Liangxing 99/3059b.jpg]

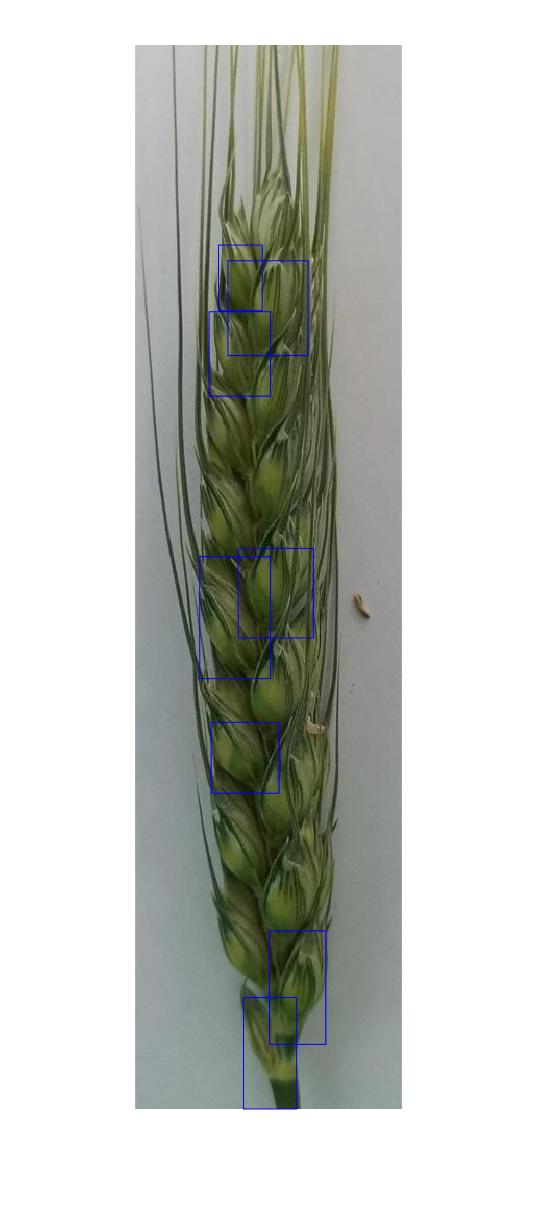

Supplement: Supplementary file 2 [file Data_Sheet_2.zip › 3. Labeling results of watershed algorithm (section Spikelet segmentation and annotation)/Liangxing 99/3060b.jpg]

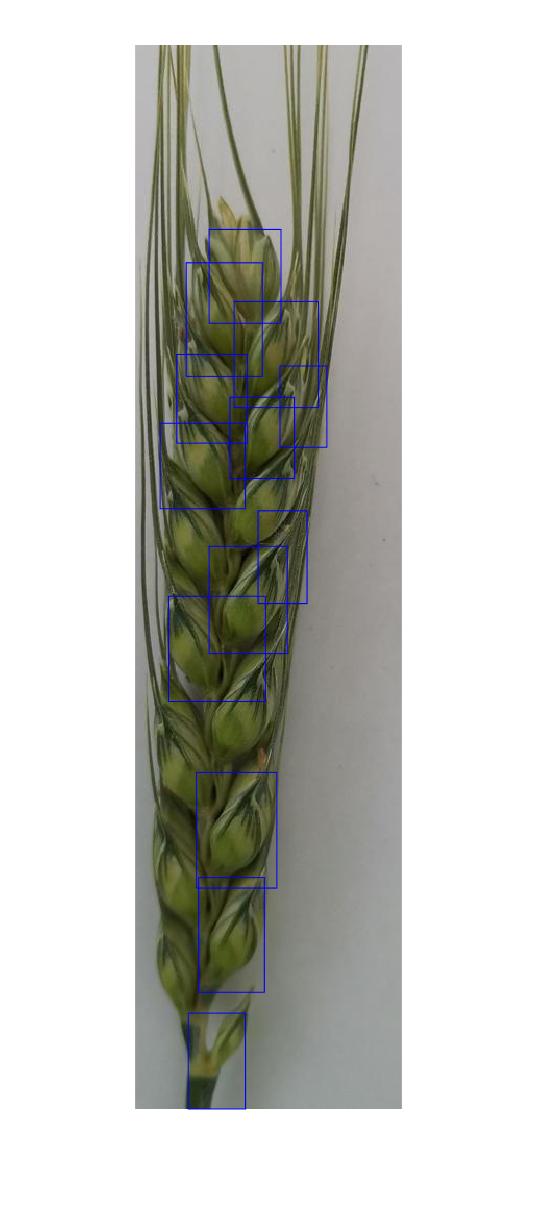

Supplement: Supplementary file 2 [file Data_Sheet_2.zip › 3. Labeling results of watershed algorithm (section Spikelet segmentation and annotation)/Liangxing 99/3064b.jpg]

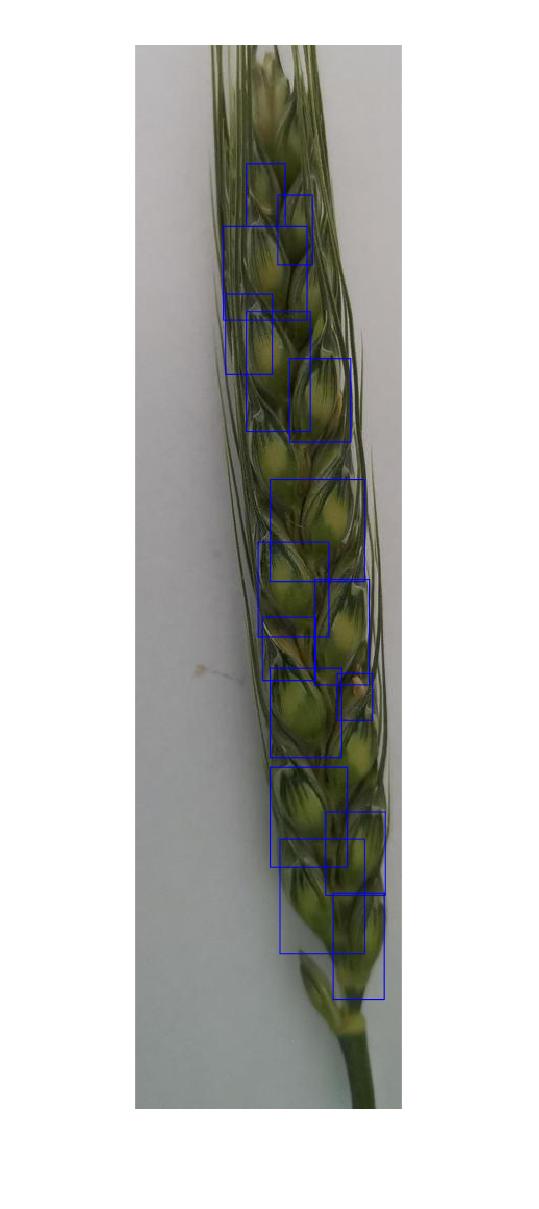

Supplement: Supplementary file 2 [file Data_Sheet_2.zip › 3. Labeling results of watershed algorithm (section Spikelet segmentation and annotation)/Liangxing 99/3071b.jpg]

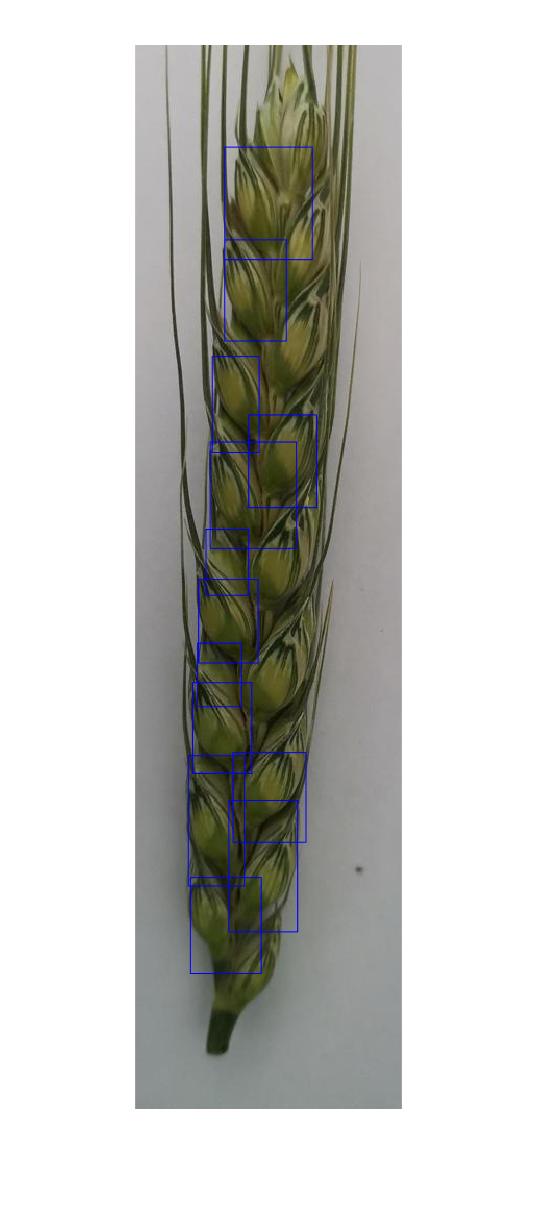

Supplement: Supplementary file 2 [file Data_Sheet_2.zip › 3. Labeling results of watershed algorithm (section Spikelet segmentation and annotation)/Liangxing 99/3073b.jpg]

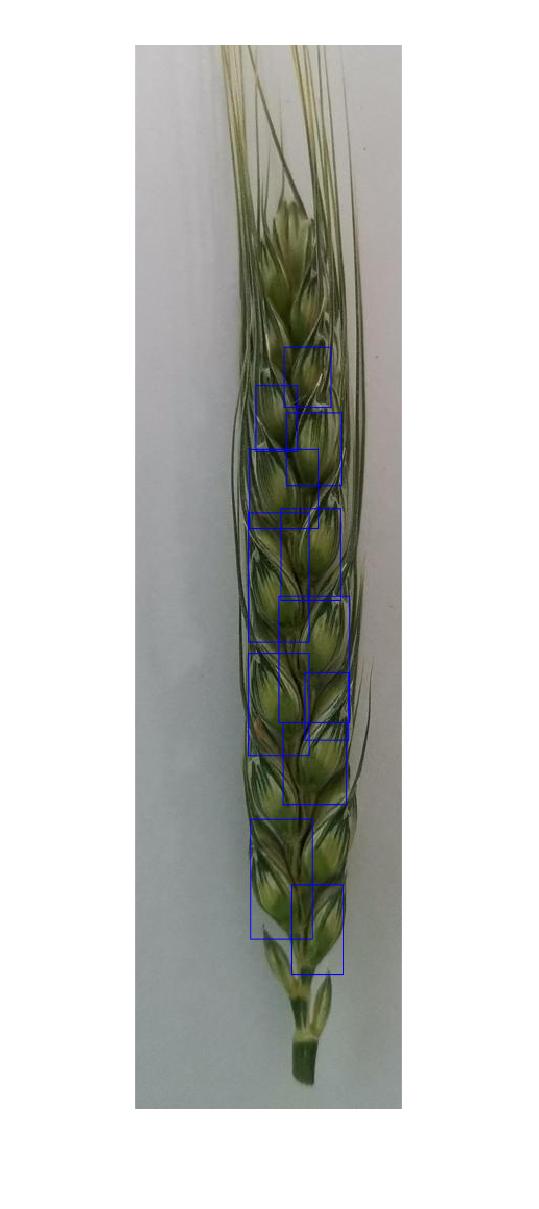

Supplement: Supplementary file 2 [file Data_Sheet_2.zip › 3. Labeling results of watershed algorithm (section Spikelet segmentation and annotation)/Liangxing 99/3075b.jpg]

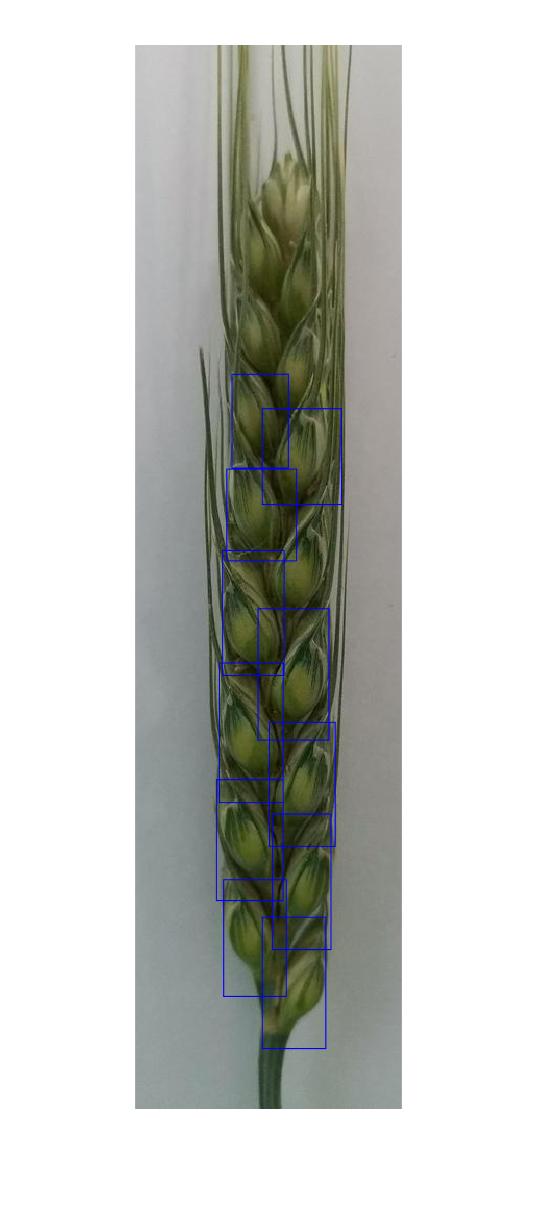

Supplement: Supplementary file 2 [file Data_Sheet_2.zip › 3. Labeling results of watershed algorithm (section Spikelet segmentation and annotation)/Liangxing 99/3076b.jpg]

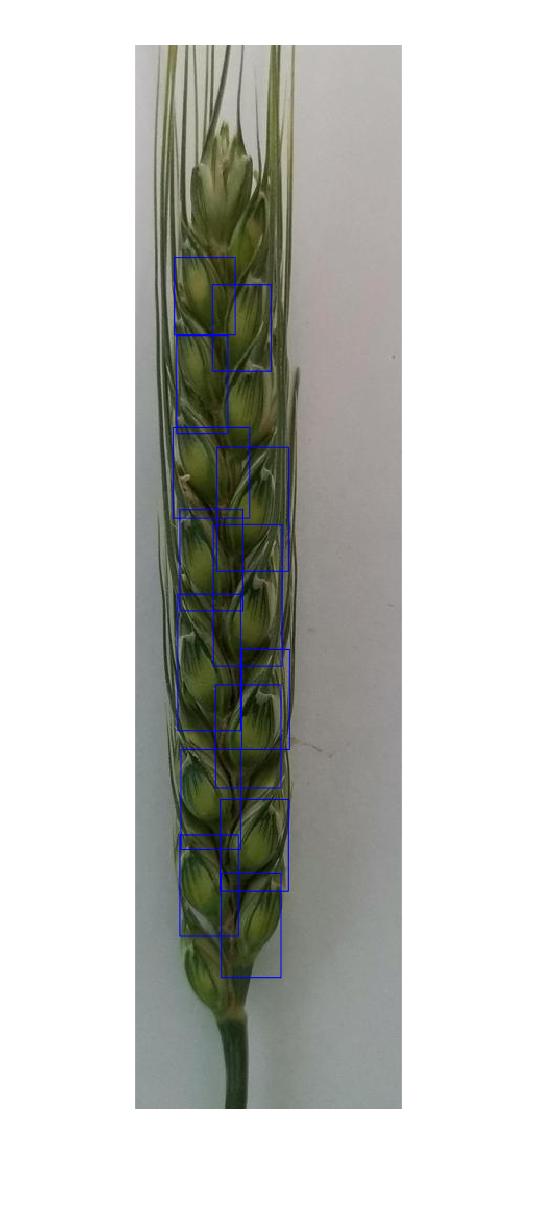

Supplement: Supplementary file 2 [file Data_Sheet_2.zip › 3. Labeling results of watershed algorithm (section Spikelet segmentation and annotation)/Liangxing 99/3077b.jpg]

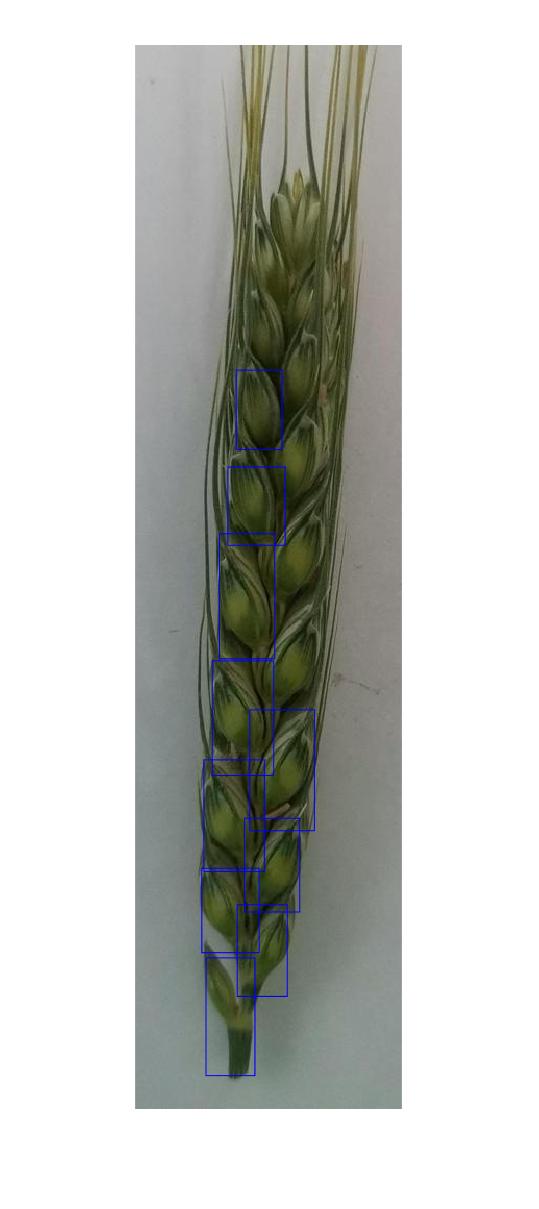

Supplement: Supplementary file 2 [file Data_Sheet_2.zip › 3. Labeling results of watershed algorithm (section Spikelet segmentation and annotation)/Liangxing 99/3078b.jpg]

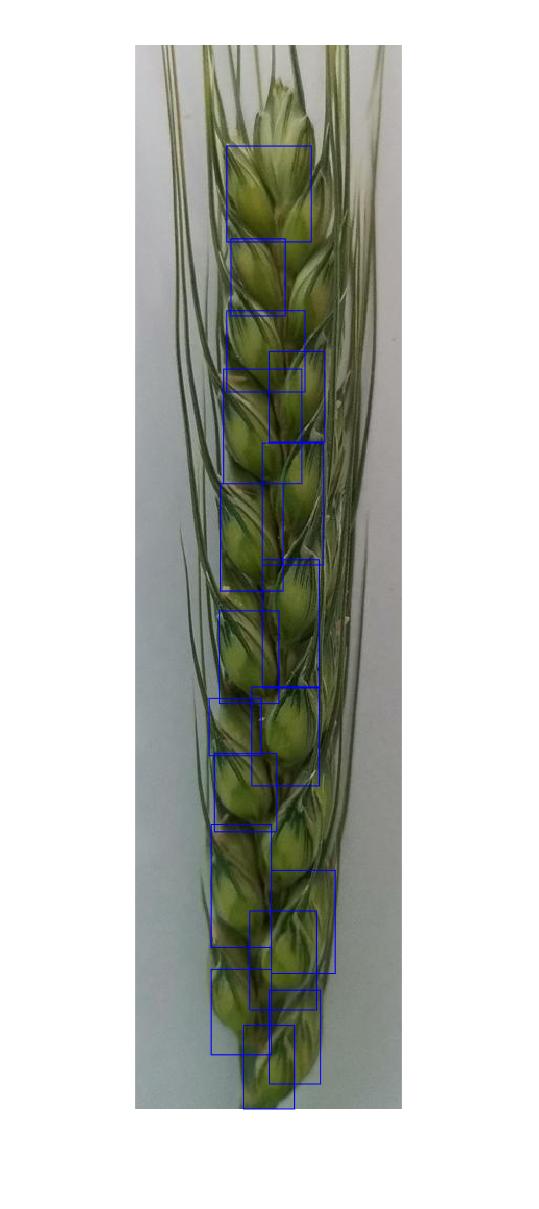

Supplement: Supplementary file 2 [file Data_Sheet_2.zip › 3. Labeling results of watershed algorithm (section Spikelet segmentation and annotation)/Liangxing 99/3082b.jpg]

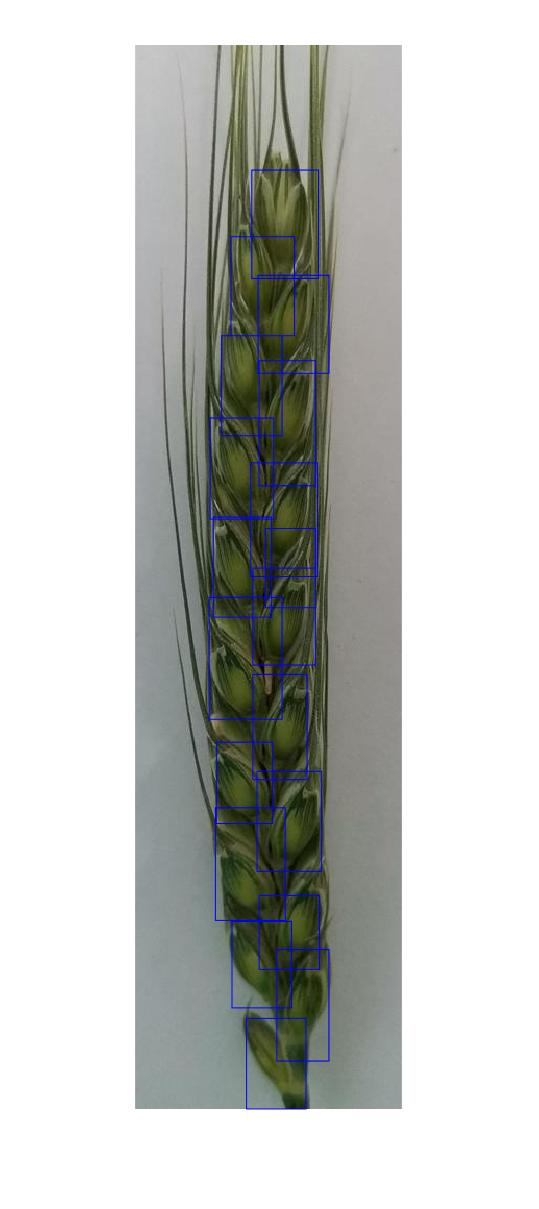

Supplement: Supplementary file 2 [file Data_Sheet_2.zip › 3. Labeling results of watershed algorithm (section Spikelet segmentation and annotation)/Liangxing 99/3086b.jpg]

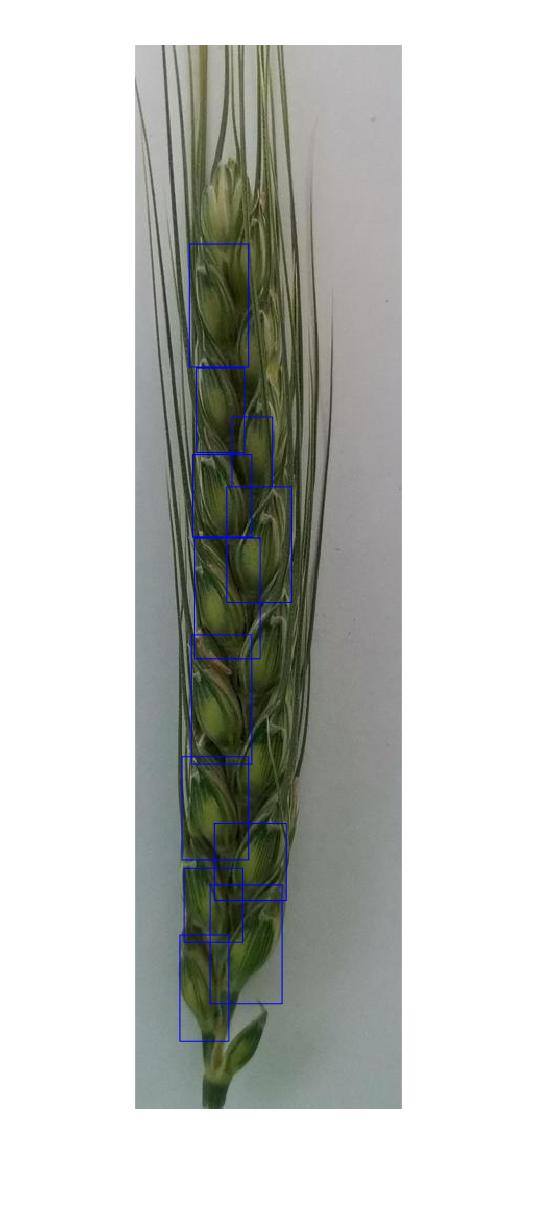

Supplement: Supplementary file 2 [file Data_Sheet_2.zip › 3. Labeling results of watershed algorithm (section Spikelet segmentation and annotation)/Liangxing 99/3087b.jpg]

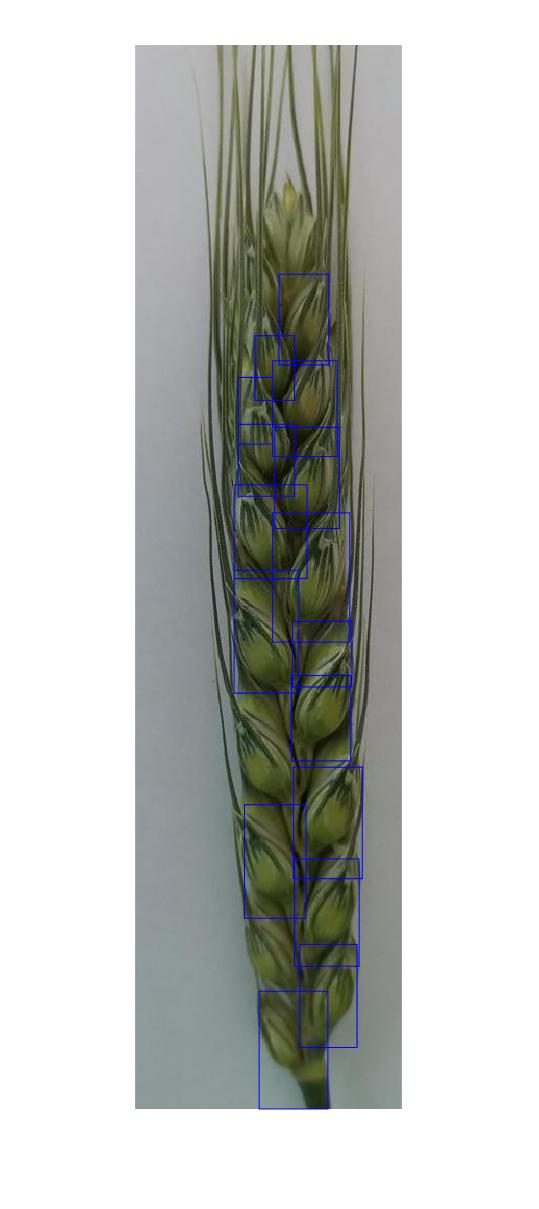

Supplement: Supplementary file 2 [file Data_Sheet_2.zip › 3. Labeling results of watershed algorithm (section Spikelet segmentation and annotation)/Liangxing 99/3094b.jpg]

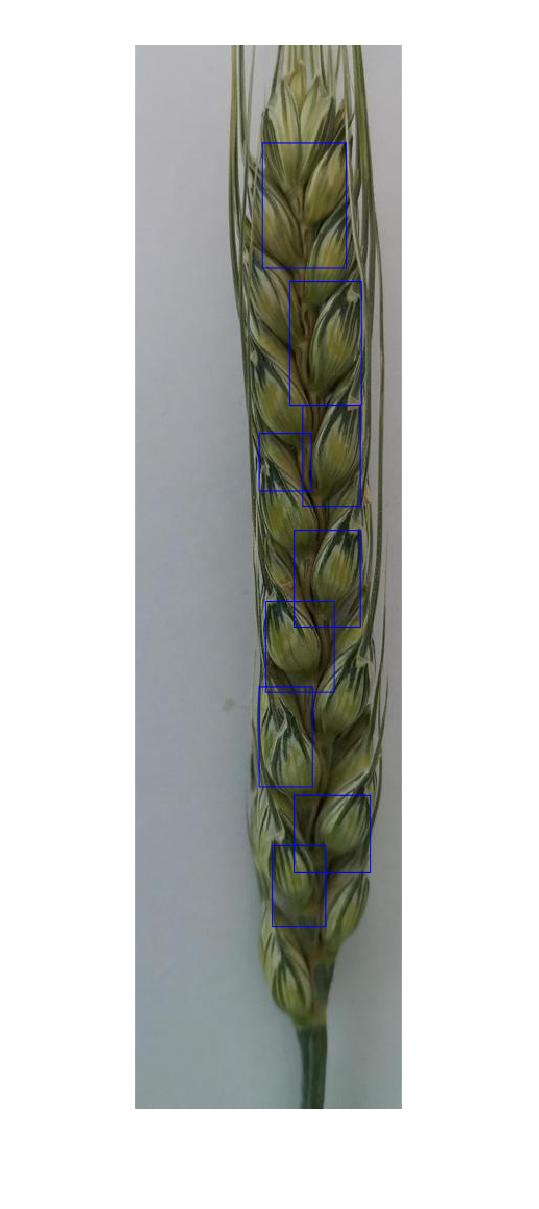

Supplement: Supplementary file 2 [file Data_Sheet_2.zip › 3. Labeling results of watershed algorithm (section Spikelet segmentation and annotation)/Liangxing 99/3096b.jpg]

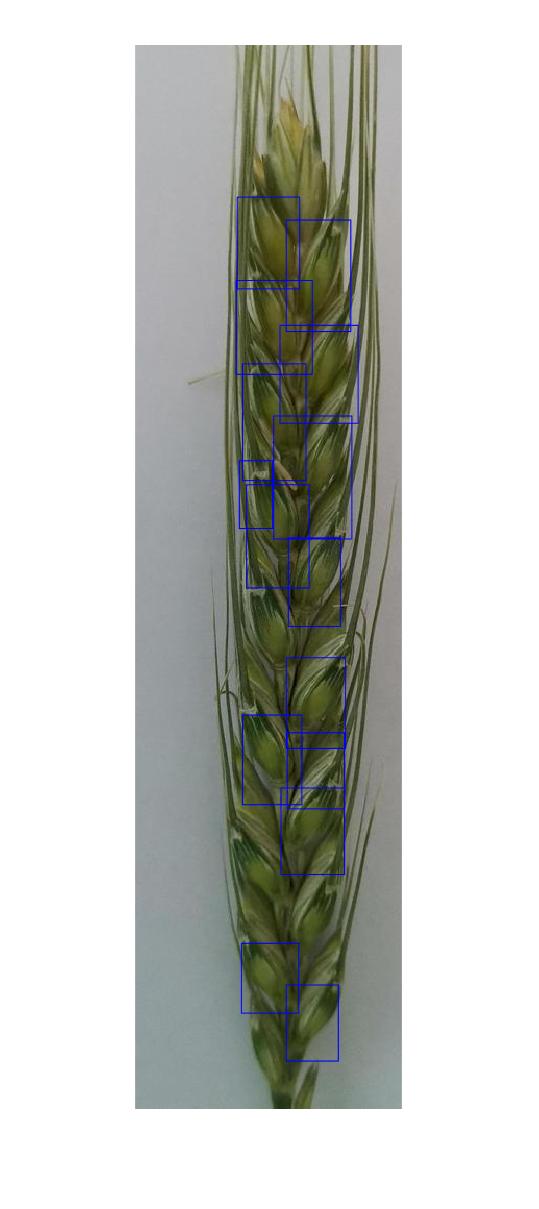

Supplement: Supplementary file 2 [file Data_Sheet_2.zip › 3. Labeling results of watershed algorithm (section Spikelet segmentation and annotation)/Liangxing 99/3099b.jpg]

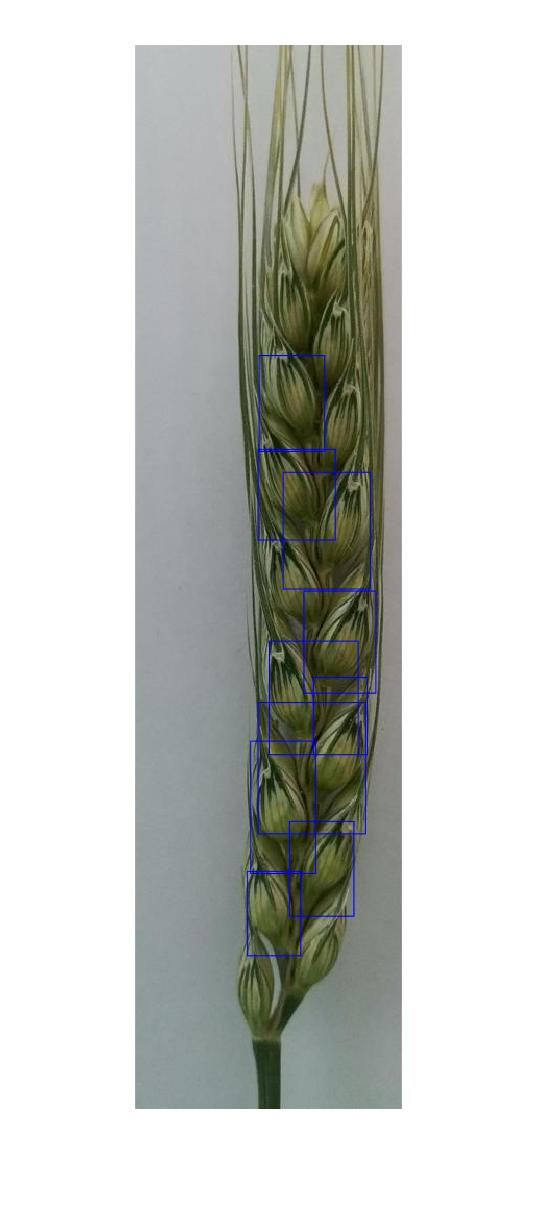

Supplement: Supplementary file 2 [file Data_Sheet_2.zip › 3. Labeling results of watershed algorithm (section Spikelet segmentation and annotation)/Liangxing 99/3101b.jpg]

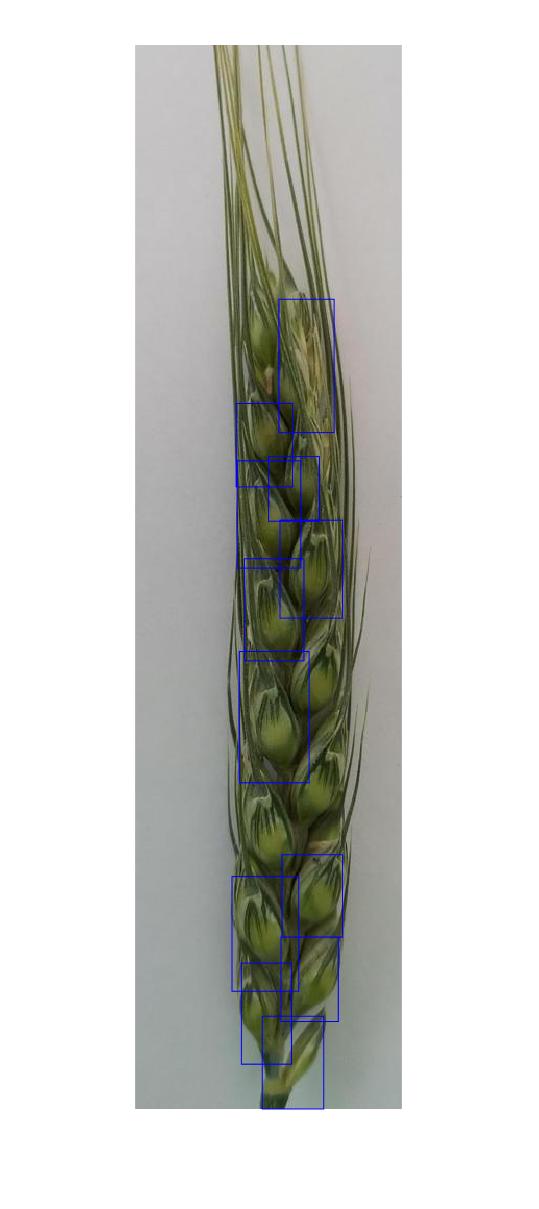

Supplement: Supplementary file 2 [file Data_Sheet_2.zip › 3. Labeling results of watershed algorithm (section Spikelet segmentation and annotation)/Liangxing 99/3105b.jpg]

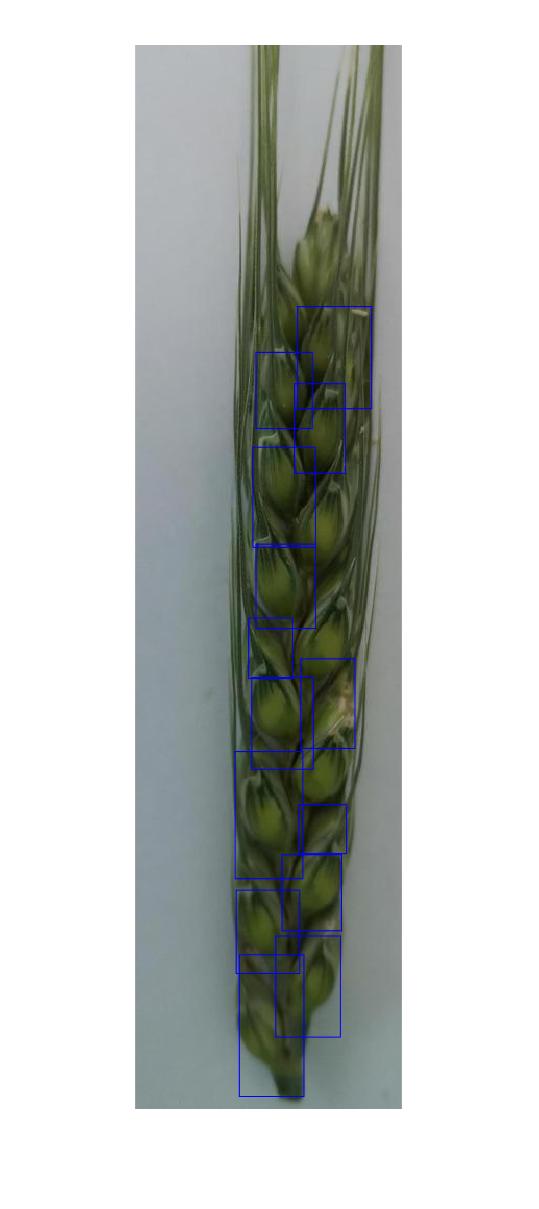

Supplement: Supplementary file 2 [file Data_Sheet_2.zip › 3. Labeling results of watershed algorithm (section Spikelet segmentation and annotation)/Liangxing 99/3108b.jpg]

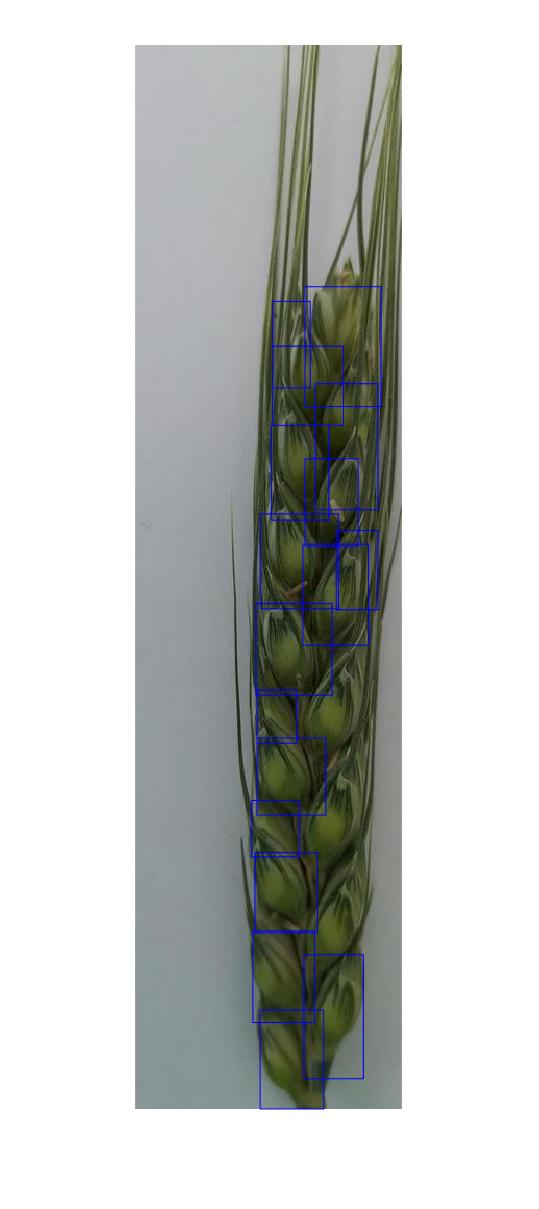

Supplement: Supplementary file 2 [file Data_Sheet_2.zip › 3. Labeling results of watershed algorithm (section Spikelet segmentation and annotation)/Liangxing 99/3110b.jpg]

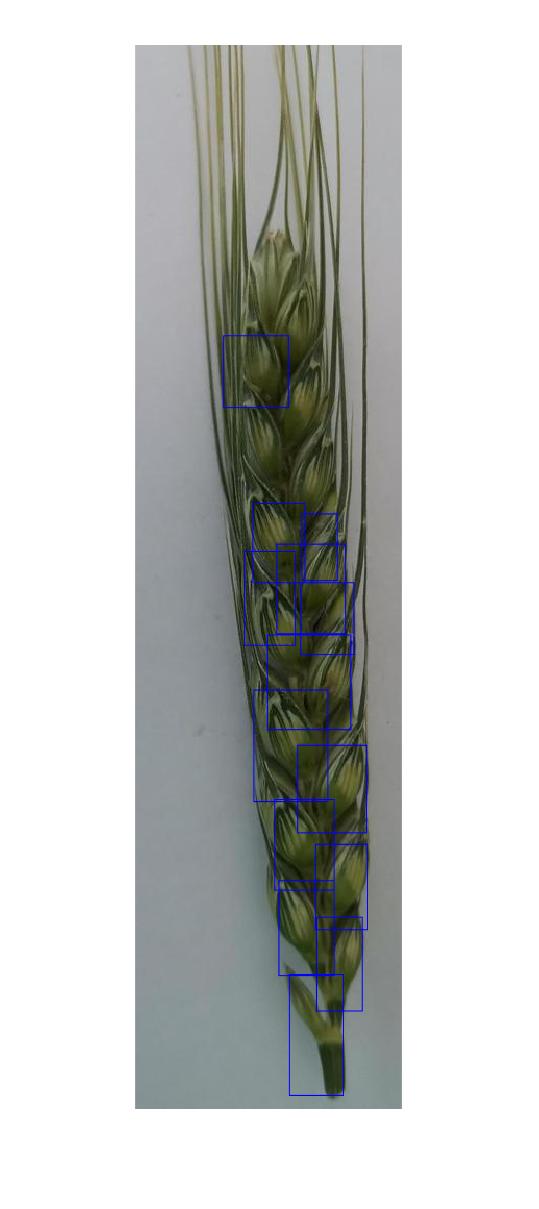

Supplement: Supplementary file 2 [file Data_Sheet_2.zip › 3. Labeling results of watershed algorithm (section Spikelet segmentation and annotation)/Liangxing 99/3115b.jpg]

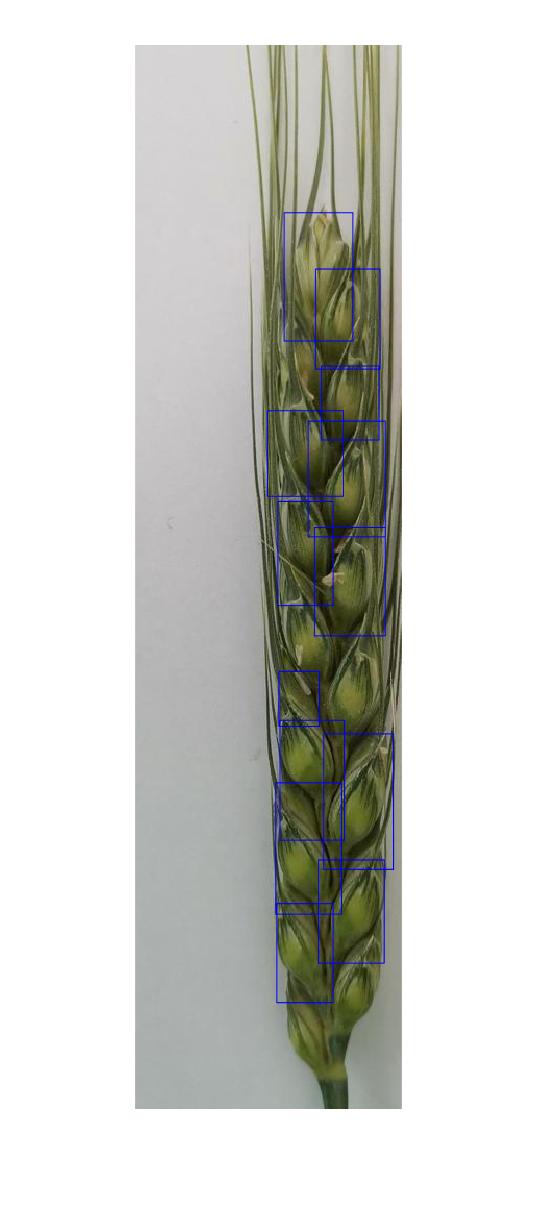

Supplement: Supplementary file 2 [file Data_Sheet_2.zip › 3. Labeling results of watershed algorithm (section Spikelet segmentation and annotation)/Liangxing 99/3116b.jpg]

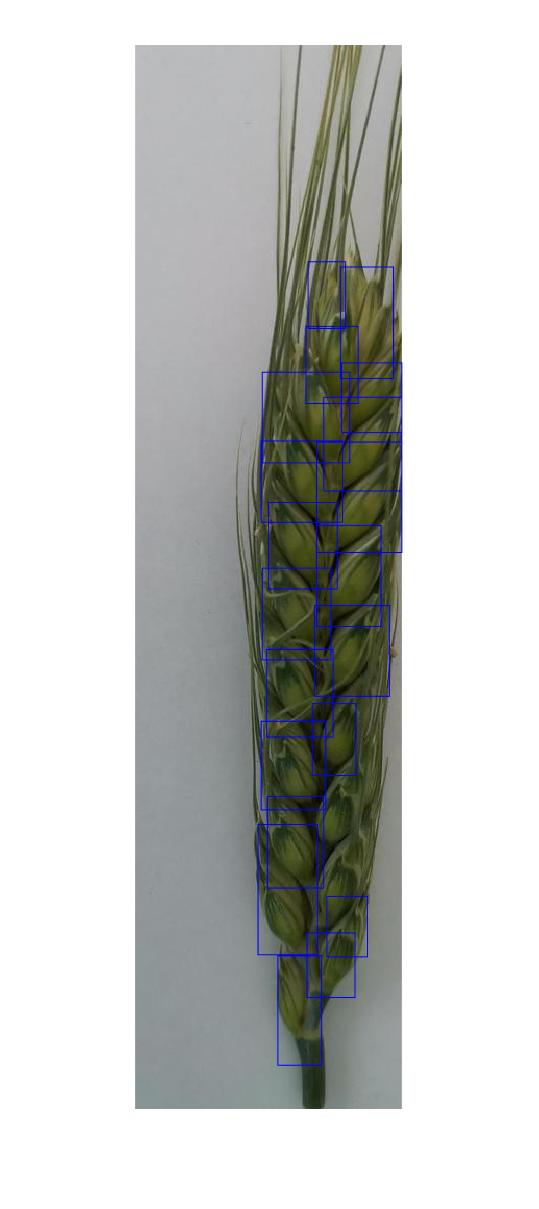

Supplement: Supplementary file 2 [file Data_Sheet_2.zip › 3. Labeling results of watershed algorithm (section Spikelet segmentation and annotation)/Liangxing 99/3120b.jpg]

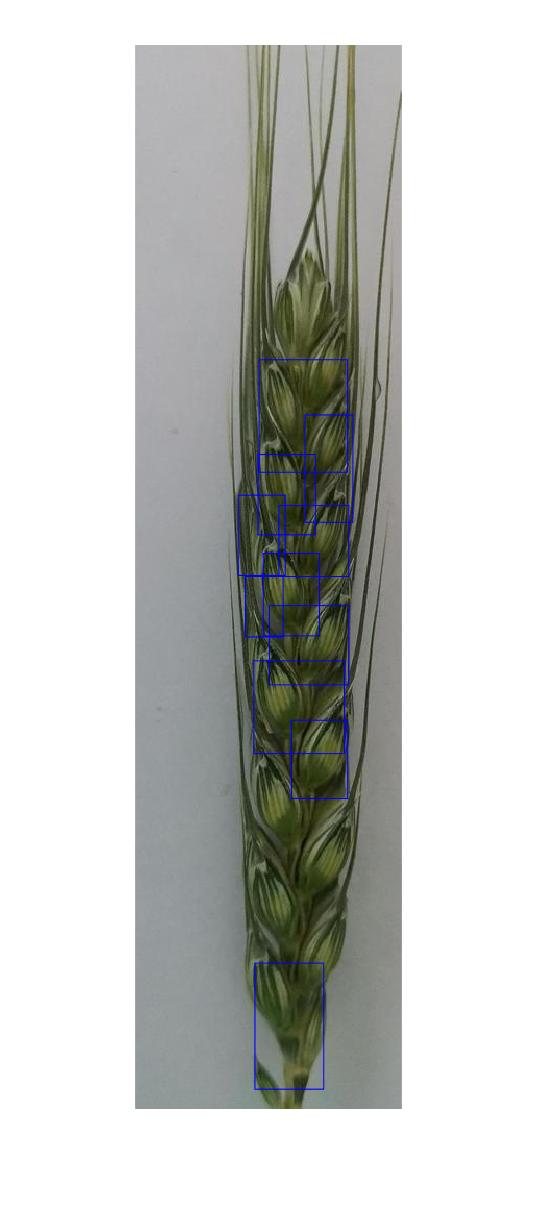

Supplement: Supplementary file 2 [file Data_Sheet_2.zip › 3. Labeling results of watershed algorithm (section Spikelet segmentation and annotation)/Liangxing 99/3133b.jpg]

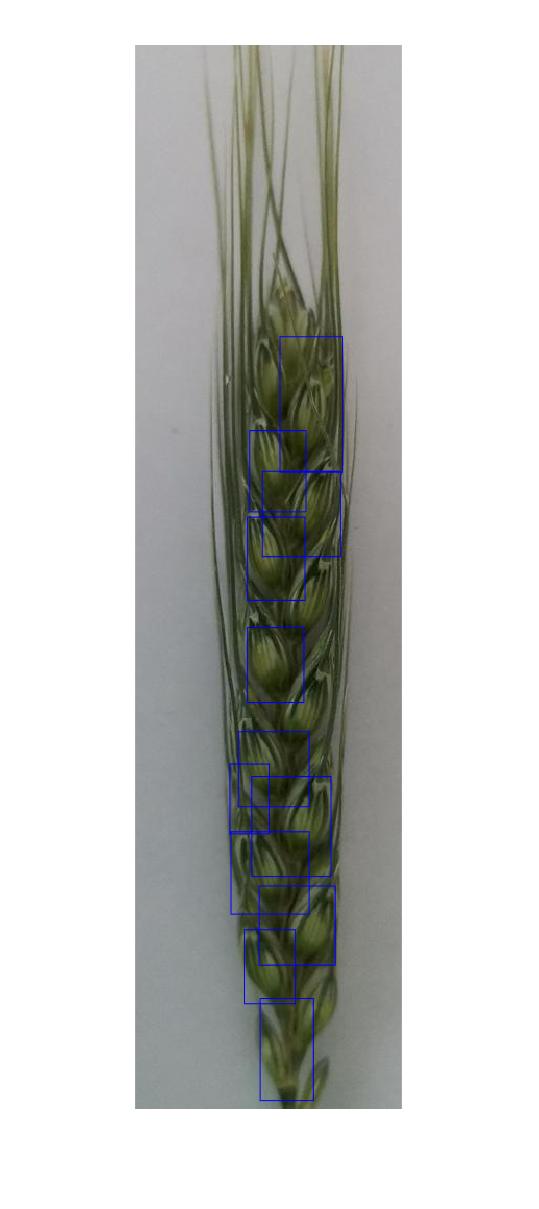

Supplement: Supplementary file 2 [file Data_Sheet_2.zip › 3. Labeling results of watershed algorithm (section Spikelet segmentation and annotation)/Liangxing 99/3134b.jpg]

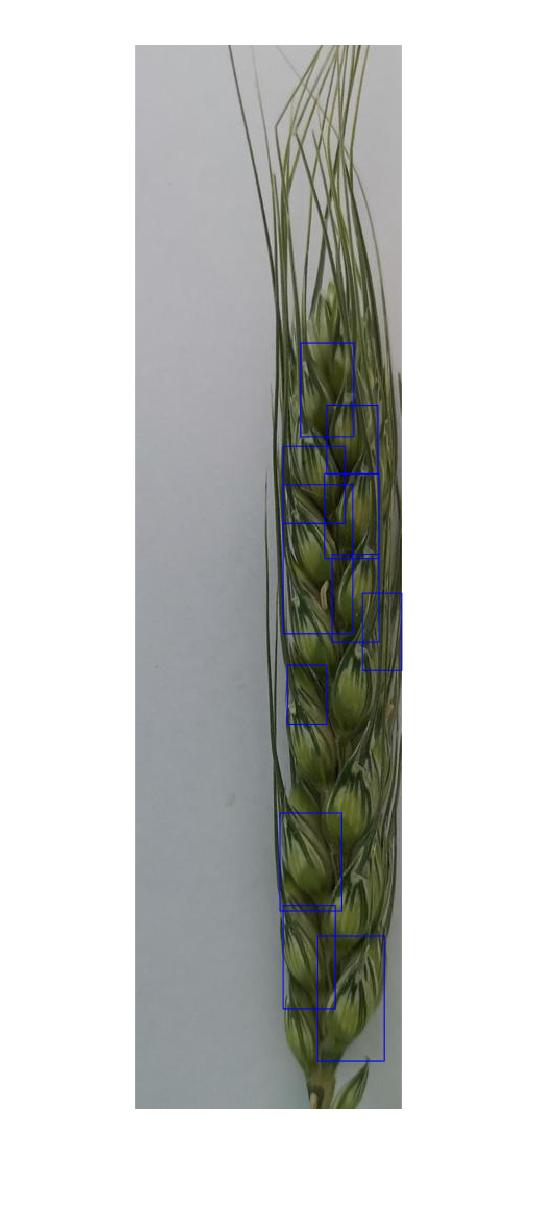

Supplement: Supplementary file 2 [file Data_Sheet_2.zip › 3. Labeling results of watershed algorithm (section Spikelet segmentation and annotation)/Liangxing 99/3140b.jpg]

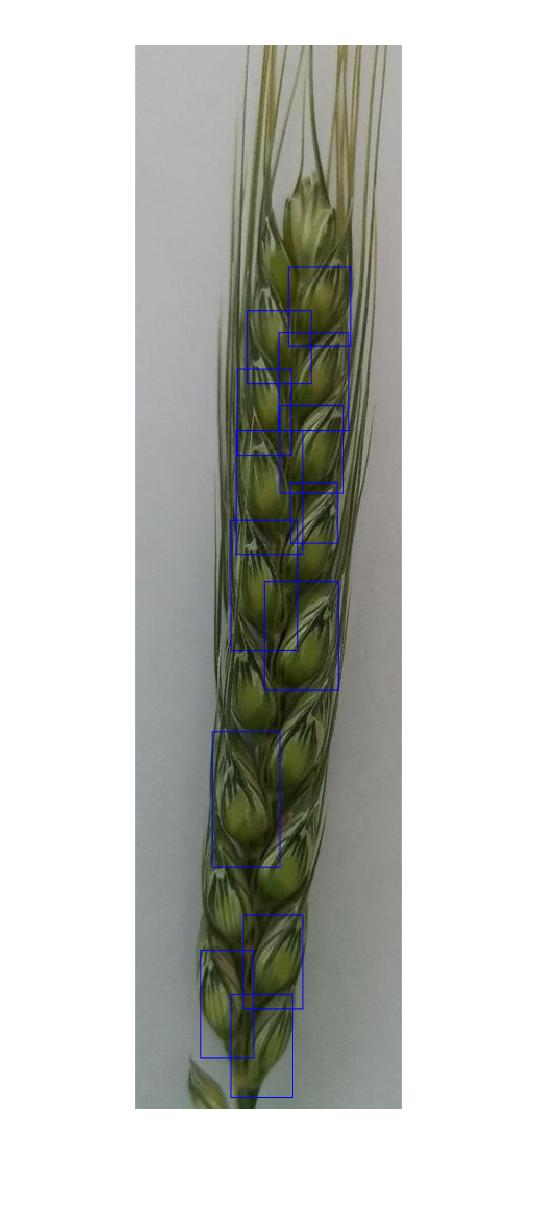

Supplement: Supplementary file 2 [file Data_Sheet_2.zip › 3. Labeling results of watershed algorithm (section Spikelet segmentation and annotation)/Liangxing 99/3143b.jpg]

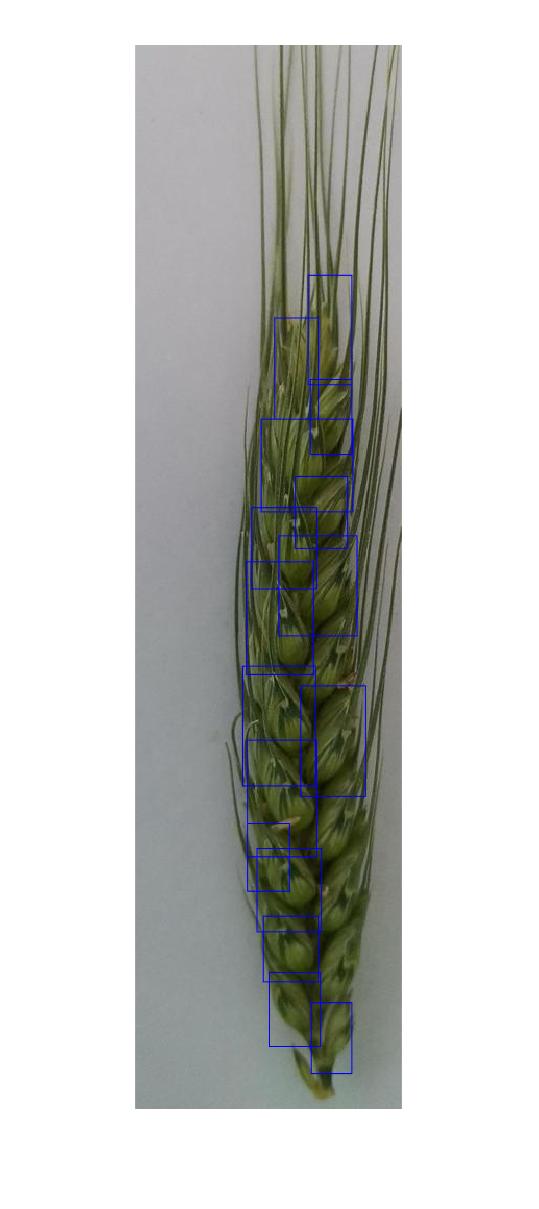

Supplement: Supplementary file 2 [file Data_Sheet_2.zip › 3. Labeling results of watershed algorithm (section Spikelet segmentation and annotation)/Liangxing 99/3149b.jpg]

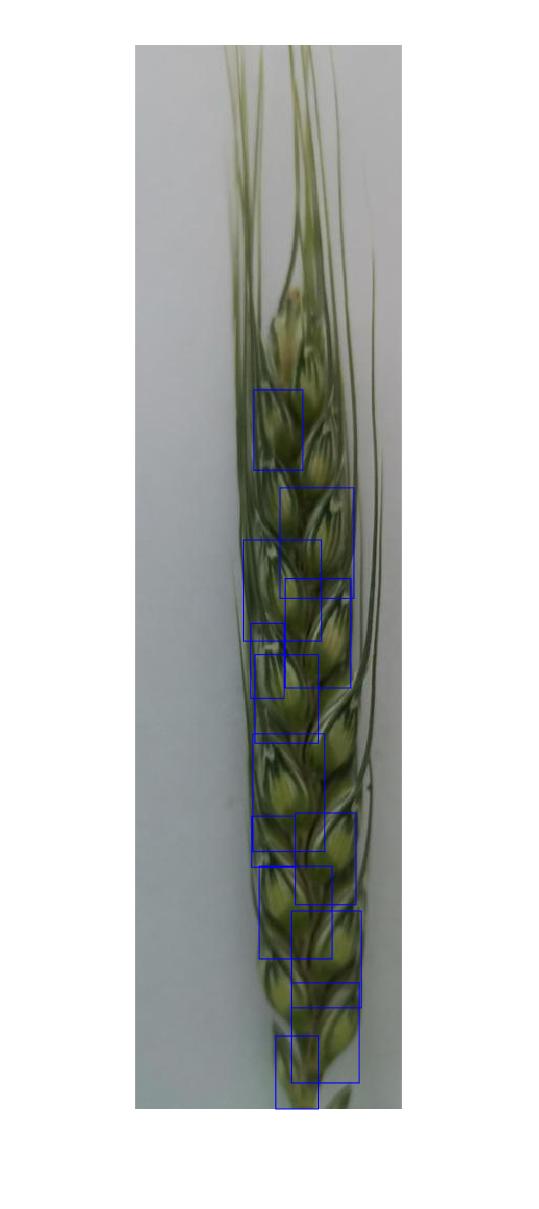

Supplement: Supplementary file 2 [file Data_Sheet_2.zip › 3. Labeling results of watershed algorithm (section Spikelet segmentation and annotation)/Liangxing 99/3151b.jpg]

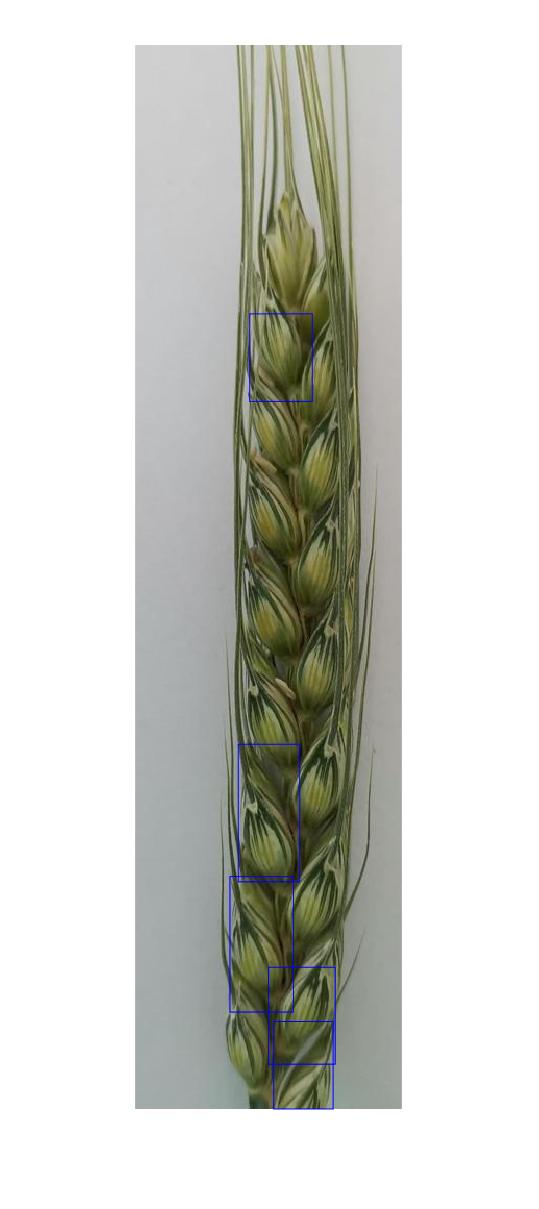

Supplement: Supplementary file 2 [file Data_Sheet_2.zip › 3. Labeling results of watershed algorithm (section Spikelet segmentation and annotation)/Liangxing 99/3153b.jpg]

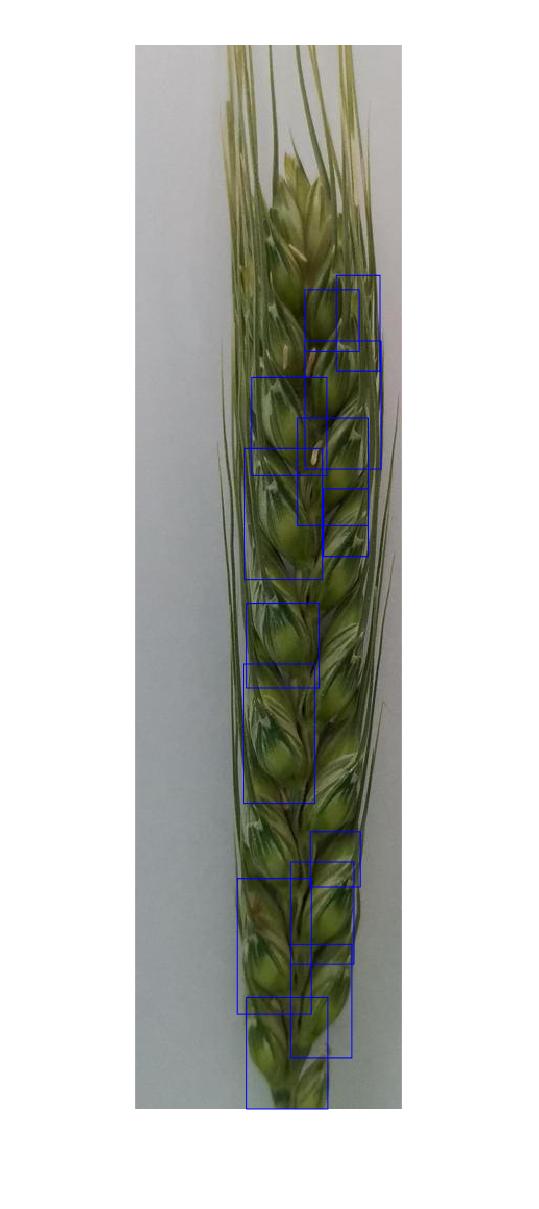

Supplement: Supplementary file 2 [file Data_Sheet_2.zip › 3. Labeling results of watershed algorithm (section Spikelet segmentation and annotation)/Liangxing 99/3157b.jpg]

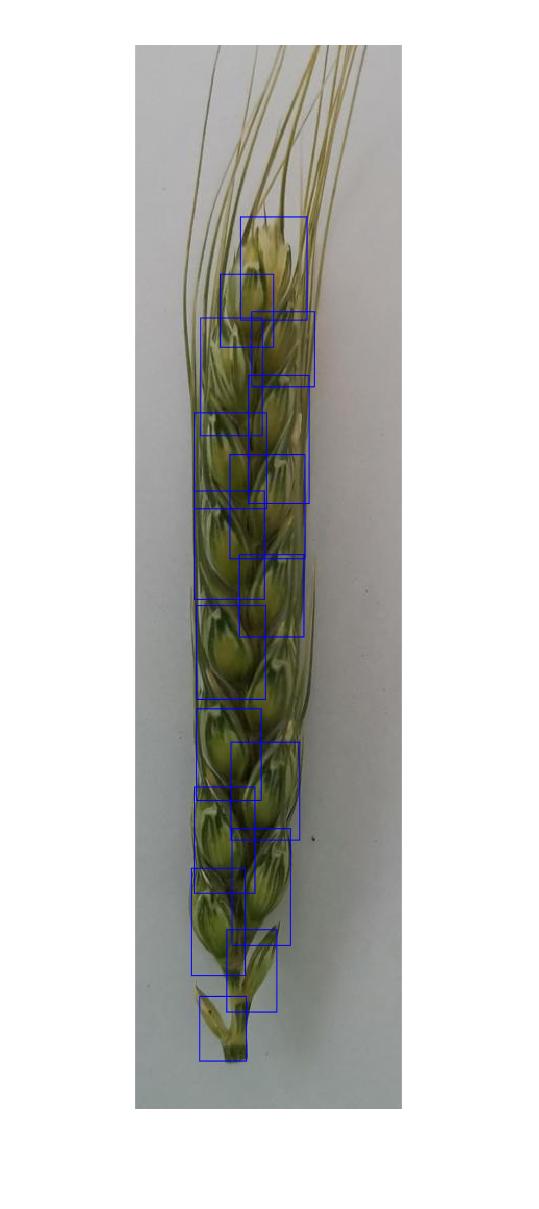

Supplement: Supplementary file 2 [file Data_Sheet_2.zip › 3. Labeling results of watershed algorithm (section Spikelet segmentation and annotation)/Liangxing 99/3166b.jpg]

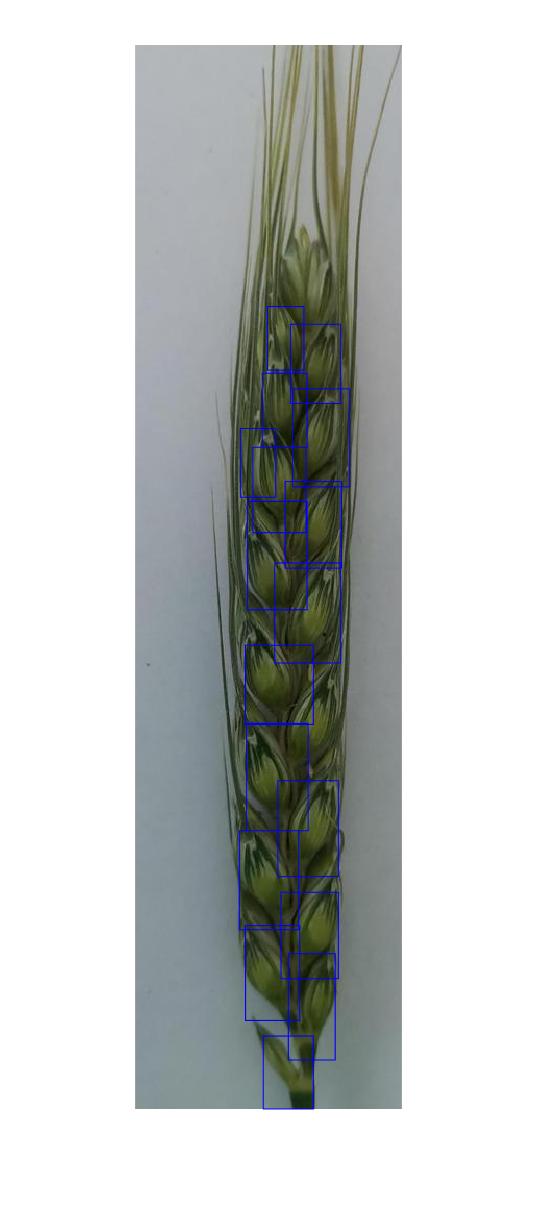

Supplement: Supplementary file 2 [file Data_Sheet_2.zip › 3. Labeling results of watershed algorithm (section Spikelet segmentation and annotation)/Liangxing 99/3170b.jpg]

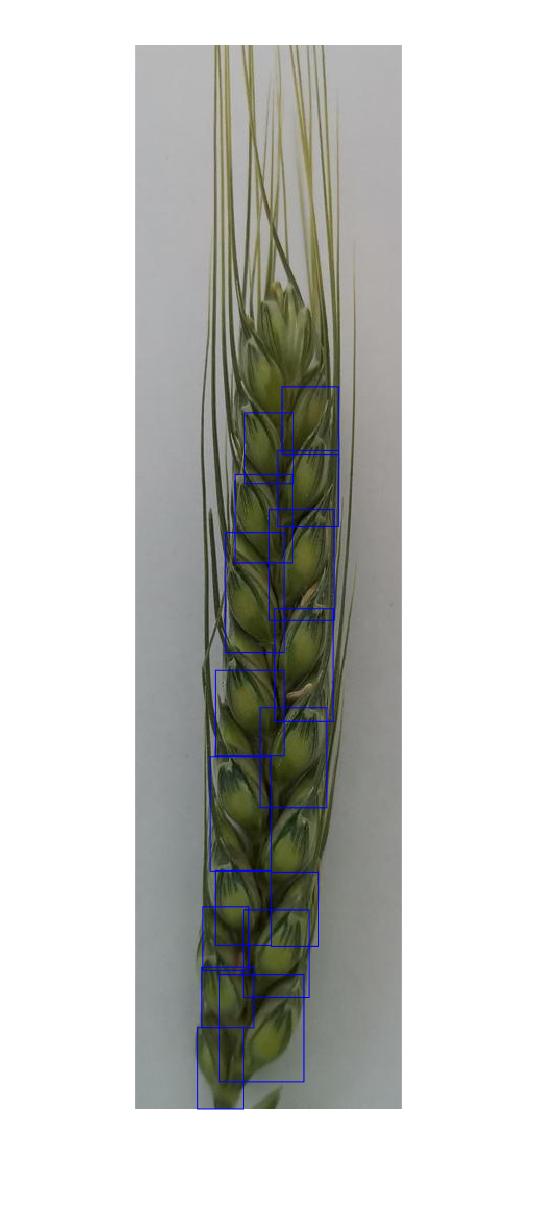

Supplement: Supplementary file 2 [file Data_Sheet_2.zip › 3. Labeling results of watershed algorithm (section Spikelet segmentation and annotation)/Liangxing 99/3174b.jpg]

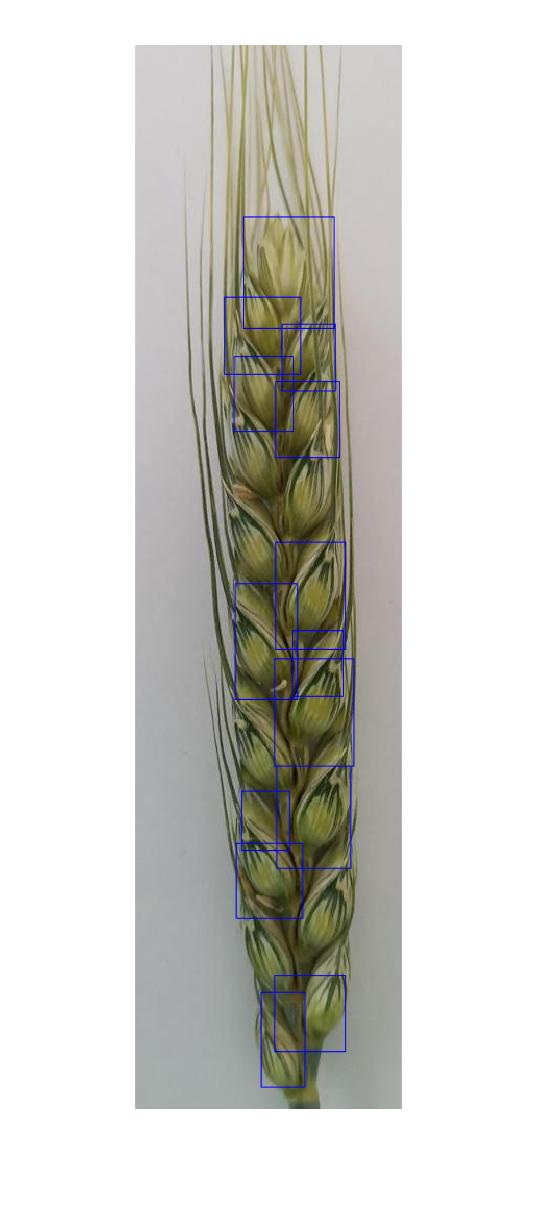

Supplement: Supplementary file 2 [file Data_Sheet_2.zip › 3. Labeling results of watershed algorithm (section Spikelet segmentation and annotation)/Liangxing 99/3175b.jpg]

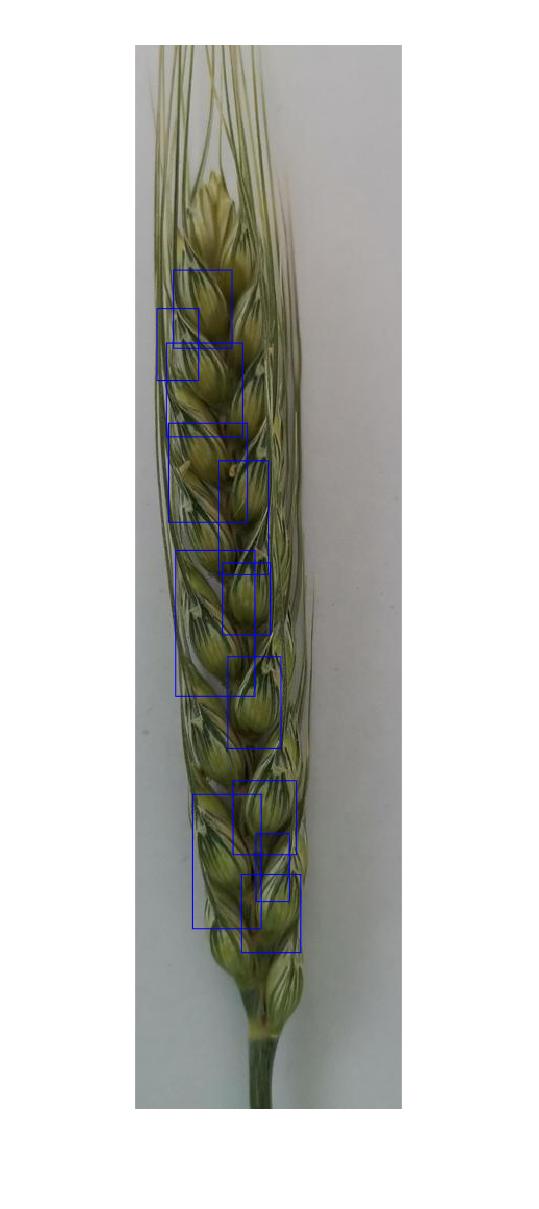

Supplement: Supplementary file 2 [file Data_Sheet_2.zip › 3. Labeling results of watershed algorithm (section Spikelet segmentation and annotation)/Liangxing 99/3176b.jpg]

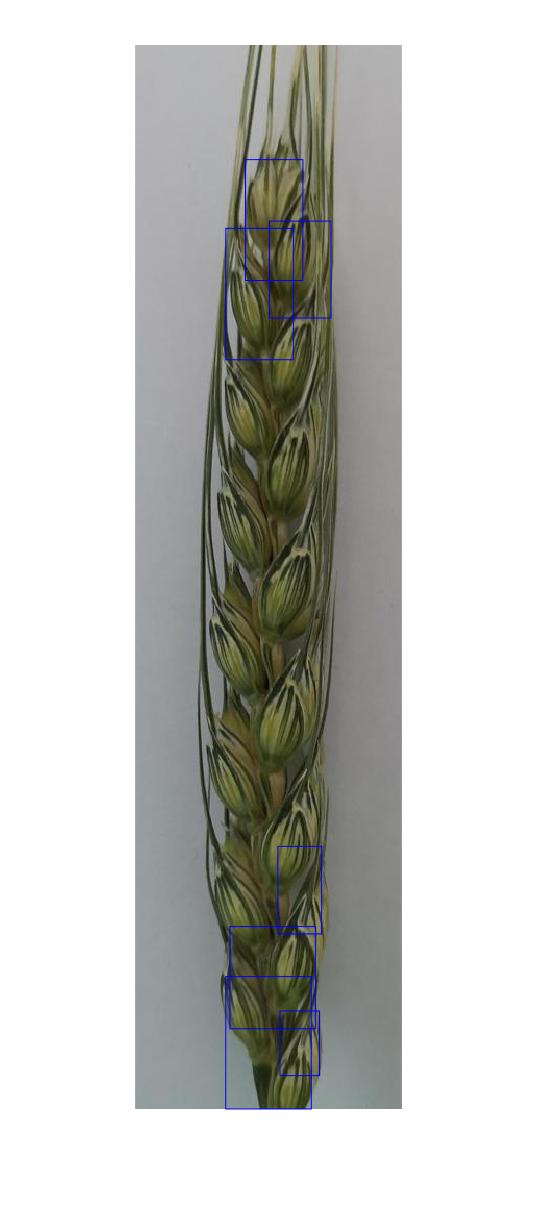

Supplement: Supplementary file 2 [file Data_Sheet_2.zip › 3. Labeling results of watershed algorithm (section Spikelet segmentation and annotation)/Liangxing 99/3182b.jpg]

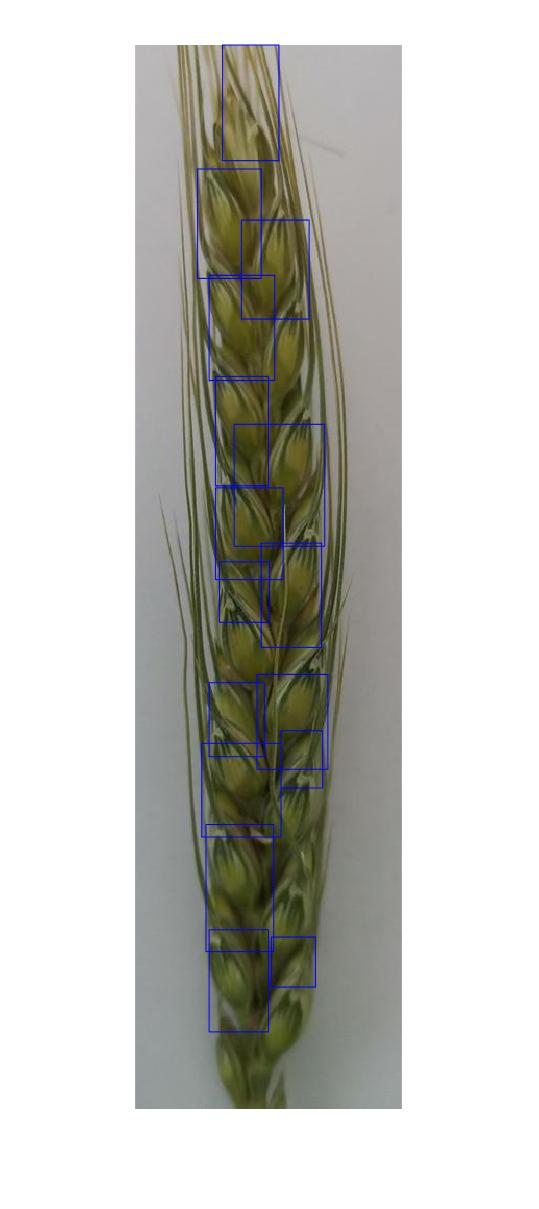

Supplement: Supplementary file 2 [file Data_Sheet_2.zip › 3. Labeling results of watershed algorithm (section Spikelet segmentation and annotation)/Liangxing 99/3184b.jpg]

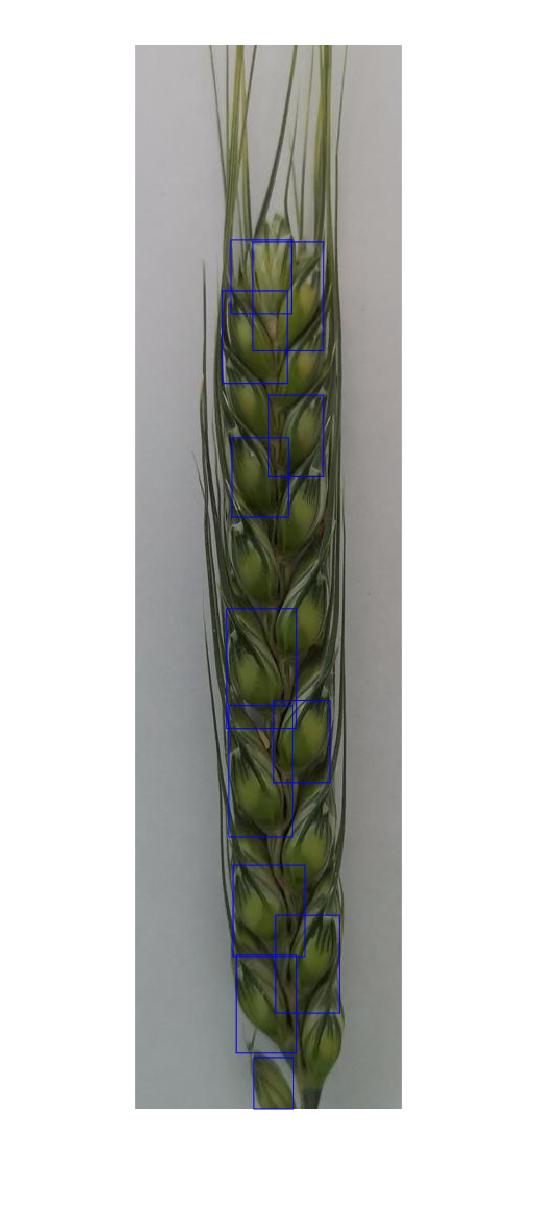

Supplement: Supplementary file 2 [file Data_Sheet_2.zip › 3. Labeling results of watershed algorithm (section Spikelet segmentation and annotation)/Liangxing 99/3185b.jpg]

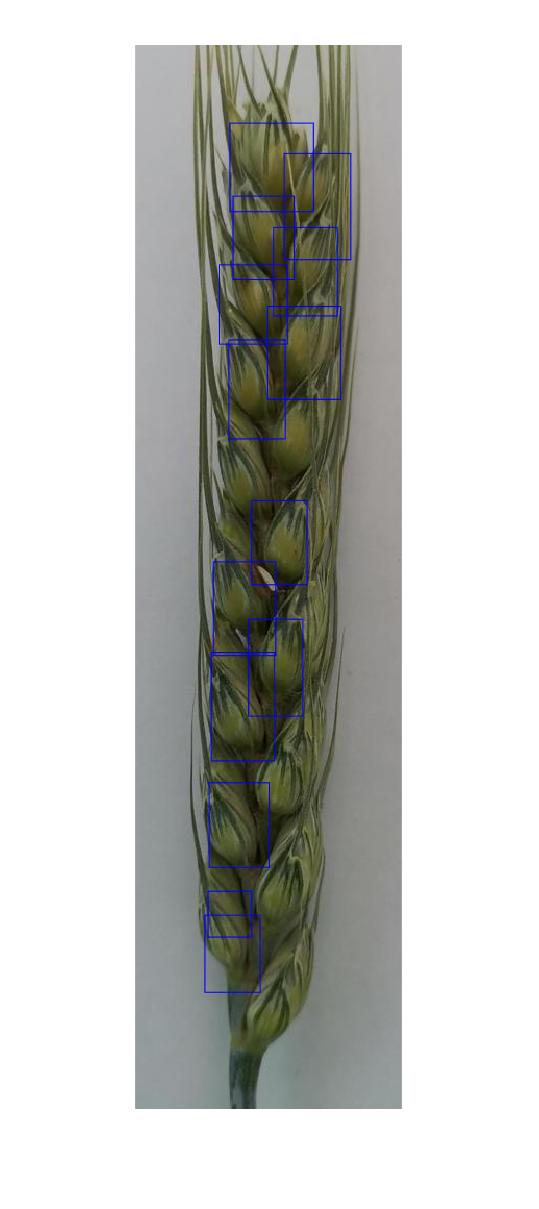

Supplement: Supplementary file 2 [file Data_Sheet_2.zip › 3. Labeling results of watershed algorithm (section Spikelet segmentation and annotation)/Liangxing 99/3188b.jpg]

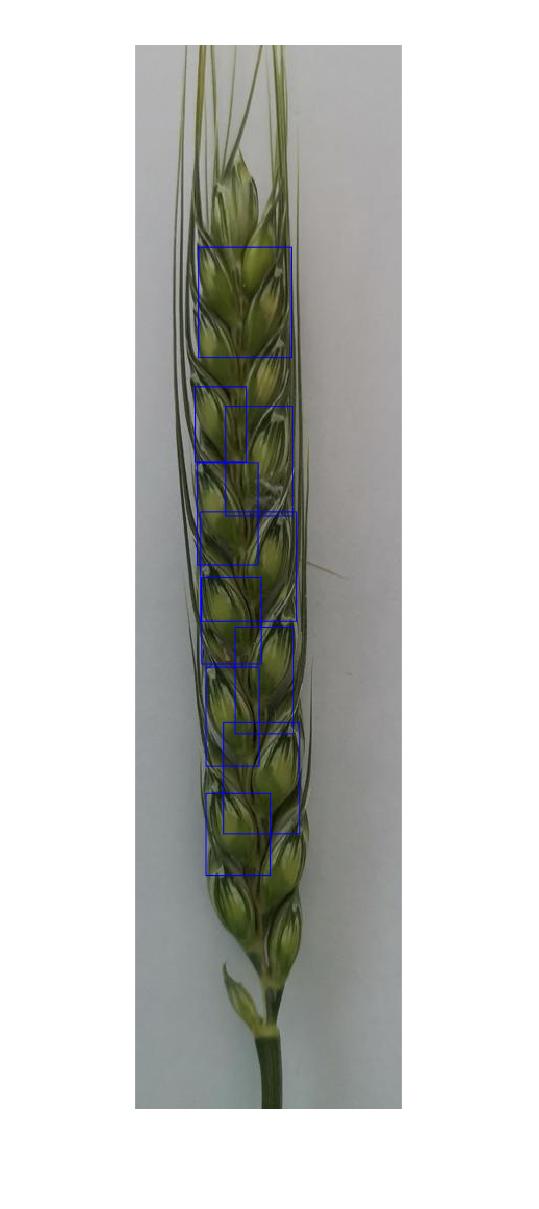

Supplement: Supplementary file 2 [file Data_Sheet_2.zip › 3. Labeling results of watershed algorithm (section Spikelet segmentation and annotation)/Liangxing 99/3189b.jpg]

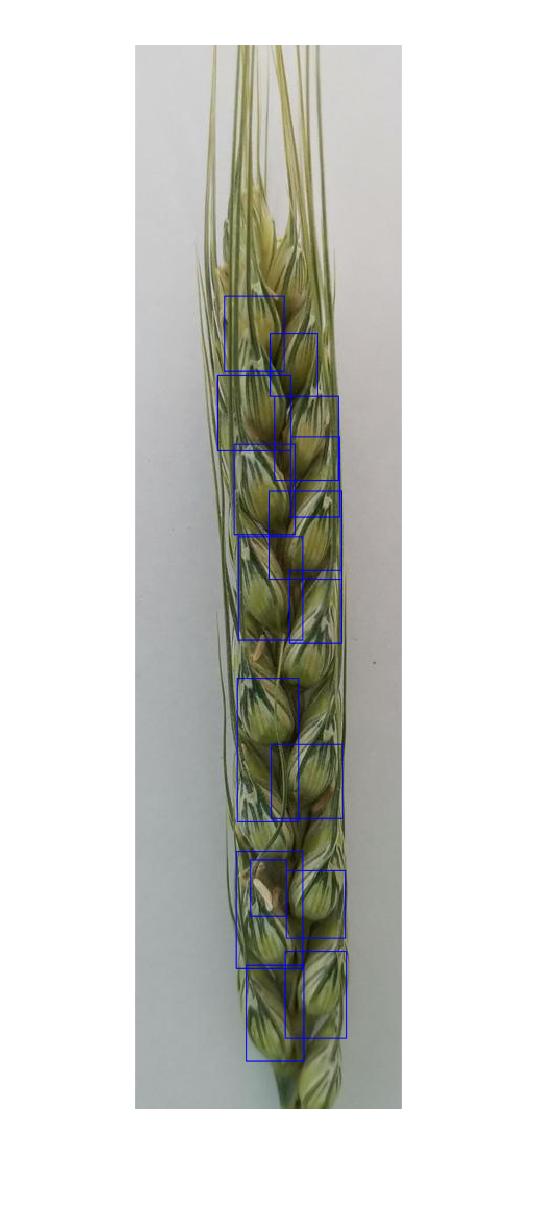

Supplement: Supplementary file 2 [file Data_Sheet_2.zip › 3. Labeling results of watershed algorithm (section Spikelet segmentation and annotation)/Liangxing 99/3190b.jpg]

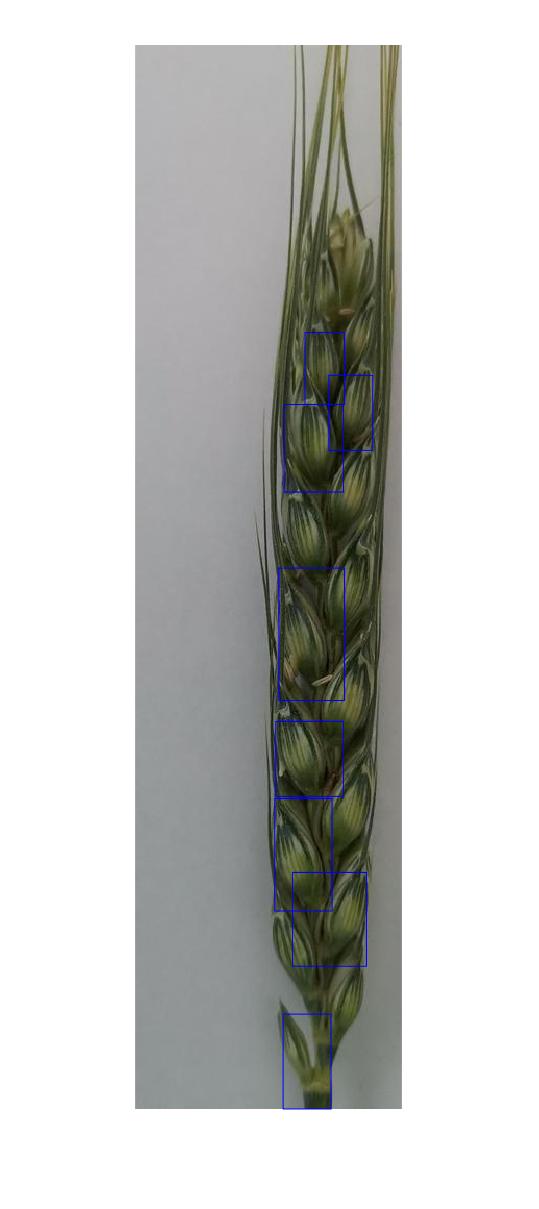

Supplement: Supplementary file 2 [file Data_Sheet_2.zip › 3. Labeling results of watershed algorithm (section Spikelet segmentation and annotation)/Liangxing 99/3193b.jpg]

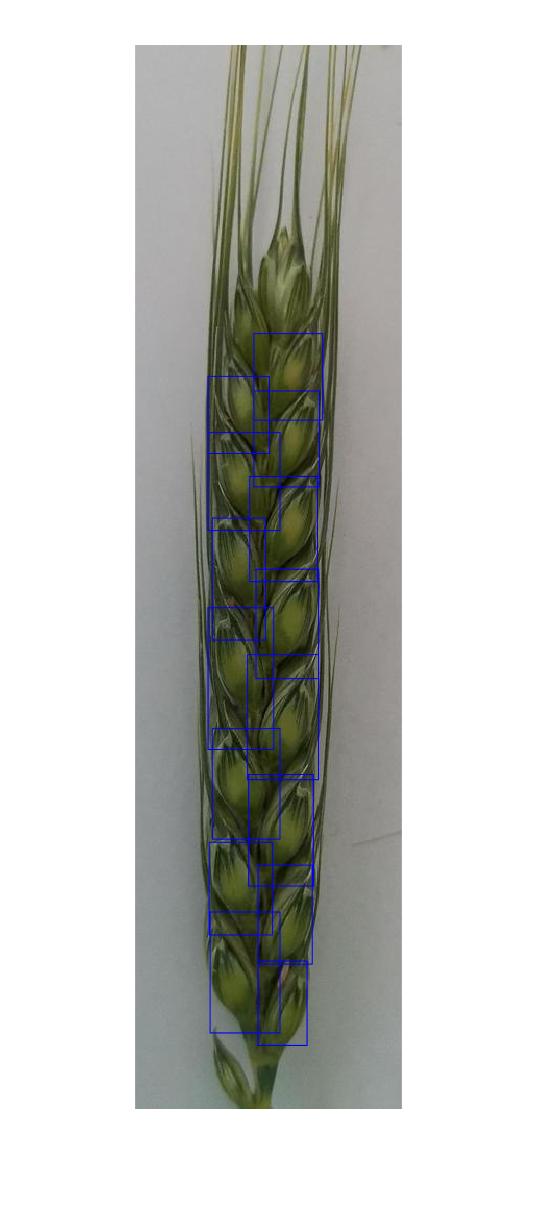

Supplement: Supplementary file 2 [file Data_Sheet_2.zip › 3. Labeling results of watershed algorithm (section Spikelet segmentation and annotation)/Liangxing 99/3195b.jpg]

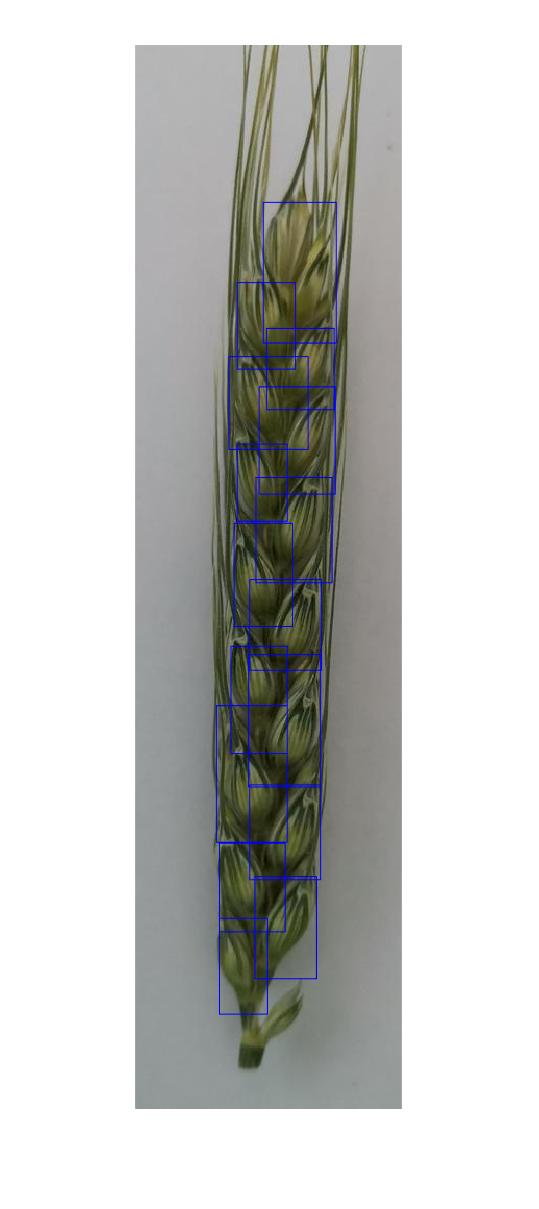

Supplement: Supplementary file 2 [file Data_Sheet_2.zip › 3. Labeling results of watershed algorithm (section Spikelet segmentation and annotation)/Liangxing 99/3204b.jpg]

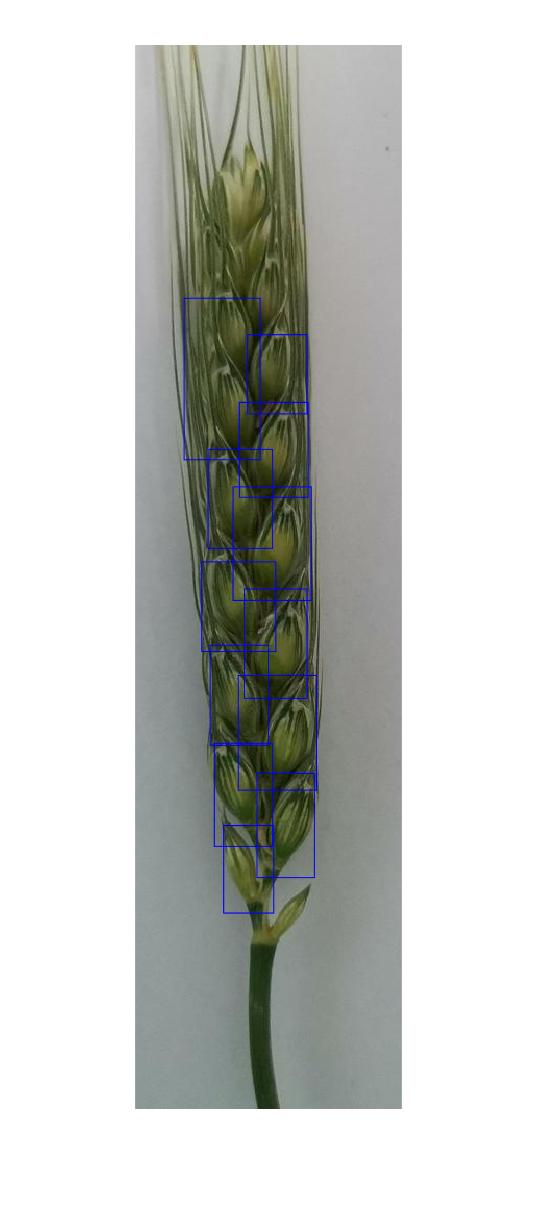

Supplement: Supplementary file 2 [file Data_Sheet_2.zip › 3. Labeling results of watershed algorithm (section Spikelet segmentation and annotation)/Liangxing 99/3205b.jpg]

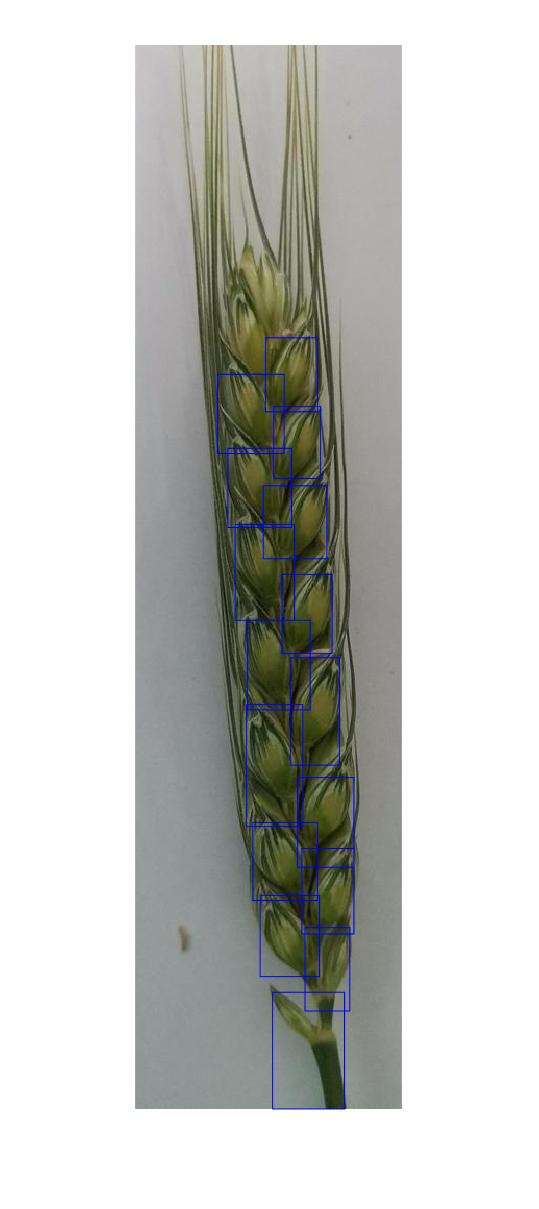

Supplement: Supplementary file 2 [file Data_Sheet_2.zip › 3. Labeling results of watershed algorithm (section Spikelet segmentation and annotation)/Liangxing 99/3206b.jpg]

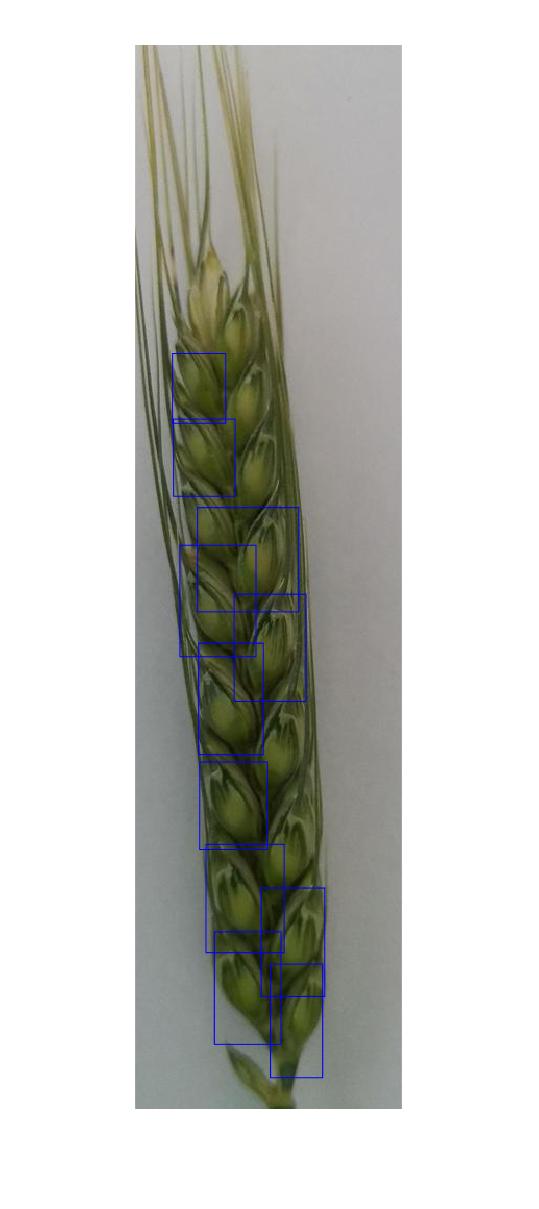

Supplement: Supplementary file 2 [file Data_Sheet_2.zip › 3. Labeling results of watershed algorithm (section Spikelet segmentation and annotation)/Liangxing 99/3209b.jpg]

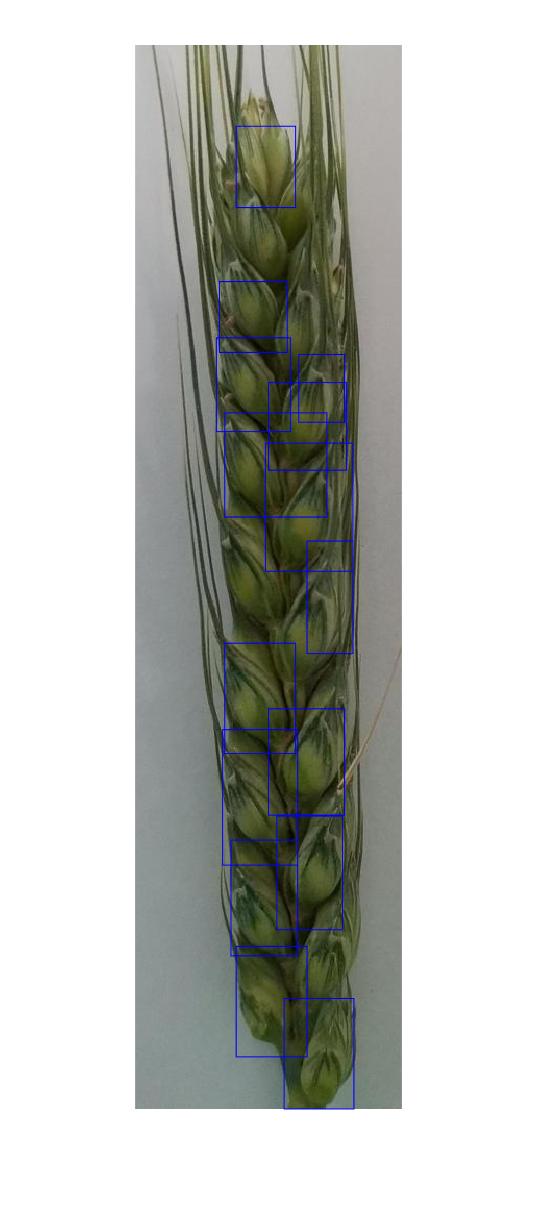

Supplement: Supplementary file 2 [file Data_Sheet_2.zip › 3. Labeling results of watershed algorithm (section Spikelet segmentation and annotation)/Liangxing 99/3217b.jpg]

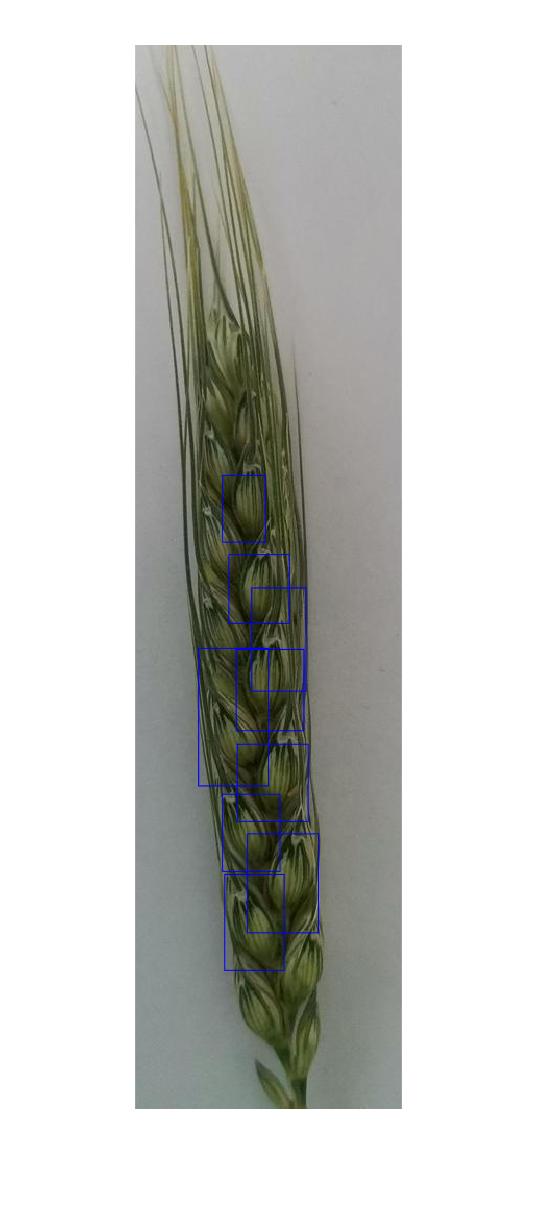

Supplement: Supplementary file 2 [file Data_Sheet_2.zip › 3. Labeling results of watershed algorithm (section Spikelet segmentation and annotation)/Liangxing 99/3219b.jpg]

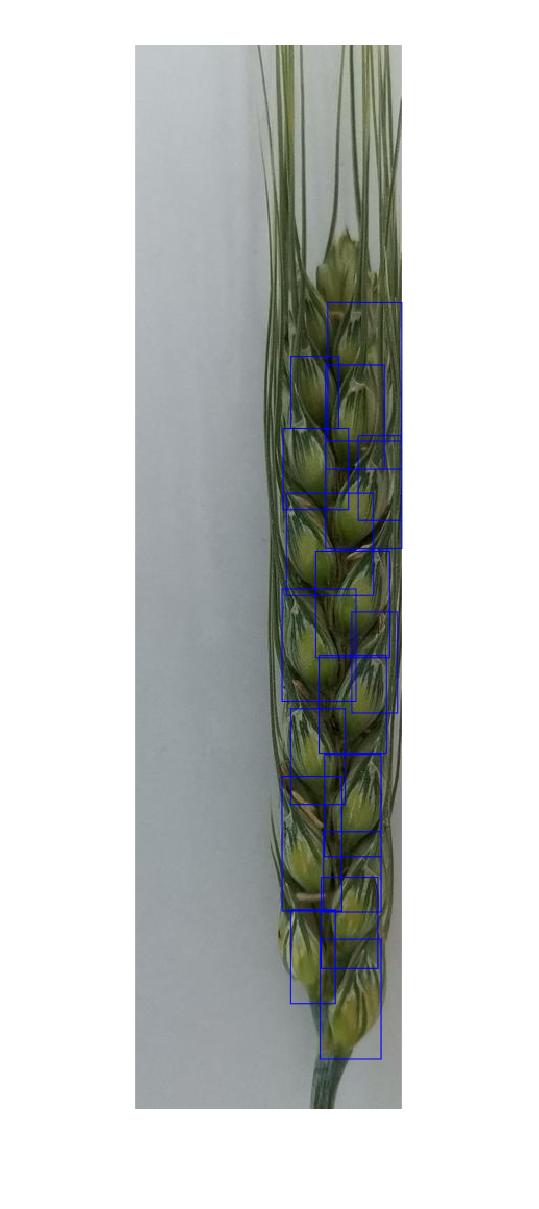

Supplement: Supplementary file 2 [file Data_Sheet_2.zip › 3. Labeling results of watershed algorithm (section Spikelet segmentation and annotation)/Liangxing 99/3223b.jpg]

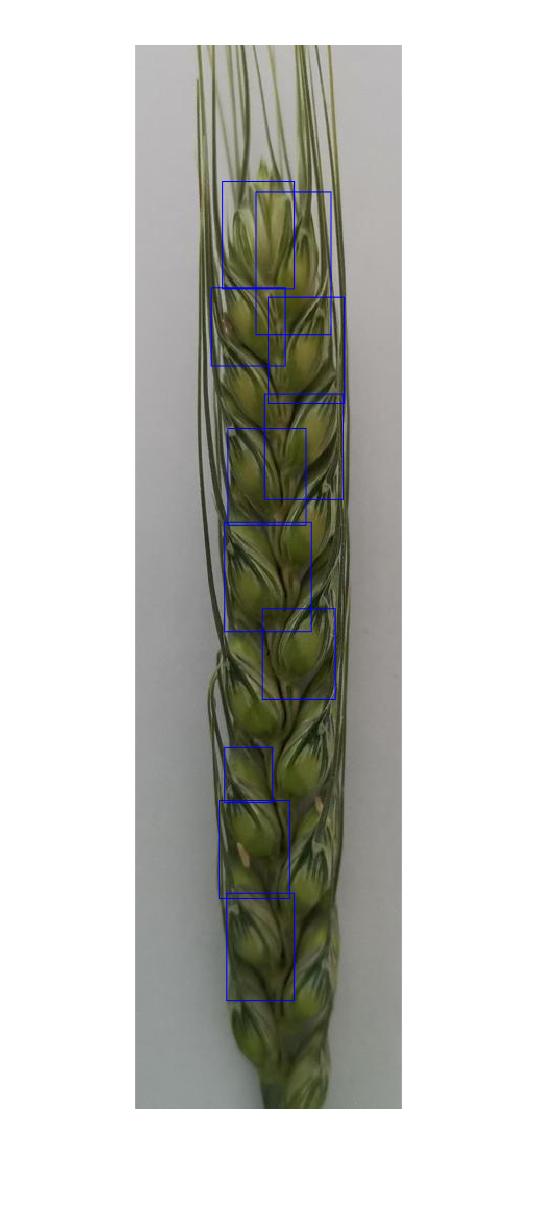

Supplement: Supplementary file 2 [file Data_Sheet_2.zip › 3. Labeling results of watershed algorithm (section Spikelet segmentation and annotation)/Liangxing 99/3225b.jpg]

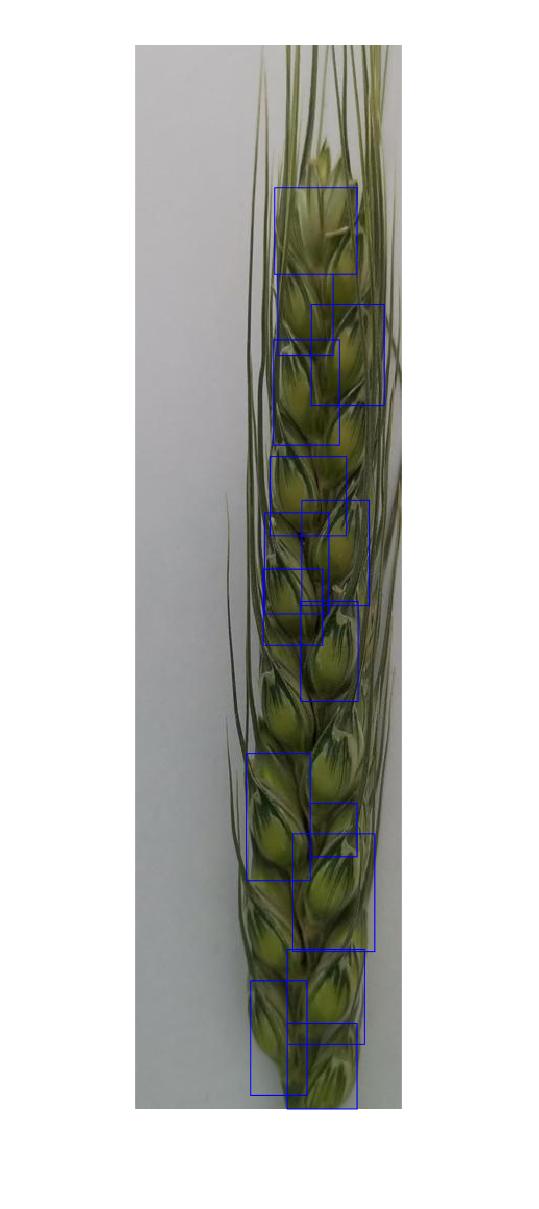

Supplement: Supplementary file 2 [file Data_Sheet_2.zip › 3. Labeling results of watershed algorithm (section Spikelet segmentation and annotation)/Liangxing 99/3227b.jpg]

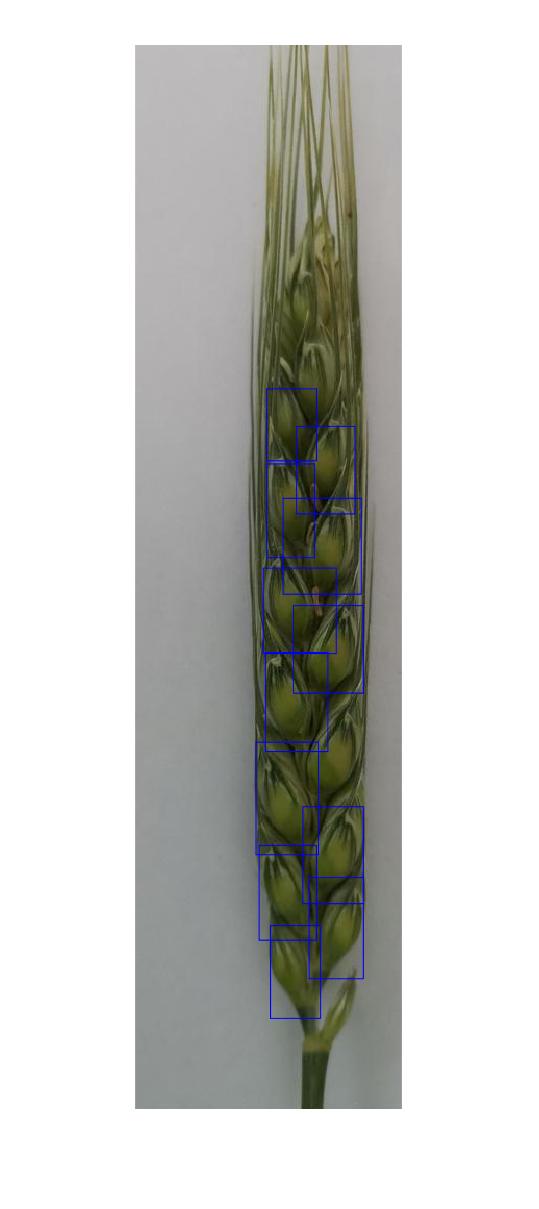

Supplement: Supplementary file 2 [file Data_Sheet_2.zip › 3. Labeling results of watershed algorithm (section Spikelet segmentation and annotation)/Liangxing 99/3230b.jpg]

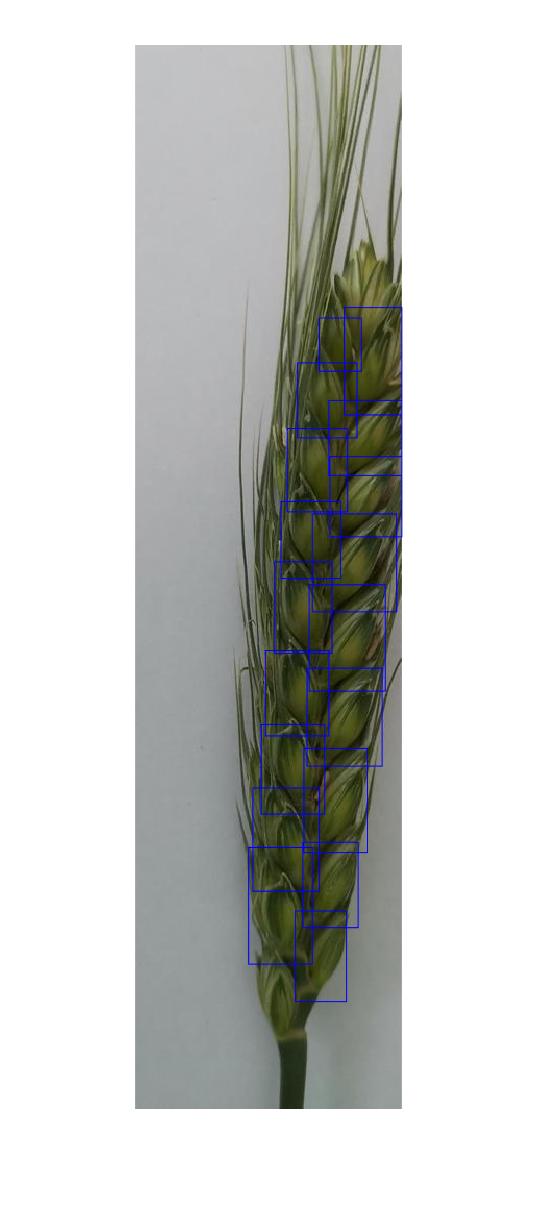

Supplement: Supplementary file 2 [file Data_Sheet_2.zip › 3. Labeling results of watershed algorithm (section Spikelet segmentation and annotation)/Liangxing 99/3233b.jpg]

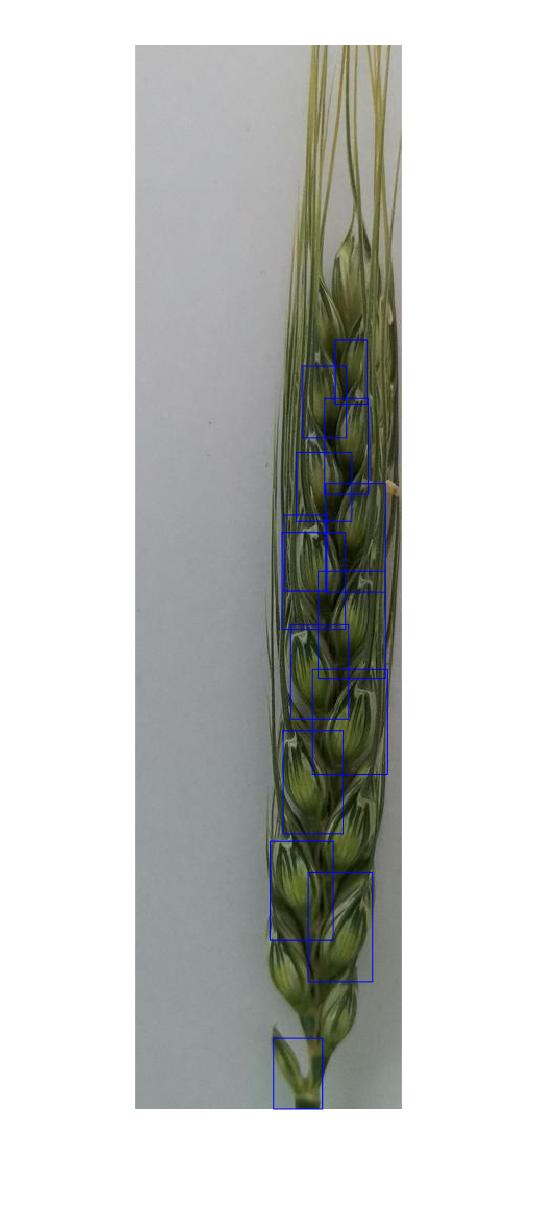

Supplement: Supplementary file 2 [file Data_Sheet_2.zip › 3. Labeling results of watershed algorithm (section Spikelet segmentation and annotation)/Liangxing 99/3235b.jpg]

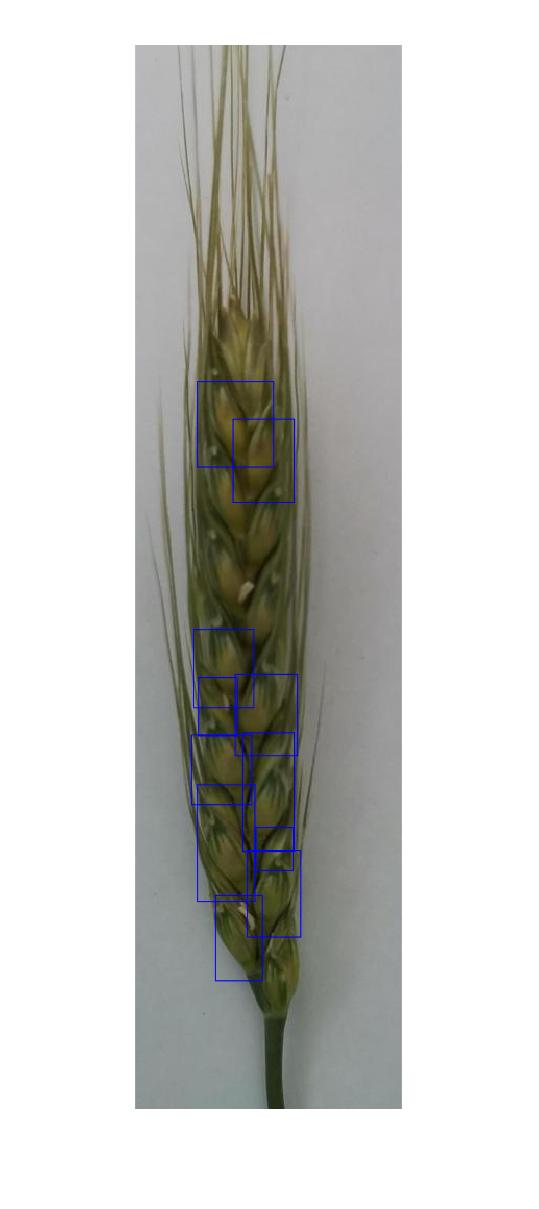

Supplement: Supplementary file 2 [file Data_Sheet_2.zip › 3. Labeling results of watershed algorithm (section Spikelet segmentation and annotation)/Liangxing 99/3238b.jpg]

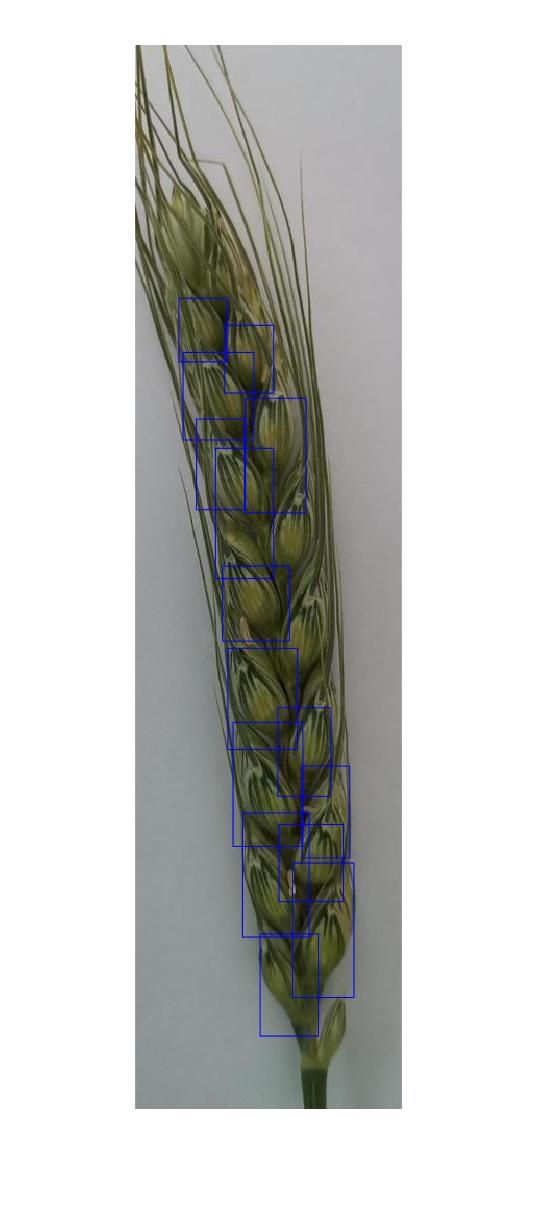

Supplement: Supplementary file 2 [file Data_Sheet_2.zip › 3. Labeling results of watershed algorithm (section Spikelet segmentation and annotation)/Liangxing 99/3241b.jpg]

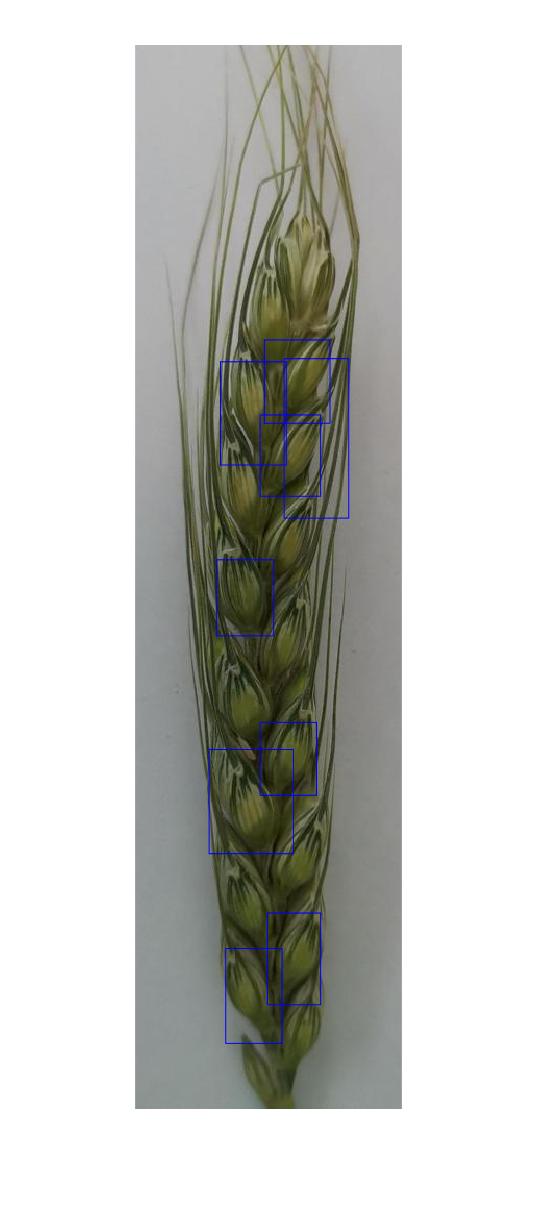

Supplement: Supplementary file 2 [file Data_Sheet_2.zip › 3. Labeling results of watershed algorithm (section Spikelet segmentation and annotation)/Liangxing 99/3242b.jpg]

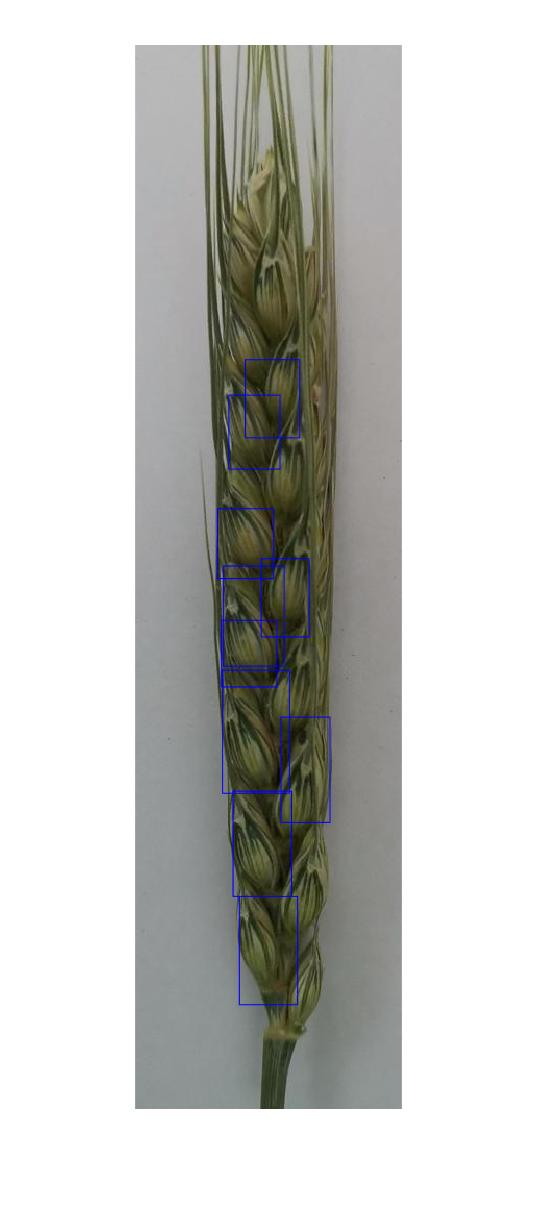

Supplement: Supplementary file 2 [file Data_Sheet_2.zip › 3. Labeling results of watershed algorithm (section Spikelet segmentation and annotation)/Liangxing 99/3247b.jpg]

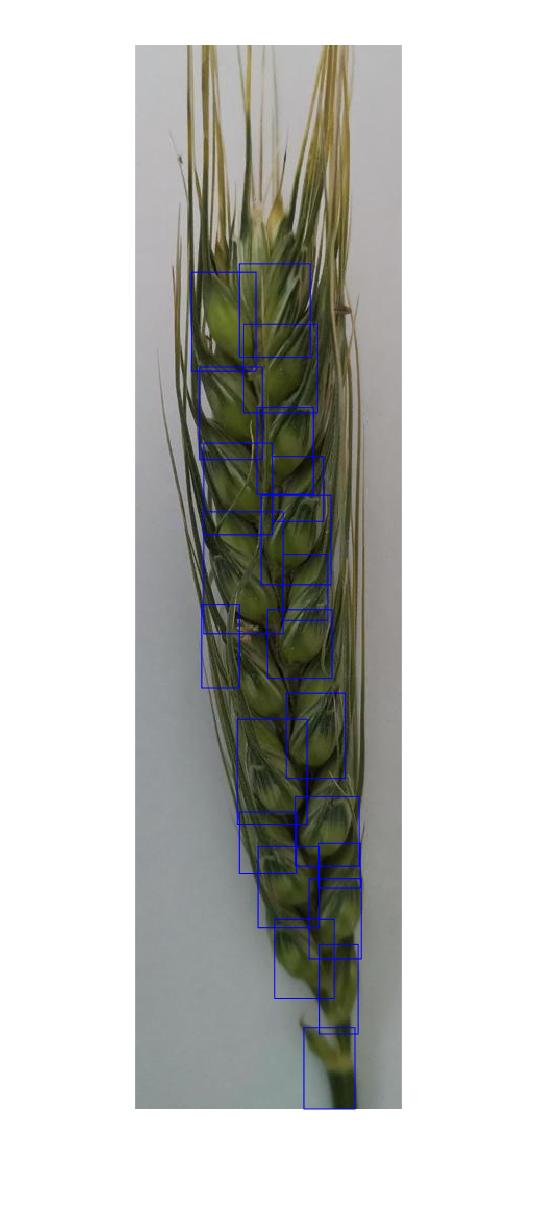

Supplement: Supplementary file 2 [file Data_Sheet_2.zip › 3. Labeling results of watershed algorithm (section Spikelet segmentation and annotation)/Liangxing 99/3252b.jpg]

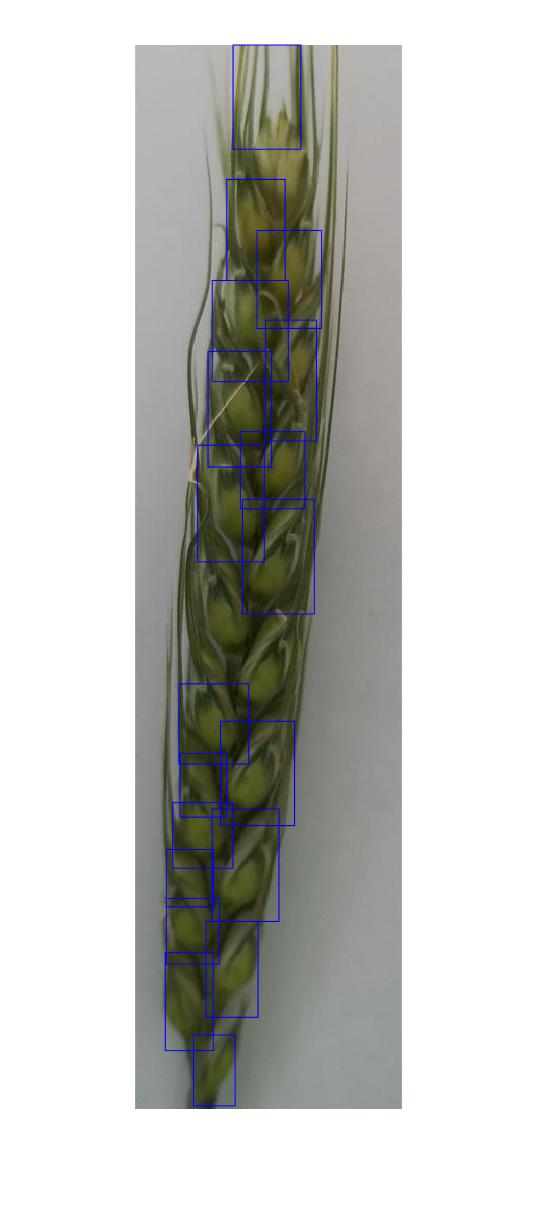

Supplement: Supplementary file 2 [file Data_Sheet_2.zip › 3. Labeling results of watershed algorithm (section Spikelet segmentation and annotation)/Liangxing 99/3253b.jpg]

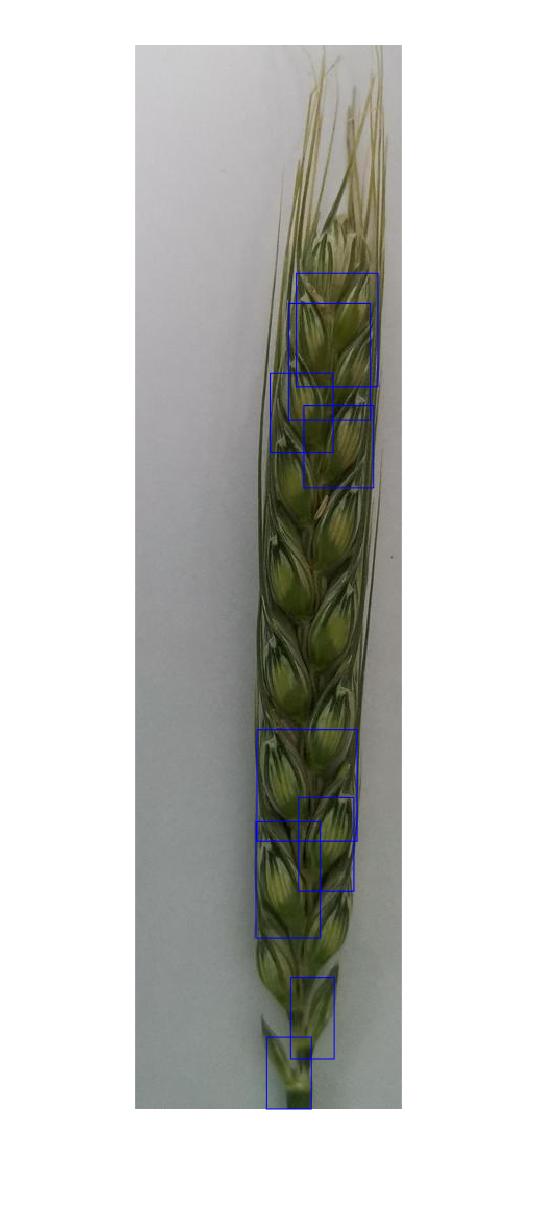

Supplement: Supplementary file 2 [file Data_Sheet_2.zip › 3. Labeling results of watershed algorithm (section Spikelet segmentation and annotation)/Liangxing 99/3262b.jpg]

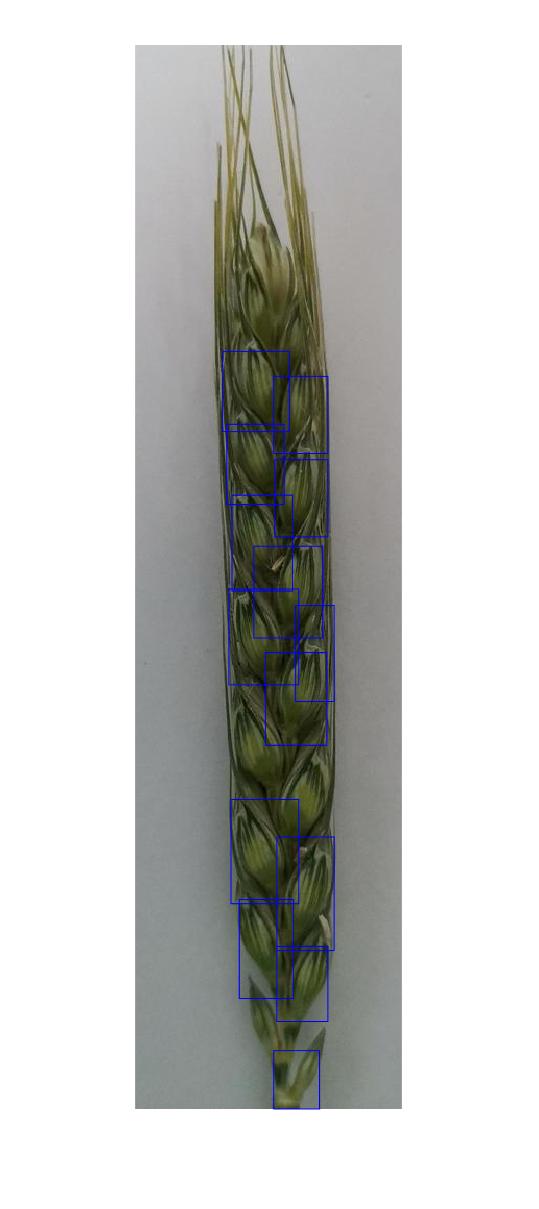

Supplement: Supplementary file 2 [file Data_Sheet_2.zip › 3. Labeling results of watershed algorithm (section Spikelet segmentation and annotation)/Liangxing 99/3263b.jpg]

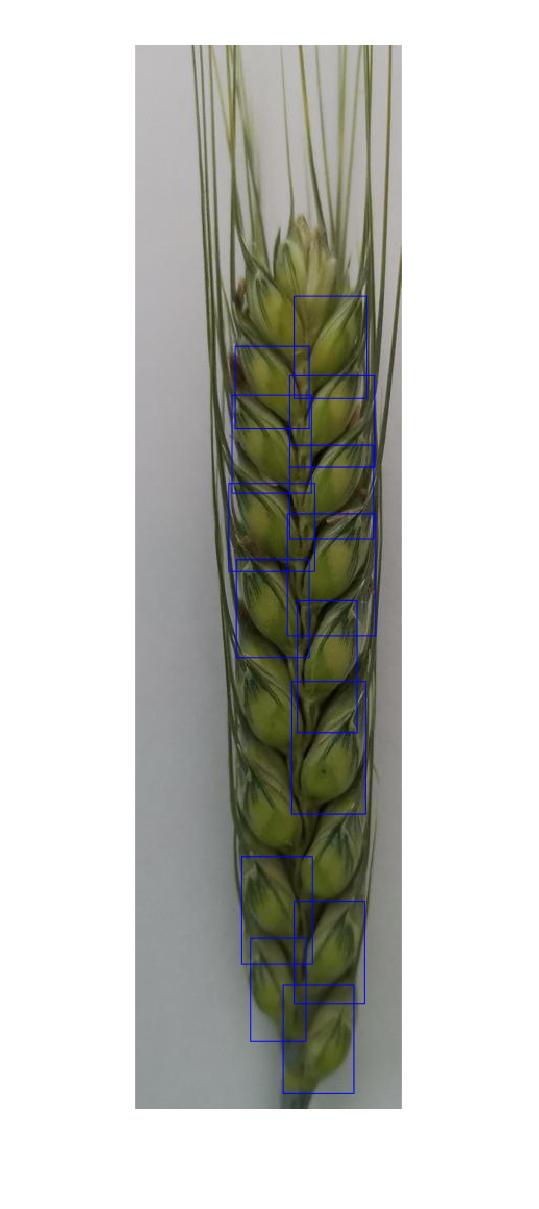

Supplement: Supplementary file 2 [file Data_Sheet_2.zip › 3. Labeling results of watershed algorithm (section Spikelet segmentation and annotation)/Liangxing 99/3264b.jpg]

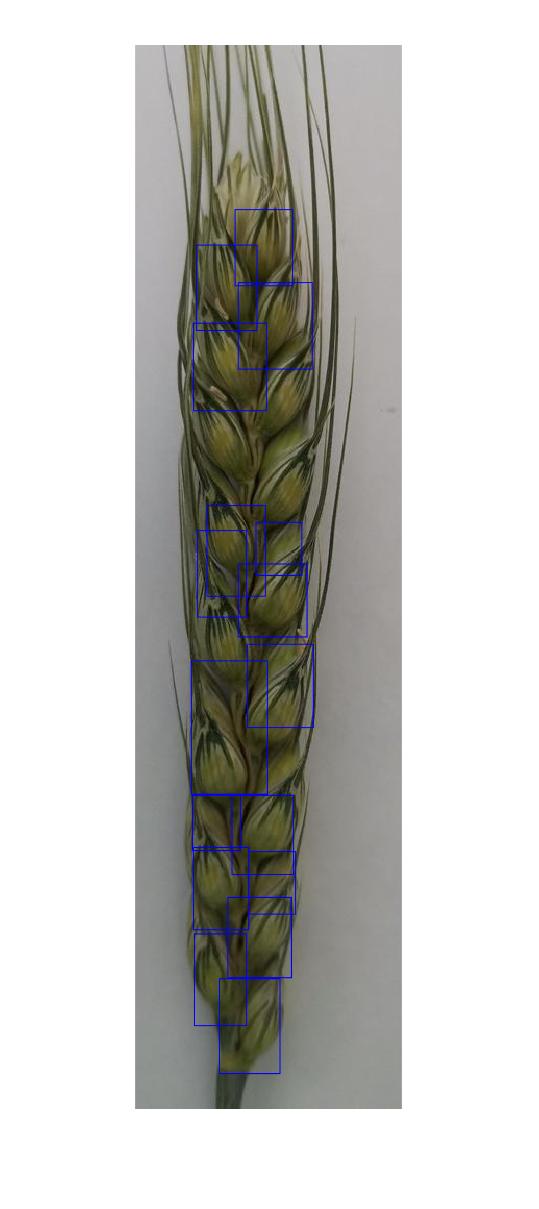

Supplement: Supplementary file 2 [file Data_Sheet_2.zip › 3. Labeling results of watershed algorithm (section Spikelet segmentation and annotation)/Liangxing 99/3278b.jpg]

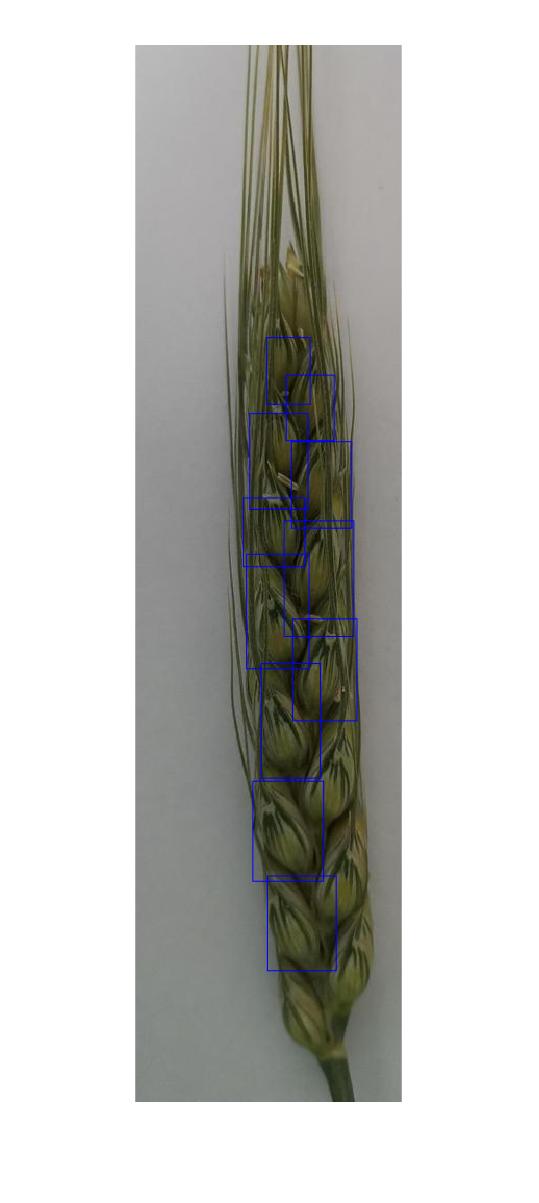

Supplement: Supplementary file 2 [file Data_Sheet_2.zip › 3. Labeling results of watershed algorithm (section Spikelet segmentation and annotation)/Liangxing 99/3282b.jpg]

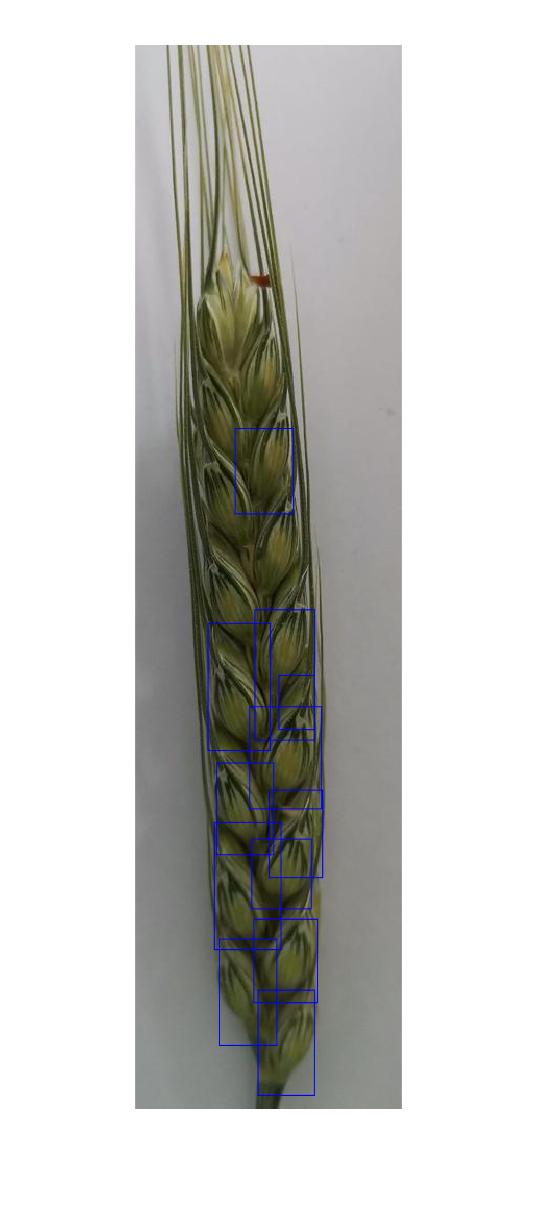

Supplement: Supplementary file 2 [file Data_Sheet_2.zip › 3. Labeling results of watershed algorithm (section Spikelet segmentation and annotation)/Liangxing 99/3283b.jpg]

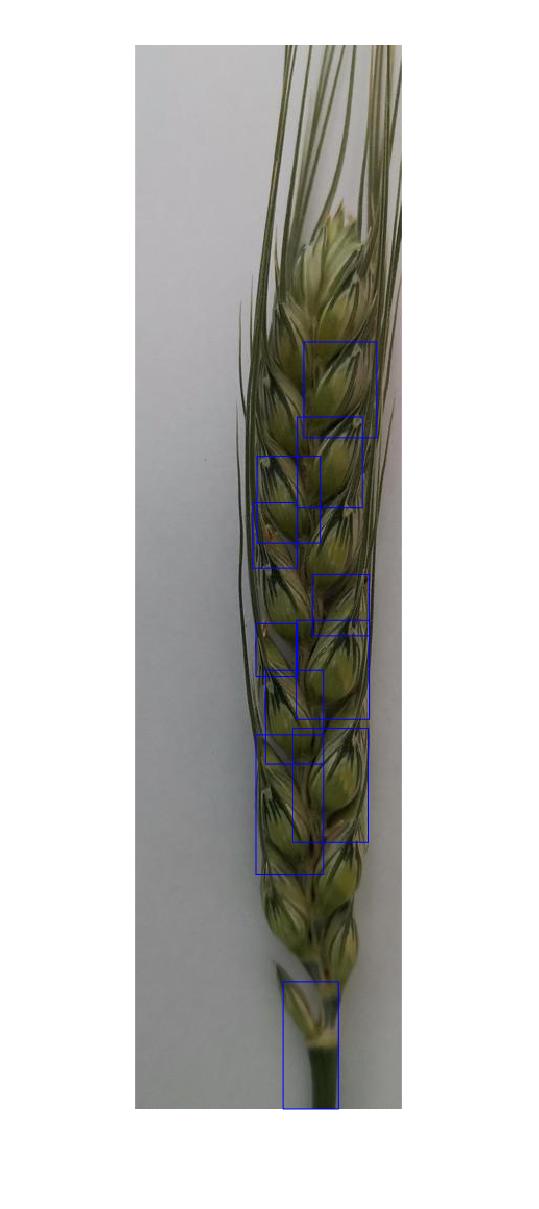

Supplement: Supplementary file 2 [file Data_Sheet_2.zip › 3. Labeling results of watershed algorithm (section Spikelet segmentation and annotation)/Liangxing 99/3295b.jpg]

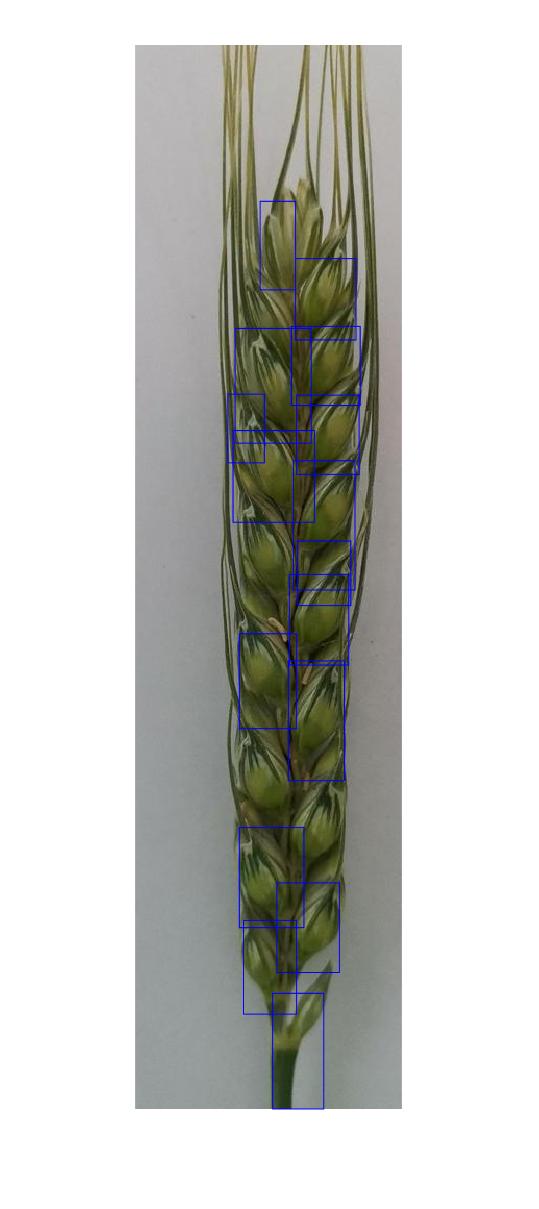

Supplement: Supplementary file 2 [file Data_Sheet_2.zip › 3. Labeling results of watershed algorithm (section Spikelet segmentation and annotation)/Liangxing 99/3301b.jpg]

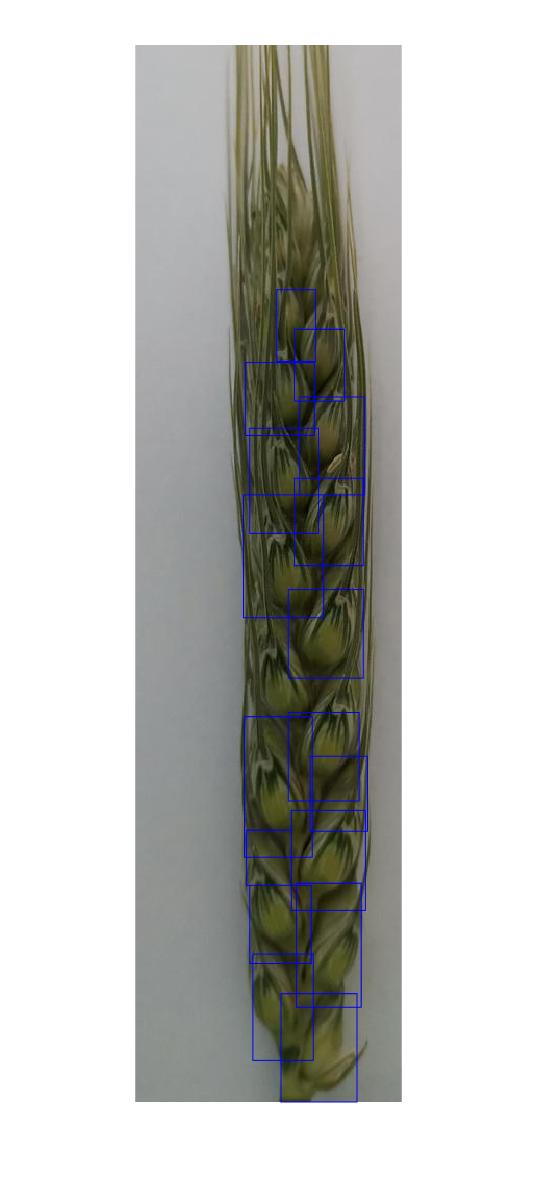

Supplement: Supplementary file 2 [file Data_Sheet_2.zip › 3. Labeling results of watershed algorithm (section Spikelet segmentation and annotation)/Liangxing 99/3310b.jpg]

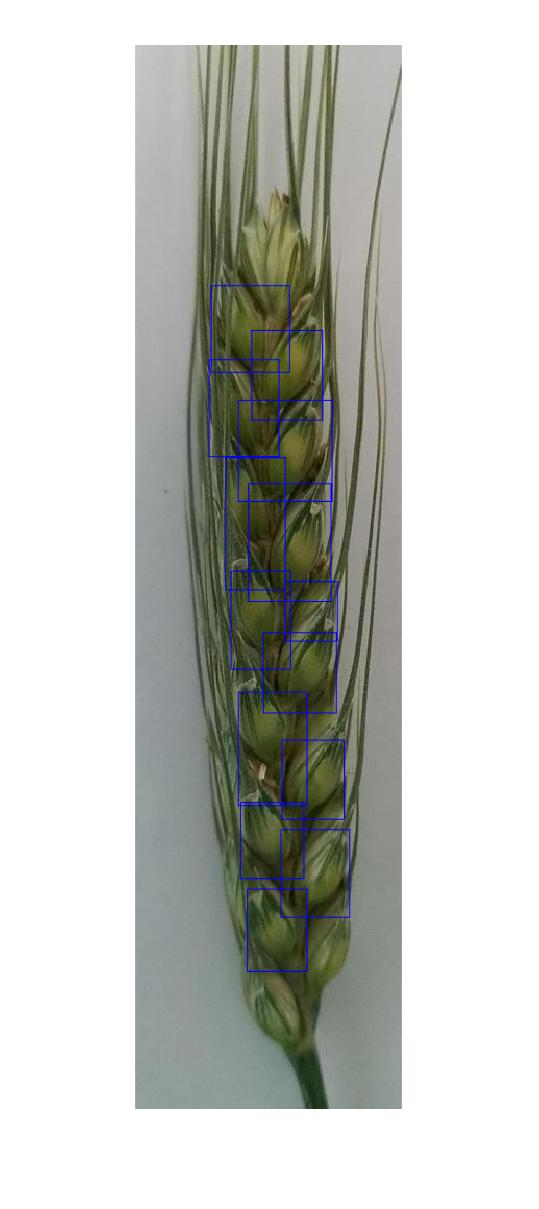

Supplement: Supplementary file 2 [file Data_Sheet_2.zip › 3. Labeling results of watershed algorithm (section Spikelet segmentation and annotation)/Liangxing 99/3327b.jpg]

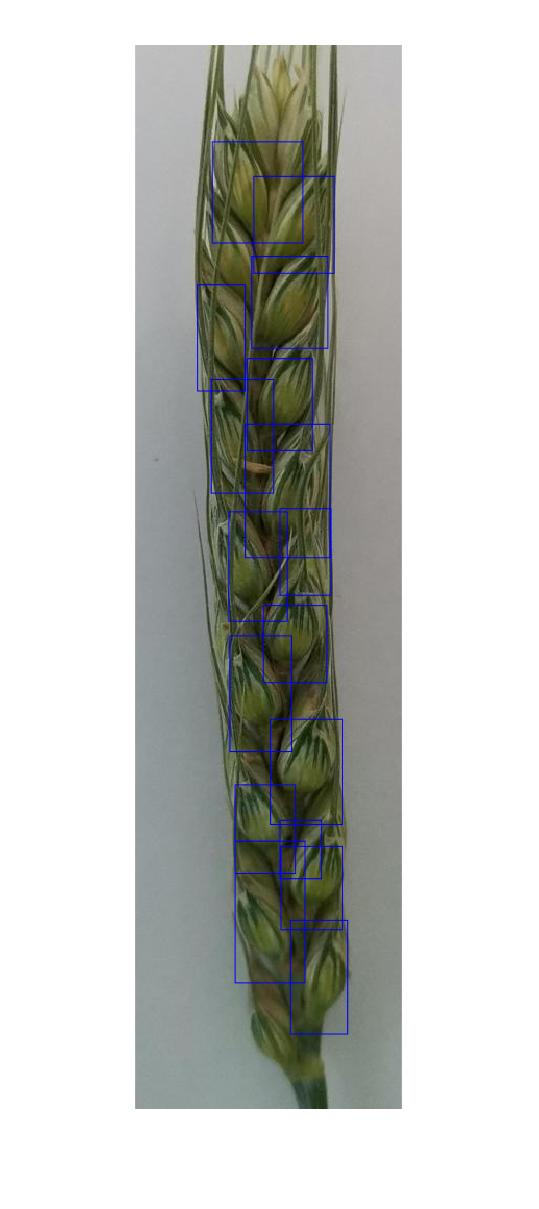

Supplement: Supplementary file 2 [file Data_Sheet_2.zip › 3. Labeling results of watershed algorithm (section Spikelet segmentation and annotation)/Liangxing 99/3328b.jpg]

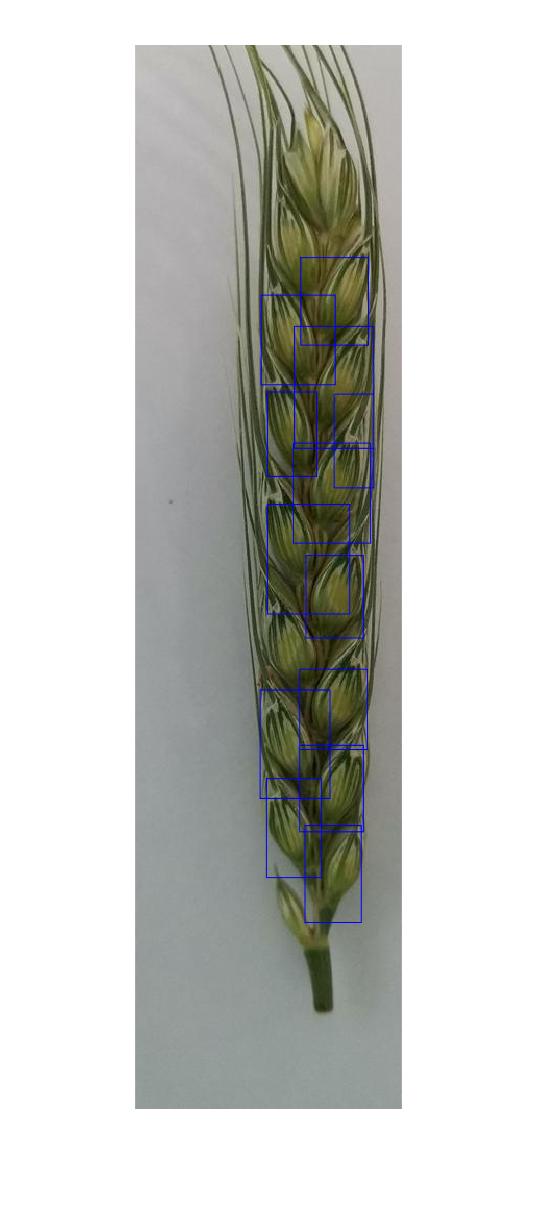

Supplement: Supplementary file 2 [file Data_Sheet_2.zip › 3. Labeling results of watershed algorithm (section Spikelet segmentation and annotation)/Liangxing 99/3329b.jpg]

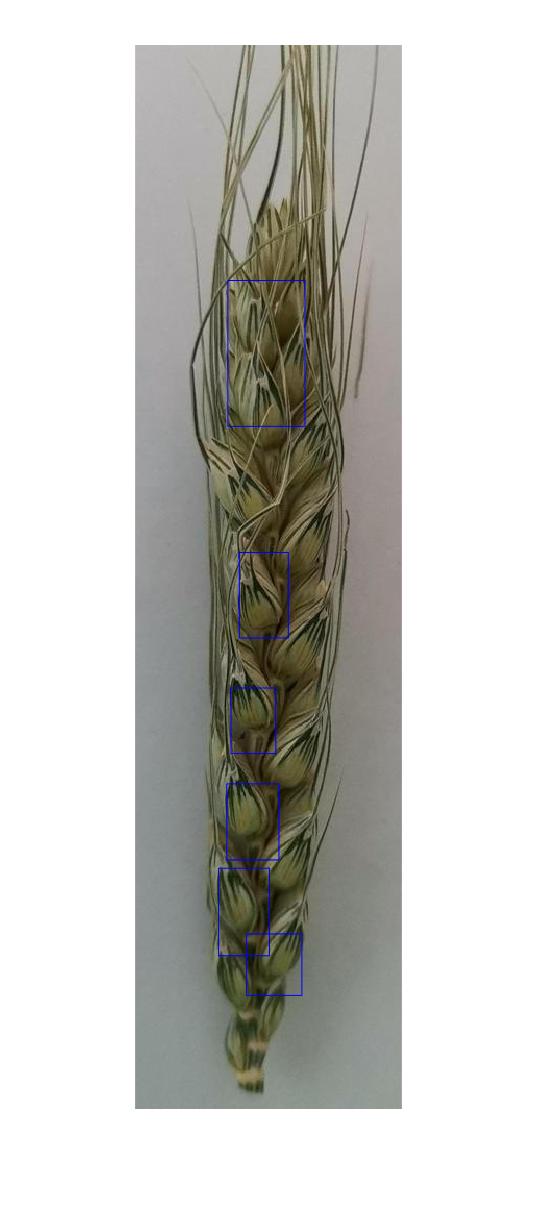

Supplement: Supplementary file 2 [file Data_Sheet_2.zip › 3. Labeling results of watershed algorithm (section Spikelet segmentation and annotation)/Liangxing 99/3333b.jpg]
