# Supplementary figures and images for: Automatic Detection and Counting of Wheat Spikelet Using Semi-Automatic Labeling and Deep Learning (part 5 of 8)
Source: Front Plant Sci. 2022 May 30;13:872555. doi: 10.3389/fpls.2022.872555 (PMC9189412; doi:10.3389/fpls.2022.872555)

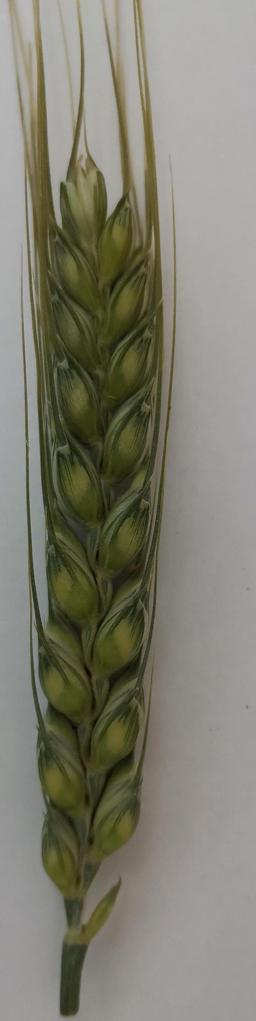

Supplement: Supplementary file 3 [file Data_Sheet_3.zip › 4. Datasets for first model training (section DCNN model training)/training dataset/1003.jpg]

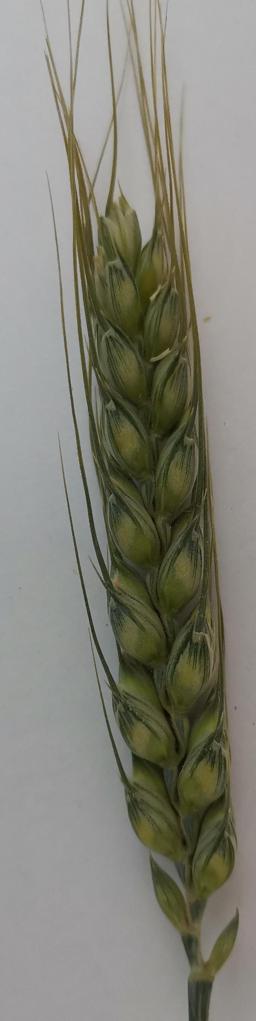

Supplement: Supplementary file 3 [file Data_Sheet_3.zip › 4. Datasets for first model training (section DCNN model training)/training dataset/1005.jpg]

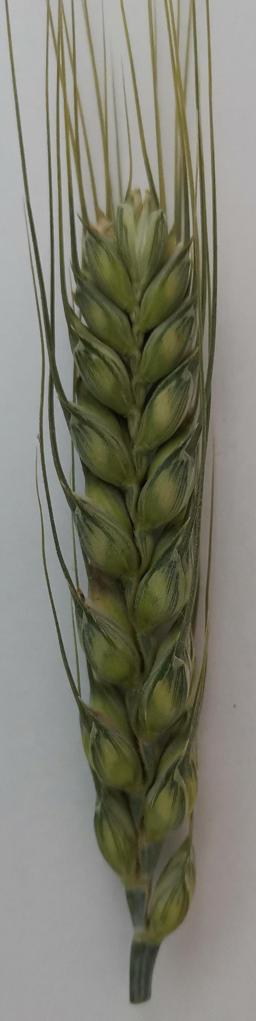

Supplement: Supplementary file 3 [file Data_Sheet_3.zip › 4. Datasets for first model training (section DCNN model training)/training dataset/1006.jpg]

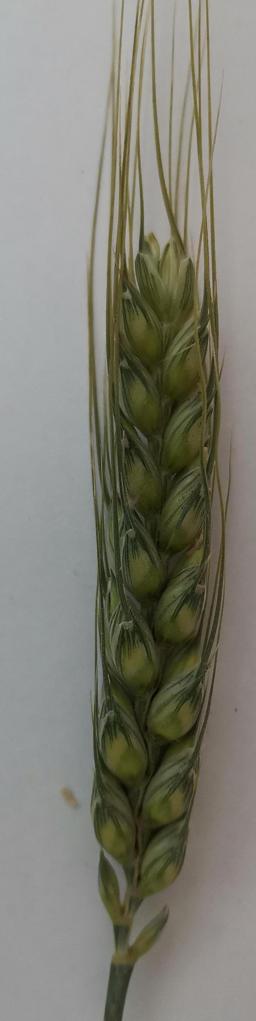

Supplement: Supplementary file 3 [file Data_Sheet_3.zip › 4. Datasets for first model training (section DCNN model training)/training dataset/1009.jpg]

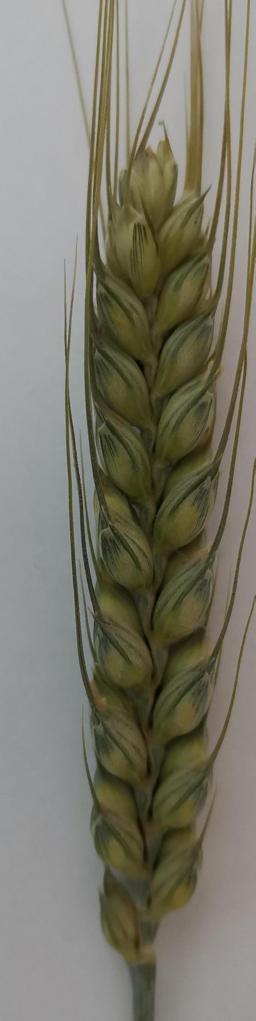

Supplement: Supplementary file 3 [file Data_Sheet_3.zip › 4. Datasets for first model training (section DCNN model training)/training dataset/1014.jpg]

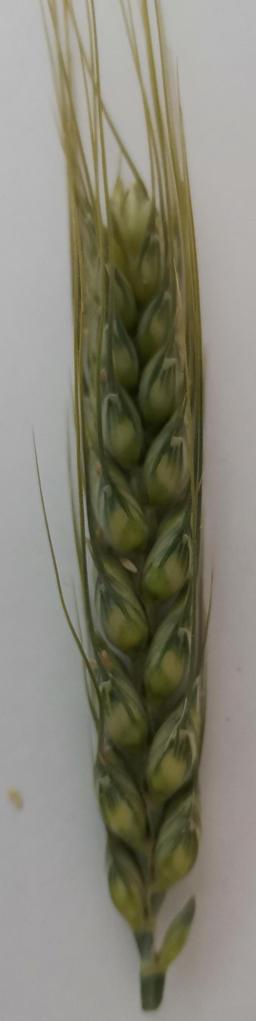

Supplement: Supplementary file 3 [file Data_Sheet_3.zip › 4. Datasets for first model training (section DCNN model training)/training dataset/1015.jpg]

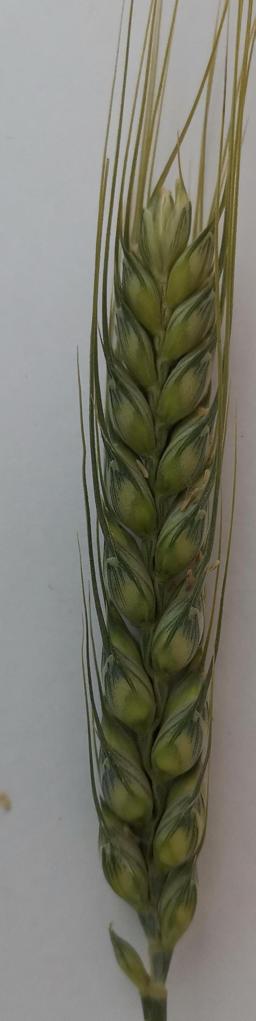

Supplement: Supplementary file 3 [file Data_Sheet_3.zip › 4. Datasets for first model training (section DCNN model training)/training dataset/1016.jpg]

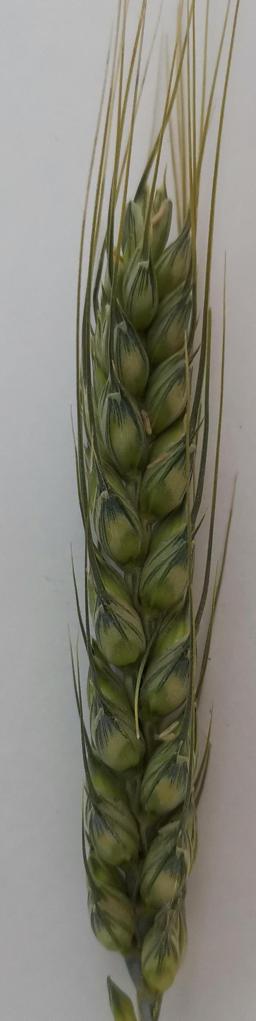

Supplement: Supplementary file 3 [file Data_Sheet_3.zip › 4. Datasets for first model training (section DCNN model training)/training dataset/1017.jpg]

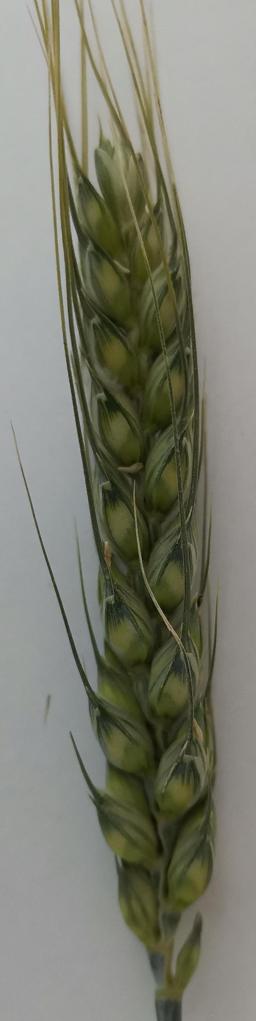

Supplement: Supplementary file 3 [file Data_Sheet_3.zip › 4. Datasets for first model training (section DCNN model training)/training dataset/1018.jpg]

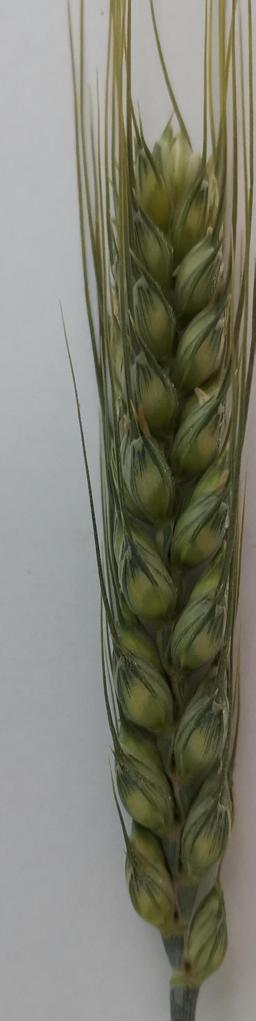

Supplement: Supplementary file 3 [file Data_Sheet_3.zip › 4. Datasets for first model training (section DCNN model training)/training dataset/1021.jpg]

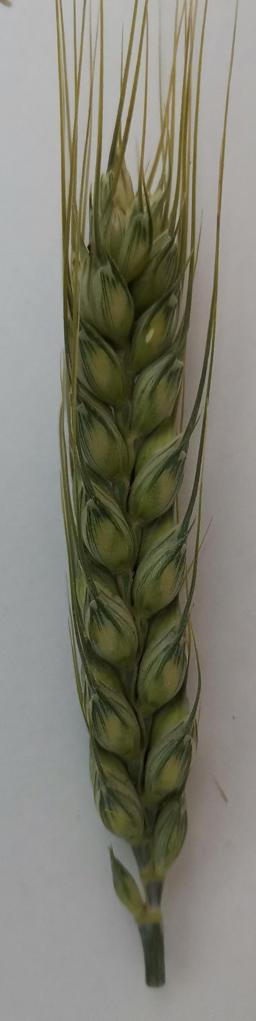

Supplement: Supplementary file 3 [file Data_Sheet_3.zip › 4. Datasets for first model training (section DCNN model training)/training dataset/1027.jpg]

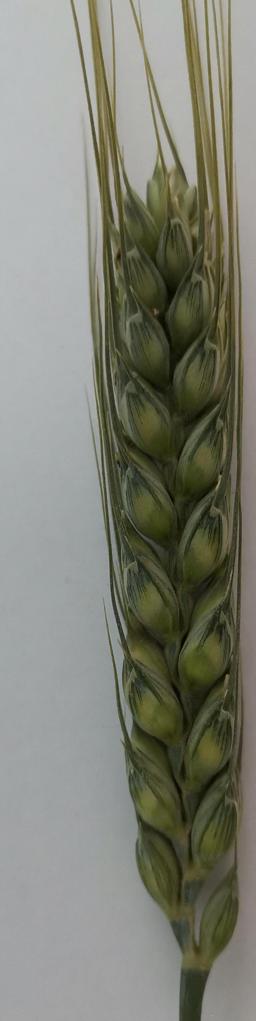

Supplement: Supplementary file 3 [file Data_Sheet_3.zip › 4. Datasets for first model training (section DCNN model training)/training dataset/1033.jpg]

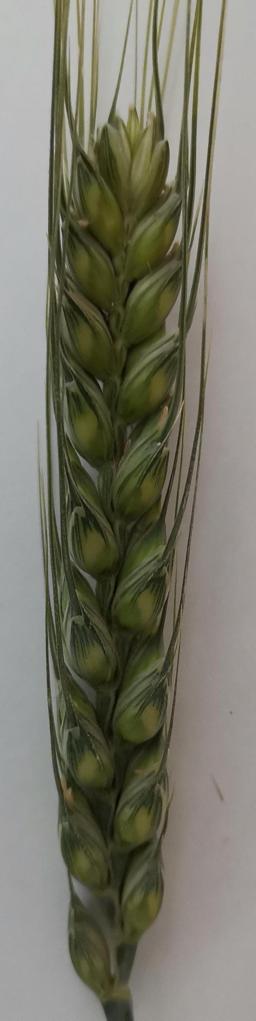

Supplement: Supplementary file 3 [file Data_Sheet_3.zip › 4. Datasets for first model training (section DCNN model training)/training dataset/1036.jpg]

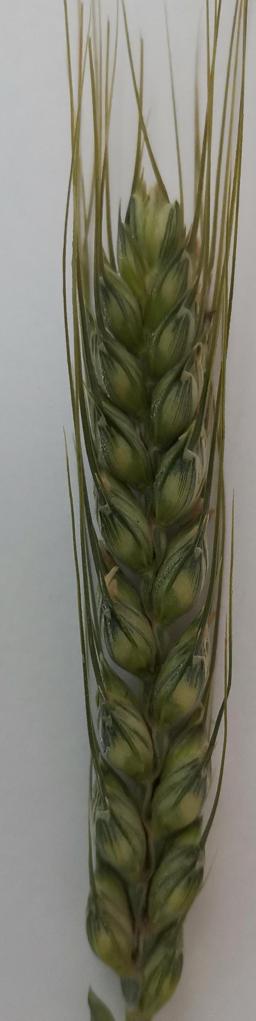

Supplement: Supplementary file 3 [file Data_Sheet_3.zip › 4. Datasets for first model training (section DCNN model training)/training dataset/1037.jpg]

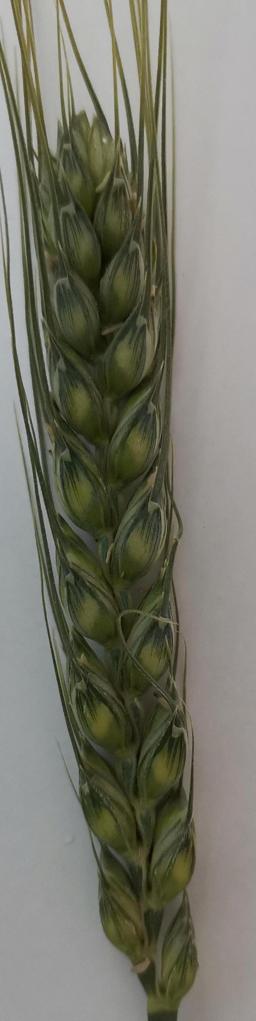

Supplement: Supplementary file 3 [file Data_Sheet_3.zip › 4. Datasets for first model training (section DCNN model training)/training dataset/1040.jpg]

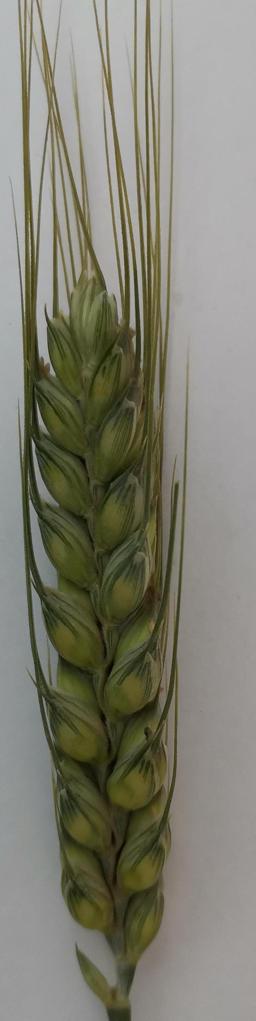

Supplement: Supplementary file 3 [file Data_Sheet_3.zip › 4. Datasets for first model training (section DCNN model training)/training dataset/1043.jpg]

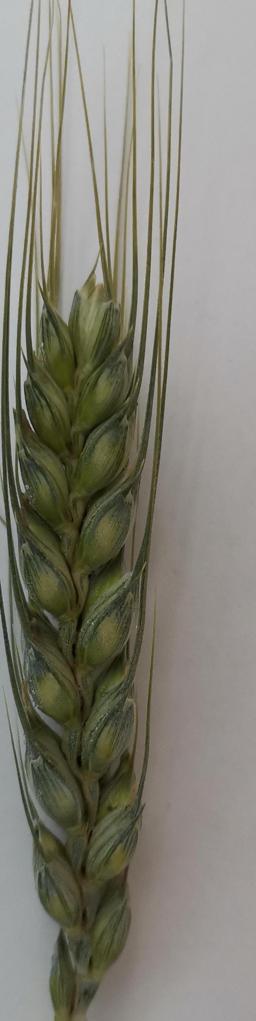

Supplement: Supplementary file 3 [file Data_Sheet_3.zip › 4. Datasets for first model training (section DCNN model training)/training dataset/1048.jpg]

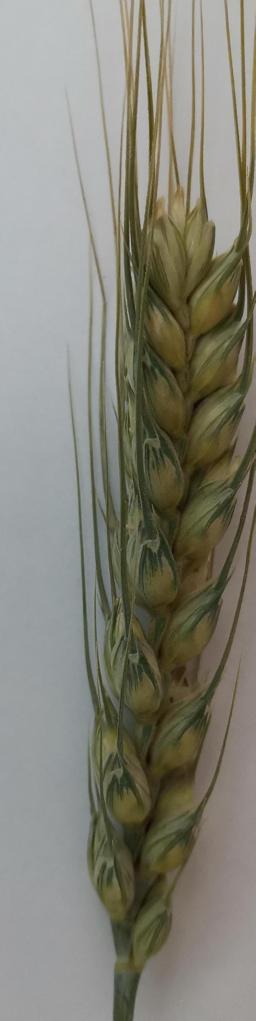

Supplement: Supplementary file 3 [file Data_Sheet_3.zip › 4. Datasets for first model training (section DCNN model training)/training dataset/1050.jpg]

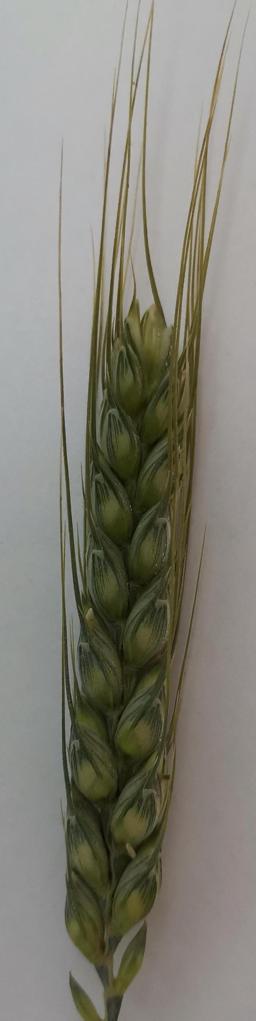

Supplement: Supplementary file 3 [file Data_Sheet_3.zip › 4. Datasets for first model training (section DCNN model training)/training dataset/1051.jpg]

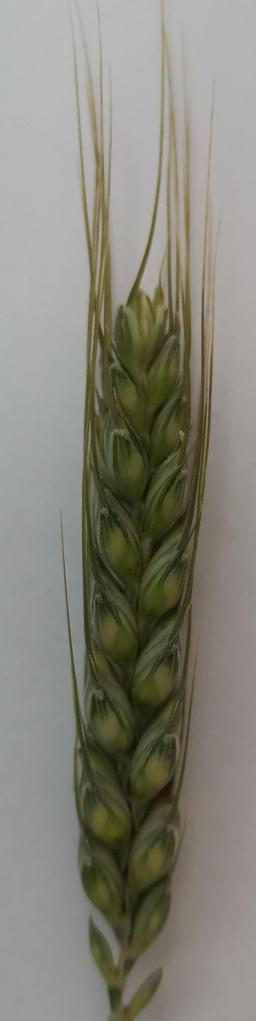

Supplement: Supplementary file 3 [file Data_Sheet_3.zip › 4. Datasets for first model training (section DCNN model training)/training dataset/1052.jpg]

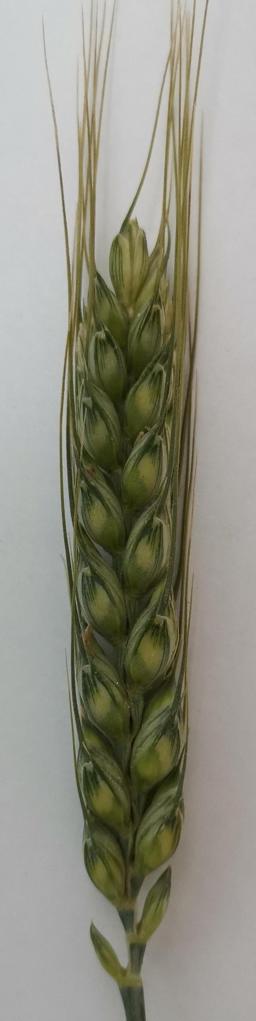

Supplement: Supplementary file 3 [file Data_Sheet_3.zip › 4. Datasets for first model training (section DCNN model training)/training dataset/1053.jpg]

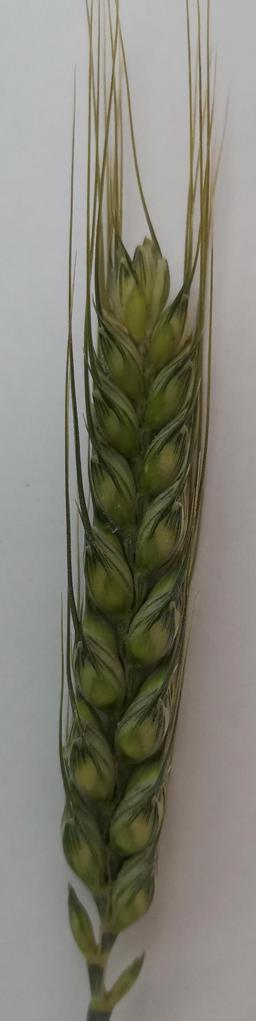

Supplement: Supplementary file 3 [file Data_Sheet_3.zip › 4. Datasets for first model training (section DCNN model training)/training dataset/1054.jpg]

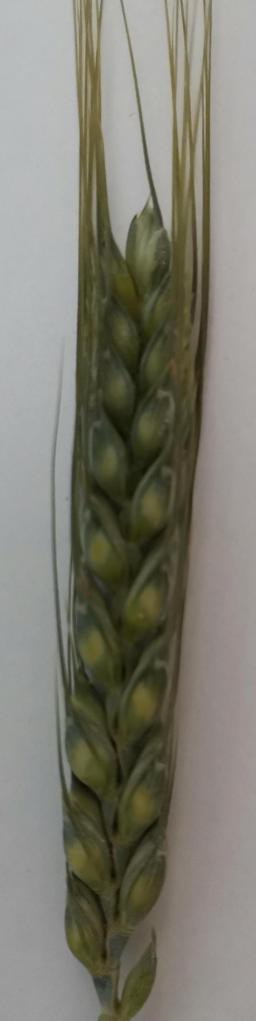

Supplement: Supplementary file 3 [file Data_Sheet_3.zip › 4. Datasets for first model training (section DCNN model training)/training dataset/1055.jpg]

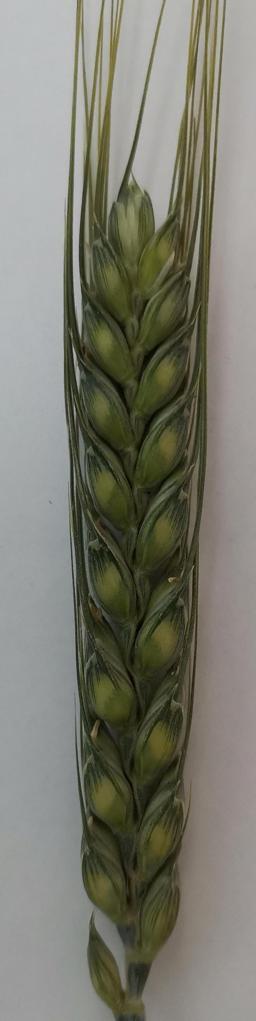

Supplement: Supplementary file 3 [file Data_Sheet_3.zip › 4. Datasets for first model training (section DCNN model training)/training dataset/1056.jpg]

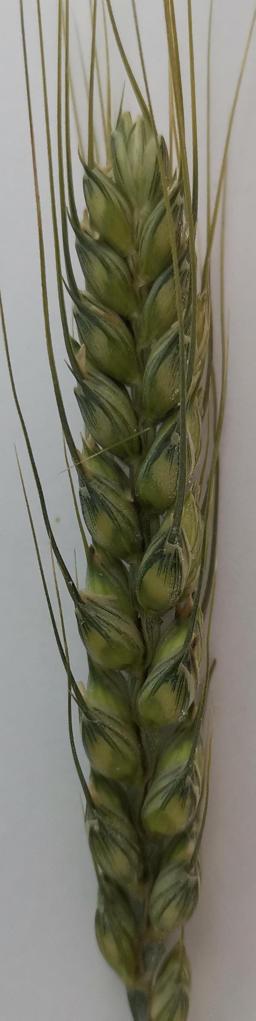

Supplement: Supplementary file 3 [file Data_Sheet_3.zip › 4. Datasets for first model training (section DCNN model training)/training dataset/1057.jpg]

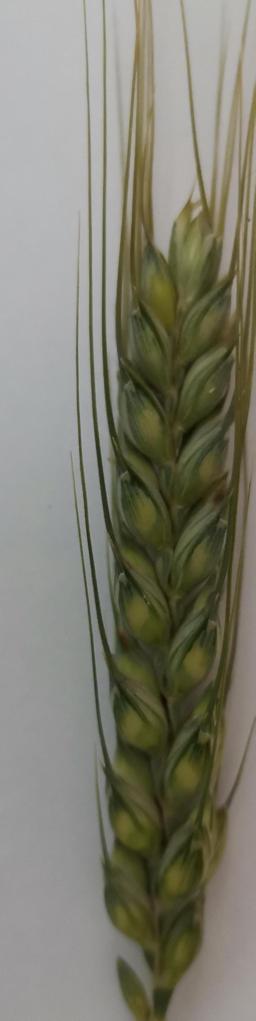

Supplement: Supplementary file 3 [file Data_Sheet_3.zip › 4. Datasets for first model training (section DCNN model training)/training dataset/1058.jpg]

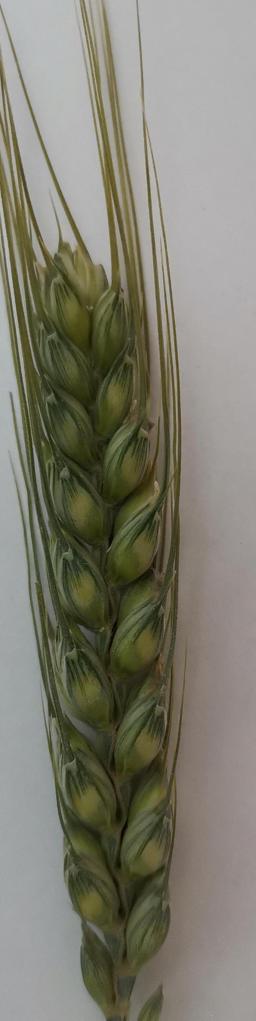

Supplement: Supplementary file 3 [file Data_Sheet_3.zip › 4. Datasets for first model training (section DCNN model training)/training dataset/1062.jpg]

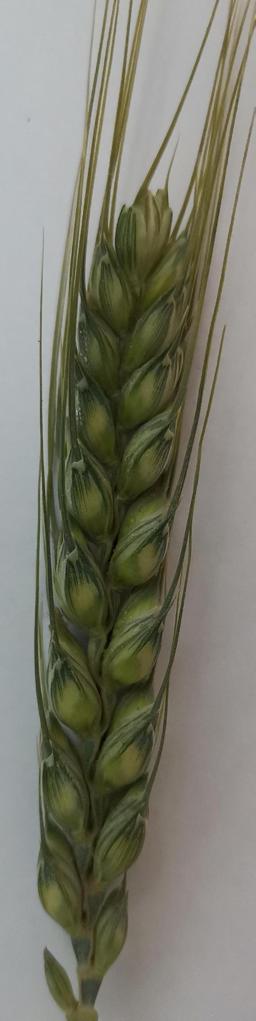

Supplement: Supplementary file 3 [file Data_Sheet_3.zip › 4. Datasets for first model training (section DCNN model training)/training dataset/1063.jpg]

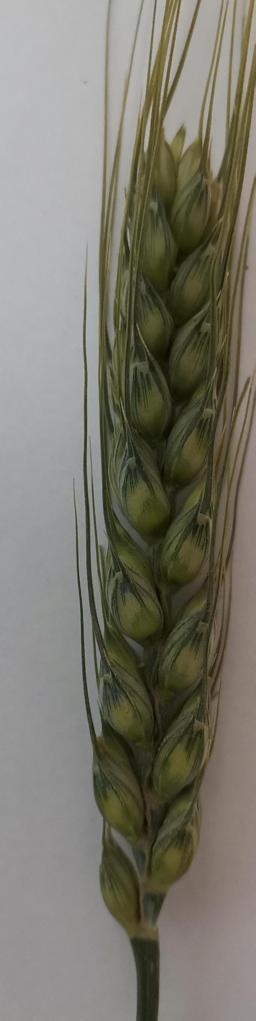

Supplement: Supplementary file 3 [file Data_Sheet_3.zip › 4. Datasets for first model training (section DCNN model training)/training dataset/1064.jpg]

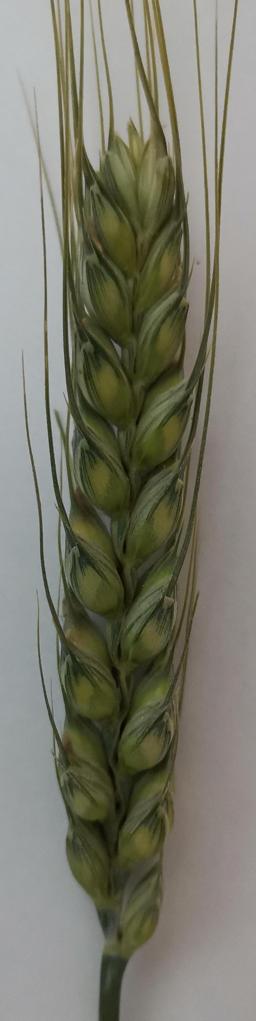

Supplement: Supplementary file 3 [file Data_Sheet_3.zip › 4. Datasets for first model training (section DCNN model training)/training dataset/1065.jpg]

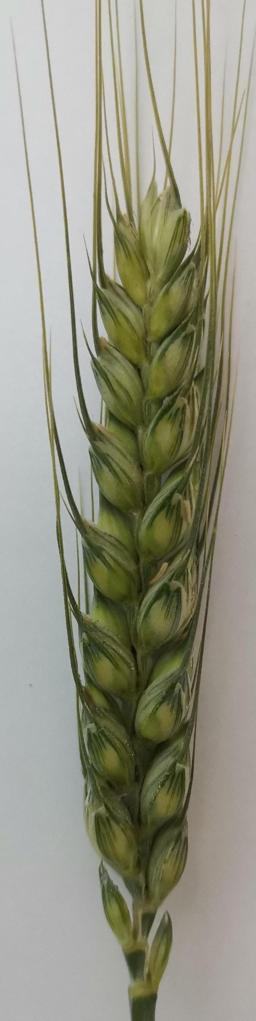

Supplement: Supplementary file 3 [file Data_Sheet_3.zip › 4. Datasets for first model training (section DCNN model training)/training dataset/1066.jpg]

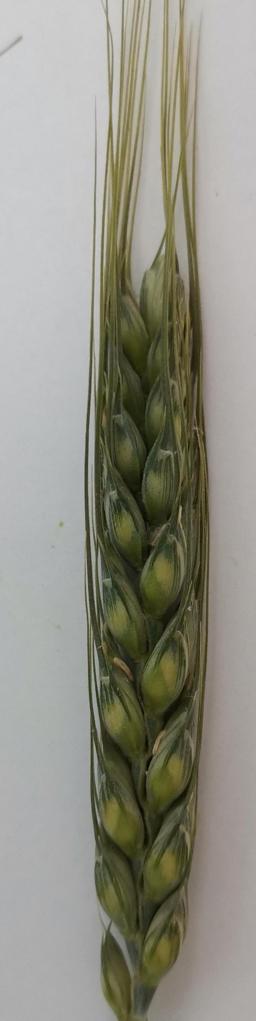

Supplement: Supplementary file 3 [file Data_Sheet_3.zip › 4. Datasets for first model training (section DCNN model training)/training dataset/1068.jpg]

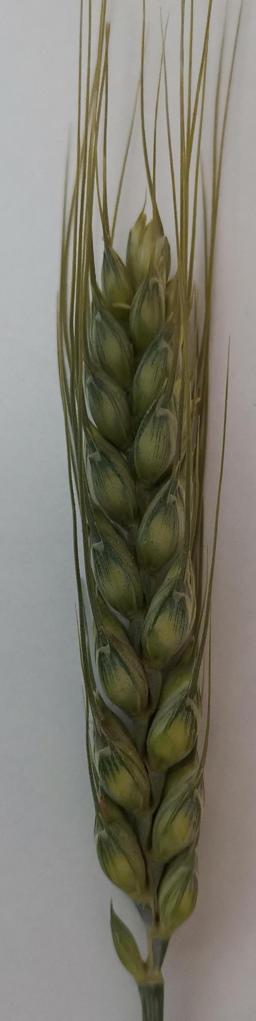

Supplement: Supplementary file 3 [file Data_Sheet_3.zip › 4. Datasets for first model training (section DCNN model training)/training dataset/1071.jpg]

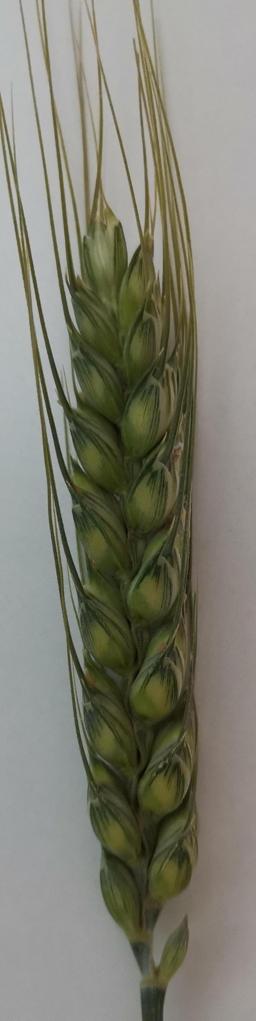

Supplement: Supplementary file 3 [file Data_Sheet_3.zip › 4. Datasets for first model training (section DCNN model training)/training dataset/1072.jpg]

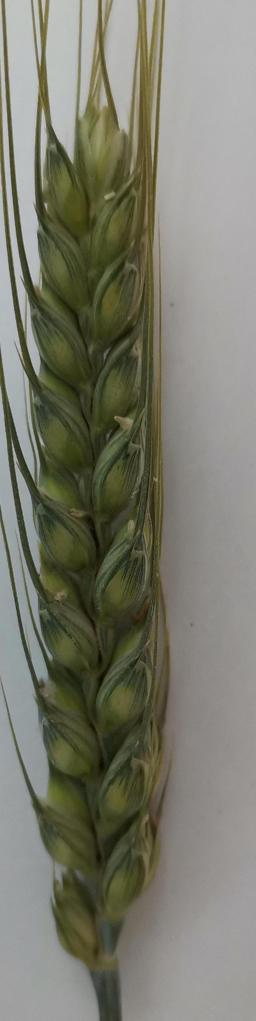

Supplement: Supplementary file 3 [file Data_Sheet_3.zip › 4. Datasets for first model training (section DCNN model training)/training dataset/1137.jpg]

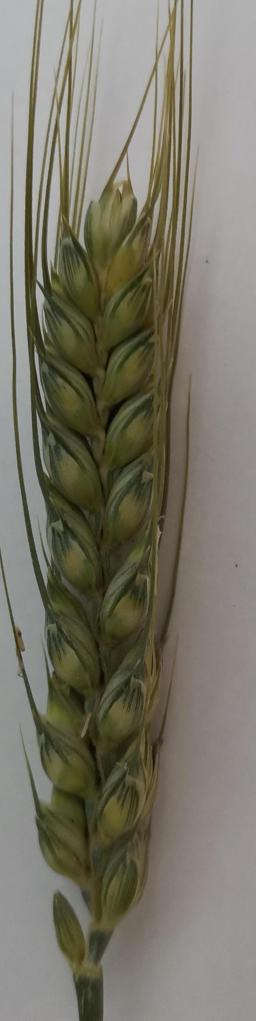

Supplement: Supplementary file 3 [file Data_Sheet_3.zip › 4. Datasets for first model training (section DCNN model training)/training dataset/1139.jpg]

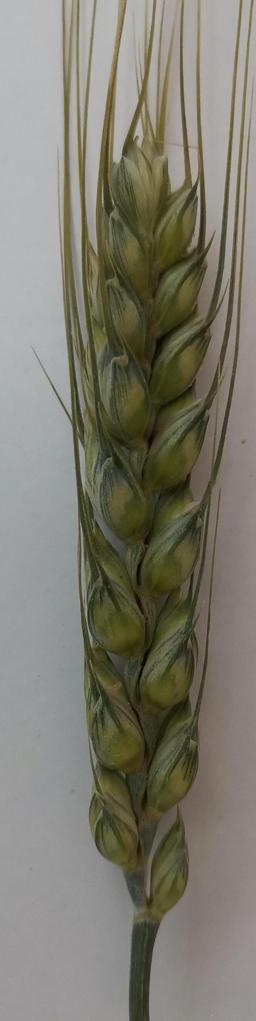

Supplement: Supplementary file 3 [file Data_Sheet_3.zip › 4. Datasets for first model training (section DCNN model training)/training dataset/1141.jpg]

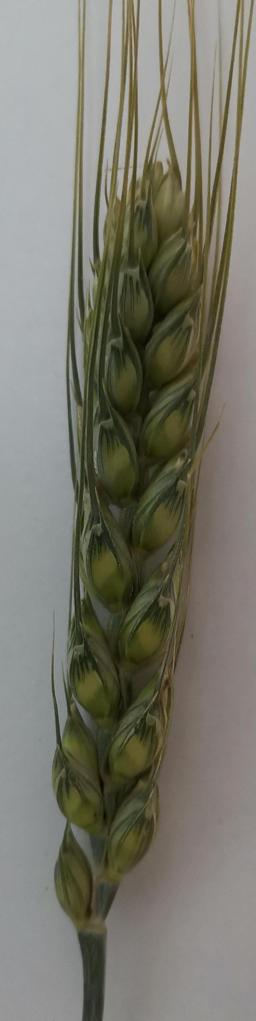

Supplement: Supplementary file 3 [file Data_Sheet_3.zip › 4. Datasets for first model training (section DCNN model training)/training dataset/1142.jpg]

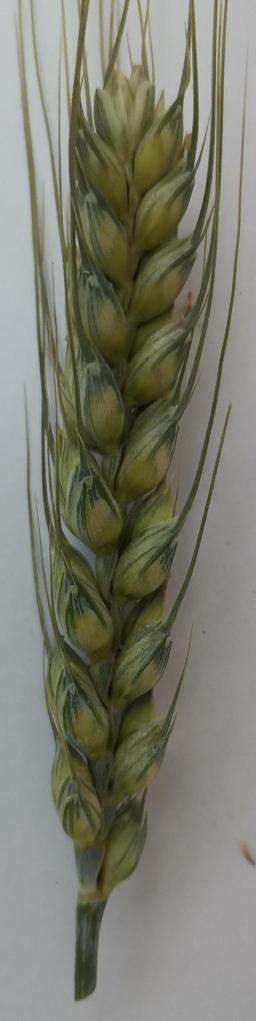

Supplement: Supplementary file 3 [file Data_Sheet_3.zip › 4. Datasets for first model training (section DCNN model training)/training dataset/1147.jpg]

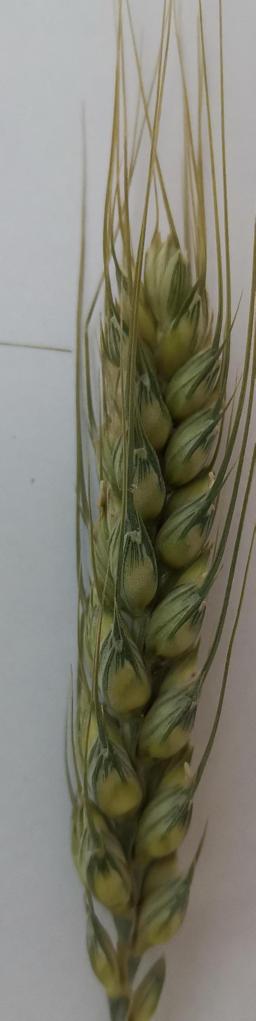

Supplement: Supplementary file 3 [file Data_Sheet_3.zip › 4. Datasets for first model training (section DCNN model training)/training dataset/1153.jpg]

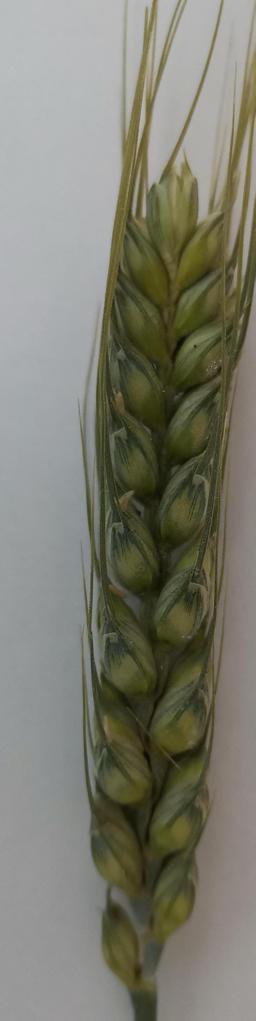

Supplement: Supplementary file 3 [file Data_Sheet_3.zip › 4. Datasets for first model training (section DCNN model training)/training dataset/1156.jpg]

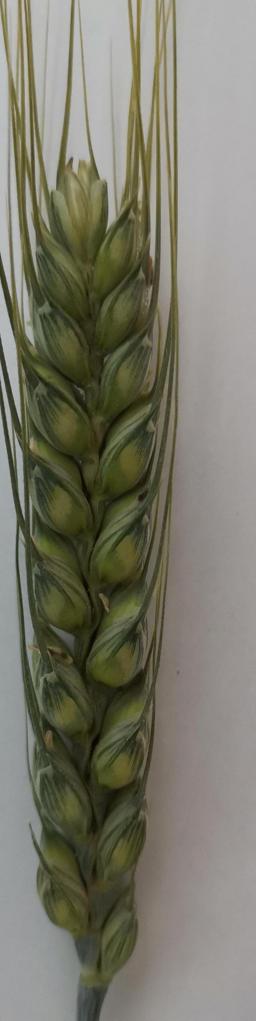

Supplement: Supplementary file 3 [file Data_Sheet_3.zip › 4. Datasets for first model training (section DCNN model training)/training dataset/1160.jpg]

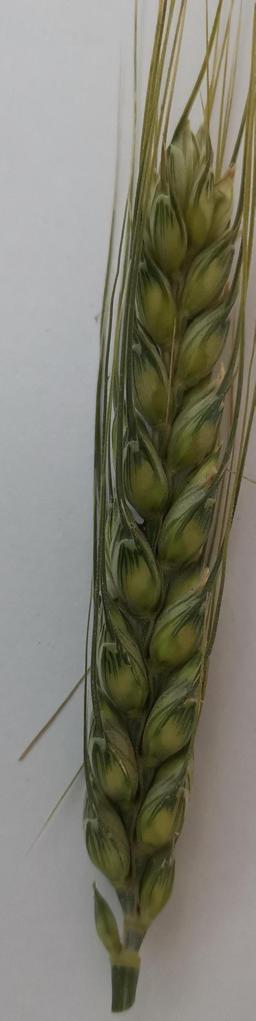

Supplement: Supplementary file 3 [file Data_Sheet_3.zip › 4. Datasets for first model training (section DCNN model training)/training dataset/1164.jpg]

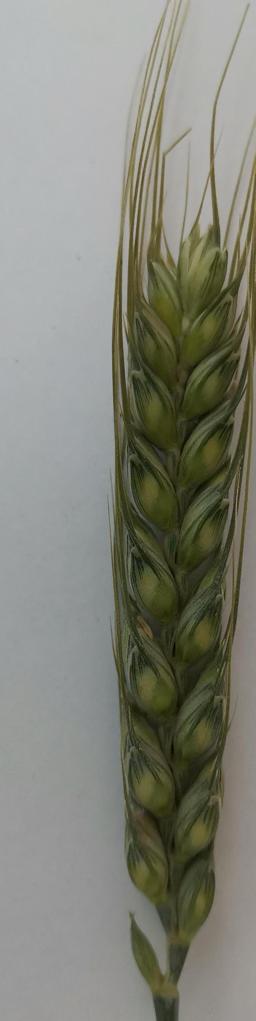

Supplement: Supplementary file 3 [file Data_Sheet_3.zip › 4. Datasets for first model training (section DCNN model training)/training dataset/1169.jpg]

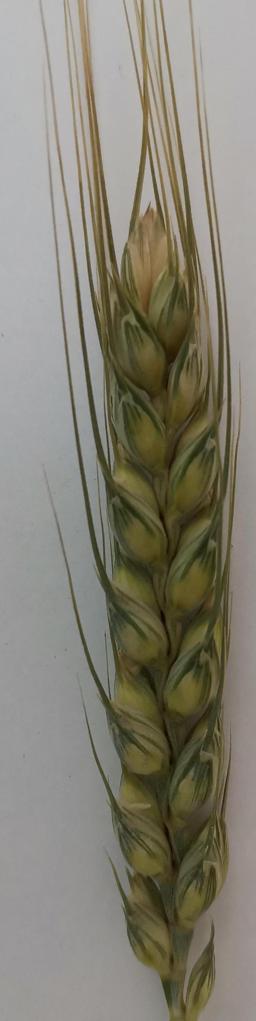

Supplement: Supplementary file 3 [file Data_Sheet_3.zip › 4. Datasets for first model training (section DCNN model training)/training dataset/1170.jpg]

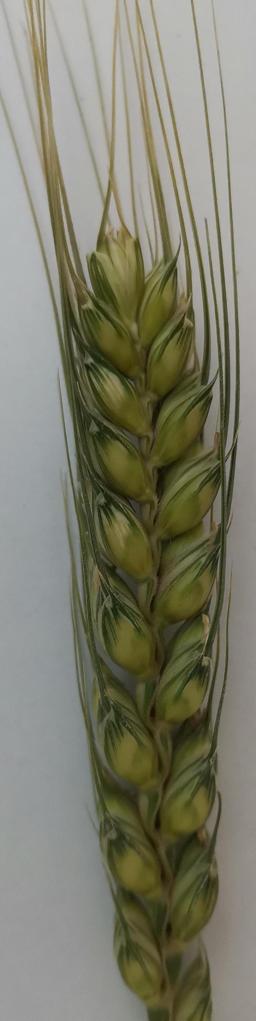

Supplement: Supplementary file 3 [file Data_Sheet_3.zip › 4. Datasets for first model training (section DCNN model training)/training dataset/1172.jpg]

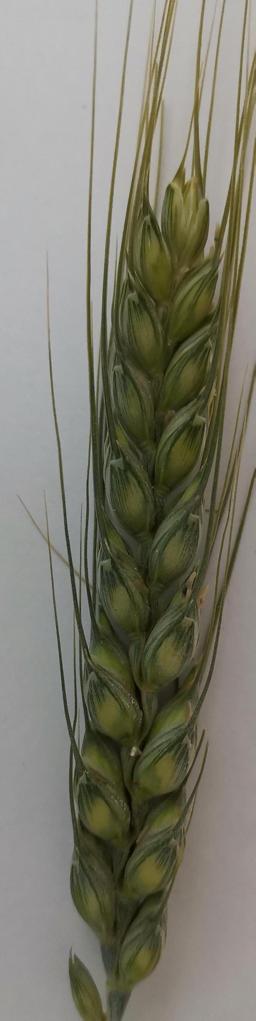

Supplement: Supplementary file 3 [file Data_Sheet_3.zip › 4. Datasets for first model training (section DCNN model training)/training dataset/1173.jpg]

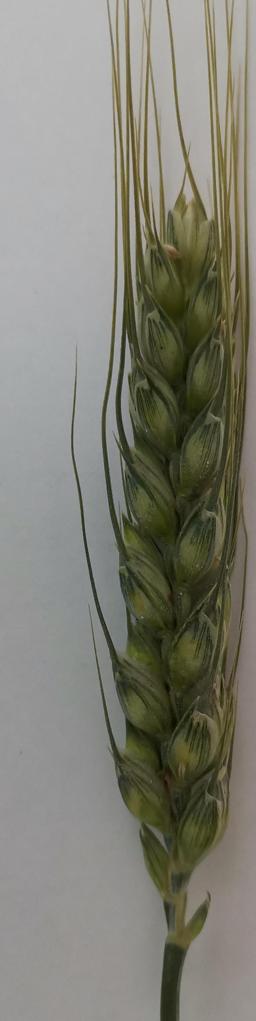

Supplement: Supplementary file 3 [file Data_Sheet_3.zip › 4. Datasets for first model training (section DCNN model training)/training dataset/1181.jpg]

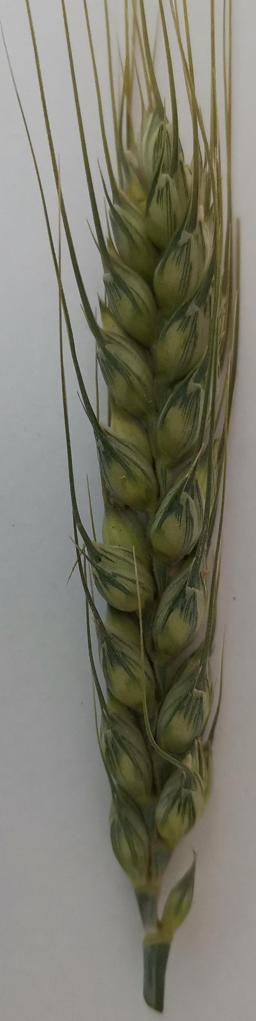

Supplement: Supplementary file 3 [file Data_Sheet_3.zip › 4. Datasets for first model training (section DCNN model training)/training dataset/1188.jpg]

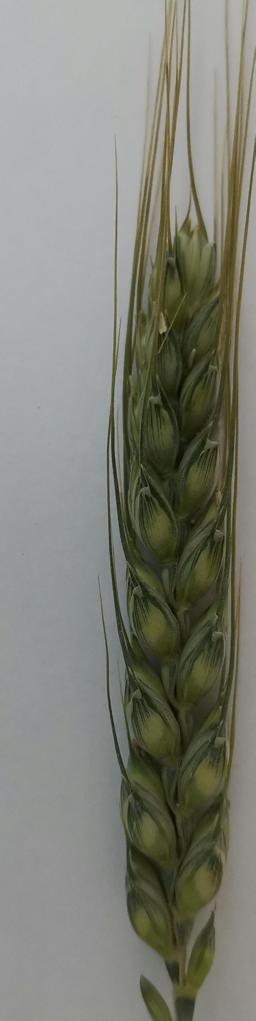

Supplement: Supplementary file 3 [file Data_Sheet_3.zip › 4. Datasets for first model training (section DCNN model training)/training dataset/1194.jpg]

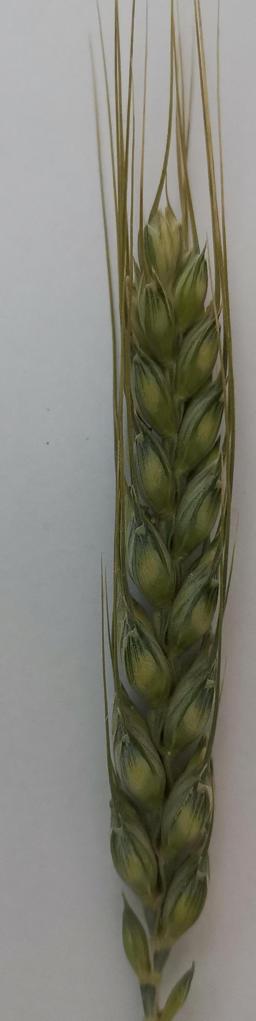

Supplement: Supplementary file 3 [file Data_Sheet_3.zip › 4. Datasets for first model training (section DCNN model training)/training dataset/1195.jpg]

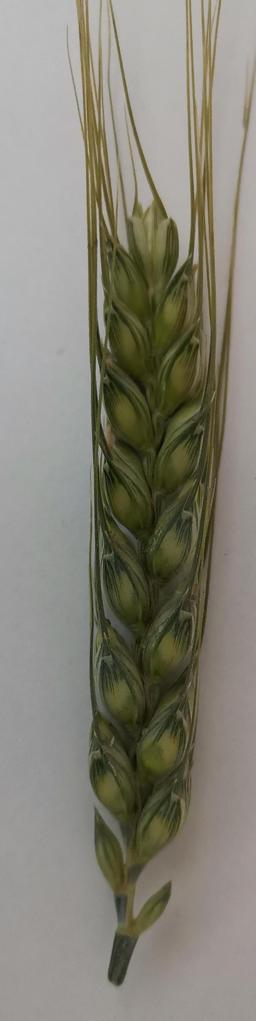

Supplement: Supplementary file 3 [file Data_Sheet_3.zip › 4. Datasets for first model training (section DCNN model training)/training dataset/1197.jpg]

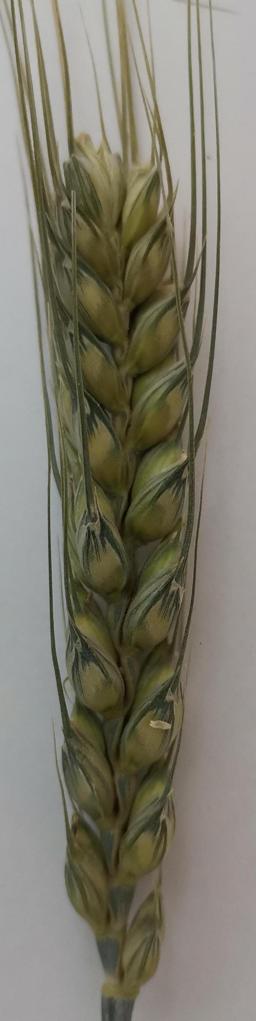

Supplement: Supplementary file 3 [file Data_Sheet_3.zip › 4. Datasets for first model training (section DCNN model training)/training dataset/1201.jpg]

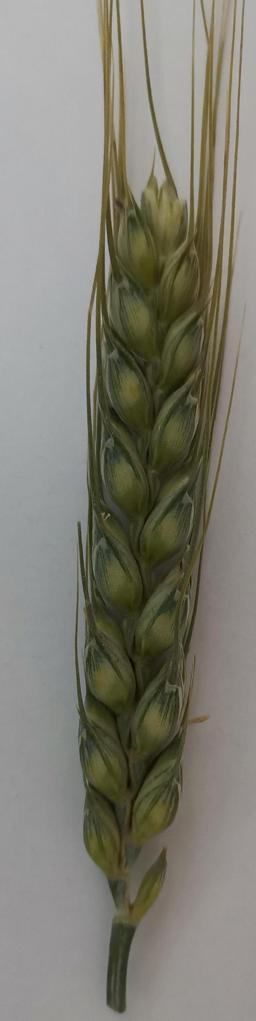

Supplement: Supplementary file 3 [file Data_Sheet_3.zip › 4. Datasets for first model training (section DCNN model training)/training dataset/1205.jpg]

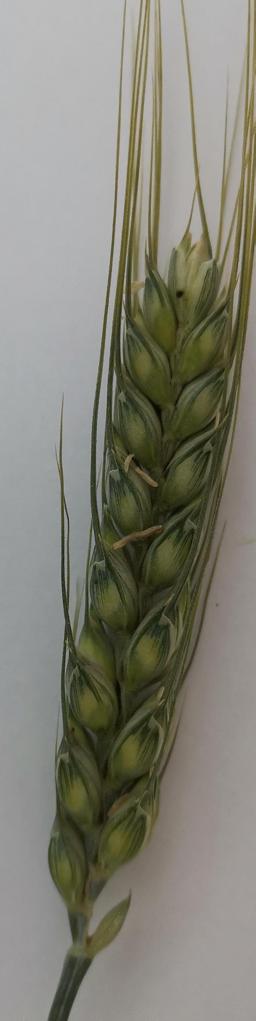

Supplement: Supplementary file 3 [file Data_Sheet_3.zip › 4. Datasets for first model training (section DCNN model training)/training dataset/1207.jpg]

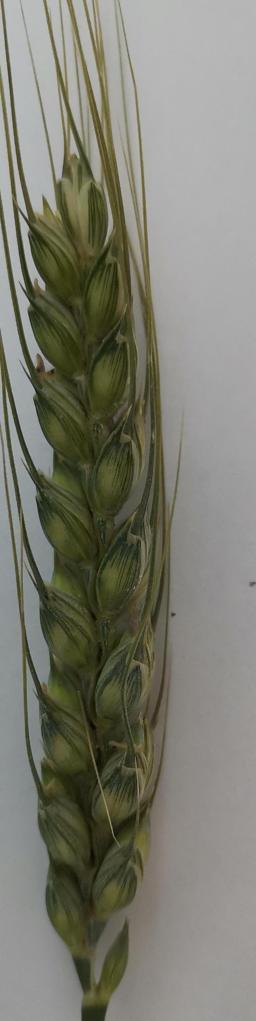

Supplement: Supplementary file 3 [file Data_Sheet_3.zip › 4. Datasets for first model training (section DCNN model training)/training dataset/1210.jpg]

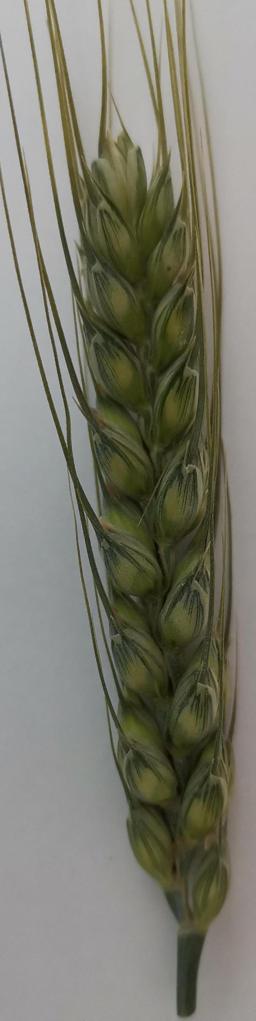

Supplement: Supplementary file 3 [file Data_Sheet_3.zip › 4. Datasets for first model training (section DCNN model training)/training dataset/1214.jpg]

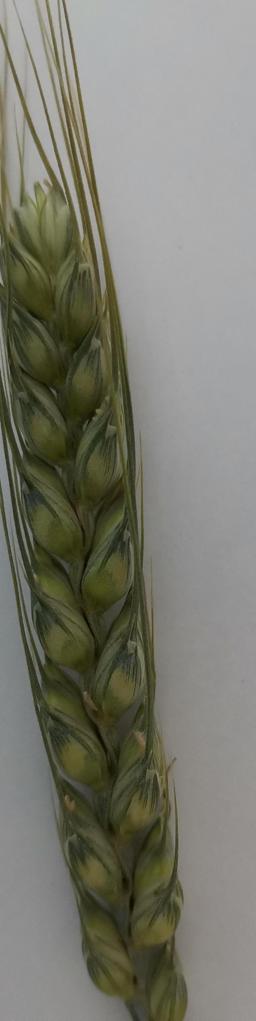

Supplement: Supplementary file 3 [file Data_Sheet_3.zip › 4. Datasets for first model training (section DCNN model training)/training dataset/1287.jpg]

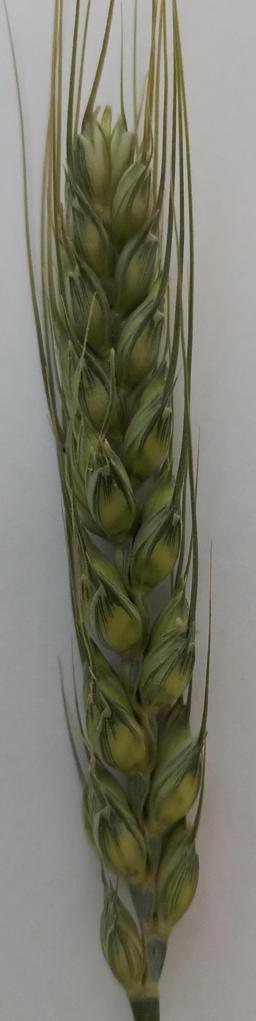

Supplement: Supplementary file 3 [file Data_Sheet_3.zip › 4. Datasets for first model training (section DCNN model training)/training dataset/1288.jpg]

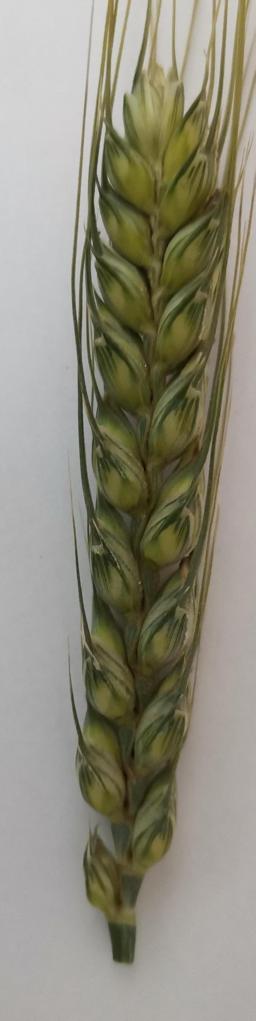

Supplement: Supplementary file 3 [file Data_Sheet_3.zip › 4. Datasets for first model training (section DCNN model training)/training dataset/1301.jpg]

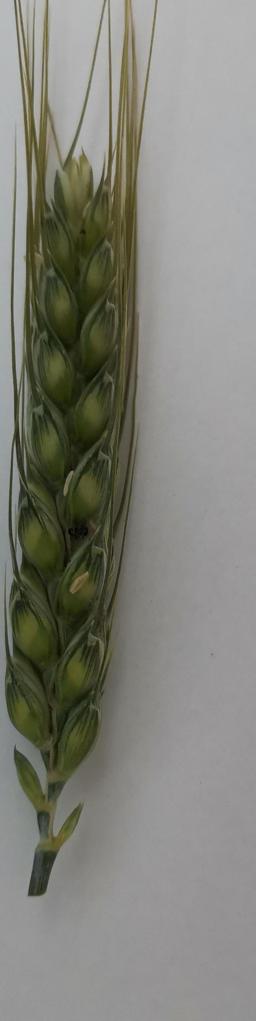

Supplement: Supplementary file 3 [file Data_Sheet_3.zip › 4. Datasets for first model training (section DCNN model training)/training dataset/1303.jpg]

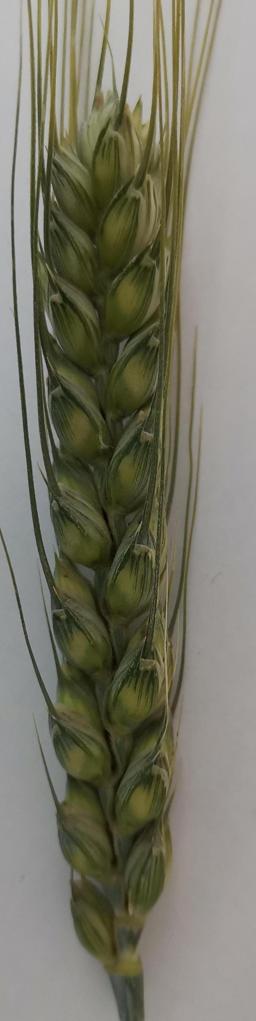

Supplement: Supplementary file 3 [file Data_Sheet_3.zip › 4. Datasets for first model training (section DCNN model training)/training dataset/1310.jpg]

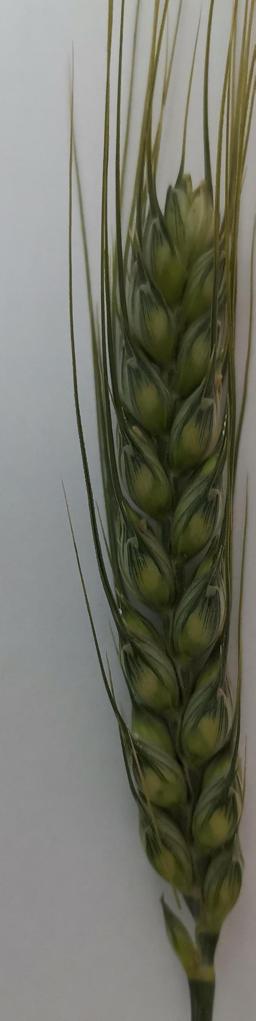

Supplement: Supplementary file 3 [file Data_Sheet_3.zip › 4. Datasets for first model training (section DCNN model training)/training dataset/1311.jpg]

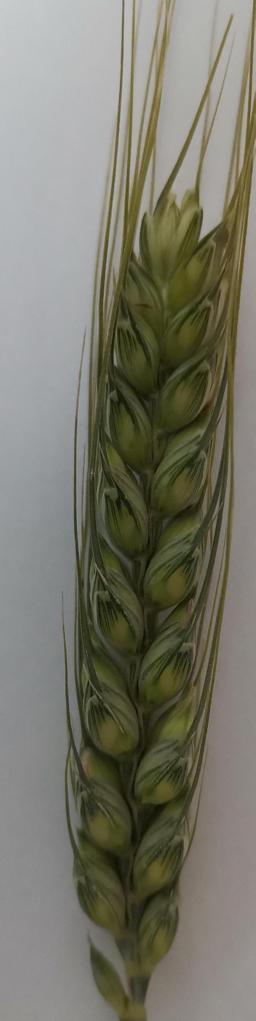

Supplement: Supplementary file 3 [file Data_Sheet_3.zip › 4. Datasets for first model training (section DCNN model training)/training dataset/1316.jpg]

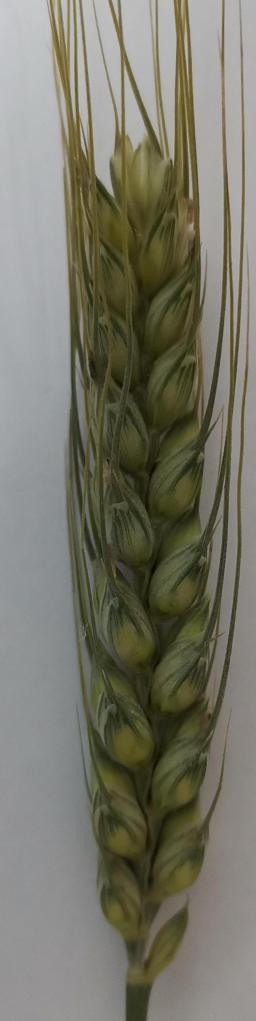

Supplement: Supplementary file 3 [file Data_Sheet_3.zip › 4. Datasets for first model training (section DCNN model training)/training dataset/1317.jpg]

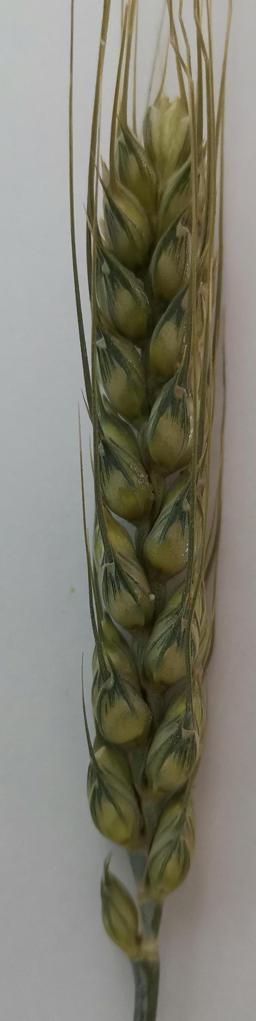

Supplement: Supplementary file 3 [file Data_Sheet_3.zip › 4. Datasets for first model training (section DCNN model training)/training dataset/1323.jpg]

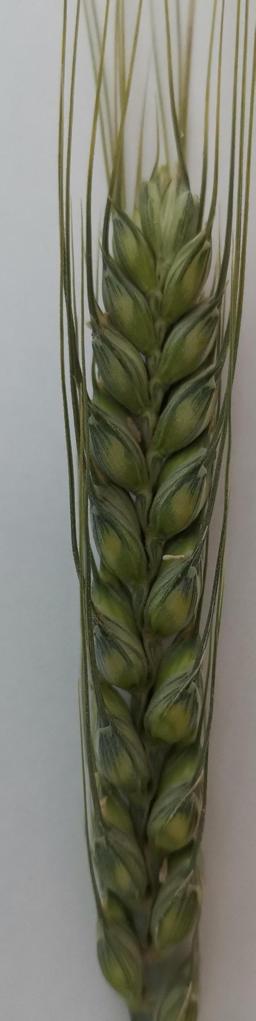

Supplement: Supplementary file 3 [file Data_Sheet_3.zip › 4. Datasets for first model training (section DCNN model training)/training dataset/1376.jpg]

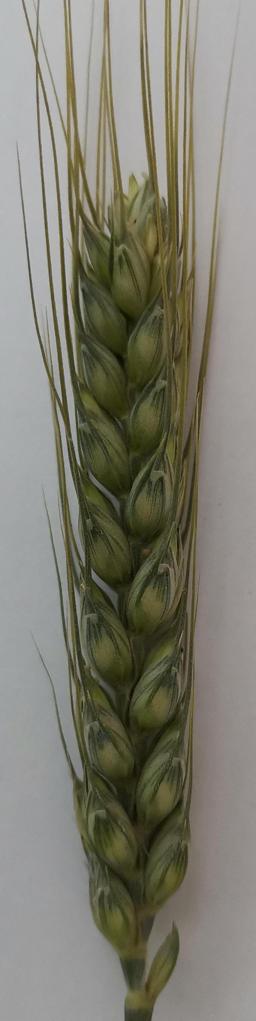

Supplement: Supplementary file 3 [file Data_Sheet_3.zip › 4. Datasets for first model training (section DCNN model training)/training dataset/1384.jpg]

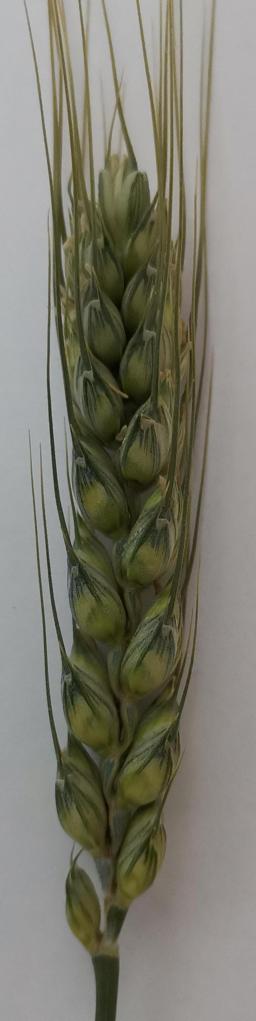

Supplement: Supplementary file 3 [file Data_Sheet_3.zip › 4. Datasets for first model training (section DCNN model training)/training dataset/1385.jpg]

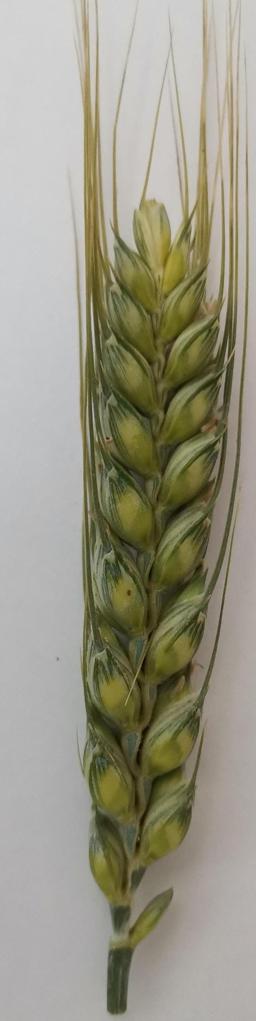

Supplement: Supplementary file 3 [file Data_Sheet_3.zip › 4. Datasets for first model training (section DCNN model training)/training dataset/1387.jpg]

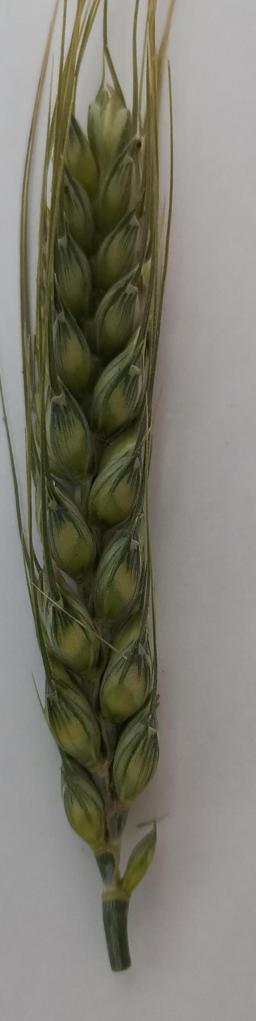

Supplement: Supplementary file 3 [file Data_Sheet_3.zip › 4. Datasets for first model training (section DCNN model training)/training dataset/1391.jpg]

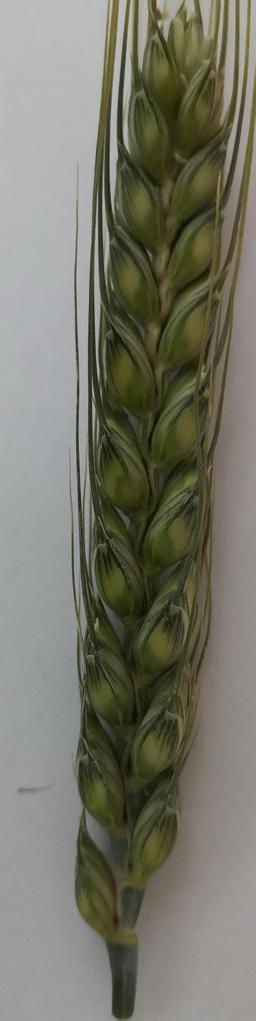

Supplement: Supplementary file 3 [file Data_Sheet_3.zip › 4. Datasets for first model training (section DCNN model training)/training dataset/1393.jpg]

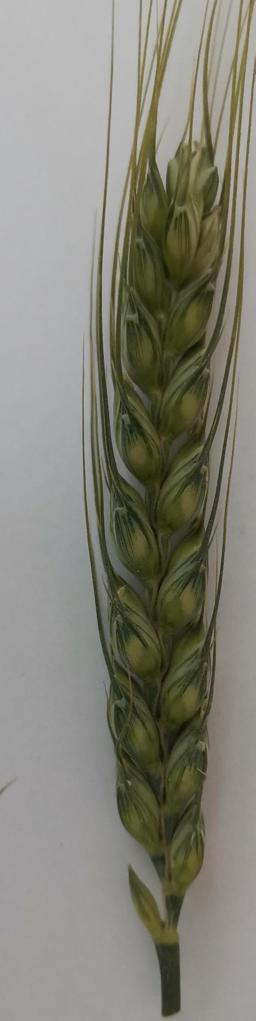

Supplement: Supplementary file 3 [file Data_Sheet_3.zip › 4. Datasets for first model training (section DCNN model training)/training dataset/1395.jpg]

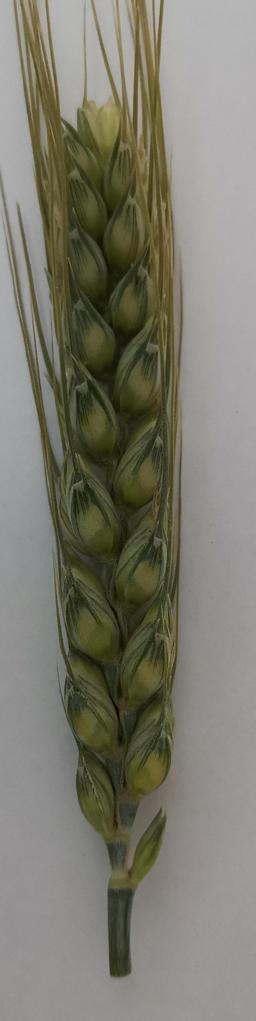

Supplement: Supplementary file 3 [file Data_Sheet_3.zip › 4. Datasets for first model training (section DCNN model training)/training dataset/1397.jpg]

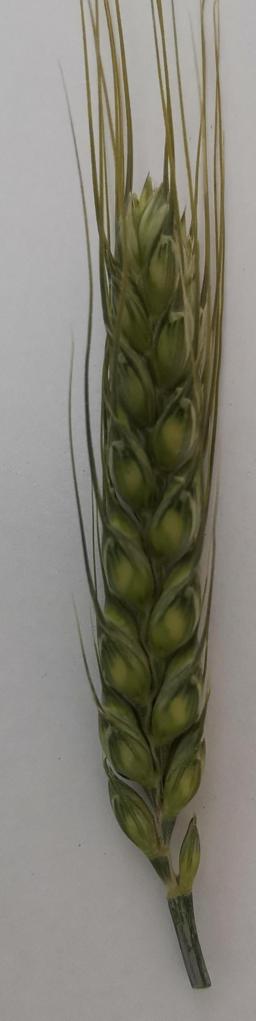

Supplement: Supplementary file 3 [file Data_Sheet_3.zip › 4. Datasets for first model training (section DCNN model training)/training dataset/1398.jpg]

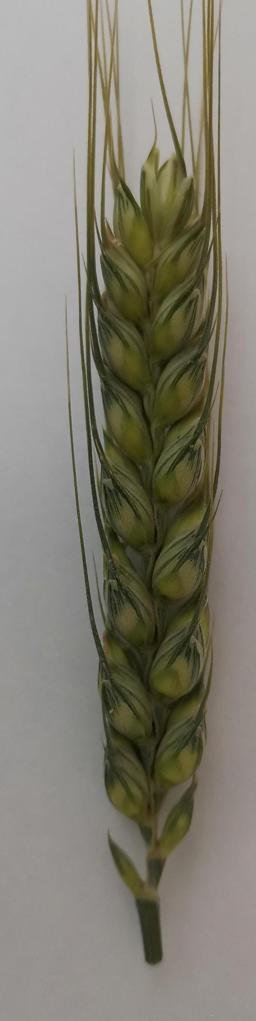

Supplement: Supplementary file 3 [file Data_Sheet_3.zip › 4. Datasets for first model training (section DCNN model training)/training dataset/1400.jpg]

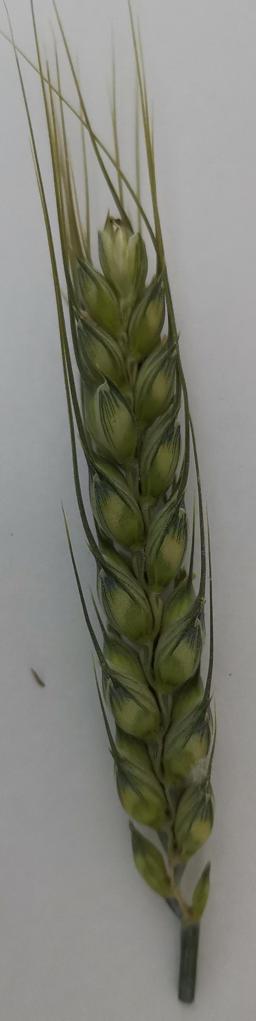

Supplement: Supplementary file 3 [file Data_Sheet_3.zip › 4. Datasets for first model training (section DCNN model training)/training dataset/1409.jpg]

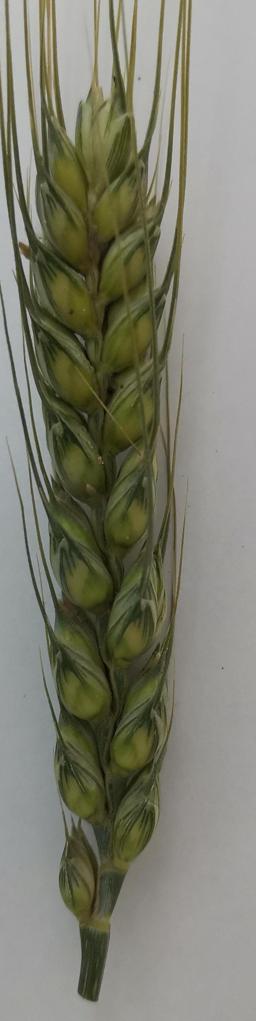

Supplement: Supplementary file 3 [file Data_Sheet_3.zip › 4. Datasets for first model training (section DCNN model training)/training dataset/1411.jpg]

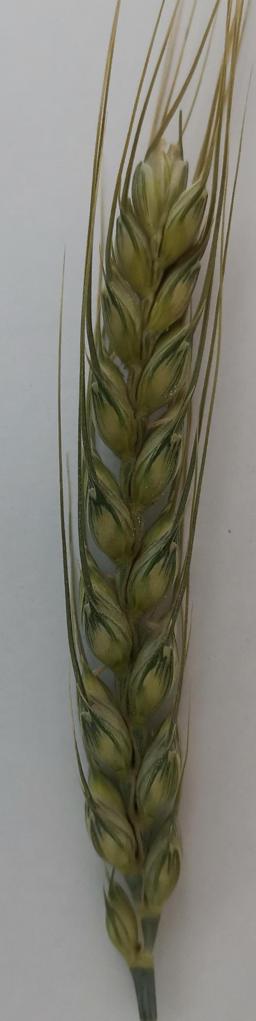

Supplement: Supplementary file 3 [file Data_Sheet_3.zip › 4. Datasets for first model training (section DCNN model training)/training dataset/1418.jpg]

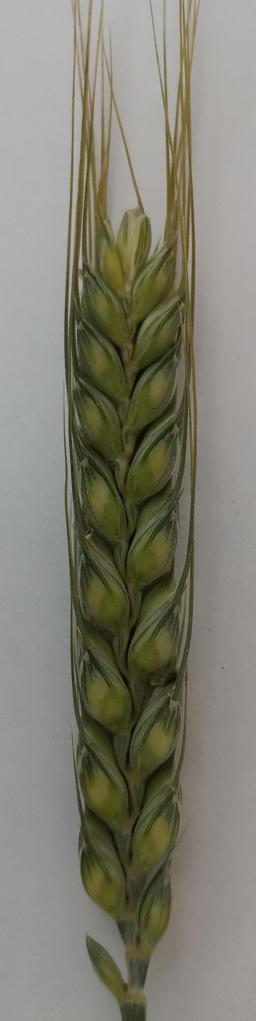

Supplement: Supplementary file 3 [file Data_Sheet_3.zip › 4. Datasets for first model training (section DCNN model training)/training dataset/1420.jpg]

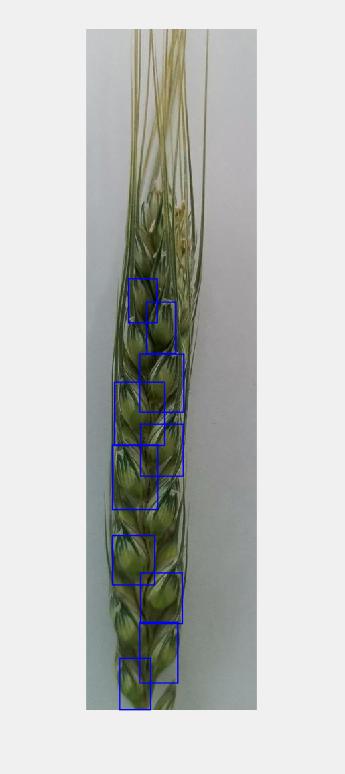

Supplement: Supplementary file 4 [file Data_Sheet_4.zip › 5. Detection results output by DCNN model (section Dataset optimization)/training dataset/Liangxing 99/3005MTL.jpg]

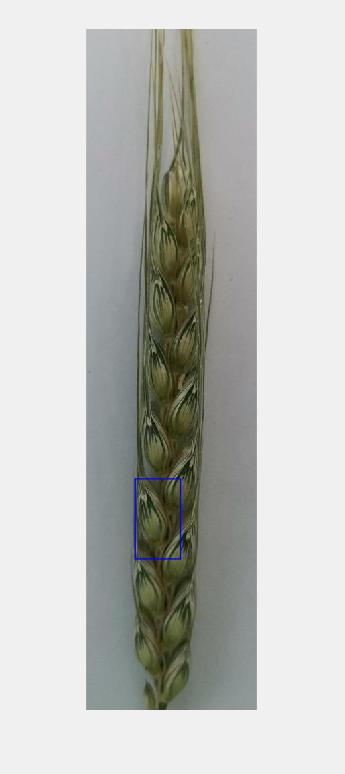

Supplement: Supplementary file 4 [file Data_Sheet_4.zip › 5. Detection results output by DCNN model (section Dataset optimization)/training dataset/Liangxing 99/3009MTL.jpg]

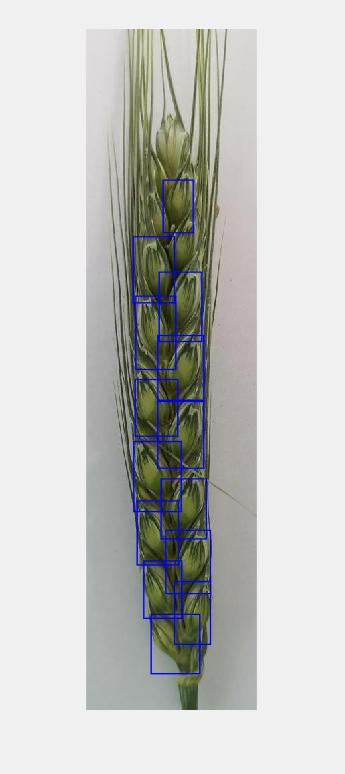

Supplement: Supplementary file 4 [file Data_Sheet_4.zip › 5. Detection results output by DCNN model (section Dataset optimization)/training dataset/Liangxing 99/3010MTL.jpg]

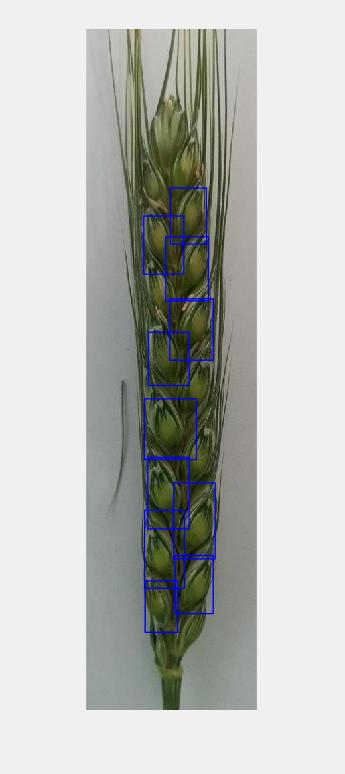

Supplement: Supplementary file 4 [file Data_Sheet_4.zip › 5. Detection results output by DCNN model (section Dataset optimization)/training dataset/Liangxing 99/3011MTL.jpg]

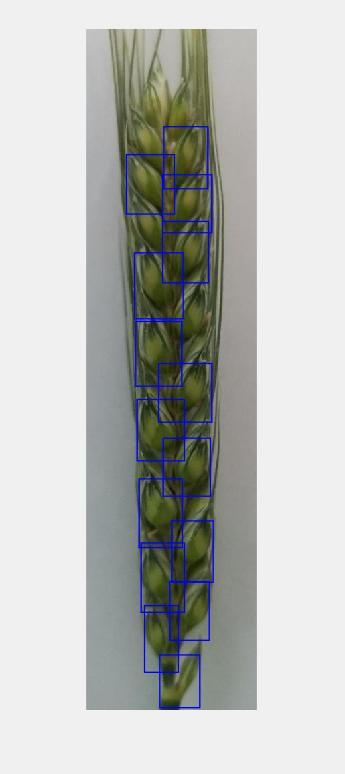

Supplement: Supplementary file 4 [file Data_Sheet_4.zip › 5. Detection results output by DCNN model (section Dataset optimization)/training dataset/Liangxing 99/3014MTL.jpg]

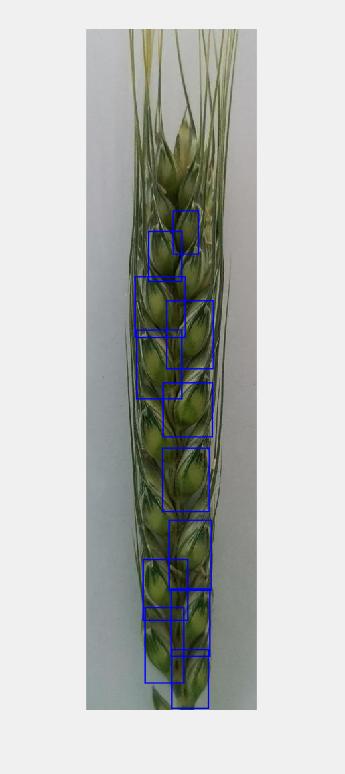

Supplement: Supplementary file 4 [file Data_Sheet_4.zip › 5. Detection results output by DCNN model (section Dataset optimization)/training dataset/Liangxing 99/3015MTL.jpg]

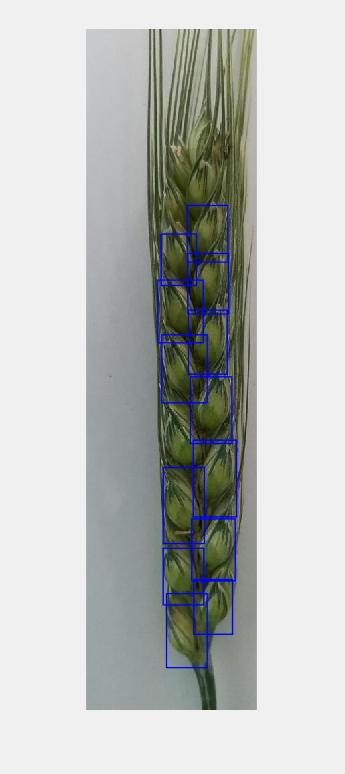

Supplement: Supplementary file 4 [file Data_Sheet_4.zip › 5. Detection results output by DCNN model (section Dataset optimization)/training dataset/Liangxing 99/3018MTL.jpg]

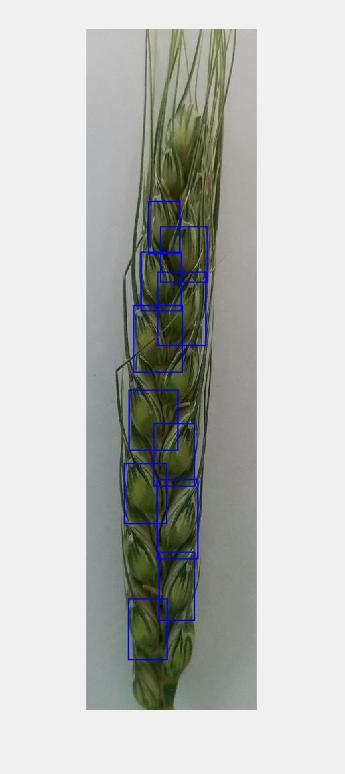

Supplement: Supplementary file 4 [file Data_Sheet_4.zip › 5. Detection results output by DCNN model (section Dataset optimization)/training dataset/Liangxing 99/3020MTL.jpg]

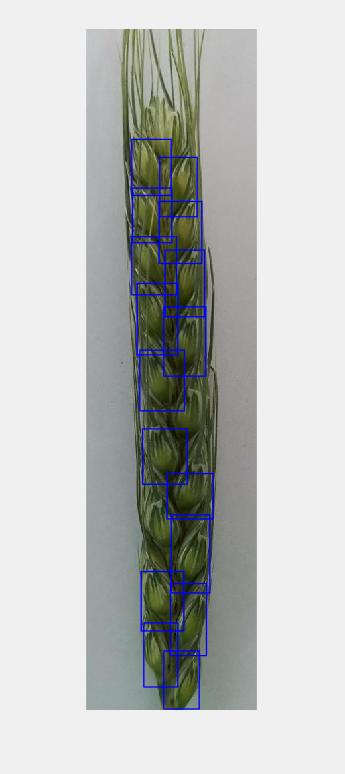

Supplement: Supplementary file 4 [file Data_Sheet_4.zip › 5. Detection results output by DCNN model (section Dataset optimization)/training dataset/Liangxing 99/3021MTL.jpg]

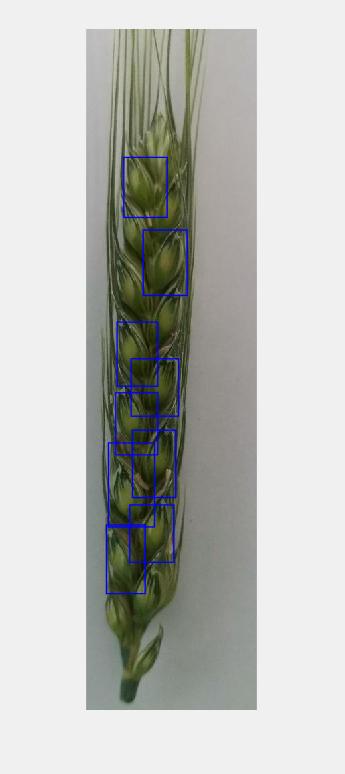

Supplement: Supplementary file 4 [file Data_Sheet_4.zip › 5. Detection results output by DCNN model (section Dataset optimization)/training dataset/Liangxing 99/3022MTL.jpg]

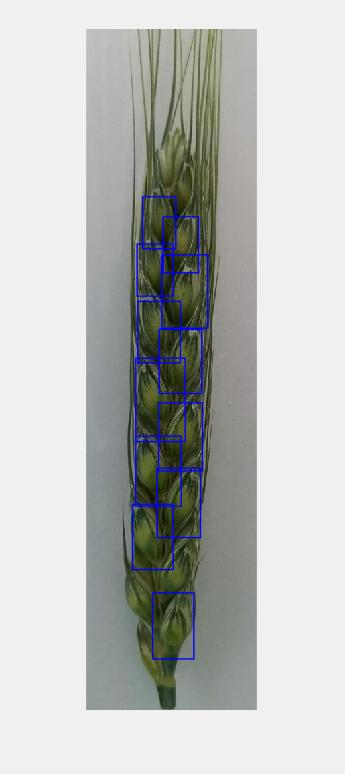

Supplement: Supplementary file 4 [file Data_Sheet_4.zip › 5. Detection results output by DCNN model (section Dataset optimization)/training dataset/Liangxing 99/3023MTL.jpg]

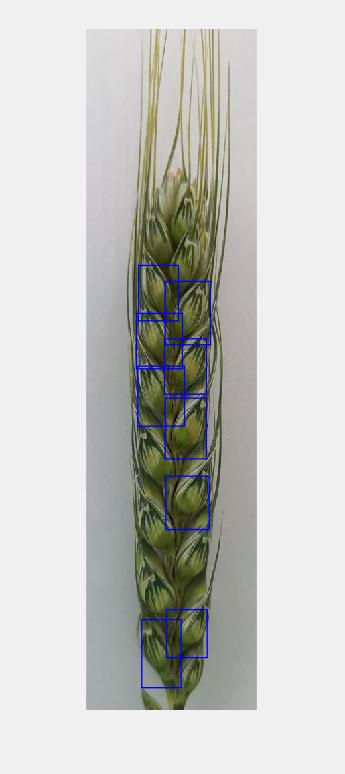

Supplement: Supplementary file 4 [file Data_Sheet_4.zip › 5. Detection results output by DCNN model (section Dataset optimization)/training dataset/Liangxing 99/3027MTL.jpg]

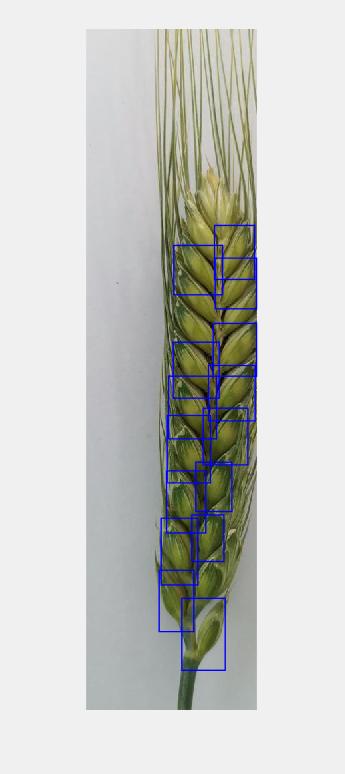

Supplement: Supplementary file 4 [file Data_Sheet_4.zip › 5. Detection results output by DCNN model (section Dataset optimization)/training dataset/Liangxing 99/3030MTL.jpg]

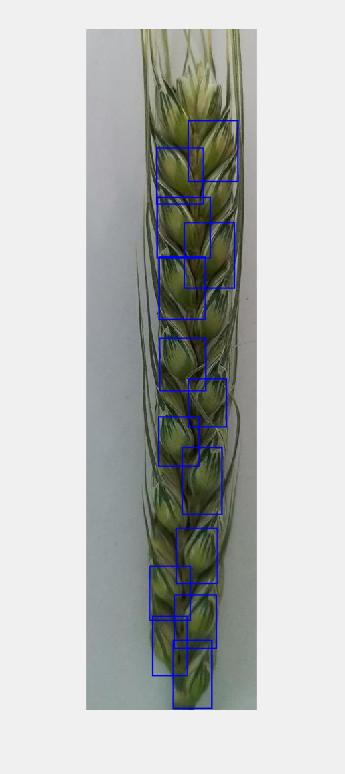

Supplement: Supplementary file 4 [file Data_Sheet_4.zip › 5. Detection results output by DCNN model (section Dataset optimization)/training dataset/Liangxing 99/3033MTL.jpg]

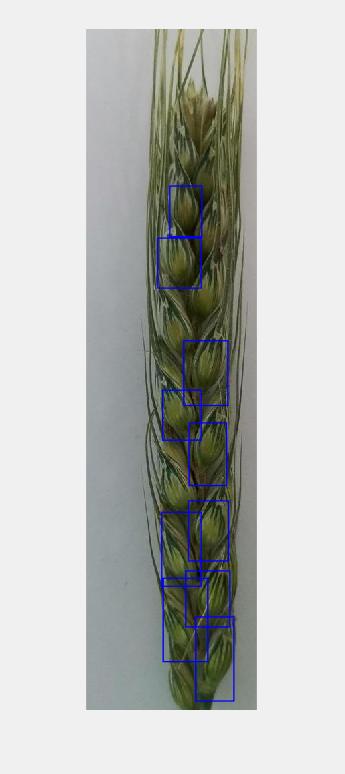

Supplement: Supplementary file 4 [file Data_Sheet_4.zip › 5. Detection results output by DCNN model (section Dataset optimization)/training dataset/Liangxing 99/3034MTL.jpg]

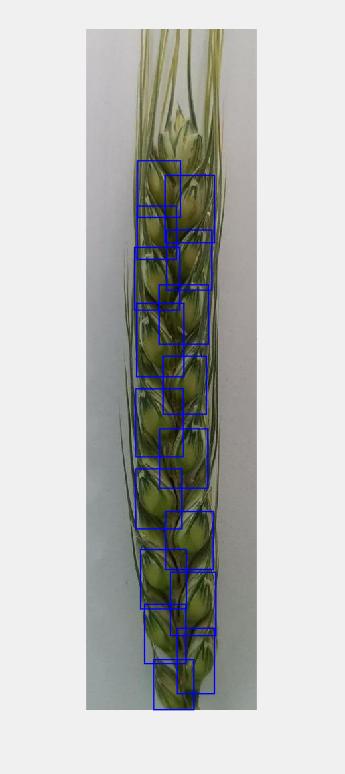

Supplement: Supplementary file 4 [file Data_Sheet_4.zip › 5. Detection results output by DCNN model (section Dataset optimization)/training dataset/Liangxing 99/3036MTL.jpg]

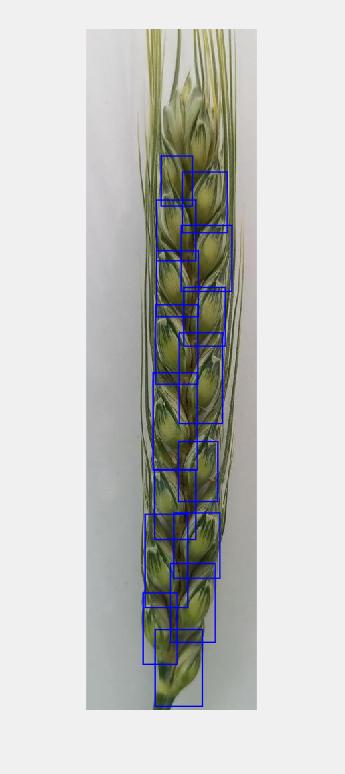

Supplement: Supplementary file 4 [file Data_Sheet_4.zip › 5. Detection results output by DCNN model (section Dataset optimization)/training dataset/Liangxing 99/3037MTL.jpg]

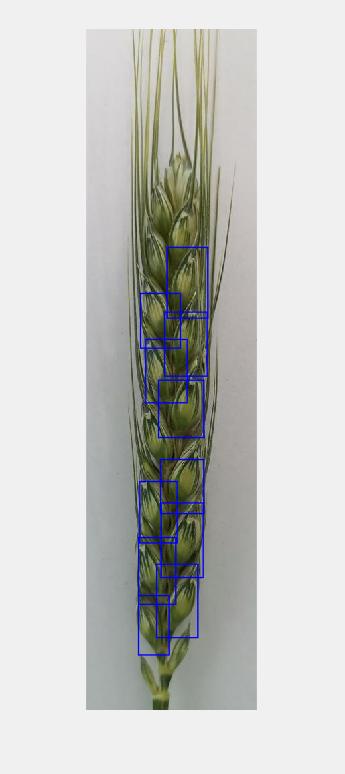

Supplement: Supplementary file 4 [file Data_Sheet_4.zip › 5. Detection results output by DCNN model (section Dataset optimization)/training dataset/Liangxing 99/3039MTL.jpg]

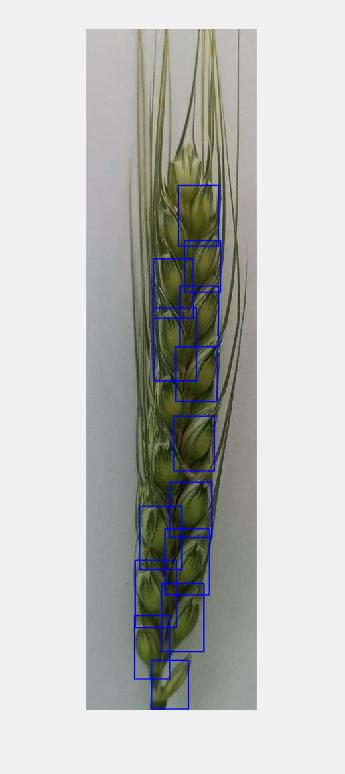

Supplement: Supplementary file 4 [file Data_Sheet_4.zip › 5. Detection results output by DCNN model (section Dataset optimization)/training dataset/Liangxing 99/3040MTL.jpg]

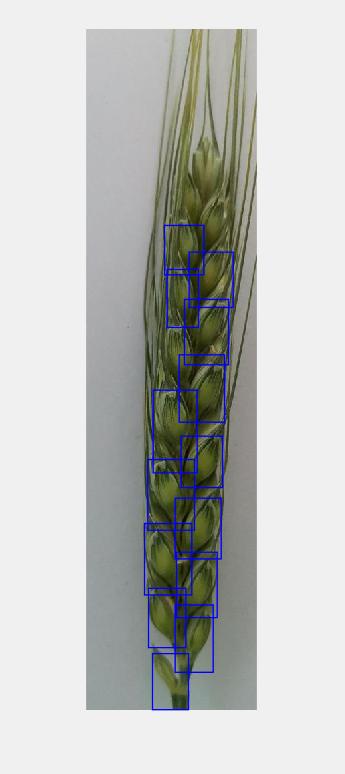

Supplement: Supplementary file 4 [file Data_Sheet_4.zip › 5. Detection results output by DCNN model (section Dataset optimization)/training dataset/Liangxing 99/3041MTL.jpg]
