# Supplementary figures and images for: Automatic Detection and Counting of Wheat Spikelet Using Semi-Automatic Labeling and Deep Learning (part 6 of 8)
Source: Front Plant Sci. 2022 May 30;13:872555. doi: 10.3389/fpls.2022.872555 (PMC9189412; doi:10.3389/fpls.2022.872555)

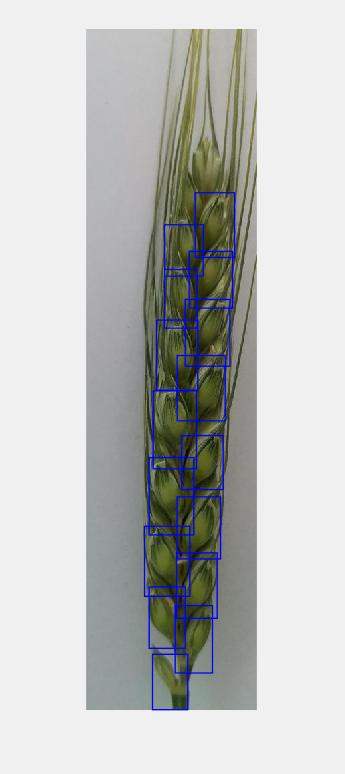

Supplement: Supplementary file 4 [file Data_Sheet_4.zip › 5. Detection results output by DCNN model (section Dataset optimization)/training dataset/Liangxing 99/3042MTL.jpg]

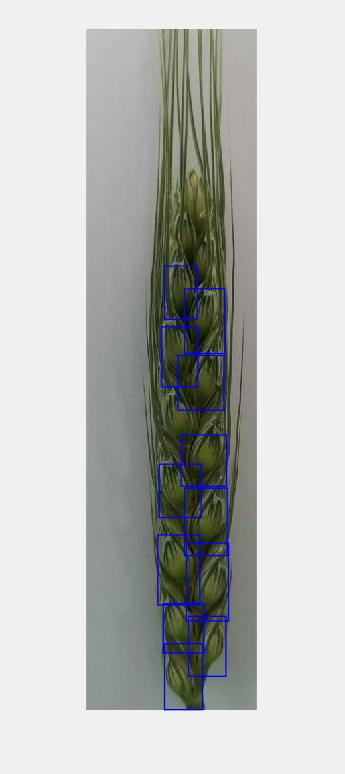

Supplement: Supplementary file 4 [file Data_Sheet_4.zip › 5. Detection results output by DCNN model (section Dataset optimization)/training dataset/Liangxing 99/3043MTL.jpg]

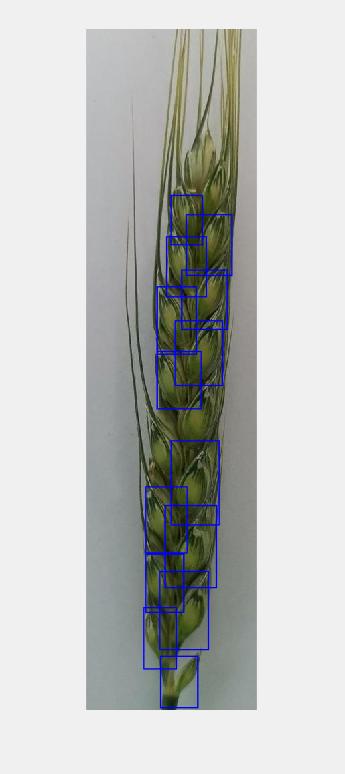

Supplement: Supplementary file 4 [file Data_Sheet_4.zip › 5. Detection results output by DCNN model (section Dataset optimization)/training dataset/Liangxing 99/3052MTL.jpg]

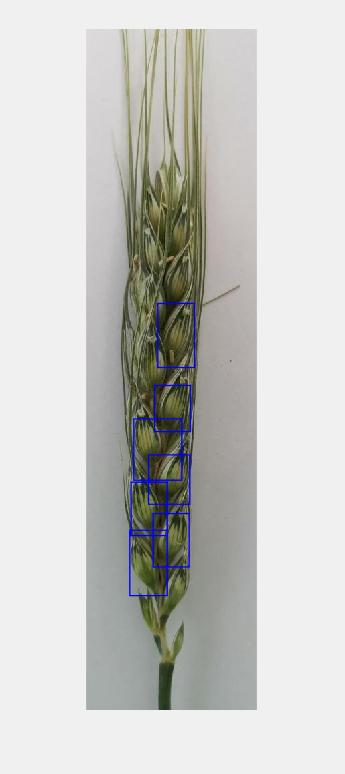

Supplement: Supplementary file 4 [file Data_Sheet_4.zip › 5. Detection results output by DCNN model (section Dataset optimization)/training dataset/Liangxing 99/3055MTL.jpg]

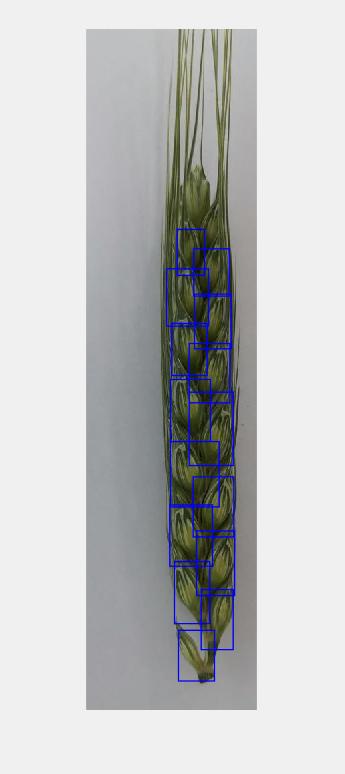

Supplement: Supplementary file 4 [file Data_Sheet_4.zip › 5. Detection results output by DCNN model (section Dataset optimization)/training dataset/Liangxing 99/3057MTL.jpg]

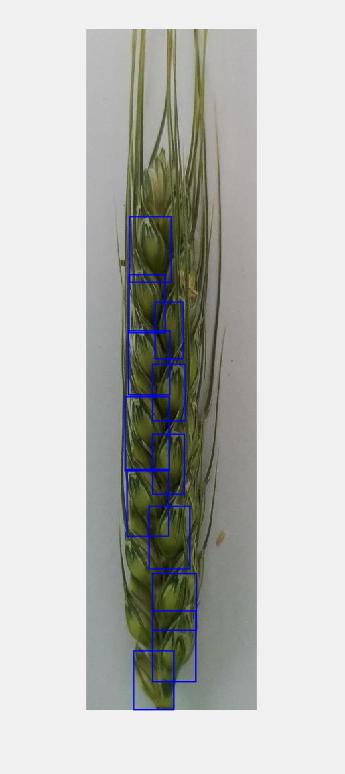

Supplement: Supplementary file 4 [file Data_Sheet_4.zip › 5. Detection results output by DCNN model (section Dataset optimization)/training dataset/Liangxing 99/3058MTL.jpg]

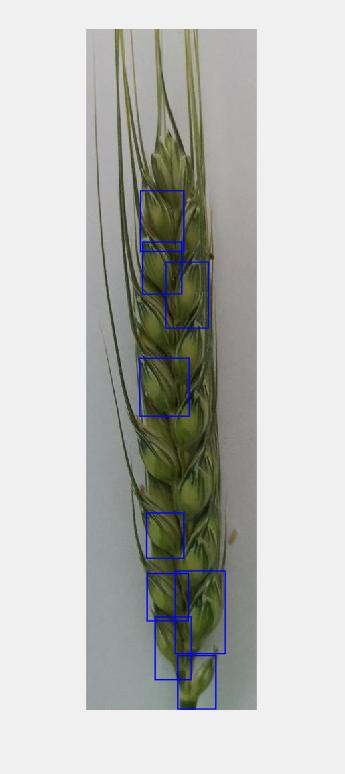

Supplement: Supplementary file 4 [file Data_Sheet_4.zip › 5. Detection results output by DCNN model (section Dataset optimization)/training dataset/Liangxing 99/3059MTL.jpg]

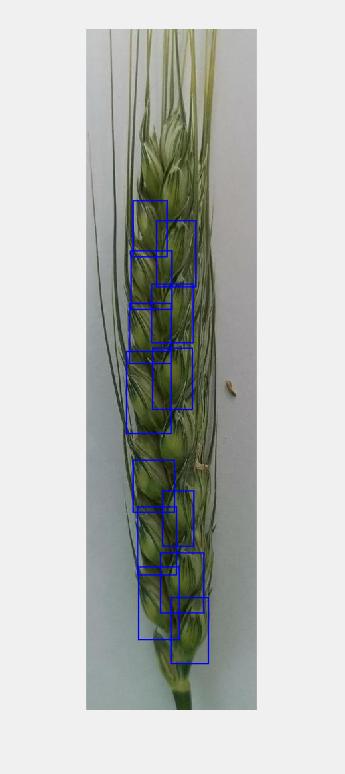

Supplement: Supplementary file 4 [file Data_Sheet_4.zip › 5. Detection results output by DCNN model (section Dataset optimization)/training dataset/Liangxing 99/3060MTL.jpg]

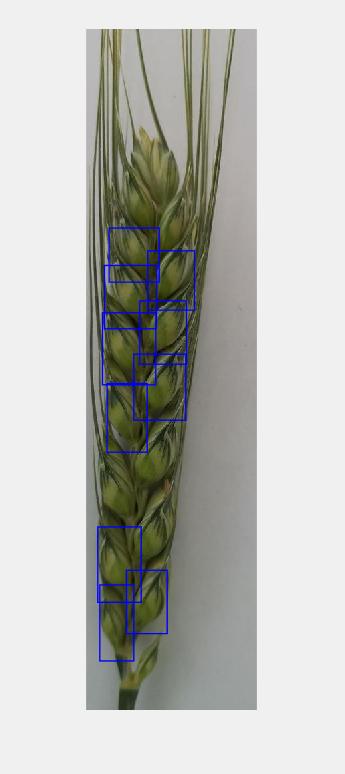

Supplement: Supplementary file 4 [file Data_Sheet_4.zip › 5. Detection results output by DCNN model (section Dataset optimization)/training dataset/Liangxing 99/3064MTL.jpg]

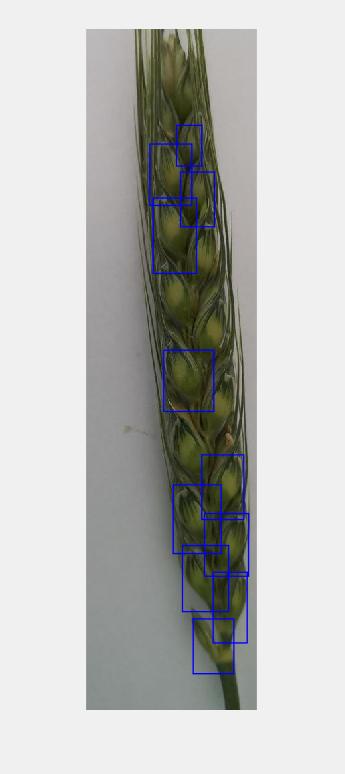

Supplement: Supplementary file 4 [file Data_Sheet_4.zip › 5. Detection results output by DCNN model (section Dataset optimization)/training dataset/Liangxing 99/3071MTL.jpg]

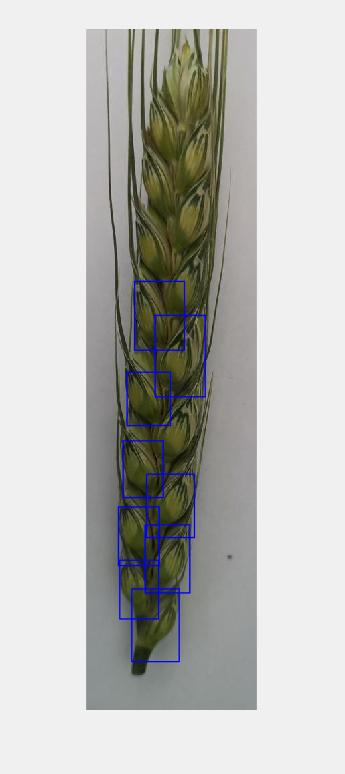

Supplement: Supplementary file 4 [file Data_Sheet_4.zip › 5. Detection results output by DCNN model (section Dataset optimization)/training dataset/Liangxing 99/3073MTL.jpg]

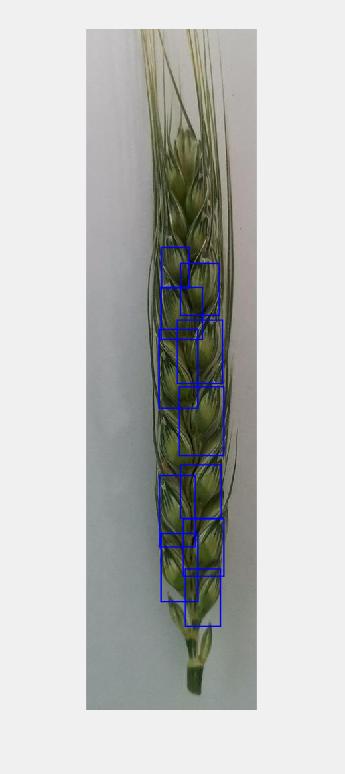

Supplement: Supplementary file 4 [file Data_Sheet_4.zip › 5. Detection results output by DCNN model (section Dataset optimization)/training dataset/Liangxing 99/3075MTL.jpg]

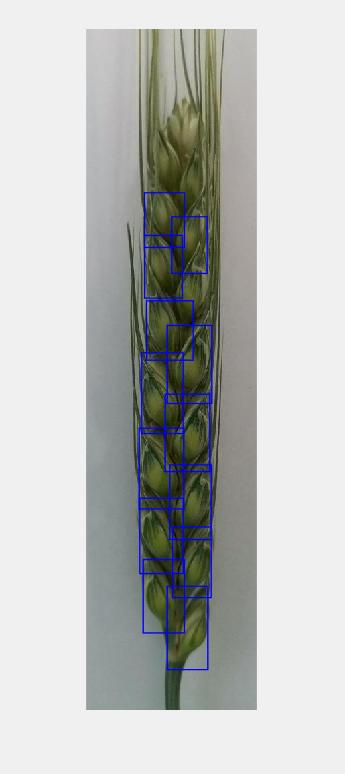

Supplement: Supplementary file 4 [file Data_Sheet_4.zip › 5. Detection results output by DCNN model (section Dataset optimization)/training dataset/Liangxing 99/3076MTL.jpg]

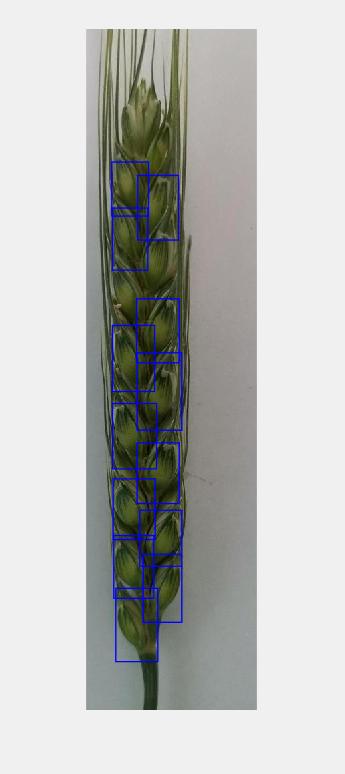

Supplement: Supplementary file 4 [file Data_Sheet_4.zip › 5. Detection results output by DCNN model (section Dataset optimization)/training dataset/Liangxing 99/3077MTL.jpg]

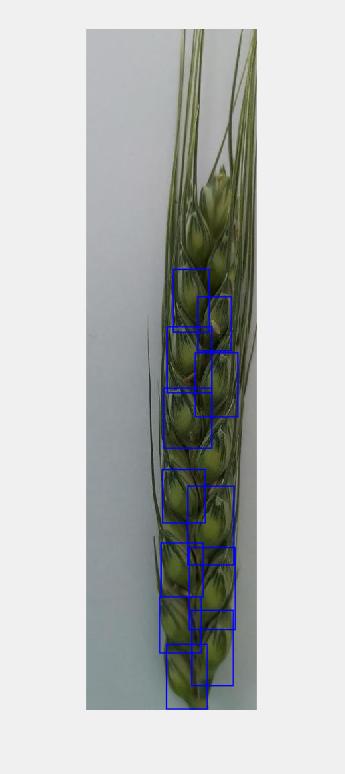

Supplement: Supplementary file 4 [file Data_Sheet_4.zip › 5. Detection results output by DCNN model (section Dataset optimization)/training dataset/Liangxing 99/3110MTL.jpg]

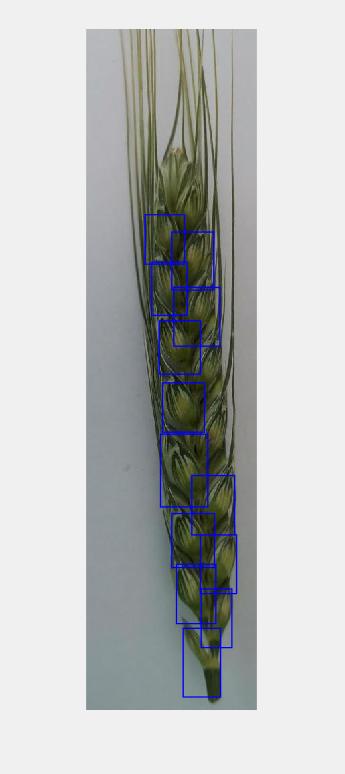

Supplement: Supplementary file 4 [file Data_Sheet_4.zip › 5. Detection results output by DCNN model (section Dataset optimization)/training dataset/Liangxing 99/3115MTL.jpg]

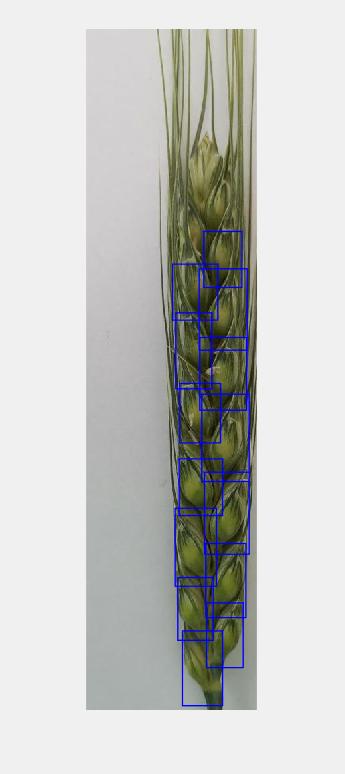

Supplement: Supplementary file 4 [file Data_Sheet_4.zip › 5. Detection results output by DCNN model (section Dataset optimization)/training dataset/Liangxing 99/3116MTL.jpg]

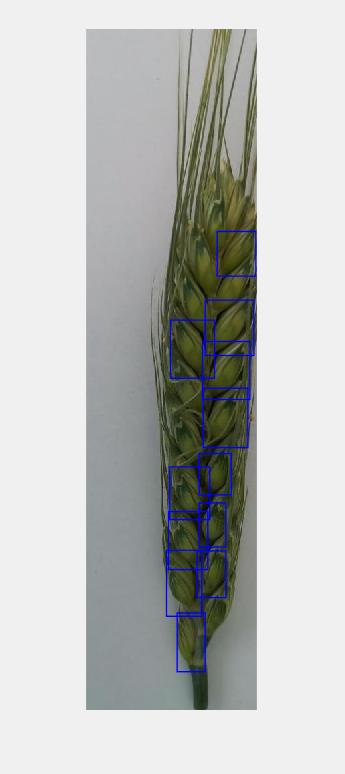

Supplement: Supplementary file 4 [file Data_Sheet_4.zip › 5. Detection results output by DCNN model (section Dataset optimization)/training dataset/Liangxing 99/3120MTL.jpg]

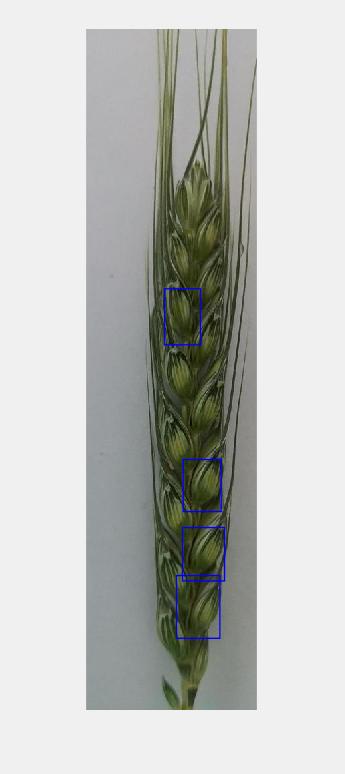

Supplement: Supplementary file 4 [file Data_Sheet_4.zip › 5. Detection results output by DCNN model (section Dataset optimization)/training dataset/Liangxing 99/3133MTL.jpg]

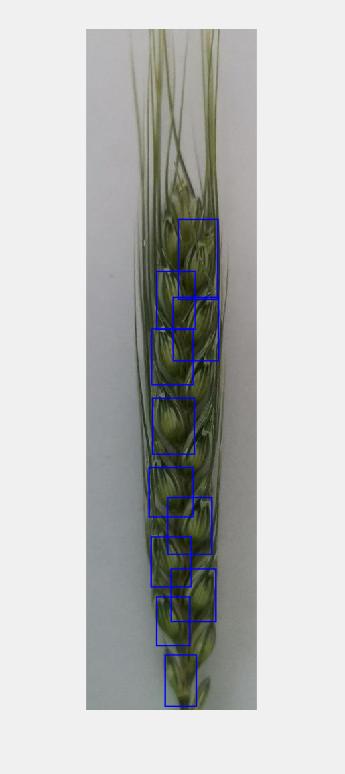

Supplement: Supplementary file 4 [file Data_Sheet_4.zip › 5. Detection results output by DCNN model (section Dataset optimization)/training dataset/Liangxing 99/3134MTL.jpg]

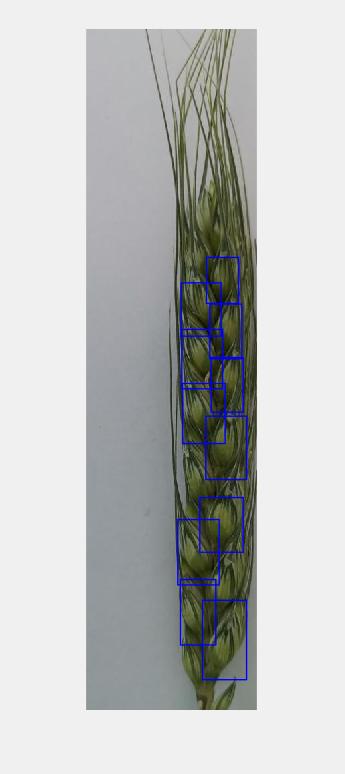

Supplement: Supplementary file 4 [file Data_Sheet_4.zip › 5. Detection results output by DCNN model (section Dataset optimization)/training dataset/Liangxing 99/3140MTL.jpg]

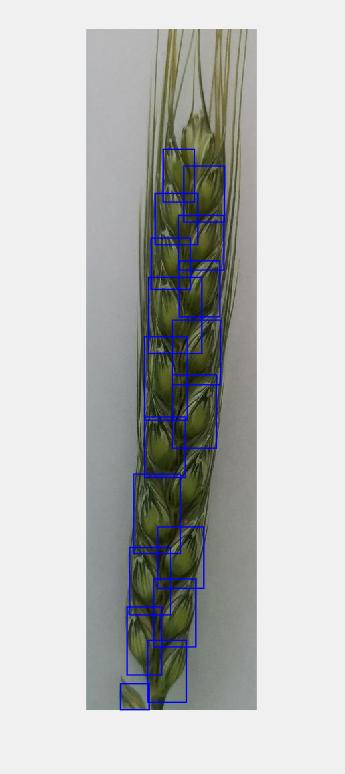

Supplement: Supplementary file 4 [file Data_Sheet_4.zip › 5. Detection results output by DCNN model (section Dataset optimization)/training dataset/Liangxing 99/3143MTL.jpg]

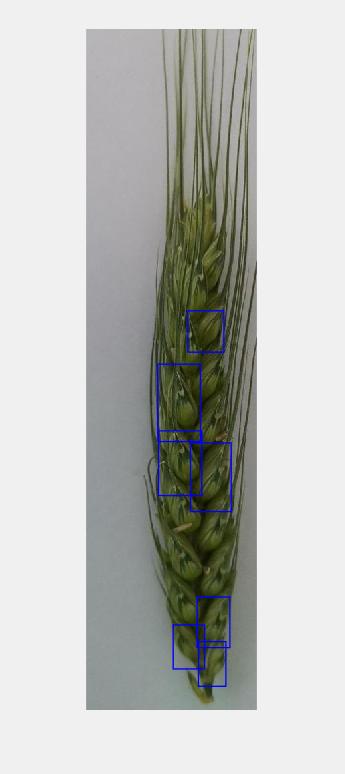

Supplement: Supplementary file 4 [file Data_Sheet_4.zip › 5. Detection results output by DCNN model (section Dataset optimization)/training dataset/Liangxing 99/3149MTL.jpg]

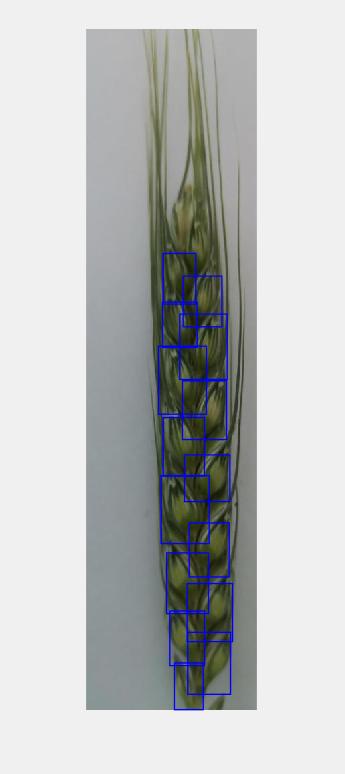

Supplement: Supplementary file 4 [file Data_Sheet_4.zip › 5. Detection results output by DCNN model (section Dataset optimization)/training dataset/Liangxing 99/3151MTL.jpg]

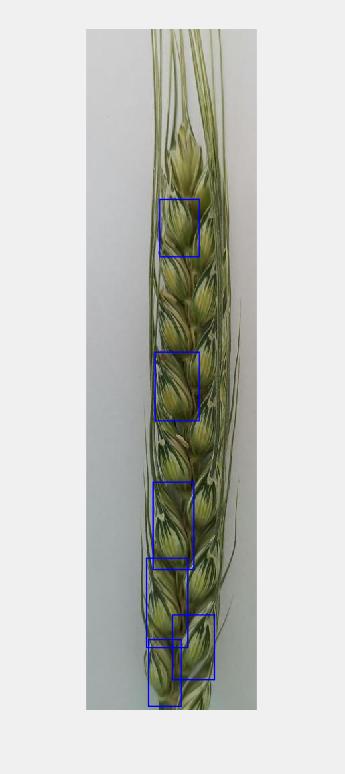

Supplement: Supplementary file 4 [file Data_Sheet_4.zip › 5. Detection results output by DCNN model (section Dataset optimization)/training dataset/Liangxing 99/3153MTL.jpg]

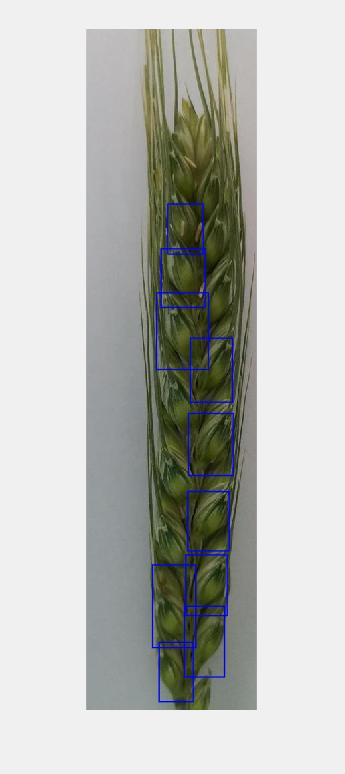

Supplement: Supplementary file 4 [file Data_Sheet_4.zip › 5. Detection results output by DCNN model (section Dataset optimization)/training dataset/Liangxing 99/3157MTL.jpg]

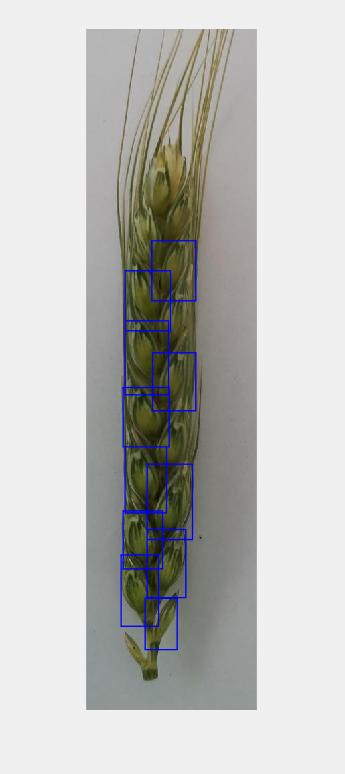

Supplement: Supplementary file 4 [file Data_Sheet_4.zip › 5. Detection results output by DCNN model (section Dataset optimization)/training dataset/Liangxing 99/3166MTL.jpg]

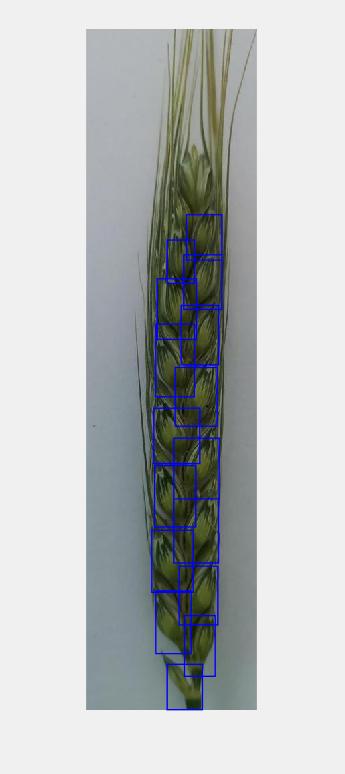

Supplement: Supplementary file 4 [file Data_Sheet_4.zip › 5. Detection results output by DCNN model (section Dataset optimization)/training dataset/Liangxing 99/3170MTL.jpg]

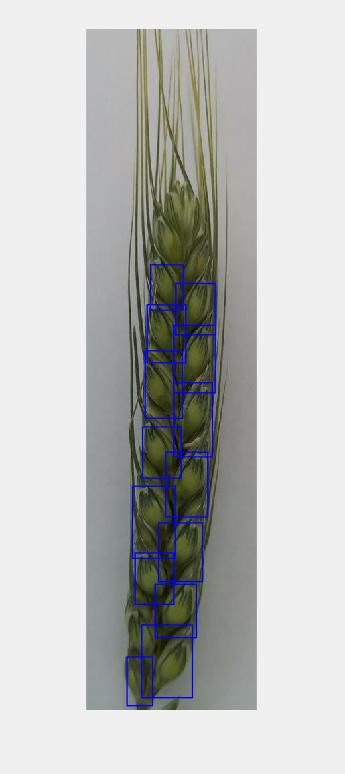

Supplement: Supplementary file 4 [file Data_Sheet_4.zip › 5. Detection results output by DCNN model (section Dataset optimization)/training dataset/Liangxing 99/3174MTL.jpg]

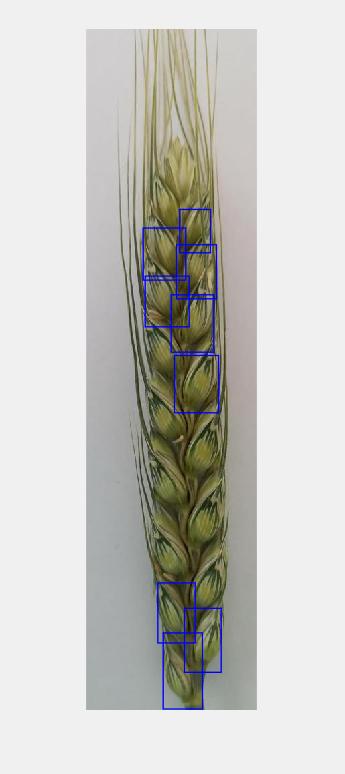

Supplement: Supplementary file 4 [file Data_Sheet_4.zip › 5. Detection results output by DCNN model (section Dataset optimization)/training dataset/Liangxing 99/3175MTL.jpg]

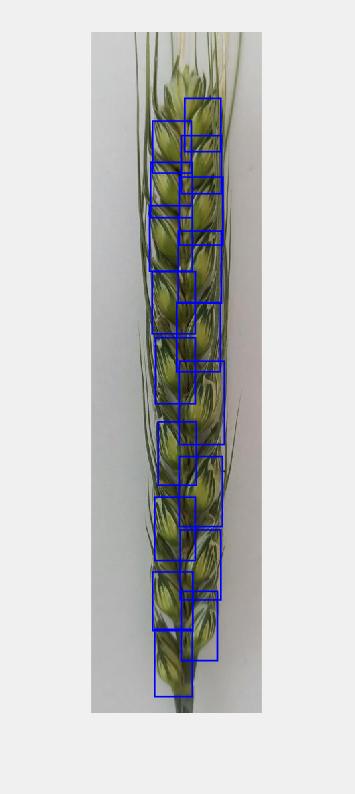

Supplement: Supplementary file 6 [file Data_Sheet_6.ZIP › 7. Detection results/Liangxing 99/3001MTL.jpg]

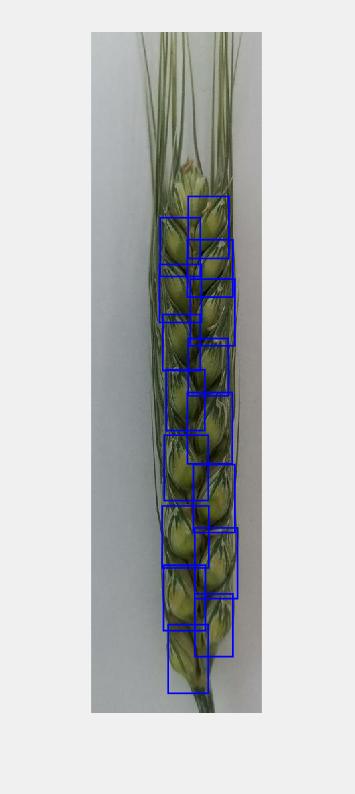

Supplement: Supplementary file 6 [file Data_Sheet_6.ZIP › 7. Detection results/Liangxing 99/3004MTL.jpg]

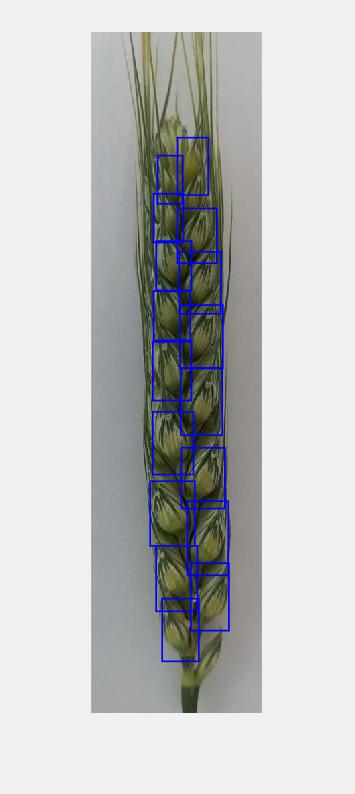

Supplement: Supplementary file 6 [file Data_Sheet_6.ZIP › 7. Detection results/Liangxing 99/3006MTL.jpg]

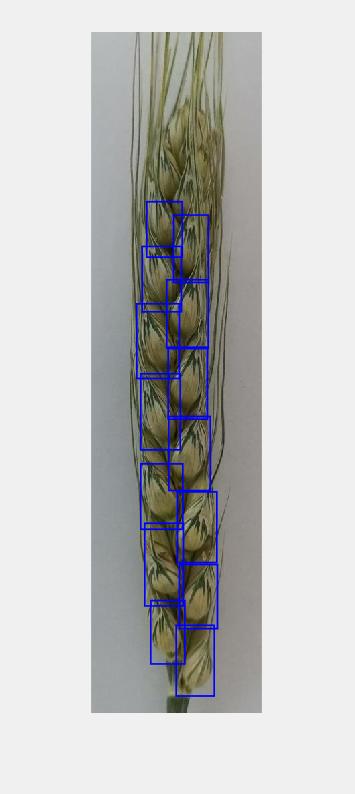

Supplement: Supplementary file 6 [file Data_Sheet_6.ZIP › 7. Detection results/Liangxing 99/3007MTL.jpg]

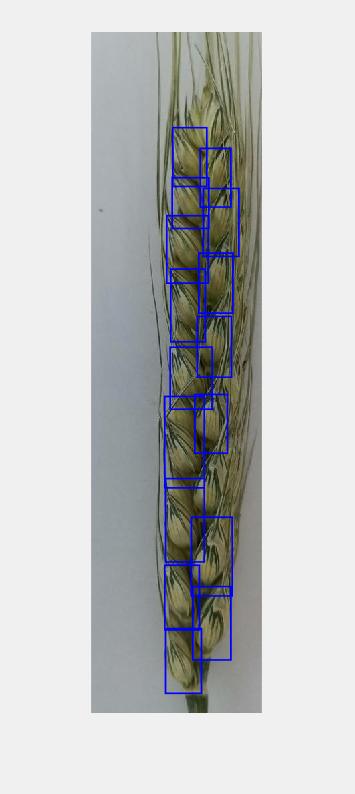

Supplement: Supplementary file 6 [file Data_Sheet_6.ZIP › 7. Detection results/Liangxing 99/3008MTL.jpg]

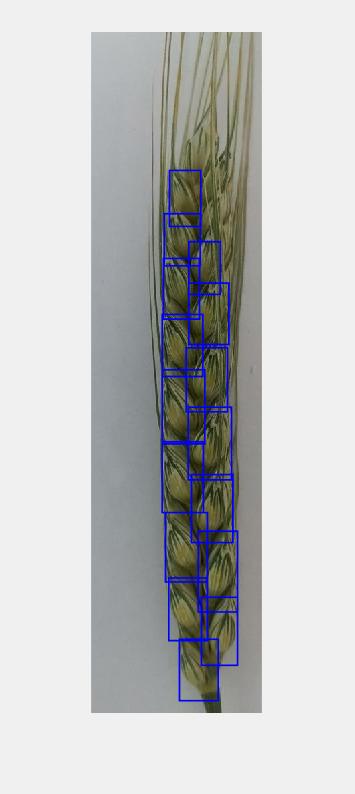

Supplement: Supplementary file 6 [file Data_Sheet_6.ZIP › 7. Detection results/Liangxing 99/3013MTL.jpg]

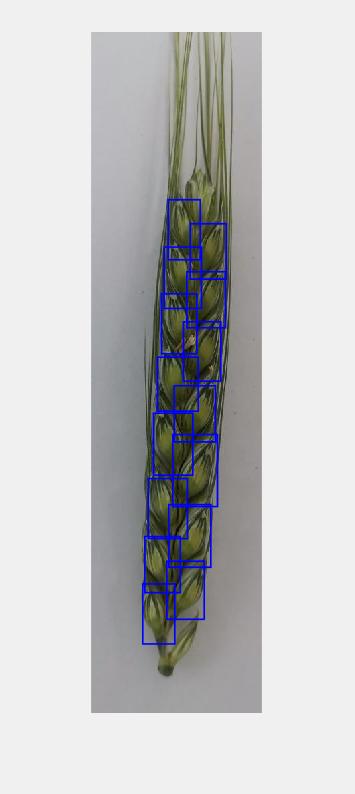

Supplement: Supplementary file 6 [file Data_Sheet_6.ZIP › 7. Detection results/Liangxing 99/3016MTL.jpg]

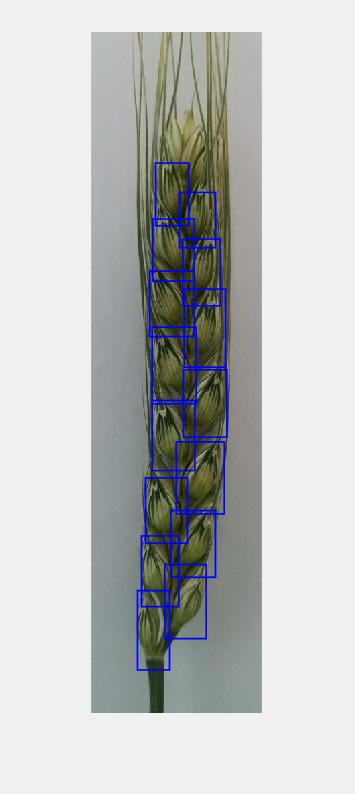

Supplement: Supplementary file 6 [file Data_Sheet_6.ZIP › 7. Detection results/Liangxing 99/3019MTL.jpg]

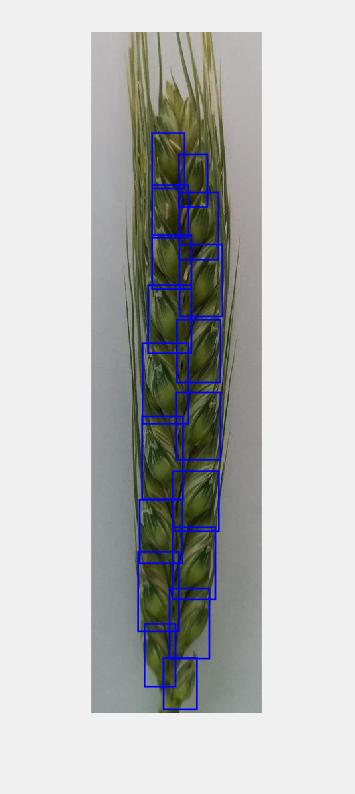

Supplement: Supplementary file 6 [file Data_Sheet_6.ZIP › 7. Detection results/Liangxing 99/3024MTL.jpg]

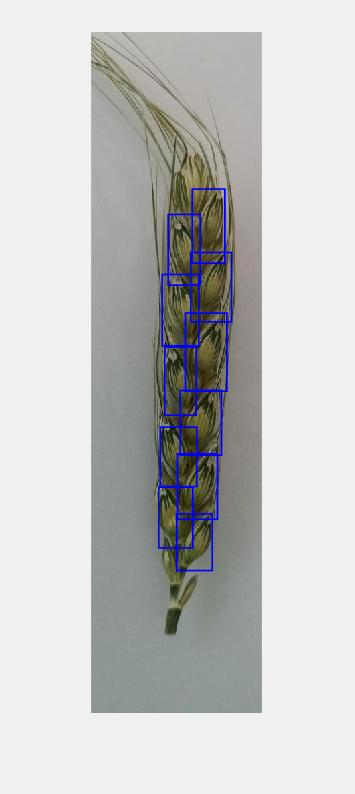

Supplement: Supplementary file 6 [file Data_Sheet_6.ZIP › 7. Detection results/Liangxing 99/3025MTL.jpg]

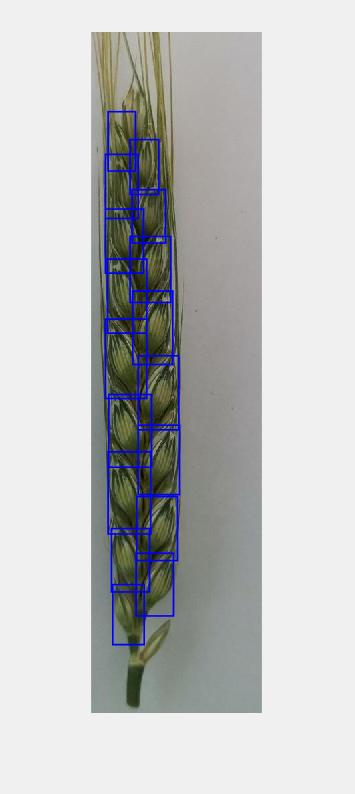

Supplement: Supplementary file 6 [file Data_Sheet_6.ZIP › 7. Detection results/Liangxing 99/3029MTL.jpg]

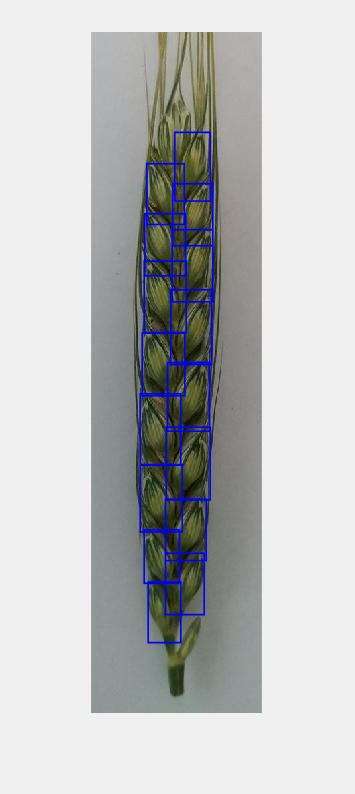

Supplement: Supplementary file 6 [file Data_Sheet_6.ZIP › 7. Detection results/Liangxing 99/3032MTL.jpg]

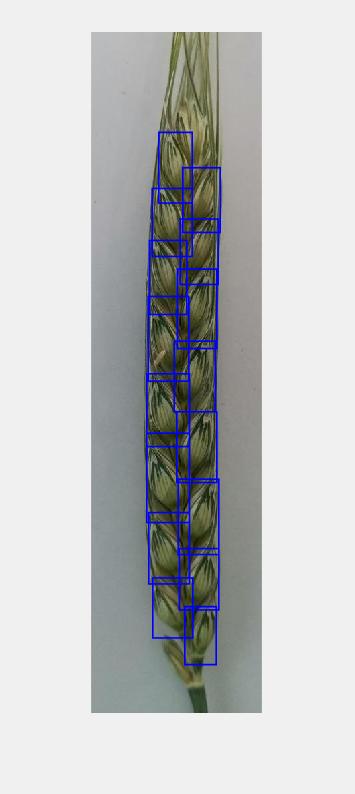

Supplement: Supplementary file 6 [file Data_Sheet_6.ZIP › 7. Detection results/Liangxing 99/3035MTL.jpg]

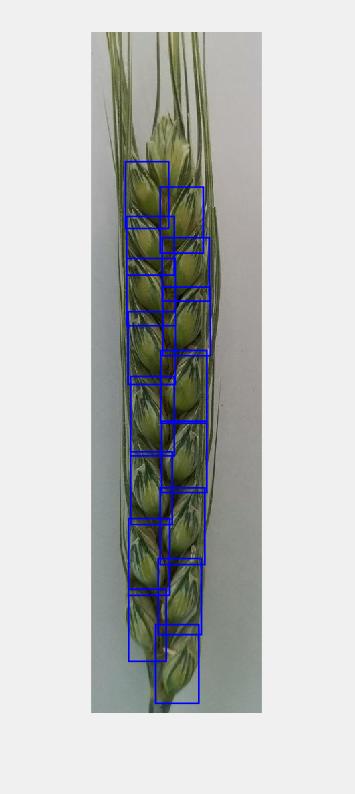

Supplement: Supplementary file 6 [file Data_Sheet_6.ZIP › 7. Detection results/Liangxing 99/3038MTL.jpg]

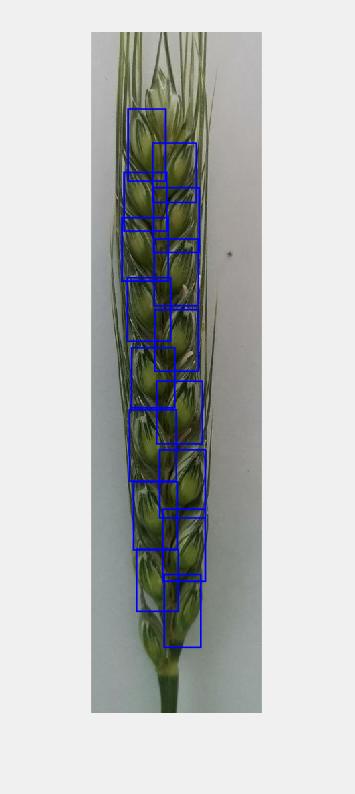

Supplement: Supplementary file 6 [file Data_Sheet_6.ZIP › 7. Detection results/Liangxing 99/3044MTL.jpg]

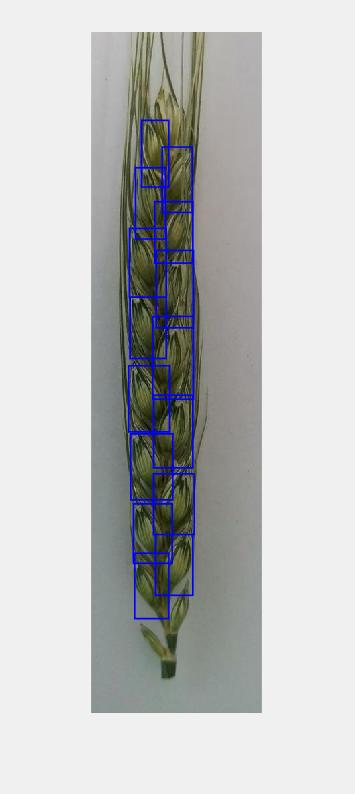

Supplement: Supplementary file 6 [file Data_Sheet_6.ZIP › 7. Detection results/Liangxing 99/3045MTL.jpg]

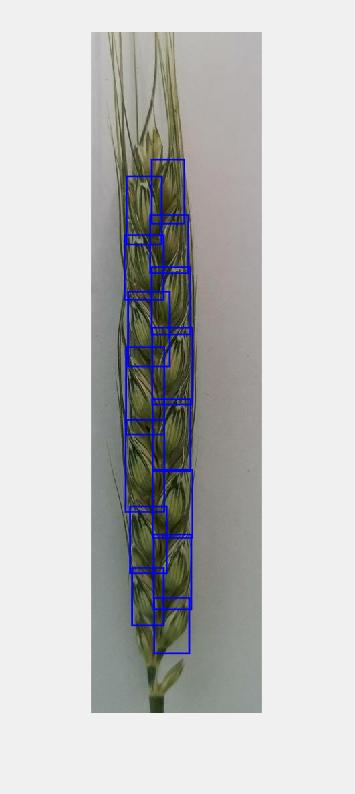

Supplement: Supplementary file 6 [file Data_Sheet_6.ZIP › 7. Detection results/Liangxing 99/3046MTL.jpg]

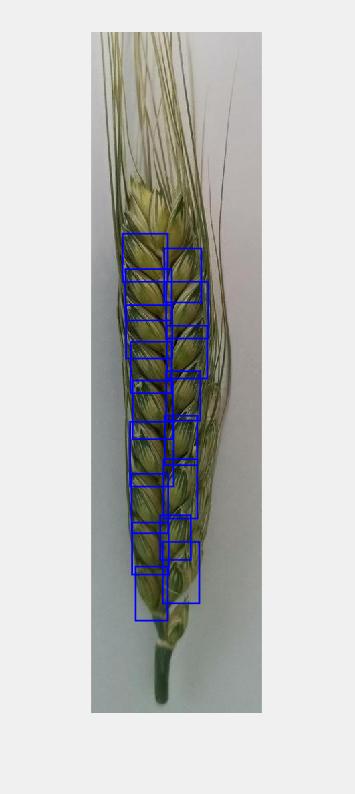

Supplement: Supplementary file 6 [file Data_Sheet_6.ZIP › 7. Detection results/Liangxing 99/3047MTL.jpg]

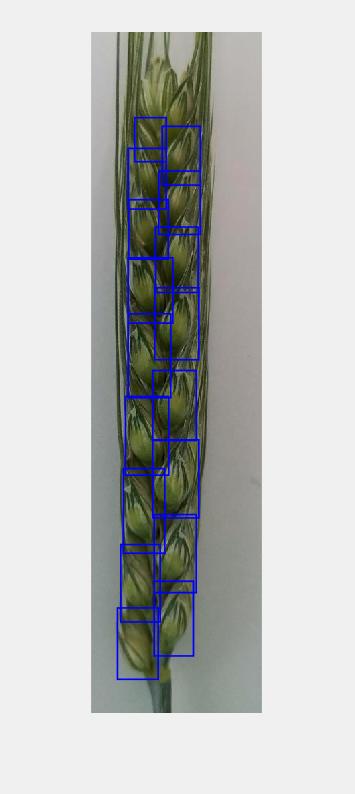

Supplement: Supplementary file 6 [file Data_Sheet_6.ZIP › 7. Detection results/Liangxing 99/3048MTL.jpg]

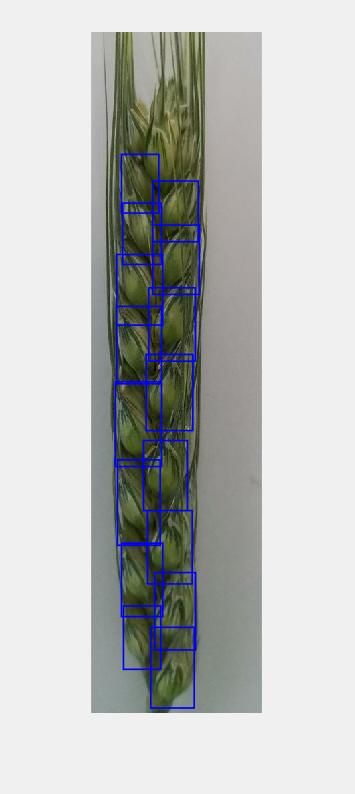

Supplement: Supplementary file 6 [file Data_Sheet_6.ZIP › 7. Detection results/Liangxing 99/3049MTL.jpg]

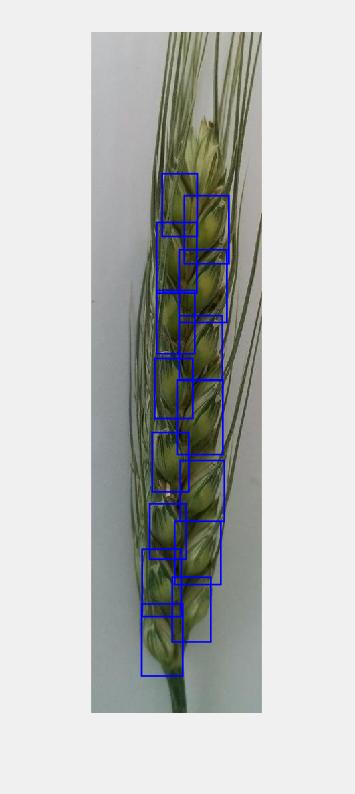

Supplement: Supplementary file 6 [file Data_Sheet_6.ZIP › 7. Detection results/Liangxing 99/3050MTL.jpg]

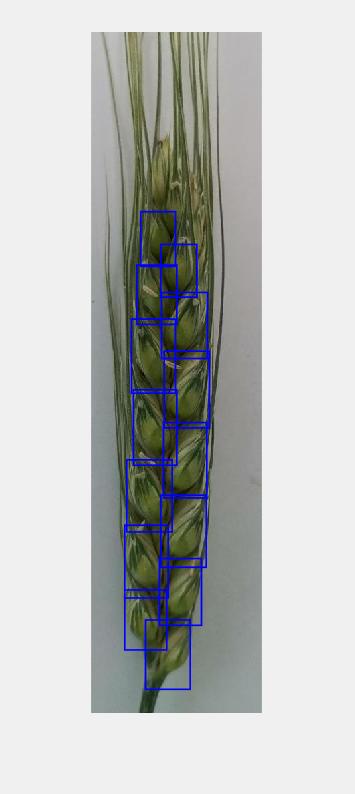

Supplement: Supplementary file 6 [file Data_Sheet_6.ZIP › 7. Detection results/Liangxing 99/3051MTL.jpg]

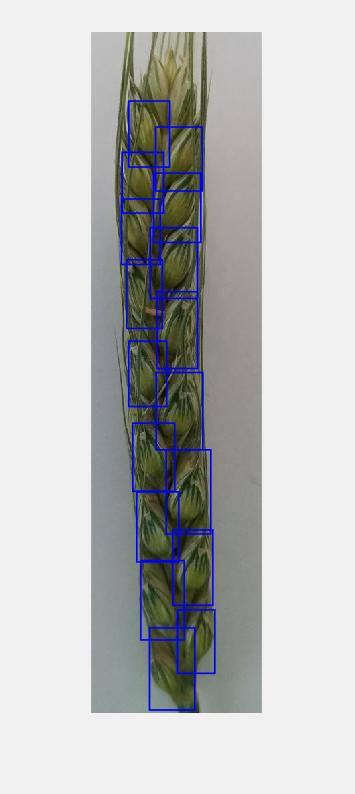

Supplement: Supplementary file 6 [file Data_Sheet_6.ZIP › 7. Detection results/Liangxing 99/3053MTL.jpg]

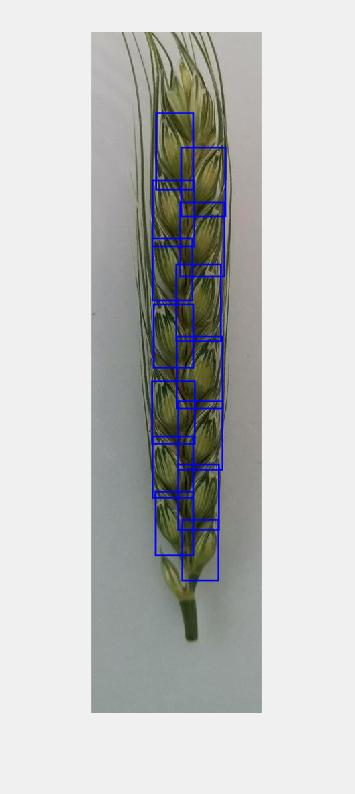

Supplement: Supplementary file 6 [file Data_Sheet_6.ZIP › 7. Detection results/Liangxing 99/3054MTL.jpg]

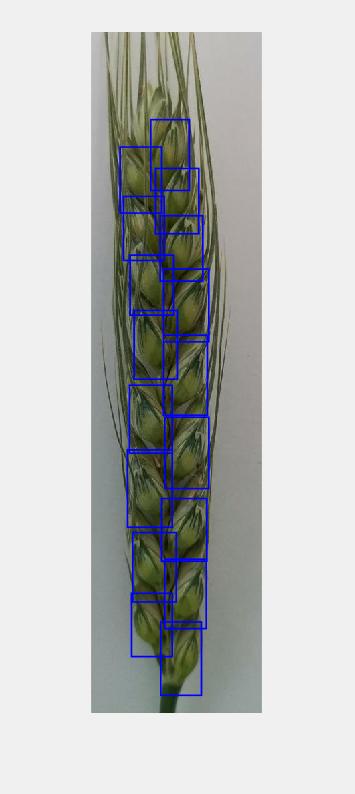

Supplement: Supplementary file 6 [file Data_Sheet_6.ZIP › 7. Detection results/Liangxing 99/3056MTL.jpg]

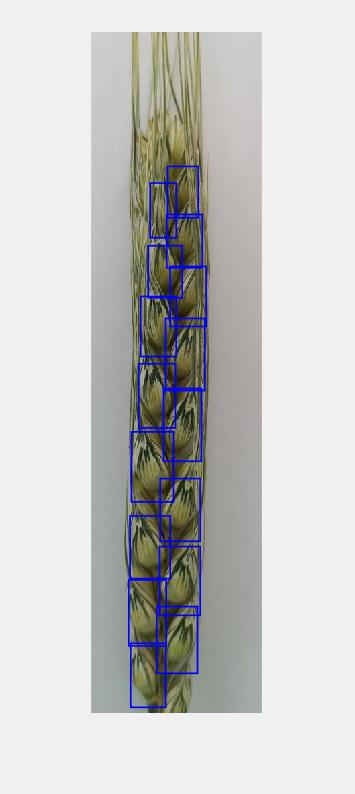

Supplement: Supplementary file 6 [file Data_Sheet_6.ZIP › 7. Detection results/Liangxing 99/3062MTL.jpg]

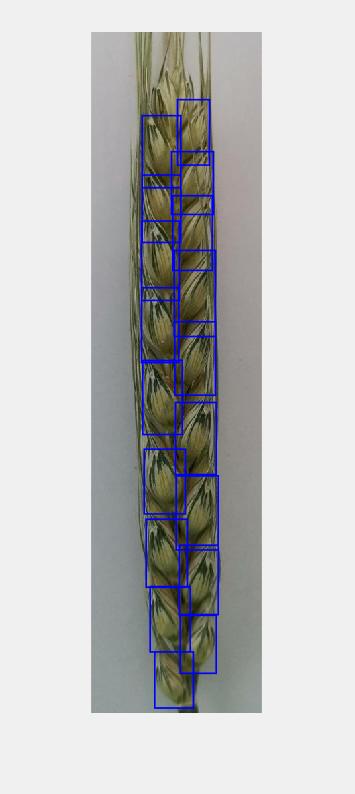

Supplement: Supplementary file 6 [file Data_Sheet_6.ZIP › 7. Detection results/Liangxing 99/3063MTL.jpg]

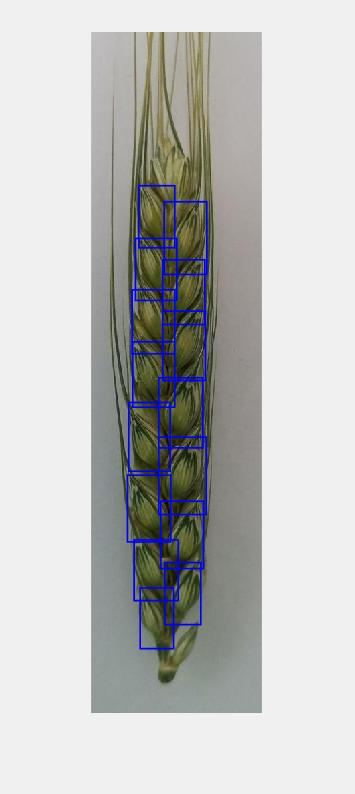

Supplement: Supplementary file 6 [file Data_Sheet_6.ZIP › 7. Detection results/Liangxing 99/3065MTL.jpg]

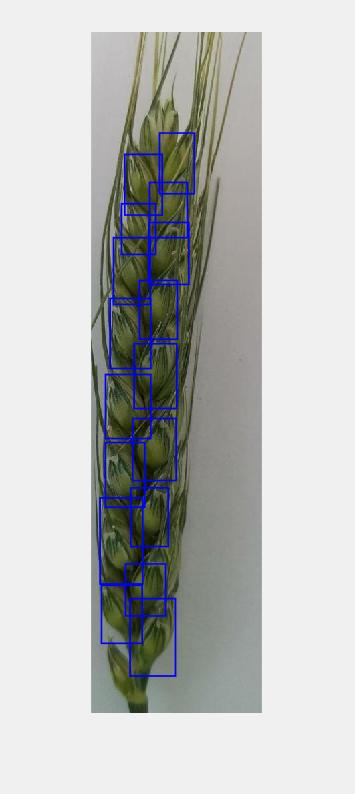

Supplement: Supplementary file 6 [file Data_Sheet_6.ZIP › 7. Detection results/Liangxing 99/3066MTL.jpg]

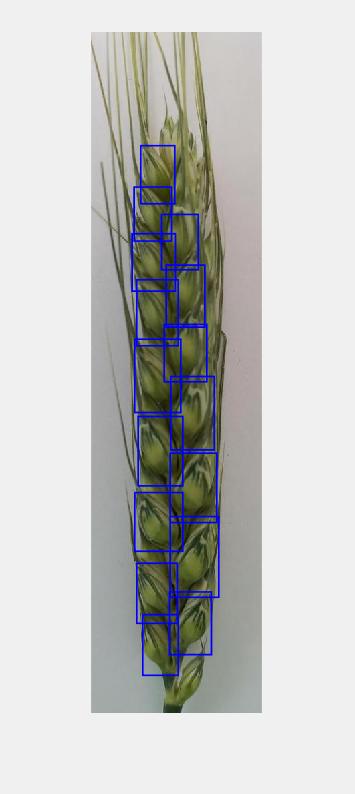

Supplement: Supplementary file 6 [file Data_Sheet_6.ZIP › 7. Detection results/Liangxing 99/3067MTL.jpg]

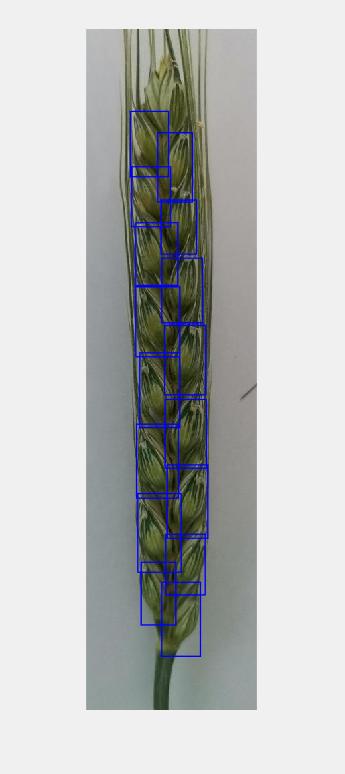

Supplement: Supplementary file 6 [file Data_Sheet_6.ZIP › 7. Detection results/Liangxing 99/3068MTL.jpg]

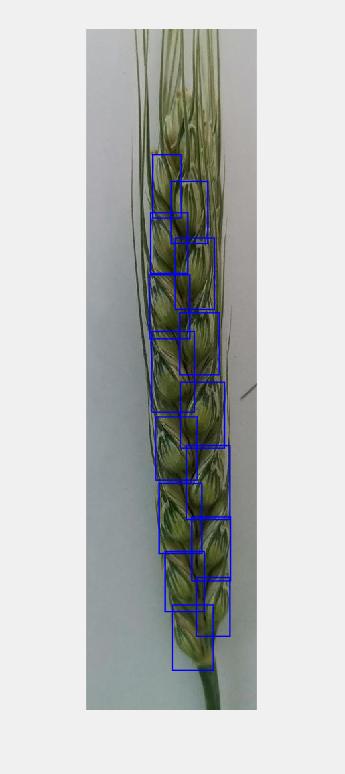

Supplement: Supplementary file 6 [file Data_Sheet_6.ZIP › 7. Detection results/Liangxing 99/3069MTL.jpg]

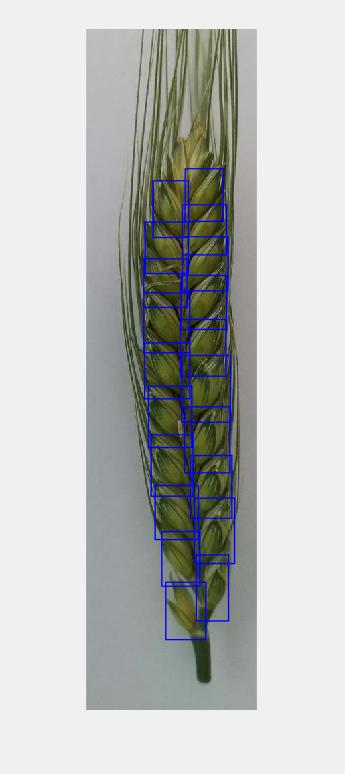

Supplement: Supplementary file 6 [file Data_Sheet_6.ZIP › 7. Detection results/Liangxing 99/3074MTL.jpg]

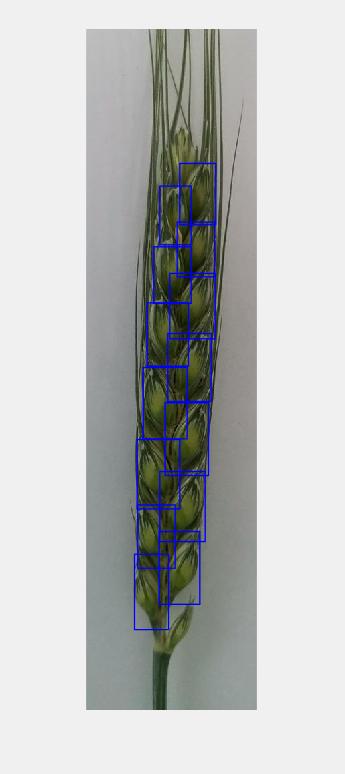

Supplement: Supplementary file 6 [file Data_Sheet_6.ZIP › 7. Detection results/Liangxing 99/3079MTL.jpg]

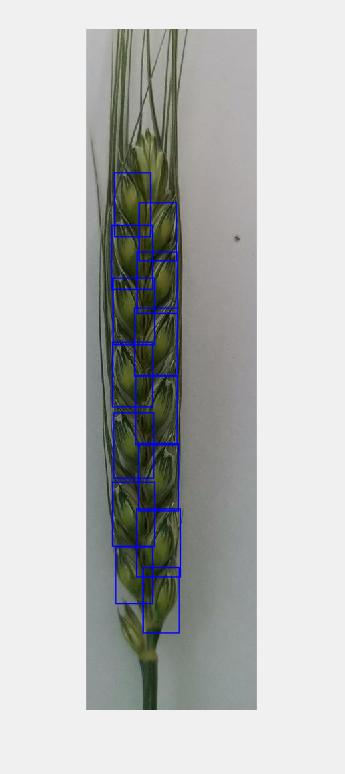

Supplement: Supplementary file 6 [file Data_Sheet_6.ZIP › 7. Detection results/Liangxing 99/3080MTL.jpg]

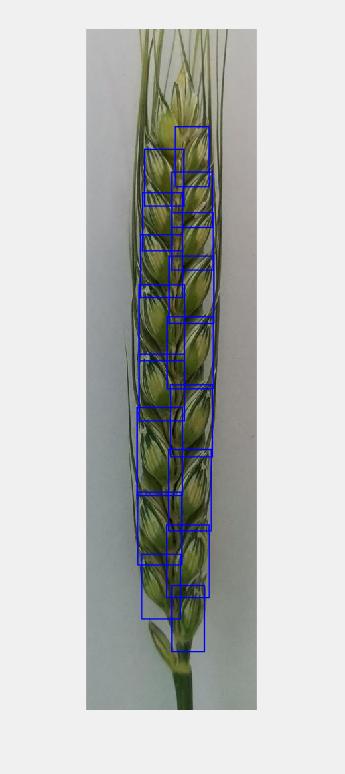

Supplement: Supplementary file 6 [file Data_Sheet_6.ZIP › 7. Detection results/Liangxing 99/3084MTL.jpg]

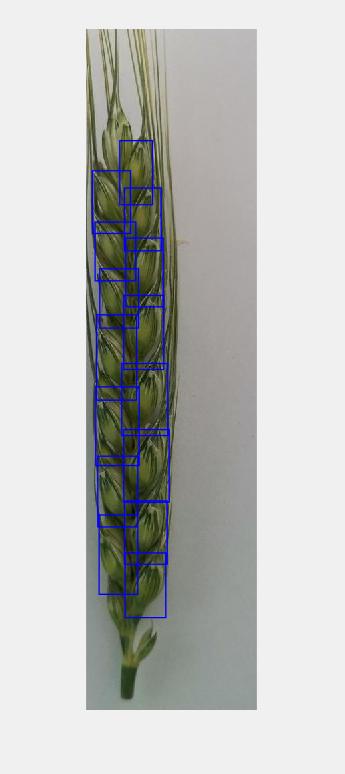

Supplement: Supplementary file 6 [file Data_Sheet_6.ZIP › 7. Detection results/Liangxing 99/3085MTL.jpg]

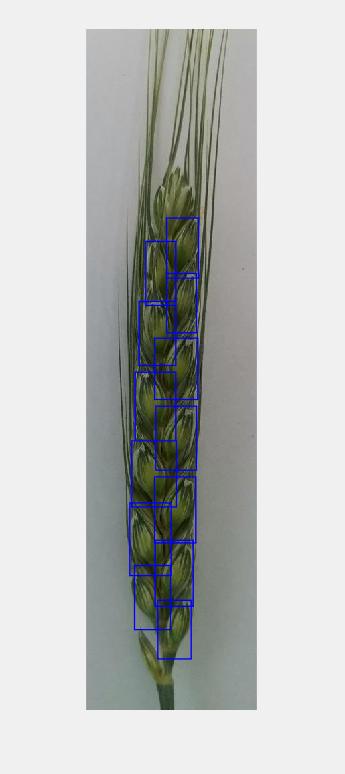

Supplement: Supplementary file 6 [file Data_Sheet_6.ZIP › 7. Detection results/Liangxing 99/3089MTL.jpg]

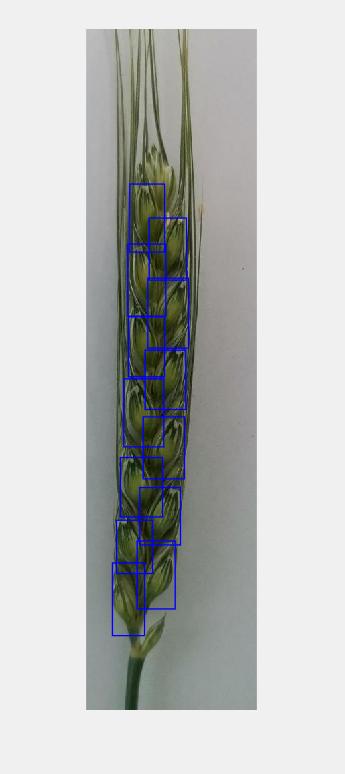

Supplement: Supplementary file 6 [file Data_Sheet_6.ZIP › 7. Detection results/Liangxing 99/3090MTL.jpg]

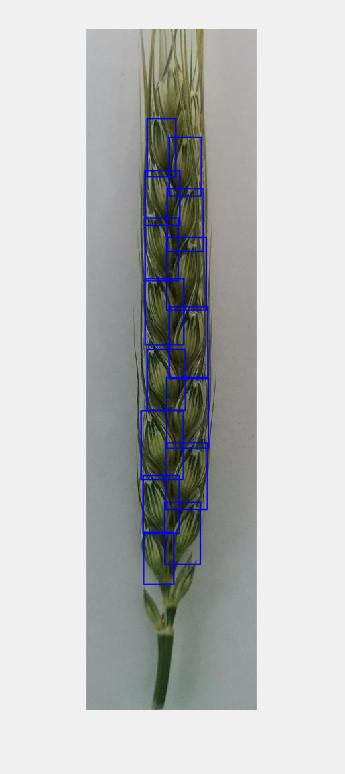

Supplement: Supplementary file 6 [file Data_Sheet_6.ZIP › 7. Detection results/Liangxing 99/3091MTL.jpg]

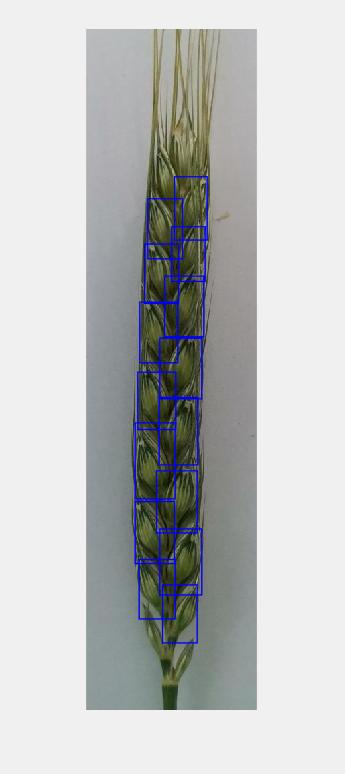

Supplement: Supplementary file 6 [file Data_Sheet_6.ZIP › 7. Detection results/Liangxing 99/3092MTL.jpg]

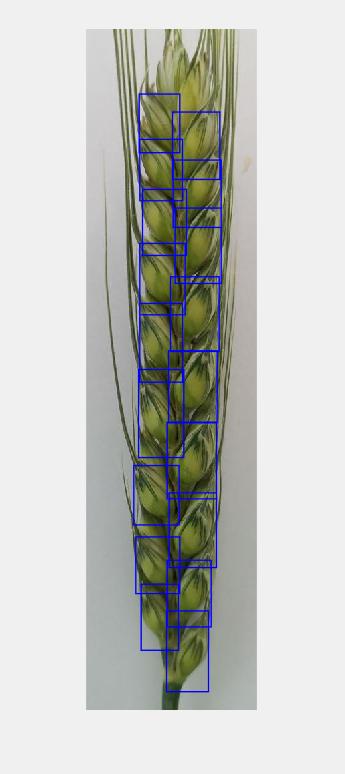

Supplement: Supplementary file 6 [file Data_Sheet_6.ZIP › 7. Detection results/Liangxing 99/3093MTL.jpg]

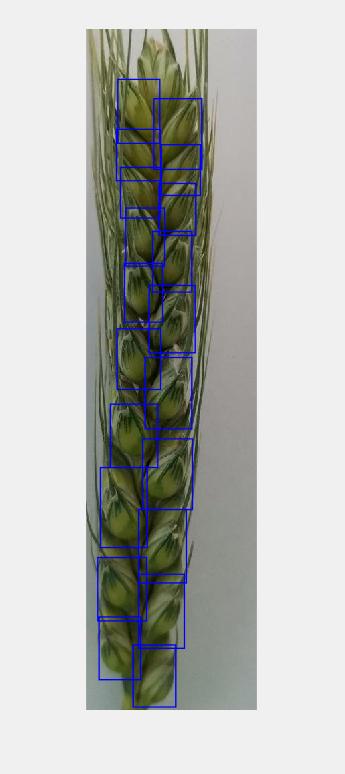

Supplement: Supplementary file 6 [file Data_Sheet_6.ZIP › 7. Detection results/Liangxing 99/3095MTL.jpg]

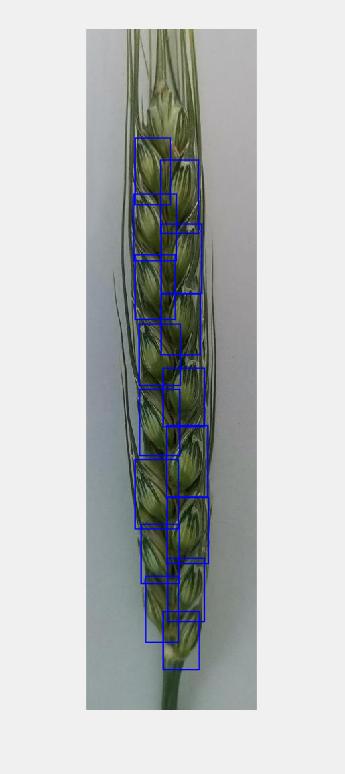

Supplement: Supplementary file 6 [file Data_Sheet_6.ZIP › 7. Detection results/Liangxing 99/3098MTL.jpg]

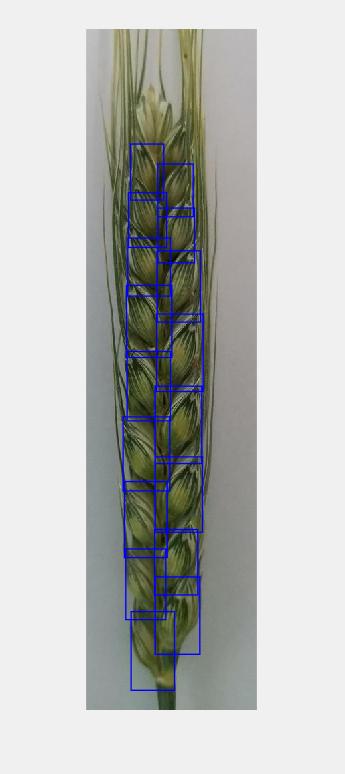

Supplement: Supplementary file 6 [file Data_Sheet_6.ZIP › 7. Detection results/Liangxing 99/3103MTL.jpg]

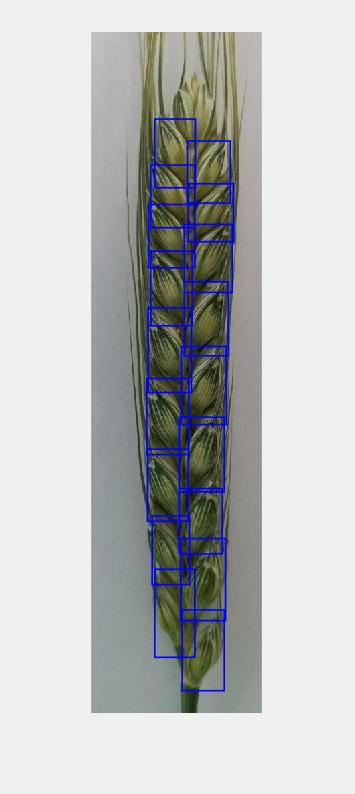

Supplement: Supplementary file 6 [file Data_Sheet_6.ZIP › 7. Detection results/Liangxing 99/3104MTL.jpg]

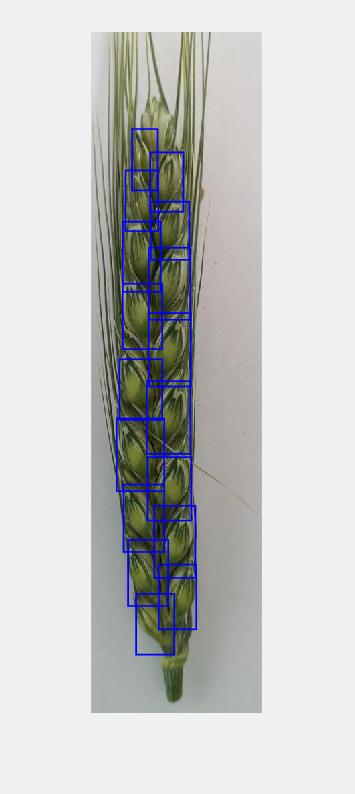

Supplement: Supplementary file 6 [file Data_Sheet_6.ZIP › 7. Detection results/Liangxing 99/3107MTL.jpg]

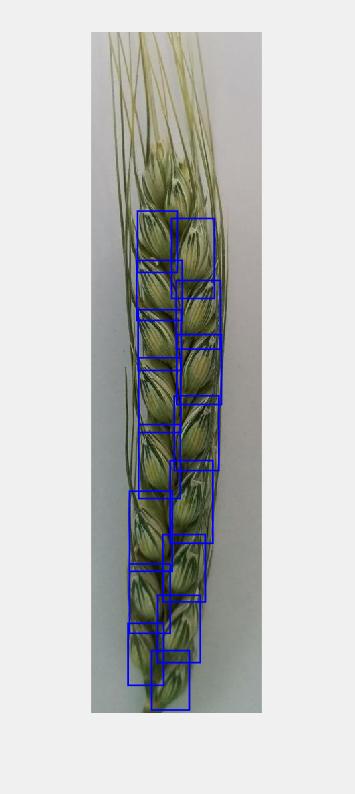

Supplement: Supplementary file 6 [file Data_Sheet_6.ZIP › 7. Detection results/Liangxing 99/3109MTL.jpg]

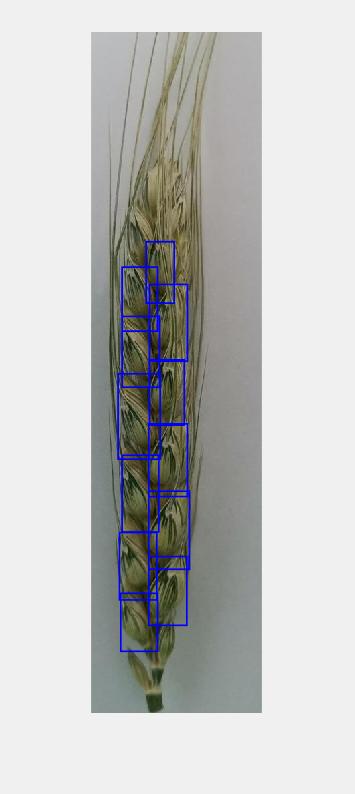

Supplement: Supplementary file 6 [file Data_Sheet_6.ZIP › 7. Detection results/Liangxing 99/3112MTL.jpg]

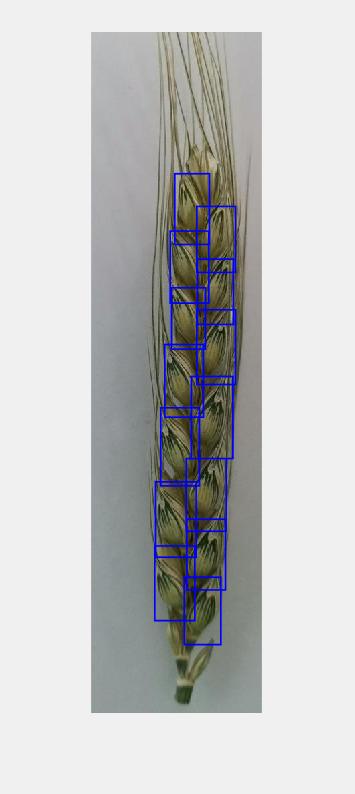

Supplement: Supplementary file 6 [file Data_Sheet_6.ZIP › 7. Detection results/Liangxing 99/3113MTL.jpg]

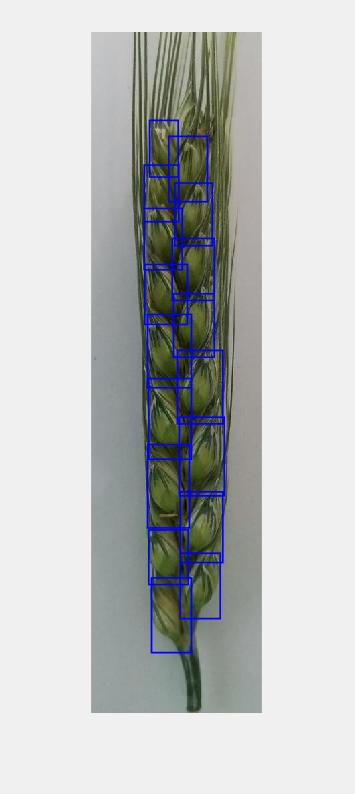

Supplement: Supplementary file 6 [file Data_Sheet_6.ZIP › 7. Detection results/Liangxing 99/3114MTL.jpg]

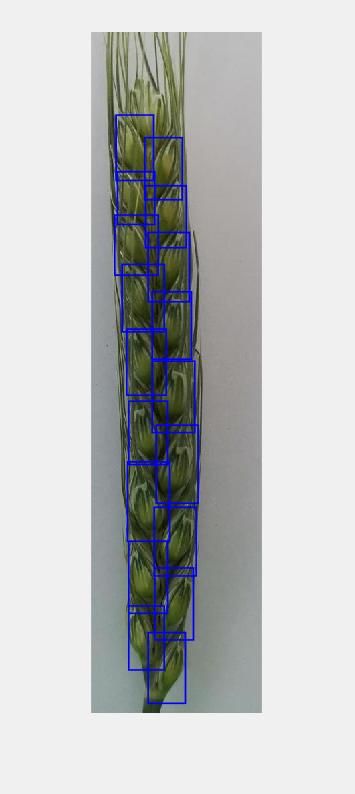

Supplement: Supplementary file 6 [file Data_Sheet_6.ZIP › 7. Detection results/Liangxing 99/3117MTL.jpg]

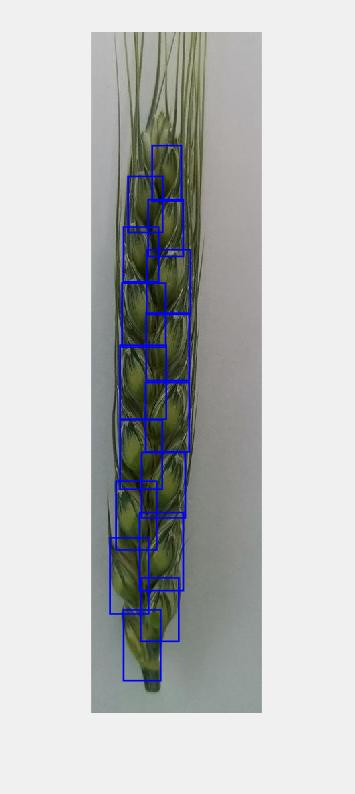

Supplement: Supplementary file 6 [file Data_Sheet_6.ZIP › 7. Detection results/Liangxing 99/3119MTL.jpg]

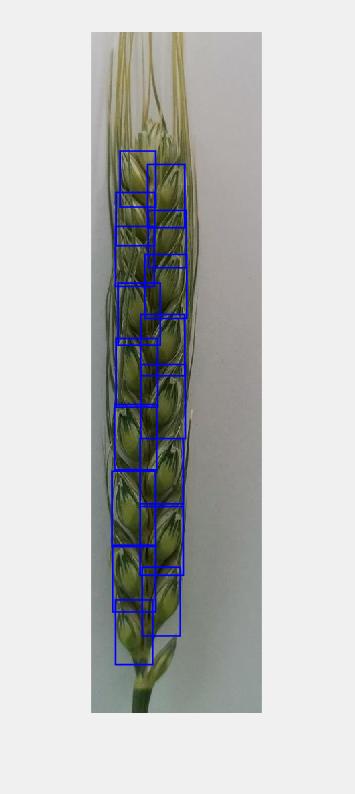

Supplement: Supplementary file 6 [file Data_Sheet_6.ZIP › 7. Detection results/Liangxing 99/3121MTL.jpg]

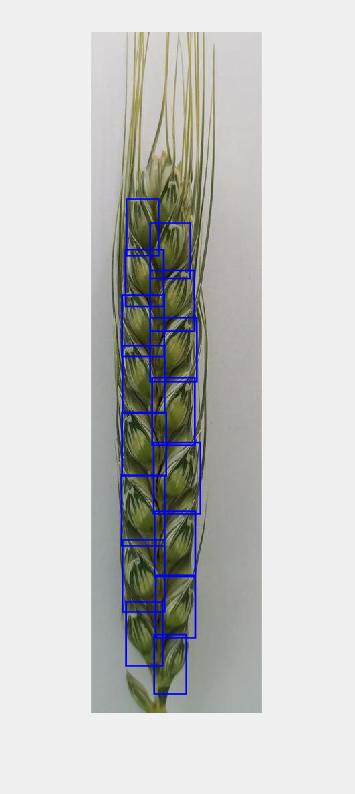

Supplement: Supplementary file 6 [file Data_Sheet_6.ZIP › 7. Detection results/Liangxing 99/3122MTL.jpg]

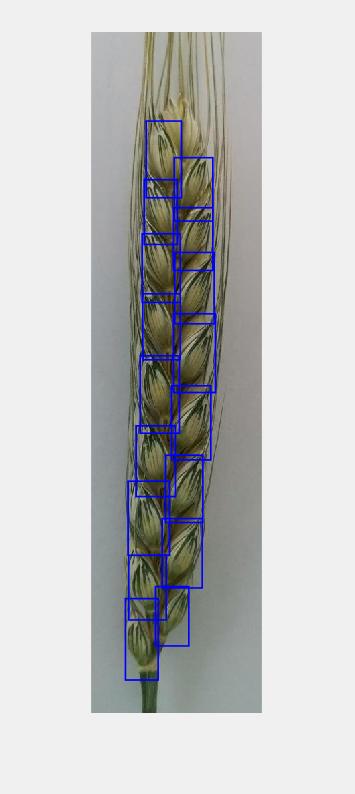

Supplement: Supplementary file 6 [file Data_Sheet_6.ZIP › 7. Detection results/Liangxing 99/3123MTL.jpg]

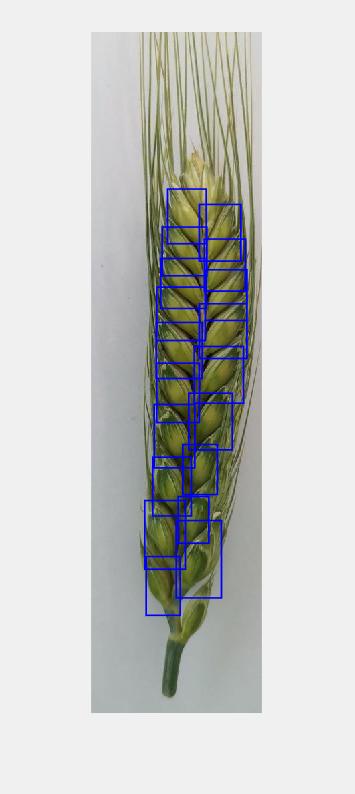

Supplement: Supplementary file 6 [file Data_Sheet_6.ZIP › 7. Detection results/Liangxing 99/3125MTL.jpg]

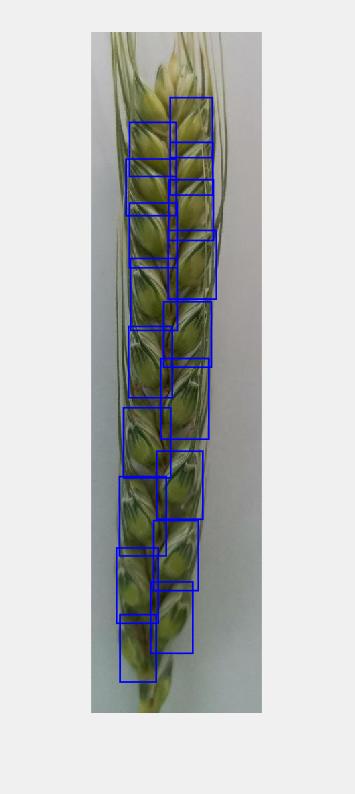

Supplement: Supplementary file 6 [file Data_Sheet_6.ZIP › 7. Detection results/Liangxing 99/3126MTL.jpg]

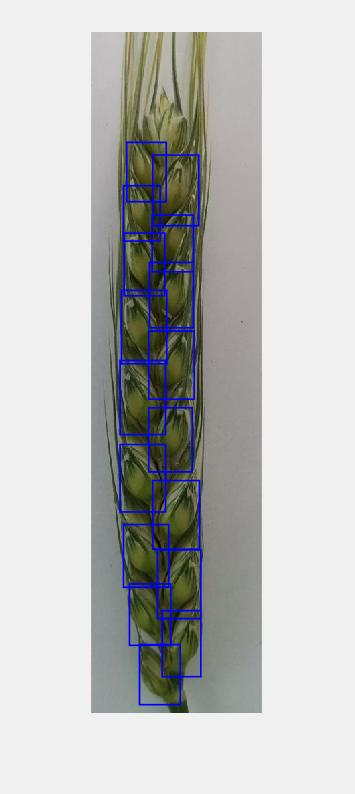

Supplement: Supplementary file 6 [file Data_Sheet_6.ZIP › 7. Detection results/Liangxing 99/3130MTL.jpg]

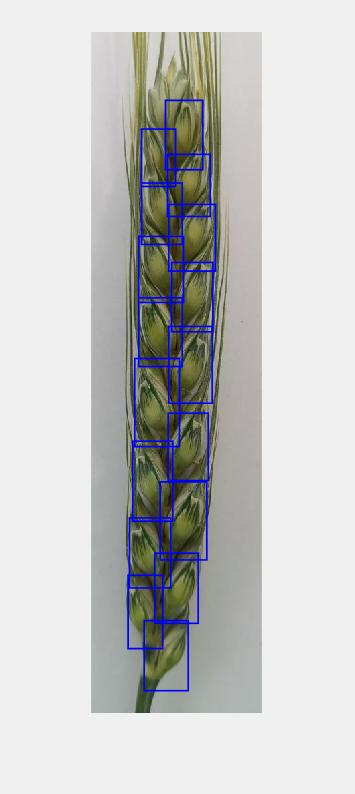

Supplement: Supplementary file 6 [file Data_Sheet_6.ZIP › 7. Detection results/Liangxing 99/3131MTL.jpg]

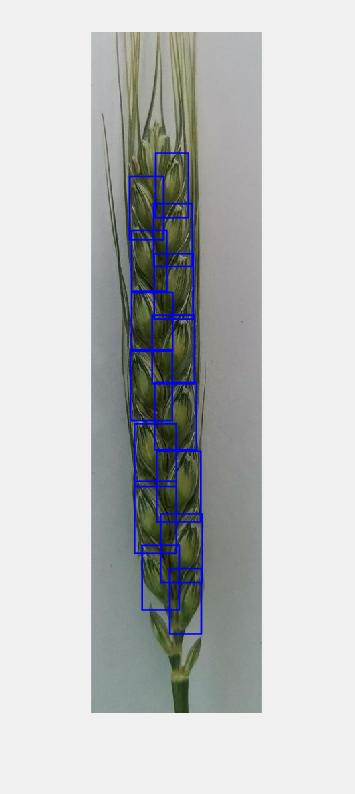

Supplement: Supplementary file 6 [file Data_Sheet_6.ZIP › 7. Detection results/Liangxing 99/3132MTL.jpg]

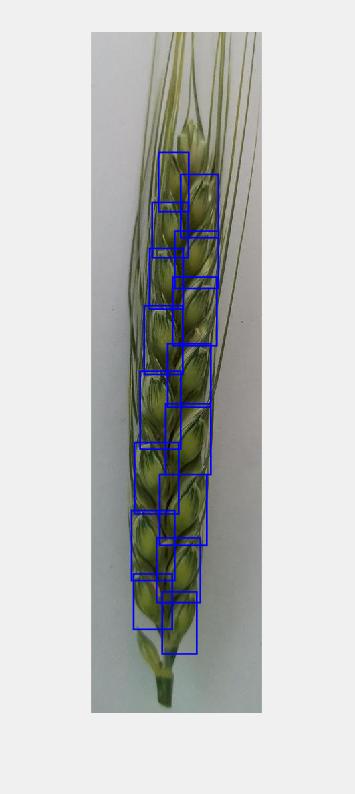

Supplement: Supplementary file 6 [file Data_Sheet_6.ZIP › 7. Detection results/Liangxing 99/3135MTL.jpg]

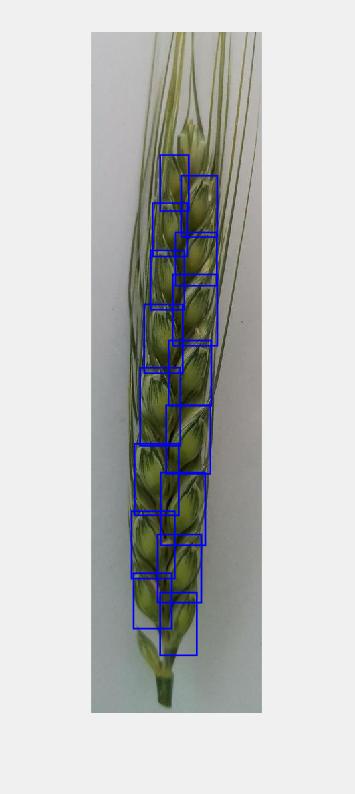

Supplement: Supplementary file 6 [file Data_Sheet_6.ZIP › 7. Detection results/Liangxing 99/3136MTL.jpg]

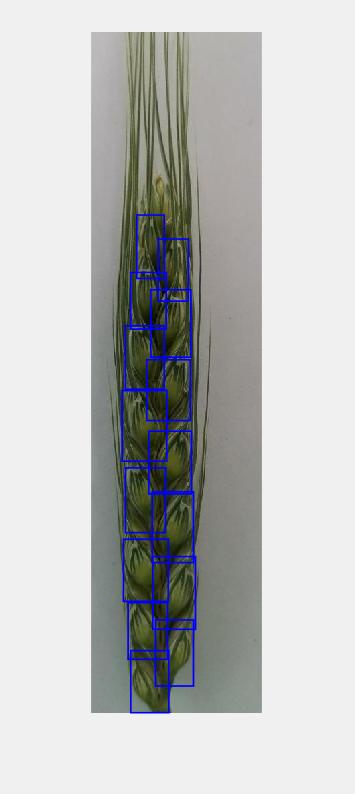

Supplement: Supplementary file 6 [file Data_Sheet_6.ZIP › 7. Detection results/Liangxing 99/3137MTL.jpg]

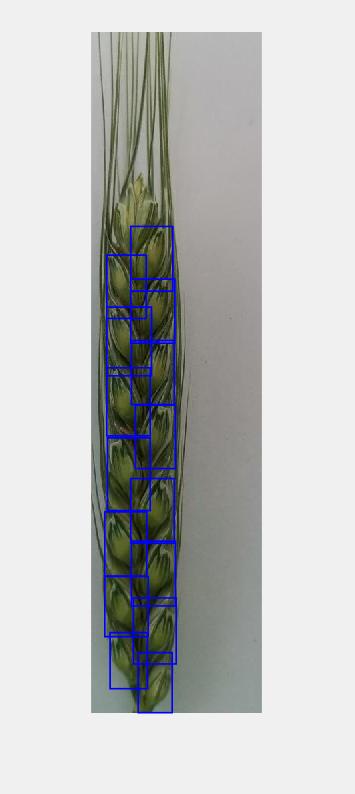

Supplement: Supplementary file 6 [file Data_Sheet_6.ZIP › 7. Detection results/Liangxing 99/3138MTL.jpg]

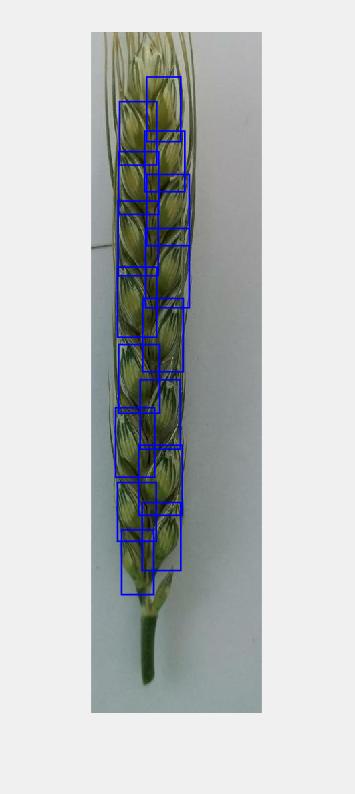

Supplement: Supplementary file 6 [file Data_Sheet_6.ZIP › 7. Detection results/Liangxing 99/3139MTL.jpg]

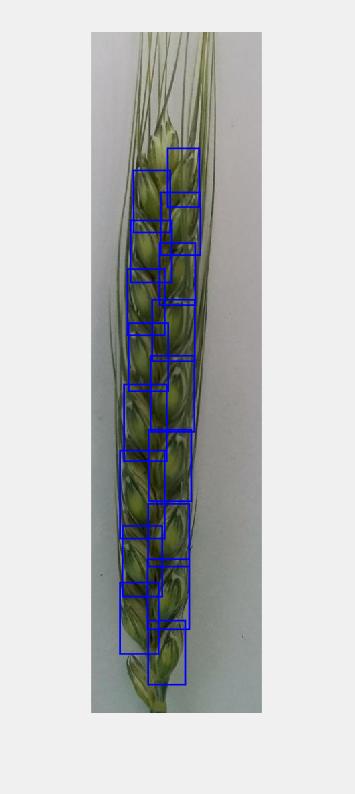

Supplement: Supplementary file 6 [file Data_Sheet_6.ZIP › 7. Detection results/Liangxing 99/3141MTL.jpg]

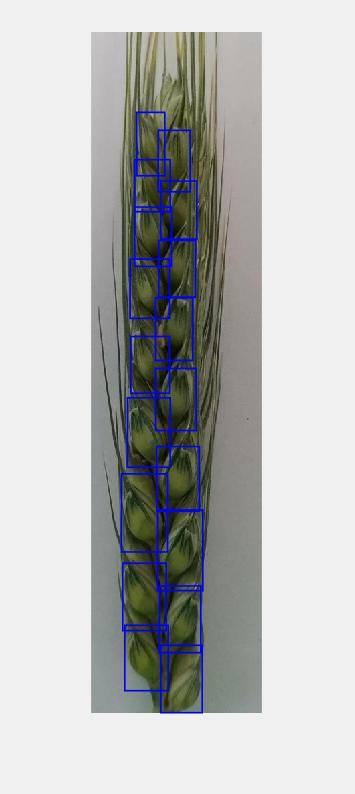

Supplement: Supplementary file 6 [file Data_Sheet_6.ZIP › 7. Detection results/Liangxing 99/3142MTL.jpg]

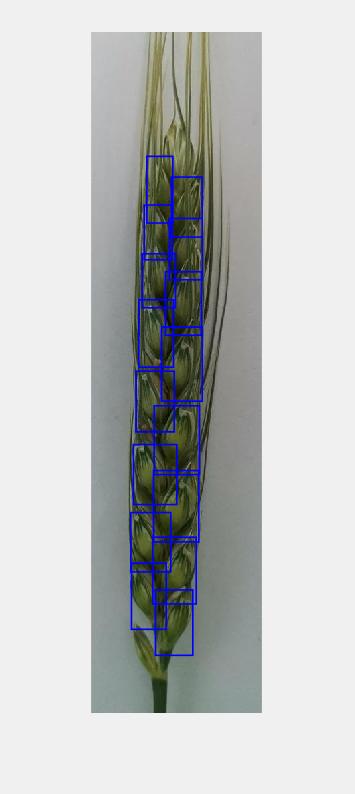

Supplement: Supplementary file 6 [file Data_Sheet_6.ZIP › 7. Detection results/Liangxing 99/3144MTL.jpg]

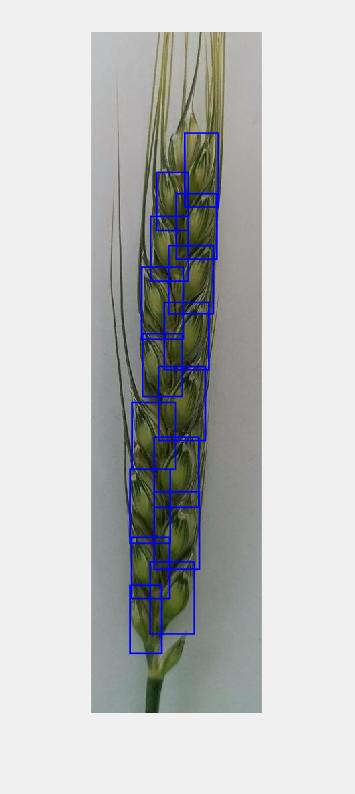

Supplement: Supplementary file 6 [file Data_Sheet_6.ZIP › 7. Detection results/Liangxing 99/3145MTL.jpg]
