# Supplementary figures and images for: Automatic Detection and Counting of Wheat Spikelet Using Semi-Automatic Labeling and Deep Learning (part 8 of 8)
Source: Front Plant Sci. 2022 May 30;13:872555. doi: 10.3389/fpls.2022.872555 (PMC9189412; doi:10.3389/fpls.2022.872555)

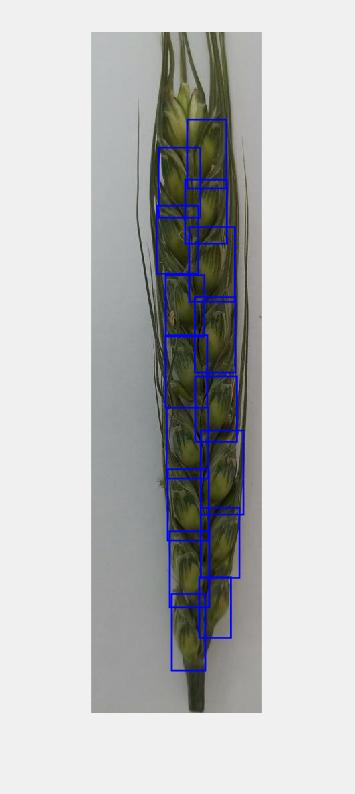

Supplement: Supplementary file 6 [file Data_Sheet_6.ZIP › 7. Detection results/Shannong 25/2001MTL.jpg]

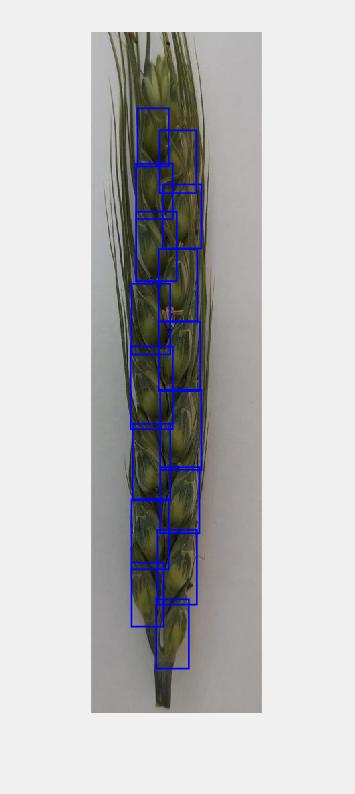

Supplement: Supplementary file 6 [file Data_Sheet_6.ZIP › 7. Detection results/Shannong 25/2002MTL.jpg]

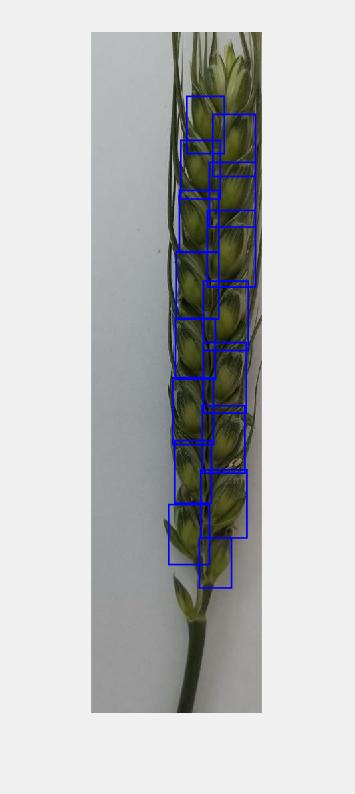

Supplement: Supplementary file 6 [file Data_Sheet_6.ZIP › 7. Detection results/Shannong 25/2005MTL.jpg]

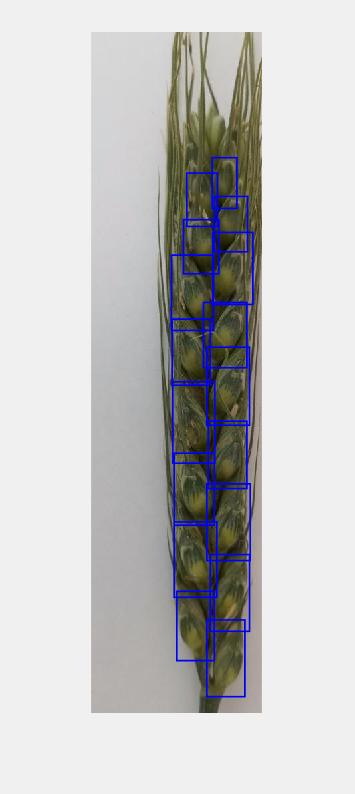

Supplement: Supplementary file 6 [file Data_Sheet_6.ZIP › 7. Detection results/Shannong 25/2007MTL.jpg]

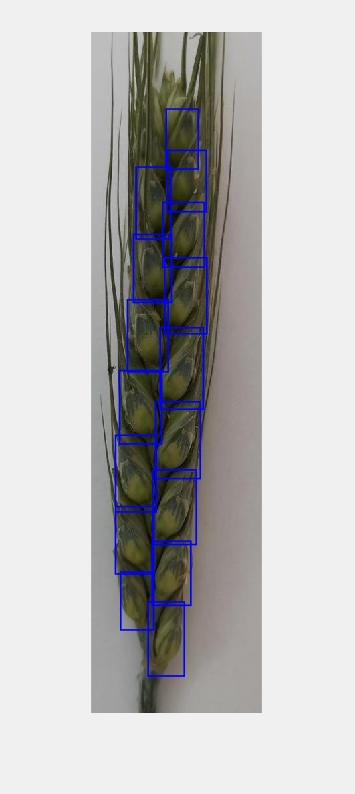

Supplement: Supplementary file 6 [file Data_Sheet_6.ZIP › 7. Detection results/Shannong 25/2010MTL.jpg]

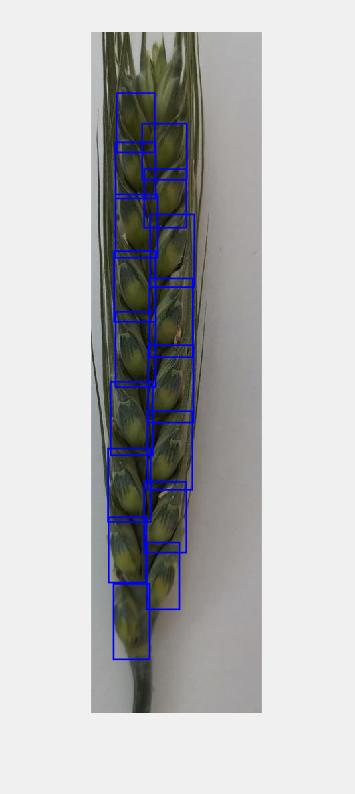

Supplement: Supplementary file 6 [file Data_Sheet_6.ZIP › 7. Detection results/Shannong 25/2011MTL.jpg]

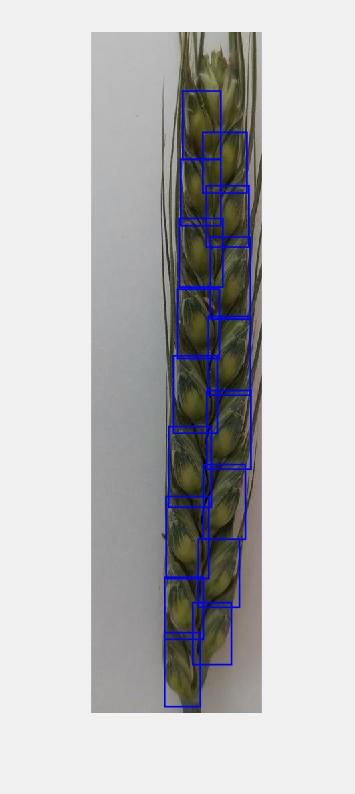

Supplement: Supplementary file 6 [file Data_Sheet_6.ZIP › 7. Detection results/Shannong 25/2013MTL.jpg]

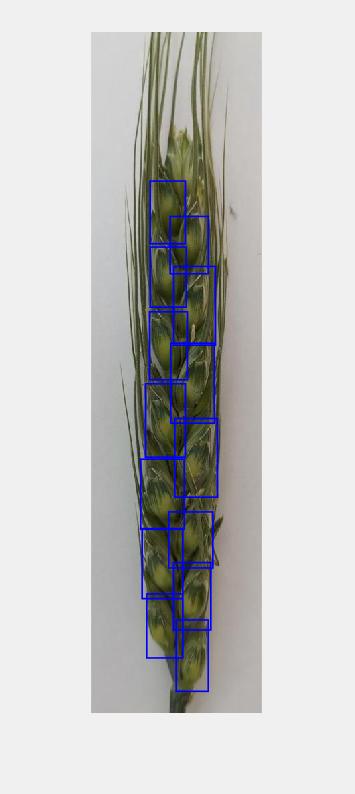

Supplement: Supplementary file 6 [file Data_Sheet_6.ZIP › 7. Detection results/Shannong 25/2017MTL.jpg]

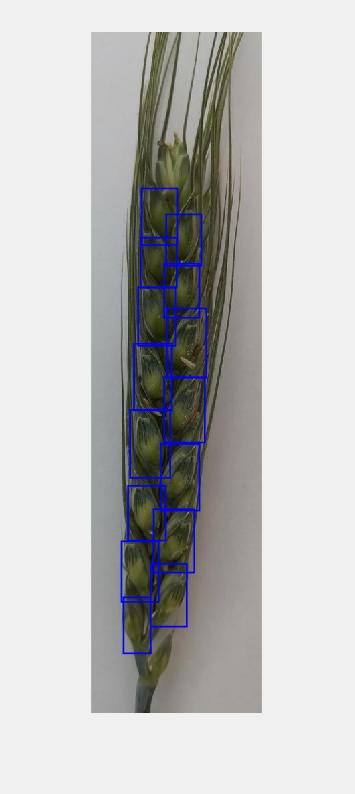

Supplement: Supplementary file 6 [file Data_Sheet_6.ZIP › 7. Detection results/Shannong 25/2018MTL.jpg]

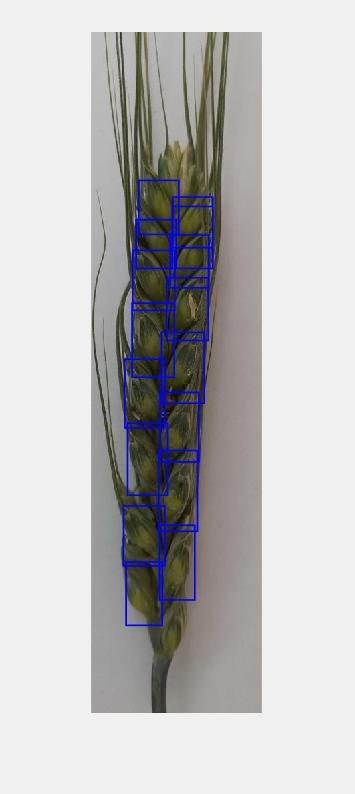

Supplement: Supplementary file 6 [file Data_Sheet_6.ZIP › 7. Detection results/Shannong 25/2020MTL.jpg]

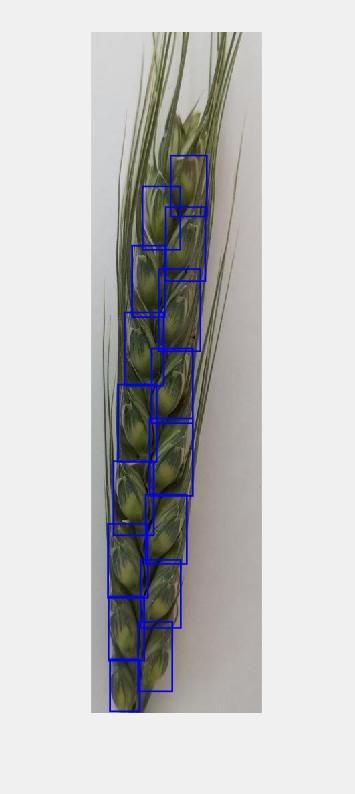

Supplement: Supplementary file 6 [file Data_Sheet_6.ZIP › 7. Detection results/Shannong 25/2022MTL.jpg]

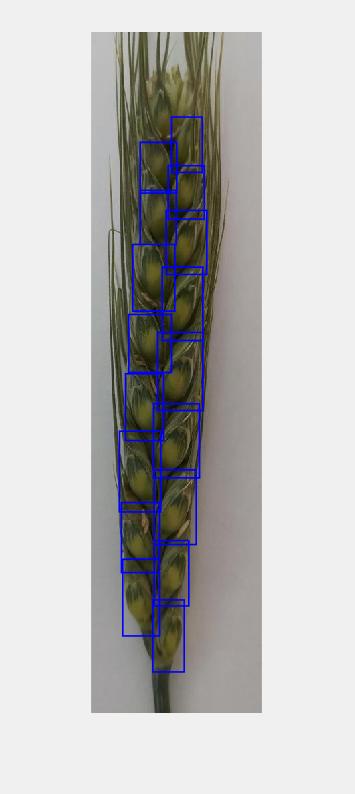

Supplement: Supplementary file 6 [file Data_Sheet_6.ZIP › 7. Detection results/Shannong 25/2024MTL.jpg]

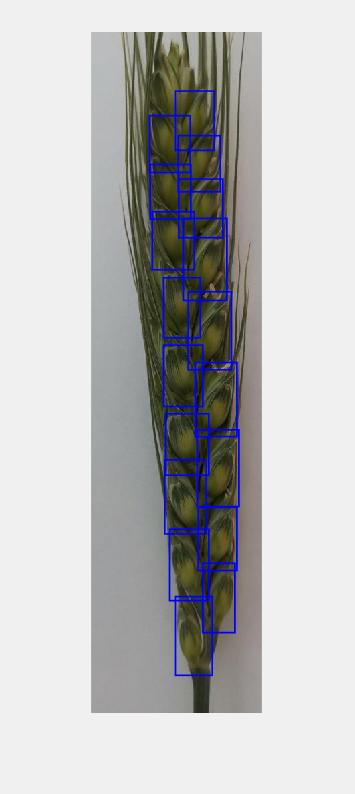

Supplement: Supplementary file 6 [file Data_Sheet_6.ZIP › 7. Detection results/Shannong 25/2025MTL.jpg]

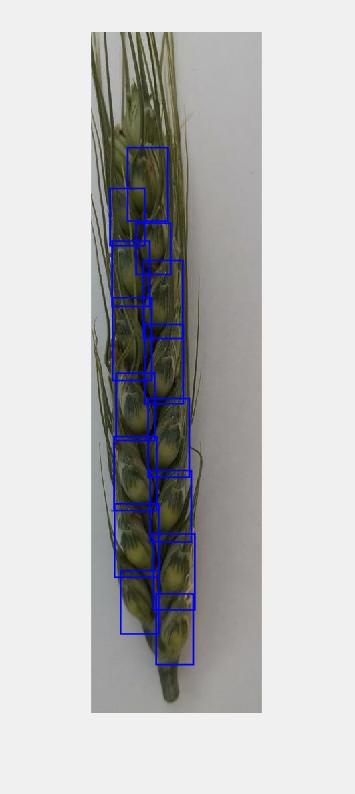

Supplement: Supplementary file 6 [file Data_Sheet_6.ZIP › 7. Detection results/Shannong 25/2028MTL.jpg]

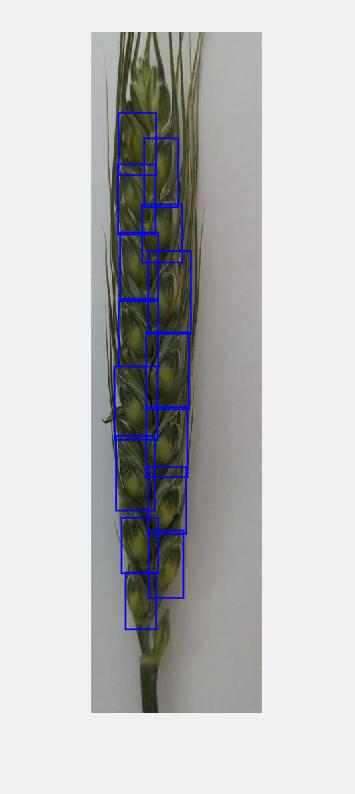

Supplement: Supplementary file 6 [file Data_Sheet_6.ZIP › 7. Detection results/Shannong 25/2030MTL.jpg]

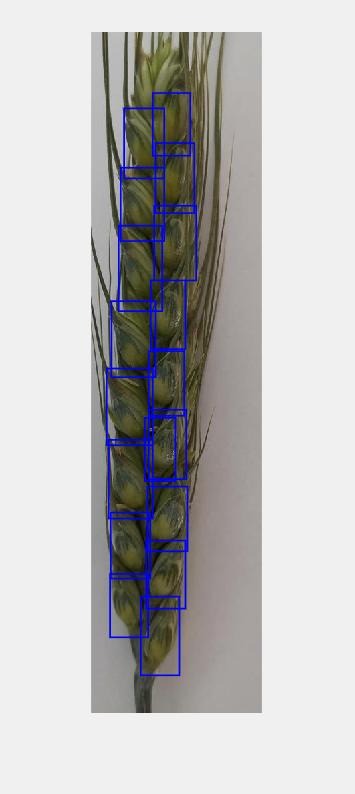

Supplement: Supplementary file 6 [file Data_Sheet_6.ZIP › 7. Detection results/Shannong 25/2031MTL.jpg]

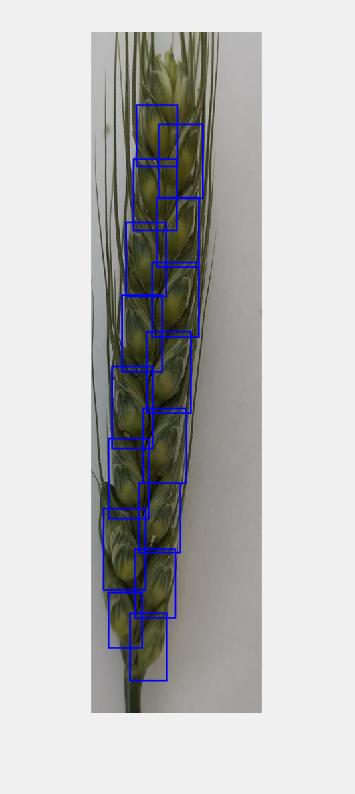

Supplement: Supplementary file 6 [file Data_Sheet_6.ZIP › 7. Detection results/Shannong 25/2033MTL.jpg]

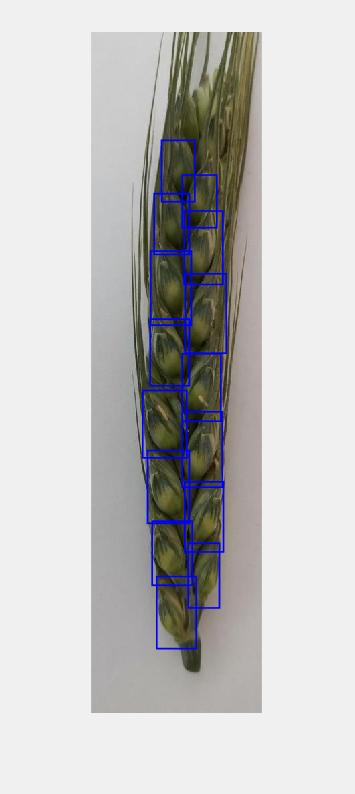

Supplement: Supplementary file 6 [file Data_Sheet_6.ZIP › 7. Detection results/Shannong 25/2035MTL.jpg]

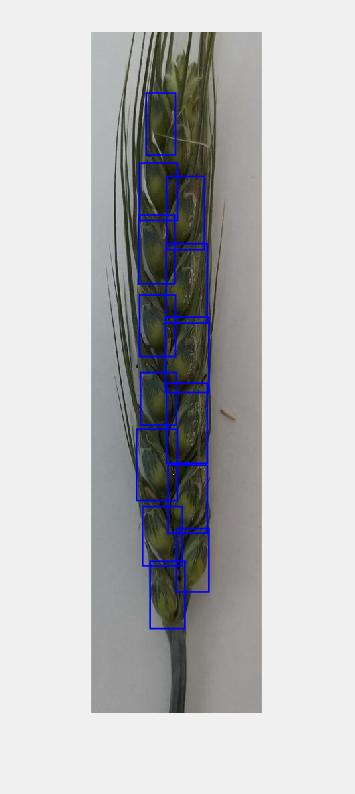

Supplement: Supplementary file 6 [file Data_Sheet_6.ZIP › 7. Detection results/Shannong 25/2037MTL.jpg]

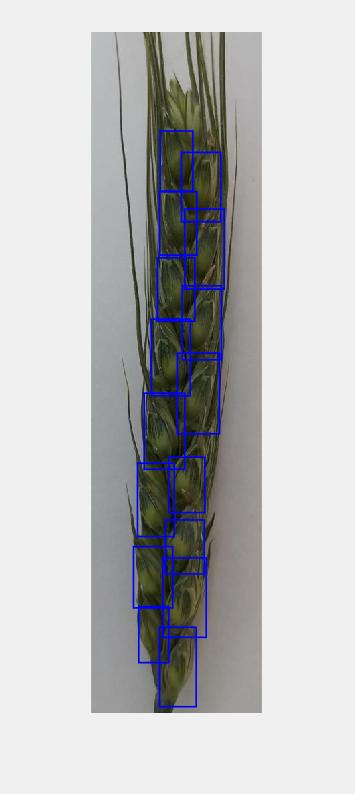

Supplement: Supplementary file 6 [file Data_Sheet_6.ZIP › 7. Detection results/Shannong 25/2038MTL.jpg]

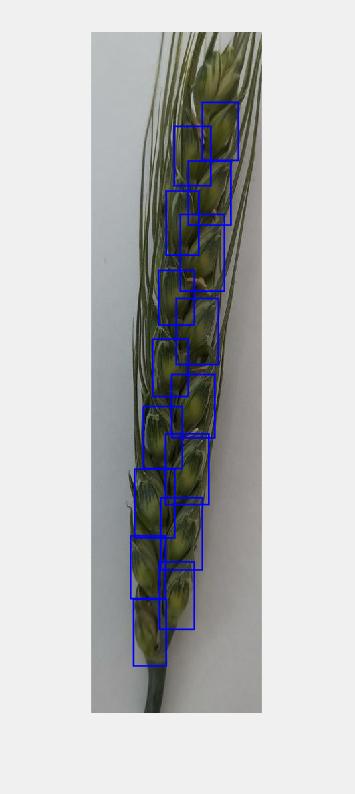

Supplement: Supplementary file 6 [file Data_Sheet_6.ZIP › 7. Detection results/Shannong 25/2039MTL.jpg]

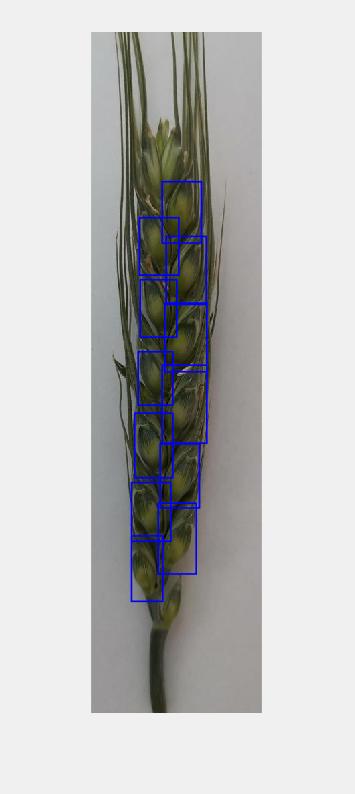

Supplement: Supplementary file 6 [file Data_Sheet_6.ZIP › 7. Detection results/Shannong 25/2041MTL.jpg]

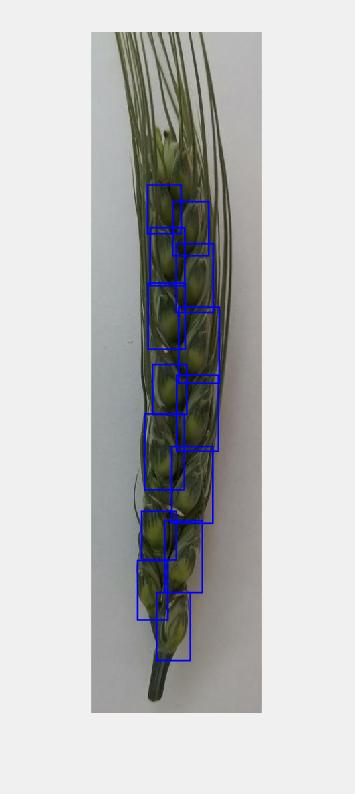

Supplement: Supplementary file 6 [file Data_Sheet_6.ZIP › 7. Detection results/Shannong 25/2042MTL.jpg]

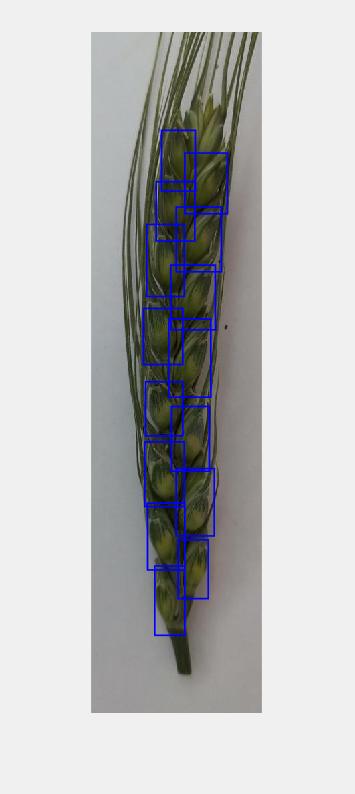

Supplement: Supplementary file 6 [file Data_Sheet_6.ZIP › 7. Detection results/Shannong 25/2043MTL.jpg]

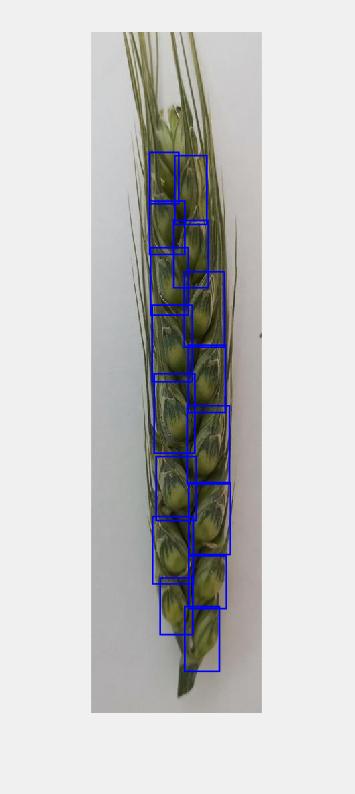

Supplement: Supplementary file 6 [file Data_Sheet_6.ZIP › 7. Detection results/Shannong 25/2044MTL.jpg]

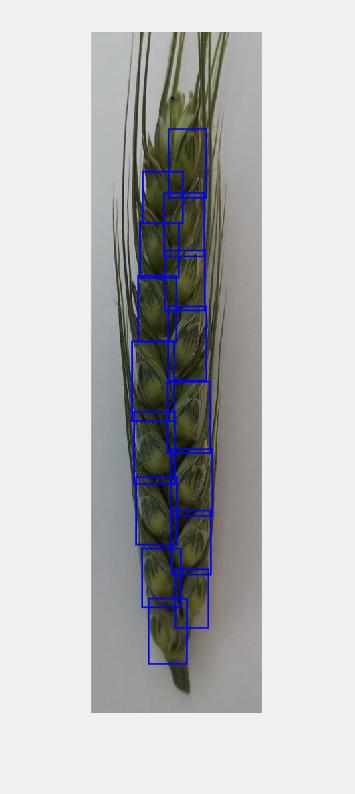

Supplement: Supplementary file 6 [file Data_Sheet_6.ZIP › 7. Detection results/Shannong 25/2045MTL.jpg]

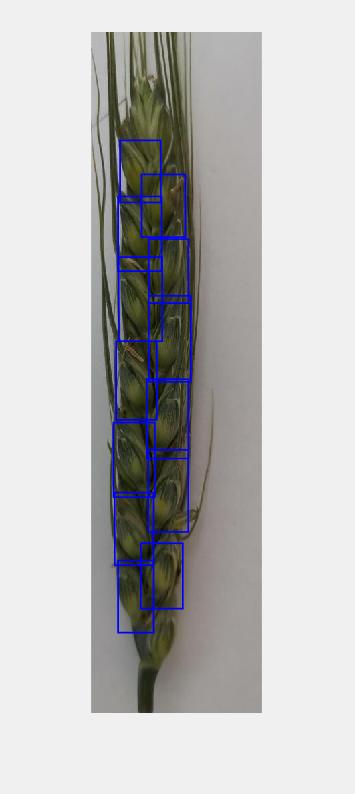

Supplement: Supplementary file 6 [file Data_Sheet_6.ZIP › 7. Detection results/Shannong 25/2048MTL.jpg]

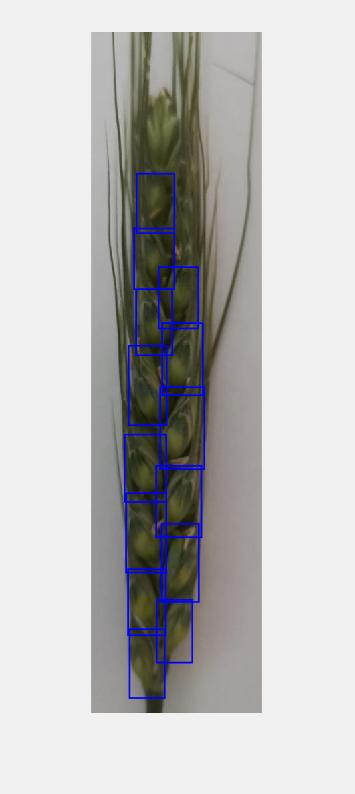

Supplement: Supplementary file 6 [file Data_Sheet_6.ZIP › 7. Detection results/Shannong 25/2049MTL.jpg]

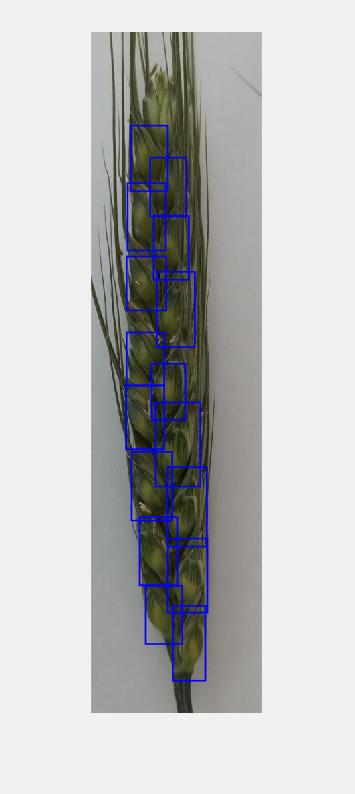

Supplement: Supplementary file 6 [file Data_Sheet_6.ZIP › 7. Detection results/Shannong 25/2050MTL.jpg]

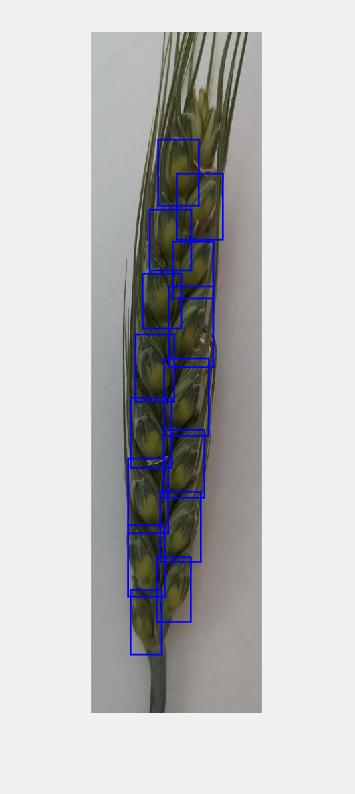

Supplement: Supplementary file 6 [file Data_Sheet_6.ZIP › 7. Detection results/Shannong 25/2055MTL.jpg]

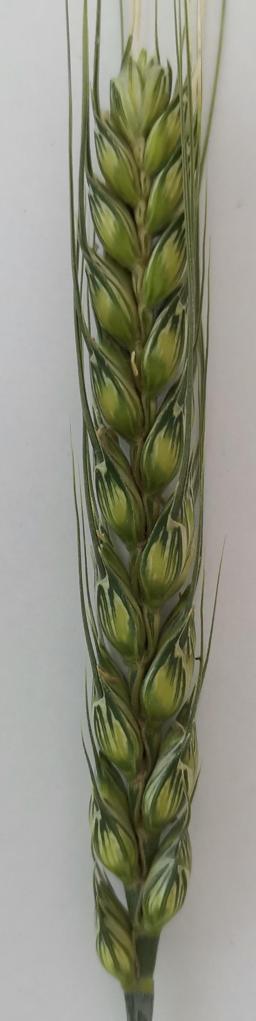

Supplement: Supplementary file 7 [file Data_Sheet_7.ZIP › 8. Detection results (output by DCNN model)/Liangxing 99/3001.jpg]

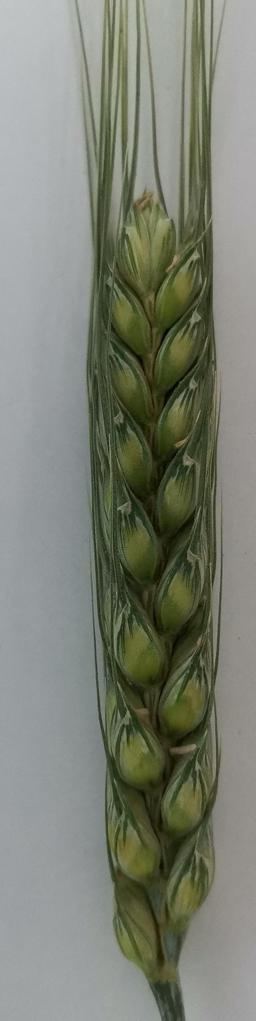

Supplement: Supplementary file 7 [file Data_Sheet_7.ZIP › 8. Detection results (output by DCNN model)/Liangxing 99/3004.jpg]

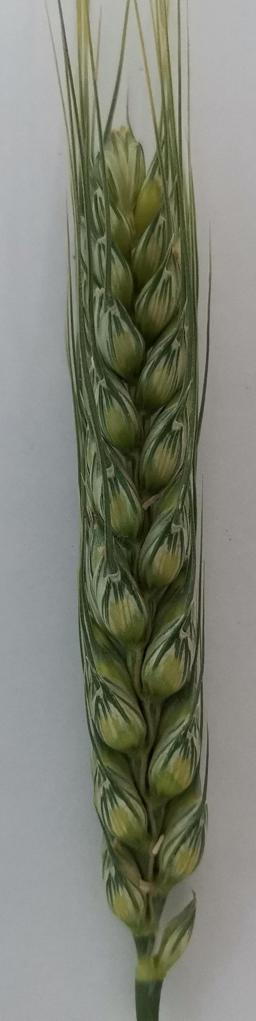

Supplement: Supplementary file 7 [file Data_Sheet_7.ZIP › 8. Detection results (output by DCNN model)/Liangxing 99/3006.jpg]

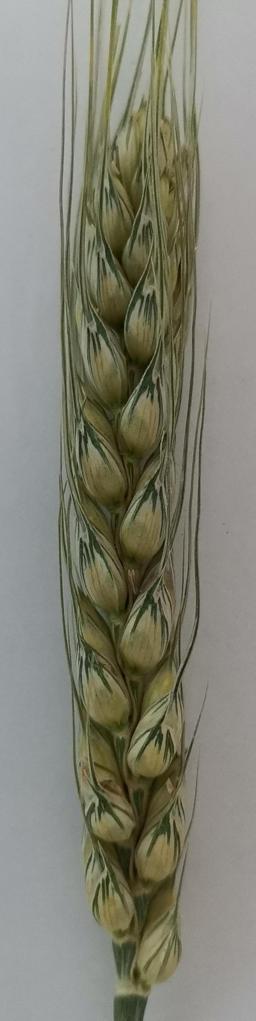

Supplement: Supplementary file 7 [file Data_Sheet_7.ZIP › 8. Detection results (output by DCNN model)/Liangxing 99/3007.jpg]

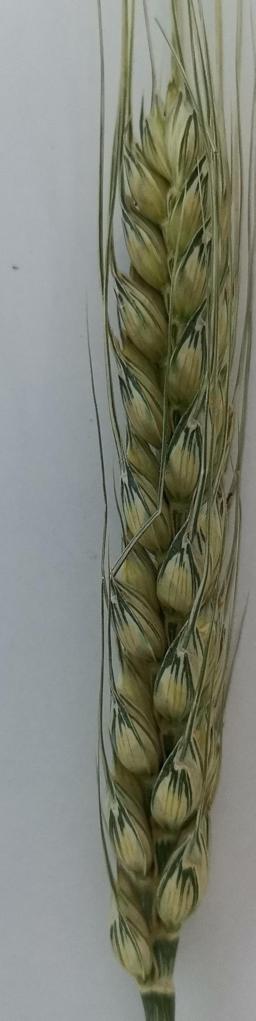

Supplement: Supplementary file 7 [file Data_Sheet_7.ZIP › 8. Detection results (output by DCNN model)/Liangxing 99/3008.jpg]

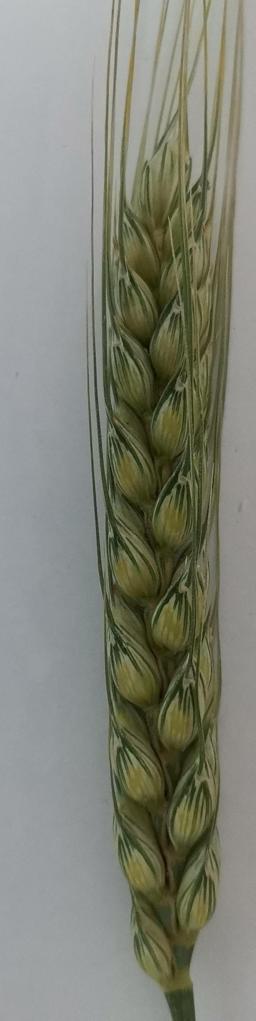

Supplement: Supplementary file 7 [file Data_Sheet_7.ZIP › 8. Detection results (output by DCNN model)/Liangxing 99/3013.jpg]

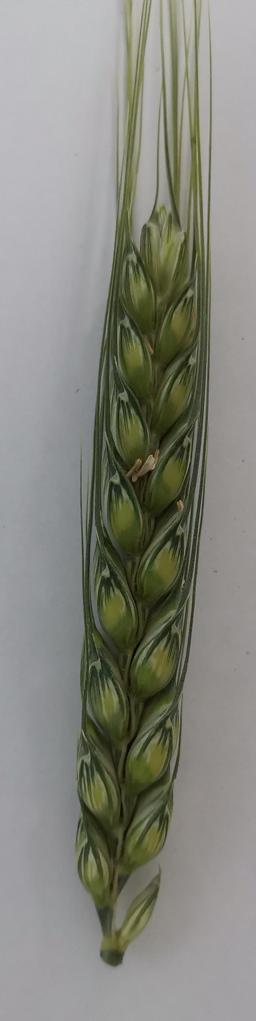

Supplement: Supplementary file 7 [file Data_Sheet_7.ZIP › 8. Detection results (output by DCNN model)/Liangxing 99/3016.jpg]

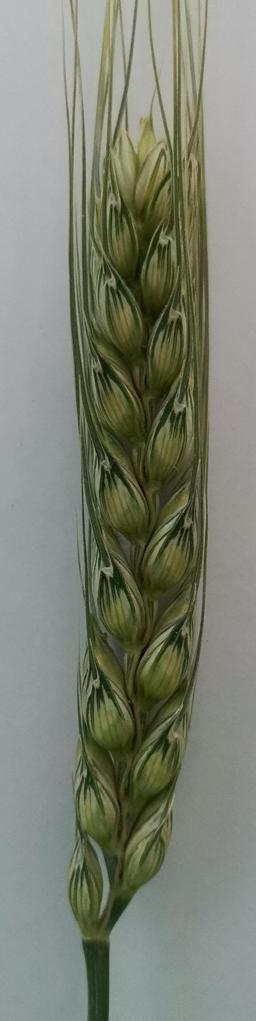

Supplement: Supplementary file 7 [file Data_Sheet_7.ZIP › 8. Detection results (output by DCNN model)/Liangxing 99/3019.jpg]

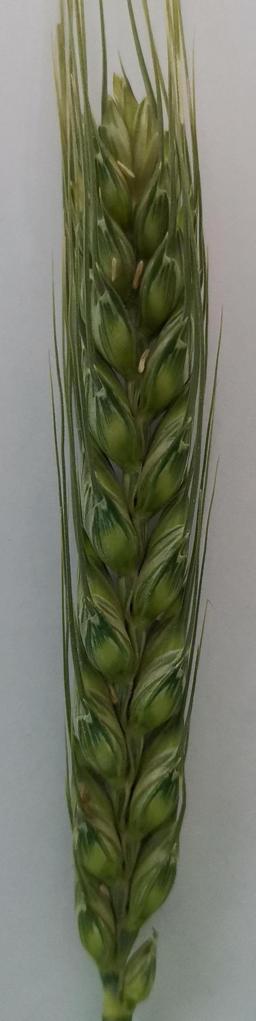

Supplement: Supplementary file 7 [file Data_Sheet_7.ZIP › 8. Detection results (output by DCNN model)/Liangxing 99/3024.jpg]

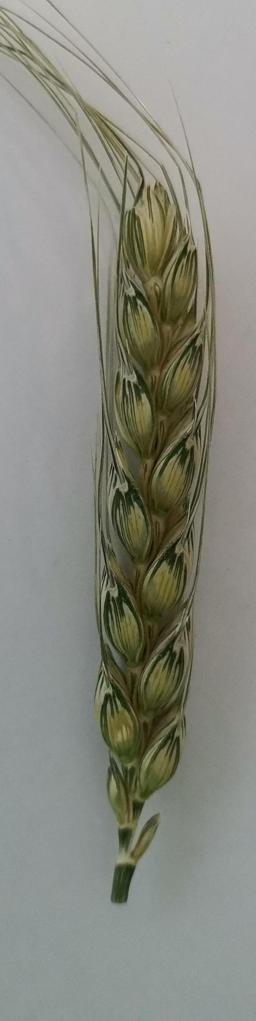

Supplement: Supplementary file 7 [file Data_Sheet_7.ZIP › 8. Detection results (output by DCNN model)/Liangxing 99/3025.jpg]

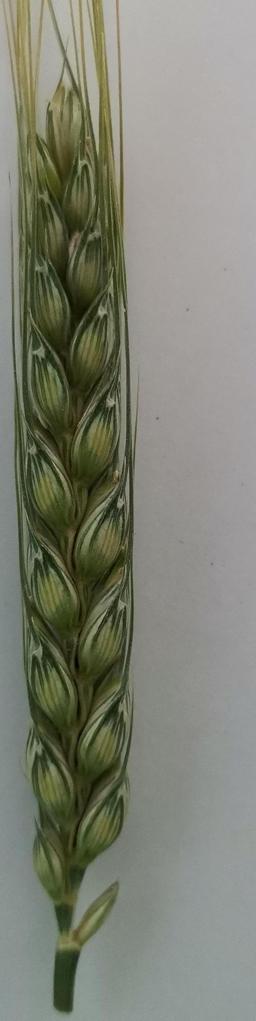

Supplement: Supplementary file 7 [file Data_Sheet_7.ZIP › 8. Detection results (output by DCNN model)/Liangxing 99/3029.jpg]

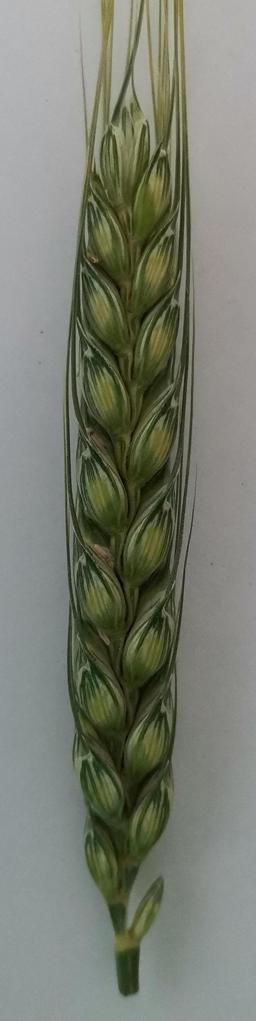

Supplement: Supplementary file 7 [file Data_Sheet_7.ZIP › 8. Detection results (output by DCNN model)/Liangxing 99/3032.jpg]

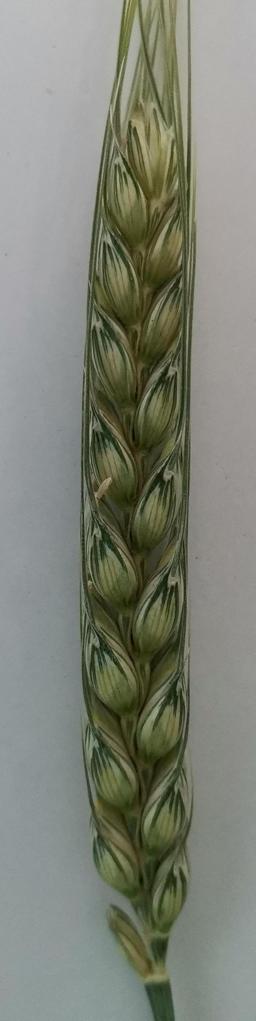

Supplement: Supplementary file 7 [file Data_Sheet_7.ZIP › 8. Detection results (output by DCNN model)/Liangxing 99/3035.jpg]

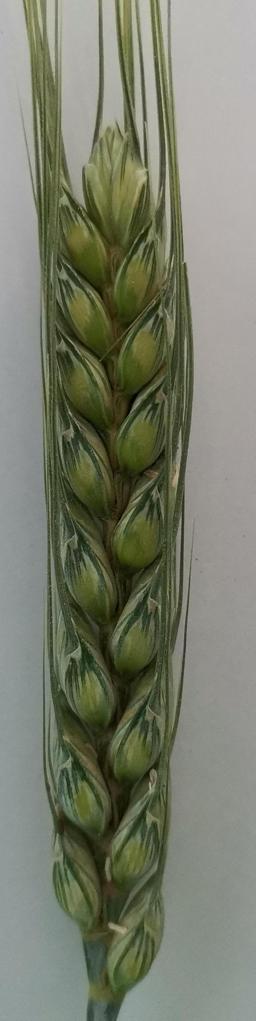

Supplement: Supplementary file 7 [file Data_Sheet_7.ZIP › 8. Detection results (output by DCNN model)/Liangxing 99/3038.jpg]

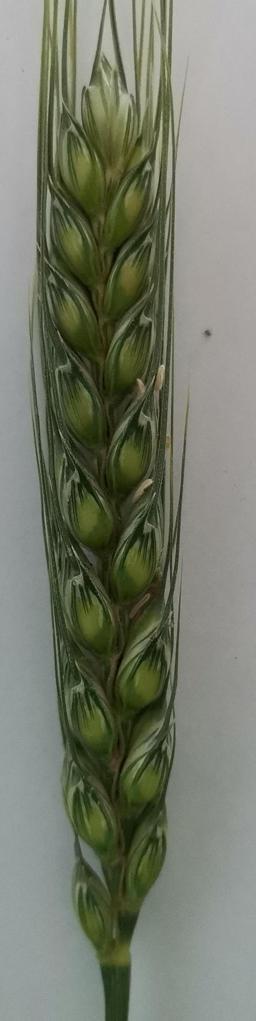

Supplement: Supplementary file 7 [file Data_Sheet_7.ZIP › 8. Detection results (output by DCNN model)/Liangxing 99/3044.jpg]

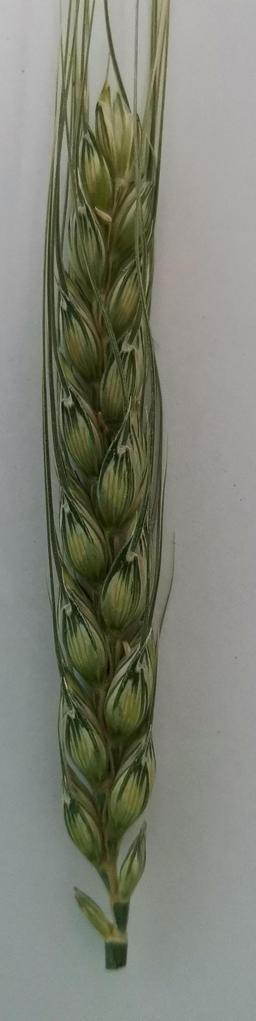

Supplement: Supplementary file 7 [file Data_Sheet_7.ZIP › 8. Detection results (output by DCNN model)/Liangxing 99/3045.jpg]

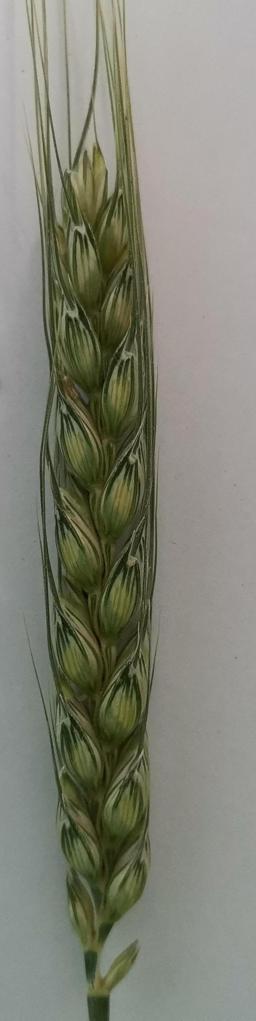

Supplement: Supplementary file 7 [file Data_Sheet_7.ZIP › 8. Detection results (output by DCNN model)/Liangxing 99/3046.jpg]

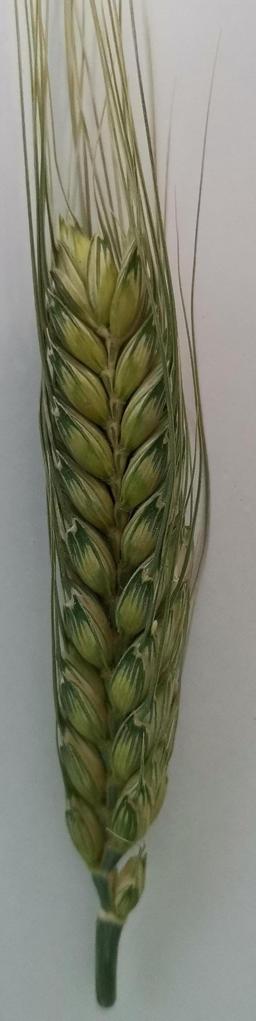

Supplement: Supplementary file 7 [file Data_Sheet_7.ZIP › 8. Detection results (output by DCNN model)/Liangxing 99/3047.jpg]

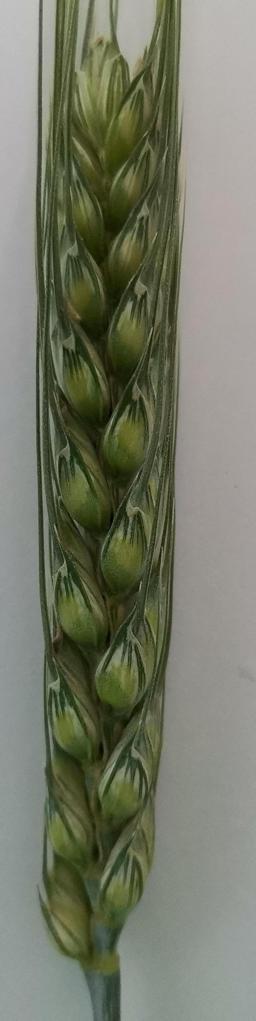

Supplement: Supplementary file 7 [file Data_Sheet_7.ZIP › 8. Detection results (output by DCNN model)/Liangxing 99/3048.jpg]

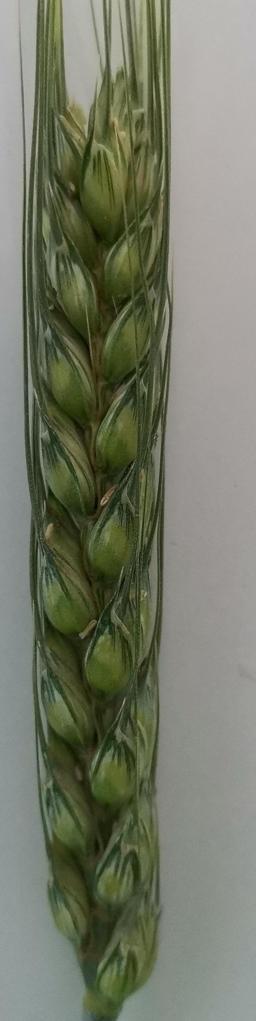

Supplement: Supplementary file 7 [file Data_Sheet_7.ZIP › 8. Detection results (output by DCNN model)/Liangxing 99/3049.jpg]

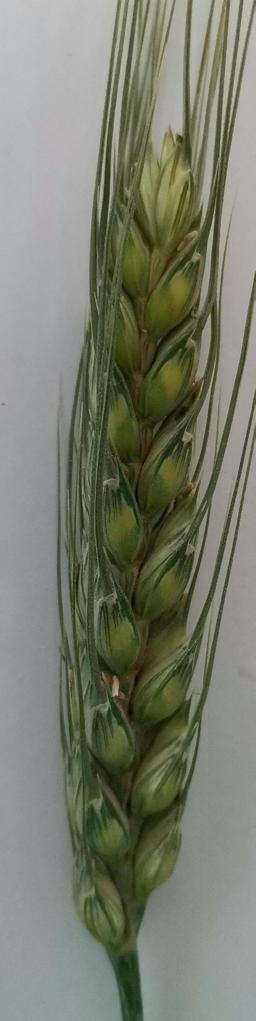

Supplement: Supplementary file 7 [file Data_Sheet_7.ZIP › 8. Detection results (output by DCNN model)/Liangxing 99/3050.jpg]

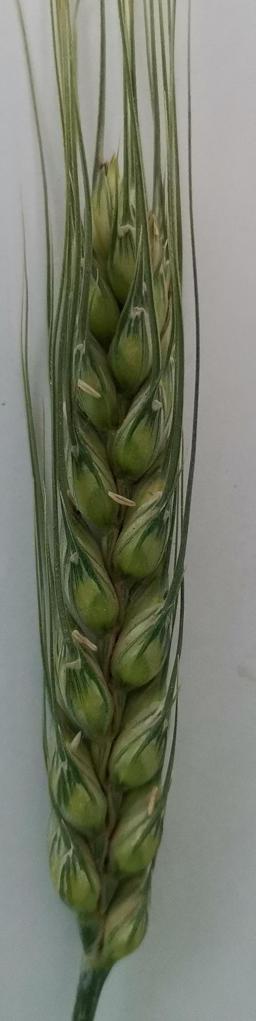

Supplement: Supplementary file 7 [file Data_Sheet_7.ZIP › 8. Detection results (output by DCNN model)/Liangxing 99/3051.jpg]

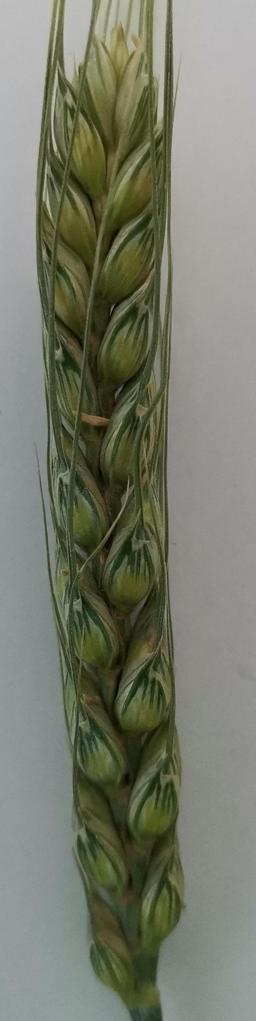

Supplement: Supplementary file 7 [file Data_Sheet_7.ZIP › 8. Detection results (output by DCNN model)/Liangxing 99/3053.jpg]

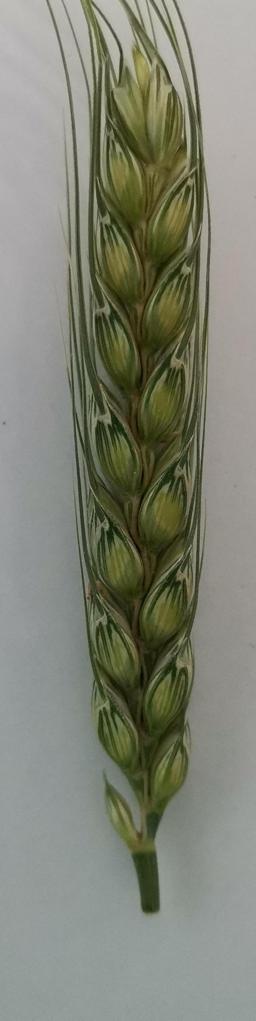

Supplement: Supplementary file 7 [file Data_Sheet_7.ZIP › 8. Detection results (output by DCNN model)/Liangxing 99/3054.jpg]

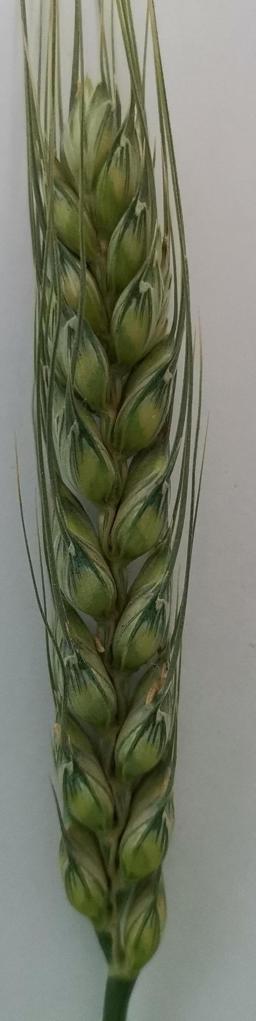

Supplement: Supplementary file 7 [file Data_Sheet_7.ZIP › 8. Detection results (output by DCNN model)/Liangxing 99/3056.jpg]

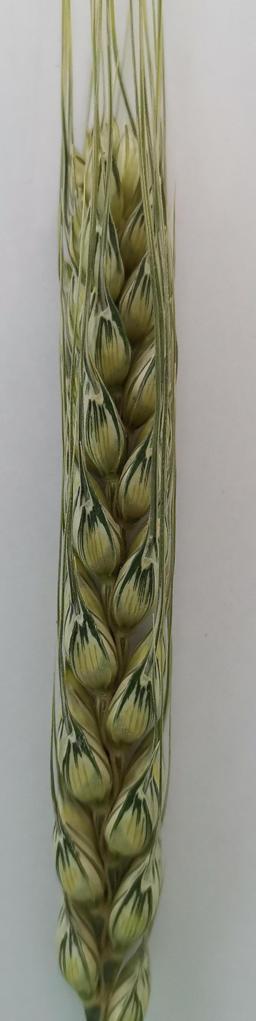

Supplement: Supplementary file 7 [file Data_Sheet_7.ZIP › 8. Detection results (output by DCNN model)/Liangxing 99/3062.jpg]

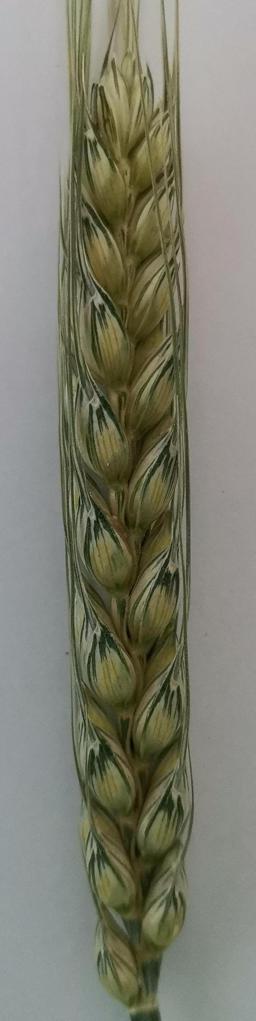

Supplement: Supplementary file 7 [file Data_Sheet_7.ZIP › 8. Detection results (output by DCNN model)/Liangxing 99/3063.jpg]

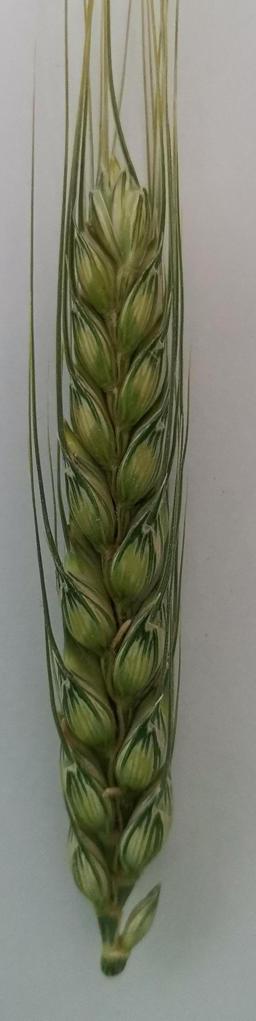

Supplement: Supplementary file 7 [file Data_Sheet_7.ZIP › 8. Detection results (output by DCNN model)/Liangxing 99/3065.jpg]

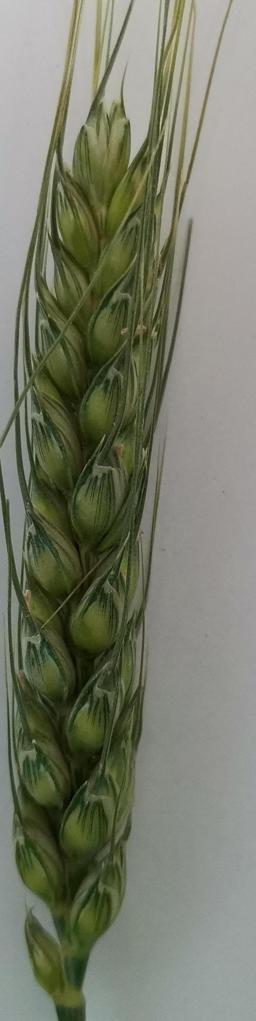

Supplement: Supplementary file 7 [file Data_Sheet_7.ZIP › 8. Detection results (output by DCNN model)/Liangxing 99/3066.jpg]

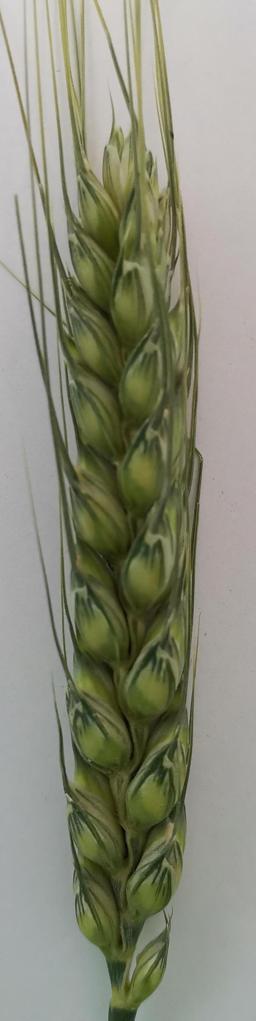

Supplement: Supplementary file 7 [file Data_Sheet_7.ZIP › 8. Detection results (output by DCNN model)/Liangxing 99/3067.jpg]

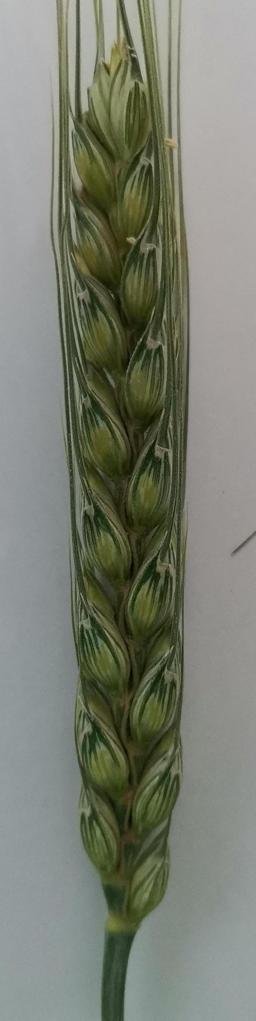

Supplement: Supplementary file 7 [file Data_Sheet_7.ZIP › 8. Detection results (output by DCNN model)/Liangxing 99/3068.jpg]

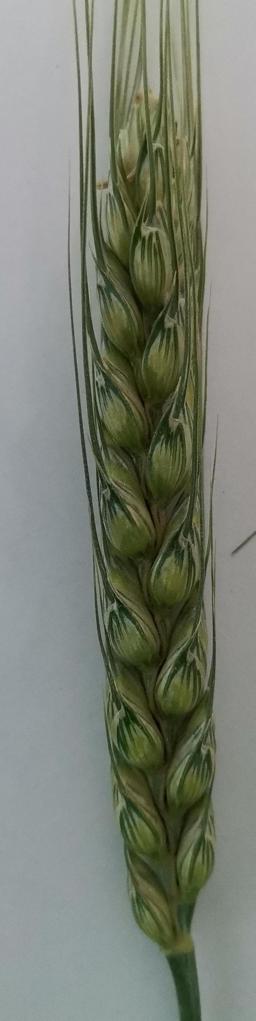

Supplement: Supplementary file 7 [file Data_Sheet_7.ZIP › 8. Detection results (output by DCNN model)/Liangxing 99/3069.jpg]

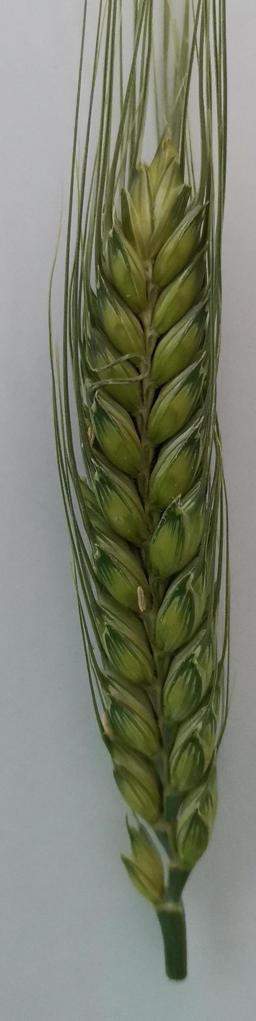

Supplement: Supplementary file 7 [file Data_Sheet_7.ZIP › 8. Detection results (output by DCNN model)/Liangxing 99/3074.jpg]

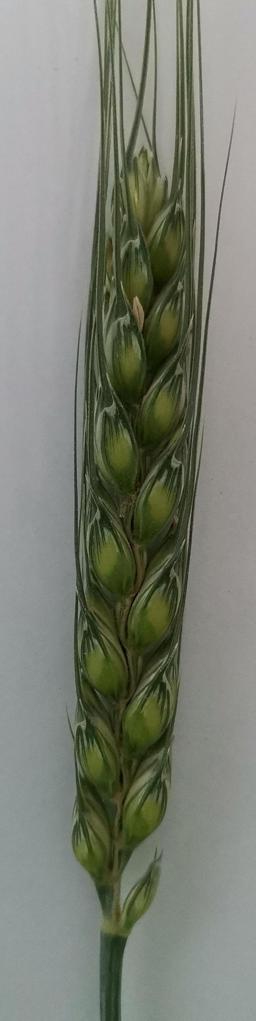

Supplement: Supplementary file 7 [file Data_Sheet_7.ZIP › 8. Detection results (output by DCNN model)/Liangxing 99/3079.jpg]

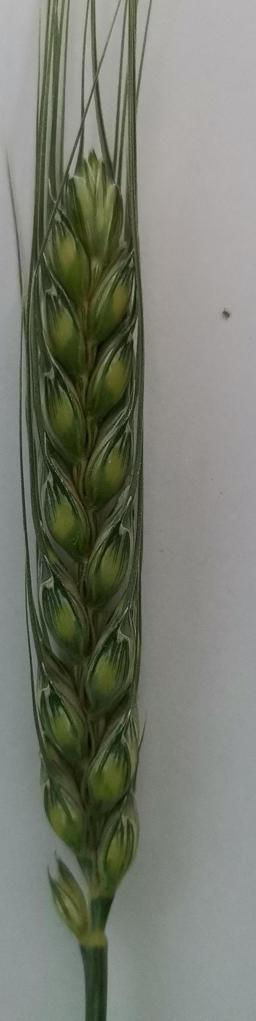

Supplement: Supplementary file 7 [file Data_Sheet_7.ZIP › 8. Detection results (output by DCNN model)/Liangxing 99/3080.jpg]

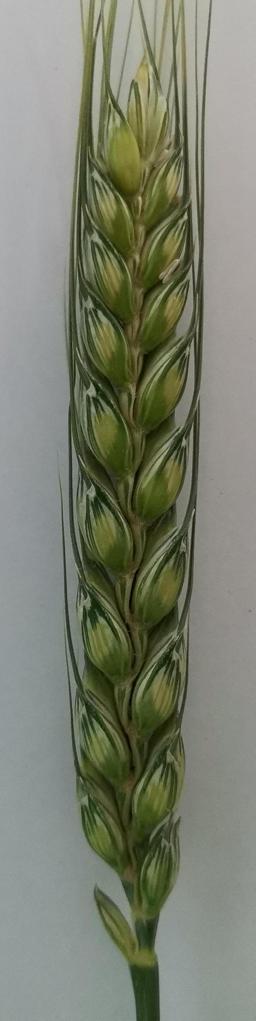

Supplement: Supplementary file 7 [file Data_Sheet_7.ZIP › 8. Detection results (output by DCNN model)/Liangxing 99/3084.jpg]

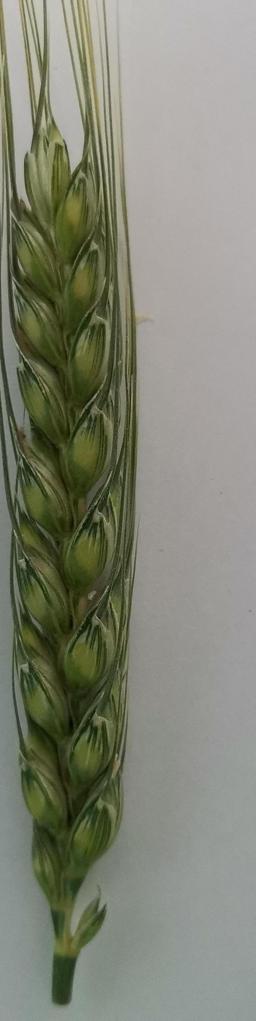

Supplement: Supplementary file 7 [file Data_Sheet_7.ZIP › 8. Detection results (output by DCNN model)/Liangxing 99/3085.jpg]

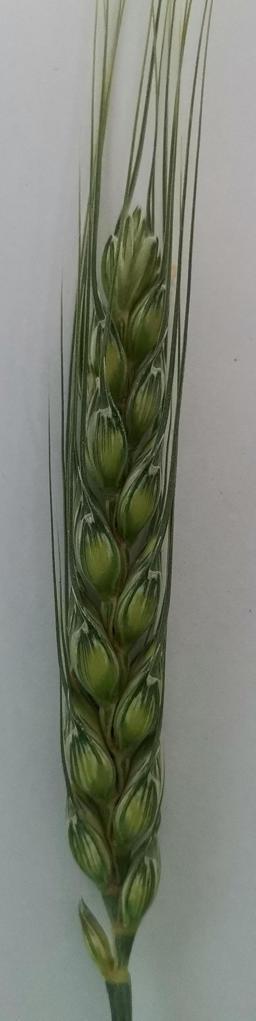

Supplement: Supplementary file 7 [file Data_Sheet_7.ZIP › 8. Detection results (output by DCNN model)/Liangxing 99/3089.jpg]

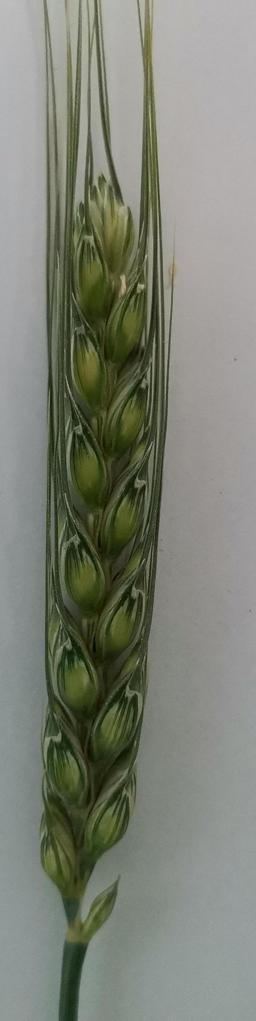

Supplement: Supplementary file 7 [file Data_Sheet_7.ZIP › 8. Detection results (output by DCNN model)/Liangxing 99/3090.jpg]

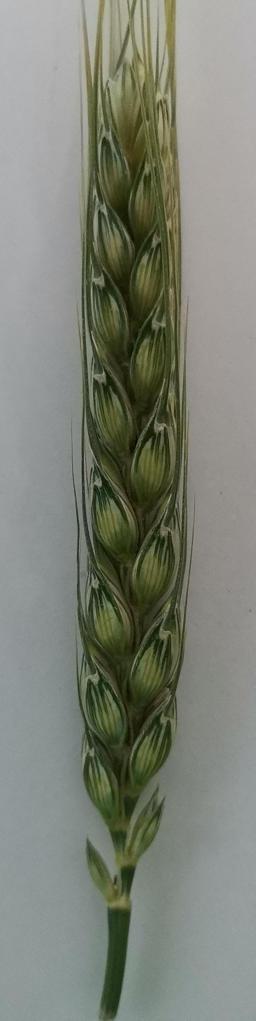

Supplement: Supplementary file 7 [file Data_Sheet_7.ZIP › 8. Detection results (output by DCNN model)/Liangxing 99/3091.jpg]

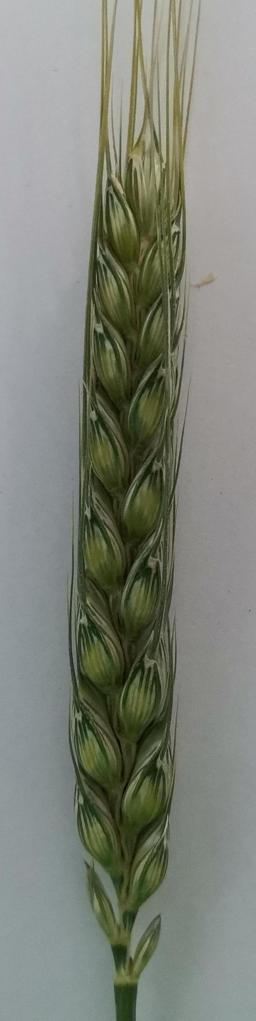

Supplement: Supplementary file 7 [file Data_Sheet_7.ZIP › 8. Detection results (output by DCNN model)/Liangxing 99/3092.jpg]

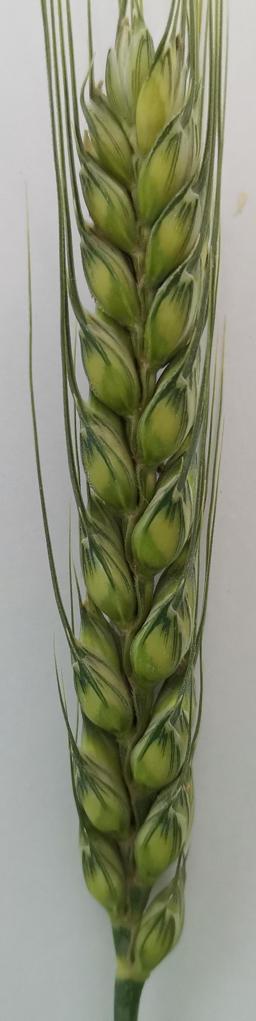

Supplement: Supplementary file 7 [file Data_Sheet_7.ZIP › 8. Detection results (output by DCNN model)/Liangxing 99/3093.jpg]

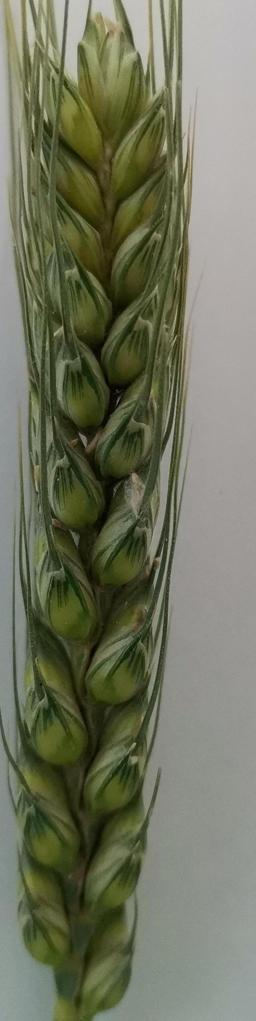

Supplement: Supplementary file 7 [file Data_Sheet_7.ZIP › 8. Detection results (output by DCNN model)/Liangxing 99/3095.jpg]

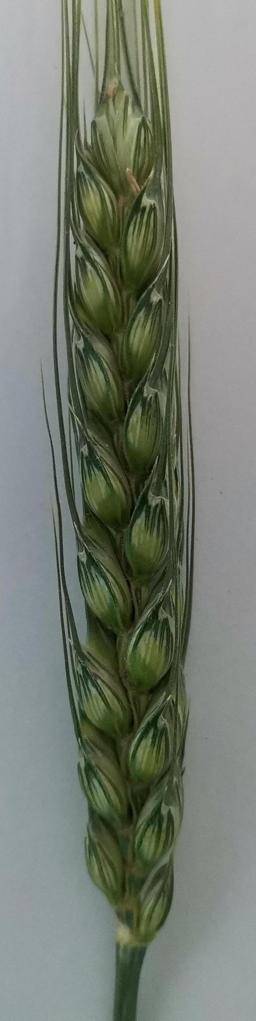

Supplement: Supplementary file 7 [file Data_Sheet_7.ZIP › 8. Detection results (output by DCNN model)/Liangxing 99/3098.jpg]

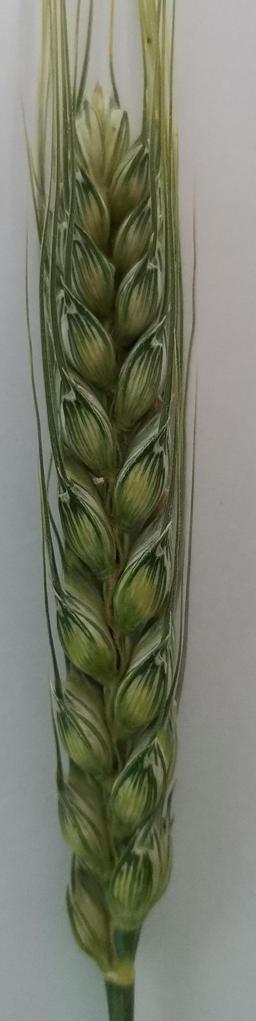

Supplement: Supplementary file 7 [file Data_Sheet_7.ZIP › 8. Detection results (output by DCNN model)/Liangxing 99/3103.jpg]

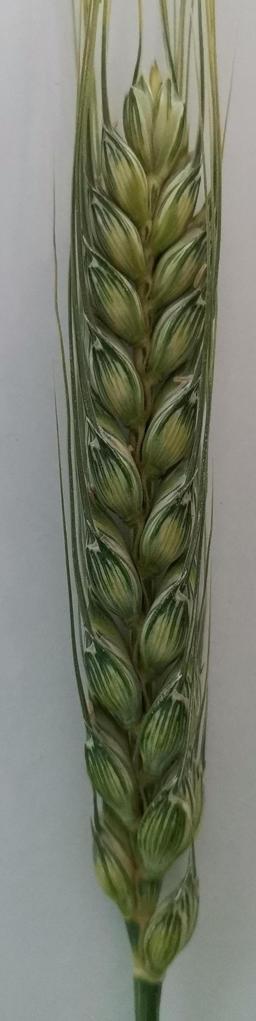

Supplement: Supplementary file 7 [file Data_Sheet_7.ZIP › 8. Detection results (output by DCNN model)/Liangxing 99/3104.jpg]

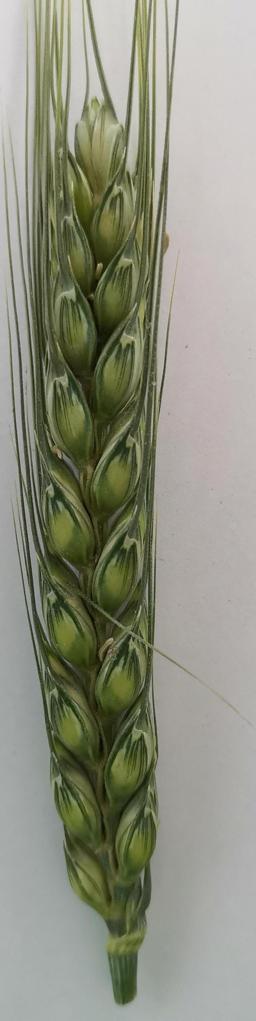

Supplement: Supplementary file 7 [file Data_Sheet_7.ZIP › 8. Detection results (output by DCNN model)/Liangxing 99/3107.jpg]

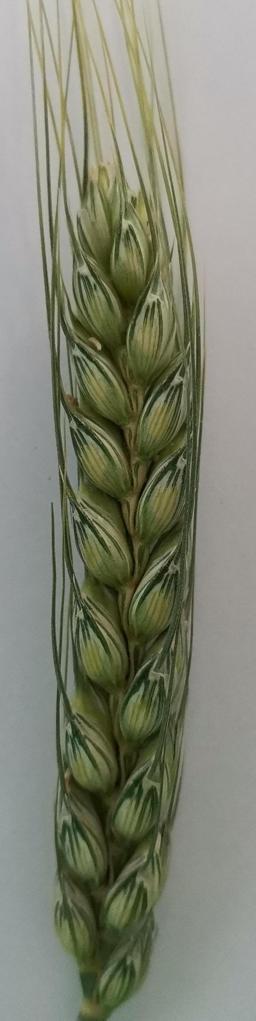

Supplement: Supplementary file 7 [file Data_Sheet_7.ZIP › 8. Detection results (output by DCNN model)/Liangxing 99/3109.jpg]

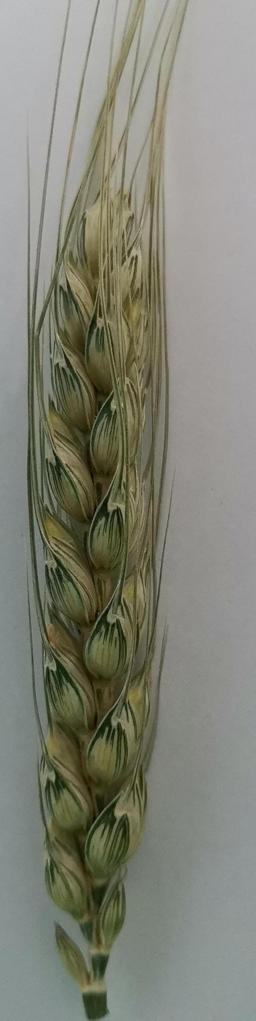

Supplement: Supplementary file 7 [file Data_Sheet_7.ZIP › 8. Detection results (output by DCNN model)/Liangxing 99/3112.jpg]

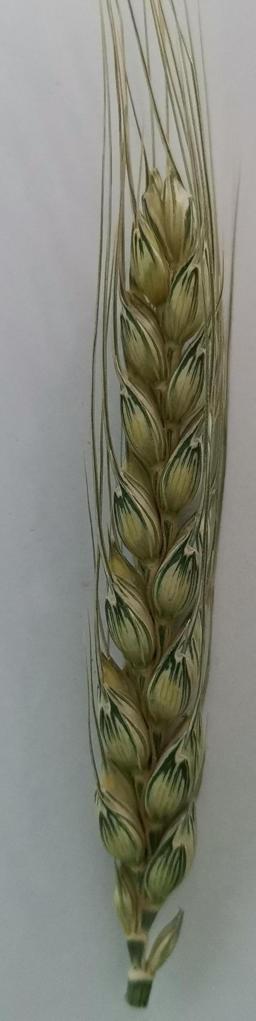

Supplement: Supplementary file 7 [file Data_Sheet_7.ZIP › 8. Detection results (output by DCNN model)/Liangxing 99/3113.jpg]

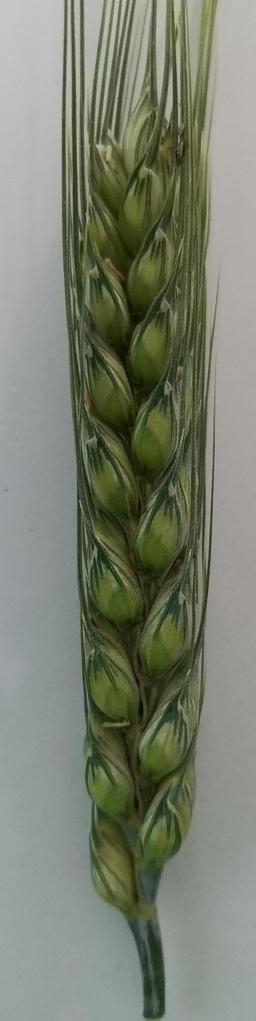

Supplement: Supplementary file 7 [file Data_Sheet_7.ZIP › 8. Detection results (output by DCNN model)/Liangxing 99/3114.jpg]

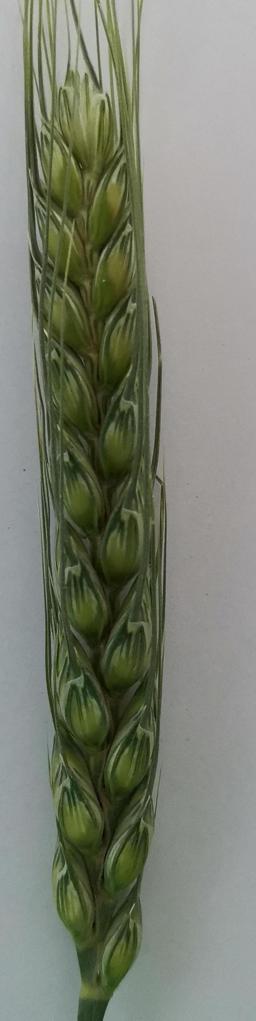

Supplement: Supplementary file 7 [file Data_Sheet_7.ZIP › 8. Detection results (output by DCNN model)/Liangxing 99/3117.jpg]

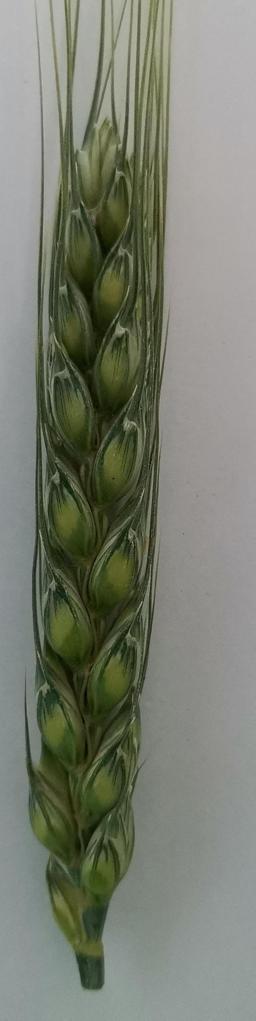

Supplement: Supplementary file 7 [file Data_Sheet_7.ZIP › 8. Detection results (output by DCNN model)/Liangxing 99/3119.jpg]

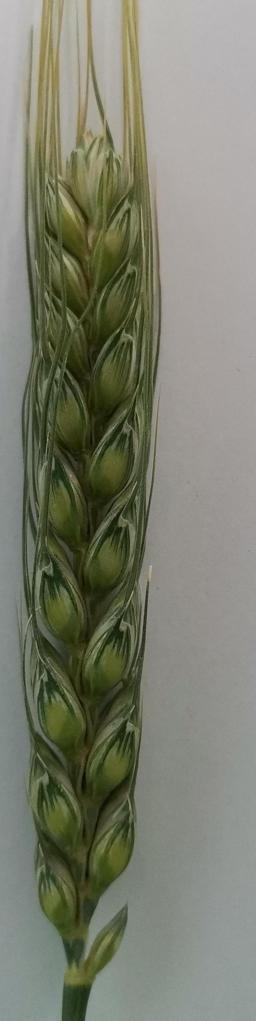

Supplement: Supplementary file 7 [file Data_Sheet_7.ZIP › 8. Detection results (output by DCNN model)/Liangxing 99/3121.jpg]

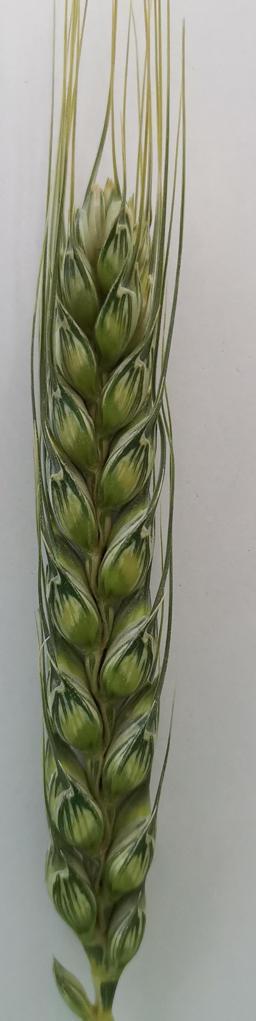

Supplement: Supplementary file 7 [file Data_Sheet_7.ZIP › 8. Detection results (output by DCNN model)/Liangxing 99/3122.jpg]

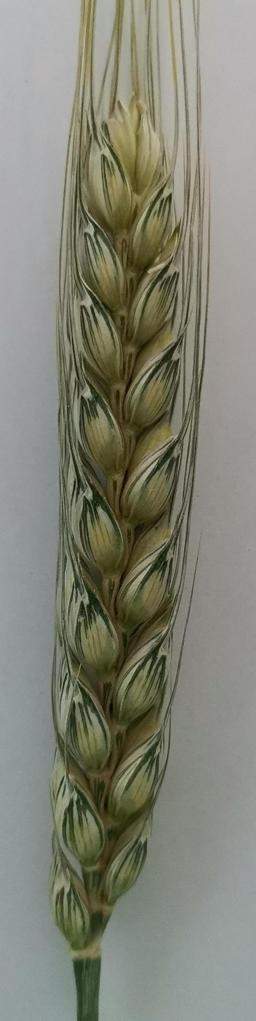

Supplement: Supplementary file 7 [file Data_Sheet_7.ZIP › 8. Detection results (output by DCNN model)/Liangxing 99/3123.jpg]

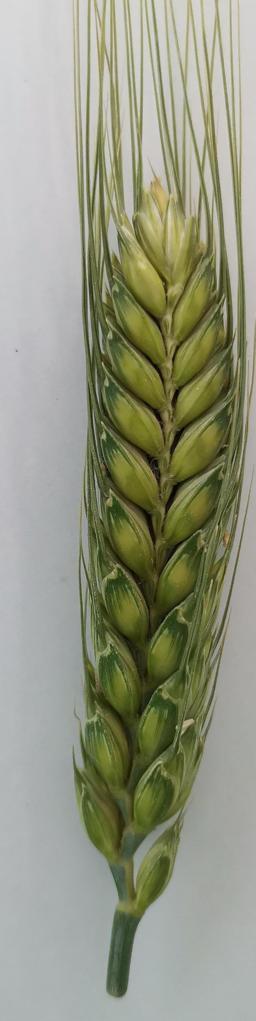

Supplement: Supplementary file 7 [file Data_Sheet_7.ZIP › 8. Detection results (output by DCNN model)/Liangxing 99/3125.jpg]

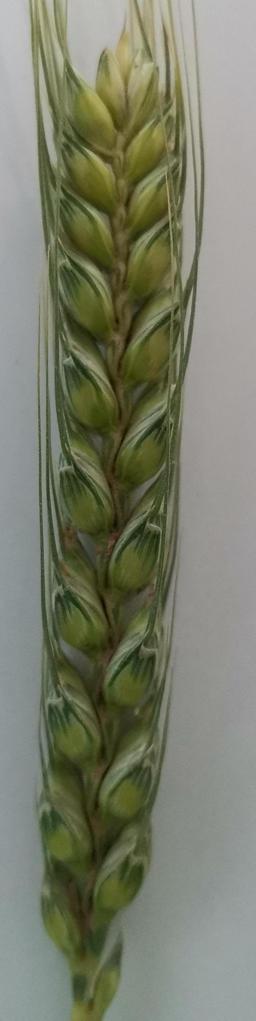

Supplement: Supplementary file 7 [file Data_Sheet_7.ZIP › 8. Detection results (output by DCNN model)/Liangxing 99/3126.jpg]

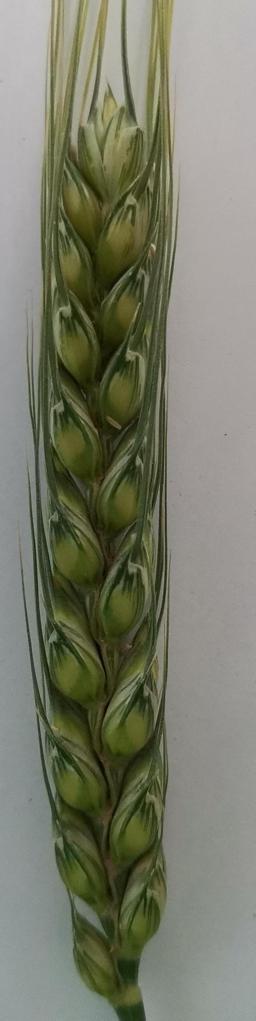

Supplement: Supplementary file 7 [file Data_Sheet_7.ZIP › 8. Detection results (output by DCNN model)/Liangxing 99/3130.jpg]

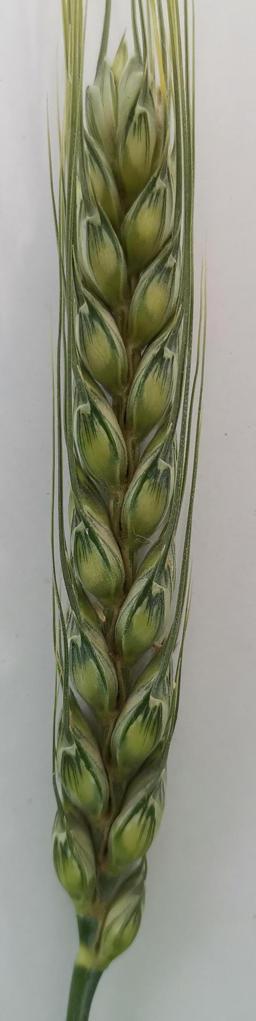

Supplement: Supplementary file 7 [file Data_Sheet_7.ZIP › 8. Detection results (output by DCNN model)/Liangxing 99/3131.jpg]

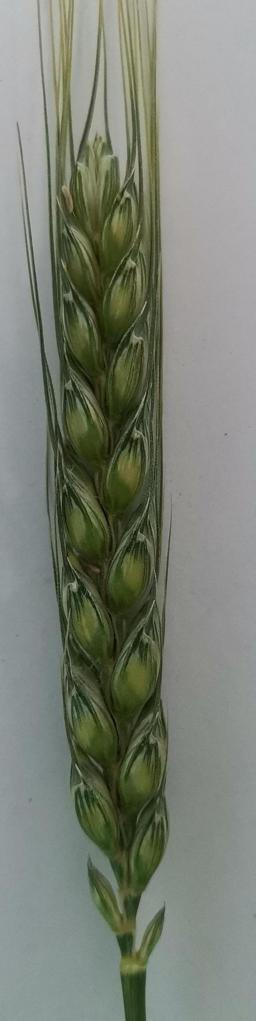

Supplement: Supplementary file 7 [file Data_Sheet_7.ZIP › 8. Detection results (output by DCNN model)/Liangxing 99/3132.jpg]

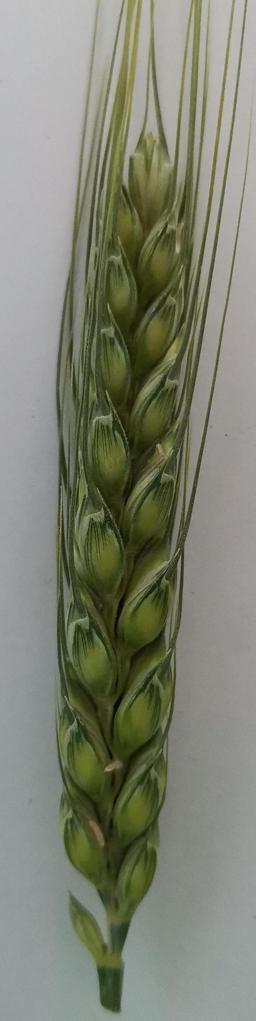

Supplement: Supplementary file 7 [file Data_Sheet_7.ZIP › 8. Detection results (output by DCNN model)/Liangxing 99/3135.jpg]

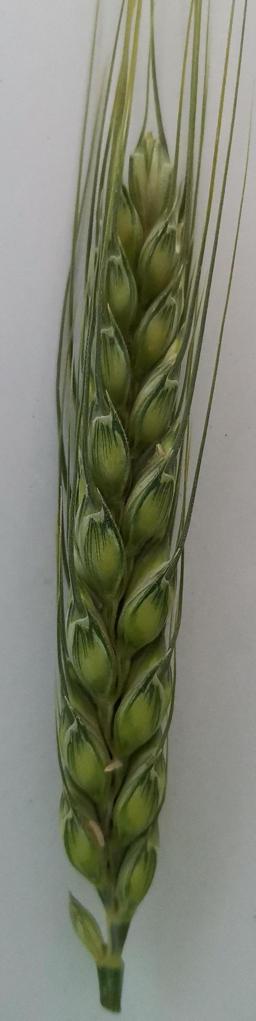

Supplement: Supplementary file 7 [file Data_Sheet_7.ZIP › 8. Detection results (output by DCNN model)/Liangxing 99/3136.jpg]

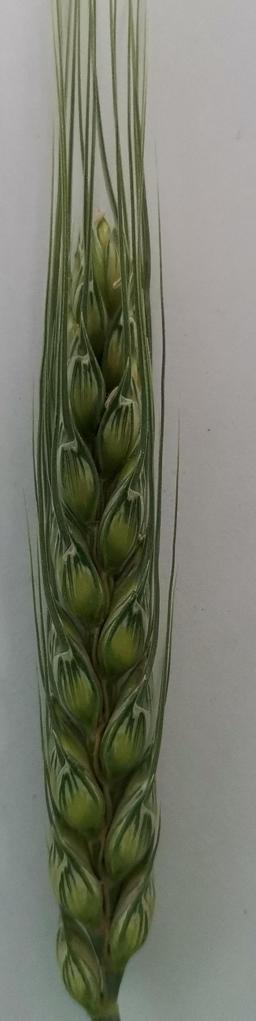

Supplement: Supplementary file 7 [file Data_Sheet_7.ZIP › 8. Detection results (output by DCNN model)/Liangxing 99/3137.jpg]

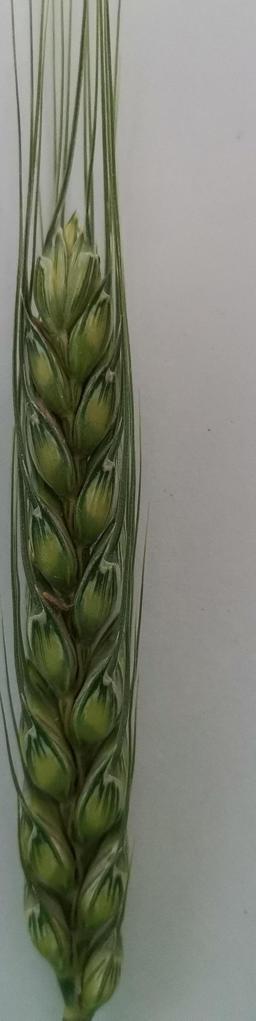

Supplement: Supplementary file 7 [file Data_Sheet_7.ZIP › 8. Detection results (output by DCNN model)/Liangxing 99/3138.jpg]

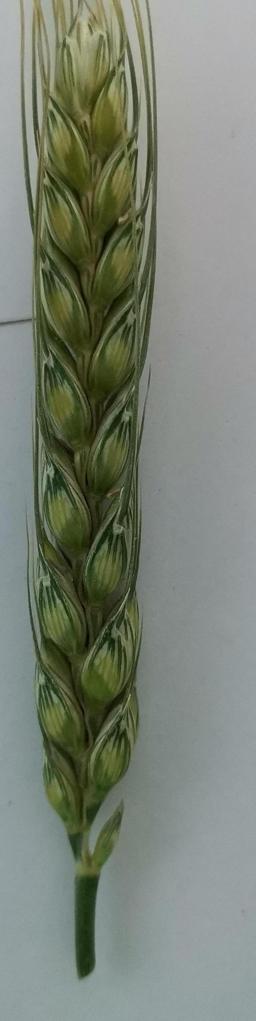

Supplement: Supplementary file 7 [file Data_Sheet_7.ZIP › 8. Detection results (output by DCNN model)/Liangxing 99/3139.jpg]

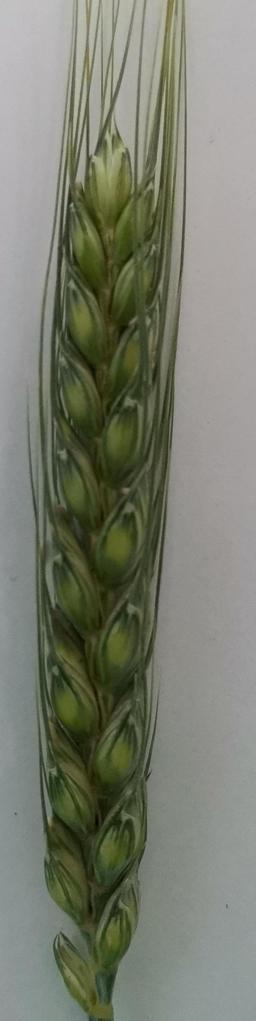

Supplement: Supplementary file 7 [file Data_Sheet_7.ZIP › 8. Detection results (output by DCNN model)/Liangxing 99/3141.jpg]
