# Supplementary figures and images for: Automatic Detection and Counting of Wheat Spikelet Using Semi-Automatic Labeling and Deep Learning (part 7 of 8)
Source: Front Plant Sci. 2022 May 30;13:872555. doi: 10.3389/fpls.2022.872555 (PMC9189412; doi:10.3389/fpls.2022.872555)

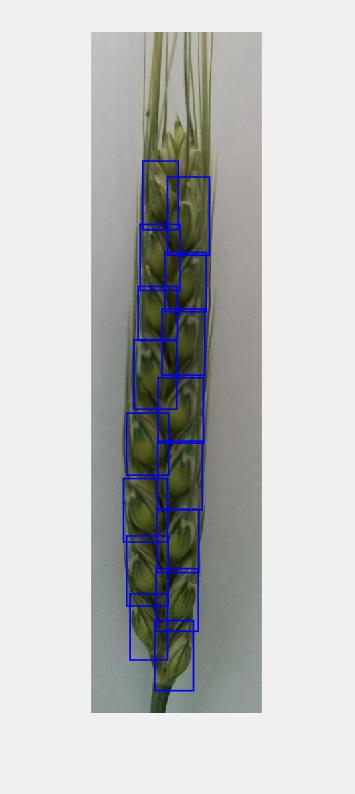

Supplement: Supplementary file 6 [file Data_Sheet_6.ZIP › 7. Detection results/Liangxing 99/3146MTL.jpg]

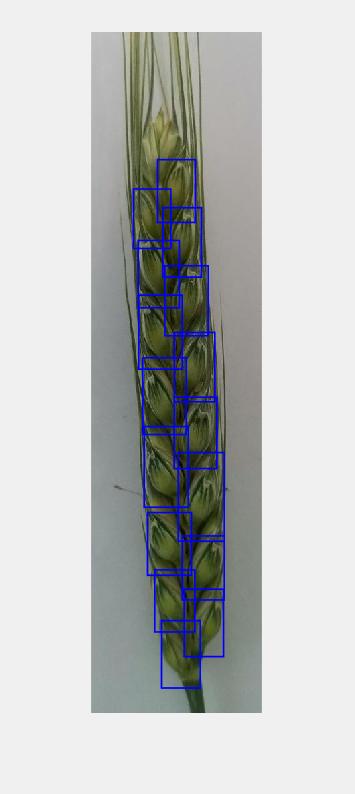

Supplement: Supplementary file 6 [file Data_Sheet_6.ZIP › 7. Detection results/Liangxing 99/3147MTL.jpg]

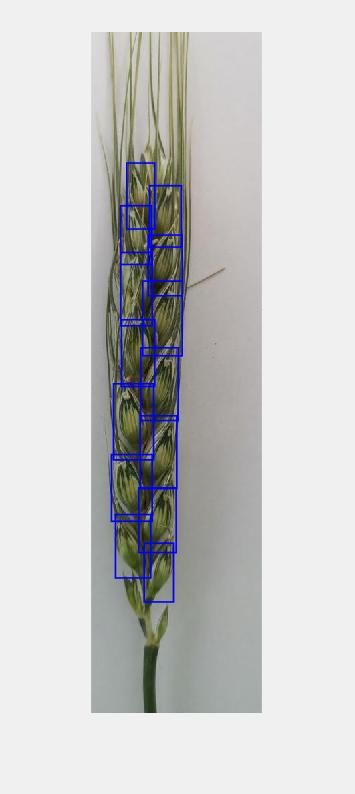

Supplement: Supplementary file 6 [file Data_Sheet_6.ZIP › 7. Detection results/Liangxing 99/3148MTL.jpg]

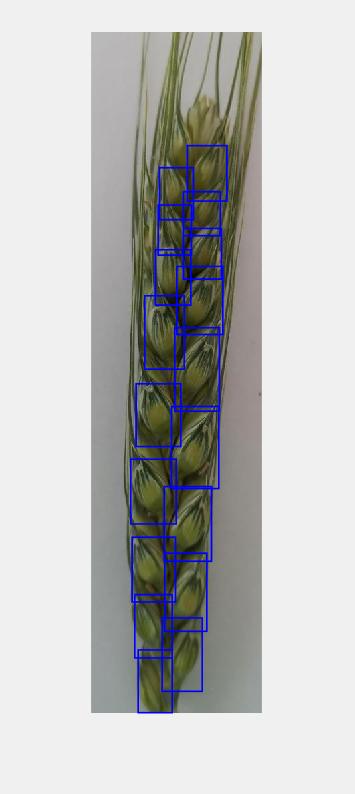

Supplement: Supplementary file 6 [file Data_Sheet_6.ZIP › 7. Detection results/Liangxing 99/3154MTL.jpg]

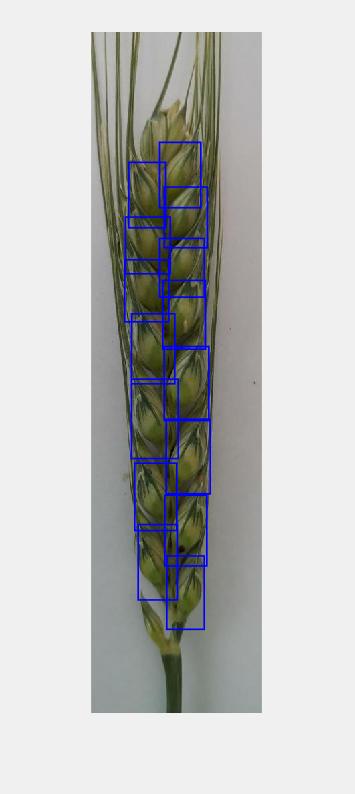

Supplement: Supplementary file 6 [file Data_Sheet_6.ZIP › 7. Detection results/Liangxing 99/3155MTL.jpg]

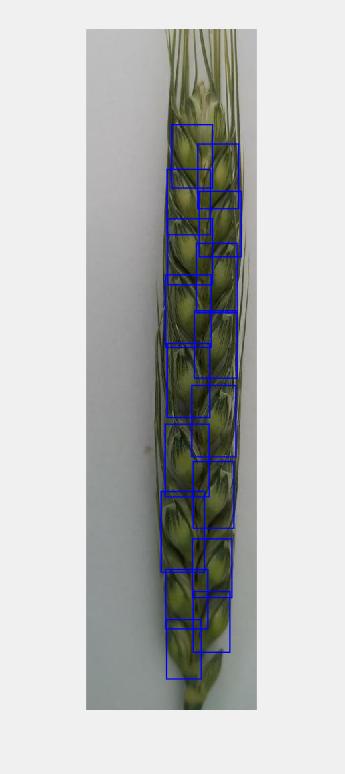

Supplement: Supplementary file 6 [file Data_Sheet_6.ZIP › 7. Detection results/Liangxing 99/3161MTL.jpg]

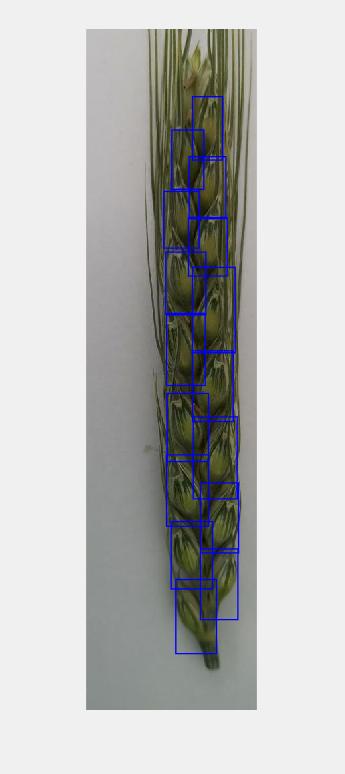

Supplement: Supplementary file 6 [file Data_Sheet_6.ZIP › 7. Detection results/Liangxing 99/3163MTL.jpg]

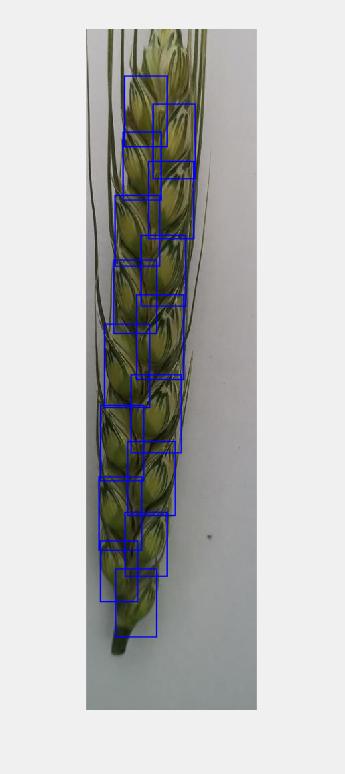

Supplement: Supplementary file 6 [file Data_Sheet_6.ZIP › 7. Detection results/Liangxing 99/3164MTL.jpg]

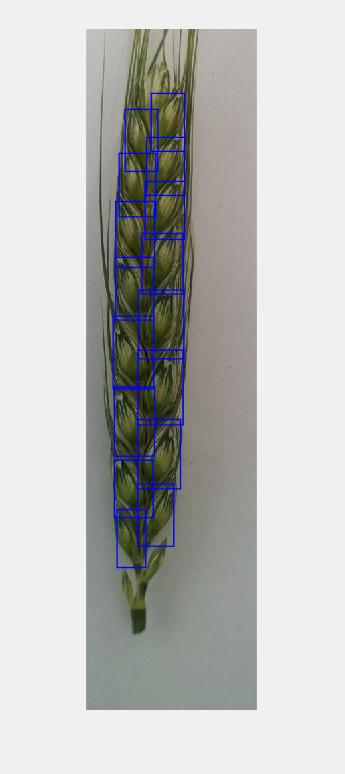

Supplement: Supplementary file 6 [file Data_Sheet_6.ZIP › 7. Detection results/Liangxing 99/3165MTL.jpg]

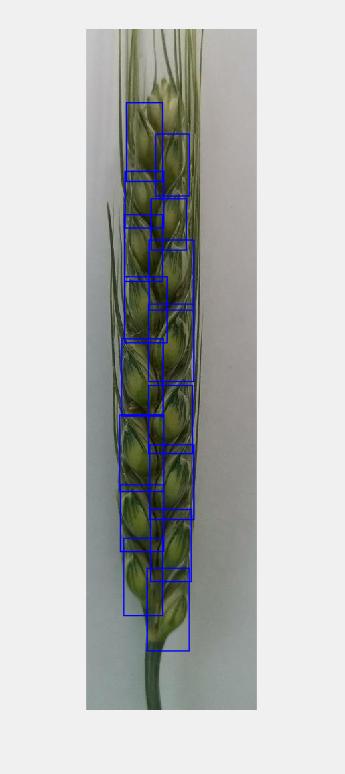

Supplement: Supplementary file 6 [file Data_Sheet_6.ZIP › 7. Detection results/Liangxing 99/3167MTL.jpg]

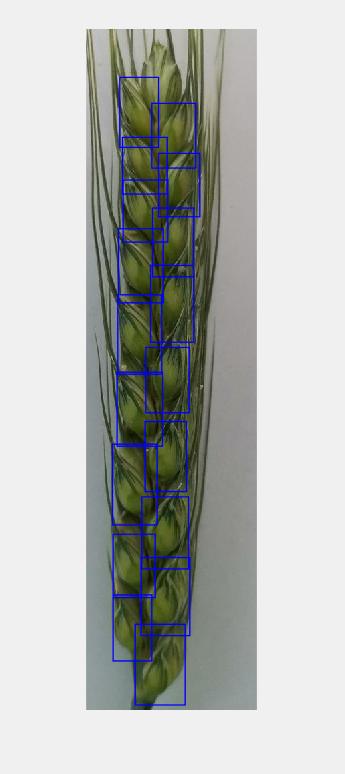

Supplement: Supplementary file 6 [file Data_Sheet_6.ZIP › 7. Detection results/Liangxing 99/3172MTL.jpg]

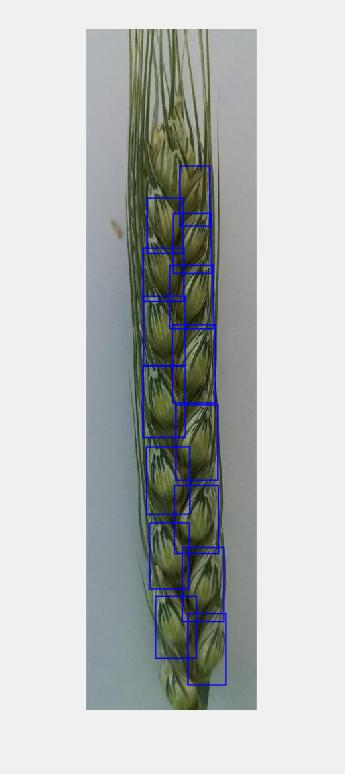

Supplement: Supplementary file 6 [file Data_Sheet_6.ZIP › 7. Detection results/Liangxing 99/3178MTL.jpg]

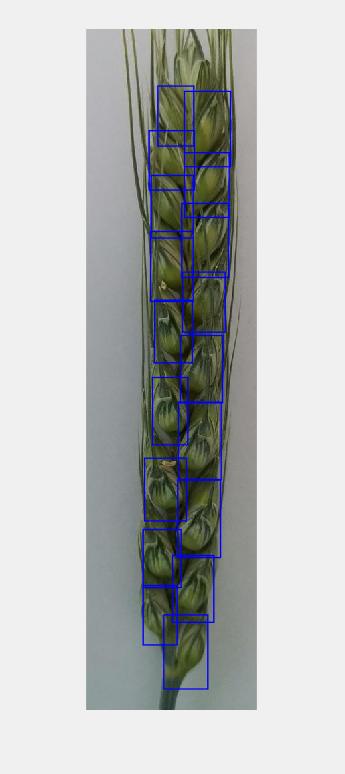

Supplement: Supplementary file 6 [file Data_Sheet_6.ZIP › 7. Detection results/Liangxing 99/3180MTL.jpg]

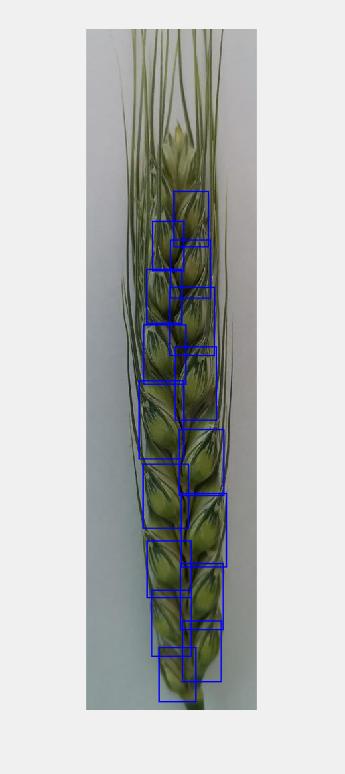

Supplement: Supplementary file 6 [file Data_Sheet_6.ZIP › 7. Detection results/Liangxing 99/3183MTL.jpg]

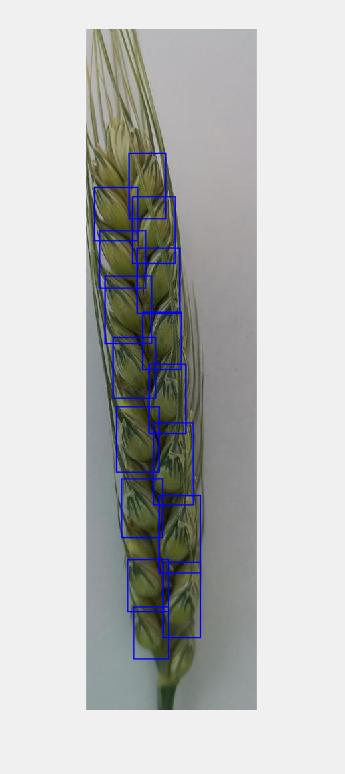

Supplement: Supplementary file 6 [file Data_Sheet_6.ZIP › 7. Detection results/Liangxing 99/3186MTL.jpg]

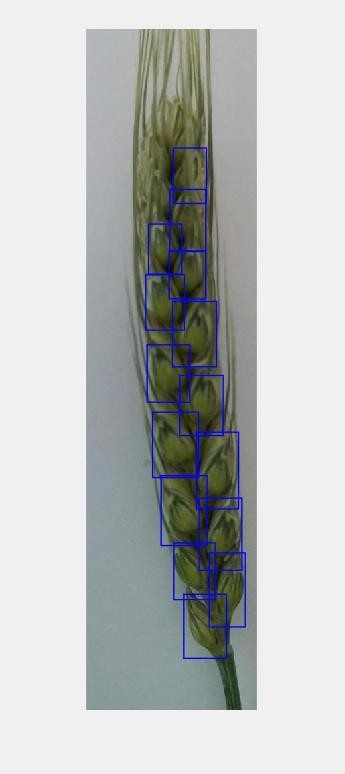

Supplement: Supplementary file 6 [file Data_Sheet_6.ZIP › 7. Detection results/Liangxing 99/3187MTL.jpg]

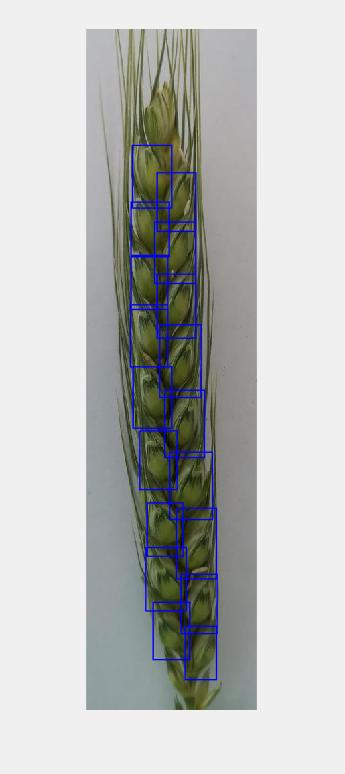

Supplement: Supplementary file 6 [file Data_Sheet_6.ZIP › 7. Detection results/Liangxing 99/3191MTL.jpg]

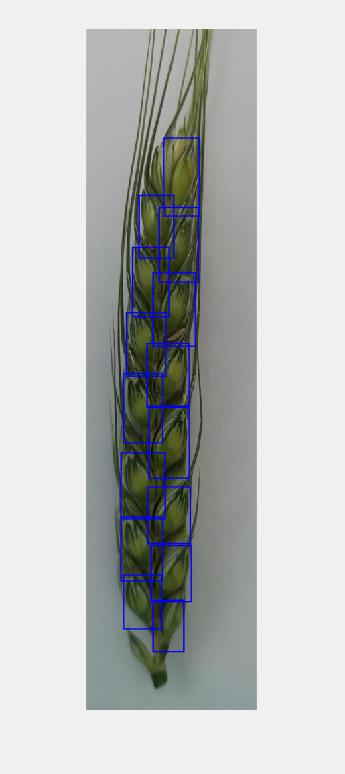

Supplement: Supplementary file 6 [file Data_Sheet_6.ZIP › 7. Detection results/Liangxing 99/3192MTL.jpg]

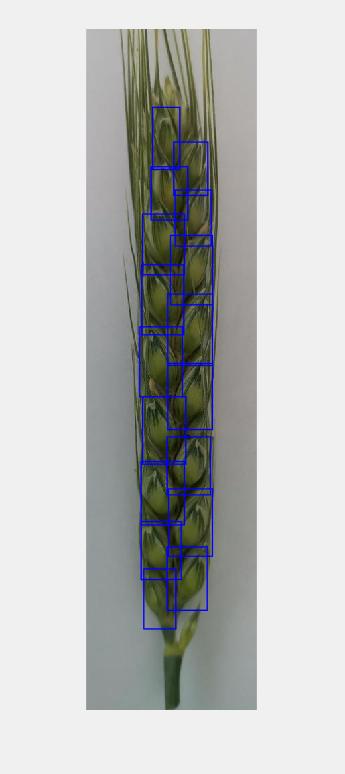

Supplement: Supplementary file 6 [file Data_Sheet_6.ZIP › 7. Detection results/Liangxing 99/3194MTL.jpg]

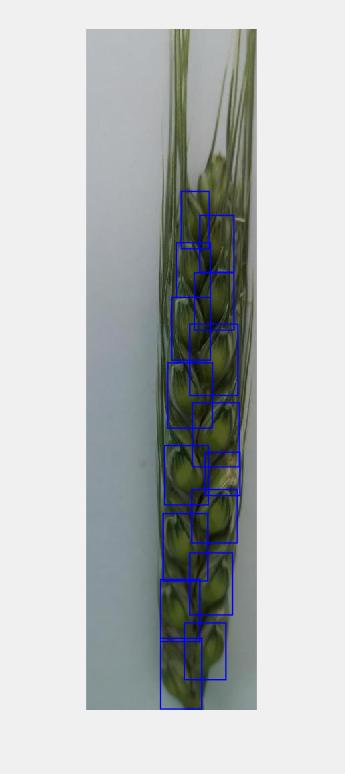

Supplement: Supplementary file 6 [file Data_Sheet_6.ZIP › 7. Detection results/Liangxing 99/3196MTL.jpg]

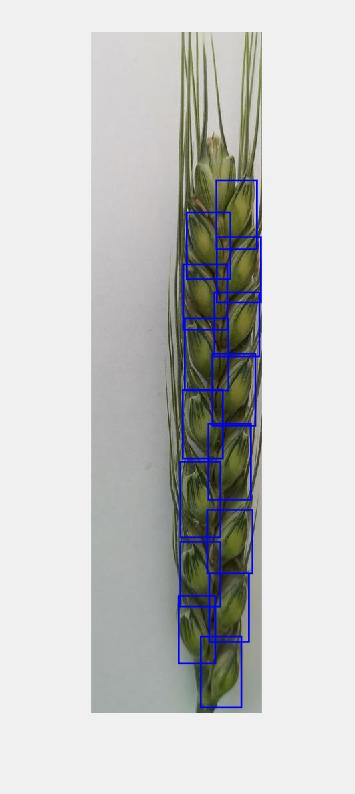

Supplement: Supplementary file 6 [file Data_Sheet_6.ZIP › 7. Detection results/Liangxing 99/3197MTL.jpg]

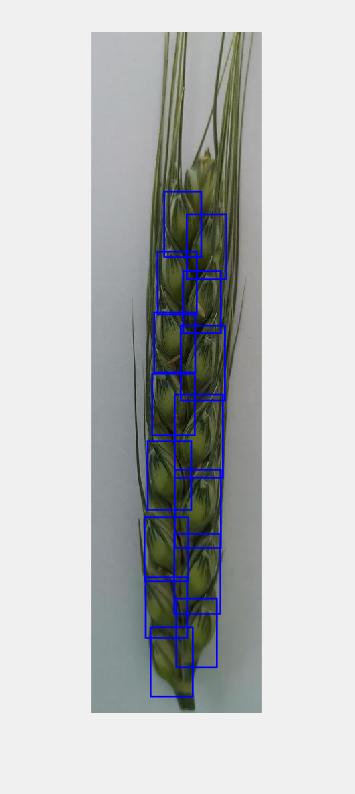

Supplement: Supplementary file 6 [file Data_Sheet_6.ZIP › 7. Detection results/Liangxing 99/3198MTL.jpg]

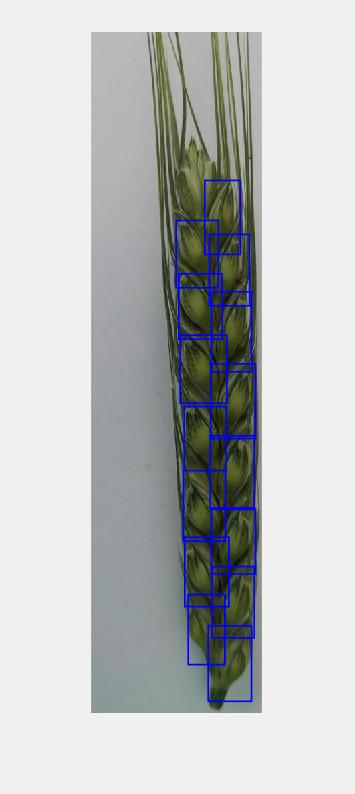

Supplement: Supplementary file 6 [file Data_Sheet_6.ZIP › 7. Detection results/Liangxing 99/3199MTL.jpg]

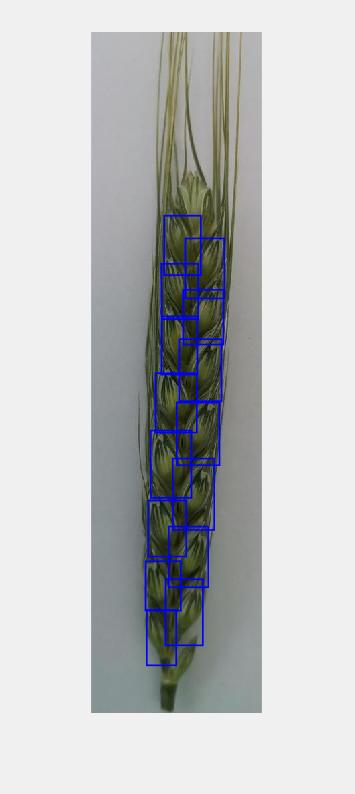

Supplement: Supplementary file 6 [file Data_Sheet_6.ZIP › 7. Detection results/Liangxing 99/3200MTL.jpg]

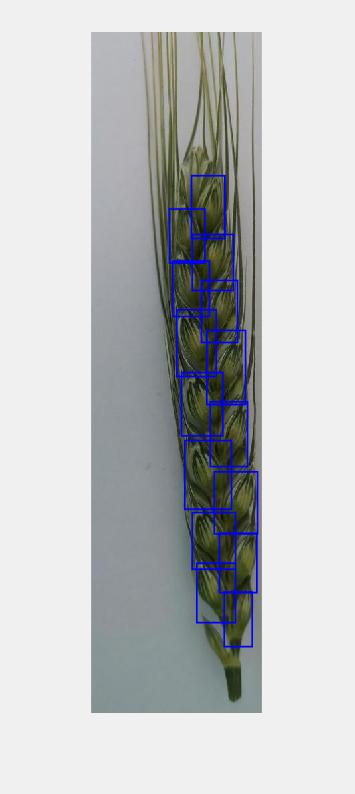

Supplement: Supplementary file 6 [file Data_Sheet_6.ZIP › 7. Detection results/Liangxing 99/3201MTL.jpg]

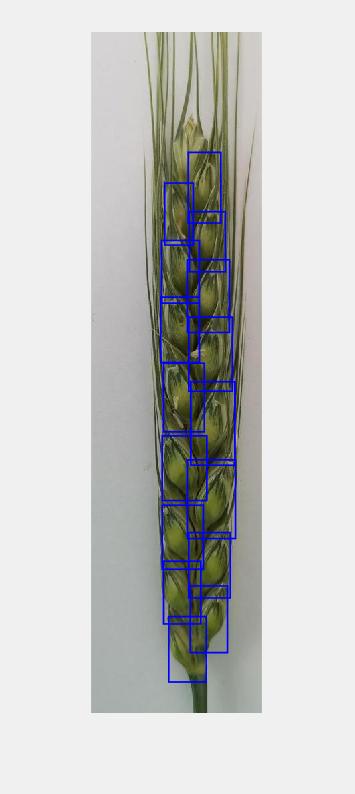

Supplement: Supplementary file 6 [file Data_Sheet_6.ZIP › 7. Detection results/Liangxing 99/3202MTL.jpg]

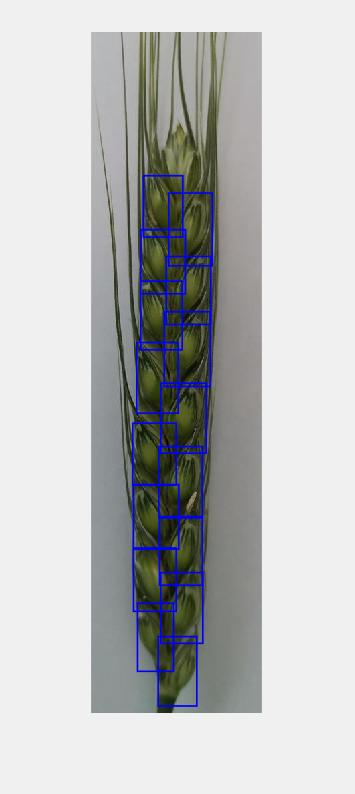

Supplement: Supplementary file 6 [file Data_Sheet_6.ZIP › 7. Detection results/Liangxing 99/3203MTL.jpg]

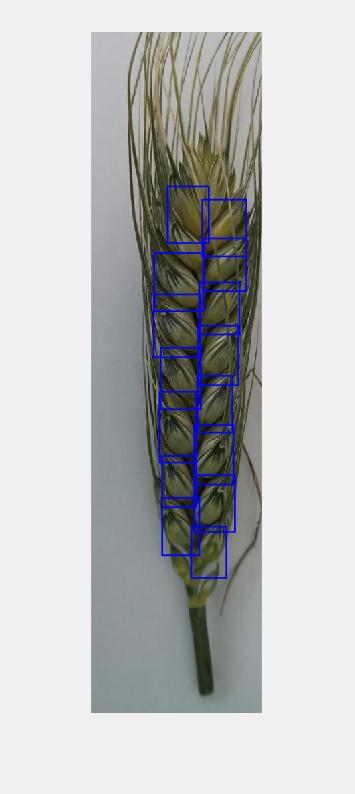

Supplement: Supplementary file 6 [file Data_Sheet_6.ZIP › 7. Detection results/Liangxing 99/3207MTL.jpg]

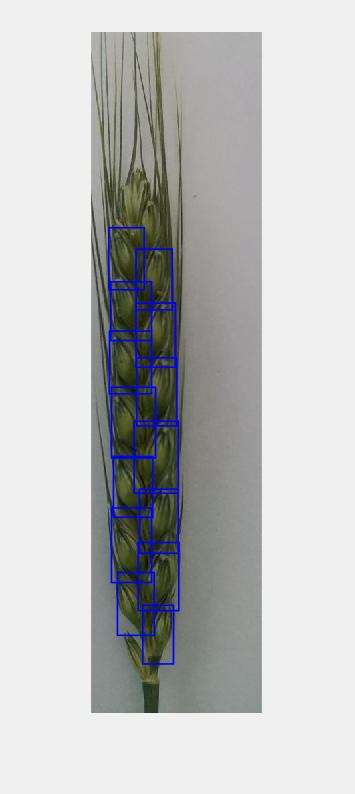

Supplement: Supplementary file 6 [file Data_Sheet_6.ZIP › 7. Detection results/Liangxing 99/3210MTL.jpg]

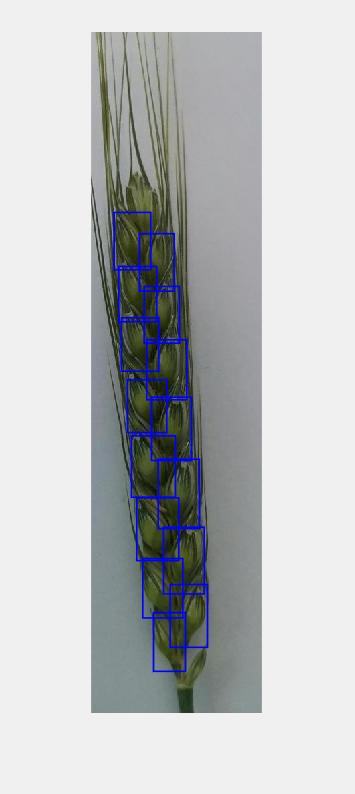

Supplement: Supplementary file 6 [file Data_Sheet_6.ZIP › 7. Detection results/Liangxing 99/3211MTL.jpg]

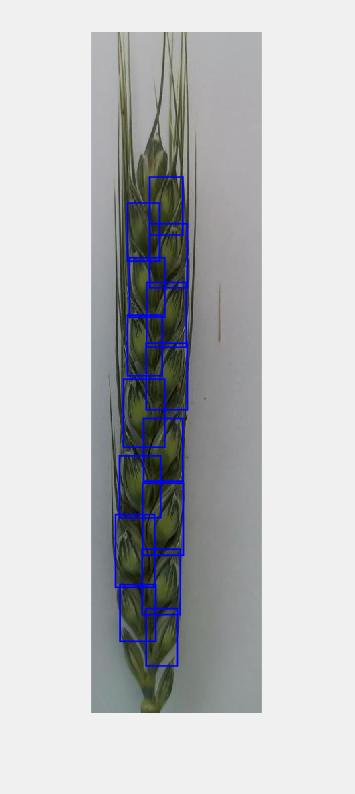

Supplement: Supplementary file 6 [file Data_Sheet_6.ZIP › 7. Detection results/Liangxing 99/3213MTL.jpg]

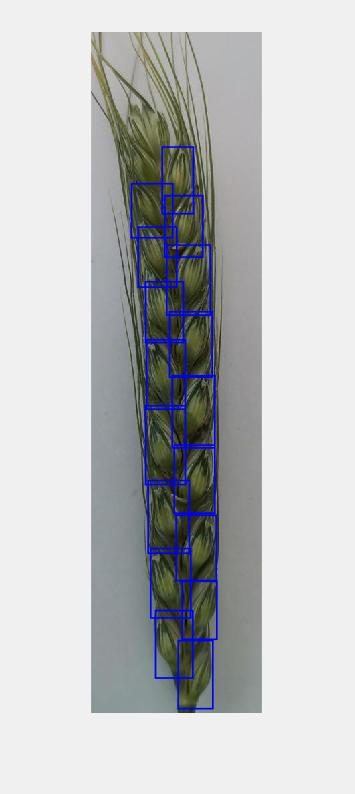

Supplement: Supplementary file 6 [file Data_Sheet_6.ZIP › 7. Detection results/Liangxing 99/3214MTL.jpg]

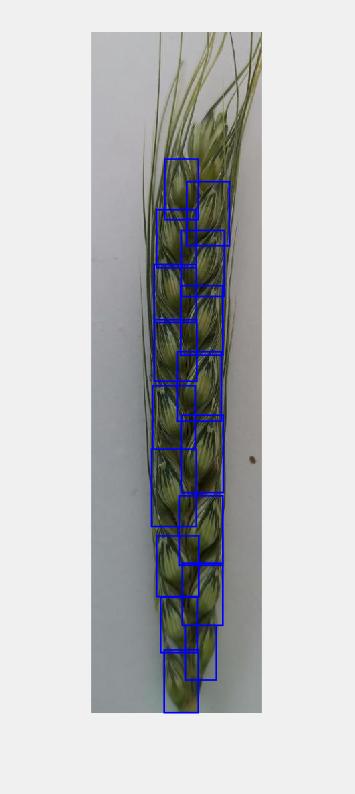

Supplement: Supplementary file 6 [file Data_Sheet_6.ZIP › 7. Detection results/Liangxing 99/3215MTL.jpg]

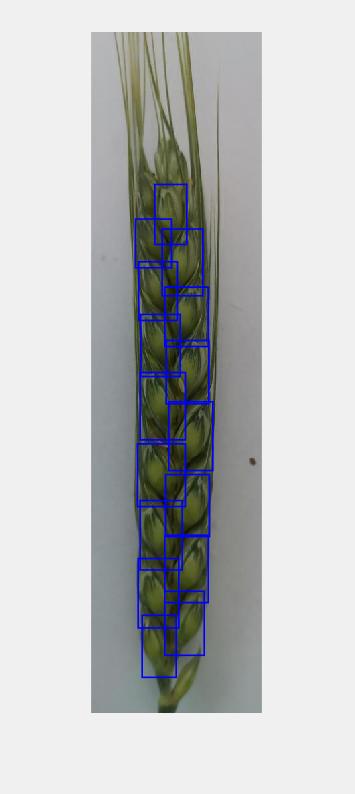

Supplement: Supplementary file 6 [file Data_Sheet_6.ZIP › 7. Detection results/Liangxing 99/3216MTL.jpg]

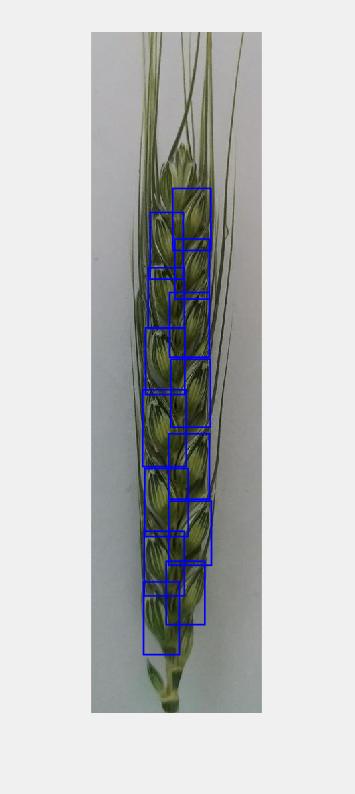

Supplement: Supplementary file 6 [file Data_Sheet_6.ZIP › 7. Detection results/Liangxing 99/3218MTL.jpg]

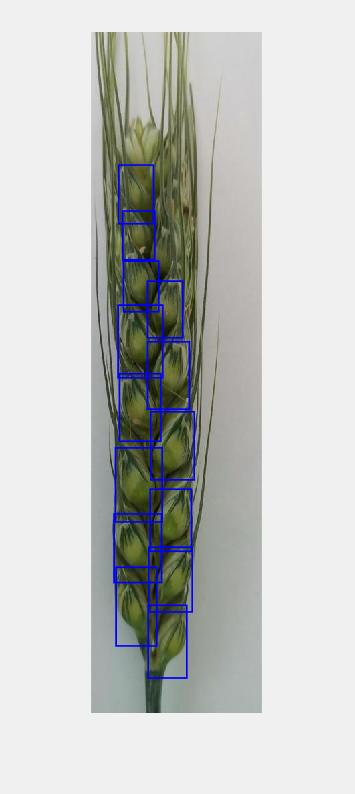

Supplement: Supplementary file 6 [file Data_Sheet_6.ZIP › 7. Detection results/Liangxing 99/3220MTL.jpg]

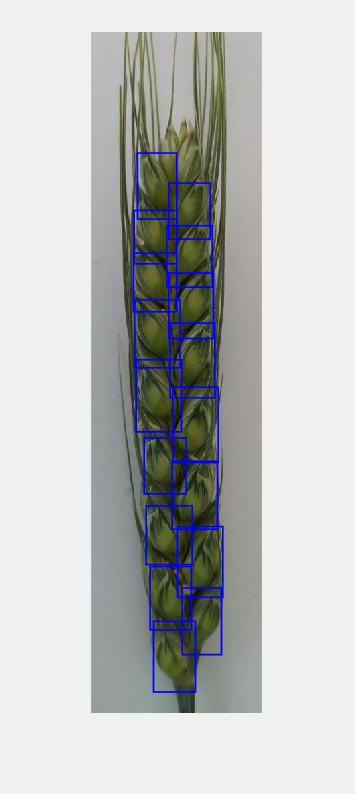

Supplement: Supplementary file 6 [file Data_Sheet_6.ZIP › 7. Detection results/Liangxing 99/3221MTL.jpg]

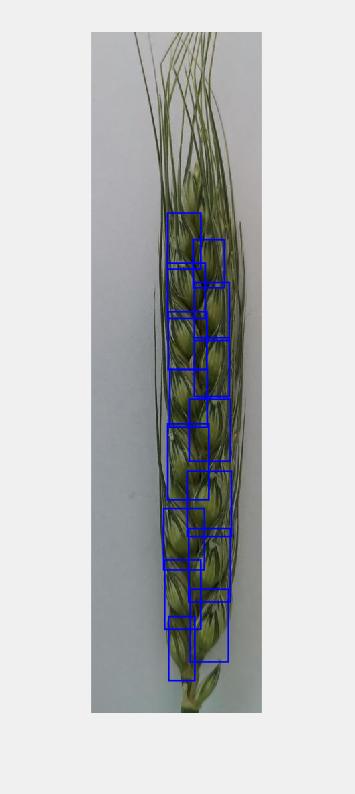

Supplement: Supplementary file 6 [file Data_Sheet_6.ZIP › 7. Detection results/Liangxing 99/3224MTL.jpg]

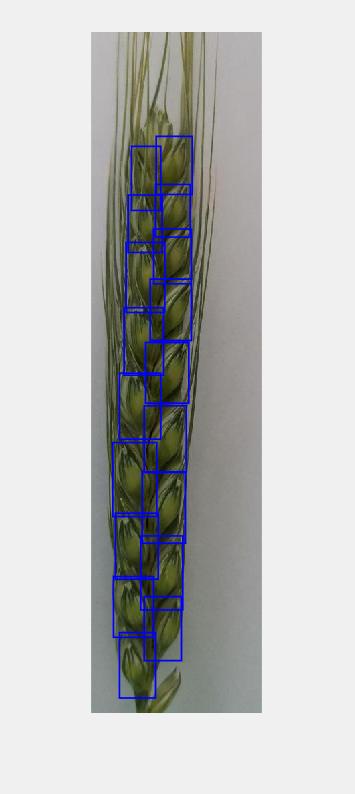

Supplement: Supplementary file 6 [file Data_Sheet_6.ZIP › 7. Detection results/Liangxing 99/3228MTL.jpg]

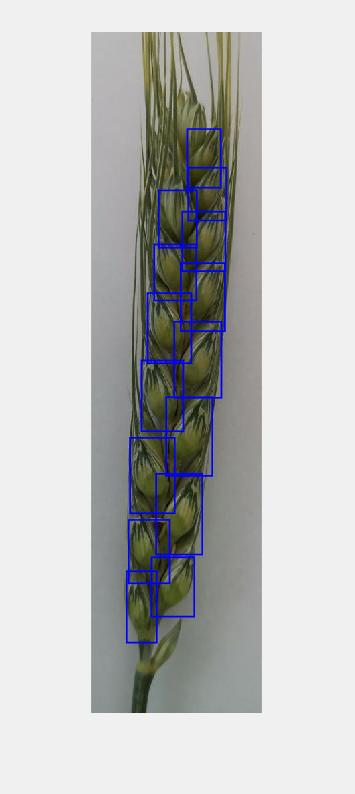

Supplement: Supplementary file 6 [file Data_Sheet_6.ZIP › 7. Detection results/Liangxing 99/3229MTL.jpg]

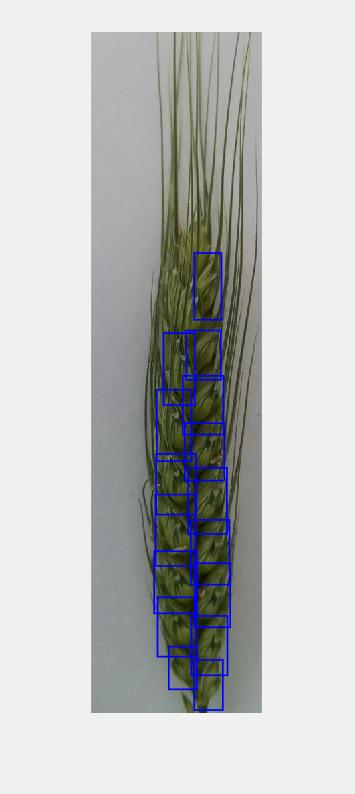

Supplement: Supplementary file 6 [file Data_Sheet_6.ZIP › 7. Detection results/Liangxing 99/3232MTL.jpg]

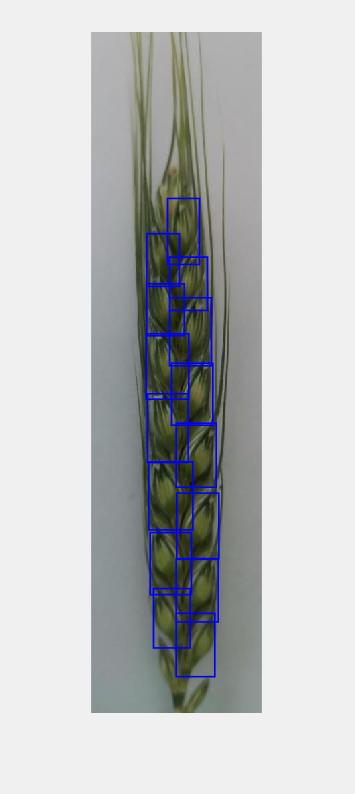

Supplement: Supplementary file 6 [file Data_Sheet_6.ZIP › 7. Detection results/Liangxing 99/3234MTL.jpg]

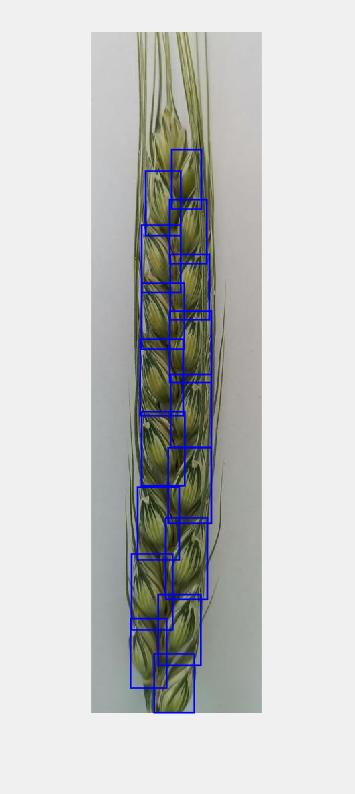

Supplement: Supplementary file 6 [file Data_Sheet_6.ZIP › 7. Detection results/Liangxing 99/3236MTL.jpg]

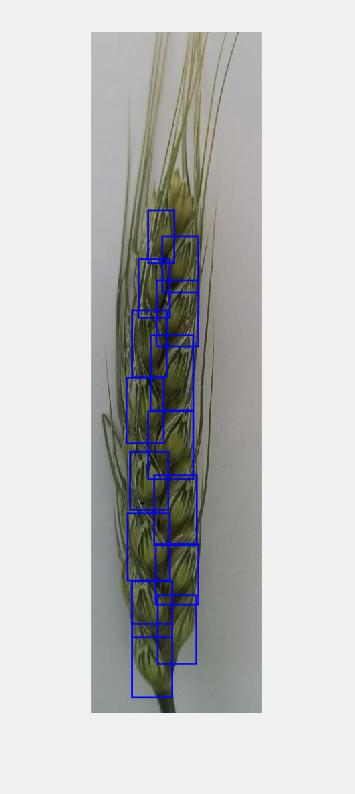

Supplement: Supplementary file 6 [file Data_Sheet_6.ZIP › 7. Detection results/Liangxing 99/3237MTL.jpg]

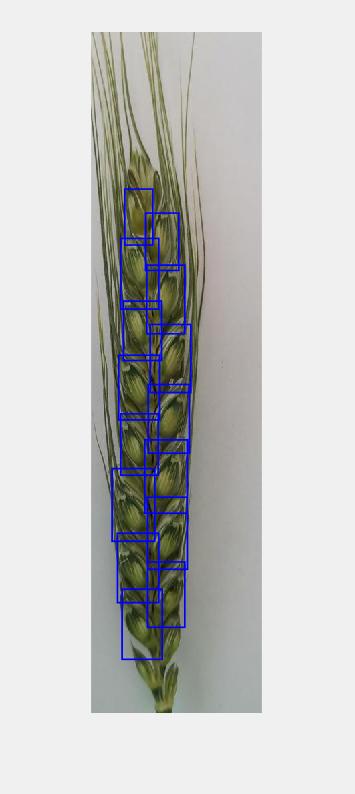

Supplement: Supplementary file 6 [file Data_Sheet_6.ZIP › 7. Detection results/Liangxing 99/3239MTL.jpg]

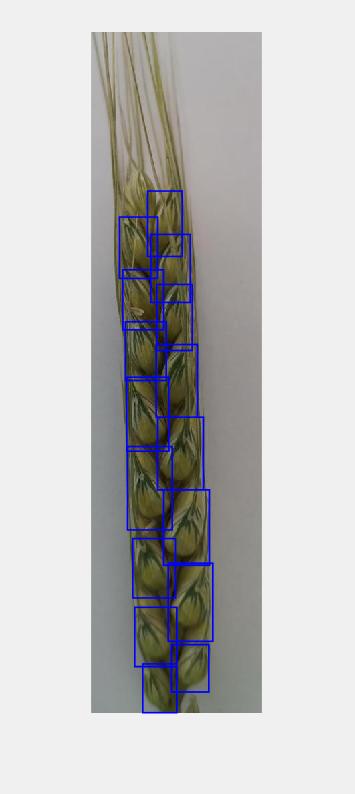

Supplement: Supplementary file 6 [file Data_Sheet_6.ZIP › 7. Detection results/Liangxing 99/3243MTL.jpg]

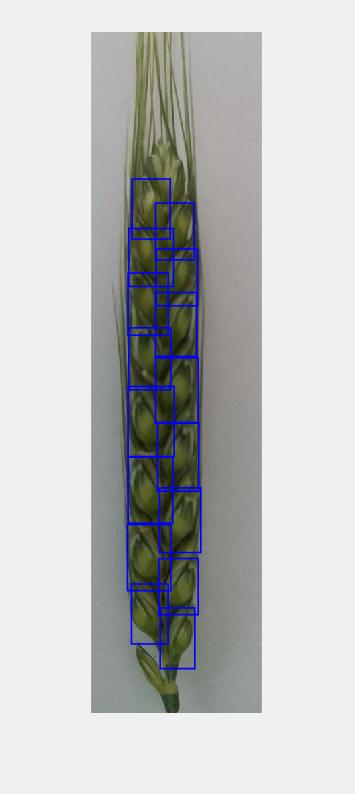

Supplement: Supplementary file 6 [file Data_Sheet_6.ZIP › 7. Detection results/Liangxing 99/3244MTL.jpg]

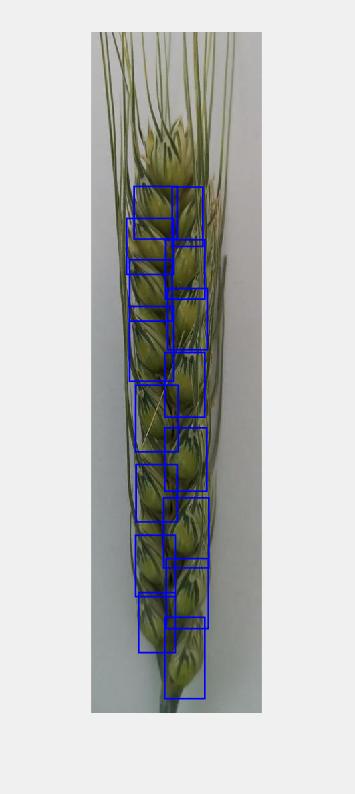

Supplement: Supplementary file 6 [file Data_Sheet_6.ZIP › 7. Detection results/Liangxing 99/3246MTL.jpg]

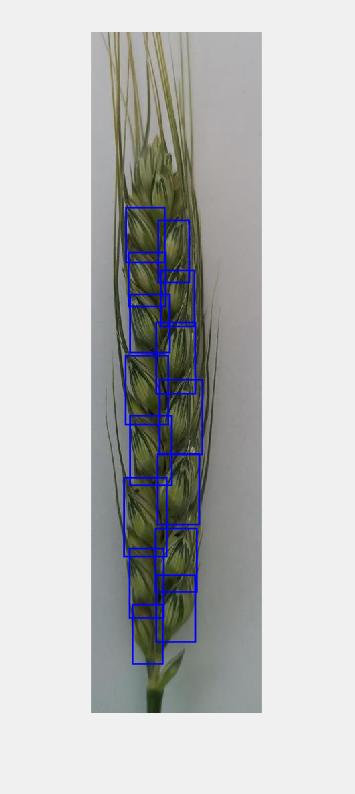

Supplement: Supplementary file 6 [file Data_Sheet_6.ZIP › 7. Detection results/Liangxing 99/3250MTL.jpg]

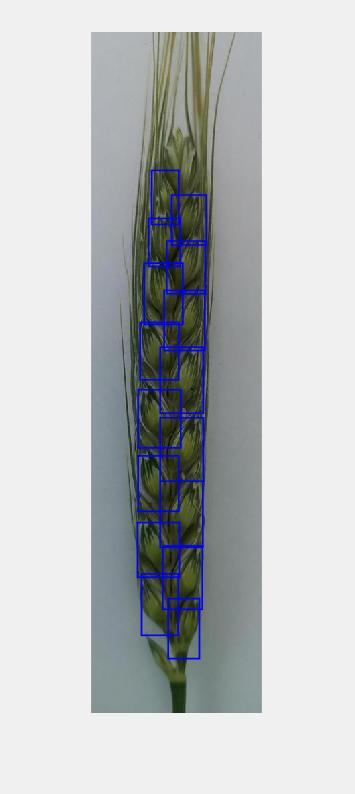

Supplement: Supplementary file 6 [file Data_Sheet_6.ZIP › 7. Detection results/Liangxing 99/3251MTL.jpg]

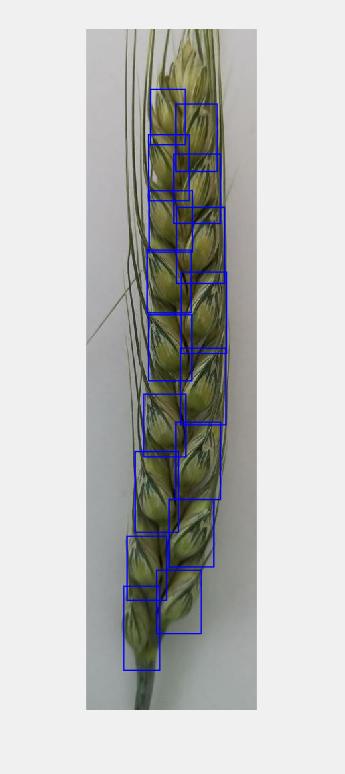

Supplement: Supplementary file 6 [file Data_Sheet_6.ZIP › 7. Detection results/Liangxing 99/3254MTL.jpg]

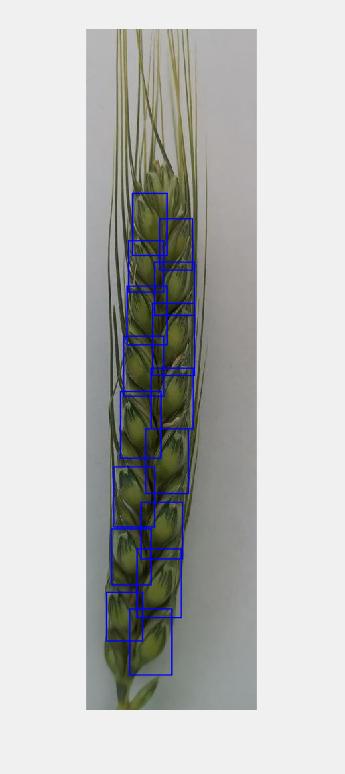

Supplement: Supplementary file 6 [file Data_Sheet_6.ZIP › 7. Detection results/Liangxing 99/3255MTL.jpg]

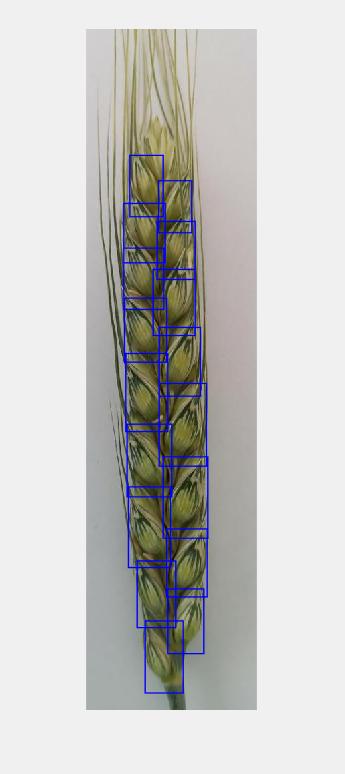

Supplement: Supplementary file 6 [file Data_Sheet_6.ZIP › 7. Detection results/Liangxing 99/3256MTL.jpg]

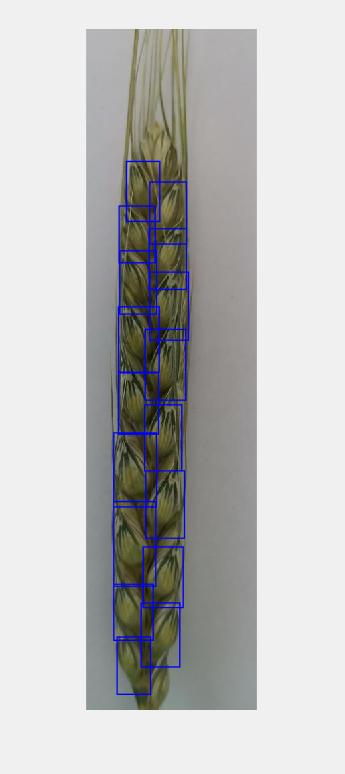

Supplement: Supplementary file 6 [file Data_Sheet_6.ZIP › 7. Detection results/Liangxing 99/3259MTL.jpg]

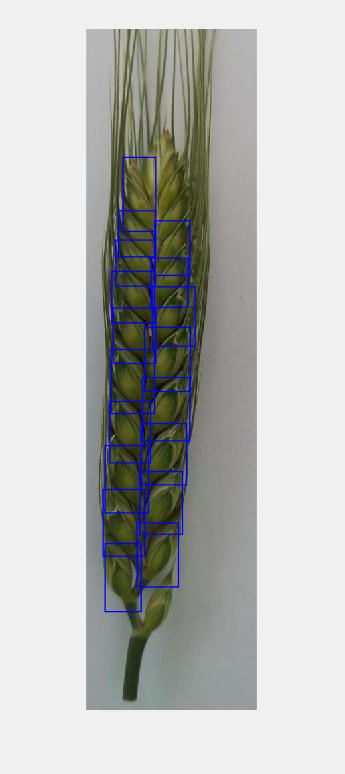

Supplement: Supplementary file 6 [file Data_Sheet_6.ZIP › 7. Detection results/Liangxing 99/3261MTL.jpg]

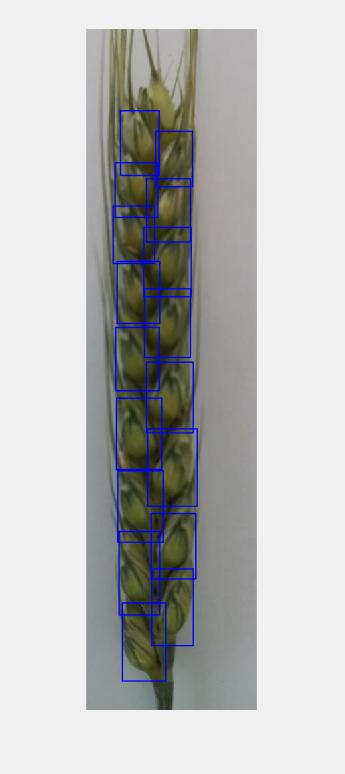

Supplement: Supplementary file 6 [file Data_Sheet_6.ZIP › 7. Detection results/Liangxing 99/3267MTL.jpg]

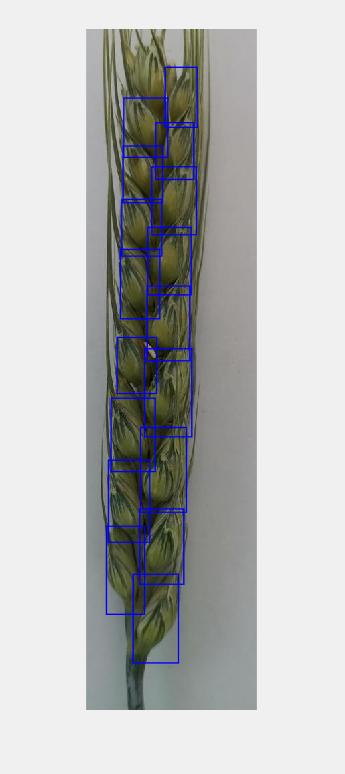

Supplement: Supplementary file 6 [file Data_Sheet_6.ZIP › 7. Detection results/Liangxing 99/3268MTL.jpg]

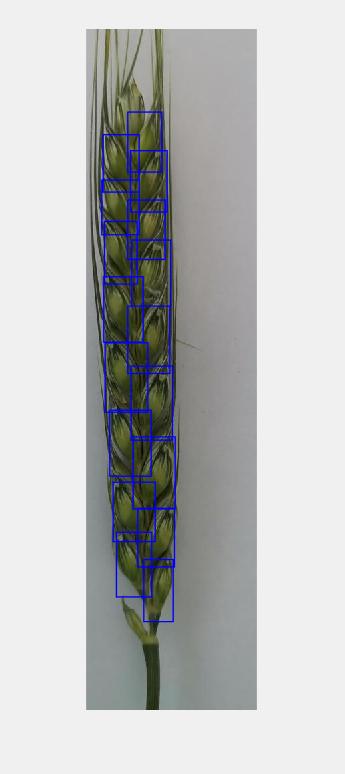

Supplement: Supplementary file 6 [file Data_Sheet_6.ZIP › 7. Detection results/Liangxing 99/3269MTL.jpg]

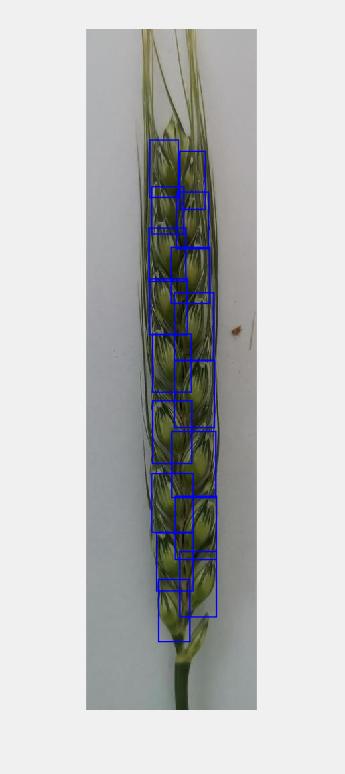

Supplement: Supplementary file 6 [file Data_Sheet_6.ZIP › 7. Detection results/Liangxing 99/3270MTL.jpg]

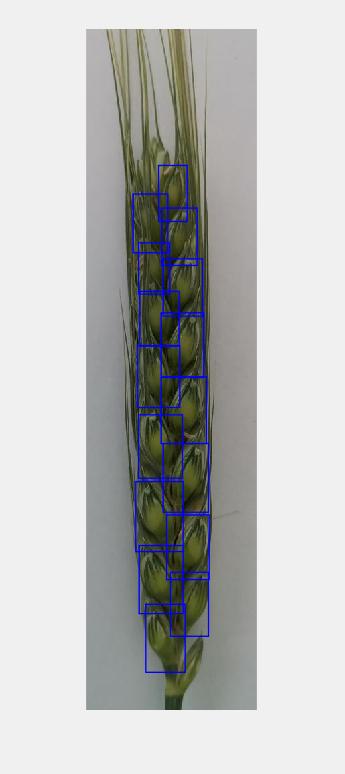

Supplement: Supplementary file 6 [file Data_Sheet_6.ZIP › 7. Detection results/Liangxing 99/3273MTL.jpg]

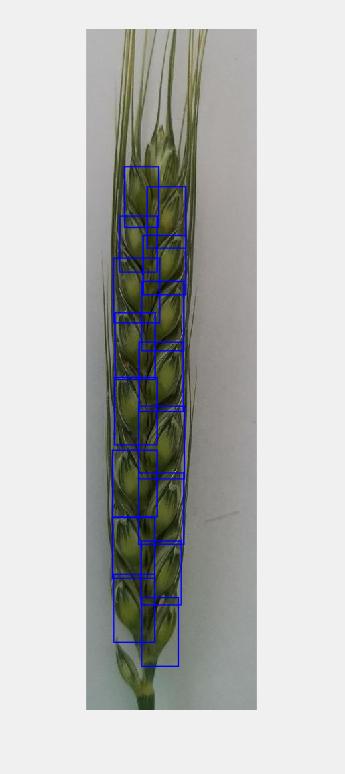

Supplement: Supplementary file 6 [file Data_Sheet_6.ZIP › 7. Detection results/Liangxing 99/3274MTL.jpg]

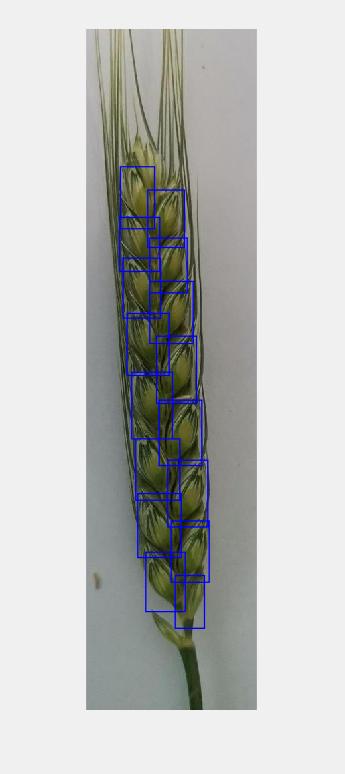

Supplement: Supplementary file 6 [file Data_Sheet_6.ZIP › 7. Detection results/Liangxing 99/3284MTL.jpg]

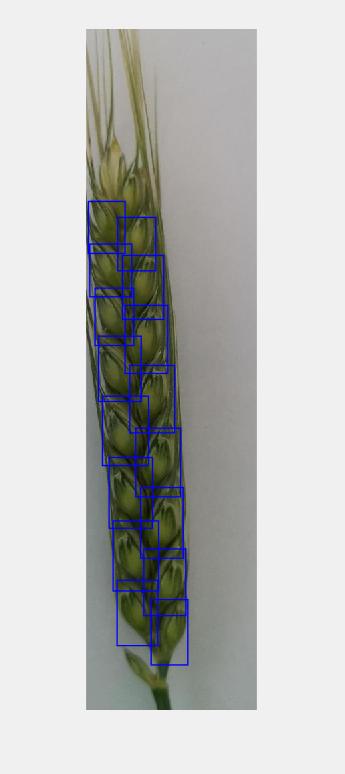

Supplement: Supplementary file 6 [file Data_Sheet_6.ZIP › 7. Detection results/Liangxing 99/3287MTL.jpg]

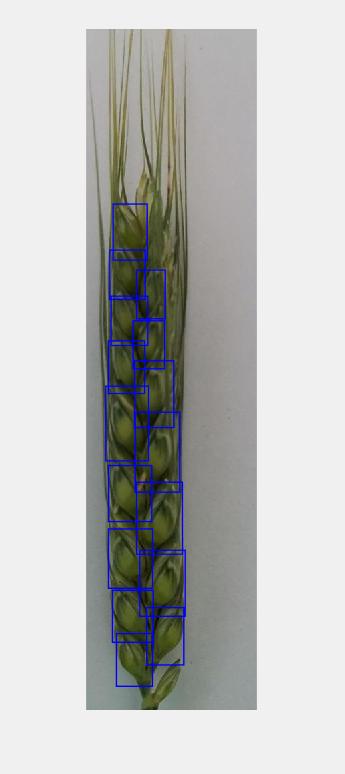

Supplement: Supplementary file 6 [file Data_Sheet_6.ZIP › 7. Detection results/Liangxing 99/3288MTL.jpg]

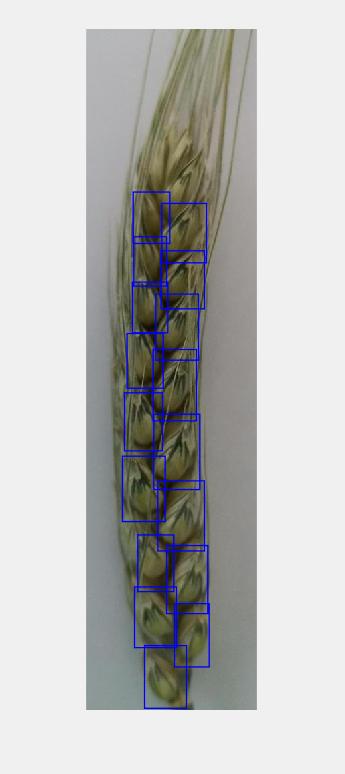

Supplement: Supplementary file 6 [file Data_Sheet_6.ZIP › 7. Detection results/Liangxing 99/3289MTL.jpg]

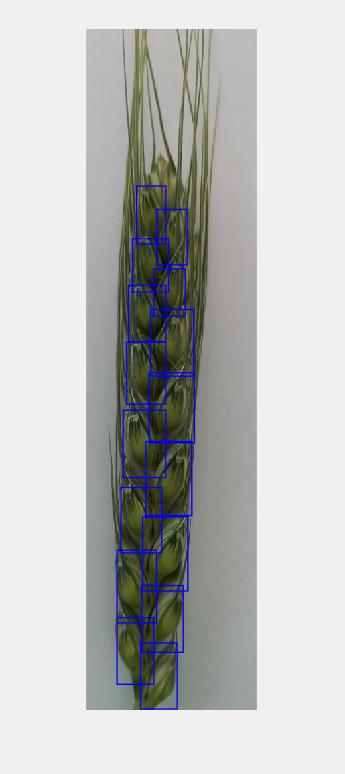

Supplement: Supplementary file 6 [file Data_Sheet_6.ZIP › 7. Detection results/Liangxing 99/3290MTL.jpg]

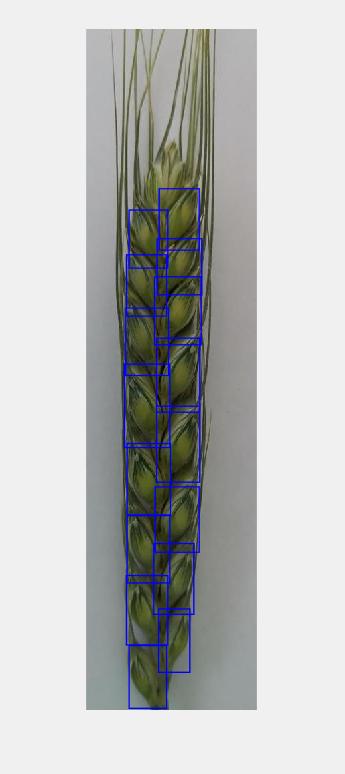

Supplement: Supplementary file 6 [file Data_Sheet_6.ZIP › 7. Detection results/Liangxing 99/3291MTL.jpg]

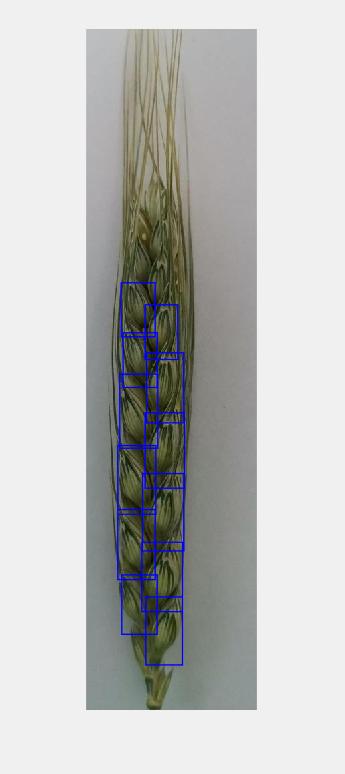

Supplement: Supplementary file 6 [file Data_Sheet_6.ZIP › 7. Detection results/Liangxing 99/3292MTL.jpg]

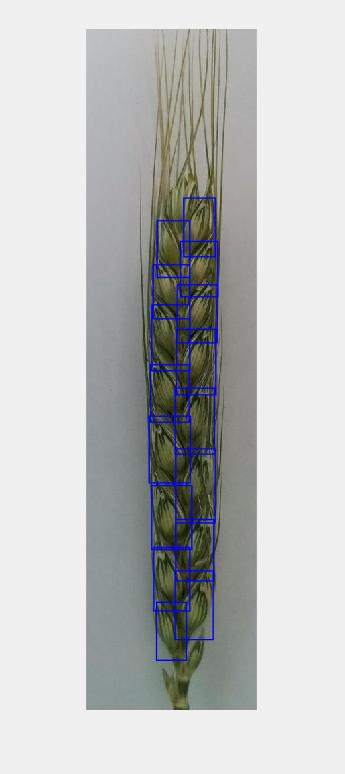

Supplement: Supplementary file 6 [file Data_Sheet_6.ZIP › 7. Detection results/Liangxing 99/3293MTL.jpg]

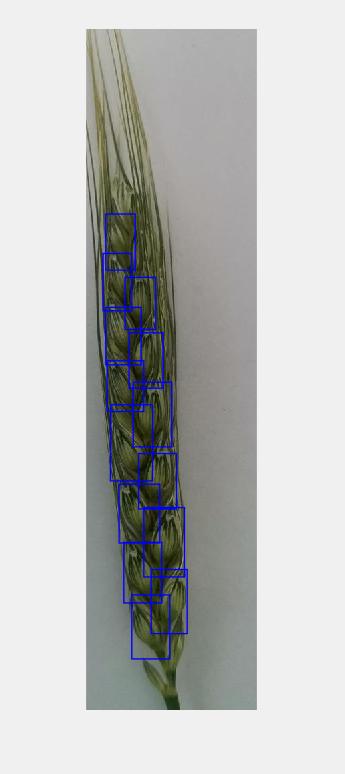

Supplement: Supplementary file 6 [file Data_Sheet_6.ZIP › 7. Detection results/Liangxing 99/3296MTL.jpg]

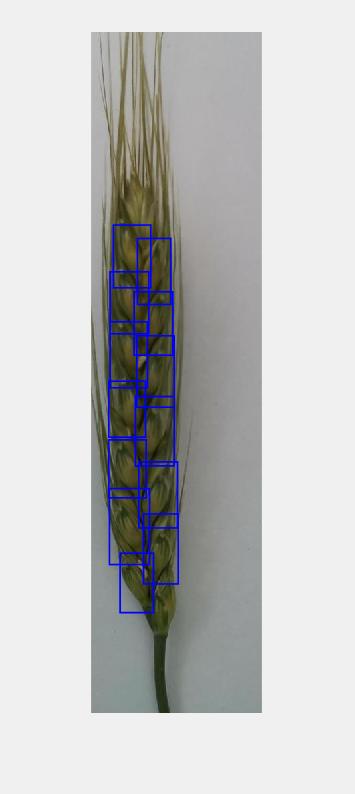

Supplement: Supplementary file 6 [file Data_Sheet_6.ZIP › 7. Detection results/Liangxing 99/3312MTL.jpg]

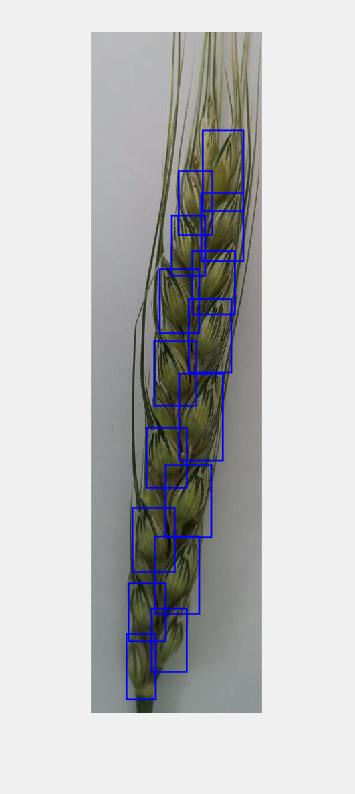

Supplement: Supplementary file 6 [file Data_Sheet_6.ZIP › 7. Detection results/Liangxing 99/3314MTL.jpg]

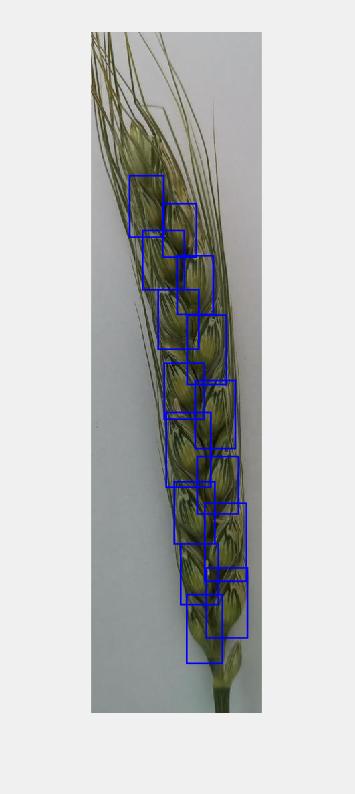

Supplement: Supplementary file 6 [file Data_Sheet_6.ZIP › 7. Detection results/Liangxing 99/3315MTL.jpg]

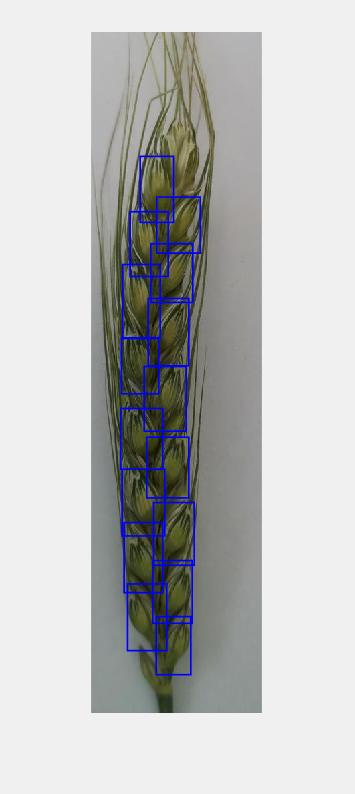

Supplement: Supplementary file 6 [file Data_Sheet_6.ZIP › 7. Detection results/Liangxing 99/3316MTL.jpg]

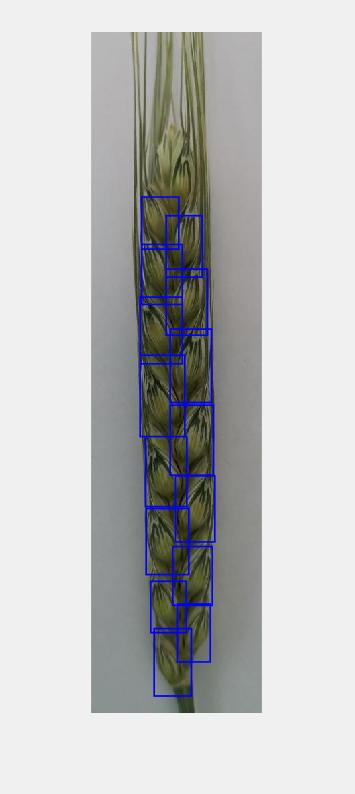

Supplement: Supplementary file 6 [file Data_Sheet_6.ZIP › 7. Detection results/Liangxing 99/3319MTL.jpg]

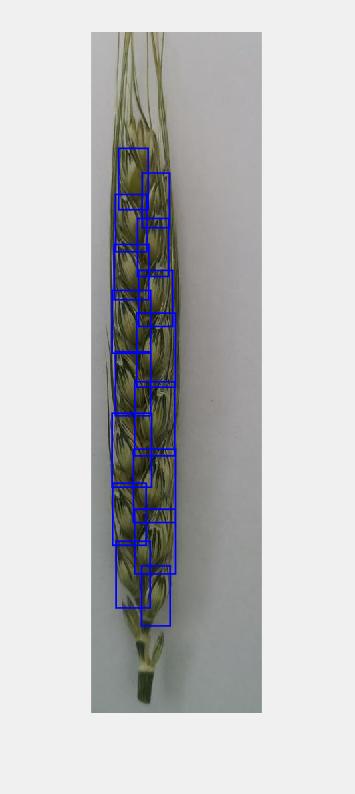

Supplement: Supplementary file 6 [file Data_Sheet_6.ZIP › 7. Detection results/Liangxing 99/3321MTL.jpg]

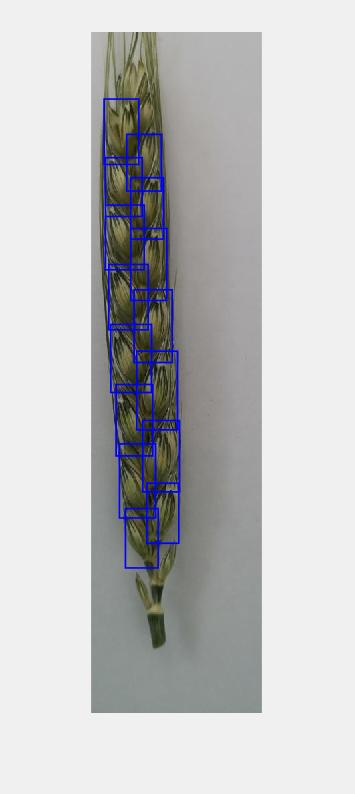

Supplement: Supplementary file 6 [file Data_Sheet_6.ZIP › 7. Detection results/Liangxing 99/3322MTL.jpg]

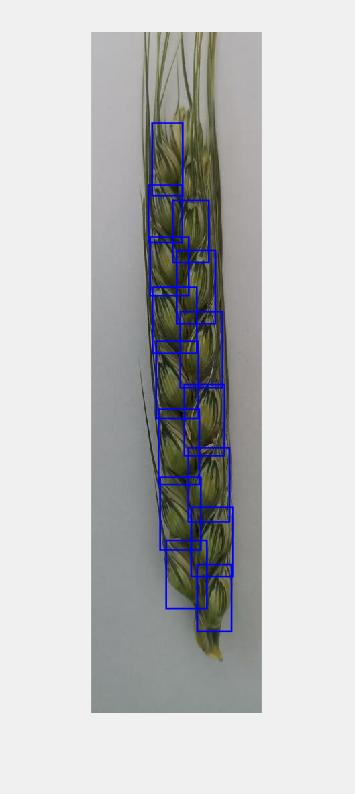

Supplement: Supplementary file 6 [file Data_Sheet_6.ZIP › 7. Detection results/Liangxing 99/3323MTL.jpg]

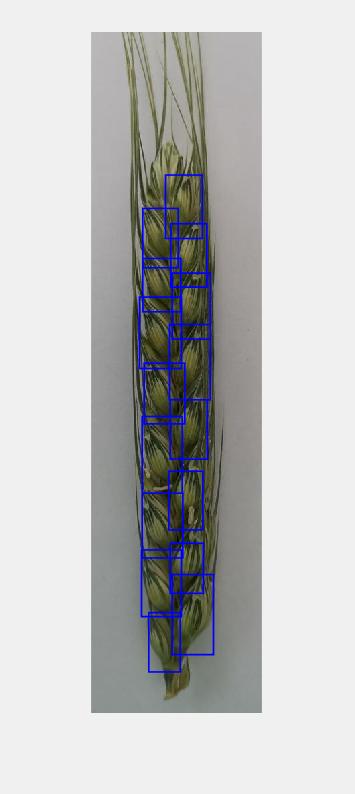

Supplement: Supplementary file 6 [file Data_Sheet_6.ZIP › 7. Detection results/Liangxing 99/3324MTL.jpg]

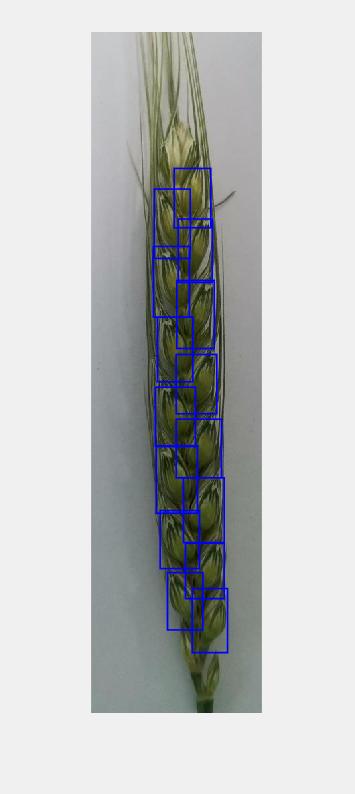

Supplement: Supplementary file 6 [file Data_Sheet_6.ZIP › 7. Detection results/Liangxing 99/3332MTL.jpg]

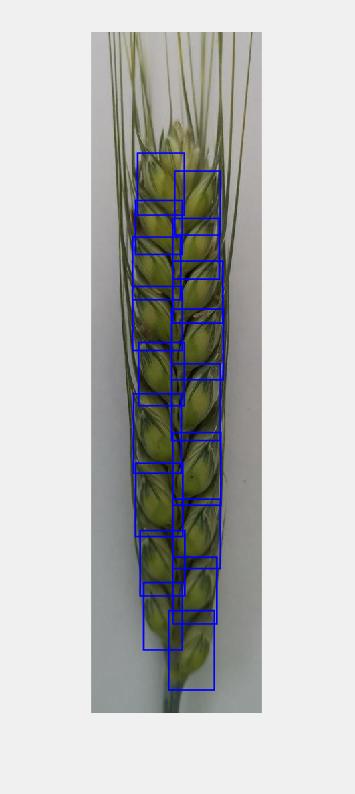

Supplement: Supplementary file 6 [file Data_Sheet_6.ZIP › 7. Detection results/Liangxing 99/3336MTL.jpg]

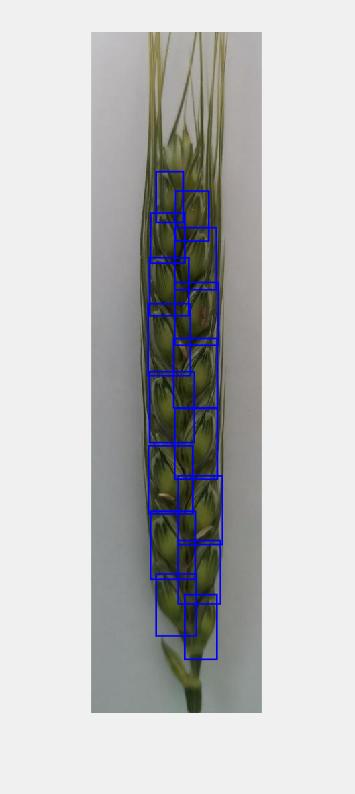

Supplement: Supplementary file 6 [file Data_Sheet_6.ZIP › 7. Detection results/Liangxing 99/3337MTL.jpg]

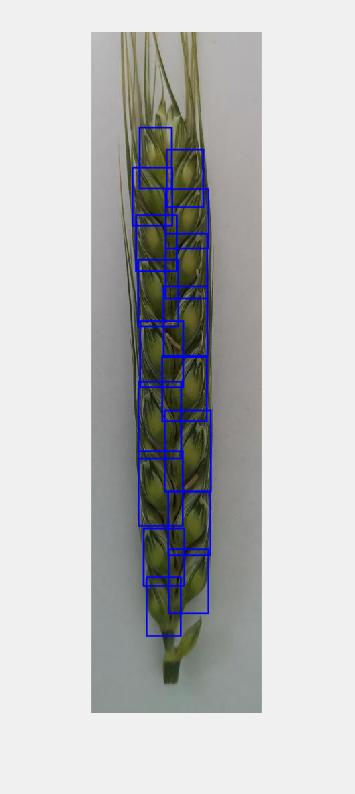

Supplement: Supplementary file 6 [file Data_Sheet_6.ZIP › 7. Detection results/Liangxing 99/3338MTL.jpg]

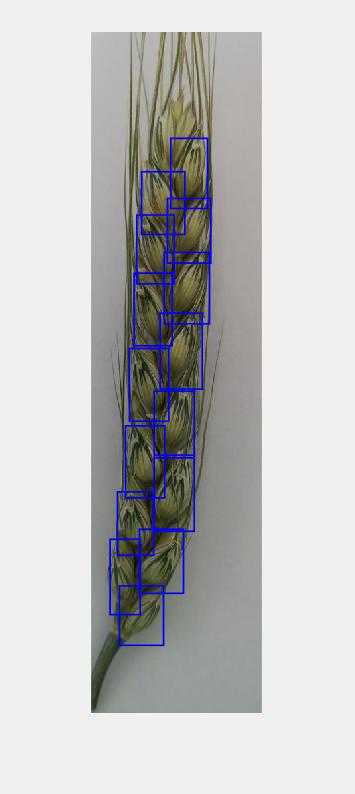

Supplement: Supplementary file 6 [file Data_Sheet_6.ZIP › 7. Detection results/Liangxing 99/3339MTL.jpg]

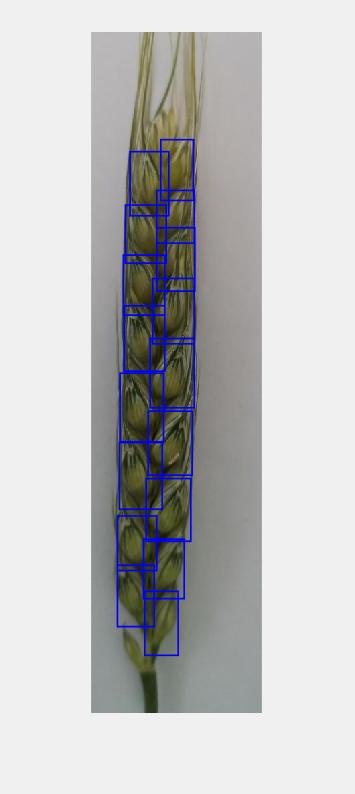

Supplement: Supplementary file 6 [file Data_Sheet_6.ZIP › 7. Detection results/Liangxing 99/3341MTL.jpg]

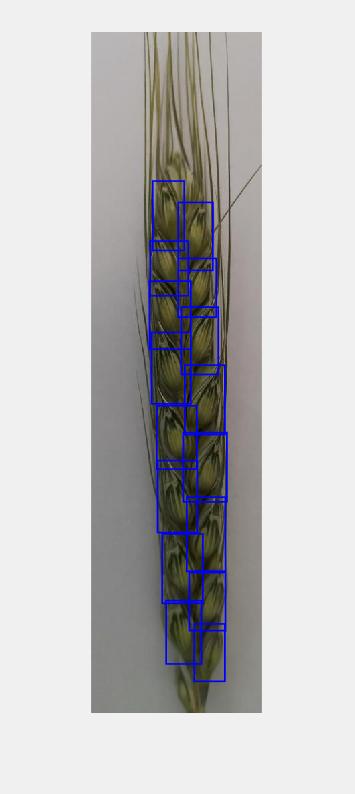

Supplement: Supplementary file 6 [file Data_Sheet_6.ZIP › 7. Detection results/Liangxing 99/3342MTL.jpg]

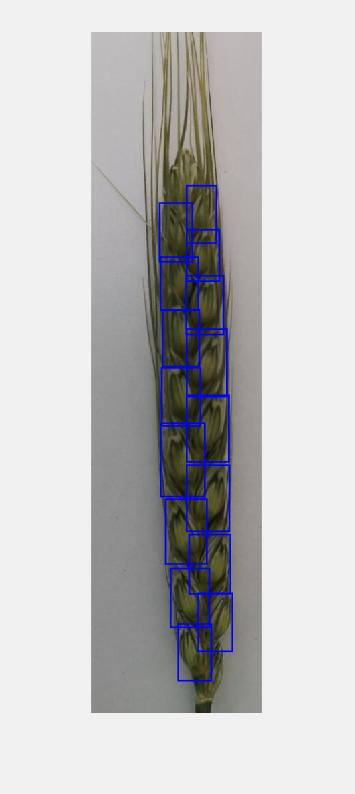

Supplement: Supplementary file 6 [file Data_Sheet_6.ZIP › 7. Detection results/Liangxing 99/3343MTL.jpg]

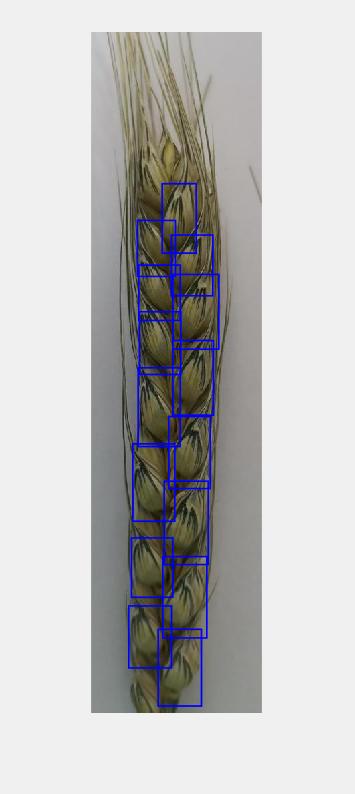

Supplement: Supplementary file 6 [file Data_Sheet_6.ZIP › 7. Detection results/Liangxing 99/3344MTL.jpg]

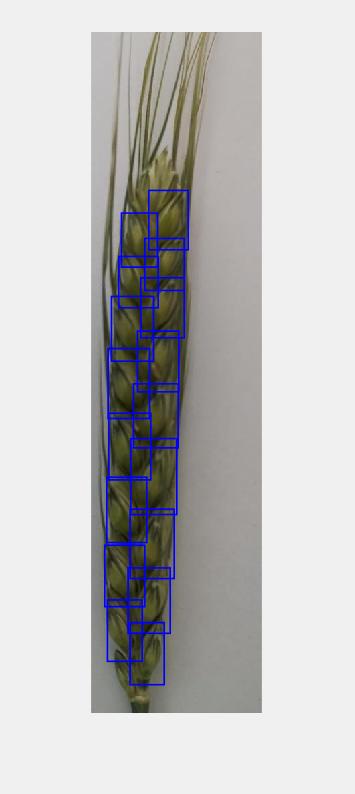

Supplement: Supplementary file 6 [file Data_Sheet_6.ZIP › 7. Detection results/Liangxing 99/3346MTL.jpg]

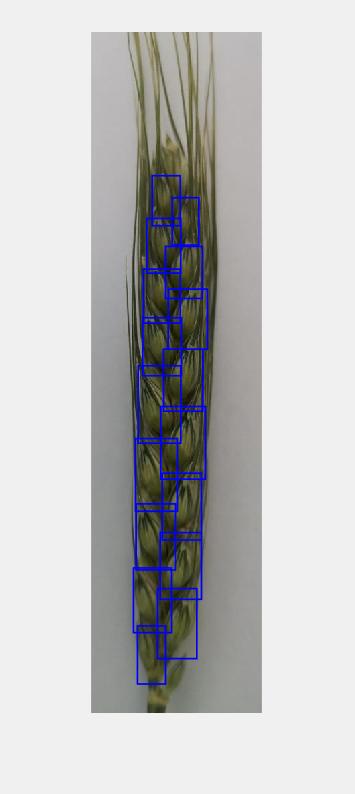

Supplement: Supplementary file 6 [file Data_Sheet_6.ZIP › 7. Detection results/Liangxing 99/3347MTL.jpg]

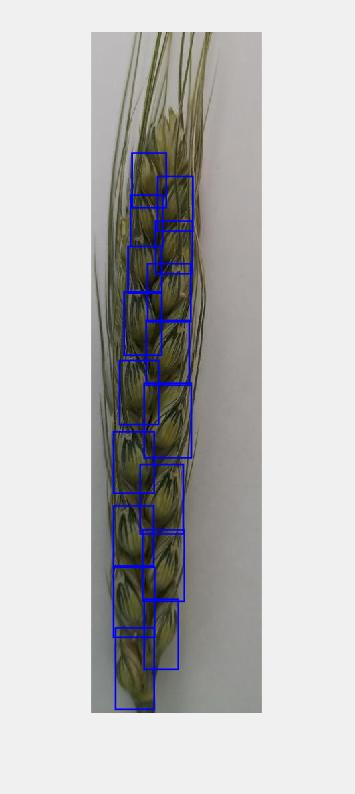

Supplement: Supplementary file 6 [file Data_Sheet_6.ZIP › 7. Detection results/Liangxing 99/3348MTL.jpg]

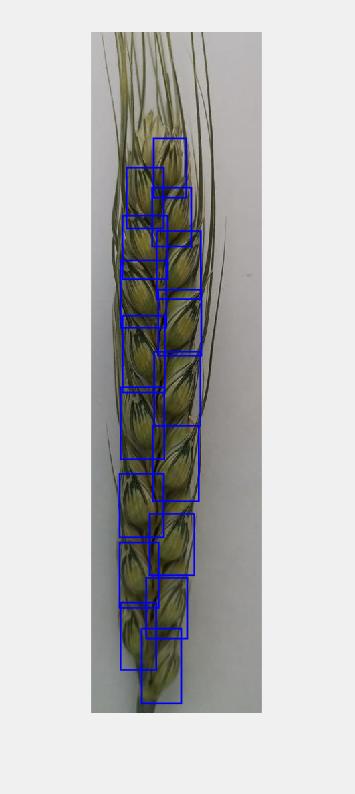

Supplement: Supplementary file 6 [file Data_Sheet_6.ZIP › 7. Detection results/Liangxing 99/3349MTL.jpg]

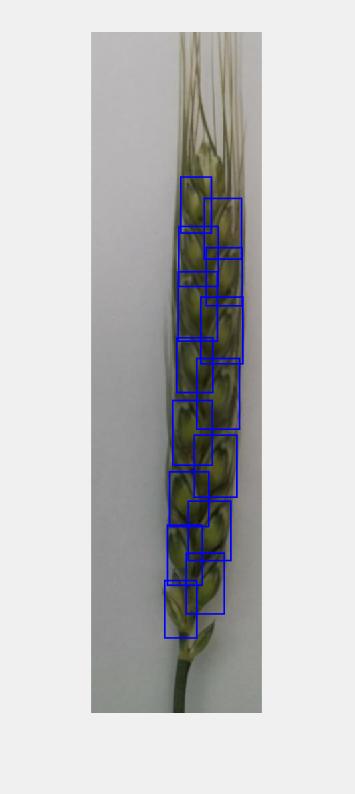

Supplement: Supplementary file 6 [file Data_Sheet_6.ZIP › 7. Detection results/Liangxing 99/3350MTL.jpg]

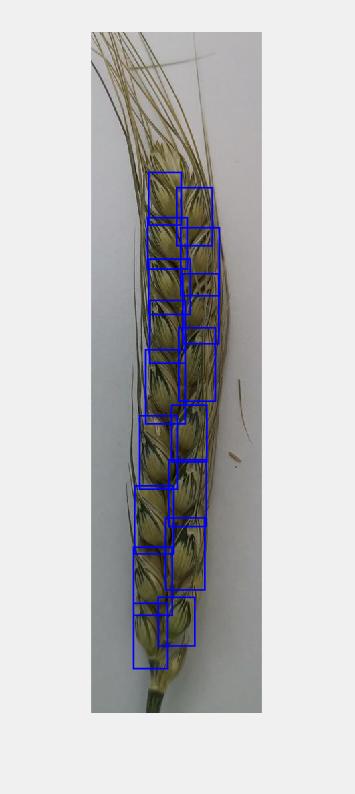

Supplement: Supplementary file 6 [file Data_Sheet_6.ZIP › 7. Detection results/Liangxing 99/3359MTL.jpg]

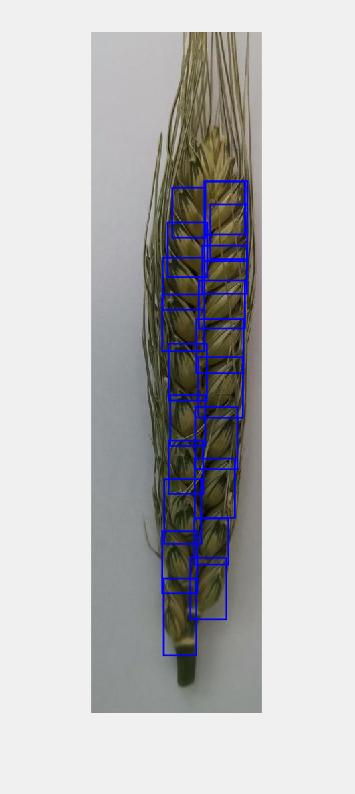

Supplement: Supplementary file 6 [file Data_Sheet_6.ZIP › 7. Detection results/Liangxing 99/3361MTL.jpg]

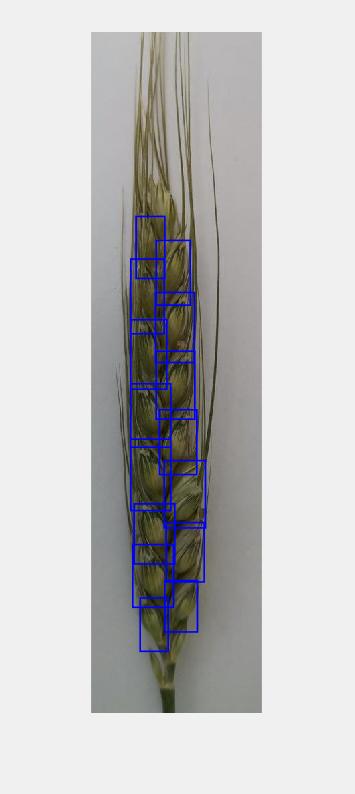

Supplement: Supplementary file 6 [file Data_Sheet_6.ZIP › 7. Detection results/Liangxing 99/3362MTL.jpg]

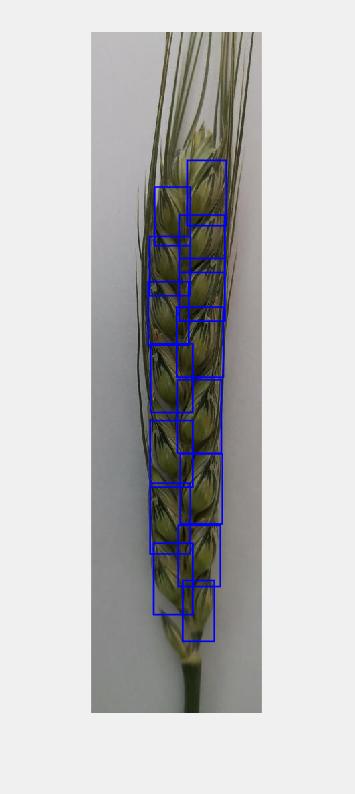

Supplement: Supplementary file 6 [file Data_Sheet_6.ZIP › 7. Detection results/Liangxing 99/3364MTL.jpg]

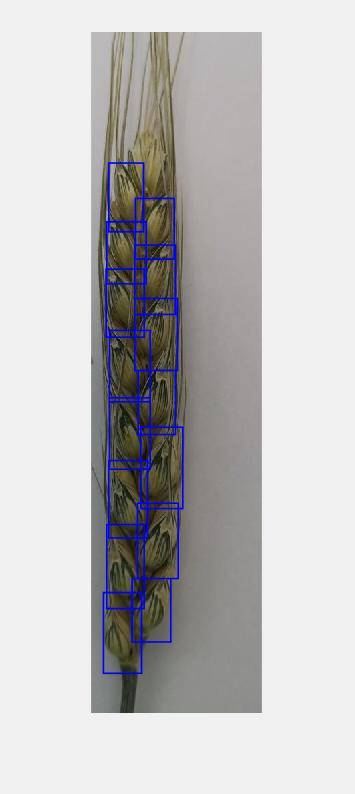

Supplement: Supplementary file 6 [file Data_Sheet_6.ZIP › 7. Detection results/Liangxing 99/3368MTL.jpg]

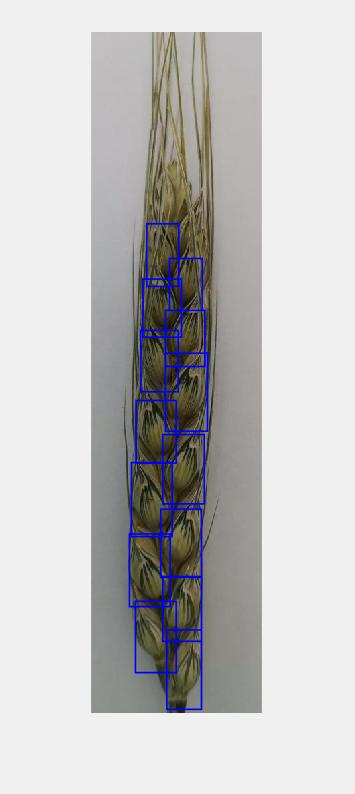

Supplement: Supplementary file 6 [file Data_Sheet_6.ZIP › 7. Detection results/Liangxing 99/3369MTL.jpg]

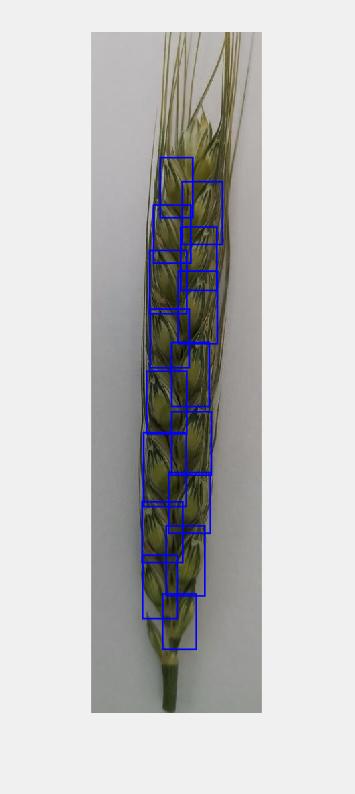

Supplement: Supplementary file 6 [file Data_Sheet_6.ZIP › 7. Detection results/Liangxing 99/3373MTL.jpg]
